# A Generally Applicable Computer Algorithm Based on the Group Additivity Method for the Calculation of Seven Molecular Descriptors: Heat of Combustion, LogP_O/W_, LogS, Refractivity, Polarizability, Toxicity and LogBB of Organic Compounds; Scope and Limits of Applicability

**DOI:** 10.3390/molecules201018279

**Published:** 2015-10-07

**Authors:** Rudolf Naef

**Affiliations:** Department of Chemistry, University of Basel, Basel 4003, Switzerland; E-Mail: rudolf.naef@unibas.ch; Tel.: 41-61-9119273

**Keywords:** heat of combustion, heat of formation, LogP, LogS, molar refractivity, molecular polarizability, toxicity, LogBB, group-additivity method

## Abstract

A generally applicable computer algorithm for the calculation of the seven molecular descriptors heat of combustion, logP_octanol/water_, logS (water solubility), molar refractivity, molecular polarizability, aqueous toxicity (protozoan growth inhibition) and logBB (log (c_blood_/c_brain_)) is presented. The method, an extendable form of the group-additivity method, is based on the complete break-down of the molecules into their constituting atoms and their immediate neighbourhood. The contribution of the resulting atom groups to the descriptor values is calculated using the Gauss-Seidel fitting method, based on experimental data gathered from literature. The plausibility of the method was tested for each descriptor by means of a k-fold cross-validation procedure demonstrating good to excellent predictive power for the former six descriptors and low reliability of logBB predictions. The goodness of fit (Q^2^) and the standard deviation of the 10-fold cross-validation calculation was >0.9999 and 25.2 kJ/mol, respectively, (based on N = 1965 test compounds) for the heat of combustion, 0.9451 and 0.51 (N = 2640) for logP, 0.8838 and 0.74 (N = 1419) for logS, 0.9987 and 0.74 (N = 4045) for the molar refractivity, 0.9897 and 0.77 (N = 308) for the molecular polarizability, 0.8404 and 0.42 (N = 810) for the toxicity and 0.4709 and 0.53 (N = 383) for logBB. The latter descriptor revealing a very low Q^2^ for the test molecules (R^2^ was 0.7068 and standard deviation 0.38 for N = 413 training molecules) is included as an example to show the limits of the group-additivity method. An eighth molecular descriptor, the heat of formation, was indirectly calculated from the heat of combustion data and correlated with published experimental heat of formation data with a correlation coefficient R^2^ of 0.9974 (N = 2031).

## 1. Introduction

The published methods for the calculation of a molecular descriptor, if based on a given set of experimental data for known molecules, usually cannot be generalized, be it that they are based on certain molecular fragment parameters such as bond energies [1,2,3], only applicable for thermodynamic properties, be it that they are founded on simple atom contribution methods [4], referring to the atoms’ properties themselves or on substituents [5], which are also of limited viability. Hence, the goal was to find a method which would overcome all of these limitations and, beyond this, would allow the development of a general computer algorithm for the reliable calculation of as many molecular descriptors as possible which utilises the molecular structures and properties as available from a given compounds database.

The most promising approach was described by Ghose and Crippen for the calculation of the logP_O/W_ values [6,7], where the molecules are broken down into a set of up to 110 atom types, for which the hydrophobicity contribution was calculated from experimental data using the group-additivity model and least-squares technique. Analogously, the authors used this approach for the evaluation of the molar refractivity [8]. The standard fitting procedure for the latter, however, was replaced by a quadratic programming algorithm, arguing that the “physical concept of molar refractivity is the volume of the molecule or atom, which cannot have a negative value”, which is not guaranteed if the standard procedure is applied.

Furthermore, K. J. Miller [9,10] applied the group additivity method for the calculation of the molecular polarizability using atomic hybrid components and atomic hybrid polarizabilites, an approach which differs from the present one in that the type of the neighbourhood atoms is ignored.

Klopman, Wang and Balthasar [11] tried a similar method to Ghose and Crippen’s for the estimation of the aqueous solubility of organic compounds, deriving their own experience on the applicability of the group-additivity method for the calculation of the logP values. Analogously, H. Sun [12] developed a universal group-additivity system for the prediction of logP, solubility logS, logBB (to which will be referred to later) and human intestinal absorption.

Earlier methods for the calculation of the heat of combustion have either been derived from the additivity of bond energies as suggested by Pauling [1], Klages [2] and Wheland [3], or are based on various empirical relations between certain features of a series of molecules, such as the percentage of carbon [13] or hydrogen [14], and their heat of combustion. Further attempts [15] have been made using group contributions, which are based on theoretical assumptions and the “heats of atomization”. Another approach has been chosen by Kharash [16,17] in that his method of calculation depends on the number of electrons in a molecule, multiplied by the combustion value of each electron and the result corrected for structural and functional features. There are many more publications suggesting various empirical methods for the calculation of the heat of combustion from experimental data (short abstracts of which have been given by Handrick [18]), however, in all these cases they are limited to specific classes of molecules. In 1956, Handrick [18] published a method which is “based on adequate experimental evidence that the molar heat of combustion of any organic homologous series bearsa straight-line relation to the number of atoms of oxygen lacking in the molecule which are required to burn the compounds to carbon dioxide, water, nitrogen, HX, and sulfur dioxide.” He called this number “molecular oxygen balance”. For the calculation he used this parameter together with a number of rules for various functional groups and applying paraffin as a base. Evidently, none of the methods described so far provides a straightforward pathway to a simple algorithm for the calculation of the heat of combustion, which is generally applicable for any kind of complexities of molecules. Nevertheless, Handrick’s observation of the rigid relation between starting material and combustion products clearly indicated that a generalizable approach for the calculation of the heat of combustion is achievable.

For the calculation of the heat of formation there are many highly sophisticated quantum-theoretical methods on the market nowadays, (see, e.g., Ohlinger *et al.* [19]). However, these methods have a few disadvantages in that they are usually progressively time-consuming and thus expensive for routine evaluations and limited to relatively small molecules. Beyond this, the accuracy of their results is by no means better than the one achieved by group-additivity methods. Therefore, the latter approach, as described in 1993 by Cohen and Benson [20] for enthalpy-of-formation calculations, has still found its justification in that it is very fast and its parameters are based on experimental data.

A particularly difficult field in computer chemistry is the prediction of the biological activity of molecules, because in most cases their mode of action is unknown and even varies from molecule to molecule. Therefore, studies dealing with the calculation of bioactivity descriptors based on a series of experimental data usually do not, or only summarily, discuss the reason as to why a certain set of molecular parameters has been applied. Typical examples are the descriptors toxicity and the blood-brain barrier described in the following. 

Prediction of the toxicity of organic compounds in water has become another important area for QSAR studies. In most cases the experimental data for a series of commonly used compounds have been determined by their effects on the protozoan *Tetrahymena pyriformis*. Various methods have been applied to predict this descriptor: recently, Schultz [21] derived the toxicity of a series of substituted benzenes from the hydrophobicity, determined as logP_O/W_, plus the electrophilic reactivity, quantified by the maximum superdelocalizability S_max_; Duchowicz *et al.* [22] filtered out seven parameters from a set of 1338 topological, geometrical and electronic molecular descriptors, feeding them into an artificial neural network to evaluate the toxicity of 250 phenol derivatives; similarly, Melagraki *et al.* [23] used the hydrophobicity (logP_O/W_), the acidity constant (pKa), the HOMO and LUMO orbital energies and the hydrogen bond donor number (N_hdon_) and applying an ANN method based on the radial basis function architecture for the prediction of the toxicity of 221 phenols and compared the data to standard multiple linear regression models; Ellison [24] reduced the number of parameters to the hydrophobicity logP_O/W_ itself plus a constant to derive the toxicity of alcohols, esters, ketones and cyanides, defining for each of these groups a structural range of applicability; density functional theory as well as other semiempirical Hamiltonian methods have been used by Pasha [25] to evaluate—besides the molecular weight—the hardness, chemical potential, total energy and electrophilic index, which are then introduced into a multiple linear regression analysis and various other regression calculations for the evaluation of the toxicity of 50 phenol derivatives. A preliminary attempt, induced by Ellison’s work, to directly correlate logP_O/W_ with toxicology data of 335 compounds for which both experimental data are known and which encompass the whole range of chemical structures mentioned above yielded a correlation coefficient R^2^ of 0.7043 (the correlation diagram of which is shown further down). This encouraging result gave reason to try to apply the group-contribution method itself for the calculation of a compound’s toxicology value, based on the experimental data of the entire spectrum of chemical structures as far as their experimental data were available.

The blood-brain barrier (BBB) is a very efficient cellular system to protect the brain from unwanted content in the surrounding blood stream. In most cases, this may be desirable to prevent CNS-related side-effects of drugs. Logically, however, this barrier also tries to prevent intrusion of therapeutic chemicals for treatment of cerebral diseases. Fortunately, at least in the therapeutic sense, this barrier is not completely insurmountable, but the experimental determination of the barrier penetration of a new drug is time-consuming and expensive. Therefore, many attempts to predict the degree of BBB penetration, defined as the steady-state brain/blood distribution ratio logBB, have been published: Luco [26] used topological descriptors in partial least-squares analysis for the modeling logBB of 61 compounds; Fu *et al.* [27] based their model on the molecular volume and polar surface area of 79 compounds; the electrotopological states of the constituting atoms of 106 molecules was used by Rose *et al.* [28]. Thermodynamic calculations, such as the evaluation of the free solvation energy by Keserü and Molnar [29] as well as molecular dynamics simulations, e.g., by Carpenter *et al.* [30], have been applied to predict logBB, based on a very limited number of examples. Genetic algorithms have been used by Hou and Xu [31] on a series of 27 descriptors calculated from 96 structurally diverse compounds in order to select the statistically most significant groups of linear models with up to three or four descriptors. They concluded from the best-fitting models that logP and the partial negative solvent-accessible surface area play a crucial role in the BBB permeability. Similarly, Chen *et al.* [32] also observed the importance of the polar surface area and logP, using an artificial neural network model. On the other hand, P. Garg and J. Verma [33], also based on an ANN model, concluded that the order of importance in the evaluation of the BBB permeability is the molecular weight, followed by the polar surface area, logP, the number of H-bond acceptors and the number of H-bond donors. Quantum chemical descriptors (dipole moment, polarizability, equalized molecular electronegativity, molecular hardness, molecular softness, molecular electrophilicity, charges, charge separations, covalent H-bond acidity and basicity as well as electrostatic potential derived properties), calculated by an ab initio method, have been put together by van Damme *et al.* [34] with a series of classical descriptors encompassing logP, molecular weight, polar surface area and further structure- and shape-related properties in a model of finally eight parameters. Again, it turned out that loP and the polar surface area, besides the Mulliken charge-related descriptors, seem to be essential attributes of the model to reproduce the logBB data best, which they ascribe to the assumption that “logBB is a function of the lipophilicity and electronic properties of the molecule” [34]. Several further authors carried out logBB calculations based on the two parameters logP and polar surface area of the molecules, either on these parameters alone such as Clark [35] or together with the polarizabilty (De Sä *et al.* [36]), or including the number of acidic or basic atoms (Vilar *et al.* [37]), or only logP together with the molecular mass or the isolated atomic energy (Bujak *et al.* [38]). Interestingly however, Lanevskij *et al.* [39] observed that there is no direct correlation between logP_O/W_ and logBB at all (a fact which is confirmed in the present work), indicating “that logBB is not a measure of lipophilicity-driven BBB permeability” [39]. They found that replacement of the experimental logBB values by the ratios of total brain to unbound plasma concentrations (which meant to correct logBB by the amount of protein binding in the plasma) considerably improved correlation with logP. Sun [12] tried a direct approach to evaluate logBB by applying a number of atom type descriptors, which is very similar to the present group-additivity method, characterizing 57 compounds, representing a limited structural diversification set.

In view of the many different—successful but mostly elaborate—attempts to reliably evaluate all the molecular descriptors mentioned above it seemed unrealistic to propose a general and simple computer algorithm which would be able to calculate all the descriptors at once. However, as will be shown here, the present algorithm lifts all the limitations discussed above and is not only suitable for the calculation of thermodynamic (heat of combustion and—indirectly-formation), solubility-related (logP and logS), optical (molar refractivity), electrical (molecular polarizability) as well as biological (toxicology and potentially CNS-related) properties of a molecule at once, but also delivers reliable results and, beyond this, has the advantage of being easily extendable to compounds with structural features for which as yet no parameters are known without the need to readjust the computer algorithm.

## 2. General Procedure 

The general algorithm for the calculation of the mentioned molecular descriptors is founded on the principle of atom group contributions in analogy to the method described by Ghose and Crippen [6,7], extended in some cases by a few specific terms which will be outlined later on.

### 2.1. Definition of the Atom Groups

The present calculation procedure takes advantage of a knowledge database of presently more than 20,000 compounds, stored in geometry-optimized three-dimensional form, wherein—fulfilling the first requirement—for a certain number of molecules the experimental values for the molecular descriptors considered here are known and included in the database, each by a specific term known to the computer algorithm.

The second requirement for the calculation of the contributions of the atom groups is their definition. Since in the present approach, which should be equally applicable for the calculation of various molecular descriptors which have nothing in common but the molecular structure as a whole, no prior assumption was allowed as to the method of partitioning the molecule into its fragments. Therefore, in a potentially naive attempt, the molecular structures are broken down into their lowest-possible but still distinguishable fragments, *i.e.*, into the constituting atoms and their immediate neighbourhood as was suggested by Cohen and Benson [20,40]. Under this prerequisite, in principle, the definition of the group terms and their setup in a table could have been taken over by a computer algorithm, which would make use of the structural information of all the molecules in the database for which the requested experimental data are known, but in order to maintain a certain logic in the table order, the group terms have been generated manually and set up in a general table, which then should serve as a “mother” table for the individual parameters tables.

The above-mentioned fragmentation principle made it easy to define the atom groups in a standardized way enabling it to be set up into a programmable algorithm: each group consists of a central atom and its immediate neighbour atoms. The central atom, called “backbone atom”, is bound to at least two other atoms and is characterized by its atom name, its atom type being defined by either its orbital hybridization or bond type or its number of bonds, where required for distinction, and by its charge, if not zero. The neighbour atoms are collected in a term which lists all the neighbours following the order H > B > C > N > O > S > P > Si > F > Cl > Br > I and for each encompasses—in this order—the bond type of its bond with the backbone atom (if not single), its atom name and its number of occurrences (if >1). (For better readability of a neighbours term containing iodine its symbol is written as J.) Additionally, if the total net charge of the neighbour atoms is non-zero, the charge is appended to the neigbour term by a “(+)” or “(−)”, respectively.

Finally, for N with three single bonds (atom type “N sp3”) and O and S with two single bonds (atom types “O” and “S2”, respectively), where neighbour atoms are part of a conjugated moiety, the neighbour term is further supplemented by the terms “(pi)”, “(2pi)” or “(3pi)”, respectively. This is to take account of the increased strength of a group’s bonds due to the π-orbital conjugation of the backbone atom’s lone-pair electrons with conjugated neighbour moieties.

Hence, an atom group is uniquely defined by the term for the backbone-atom type and the term for its neighbours, which is easily interpretable as shown in the examples Table 1. For clarity the backbone atom is pronounced in the “meaning” column in boldface.

**Table 1:** Group examples and their meaning.

Atom Type	Neighbours	Meaning	Atom Type	Neighbours	Meaning
C sp3	H3C	C–**C**H_3_	N sp3	H2C	C–**N**H_2_
C sp3	H3N	N–**C**H_3_	N sp3	H2C(pi)	C–N*H_2_
C sp3	H2C2	C–**C**H_2_–C	N sp3	C2N(2pi)	C–N*(N)–C
C sp3	H2CO	C–**C**H_2_–O	N sp2	H=C	C=**N**H
C sp3	HC3	C–**C**H(C)–C	N sp2	C=N	N=**N**–C
C sp3	HC2Cl	C–**C**H(Cl)–C	N sp2	=CO	C=**N**–O
C sp3	HCO2	C–**C**H(O)–O	N(+) sp3	H3C	C–**N**H_3_^+^
C sp3	C3N	C–**C**(C)_2_–N	N(+) sp3	H2C2	C–**N**H_2_^+^–C
C sp3	C2F2	C–**C**F_2_–C	N(+) sp2	CO=O(−)	O=**N**^+^(O^−^)–C
C sp2	H2=C	C=**C**H_2_	N aromatic	:C2	C:**N**:C
C sp2	HC=C	C=**C**H–C	N(+) sp	=N2(−)	N=**N**^+^=N^(−)^
C sp2	HC=N	N=**C**H–C	O	HC	C–**O**H
C sp2	H=CN	C=**C**H–N	O	HC(pi)	C–O*H
C sp2	HN=O	O=**C**H–N	O	Si2	Si–**O**–Si
C sp2	C2=O	O=**C**(C)–C	P3	C3	C–**P**(C)–C
C sp2	C=CN	C=**C**(C)–N	P4	CO2=O	O=**P**(O_2_)–C
C sp2	=CNO	C=**C**(N)–O	P4	N2O=O	O=**P**(O)(N)–N
C sp2	N=NO	N=**C**(N)–O	S2	HC(pi)	C–S*H
C sp2	NO=O	O=**C**(N)–O	S2	CS	C–**S**–S
C aromatic	H:C2 ^a^	C:**C**H:C	S4	CO=O2	C–**S**(=O)_2_–O
C aromatic	H:C:N	C:**C**H:N	S4	O2=O	O–**S**(=O)–O
C aromatic	:CN:N	C:**C(**N):N	Si	C2Cl2	C–**Si**Cl_2_–C
C sp	H#C ^b^	C#**C**H	Si	OCl3	O–**Si**Cl_3_
C sp	C#N	N#**C**–C			
C sp	#CN	C#**C**–N			
C sp	=C2	C=**C**=C			
C sp	=C=O	C=**C**=O			

^a^: **:** represents an aromatic bond; ^b^: # represents a triple bond; *: lone-pair electrons form π-orbital conjugated bonds with neighbour atoms.

It is evident that this radical break down of molecules into the atom groups as shown does not reflect any knowledge about the molecules’ three-dimensional structure. Yet, it is well known that structural peculiarities such as buttressing effects, ring strains, gauche bond interactions or internal hydrogen bonds have a distinct influence on the values of the molecules’ heat of formation and combustion.

In the case of the calculation of logP values, Klopman *et al.* [41], using a different group-additivity method, found that for pure saturated and unsaturated hydrocarbons inclusion of a correction factor per carbon atom clearly improved conformance with experiments. They also added a correction parameter for non-branched (CH_2_)*_n_* chains on (hetero)aromatics with a polar end group X where n is greater than 1. Although the atom group fragmentation method in the present case is more detailed, the suggested correction factors have been included here as well (and in the case of the non-branched CH_2_ chains without restrictions). They indeed caused some improvement as will be outlined later.

In order to take account of these specific steric interactions and hydrophobic effects, the table of atom groups has been extended by some groups for which the terms “atom type” and “neighbours” are not rigorously applicable, but which are treated in the calculation of the group contributions in exactly the same way as ordinary atom groups. In Table 2, the definitions of these special groups and their explanation are given.

**Table 2:** Special Groups and their Meaning.

Atom Type	Neighbours	Meaning
H	H Acceptor	Intramolecular H bridge between acidic H (on O, N or S) and basic acceptor (O, N or F)
H	H	Intramolecular H–H distance <2 Angstroms
H	H	Intramolecular H–H distance 2–2.3 Angstroms
Angle60		Bond angle <60 deg
Angle90		Bond angle between 60 and 90 deg
Angle102		Bond angle between 90 and 102 deg
Alkane	No of C atoms	Correction factor per carbon atom in pure alkanes
Unsaturated HC	No of C atoms	Correction factor per carbon atom in pure aromatics, olefins and alkynes
X(CH2)*n*	No of CH2 groups	Correction factor per CH_2_ group in CH_2_ chains with end group X = CH_3_, NH_2_, OH, SH or halogen

The present detailed fragmentation of the molecules clearly bears positive and negative consequences. On the positive side lies the stronger “individualization” of the atom groups leading to better conformance with experimental data. This is particularly evident when dealing with molecules which can acquire various prototropic forms, e.g., ordinary amino acids, the equilibrium of which usually lies on the zwitterionic side. This paper will show that the differences between the calculated and experimental values of certain properties immediately answer the question concerning these equilibria. A second advantage of the present fragmentation method is the easy extendability of the number of atom groups if required for the inclusion of further molecules with known experimental descriptors data without the need to alter the computer algorithm. In fact, it is the applied parameters table itself instructing the computer program which atomic and special groups are to be taken into account for the calculations of the contributions and subsequently the descriptor data.

The negative side of this detailed molecule break-down, however, already shows up at the time of evaluating the group-contribution values: the number of molecules carrying a specific atom group can decrease to figures, which are no longer representative to confirm the final contribution value. In the extreme case of only one molecule for a given atom group, its calculated contribution value is merely the “last” summand to exactly fit the experimental descriptor value. The present work took account of this in that in all the consecutive calculations of molecular descriptors only atom groups were considered which were represented by at least three independent training molecules.

An obvious consequence of these conditions is apparent when entering a new molecule for which not all of the atom groups it contains are found—or if found are represented by less than three training molecules—in the parameters table. In that case the corresponding molecular descriptor can simply not be evaluated. This consequently requires that the first step of an automated calculation algorithm is to check if all these conditions are met.

### 2.2. Calculation of the Group Contributions

The algorithm for the evaluation of the atom group contributions for each of the title descriptors is identical. The only difference is given by the input data: the first step is the extraction from the database of a list of molecules with the known experimental value of the descriptor in question. For each molecule of this list the atom groups are then defined and counted following the rules given above.

The further proceeding is then ruled by the content of the manually set-up “mother”-parameters table of atomic and special groups: this mother table initially covers all possible combinations of “backbone” atom types and neighbourhoods. For a specific descriptor, however, always a certain—and for each descriptor different—surplus number of atom groups remains which is not represented in any molecule of the applied molecules list. These atom groups are removed before proceeding further, thus leaving an individual parameters table for a particular descriptor. This table is finally complemented with those special groups shown in Table 2 as required for this descriptor.

The resulting data set is then translated into an M × (N + 1) matrix where M is the number of molecules and (N + 1) the number of atomic and special groups plus an element for the experimental value. Each matrix element (*i,j*) then receives the number of occurrences of the *j*th atomic or special group in the *i*th molecule. After normalization of this matrix into an *Ax = B* matrix equation and its equalization by means of the Gauss-Seidel calculus, the resulting group-contribution values are entered into the corresponding parameters table. Additionally, to each atomic and special group the number of its occurrences (its frequency) and the number of molecules containing it are added. Next, the parameters table receives the information about the goodness of fit (R^2^), the average and standard deviation and the total number of molecules on which the calculation is based.

### 2.3. Calculation of the Descriptors

Once the group contributions are set up in the corresponding parameters tables, the computation of any of the descriptors’ values Y is a mere summing up of the contributions of the atom groups found in a molecule following the general Equation 1

(1) $$Y=\sum_{i}a_{i}A_{i}+\sum_{j}b_{j}B_{j}+C$$

wherein *a_i_* and *b_j_* are the contribution values, listed in the respective parameters table, *A_i_* is the number of occurrences of the *i*th atom group, *B_j_* is the number of occurrences of the special groups and C is a constant. However, as was mentioned earlier, this calculation is limited to molecules for which each atom group it contains (not special group!) the corresponding one is present in the corresponding parameters table and its value is confirmed by at least three training molecules. Hence, a computer algorithm has to start with the definition and counting of all the molecule’s atom groups (applying the same procedure as in the second step for the calculation of the group contributions), then check for any atom group that is missing (or is not confirmed) in the parameters table and then either continue using the above formula if all groups are found or reject further calculation. Calculation of all the title descriptors at once on a notebook is done in a split second, once the compound’s three-mensional structure is generated and added to the molecules database (see Appendix).

### 2.4. Cross-Validation Calculations

In order to check the plausibility of the results of the group-additivity method for the prediction of the molecular descriptors, in each case a k-fold cross-validation calculation is carried out, whereby, after a few tentative calculations with various *k* values, k is in all cases chosen to be 10. Accordingly, the complete list of compounds holding a particular experimental descriptor value is first copied into a training set, wherefrom a test set is extracted by the transfer of every k-th, *i.e.*, every 10th compound, thus producing a training set containing 90% of the molecules of the original list and the remaining 10% as test set. In a next step, the training set is used to calculate the atom groups parameters set and then, by means of these parameters, the prediction value is evaluated for each molecule of the test set and added to its properties list. This procedure is repeated k (=10) times, each time shifting the extraction process for the test-set from the re-setup training set by the repetition run-time number, this way making sure that each compound is used exactly once as a test molecule and that no inadvertent clusters of certain structures are extracted from the training sets. Finally, the collected prediction data of all the test molecules are used to evaluate the cross-validated regression coefficient Q^2^ and the corresponding average and standard deviation. These data are finally entered at the end of each parameters table. The number of compounds on which these cross-validation calculations are founded is in general smaller than the number of compounds used for the evaluation of the correlation coefficient R^2^, because due to the exclusion of the test compounds in the atom group parameters calculations certain atom groups may no be longer represented by enough molecules and, thus, test compounds having these atom groups are excluded from the prediction calculation.

## 3. Results 

General remark: In all the correlation diagrams of the following chapters cross-validated data, if included, are indicated as red circles.

### 3.1. Heat of Combustion

In order to achieve reproducibility over all compound classes and literature references, the experimental data have only been accepted for the calculations if the starting material as well as its combustion products are described as relaxed in their thermodynamic standard states, *i.e.*, in their stable form at 25 °C and standard atmospheric pressure. The computation of the atom group contributions listed in Table 3 are based on the experimental data of organic molecules published in several papers, essentially E. S. Domalski’s collection of compounds [42] containing the elements C, H, N, O, P and S, supplemented with data for further nitrogen compounds by Young *et al.* [43], for a series of amino acids by Ovchinnikov [44], for fluoro and chloro compounds by Cox *et al.* [45], Smith *et al.* [46] and Shaub [47], for bromo compounds by Bjellerup [48], for peroxy acids and esters by Swain Jr. *et al.* [49], for silicon-containing compounds by Tannenbaum *et al.* [50] and Good *et al.* [51], and finally by the National Institute of Standards and Technology [52] and their respective literature citations. A number of experimental heat-of-combustion data was indirectly evaluated from experimental heat-of-formation values of compounds, for which only these were cited [53], using standard heat-of-formation data for the oxidation products. Where required the data are multiplied from kcal/mol to kJ/mol by the factor 4.1868. The calculations excluded compounds containing elements that differ from H, B, C, N, O, P, S, Si or the halogens. Explanations of the groups definitions in Table 3 are given in Table 1. 

**Table 3:** Atom groups and their Contributions (in kJ/mol) for Heat-of-Combustion Calculations.

Nr	Atom Type	Neighbours	Contribution	Occurrences	Molecules
1	B	C3	−4309.05	3	3
2	C sp3	H3B	439.88	3	1
3	C sp3	H3C	−773.83	2294	1153
4	C sp3	H3N	−1199.10	110	65
5	C sp3	H3N(+)	−817.94	3	3
6	C sp3	H3O	−1112.98	178	115
7	C sp3	H3S	−1396.74	23	19
8	C sp3	H3P	−1052.64	3	1
9	C sp3	H3Si	−1008.77	51	16
10	C sp3	H2BC	553.89	6	2
11	C sp3	H2C2	−652.47	4413	912
12	C sp3	H2CN	−1074.20	183	117
13	C sp3	H2CN(+)	−705.22	44	26
14	C sp3	H2CO	−980.99	610	374
15	C sp3	H2CS	−1274.78	106	72
16	C sp3	H2CP	−852.22	5	2
17	C sp3	H2CF	−623.15	8	7
18	C sp3	H2CCl	−617.40	51	42
19	C sp3	H2CBr	−623.39	22	19
20	C sp3	H2CJ	−685.52	10	8
21	C sp3	H2CSi	−932.85	22	13
22	C sp3	H2N2	−1480.52	9	2
23	C sp3	H2N2(+)	−807.51	1	1
24	C sp3	H2NO	−1375.72	1	1
25	C sp3	H2O2	−1279.46	11	9
26	C sp3	H2OCl	−951.95	3	2
27	C sp3	H2S2	−1932.88	5	3
28	C sp3	HC3	−529.62	363	254
29	C sp3	HC2N	−957.93	47	37
30	C sp3	HC2N(+)	−575.78	33	32
31	C sp3	HC2O	−850.09	277	138
32	C sp3	HC2S	−1152.31	20	16
33	C sp3	HC2F	−504.42	3	3
34	C sp3	HC2Cl	−497.94	10	10
35	C sp3	HC2Br	−500.70	9	7
36	C sp3	HC2J	−558.92	1	1
37	C sp3	HCN2	−1363.17	1	1
38	C sp3	HCN2(+)	−672.56	2	2
39	C sp3	HCO2	−1153.93	40	30
40	C sp3	HCF2	−433.94	8	7
41	C sp3	HCFCl	−472.96	4	4
42	C sp3	HCCl2	−494.62	9	8
43	C sp3	HCClBr	−518.18	1	1
44	C sp3	HCBr2	−476.37	1	1
45	C sp3	HN3(+)	−870.19	1	1
46	C sp3	HO3	−1433.08	4	4
47	C sp3	HOF2	−729.48	2	2
48	C sp3	C4	−403.80	117	91
49	C sp3	C3N	−813.97	13	10
50	C sp3	C3N(+)	−426.89	13	12
51	C sp3	C3O	−730.08	36	30
52	C sp3	C3S	−1023.12	15	12
53	C sp3	C3F	−179.93	2	2
54	C sp3	C3Cl	−361.21	2	2
55	C sp3	C3Br	−362.53	2	2
56	C sp3	C3J	−432.30	1	1
57	C sp3	C2N2(+)	−626.56	5	4
58	C sp3	C2O2	−1004.06	25	24
59	C sp3	C2F2	−320.26	60	15
60	C sp3	C2FCl	−318.84	2	1
61	C sp3	C2Cl2	−356.73	4	4
62	C sp3	CN3(+)	−746.41	6	4
63	C sp3	CO3	−1284.92	7	6
64	C sp3	COF2	−649.83	1	1
65	C sp3	CF3	−243.86	45	36
66	C sp3	CF2Cl	−302.73	8	6
67	C sp3	CF2Br	−320.46	5	4
68	C sp3	CFCl2	−323.43	5	5
69	C sp3	CFClBr	−275.67	1	1
70	C sp3	CCl3	−366.35	14	13
71	C sp3	CBr3	−339.39	1	1
72	C sp3	N4(+)	−896.07	1	1
73	C sp3	O4	−1580.14	2	2
74	C sp3	OF3	−531.65	2	2
75	C sp2	H2=C	−702.52	164	148
76	C sp2	H2=N	−928.80	1	1
77	C sp2	HC=C	−566.63	462	270
78	C sp2	HC=N	−762.24	14	13
79	C sp2	HC=O	−396.09	60	57
80	C sp2	H=CN	−958.41	32	24
81	C sp2	H=CN(+)	−595.08	3	3
82	C sp2	H=CO	−747.98	20	18
83	C sp2	H=CS	−1161.32	11	9
84	C sp2	H=CF	−546.98	2	2
85	C sp2	H=CCl	−555.33	6	5
86	C sp2	H=CBr	−573.39	2	2
87	C sp2	H=CSi	−833.05	3	3
88	C sp2	HN=N	−1134.46	18	15
89	C sp2	HN=O	−762.26	10	10
90	C sp2	H=NO	−916.53	2	2
91	C sp2	HO=O	−545.94	19	19
92	C sp2	H=NS	−1372.72	2	2
93	C sp2	C2=C	−433.99	125	97
94	C sp2	C2=N	−630.40	6	5
95	C sp2	C2=O	−248.77	94	78
96	C sp2	C=CN	−825.51	33	26
97	C sp2	C=CO	−602.48	16	16
98	C sp2	C=CS	−1031.71	3	3
99	C sp2	C=CF	−439.03	5	3
100	C sp2	C=CCl	−397.75	8	5
101	C sp2	CN=N	−991.99	17	16
102	C sp2	CN=O	−621.43	128	95
103	C sp2	CN=S	−1460.28	3	2
104	C sp2	CO=O	−389.60	500	370
105	C sp2	CO=O(−)	−534.91	49	45
106	C sp2	C=OS	−844.48	4	4
107	C sp2	C=OF	−174.28	1	1
108	C sp2	C=OCl	−205.80	8	7
109	C sp2	C=OBr	−204.22	2	2
110	C sp2	C=OJ	−281.70	2	2
111	C sp2	=CN2	−1249.51	8	8
112	C sp2	=CNO(+)	−678.42	2	2
113	C sp2	=COF	−430.57	2	2
114	C sp2	=CF2	−415.97	9	8
115	C sp2	=CFCl	−359.75	1	1
116	C sp2	=CCl2	−407.94	4	3
117	C sp2	=CJ2	−544.25	2	1
118	C sp2	N2=N	−1416.91	40	35
119	C sp2	N2=O	−1022.83	56	47
120	C sp2	N2=S	−1839.83	5	5
121	C sp2	N=NO	−1202.52	1	1
122	C sp2	NO=O	−772.08	7	7
123	C sp2	N=OS	−1488.48	1	1
124	C sp2	NS=S	−2092.99	3	2
125	C sp2	O2=O	−546.67	6	6
126	C sp2	O=OCl	−338.25	2	2
127	C aromatic	H:C2	−543.64	3345	599
128	C aromatic	H:C:N	−776.86	47	30
129	C aromatic	H:C:N(+)	−497.10	3	2
130	C aromatic	H:N2	−1022.16	2	2
131	C aromatic	:C3	−407.72	235	72
132	C aromatic	C:C2	−413.58	769	420
133	C aromatic	C:C:N	−630.62	38	17
134	C aromatic	C:C:N(+)	−361.54	1	1
135	C aromatic	:C2N	−844.05	161	113
136	C aromatic	:C2N(+)	−494.97	144	76
137	C aromatic	:C2:N	−644.82	19	13
138	C aromatic	:C2O	−619.12	122	93
139	C aromatic	:C2S	−1044.73	21	13
140	C aromatic	:C2F	−401.83	40	14
141	C aromatic	:C2Cl	−393.22	33	20
142	C aromatic	:C2Br	−399.84	4	4
143	C aromatic	:C2J	−468.14	17	14
144	C aromatic	:C2Si	−686.64	2	1
145	C aromatic	:CN:N	−1064.47	3	2
146	C aromatic	:C:NO	−835.98	5	3
147	C aromatic	N:N2	−1260.90	6	3
148	C aromatic	:N3	−583.18	3	3
149	C aromatic	:N2Cl	−828.48	1	1
150	C sp	H#C	−653.92	34	28
151	C sp	C#C	−506.41	55	34
152	C sp	C#N	−508.61	53	40
153	C sp	#CN	−1006.69	2	2
154	C sp	#CCl	−512.21	1	1
155	C sp	N#N	−912.20	2	2
156	C sp	#NO	−801.89	1	1
157	C sp	=C2	−554.47	6	6
158	C sp	=C=N	−741.19	2	2
159	C sp	=C=O	−323.55	1	1
160	C sp	=N=O	−433.06	5	4
161	C sp	=N=S	−1250.00	1	1
162	N sp3	H2C	144.43	49	44
163	N sp3	H2C(pi)	191.61	124	102
164	N sp3	H2N	−321.73	12	11
165	N sp3	H2N(pi)	−263.42	1	1
166	N sp3	H2S	−356.54	1	1
167	N sp3	HC2	657.84	30	28
168	N sp3	HC2(pi)	707.92	58	47
169	N sp3	HC2(2pi)	714.30	117	84
170	N sp3	HCN	209.21	3	2
171	N sp3	HCN(pi)	254.66	15	9
172	N sp3	HCN(2pi)	274.14	27	25
173	N sp3	HCN(+)(2pi)	382.93	3	3
174	N sp3	C3	1170.07	22	18
175	N sp3	C3(pi)	1214.78	27	22
176	N sp3	C3(2pi)	1214.87	24	13
177	N sp3	C3(3pi)	1229.16	2	2
178	N sp3	C2N	739.41	1	1
179	N sp3	C2N(pi)	781.06	1	1
180	N sp3	C2N(+)(pi)	919.58	6	4
181	N sp3	C2N(2pi)	771.90	16	13
182	N sp3	C2N(+)(2pi)	879.10	4	3
183	N sp3	C2N(3pi)	787.25	5	5
184	N sp3	C2Si	750.91	1	1
185	N sp3	C2Cl(2pi)	747.48	1	1
186	N sp3	C2Br(2pi)	769.45	1	1
187	N sp3	CN2(2pi)	384.22	6	4
188	N sp3	CN2(3pi)	424.65	1	1
189	N sp2	H=C	−7.70	8	8
190	N sp2	C=C	550.75	37	32
191	N sp2	C=N	310.59	28	14
192	N sp2	C=N(+)	237.35	11	11
193	N sp2	=CN	119.10	51	42
194	N sp2	=CN(+)	302.14	1	1
195	N sp2	C=O	396.97	5	5
196	N sp2	=CO	192.01	12	9
197	N sp2	N=N	−89.71	64	31
198	N sp2	N=O	−43.12	2	2
199	N sp2	O=O	356.35	2	2
200	N aromatic	H2:C(+)	−122.03	7	3
201	N aromatic	HC:C(+)	814.57	1	1
202	N aromatic	C2:C(+)	1314.74	1	1
203	N aromatic	:C2	412.85	64	47
204	N aromatic	:C:N	134.45	2	1
205	N(+) sp3	H3C	259.93	36	35
206	N(+) sp3	H2C2	381.05	4	4
207	N(+) sp3	HC3	531.03	6	3
208	N(+) sp2	CO=O(−)	116.31	218	116
209	N(+) sp2	C=NO(−)	139.32	1	1
210	N(+) sp2	NO=O(−)	−143.13	14	11
211	N(+) sp2	O2=O(−)	436.53	11	6
212	N(+) aromatic	H:C2	297.18	2	2
213	N(+) sp	C#C(−)	−520.51	2	2
214	N(+) sp	=N2(−)	−156.85	10	10
215	O	HC	389.05	437	219
216	O	HC(pi)	283.41	309	243
217	O	HN(pi)	−67.43	9	6
218	O	HO	−30.25	8	7
219	O	HS	−2.73	6	5
220	O	HSi	209.41	1	1
221	O	C2	778.41	245	141
222	O	C2(pi)	676.08	299	224
223	O	C2(2pi)	540.34	43	41
224	O	CN(pi)	0.00	2	2
225	O	CN(+)(pi)	0.00	11	6
226	O	CN(2pi)	242.71	3	3
227	O	CO	377.06	11	8
228	O	CO(pi)	233.20	11	9
229	O	CS	309.60	17	9
230	O	CP	386.05	13	5
231	O	CP(pi)	225.09	3	1
232	O	CSi	392.68	23	7
233	O	Si2	34.75	8	3
234	P3	C3	0.00	1	1
235	P4	C2O=O	−128.03	1	1
236	P4	C3=O	−172.02	1	1
237	P4	O3=O	8.54	5	5
238	S2	HC	−110.34	39	35
239	S2	HC(pi)	−117.44	3	3
240	S2	C2	629.97	40	36
241	S2	C2(pi)	613.04	7	7
242	S2	C2(2pi)	652.85	12	11
243	S2	CS	25.47	16	8
244	S2	CS(pi)	13.65	6	3
245	S4	C2=O	764.28	4	4
246	S4	C2=O2	1000.31	14	14
247	S4	CO=O2(−)	113.62	1	1
248	S4	NO=O2	2.73	1	1
249	S4	O2=O	−121.52	4	4
250	S4	O2=O2	89.44	6	6
251	S4	O=O2F	−120.52	1	1
252	S4	O=O2Cl	−114.10	1	1
253	Si	H3C	−1004.63	4	4
254	Si	H2C2	−581.65	2	2
255	Si	HC3	−193.18	2	2
256	Si	HC2Cl	−561.69	1	1
257	Si	HCCl2	−414.48	1	1
258	Si	HO3	−463.15	1	1
259	Si	C4	130.90	3	3
260	Si	C3N	0.00	1	1
261	Si	C3O	−97.16	3	2
262	Si	C3Cl	70.09	1	1
263	Si	C3Br	57.55	1	1
264	Si	C2O2	38.94	8	3
265	Si	C2Cl2	−0.66	4	4
266	Si	CO3	8.62	6	5
267	Si	CCl3	−133.90	1	1
268	H	H Acceptor	1.25	100	80
269	H	.H	−1.22	1623	467
270	H	..H	−1.09	2258	595
271	Angle60		−38.45	120	38
272	Angle90		−25.28	186	87
273	Angle102		−5.65	469	184
A	Based on				2151
B	Goodness of fit	R^2^	1.00		2031
C	Deviation	Average	16.00		2031
D	Deviation	Standard	22.93		2031
E	K-fold cv	K	10.00		1965
F	Goodness of fit	Q^2^	0.9999		1965
G	Deviation	Average (cv)	17.50		1965
H	Deviation	Standard (cv)	25.20		1965

In view of the hitherto various approaches mentioned above to calculate the heat of combustion, which are mostly restricted to a limited class of compounds, it seems at first glance odd to assume that the present simple group additivity method should be able to cover the whole spectrum of classes of chemical compounds. However, on second thought this approach resembles the bond-energy addition method as suggested by Pauling [1], Klages [2] and Wheland [3], except that in this case not the energy of specific bonds are summed up but the energy of bond clusters around “backbone” atoms. In particular, the contributions of the intramolecular effects are worth mentioning, showing that while intramolecular interactions (lines 268–270) seem negligible, the ring strain effects (lines 271–273) are quite significant and follow the expected order and sign.

In Table 3, row A indicates the total number of molecules on which the calculation of the atom group parameters is based. Rows B to D, showing the correlation coefficient R^2^, average and standard deviation of the complete training set, and rows F to H, presenting the analogous values Q^2^ and deviations resulting from the k-fold cross-validation calculation with k = 10 (row E) prove the surprisingly excellent correlation of the calculated with the experimental data in view of the large range of heat-of-combustion values of between −42,860 (glyceryl tribrassidate, *calc*. −42,915) and −217.71 (oxalic acid dihydrate, *calc.* −235.5) kJ/mol with a goodness of fit R^2^ of >0.9999 and a standard deviation of <23 kJ/mol. The cross-validated correlation coefficient Q^2^ of also 0.9999 and the only slightly larger deviation values prove the excellent quality of the group-additivity method for the prediction of heat-of-combustion data. As was mentioned earlier, in all correlation and deviation calculations only atom groups are considered which are represented by at least three molecules (last column); as a consequence, the number of molecules for the evaluation of these data is smaller than the basis set (row A) and atom groups that do not fulfil this requirement should only be viewed as indicative.

The deviations are also in good agreement with the variations of experimental data from various sources for several compounds, as exemplified by the compounds listed in Table 4. (A more detailed discussion of the reliability of published data is given in the next chapter.) For the calculations the amino acids are assumed to generally adopt the zwitterionic form (except those where the amino group is bound to a conjugated system as, e.g., in *N*-phenylglycine or *N*-formylleucine). However, test calculations applying their neutral forms show only minor differences in the data in comparison with those of the zwitterions as would be expected for this prototropic equilibrium.

**Table 4:** Heat-of-Combustion: Experiment *vs.* Calculation (in kJ/mol).

Compound	Experimental		Calculated
	Domalski [42]	Various	
Valine	−2921.5	−2910.7 [44]	−2932.9
Threonine	−2102.6	−2084.6 [44]	−2090.5
l-Proline		−2746.2 [44]	−2749.6
dl-Proline	−2729.8	−2729.6 [44]	−2749.6
Isoleucine	−3586.0	−3578.3 [44]	−3587.8
l-Serine	−1455.8	−1448.2 [44]	−1441.4
dl-Serine		−1441.9 [44]	−1441.4
*N*-Carboxymethylglycine	−1657.1	−1641.8 [44]	−1670.5
*N*-Formylleucine	−3685.6	−3814.6 [44]	−3852.8
Trimyristin	−27,842	−27,643.7 [54]	−27,771.8

**Figure 1:**
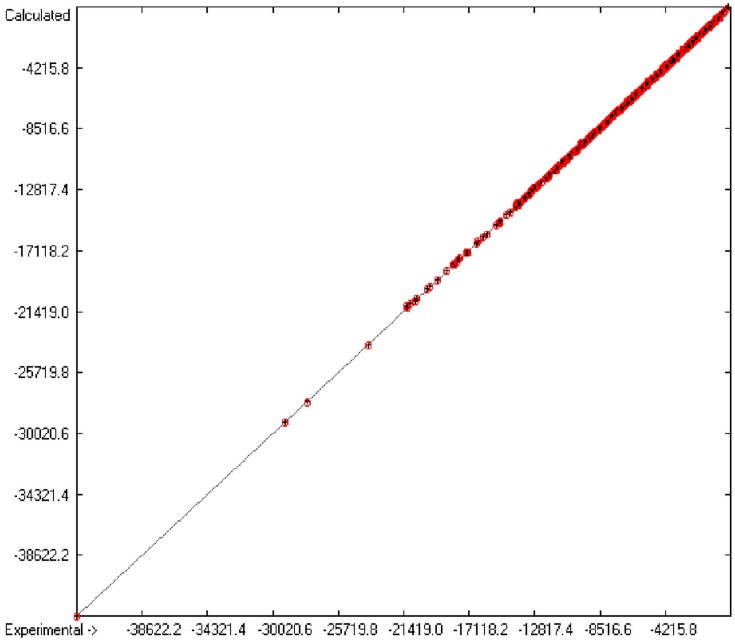
Correlation diagram of heat-of-combustion data (10-fold cross-validated: N = 2031, Q^2^ = 0.9999, slope = 1.0).

Figure 1 graphically represents perfect compliance of the calculated with the experimental data for the heat of combustion. The complete set of results is available in a separate document of the Supplementary Material under the name of “Experimental vs Calculated Heat-of-Combustion Data Table.doc”, the associated list of compounds as SD file named “Compounds List for Heat-of-Combustion Calculations.sdf”.

**Figure 2:**
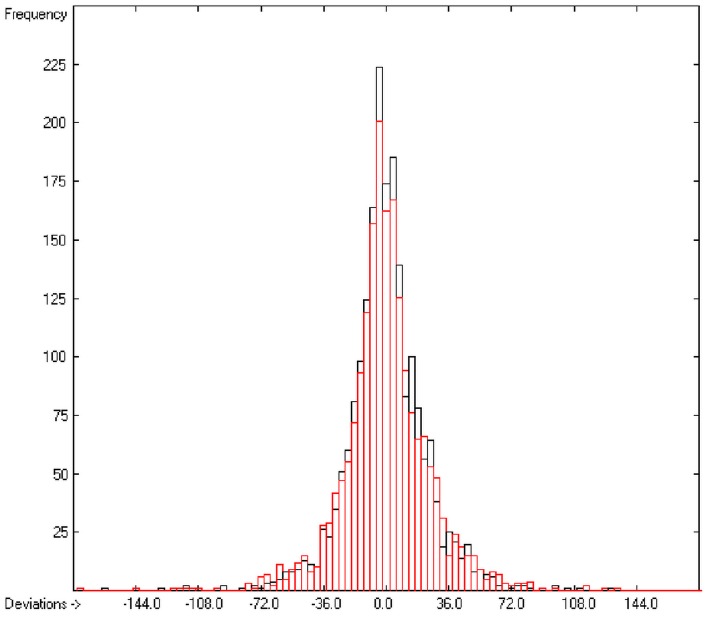
Histogram of heat-of-combustion data (S = 25.2).

In the histogram (Figure 2) the distribution of the deviations of the complete training-set and the cross-validation data show a nearly perfect Gaussian bell curve, where the cross-validation deviations (in red) are typically less populated in the center area and more in the periphery of the histogram.

### 3.2. Heat of Formation

The excellent reliability of the predicted heat of combustion data also enabled the indirect calculation of the heat of formation of the molecules making use of the heats of formation of their oxidation products. Consequently, the same limitations concerning the elements as well as the computation constraints were valid. For these evaluations the heat of formation values of CO_2_, H_2_O, H_3_BO_3_, H_2_SO_4_(+115 H_2_O), H_3_PO_4_(c), SiO_2_ and aqueous hydrogen halides, given by Skinner [55] and Domalski [20] were applied.

For comparison the predicted heat of formation values were checked against experimental values the main source of which was again Domalski’s collection of compounds [42], supplemented by data from the table volume “Standard Thermodynamic Properties of Chemical Substances” [53]. Further experimental data for hydrocarbons were provided by Domalski and Hearing [56], National Institute of Standards and Technology [52] and for amino acids by V. V. Ovchinnikov [44].

**Figure 3:**
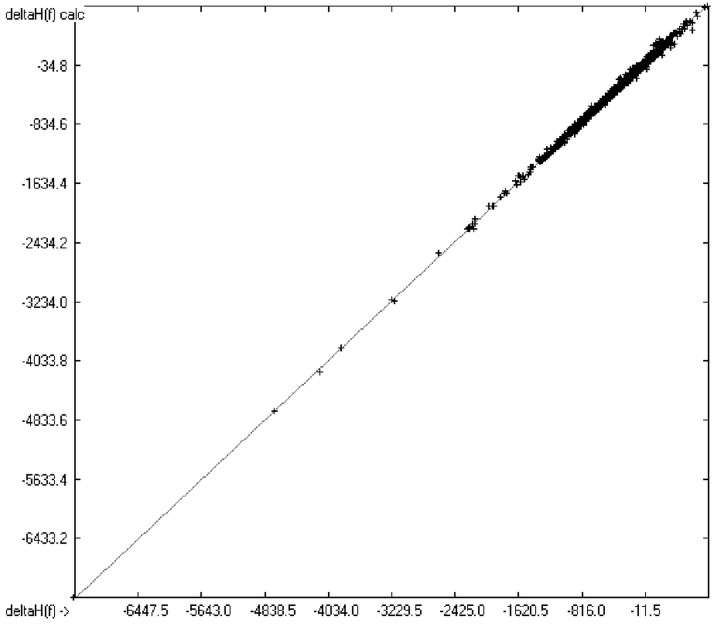
Correlation diagram of heat-of-formation data (N = 2031, R^2^ = 0.9974, slope = 1.0).

The experimental enthalpy values extended from −7251 (Perfluorohexadecane, *calc.* −7232.48) to +792 (1,1′-dimethyl-5,5′-azotetrazole, *calc.* +764.35) kJ/mol. No outlier had to be removed from the enthalpy calculations. With regard to the high correlation coefficient R^2^ and the regression line having a slope of 1 (shown in Figure 3) the conclusion seems justified that any further prediction in- and outside the given range is reliable.

Despite the surprisingly low average and standard deviations in Table 3, which translate into analogous deviations for the heat of formation due to the indirect evaluation from the heat of combustion (neglecting their increase caused by the error propagation) one should not forget that from the perspective of a kineticist who is interested in reactivities and equilibria, a “sufficiently accurate” standard deviation should not exceed 4 kJ/mol, still equivalent to a change of an equilibrium constant at room temperature by a factor of >5 or the difference between about 90% and 64% yield in a chemical reaction, independent of the enthalpy magnitude itself [20].

In order to put the the deviations also into perspective with the uncertainty of the published input data, Table 5 compares the experimental data provided by various sources of a number of compounds with the result of the present calculations.

**Table 5:** Heat of Formation: Experiment *vs.* Calculation (in kJ/mol).

Compound	Experimental		Calculated
	Domalski [42]	Various	
Ethyleneglycol	−455.1	−460.0 [53]	−461.81
Benzaldehyde	−84.2	−87.0 [53]	−86.37
Brassidic acid	−896.0	−960.7 [53]	−913.74
Triphenylene	141.2	151.8 [56]	173.36
Fluoranthene	191.6	230.3 [56]	176.03
Pyrene	114.9	125.5 [56]	152.23
Leucine	−636.3	−648.0 [44]	−639.07
*N*-Carboxymethylglycine	−919.0	−932.6 [44]	−905.86
l-Serine	−726.8	−732.7 [44]	−741.09
Isoleucine	−635.6	−640.6 [44]	−634.07

Table 4 and Table 5 also shed light onto the reliability of the published experimental thermodynamic data. Most authors discuss the probable error margins only summarily if at all. Domalski [42] defers in more detail to the uncertainties and derives their magnitude from the number of significant figures in the reported heat-of-combustion and formation data. Accordingly, a value cited to 0.01 is associated with an error of 0.05 to 0.5, a value cited to 0.1 with an error of 0.5 to 2 and a value cited to 1 with an error of 2 to 20 kcal/mol. Another important point is the state of the compound at room temperature for which the value is given. In some cases the authors provide data for two diffferent standard states; in this case the present paper applied the values for the normal state. A detailed discussion about the general accuracy of the experimental enthalpy data is given by Cohen and Benson [20].

### 3.3. Applicability and Limitations of the Group-Additivity Method for Thermodynamics Calculations

For the chemical practician the question certainly arises as to whether the present group-additivity method now is accurate enough to be applied on the thermodynamics of, e.g., chemical reactions and/or equilibria. A particularly interesting area is the issue of tautomerism, not only because it has been the subject for decennia of debates which are still ongoing but also because it can be used as a sensitive test for the applicability of the computation method. The present paper takes advantage of the ample literature concerning azo-hydrazone as well as keto-enol tautomerism to assess the quality of the present method. Table 6 presents a list of azo dyes which are known to exhibit an equilibrium between the azo and the hydrazone form. The lower enthalpy values, indicated in boldface, should correspond to the form which dominates the azo-hydrazone equilibrium. This is indeed the case: it is well known that arylazo-substituted anilines only undergo tautomerization in acidic solution, whereas arylazonaphthols generally prefer the hydrazone form, which—by the way—exhibits a large shift of the electronic absorption spectra. 2- and 4-Phenylazophenol, on the other hand, only show a weak tendency to tautomerize to the hydrazone form.

**Table 6:** Thermodynamic Data (kJ/mol) of Azo Dyes.

Compound	Hydrazone Form ∆H_f_ Calc	Azo Form ∆H_f_ Calc	^a^	Ref.
4-Phenylazophenol	154.32	**141.92**	**+**	[57]
2-Phenylazophenol	150.32	**141.92**	**+**	[57]
4-Aminoazobenzene	400.41	**315.61**	**+**	[58]
2-Aminoazobenzene	397.81	**318.90**	**+**	
1-Phenylazo-2-naphthol	**160.21**	183.51	**+**	[59,60]
4-Phenylazo-1-naphthol	**164.21**	183.51	**+**	[61]
1-Phenylazo-2-naphthylamine	410.30	**357.30**	**+**	[59,60]
4-Phenylazo-1-naphthylamine	410.30	**359.80**	**+**	[62]

^a^ Conformance with experimental data.

The limitations of the group-additivity principle are evident in Table 7. While the calculations for 1-(*N*-phenylformimidoyl)-2-naphthol are in line with experiment that it essentially exists in the enol form [41] and for acetone the calculated values for the keto and enol forms are at best inconclusive, the data for cyclohexanone and cyclopentanone are in clear contrast with the true dominant stable tautomers proven experimentally by Hine and Arata [63,64].

**Figure 4:**
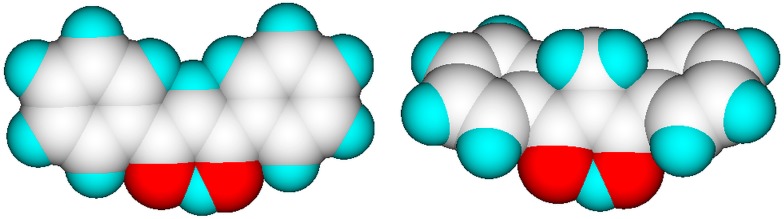
Energy-minimized enol forms of dibenzoylmethane (**left**) and of 1,1-bis(benzoyl)ethane showing the steric effect of the additional methyl group on the structure of the latter (graphics by ChemBrain IXL).

Experimental findings of the series of β-diketones (as neat liquids) are in conformance with the calculations, with the exception of 1,1-bis(benzoyl)ethane which shows the influence of steric hindrance: Allen and Dwek [65] explained the lack of enolization of this compound with the steric and/or inductive effect of the additional methyl group on the central carbon atom, clearly favouring the +I effect, which seems justified: Figure 4 shows that the additional methyl group on the central carbon atom essentially only twists the phenyl groups out of plane, but has no steric influence on the stability of the H bridge.

**Table 7:** Thermodynamic Data (kJ/mol) of Tautomeric Ketones and β-Diketones.

Compound	Keto Form ∆H_f_ Calc.	Enol Form ∆H_f_ Calc.	Experiment ∆H_f_ Exp	^a^	Ref.
1-(N-Phenylformimidoyl)-2-naphthol	70.23	**46.53**		**+**	[66]
Acetone	**−243.18**	−234.38	−248.1	**+**	[63]
Cyclohexanone	−281.24	**−296.04**	−276.1	**−**	[63]
Cyclopentanone	−233.35	**−251.75**	−240.2	**−**	[64]
Phenol	−57.50	**−166.90**	−165.2	**+**	[67]
2-Pyridone	−120.62	**−136.32**	−166.3	**−**	[68,69,70]
4-Pyridone	−98.82	**−120.52**	−148.9	**+**	[68,69,70]
Carbostyril	−74.43	**−90.53**	−144.9	**−**	[71,72,73]
Acetylacetone	−415.25	**−429.25**	−427.6	**+**	[65]
Bis(trifluoroacetyl)methane	−1659.98	**−1676.28**		**+**	[65]
Dibenzoylmethane	−203.72	**−221.02**		**+**	[65]
1,1-Bis(benzoyl)ethane	−231.31	**−258.91**		**−**	[65]

^a^ Conformance with experimental data.

The tautomeric equilibria of the pyridones have been studied extensively by many physical methods in the solid state and in solutions of various polarities (see citations in references [68,69,70]) and they indicate that in the condensed phase the equilibrium of 2-pyridone lies on the keto (lactam) side (by an indirectly measured enthalpy difference of 0.4 ± 0.6 kcal/mol [69]) and that 4-pyridone’s equilibrium is shifted to the enol (4-hydroxypyridine) side with an indirectly estimated enthalpy gap of 2.4 ± 0.6 kcal/mol [69]. Theoretical studies [68,69,70,71,72,73] also predicted a preference in the gas phase for the lactam form in the case of 2-pyridone (by *ca*. 1.7 kJ/mol), while the enol form for 4-pyridone was calculated to be more stable (by *ca*. 10 kJ/mol). The present calculations evidently only agree with the findings for 4-pyridone. On the other hand, the predicted direction of the equilibrium between the carbon-analogue phenol and its tautomers cyclohexa-2,4-diene-1-one and cyclohexa-2,5-diene-1-one is in line with experimental findings [67].

Then there is carbostyril: for more than a century this compound’s tautomerism has been under investigation [71,72,73]. The first assumption by A. Claus [71] in 1896 that the keto (lactam) form was dominant in solution rested on the analysis of its chemical selectivity towards bromination, an approach which nowadays, in view of today’s theoretical and practical knowledge about the reactivity/selectivity processes and kinetics of proton shifts, seems founded on pure speculation but was nonetheless correct as modern theoretical studies [73] confirmed. These studies, however, calculated an enthalpy difference between the lactam and lactim form of only about 1 kcal/mol. The calculated data of both forms listed in Table 7 deviate too far from the experimental ones to provide support for one or the other.

The deficiencies exhibited in Table 7 point to two principal weaknesses of the group-additiviy method: the first one is connected with the origin of the values of the group contributions and the second one is assignable to the intended isolation of the atom groups. The failure to correctly predict the keto-enol ratio in the case of acetone, cyclohexanone and cyclopentanone seems to be attributable to the fact that 12 out of the 15 compounds defining the enol moiety in the evaluation of the group contributions are aromatic systems, namely substituted furans, isoxazoles and tropolone, which could imprint the stabilizing effect of their extended conjugation onto the values of the relevant contributions. This deficiency could possibly be overcome provided that there are reliable experimental data available of isolated enols (e.g., enol ethers) which could be included in the contribution evaluations.

The second weakness of the group additivity method shows its effect in the wrong preference of the enol form for 1,1-bis(benzoyl)ethane. This deficiency is principally insurmountable because steric and electronic effects and other unusual conformational information cannot be considered by *per se* isolated atom groups. Even in the particular case of β-diketones where the hydrogen bridge normally contributes to the stabilization of the enol form, the lack of this effect in 1,1-bis(benzoyl)ethane is too little as to change the picture.

### 3.4. LogP_Octanol/Water_

The partition coefficient P between octanol and water, or more precisely: its logarithm logP, is a standard model for the expression of the lipophilicity of biological drugs in medicinal and agro chemistry and, therefore, reliable methods for its evaluation from the drugs’ structure, in particular prior to their synthesis, are very desirable. Various calculation methods have successfully been applied, of which those developed by Ghose and Crippen [6,7], Klopman *et al.* [41], Visvanadhan *et al.* [54], Leo [74], Wang *et al.* [75], Hou and Xu [76] and others may be especially mentioned, because they are also based on the atomic-group additivity method and therefore may serve as benchmarks for the present method. Most experimental log P data for this paper have been extracted from Klopman’s [41], some from Lipinski’s [77] and from Sangster’s [78] collection. Net charged compounds (not zwitterions) and strong acids are principally excluded from the present logP evaluations. Table 8 lists the atom groups and their contribution resulting from the linearization procedure using the experimental data of more than 2700 compounds of a large varietya list of which is available in the supplementary material under the name of “Compounds List for LogP Calculations.sdf”. At the same location the complete set of results is accessible under the mane of “Experimental vs Calculated LogP Data Table.doc”.

The only difference to the enthalpy Table A1 lies in the special groups 273–276 in Table 8 which replace the special groups required to factor in intramolecular and ring-strain effects on the heats of combustion and formation. These new special groups were suggested by Klopman *et al.* [41]. Groups 274 and 275 take account of the particularities of saturated and unsaturated hydrocarbons and are therefore only included in the calculations if no heteroatoms are present in the compound. In that case the contribution is multiplied by the number of carbon atoms in the molecule. The meaning of group 276 has been extended over that of Klopman’s intention in that it is considered in all classes of compounds having CH_2_ chains ending with CH_3_, NH_2_, OH, SH or halogen. Another evidently important contributor is the H-bridge special group (no. 273) which—if found in the compound—increases the lipophilicity by 0.49 units.

The resulting goodness of fit R^2^ of 0.9543 for 2697 training compounds and the cross-validated correlation coefficient Q^2^ of 0.9448 for 2638 test molecules covering a logP range of between −4.41 (Ornithine, calc. −3.54) and 12.53 (Tetracosane, calc. 12.75) is within the same area of those published elsewhere, the average and standard deviations are within the experimental error. For comparison, Klopman *et al.* [19], using an extended group-contribution approach similar to the present, achieved an R^2^ of 0.93, a cross-validated Q^2^ of 0.926, a standard deviation of 0.38 (cross-validated 0.404), based on 1663 compounds. R. Wang’s XLOGP model [75] yielded, based on 1831 molecules, an R^2^ of 0.968 and a standard deviation of 0.37.

**Figure 5:**
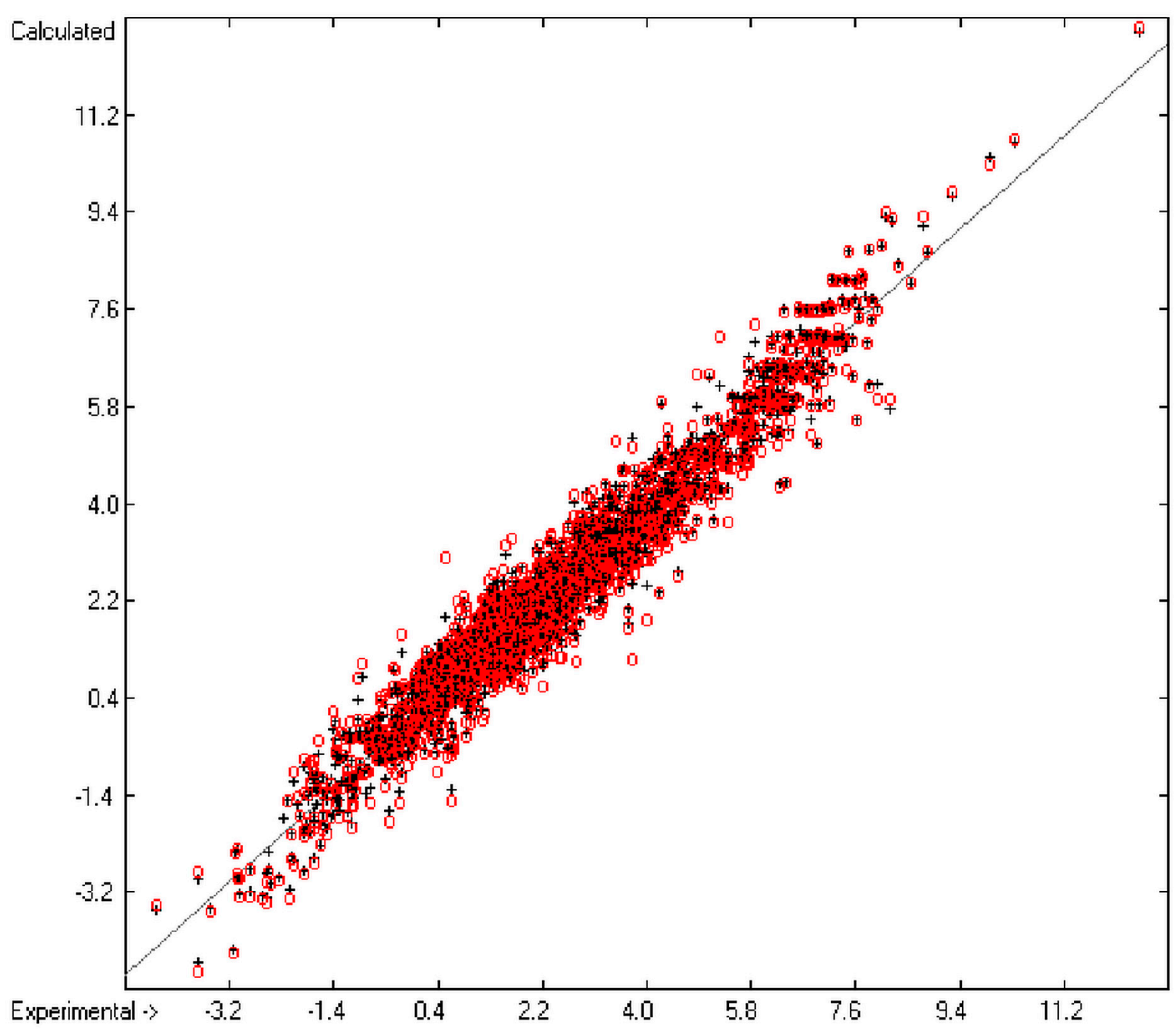
Correlation diagram of logP data (10-fold cross-validated: N = 2640, Q^2^ = 0.9451, slope = 0.96).

**Figure 6:**
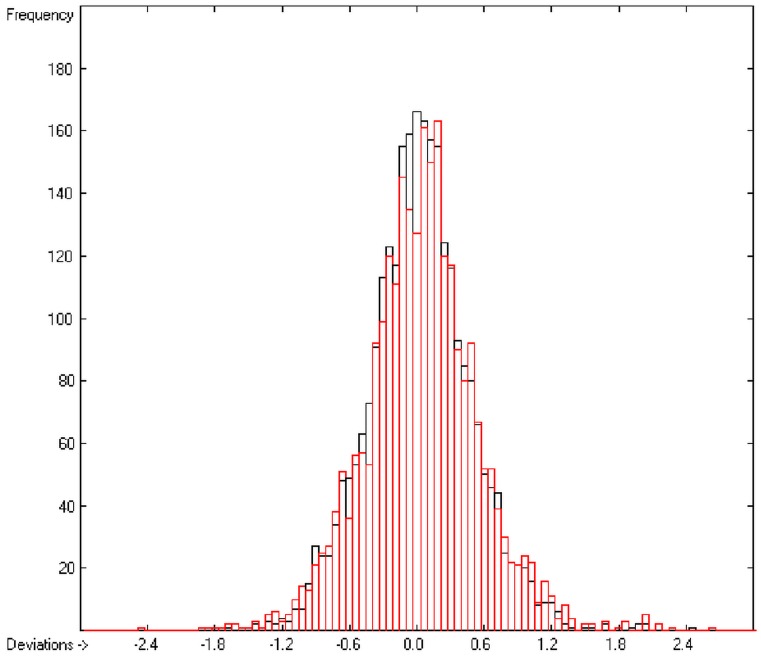
Histogram of logP data (S = 0.51).

An analysis of the error distribution shows that the calculated logP values of 2041 of the 2697 compounds (76%) deviates by less than or equal to the cross-validated standard error (S = 0.51) from the experimental value, while only 85 compounds (3%) are outliers with errors of more than twice that standard error. Figure 5 presents the correlation diagram of the logP data, showing that the data points of the cross-validated test set (red circles) in most cases overlap the black crosses of the training set, while the histogram (Figure 6) proves the evenness of the deviation distribution about the experimental values for both the training and test sets. The slope of the regression line in Figure 5 is slightly below 1 at 0.96.

**Table 8:** Atom group Contributions for LogP Calculations.

Nr	Atom Type	Neighbours	Contribution	Occurrences	Molecules
1	Const		0.25	2780	2780
2	C sp3	H3C	0.47	1969	1118
3	C sp3	H3N	0.39	435	300
4	C sp3	H3N(+)	−0.31	1	1
5	C sp3	H3O	−0.09	340	250
6	C sp3	H3S	−0.19	56	51
7	C sp3	H2C2	0.35	2064	714
8	C sp3	H2CN	0.36	701	387
9	C sp3	H2CN(+)	−0.34	23	19
10	C sp3	H2CO	−0.24	558	430
11	C sp3	H2CS	−0.38	76	59
12	C sp3	H2CF	−0.31	5	5
13	C sp3	H2CCl	0.48	51	38
14	C sp3	H2CBr	0.88	22	19
15	C sp3	H2CJ	0.99	3	3
16	C sp3	H2CP	2.89	1	1
17	C sp3	H2N2	1.57	4	4
18	C sp3	H2NO	0.15	5	5
19	C sp3	H2NS	0.64	3	3
20	C sp3	H2O2	−0.06	7	7
21	C sp3	H2S2	−1.23	4	4
22	C sp3	HC3	0.21	388	230
23	C sp3	HC2N	0.32	210	167
24	C sp3	HC2N(+)	−0.36	27	26
25	C sp3	HC2O	−0.18	389	193
26	C sp3	HC2S	−0.64	8	8
27	C sp3	HC2F	0.21	1	1
28	C sp3	HC2Cl	0.61	60	18
29	C sp3	HC2Br	0.71	7	5
30	C sp3	HCN2	1.00	6	5
31	C sp3	HCNO	0.64	20	20
32	C sp3	HCNS	0.52	30	30
33	C sp3	HCO2	−0.47	41	24
34	C sp3	HCOS	0.00	3	3
35	C sp3	HCOCl	0.11	3	1
36	C sp3	HCOBr	1.25	1	1
37	C sp3	HCOP	0.44	1	1
38	C sp3	HCF2	0.35	2	2
39	C sp3	HCCl2	1.15	10	9
40	C sp3	HOF2	−0.14	1	1
41	C sp3	C4	−0.09	131	101
42	C sp3	C3N	0.33	31	30
43	C sp3	C3N(+)	−0.78	1	1
44	C sp3	C3O	−0.23	71	59
45	C sp3	C3S	−0.46	17	17
46	C sp3	C3F	0.94	3	3
47	C sp3	C3Cl	0.56	28	8
48	C sp3	C3Br	0.70	1	1
49	C sp3	C2N2	−1.56	1	1
50	C sp3	C2NO	−0.04	5	5
51	C sp3	C2O2	0.22	6	6
52	C sp3	C2F2	0.63	2	2
53	C sp3	C2Cl2	0.73	11	10
54	C sp3	CNO2	1.35	1	1
55	C sp3	CF3	1.06	74	72
56	C sp3	CF2Cl	1.34	3	2
57	C sp3	CFCl2	1.34	3	2
58	C sp3	CCl3	1.71	20	18
59	C sp3	CCl2Br	0.00	1	1
60	C sp3	OF3	1.05	2	2
61	C sp3	SF3	1.24	7	7
62	C sp3	SFCl2	1.20	1	1
63	C sp3	SCl3	0.93	3	3
64	C sp2	H2=C	0.57	74	65
65	C sp2	H2=N	−0.77	1	1
66	C sp2	HC=C	0.25	390	249
67	C sp2	HC=N	−0.64	24	24
68	C sp2	HC=O	−0.48	32	32
69	C sp2	H=CN	0.02	104	90
70	C sp2	H=CN(+)	−0.23	17	17
71	C sp2	H=CO	0.70	13	12
72	C sp2	H=CS	−0.37	15	14
73	C sp2	H=CCl	0.77	10	8
74	C sp2	H=CBr	0.75	1	1
75	C sp2	HN=N	0.19	70	54
76	C sp2	HN=O	−0.38	12	11
77	C sp2	HO=O	−0.06	5	5
78	C sp2	H=NS	−0.35	4	4
79	C sp2	C2=C	0.19	150	126
80	C sp2	C2=N	−0.06	88	85
81	C sp2	C2=N(+)	1.59	1	1
82	C sp2	C2=O	−0.61	209	166
83	C sp2	C=CN	0.50	86	73
84	C sp2	C=CN(+)	−0.36	3	3
85	C sp2	C=CO	0.59	43	38
86	C sp2	C=CS	−0.15	19	14
87	C sp2	C=CF	0.02	3	3
88	C sp2	C=CCl	0.97	30	20
89	C sp2	C=CBr	0.93	4	4
90	C sp2	C=CJ	0.95	1	1
91	C sp2	C=CP	0.00	1	1
92	C sp2	=CN2	0.98	24	24
93	C sp2	=CN2(+)	0.65	11	11
94	C sp2	CN=N	0.32	68	65
95	C sp2	CN=N(+)	−0.10	2	2
96	C sp2	CN=O	−0.59	468	376
97	C sp2	C=NO	−0.60	1	1
98	C sp2	=CNO	0.61	4	4
99	C sp2	=CNO(+)	0.03	2	2
100	C sp2	CN=S	−0.24	8	8
101	C sp2	C=NS	−0.45	6	5
102	C sp2	=CNS	−0.52	5	5
103	C sp2	=CNCl	3.11	1	1
104	C sp2	=CNBr	1.00	5	3
105	C sp2	C=NCl	2.40	1	1
106	C sp2	CO=O	0.14	522	473
107	C sp2	CO=O(−)	−2.32	43	43
108	C sp2	C=OS	−1.34	4	4
109	C sp2	=COCl	1.54	1	1
110	C sp2	=CSBr	−1.69	1	1
111	C sp2	=CF2	0.42	1	1
112	C sp2	=CCl2	1.40	12	10
113	C sp2	=CBr2	1.48	1	1
114	C sp2	N2=N	0.77	28	27
115	C sp2	N2=N(+)	0.92	2	2
116	C sp2	N2=O	0.10	141	139
117	C sp2	N=NO	0.20	1	1
118	C sp2	N2=S	0.33	8	7
119	C sp2	N=NS	0.15	26	26
120	C sp2	N=NCl	1.79	3	3
121	C sp2	N=NBr	0.79	3	2
122	C sp2	NO=O	0.33	116	113
123	C sp2	=NOS	−0.06	1	1
124	C sp2	N=OS	−0.13	7	7
125	C sp2	NO=S	0.93	1	1
126	C sp2	=NS2	−1.52	2	2
127	C sp2	NS=S	−0.79	5	3
128	C sp2	=NSCl	0.71	1	1
129	C aromatic	H:C2	0.32	9660	2071
130	C aromatic	H:C:N	−0.40	277	192
131	C aromatic	H:C:N(+)	−0.94	24	23
132	C aromatic	H:N2	−1.08	10	10
133	C aromatic	:C3	0.16	390	171
134	C aromatic	C:C2	0.18	1982	1323
135	C aromatic	C:C:N	−0.49	73	63
136	C aromatic	C:C:N(+)	−0.45	4	4
137	C aromatic	:C2N	0.28	639	526
138	C aromatic	:C2N(+)	0.10	188	154
139	C aromatic	:C2:N	−0.10	93	72
140	C aromatic	:C2:N(+)	−0.01	20	20
141	C aromatic	:C2O	0.62	1096	749
142	C aromatic	:C2S	−0.15	177	143
143	C aromatic	:C2F	0.40	103	72
144	C aromatic	:C2Cl	0.86	1707	556
145	C aromatic	:C2Br	0.97	242	105
146	C aromatic	:C2J	1.32	50	34
147	C aromatic	:C2P	0.62	1	1
148	C aromatic	C:N2	−1.31	8	8
149	C aromatic	:C:N2	−1.33	1	1
150	C aromatic	:CN:N	0.68	36	32
151	C aromatic	:C:NO	0.57	26	18
152	C aromatic	:C:NS	−0.07	5	5
153	C aromatic	:C:NF	0.40	1	1
154	C aromatic	:C:NCl	0.31	18	16
155	C aromatic	:C:NBr	0.20	1	1
156	C aromatic	N:N2	0.36	54	42
157	C aromatic	:N3	−0.41	4	4
158	C aromatic	:N2O	0.55	9	9
159	C aromatic	:N2S	−0.51	3	3
160	C aromatic	:N2Cl	−0.23	8	7
161	C sp	H#C	−0.16	10	10
162	C sp	C#C	0.28	18	14
163	C sp	C#N	−0.18	90	86
164	C sp	N#N	0.68	2	2
165	C sp	#NS	−0.62	3	3
166	C sp	=N=S	1.86	22	21
167	N sp3	H2C	−1.37	56	56
168	N sp3	H2C(pi)	−0.84	313	287
169	N sp3	H2N	−0.58	17	17
170	N sp3	H2S	−1.13	36	36
171	N sp3	HC2	−1.19	64	63
172	N sp3	HC2(pi)	−0.89	237	213
173	N sp3	HC2(2pi)	−0.40	328	283
174	N sp3	HCN	−1.11	4	3
175	N sp3	HCN(pi)	−0.41	10	9
176	N sp3	HCN(2pi)	0.77	48	48
177	N sp3	HCO	−2.22	1	1
178	N sp3	HCO(pi)	−1.14	8	8
179	N sp3	HCS	−1.44	4	4
180	N sp3	HCS(pi)	−1.23	50	50
181	N sp3	HCP	−2.08	3	3
182	N sp3	HCP(pi)	−0.68	1	1
183	N sp3	C3	−1.31	136	120
184	N sp3	C3(pi)	−0.97	151	136
185	N sp3	C3(2pi)	−0.73	153	140
186	N sp3	C3(3pi)	−0.84	23	23
187	N sp3	C2N	−1.66	1	1
188	N sp3	C2N(pi)	−1.58	31	28
189	N sp3	C2N(2pi)	−0.75	54	50
190	N sp3	C2N(3pi)	−0.91	8	8
191	N sp3	C2O(pi)	−0.38	5	5
192	N sp3	C2S	−1.18	7	7
193	N sp3	C2S(pi)	0.07	7	6
194	N sp3	C2S(2pi)	0.99	2	2
195	N sp3	C2P	0.02	2	2
196	N sp3	CN2(2pi)	2.12	1	1
197	N sp3	CS2	−0.28	1	1
198	N sp3	CS2(pi)	−0.55	1	1
199	N sp2	H=C	−0.77	16	13
200	N sp2	C=C	−0.71	195	173
201	N sp2	C=N	−0.15	17	16
202	N sp2	=CN	0.36	100	81
203	N sp2	C=N(+)	−6.37	1	1
204	N sp2	=CN(+)	−0.85	2	2
205	N sp2	=CO	−0.26	34	29
206	N sp2	C=O	−0.74	2	2
207	N sp2	=CS	−1.52	6	5
208	N sp2	N=N	−0.61	29	22
209	N sp2	N=O	0.25	40	37
210	N aromatic	H2:C(+)	0.51	7	4
211	N aromatic	HC:C(+)	−0.13	4	3
212	N aromatic	C2:C(+)	−0.55	1	1
213	N aromatic	:C2	0.43	356	258
214	N aromatic	:C:N	−0.27	4	2
215	N(+) sp3	H3C	−0.81	29	29
216	N(+) sp3	H2C2	0.08	5	5
217	N(+) sp3	HC3	1.15	1	1
218	N(+) sp2	C=CO(−)	0.08	1	1
219	N(+) sp2	CO=O(−)	0.14	233	195
220	N(+) sp2	NO=O(−)	−0.21	2	2
221	N(+) sp2	O2=O(−)	0.53	1	1
222	N(+) aromatic	H:C2	0.92	4	4
223	N(+) aromatic	:C2O(−)	−0.58	20	20
224	N(+) sp	=C=N(−)	1.60	1	1
225	N(+) sp	=N2(−)	0.00	1	1
226	O	HC	−0.55	424	263
227	O	HC(pi)	−0.69	587	528
228	O	HN	−0.06	10	10
229	O	HN(pi)	0.02	6	6
230	O	C2	0.30	188	114
231	O	C2(pi)	−0.29	599	478
232	O	C2(2pi)	−0.78	298	277
233	O	CN	0.19	3	3
234	O	CN(pi)	0.43	7	7
235	O	CN(+)(pi)	−0.04	1	1
236	O	CN(2pi)	0.02	14	13
237	O	CS	0.05	4	2
238	O	CS(pi)	−1.19	4	4
239	O	CP	0.34	96	49
240	O	CP(pi)	−0.70	40	28
241	O	N2(2pi)	0.80	4	4
242	S2	HC	0.87	6	6
243	S2	HC(pi)	0.32	3	3
244	S2	C2	1.35	47	46
245	S2	C2(pi)	1.18	63	61
246	S2	C2(2pi)	1.42	49	48
247	S2	CN	0.00	3	3
248	S2	CN(2pi)	2.51	1	1
249	S2	CS	0.95	2	1
250	S2	CS(pi)	1.72	4	2
251	S2	CP	1.11	16	14
252	S2	CP(pi)	0.63	3	2
253	S2	N2	−2.67	2	2
254	S2	N2(2pi)	5.74	1	1
255	S4	C2=O	−0.71	9	9
256	S4	C2=O2	−0.22	14	14
257	S4	CO=O2	−0.36	2	1
258	S4	CN=O2	0.01	92	86
259	S4	C=O2F	0.62	2	2
260	S4	NO=O2	0.00	4	4
261	S4	N2=O2	1.27	5	5
262	S4	O2=O	0.71	1	1
263	P4	CO2=O	−1.84	2	2
264	P4	CO2=S	0.36	1	1
265	P4	COS=S	−2.39	1	1
266	P4	O3=O	−0.95	21	21
267	P4	O3=S	0.98	12	12
268	P4	O2=OS	0.43	1	1
269	P4	O2S=S	0.63	11	10
270	P4	O=OS2	−0.82	2	2
271	P4	N2O=O	−0.34	2	2
272	P4	NO=OS	−1.47	2	2
273	H	H Acceptor	0.49	151	139
274	Alkane	No of C atoms	0.16	274	30
275	Unsaturated HC	No of C atoms	0.05	1473	125
276	X(CH2)n	No of CH2 groups	0.10	1362	579
A	Based on				2780
B	Goodness of fit	R^2^	0.9543		2697
C	Deviation	Average	0.35		2697
D	Deviation	Standard	0.46		2697
E	K-fold cv	K	10.00		2638
F	Goodness of fit	Q^2^	0.9448		2638
G	Deviation	Average (cv)	0.38		2638
H	Deviation	Standard (cv)	0.51		2638

Wang *et al.* [75] added some further special groups as correction factors into their XLOGP program among which the amino acid indicator is worth mentioning because it seems to have a dramatically improving effect on the standard deviation in their program. The present method, however, does not require the incorporation of this indicator because the amino acids, being generally considered in solution as existing in the form of zwitterions, are accordingly included in the contribution calculation with the exception of those where the amino group is conjugated with a double-bonded or aryl moiety which lowers its basicity and thus causes the non-ionic form to be more stable. The experimental values confirm in all cases the zwitterionic form except—as expected—for *N*-phenylglycine. The difference of the logP between the non-ionic and the zwitterionic form (except for *N*-phenylglycine) amounts to ca. −1.87 units, as is shown in Table 9, close to Wang’s amino acid indicator value of −2.27. The calculated logP value of the dominant form is written in boldface.

A more opaque picture is found with compounds which undergo keto-enol tautomerism as shown in Table 10. While the calculated logP data for phenol, carbostyril, the 4-hydroxyform of uracil and acetylacetone and their tautomeric forms agree within the standard deviation with the experimental values, they can only be viewed as indicative in the case of acetone, cyclohexanone and 2-pyridone as both logP values for the respective tautomers exceed the standard deviations. Beyond this, acetylacetone is a tautomeric chameleon in that its tautomeric equilibrium strongly depends on the solvent: Allen and Dwek [65] showed that the percentage of enol decreased from 95% in cyclohexane to 75% in acetone and to 60% in dimethyl sulfoxide. In water the equilibrium is definitively shifted to the diketo side due to the strong intermolecular hydrogen bonding with the keto groups which obstructs the stabilizing effect of the intramolecular H-bridge [79].

**Table 9:** LogP of Amino acids.

Compound	Zwitterionic LogP Calc	Experiment LogP Exp	Non-ionic LogP Calc
Aspartic acid	**−2.93**	−3.70	−1.06
Threonine	**−3.48**	−3.50	−1.61
Glycine	**−3.22**	−3.00	−1.31
Ornithine	**−3.54**	−2.89	−1.67
Alanine	**−2.75**	−2.83	−0.88
Lysine	**−3.19**	−2.82	−0.92
Levodopa	**−1.90**	−2.74	−0.03
Histidine	**−3.27**	−2.52	−1.40
Cysteine	**−2.75**	−2.49	−0.78
Valine	**−2.08**	−2.10	−0.21
Methionine	**−2.10**	−1.87	−0.23
Tyrosine	**−1.51**	−1.80	0.36
Isoleucine	**−1.73**	−1.69	0.14
Leucine	**−1.73**	−1.57	0.14
Phenylalanine	**−1.12**	−1.43	0.75
Tryptophane	**−1.34**	−1.04	0.53
2-Amino-5-phenylvaleric acid	**−0.42**	−0.36	1.45
*N*-Phenylglycine	−0.66	0.62	**1.02**

**Table 10:** LogP of Ketones and Lactams.

Compound	Keto form LogP_Calc_	Experiment LogP_Exp_	Enol form LogP_Calc_	^a^
Acetone	**0.6**	−0.24	1.20	(**+**)
Cyclohexanone	**1.43**	0.81	1.82	(**+**)
Phenol	0.99	1.46	**1.76**	**+**
2-Pyridone	**0.02**	−0.58	1.09	(**+**)
Carbostyril	**1.49**	1.26	2.51	**+**
Uracil	−0.77	−1.07	**−1.25 ^b^**	**+**
Acetylacetone	**0.34**	0.4	1.23	**+**

^a^ Conformance with experimental data; ^b^ 4-hydroxy form.

**Figure 7:**
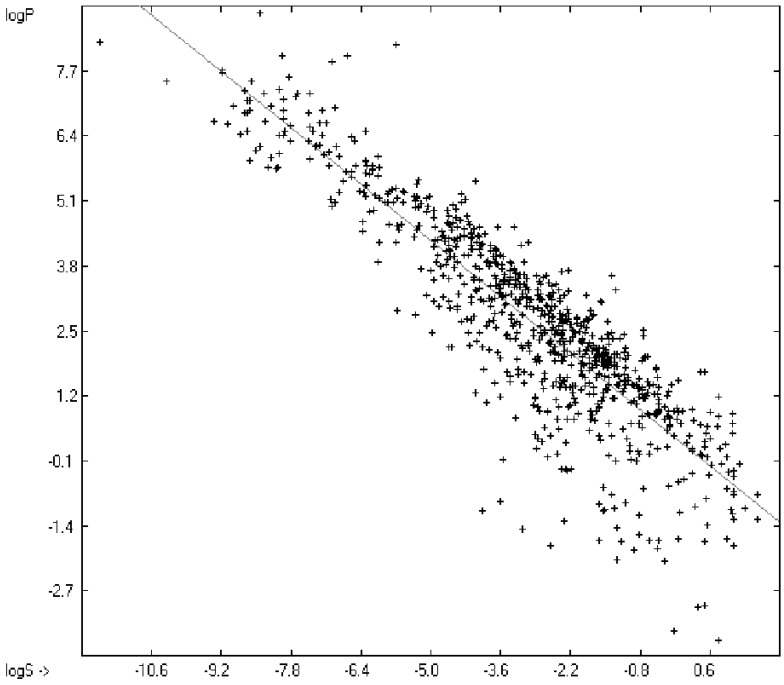
Correlation of logP with logS (N = 839, R^2^ = 0.7817).

### 3.5. Aqueous Solubility

Solubility in water is one of the most important properties of organic compounds since the first raindrops filled the oceans of this planet, otherwise the astrobiologist’s sentence: “where there is water, there is life” would be utterly senseless. Nowadays its importance is evident not only with respect to environmental considerations, e.g., in synthetical processes, but also in view of the biological activity of drugs, where it plays a key role. This has already been indirectly expressed in the descriptor logP_O/W_. While this descriptor defines the relative solubility of a solute between octanol and water, where saturation is not required, the aqueous solubility in mol/L, expressed as logS, *i.e.*, the logartihm of the solubility, is defined as the amount of solute in a saturated water solution. Nevertheless, as Banerjee *et al.* [80] showed on a selected set of 27 examples, there is a direct inverted correlation between logP and logS with a correlation coefficient of 0.94, resulting in the linear regression equation logP = 5.2 − 0.68 × logS. This compares with a calculation in the present work, where these two descriptors were correlated based on 839 compounds yielding a correlation coefficient of 0.78 and the regression equation logP = 0.32 − 0.80 × logS (Figure 7). Solubility data were extracted from a database provided by Hou *et al.* [81] and Wang *et al.* [82] on the ADME website [83] in the internet. Analogous to the atom groups calculations for logP net-charged compounds as well as strong acids are excluded from the logS calculations. In contrast to Hou’s and Wang’s approach, compounds that normally exist as twitter ions such as amino acids are entered in the twitter-ionic form in these calculations. In Table 11 the group contributions resulting from as set of 1487 molecules of a great structural variety are collected.

**Table 11:** Atom group Contributions for LogS Calculations.

Nr	Atom Type	Neighbours	Contribution	Occurrences	Molecules
1	Const		0.44	1492	1492
2	C sp3	H3C	−0.31	1571	806
3	C sp3	H3N	−0.87	173	113
4	C sp3	H3N(+)	−0.03	2	2
5	C sp3	H3O	−0.32	157	110
6	C sp3	H3S	−0.15	12	10
7	C sp3	H2C2	−0.32	2091	604
8	C sp3	H2CN	−0.85	278	144
9	C sp3	H2CN(+)	−0.68	6	5
10	C sp3	H2CO	−0.29	328	248
11	C sp3	H2CS	−0.10	43	30
12	C sp3	H2CP	−5.15	1	1
13	C sp3	H2CF	−0.83	1	1
14	C sp3	H2CCl	−0.62	41	34
15	C sp3	H2CBr	−1.29	18	16
16	C sp3	H2CJ	−1.75	5	5
17	C sp3	H2N2	−1.58	2	2
18	C sp3	H2NO	−0.99	9	9
19	C sp3	H2NS	−1.11	2	2
20	C sp3	H2O2	−0.67	6	6
21	C sp3	H2S2	−0.47	5	5
22	C sp3	H2SCl	−1.06	1	1
23	C sp3	HC3	−0.26	531	270
24	C sp3	HC2N	−0.81	72	60
25	C sp3	HC2N(+)	−0.71	23	22
26	C sp3	HC2O	−0.38	321	174
27	C sp3	HC2S	−0.46	8	6
28	C sp3	HC2F	−1.85	1	1
29	C sp3	HC2Cl	−0.89	28	16
30	C sp3	HC2Br	−1.02	4	4
31	C sp3	HC2J	−1.90	1	1
32	C sp3	HCO2	−0.69	28	17
33	C sp3	HCOBr	−4.65	1	1
34	C sp3	HCCl2	−1.24	13	12
35	C sp3	HCClBr	−1.05	1	1
36	C sp3	HOF2	−0.36	1	1
37	C sp3	C4	−0.17	234	162
38	C sp3	C3N	−0.64	16	16
39	C sp3	C3O	−0.15	91	82
40	C sp3	C3S	0.18	2	2
41	C sp3	C3F	−0.52	10	10
42	C sp3	C3Cl	−0.42	34	12
43	C sp3	C3Br	−0.69	1	1
44	C sp3	C2O2	−1.35	8	8
45	C sp3	C2Cl2	−2.25	11	10
46	C sp3	CF3	−1.09	24	24
47	C sp3	CF2Cl	−1.78	3	2
48	C sp3	CFCl2	−1.70	1	1
49	C sp3	CCl3	−2.12	12	11
50	C sp3	CCl2Br	0.00	1	1
51	C sp2	H2=C	−0.53	74	63
52	C sp2	HC=C	−0.27	338	204
53	C sp2	HC=N	−2.17	9	9
54	C sp2	HC=O	0.24	22	22
55	C sp2	H=CN	−0.54	23	21
56	C sp2	H=CO	−0.13	8	7
57	C sp2	H=CS	−0.32	9	6
58	C sp2	H=CCl	−1.00	6	5
59	C sp2	H=CBr	−0.88	2	1
60	C sp2	H=CJ	−1.83	2	1
61	C sp2	HN=N	−1.95	19	12
62	C sp2	HN=O	−0.19	2	2
63	C sp2	H=NO	−0.65	1	1
64	C sp2	HO=O	−0.22	7	7
65	C sp2	H=NS	−0.25	1	1
66	C sp2	C2=C	−0.26	153	128
67	C sp2	C2=N	−0.84	11	10
68	C sp2	C2=O	0.01	188	132
69	C sp2	C=CN	−1.05	25	22
70	C sp2	C=CO	−0.35	44	32
71	C sp2	C=CS	−0.14	5	5
72	C sp2	C=CF	−0.62	2	2
73	C sp2	C=CCl	−0.91	45	25
74	C sp2	C=CBr	−0.45	3	3
75	C sp2	CN=N	−1.67	9	8
76	C sp2	CN=O	−0.33	261	201
77	C sp2	C=NO	−1.87	5	5
78	C sp2	=CNO(+)	−1.68	2	2
79	C sp2	C=NS	−0.34	2	2
80	C sp2	CO=O	−0.06	306	266
81	C sp2	CO=O(−)	0.50	23	23
82	C sp2	C=OS	2.17	1	1
83	C sp2	=CCl2	−1.66	14	11
84	C sp2	=CBr2	−3.04	1	1
85	C sp2	N2=O	−1.46	98	95
86	C sp2	N2=S	−1.93	10	10
87	C sp2	NO=O	−0.55	48	45
88	C sp2	N=OS	−0.83	7	7
89	C sp2	=NS2	−1.09	1	1
90	C aromatic	H:C2	−0.30	4203	812
91	C aromatic	H:C:N	0.51	91	60
92	C aromatic	H:N2	0.37	7	7
93	C aromatic	:C3	−0.36	281	87
94	C aromatic	C:C2	−0.39	927	556
95	C aromatic	C:C:N	0.65	27	23
96	C aromatic	:C2N	−0.74	270	216
97	C aromatic	:C2N(+)	−0.72	68	50
98	C aromatic	:C2:N	−0.31	29	22
99	C aromatic	:C2O	−0.25	376	252
100	C aromatic	:C2S	−0.23	42	26
101	C aromatic	:C2F	−0.61	36	19
102	C aromatic	:C2Cl	−1.10	570	215
103	C aromatic	:C2Br	−1.53	38	24
104	C aromatic	:C2J	−1.47	21	16
105	C aromatic	:CN:N	−0.91	34	24
106	C aromatic	C:N2	0.10	2	2
107	C aromatic	:C:NO	0.13	12	12
108	C aromatic	:C:NCl	−0.87	5	5
109	C aromatic	N:N2	−0.94	24	15
110	C aromatic	:N2Cl	−0.54	7	7
111	C sp	H#C	−0.21	17	16
112	C sp	C#C	−0.55	19	17
113	C sp	C#N	−0.19	26	24
114	C sp	=N=S	−2.99	1	1
115	N sp3	H2C	0.93	12	9
116	N sp3	H2C(pi)	0.60	111	99
117	N sp3	H2N	0.61	4	4
118	N sp3	HC2	2.25	20	17
119	N sp3	HC2(pi)	1.29	75	66
120	N sp3	HC2(2pi)	0.74	211	158
121	N sp3	HCN	0.76	2	2
122	N sp3	HCN(pi)	0.34	7	6
123	N sp3	HCN(2pi)	−0.41	3	3
124	N sp3	C3	3.15	64	57
125	N sp3	C3(pi)	2.20	66	60
126	N sp3	C3(2pi)	1.62	80	75
127	N sp3	C3(3pi)	1.30	7	7
128	N sp3	C2N	1.44	1	1
129	N sp3	C2N(pi)	2.80	4	4
130	N sp3	C2N(2pi)	1.30	17	13
131	N sp3	C2N(3pi)	0.72	6	6
132	N sp2	C=C	1.49	35	32
133	N sp2	C=N	−0.22	3	2
134	N sp2	=CN	1.83	15	13
135	N sp2	=CO	1.52	7	7
136	N sp2	=CS	−0.37	2	1
137	N sp2	N=N	2.08	1	1
138	N sp2	N=O	−0.54	4	4
139	N(+) sp3	H3C	0.50	21	21
140	N(+) sp3	H2C2	0.29	1	1
141	N(+) sp3	HC3	1.97	1	1
142	N(+) sp2	CO=O(−)	−0.15	75	57
143	N(+) sp2	O2=O(−)	−0.54	5	2
144	N aromatic	:C2	−0.58	138	89
145	N aromatic	:C:N	0.11	2	1
146	O	HC	0.60	377	217
147	O	HC(pi)	0.34	306	240
148	O	HN(pi)	1.00	1	1
149	O	C2	0.69	106	63
150	O	C2(pi)	0.24	320	249
151	O	C2(2pi)	−0.25	76	72
152	O	CN(+)(pi)	−0.21	5	2
153	O	CN(2pi)	−0.30	6	6
154	O	CP	−0.07	78	36
155	O	CP(pi)	−1.23	25	20
156	P4	CO2=S	5.44	1	1
157	P4	O3=O	2.79	7	7
158	P4	O3=S	0.45	16	15
159	P4	O2=OS	0.67	2	2
160	P4	O2S=S	−1.43	14	13
161	S2	HC	−0.54	3	3
162	S2	HC(pi)	−0.84	2	2
163	S2	C2	−0.53	14	14
164	S2	C2(pi)	−1.03	12	12
165	S2	C2(2pi)	−1.02	25	25
166	S2	CP	0.21	16	15
167	S2	CS	−0.84	5	3
168	S2	N2(2pi)	0.00	1	1
169	S4	C2=O	0.91	3	3
170	S4	C2=O2	0.09	6	6
171	S4	C=OS	1.38	1	1
172	H	H Acceptor	−0.48	85	68
173	Alkane	No of C atoms	−0.33	282	39
174	Unsaturated HC	No of C atoms	−0.10	1350	121
175	X(CH2)n	No of CH2 groups	−0.12	1220	426
A	Based on		0.00		1492
B	Goodness of fit	R^2^	0.9051		1441
C	Deviation	Average	0.52		1441
D	Deviation	Standard	0.67		1441
E	K-fold cv	K	10.00		1419
F	Goodness of fit	Q^2^	0.8838		1419
G	Deviation	Average (cv)	0.57		1419
H	Deviation	Standard (cv)	0.74		1419

Hou’s group-additivity method [81], which based on a 2D-molecular topology, included—besides the atom groups in a SMARTS representation—the square of the molecular weight and a term called “hydrophobic carbon” to achieve better correlation. They achieved a correlation coefficient R of 0.96 (R^2^ = 0.92) and a standard deviation of 0.61, based on 1290 compounds. Wang’s [82] team, on the other hand, based their group-additivity approach on the solvent-accessible surface area (SASA) of each atom type and added the calculated logP value and the square of the molecular weight. Their best results showed a correlation coefficient R^2^ of 0.886 and a root mean square error of 0.705, using 1708 molecules.

**Figure 8:**
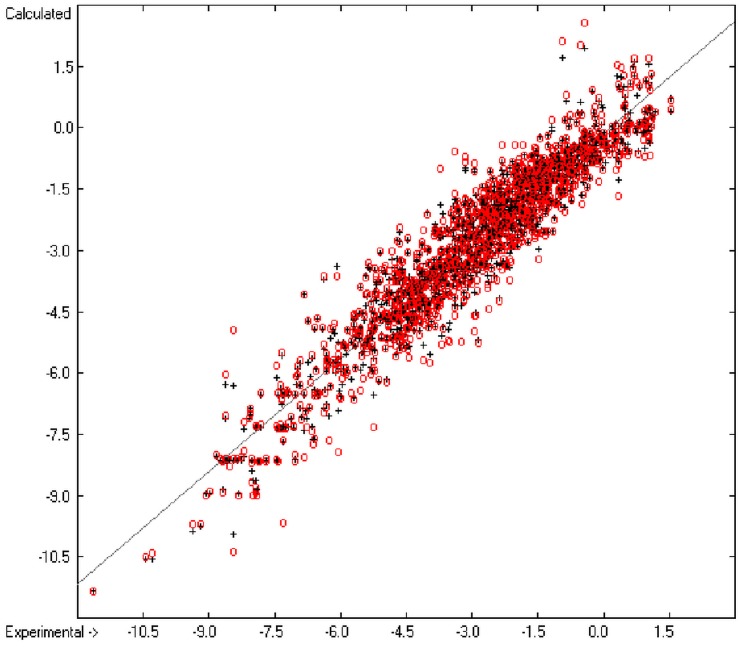
Correlation diagram of logS data (10-fold cross-validated: N = 1419, Q^2^ = 0.8838, slope = 0.92).

**Figure 9:**
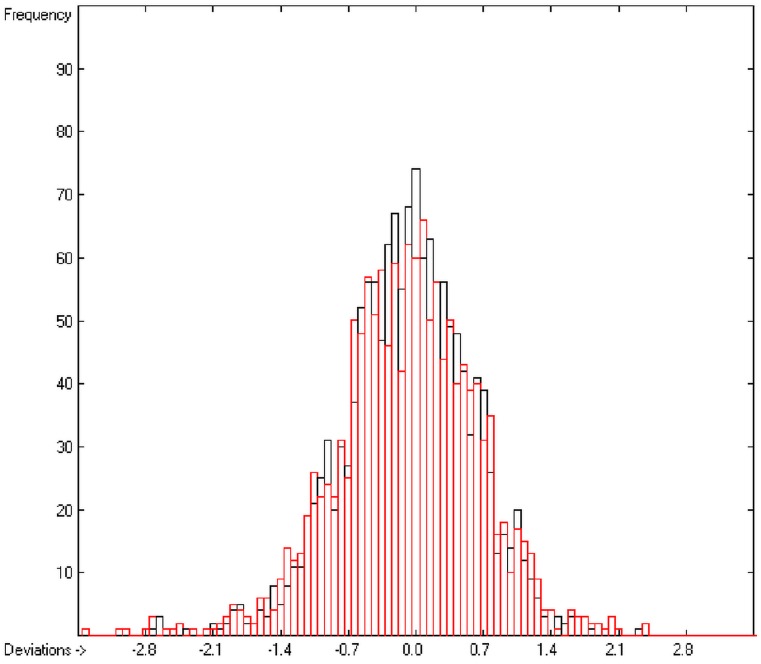
Histogram of logS data (S = 0.74).

The present list of groups encloses two groups which can be viewed as replacement of the Hou’s “hydrophobic carbon”: the terms “Alkane” and “Unsaturated HC” (no. 173 an 174). These two groups only apply for pure hydrocarbons. The last term “X(CH2)n” (no. 175) takes account of the hydrophobicity of alkyl chains. Group 172, on the other hand, considers the hydrophobic effect of intramolecular H-bridges. While Hou’s correlation is better (correleation coefficient R = 0.96, predictive Q = 0.94, mean error 0.57 units) than the present one, Wang’s approach is in the same range with a best leave-one-out Q^2^ of 0.886 and a root-mean-square error of 0.705 (compare with lines B, F and H in Table 11). Five outliers listed in Table 12 have been omitted from the calculations because their deviations exceed by far the expectable error range. Figure 8 and Figure 9 illustrate the distribution of the 1441 compounds’ experimental *vs.* calculated and 10-fold cross-validated logS data around the linear regression line, which exhibits a slope of 0.92 and a const of −0.14. The complete list of compounds and logS results is accessible in the supplementary material under “Experimental vs Calculated LogS Data Table.doc” and “Compounds List for LogS Calculations.sdf”.

**Table 12:** Molecules with extreme LogS Deviations.

Compound Name	logS Exp	logS Calc	Deviation
1-Hexadecanol	−7.26	−4.04	−3.22
1-Octadecanol	−8.40	−4.68	−3.72
Bromadiolone	−4.45	−9.33	4.88
Eicosane	−8.17	−12.54	4.37
Hexacosane	−8.33	−16.44	8.11

### 3.6. Refractivity

In their very instructive paper, Ghose and Crippen [8] explained in a detailed rationale the physical background of the molar refractivity, relating it to the volume of the molecule and of its constituting atoms and assigning the contributions of the atom groups to the atom volumes. As a consequence this assignment did not allow the simple least-squares method because it cannot guarantee positive-only contribution values. However, since the present paper is only interested in the final result, *i.e.*, the molar refractivity value as such, and is thus not bound to the constraints of the physical arguments—analogous to the total neglect of the chemical background for the calculations of the thermodynamic data—it is free to tentatively apply the same algorithm as used for the calculation of the other descriptors. Logically, it follows that the resulting atom group contributions cannot be assigned to any physical meaning.

The experimental data for the present studies are extracted from publications of Ghose and Crippen [8], complemented by V. N. Visvanadhan *et al.* [54]. Further molar refractivity (MR) values were calculated from the refractive indices (n_D_) and densities (d) provided by the CRC Handbook of Chemistry and Physics [84], using the equation MR = (n_D_^2^ − 1)/(n_D_^2^ + 2) × (M/d), where M is the molecular weight. The scope of compounds applicable for the refractivity calculation is limited to net-uncharged molecules, containing no further elements than H, B, C, N, O, S, P, Si and halogen and that are not strong acids.A complete list of compounds applied in the refractivity calculations can be viewed in the supplementary material in “Compounds List for Refractivity Calculations.sdf”, their results in “Experimental vs Calculated Refractivity Data Table.doc”.

The range of experimental refractivity values lies between 8.23 (methanol, calc. 8.09) and 242.2 (tripalmitin, calc. 243.12). The goodness of fit of the calculated values for both the training set as well as the 10-fold cross-validated data with experiment is excellent, as is shown in Table 13 on lines D and F. Accordingly, calculated refractivity values of 3388 out of 4122 compounds (82.2%) differ by the cross-validated standard deviation or less from experimental data. These results compare very well with those presented by Ghose and Crippen [8] which—based on 504 compounds—yielded a correlation coefficient R^2^ of 0.994 and a standard deviation of 1.269.

**Table 13:** Atom group Contributions for Refractivity Calculations.

Nr	Atom Type	Neighbours	Contribution	Occurrences	Molecules
1	B	HO2	28.10	1	1
2	B	C3	43.05	4	4
3	B	O3	52.64	6	6
4	C sp3	H3C	5.68	5655	2801
5	C sp3	H3N	12.60	200	122
6	C sp3	H3N(+)	15.42	3	2
7	C sp3	H3O	13.12	418	305
8	C sp3	H3S	14.13	33	29
9	C sp3	H3P	12.09	6	5
10	C sp3	H3Si	10.03	400	88
11	C sp3	H2BC	−8.53	12	4
12	C sp3	H2C2	4.62	9101	2185
13	C sp3	H2CN	11.48	601	317
14	C sp3	H2CN(+)	14.31	19	17
15	C sp3	H2CO	12.08	1514	999
16	C sp3	H2CS	12.86	167	116
17	C sp3	H2CP	11.20	9	5
18	C sp3	H2CF	5.64	19	18
19	C sp3	H2CCl	10.49	203	173
20	C sp3	H2CBr	13.49	123	109
21	C sp3	H2CJ	18.67	36	31
22	C sp3	H2CSi	8.92	71	41
23	C sp3	H2N2	18.44	2	2
24	C sp3	H2NO	20.34	1	1
25	C sp3	H2NS	19.76	1	1
26	C sp3	H2O2	19.35	19	19
27	C sp3	H2OCl	17.90	8	7
28	C sp3	H2OBr	20.97	2	2
29	C sp3	H2S2	20.26	2	2
30	C sp3	H2SCl	18.85	2	2
31	C sp3	H2SiCl	14.75	6	5
32	C sp3	H2SiBr	17.85	4	3
33	C sp3	H2Si2	12.25	2	2
34	C sp3	HC3	3.53	993	706
35	C sp3	HC2N	10.44	85	66
36	C sp3	HC2N(+)	13.23	6	6
37	C sp3	HC2O	11.00	387	326
38	C sp3	HC2P	10.02	2	1
39	C sp3	HC2S	12.08	23	19
40	C sp3	HC2F	4.43	1	1
41	C sp3	HC2Cl	9.41	56	53
42	C sp3	HC2Br	12.45	60	53
43	C sp3	HC2J	17.86	7	7
44	C sp3	HCN2(+)	23.26	1	1
45	C sp3	HCNCl(+)	18.88	2	2
46	C sp3	HCO2	18.28	43	37
47	C sp3	HCOCl	17.16	10	8
48	C sp3	HCOBr	21.62	1	1
49	C sp3	HCS2	20.19	1	1
50	C sp3	HCF2	5.67	7	7
51	C sp3	HCFCl	10.61	7	6
52	C sp3	HCFBr	13.45	1	1
53	C sp3	HCCl2	15.35	27	26
54	C sp3	HCClBr	19.00	5	4
55	C sp3	HCBr2	21.02	13	12
56	C sp3	HCJ2	31.52	1	1
57	C sp3	HNO2	24.81	2	2
58	C sp3	HO3	25.82	4	4
59	C sp3	HOF2	13.75	1	1
60	C sp3	HOCl2	23.45	1	1
61	C sp3	HS3	28.81	1	1
62	C sp3	HSiCl2	19.68	5	4
63	C sp3	C4	2.52	249	215
64	C sp3	C3N	9.31	20	16
65	C sp3	C3N(+)	11.84	2	2
66	C sp3	C3O	10.02	101	94
67	C sp3	C3S	11.33	6	4
68	C sp3	C3F	3.33	2	2
69	C sp3	C3Cl	8.44	6	6
70	C sp3	C3Br	11.41	6	6
71	C sp3	C3J	17.06	2	2
72	C sp3	C3Si	7.55	1	1
73	C sp3	C2NCl(+)	18.58	1	1
74	C sp3	C2O2	17.33	6	6
75	C sp3	C2OCl	16.18	1	1
76	C sp3	C2F2	5.07	79	27
77	C sp3	C2FCl	9.19	2	2
78	C sp3	C2Cl2	14.19	17	14
79	C sp3	C2ClBr	17.34	1	1
80	C sp3	C2Br2	20.20	5	5
81	C sp3	C2J2	30.59	1	1
82	C sp3	CNF2	11.34	6	2
83	C sp3	CNF2(+)	15.04	2	1
84	C sp3	CO3	24.67	2	2
85	C sp3	CO2Si	19.82	1	1
86	C sp3	COF2	11.83	2	2
87	C sp3	CF3	6.09	77	61
88	C sp3	CF2Cl	10.86	10	7
89	C sp3	CF2Br	13.41	5	4
90	C sp3	CFCl2	15.48	9	7
91	C sp3	CCl3	20.40	33	31
92	C sp3	CCl2Br	25.75	1	1
93	C sp3	CBr3	29.60	4	3
94	C sp3	O4	31.58	3	3
95	C sp3	OCl3	27.56	1	1
96	C sp3	SCl3	34.86	1	1
97	C sp2	H2=C	5.46	470	408
98	C sp2	HC=C	4.64	1233	735
99	C sp2	HC=N	9.93	15	14
100	C sp2	HC=N(+)	14.93	1	1
101	C sp2	HC=O	6.34	113	110
102	C sp2	H=CN	11.20	28	20
103	C sp2	H=CN(+)	13.78	2	2
104	C sp2	H=CO	2.27	78	69
105	C sp2	H=CP	10.01	1	1
106	C sp2	H=CS	12.26	32	27
107	C sp2	H=CF	5.18	1	1
108	C sp2	H=CCl	10.19	22	19
109	C sp2	H=CBr	13.12	11	9
110	C sp2	H=CJ	18.20	1	1
111	C sp2	H=CSi	8.81	17	12
112	C sp2	HN=N	16.23	8	7
113	C sp2	HN=O	12.89	11	11
114	C sp2	H=NO	6.55	3	3
115	C sp2	HO=O	4.04	23	22
116	C sp2	H=NS	16.43	1	1
117	C sp2	C2=C	3.52	385	292
118	C sp2	C2=N	8.85	20	17
119	C sp2	C2=O	5.08	330	310
120	C sp2	C2=S	11.72	1	1
121	C sp2	C=CN	10.90	16	14
122	C sp2	C=CN(+)	13.34	1	1
123	C sp2	C=CO	1.65	56	51
124	C sp2	C=CS	11.28	14	13
125	C sp2	C=CF	4.49	9	6
126	C sp2	C=CCl	9.42	43	31
127	C sp2	C=CBr	12.05	14	14
128	C sp2	C=CJ	18.20	1	1
129	C sp2	CN=N	16.53	1	1
130	C sp2	CN=O	11.80	51	48
131	C sp2	C=NO	6.72	7	7
132	C sp2	CO=O	2.82	919	734
133	C sp2	C=NS	15.76	3	3
134	C sp2	C=OP	11.59	1	1
135	C sp2	C=OS	12.58	4	4
136	C sp2	C=OF	5.61	1	1
137	C sp2	C=OCl	11.36	73	64
138	C sp2	C=OBr	14.01	3	3
139	C sp2	C=OJ	20.47	1	1
140	C sp2	=CNO(+)	12.72	1	1
141	C sp2	=CO2	-1.06	2	2
142	C sp2	=COS	8.87	1	1
143	C sp2	=COCl	6.53	1	1
144	C sp2	=COBr	9.23	1	1
145	C sp2	=COJ	14.39	1	1
146	C sp2	=CSCl	17.00	6	4
147	C sp2	=CSBr	19.87	4	3
148	C sp2	=CSJ	24.42	1	1
149	C sp2	=CF2	6.79	5	3
150	C sp2	=CFCl	10.30	4	3
151	C sp2	=CCl2	15.25	13	11
152	C sp2	=CBr2	20.62	2	2
153	C sp2	N2=O	17.81	4	4
154	C sp2	N2=S	24.84	2	2
155	C sp2	NO=O	10.22	14	14
156	C sp2	NO=S	18.88	1	1
157	C sp2	N=OS	20.90	2	2
158	C sp2	N=OCl	17.76	1	1
159	C sp2	=NOCl	10.42	1	1
160	C sp2	=NS2	25.98	2	2
161	C sp2	=NSCl	21.31	1	1
162	C sp2	=NSBr	25.29	1	1
163	C sp2	O2=O	0.69	13	12
164	C sp2	O=OS	-13.11	1	1
165	C sp2	O=OCl	8.89	13	12
166	C sp2	OS=S	18.97	1	1
167	C sp2	S2=S	31.98	1	1
168	C sp2	=OSCl	19.34	1	1
169	C aromatic	H:C2	4.45	5576	1171
170	C aromatic	H:C:N	6.28	141	92
171	C aromatic	H:N2	8.19	1	1
172	C aromatic	:C3	4.43	153	77
173	C aromatic	C:C2	3.55	1231	850
174	C aromatic	C:C:N	5.52	52	44
175	C aromatic	C:C:N(+)	6.48	2	1
176	C aromatic	:C2N	11.36	164	149
177	C aromatic	:C2N(+)	13.98	57	50
178	C aromatic	:C2:N	6.26	15	14
179	C aromatic	:C2O	1.71	341	264
180	C aromatic	:C2S	11.96	39	36
181	C aromatic	:C2F	4.40	130	69
182	C aromatic	:C2Cl	9.11	119	92
183	C aromatic	:C2Br	11.94	59	53
184	C aromatic	:C2J	17.01	19	18
185	C aromatic	:C2P	10.22	10	7
186	C aromatic	:C2Si	7.66	45	28
187	C aromatic	:CN:N	14.09	1	1
188	C aromatic	C:N2	7.20	1	1
189	C aromatic	:C:NO	4.26	3	3
190	C aromatic	:C:NF	5.45	1	1
191	C aromatic	:C:NCl	11.18	3	3
192	C aromatic	:C:NBr	13.88	1	1
193	C aromatic	:C:NJ	20.14	1	1
194	C aromatic	N:N2	16.66	5	2
195	C aromatic	:N2Cl	11.88	1	1
196	C sp	H#C	4.25	73	67
197	C sp	C#C	4.09	164	111
198	C sp	C#N	5.53	121	104
199	C sp	#CO	1.82	5	5
200	C sp	#CSi	7.36	2	1
201	C sp	#CCl	9.68	3	2
202	C sp	#CBr	12.15	2	2
203	C sp	#CJ	17.23	1	1
204	C sp	N#N	11.94	2	2
205	C sp	#NP	−4.48	1	1
206	C sp	#NS	12.69	4	4
207	C sp	=C2	4.99	10	10
208	C sp	=C=O	5.80	3	2
209	C sp	=N2	15.59	1	1
210	C sp	=N=O	10.16	16	13
211	C sp	#NO	4.55	1	1
212	C sp	=N=S	18.38	12	12
213	N sp3	H2C	−2.38	127	113
214	N sp3	H2C(pi)	−2.88	77	71
215	N sp3	H2N	4.05	8	8
216	N sp3	HC2	−10.34	82	80
217	N sp3	HC2(pi)	−10.41	43	42
218	N sp3	HC2(2pi)	−10.98	13	13
219	N sp3	HCN	−3.22	10	6
220	N sp3	HCN(pi)	−4.03	4	4
221	N sp3	HCN(+)(pi)	4.14	2	2
222	N sp3	HCN(2pi)	−3.92	3	3
223	N sp3	HCO	−0.78	1	1
224	N sp3	HSi2	−0.18	4	2
225	N sp3	C3	−17.69	115	101
226	N sp3	C3(pi)	−18.04	60	57
227	N sp3	C3(2pi)	−18.33	17	17
228	N sp3	C3(3pi)	−20.10	3	3
229	N sp3	C2N	−11.17	4	4
230	N sp3	C2N(pi)	−10.99	8	8
231	N sp3	C2N(2pi)	−12.24	6	6
232	N sp3	C2N(3pi)	−13.16	1	1
233	N sp3	C2N(+)(pi)	−3.70	2	2
234	N sp3	C2N(+)(2pi)	−3.95	2	2
235	N sp3	C2O	−8.33	1	1
236	N sp3	C2P	−7.63	10	4
237	N sp3	C2Si	−11.17	2	2
238	N sp3	CCl2(pi)	9.04	1	1
239	N sp2	H=C	−1.83	1	1
240	N sp2	C=C	−9.29	60	56
241	N sp2	C=N	−2.00	13	7
242	N sp2	C=N(+)	0.56	6	6
243	N sp2	=CN	−2.44	11	9
244	N sp2	=CO	−0.50	17	16
245	N sp2	=CP	−7.67	1	1
246	N sp2	=CS	2.87	3	2
247	N sp2	N=N	0.22	1	1
248	N sp2	N=O	5.39	6	6
249	N sp2	O=O	−0.32	11	11
250	N(+) sp3	HC3	−21.24	1	1
251	N(+) sp2	C=NO(−)	−2.16	2	2
252	N(+) sp2	CO=O(−)	−2.94	90	79
253	N(+) sp2	NO=O(−)	0.00	6	6
254	N(+) sp2	O2=O(−)	0.75	14	11
255	N aromatic	:C2	−1.62	114	101
256	N aromatic	:C:N	0.35	6	3
257	N(+) aromatic	:C2O(−)	0.00	1	1
258	N(+) sp	C#C(−)	−3.74	3	3
259	N(+) sp	=C=N(−)	−2.82	1	1
260	N(+) sp	=N2(−)	1.08	4	4
261	O	HC	−5.03	516	451
262	O	HC(pi)	4.48	220	210
263	O	HN	0.00	2	2
264	O	HN(pi)	0.72	10	10
265	O	HO	2.64	5	5
266	O	HS	7.50	3	3
267	O	HP	5.69	6	5
268	O	HSi	1.08	2	2
269	O	BC	−22.37	18	6
270	O	BC(pi)	−10.61	2	1
271	O	C2	−13.20	392	268
272	O	C2(pi)	−3.86	1009	801
273	O	C2(2pi)	5.33	104	103
274	O	CN(pi)	0.00	11	11
275	O	CN(+)(pi)	0.91	14	11
276	O	CN(2pi)	0.27	5	5
277	O	CO	−5.35	15	10
278	O	CO(pi)	4.66	2	2
279	O	CP	−2.44	134	57
280	O	CP(pi)	6.37	39	22
281	O	CS	−1.88	35	23
282	O	CSi	−7.23	83	31
283	O	CSi(pi)	1.87	17	8
284	O	CCl	0.58	1	1
285	O	N2(2pi)	−4.29	1	1
286	O	P2	7.77	10	6
287	O	Si2	−1.25	114	29
288	P3	H2C	4.10	1	1
289	P3	HC2	0.00	1	1
290	P3	C3	−10.10	3	3
291	P3	C2Cl	−0.60	1	1
292	P3	CCl2	13.67	3	3
293	P3	O3	−3.00	9	9
294	P3	O2Cl	5.19	1	1
295	P3	OCl2	15.89	1	1
296	P4	HO2=O	1.09	5	5
297	P4	C2O=O	−8.92	1	1
298	P4	CO2=O	−6.49	8	8
299	P4	CO2=S	2.11	1	1
300	P4	C=OCl2	20.04	1	1
301	P4	CNO=O	10.07	1	1
302	P4	N3=O	−5.27	1	1
303	P4	N2O=O	−2.94	2	1
304	P4	N2=OF	0.31	1	1
305	P4	NO2=O	4.89	1	1
306	P4	O3=O	−3.70	26	19
307	P4	O3=O(-)	−3.24	1	1
308	P4	O3=S	3.88	12	10
309	P4	O2=OS	−3.47	3	3
310	P4	O2=OF	0.87	1	1
311	P4	O2=OCl	5.37	2	2
312	P4	O2S=S	4.28	2	2
313	P4	O2=SCl	13.26	1	1
314	P4	O=OCl2	15.32	1	1
315	S2	HC	0.44	56	46
316	S2	HC(pi)	0.29	11	10
317	S2	C2	−8.61	53	49
318	S2	C2(pi)	−8.17	29	27
319	S2	C2(2pi)	−8.93	34	34
320	S2	CP	3.36	5	5
321	S2	CS	−0.28	17	9
322	S2	CS(pi)	−14.19	2	1
323	S2	CCl	0.00	1	1
324	S2	N2(2pi)	−5.11	1	1
325	S2	S2	9.07	1	1
326	S4	C2=O	−7.94	3	3
327	S4	C2=O2	−7.64	7	7
328	S4	CO=O2	−4.38	10	10
329	S4	C=OCl	−8.76	1	1
330	S4	C=OS	1.60	1	1
331	S4	C=O2F	0.83	1	1
332	S4	C=O2Cl	6.94	7	7
333	S4	N=O2Cl	10.21	1	1
334	S4	O=OCl	11.29	1	1
335	S4	O2=O	0.31	8	8
336	S4	O2=O2	0.04	4	4
337	S4	O=O2Cl	10.88	2	2
338	S4	O=O2F	5.35	1	1
339	Si	H3C	7.40	4	3
340	Si	H2C2	1.36	4	4
341	Si	H2CCl	11.60	1	1
342	Si	HC3	−4.55	5	5
343	Si	HC2O	0.65	2	1
344	Si	HC2Cl	5.67	2	2
345	Si	HCO2	5.53	19	6
346	Si	C4	−9.88	18	16
347	Si	C3N	−4.24	4	3
348	Si	C3O	−5.06	45	26
349	Si	C3F	−5.72	1	1
350	Si	C3Cl	−0.46	11	11
351	Si	C3Br	2.61	1	1
352	Si	C3Si	−4.39	2	1
353	Si	C2N2	1.41	3	1
354	Si	C2O2	−0.16	85	24
355	Si	C2SiCl	5.07	2	1
356	Si	C2F2	−1.31	2	2
357	Si	C2Cl2	9.60	9	9
358	Si	CO3	5.01	17	17
359	Si	CF3	3.18	1	1
360	Si	CCl3	19.47	16	15
361	Si	CBr3	28.52	1	1
362	Si	O4	10.00	5	5
363	Si	O3Cl	15.16	1	1
364	Si	OCl3	25.28	1	1
A	Based on				4300
B	Goodness of fit	R^2^	0.9989		4122
C	Deviation	Average	0.44		4122
D	Deviation	Standard	0.66		4122
E	K-fold cv	K	10.00		4039
F	Goodness of fit	Q^2^	0.9988		4039
G	Deviation	Average (cv)	0.46		4039
H	Deviation	Standard (cv)	0.70		4039

**Figure 10:**
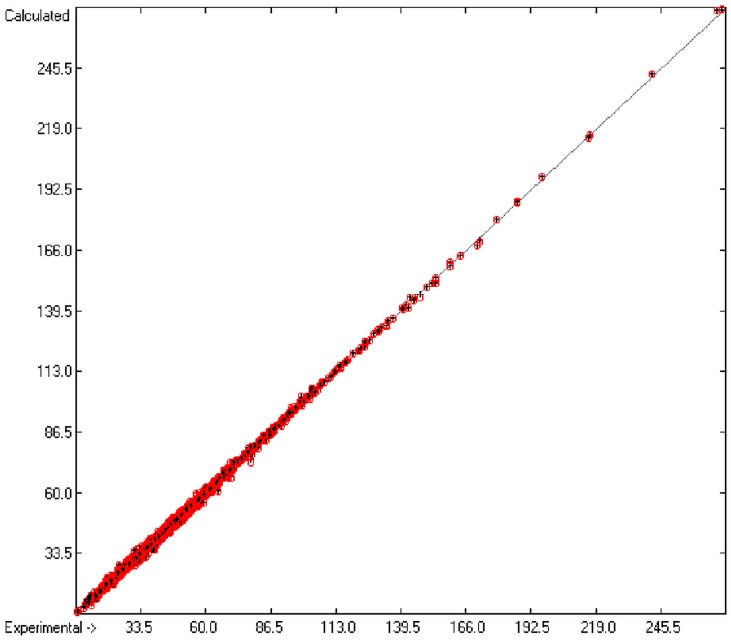
Correlation diagram of refractivity data (10-fold cross-validated: N = 4039, Q^2^ = 0.9988, slope = 1.0).

**Figure 11:**
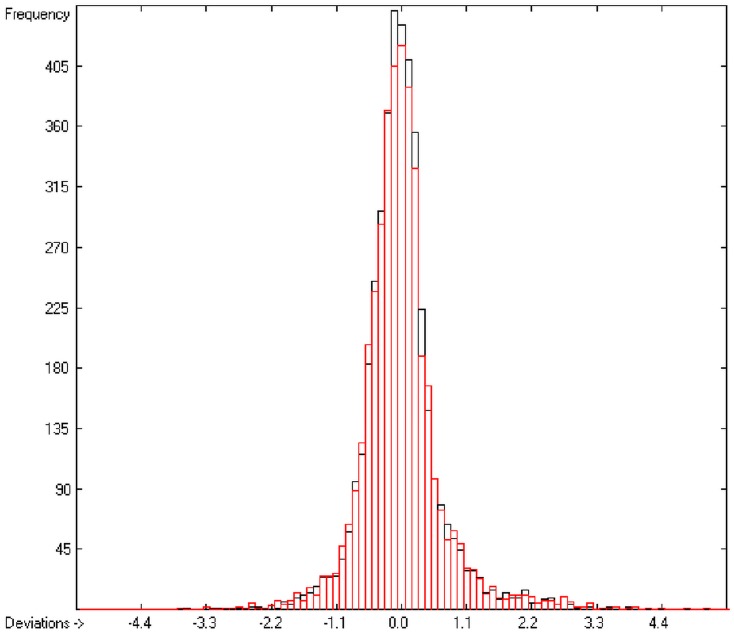
Histogram of refractivity data (S = 0.70).

In view of the large number of experimental data for the calculation of the atom group contributions, their excellent correlation coefficients R^2^ and Q^2^ and the solid physical foundation of the refractivity value itself on the molecular volume [8] it is safe to say that experimental refractivity values that deviate by more than 4 times the cross-validated standard deviation (*i.e.*, >2.8 units) from the calculated data, also observed and discussed in detail in Ghose and Crippen’s paper [8], are most probably based on incorrectly measured values of either the refractive index or the density or both or are typing errors in the source text as their deviation can no longer be ascribed to a temperature dependence of the measurements and therefore would require a re-examination. The excellent compliance between experimental and calculated refractivity data of more than 4000 compounds on the other hand—as visualized in Figure 10 and Figure 11—is proof that the present atomic-groups contribution method and the underlying algorithm are appropriate for refractivity calculations as long as one abstains from the attempt to interpret the group contribution values themselves. These results also prove that this group-additivity method is a very reliable tool for the indirect determination of the density of a compound from a simple measurement of its refractive index.

### 3.7. Polarizability

Miller and Savchik [9] were the first to apply an atomic-groups contribution method for the calculation of the molecular polarizability which, however, is only based on the atoms and their degree of hybridisation, neglecting the nature of their neighbourhood atoms. This method requires that the sum of the contributions of the atomic hybrid components is squared and then multiplied by 4/N, where N is the total number of electrons, to receive the molecular polarizability. Although this method is only based on 20 atom group parameters, the deviations between the experimental and calculated molecular polarizabilities are in line with the experimental variances [10].

In contrast to Miller’s approach the present atom groups include—besides the atomic degree of hybridisation—the central atom’s immediate neighbourhood atoms, which on the one hand has the disadvantage of requiring a larger number of atom groups to enable the calculation of a large number of compounds, but on the other hand is easily extendable to new atom groups if required. As will be shown, the results and standard deviation are comparable to Miller’s work [10].

The experimental data for the evaluation of the group contributions, listed in Table 14, are extracted from the Handbook of Chemistry and Physics [85] and Miller’s publication [10], enabling a direct comparison of the results.A table of these results can be accessed in the supplementary material under “Experimental vs Calculated Polarizability Data Table.doc”, the corresponding list of compounds in an SD file called “Compounds List for Polarizability Calculations.sdf”.

**Table 14:** Atom group Contributions for Polarizability Calculations.

Nr	Atom Type	Neighbours	Contribution	Occurrences	Molecules
1	Const		0.62	406	406
2	C sp3	H3C	1.92	351	219
3	C sp3	H3N	4.67	16	12
4	C sp3	H3O	3.50	32	23
5	C sp3	H3S	3.42	6	3
6	C sp3	H2C2	1.80	410	123
7	C sp3	H2CN	4.52	25	16
8	C sp3	H2CN(+)	4.56	2	2
9	C sp3	H2CO	3.35	78	51
10	C sp3	H2CS	3.14	3	2
11	C sp3	H2CF	2.19	11	11
12	C sp3	H2CCl	3.90	19	17
13	C sp3	H2CBr	4.86	15	14
14	C sp3	H2CJ	7.26	3	3
15	C sp3	H2O2	5.49	1	1
16	C sp3	H2OCl	5.69	1	1
17	C sp3	HC3	1.80	16	13
18	C sp3	HC2N	4.43	1	1
19	C sp3	HC2O	3.13	5	4
20	C sp3	HC2Cl	3.72	12	12
21	C sp3	HC2Br	5.13	1	1
22	C sp3	HCNCl(+)	7.56	2	2
23	C sp3	HCO2	6.37	5	3
24	C sp3	HCF2	2.07	1	1
25	C sp3	HCCl2	5.71	4	4
26	C sp3	C4	1.47	13	10
27	C sp3	C3N(+)	4.26	1	1
28	C sp3	C3Cl	6.11	1	1
29	C sp3	CF3	2.65	4	3
30	C sp3	CF2Cl	4.02	5	4
31	C sp3	CCl3	7.90	3	3
32	C sp3	O4	9.21	1	1
33	C sp2	H2=C	1.96	39	31
34	C sp2	HC=C	1.95	70	40
35	C sp2	HC=N	2.38	4	4
36	C sp2	HC=O	2.05	8	8
37	C sp2	H=CN	2.32	13	9
38	C sp2	H=CO	1.65	2	1
39	C sp2	H=CS	3.15	4	2
40	C sp2	H=CCl	3.80	10	8
41	C sp2	H=CBr	4.88	4	4
42	C sp2	H=CJ	6.72	1	1
43	C sp2	HN=N	4.11	6	5
44	C sp2	HN=O	4.32	3	3
45	C sp2	HO=O	3.17	4	4
46	C sp2	C2=C	1.83	18	14
47	C sp2	C2=N	3.64	2	1
48	C sp2	C2=O	2.48	19	14
49	C sp2	C=CN	2.13	4	4
50	C sp2	C=CO	1.82	3	3
51	C sp2	C=CCl	4.46	2	1
52	C sp2	CN=N	4.66	2	2
53	C sp2	CN=O	3.88	8	8
54	C sp2	CO=O	2.63	33	31
55	C sp2	C=OCl	4.07	1	1
56	C sp2	=CN2	3.55	2	2
57	C sp2	=CF2	0.20	1	1
58	C sp2	=CCl2	5.43	2	2
59	C sp2	N2=N	4.20	1	1
60	C sp2	N2=O	3.46	3	3
61	C sp2	O2=O	3.47	2	2
62	C sp2	O=OCl	4.72	2	2
63	C aromatic	H:C2	1.68	777	130
64	C aromatic	H:C:N	2.51	17	9
65	C aromatic	H:N2	2.86	1	1
66	C aromatic	:C3	1.91	125	40
67	C aromatic	C:C2	1.52	116	52
68	C aromatic	C:C:N	2.22	4	3
69	C aromatic	:C2N	3.59	27	24
70	C aromatic	:C2N(+)	3.94	11	8
71	C aromatic	:C2:N	2.35	17	8
72	C aromatic	:C2O	2.50	21	12
73	C aromatic	:C2S	3.45	6	3
74	C aromatic	:C2F	1.51	42	15
75	C aromatic	:C2Cl	3.47	18	12
76	C aromatic	:C2Br	4.49	10	9
77	C aromatic	:C2J	6.48	5	5
78	C sp	H#C	1.46	12	10
79	C sp	C#C	1.59	12	9
80	C sp	C#N	1.92	22	19
81	C sp	#CCl	3.99	1	1
82	C sp	#CBr	5.31	1	1
83	C sp	=C=O	1.82	1	1
84	N sp3	H2C	−1.13	7	6
85	N sp3	H2C(pi)	−0.53	25	22
86	N sp3	H2N	1.41	5	4
87	N sp3	HC2	−3.29	3	3
88	N sp3	HC2(pi)	−3.78	5	5
89	N sp3	HC2(2pi)	−1.24	11	7
90	N sp3	HCN(pi)	−1.12	1	1
91	N sp3	HCN(2pi)	−0.04	1	1
92	N sp3	C3	−6.73	3	3
93	N sp3	C3(pi)	−6.73	4	4
94	N sp3	C3(2pi)	−4.26	2	2
95	N sp3	C2N(pi)	−3.87	2	2
96	N sp3	C2N(2pi)	−2.95	3	3
97	N sp2	H=C	−1.73	1	1
98	N sp2	C=C	−0.94	8	6
99	N sp2	=CN	0.00	6	5
100	N sp2	O=O	1.11	1	1
101	N aromatic	:C2	−0.82	19	13
102	N aromatic	:C:N	0.14	2	1
103	N(+) sp2	CO=O(−)	−0.35	16	13
104	O	HC	−0.77	19	18
105	O	HC(pi)	−0.04	13	13
106	O	HS	2.38	2	1
107	O	C2	−2.71	31	21
108	O	C2(pi)	−1.68	34	31
109	O	C2(2pi)	−0.61	11	10
110	O	CN(pi)	0.00	1	1
111	O	CS	0.56	4	2
112	O	CP	−0.04	12	4
113	P3	O3	−0.61	1	1
114	P4	O3=O	−0.60	2	2
115	P4	O3=S	1.81	1	1
116	S2	HC	1.70	1	1
117	S2	C2	0.06	2	2
118	S2	C2(2pi)	−0.54	3	3
119	S4	C2=O	0.25	2	2
120	S4	C2=O2	0.08	2	2
121	S4	O2=O2	0.00	3	3
A	Based on				406
B	Goodness of fit	R^2^	0.995		351
C	Deviation	Average	0.35		351
D	Deviation	Standard	0.51		351
E	K-fold cv	K	10.00		308
F	Goodness of fit	Q^2^	0.9897		308
G	Deviation	Average (cv)	0.46		308
H	Deviation	Standard (cv)	0.76		308

**Figure 12:**
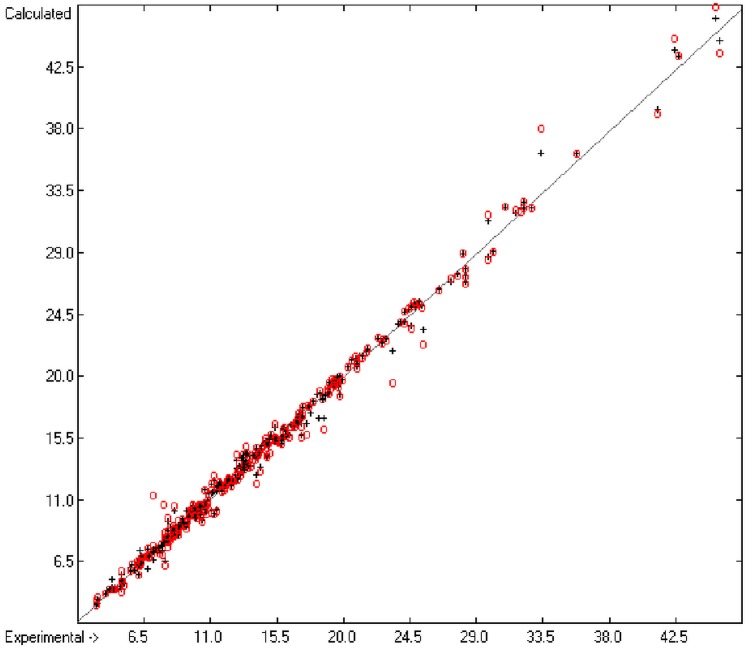
Correlation diagram of polarizability data (10-fold cross-validated: N = 308; Q^2^ = 0.9897; slope = 0.99).

**Figure 13:**
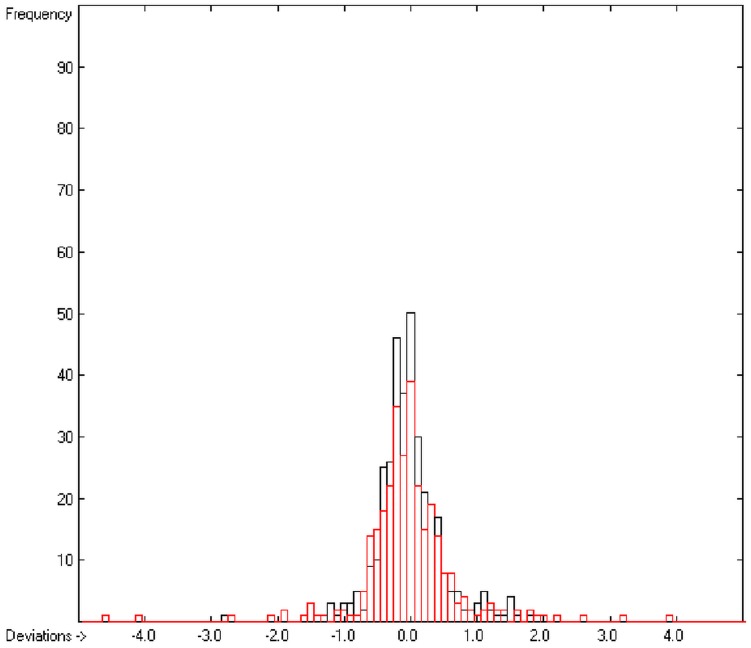
Histogram of polarizability data (S = 0.76).

It can be seen that, e.g., while Miller [10] only needed one parameter for a tetrahedral carbon (CTE in his term) the present table lists 32 different atom groups for the same type of carbon (C sp3 in this paper’s term) to cover a similar number of compounds. At this point it must be stressed again that for all the calculations of the goodness of fit and the cross validations only atom groups were considered for which the number of representative molecules (shown in the right column of the group-contribution tables) exceeds 2. Nevertheless, as the present calculation method is a simple summing up of the group contributions, the evaluation of a molecular polarizability value can in principle be done manually. The cross-validated standard deviation of 0.76 for the limited number of experimental examples is comparable to the measuring inaccuracies as discussed by Miller [10]. (Due to the relatively small set of compounds for the polarizability calculations a tentative leave-one-out cross validation calculation was carried out which resulted in a Q^2^ of 0.9901 and a standard deviation of 0.75, based on 312 molecules.) These deviations are also reflected in the dispersion of the data about the regression line in Figure 12 and the relatively wide Gaussian bell form in Figure 13. Nevertheless, the excellent correlation coefficients R^2^ and Q^2^ of the cross validation prove that the feasibility of the group-additivity method. The deviations do not correlate with the size of the molecules and, thus, the polarizabilities, however, there is evidence (see Figure 12) that the polycyclic aromatic and heteroaromatic compounds exhibit generally poorer accordance with experiment, an observation which is also reflected in Miller’s results. A reduction of this drift might be achieved if more experimental data for large conjugated molecules were available.

### 3.8. Aqueous Toxicity

**Figure 14:**
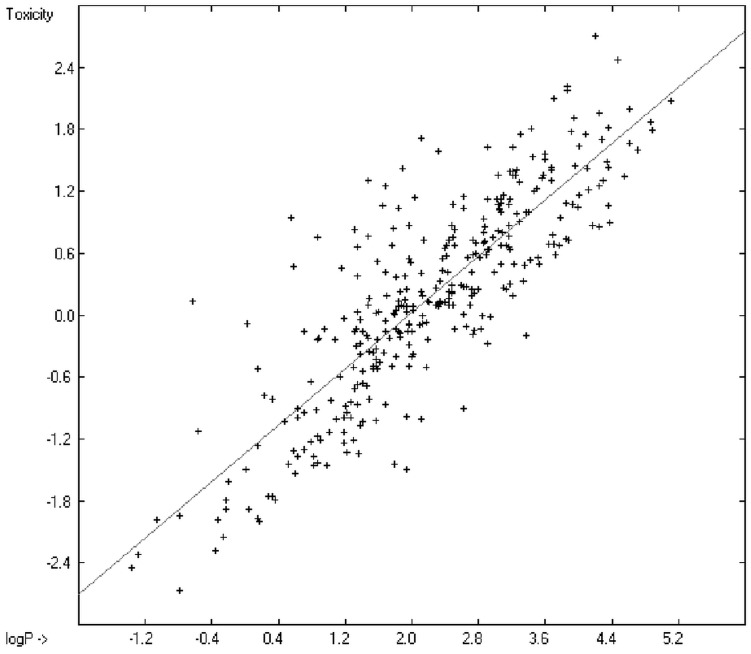
Correlation diagram of logP against toxicity (N = 335, R^2^ = 0.7043).

**Figure 15:**
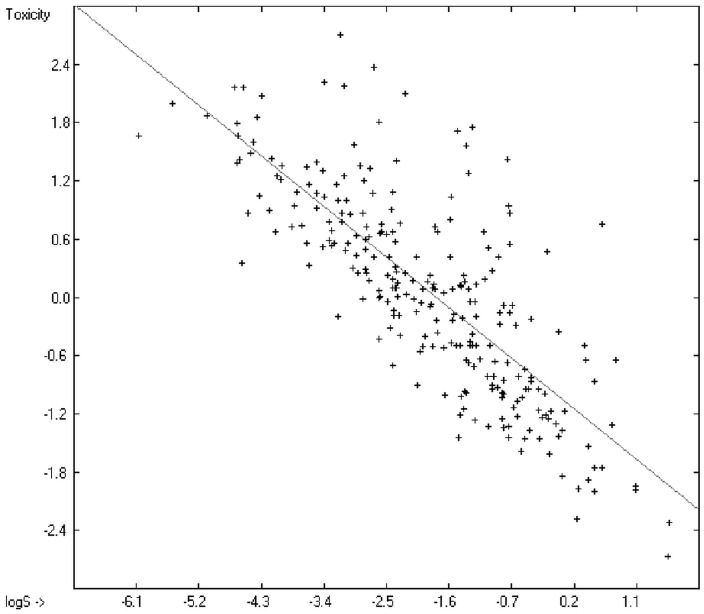
Correlation diagram of logS against toxicity (N = 253, R^2^ = 0.6186).

The most commonly used method due to its reliability and robustness for measuring aqueous toxicity is the growth inhibition of the protozoan cilate *Tetrahymena pyriformis*, defined as pIGC_50_, where IGC_50_ expresses the aqueous concentration of a molecule in mmoL/L causing a 50% growth inhibition under static conditions. Reviewing the many efforts mentioned in the introductory chapter to find reasonable physical or physico-chemical descriptors for the prediction of a molecule’s aqueous toxicity, the most evident ones are those which depend on the aqueous solubility, *i.e.*, logP_O/W_ and the molecule’s solubility itself. Ellison *et al.* [24] presented a plot of experimental toxicity data of 87 saturated alcohols and ketones against their logP (40 logP values of which were calculated), showing for this limited group a correlation coefficient of 0.96. An analogous plot, but on a much larger data basis, where both experimental logP and toxicity data are known, is shown in Figure 14. All the experimental toxicity data were made available in the publication of Ellison *et al.* [24], while logP and logS data originate from the same sources as in the previous chapters D and E. The linear regression equation pIGC_50_ = 0.68 × logP − 1.34 in Figure 14 corresponds well with Ellison’s regression formula pIGC_50_ = 0.78 × logP − 2.01. A direct but inverse correlation between the toxicity and the solubility of molecules is given in Figure 15, with a—rather more indicative—correlation coefficient of 0.6186 and a linear regression equation pIGC_50_ = −0.58 × logP − 1.03.

**Figure 16:**
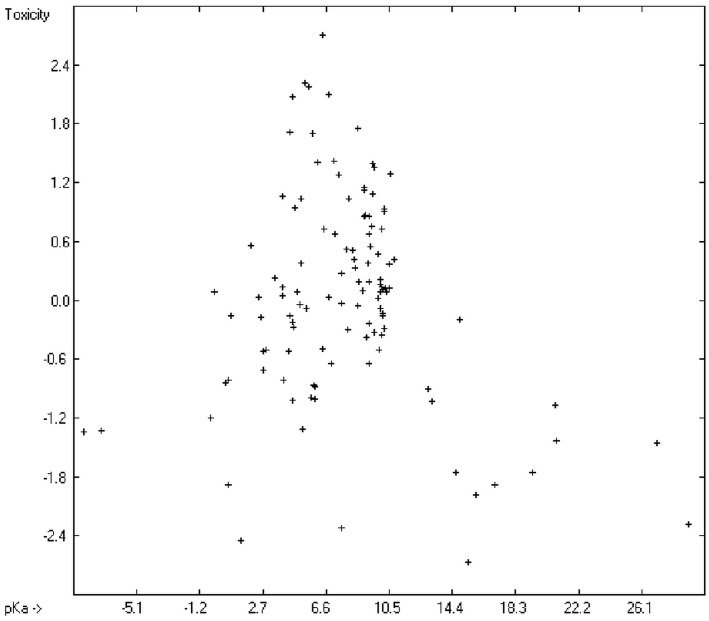
Correlation diagram of pKa against toxicity (N = 112, R^2^ = 0.0282).

Michałowicz and Duda [86], on the other hand, also ascribed the noxious effect of variously substituted phenols to their dissociation constant pKa. This assumption, however, could not be confirmed in this study as Figure 16 illustrates where the experimental pKa values of 115 compounds, extracted from the Handbook of Chemistry and Physics [87], are put in relation to their experimental toxicity data and evidently exhibit no correlation at all.

Regarding the promising correlation of the experimental logP and solubility with the toxicity data and the fact that both the former are very successfully predictable by means of the well-established group-additivity method it was obvious to try this method for the direct prediction of the toxicity of molecules without the detour via other descriptors. Table 15 shows the result of this attempt. The goodness of fit Q^2^ of 0.8404 for 810 cross-validated molecules is clearly better than the correlation coefficient R^2^ for the logP *vs.* toxicity correlation and the cross-validated standard deviation S of 0.42 is well within the experimental error range of about 0.5 as was assumed by Ellison *et al.* [24]. Taking this standard deviation as a benchmark then 78.5% of the experimental values are correctly predicted for those 836 molecules for which the conditions for the group-additivity calculation based on Table 15 are fulfilled and only for 3.6% the predicted exceed the experimental values by more than twice this deviation as can be seen in the enclosed table in the supplementary material named “Experimental vs Calculated Toxicity Data Table.doc”. The associated list of compounds is available at the same location as SD file named “Compounds List for Toxicity Calculations.sdf”.

**Table 15:** Atom group Contributions for Toxicity Calculations.

Nr	Atom Type	Neighbours	Contribution	Occurrences	Molecules
1	Const		−1.66	859	859
2	C sp3	H3C	0.24	772	469
3	C sp3	H3N	0.13	12	5
4	C sp3	H3O	0.49	72	67
5	C sp3	H3S	0.31	5	3
6	C sp3	H2C2	0.34	986	313
7	C sp3	H2CN	0.08	10	7
8	C sp3	H2CN(+)	0.55	4	4
9	C sp3	H2CO	0.58	205	188
10	C sp3	H2CS	0.34	31	18
11	C sp3	H2CCl	0.31	13	13
12	C sp3	H2CBr	0.75	15	14
13	C sp3	H2CJ	0.86	2	2
14	C sp3	HC3	0.14	63	58
15	C sp3	HC2O	0.45	51	50
16	C sp3	HC2S	0.00	1	1
17	C sp3	HC2Cl	−0.07	1	1
18	C sp3	HC2Br	0.75	4	3
19	C sp3	HCCl2	0.35	1	1
20	C sp3	HCBr2	0.88	1	1
21	C sp3	C4	0.20	32	27
22	C sp3	C3O	0.42	23	22
23	C sp3	C3N	0.21	1	1
24	C sp3	C2O2	1.06	1	1
25	C sp3	CF3	0.82	4	4
26	C sp3	CCl3	−0.03	1	1
27	C sp2	H2=C	0.09	31	30
28	C sp2	HC=C	0.20	84	57
29	C sp2	HC=N	0.48	2	2
30	C sp2	HC=O	0.05	21	21
31	C sp2	H=CO	0.27	9	8
32	C sp2	H=CS	0.39	18	11
33	C sp2	HO=O	−0.11	7	7
34	C sp2	C2=C	0.27	11	10
35	C sp2	C2=N	0.24	4	4
36	C sp2	C2=O	−0.51	62	62
37	C sp2	C=CO	0.21	7	6
38	C sp2	C=CS	0.64	5	4
39	C sp2	C=CBr	0.75	1	1
40	C sp2	CN=O	0.28	25	25
41	C sp2	CN=S	1.23	1	1
42	C sp2	CO=O	−0.04	122	116
43	C sp2	=CO2	0.18	1	1
44	C sp2	=CSCl	0.43	1	1
45	C aromatic	H:C2	0.23	2322	569
46	C aromatic	H:C:N	0.06	44	27
47	C aromatic	C:C2	0.29	485	362
48	C aromatic	:C3	0.23	44	22
49	C aromatic	C:C:N	0.00	8	8
50	C aromatic	:C2N	1.06	60	58
51	C aromatic	:C2N(+)	1.33	135	105
52	C aromatic	:C2:N	0.67	6	4
53	C aromatic	:C2O	0.48	360	282
54	C aromatic	:C2S	0.43	9	9
55	C aromatic	:C2F	0.52	75	39
56	C aromatic	:C2Cl	0.76	209	114
57	C aromatic	:C2Br	0.80	69	50
58	C aromatic	:C2J	1.18	13	11
59	C aromatic	:C:NF	0.02	5	3
60	C aromatic	:C:NCl	0.47	2	2
61	C aromatic	:C:NBr	0.67	1	1
62	C sp	H#C	0.09	8	8
63	C sp	C#C	0.17	14	11
64	C sp	C#N	−0.33	43	41
65	C sp	=N=S	0.87	1	1
66	N sp3	H2C	−0.61	3	3
67	N sp3	H2C(pi)	−0.90	66	65
68	N sp3	H2N	−0.05	1	1
69	N sp3	HC2(pi)	−0.93	5	5
70	N sp3	HC2(2pi)	−1.71	4	4
71	N sp3	HCN(pi)	0.00	1	1
72	N sp3	HCO(pi)	0.08	1	1
73	N sp3	C3	−0.54	3	1
74	N sp3	C3(pi)	−1.04	3	3
75	N sp2	C=C	0.00	1	1
76	N sp2	=CO	−0.43	6	6
77	N sp2	C=O	−0.07	1	1
78	N(+) sp2	CO=O(−)	−0.50	139	109
79	N aromatic	:C2	−0.29	33	30
80	O	HC	−1.05	163	149
81	O	HC(pi)	−0.07	295	254
82	O	C2	−1.12	4	3
83	O	C2(pi)	−0.60	182	165
84	O	HN	0.07	1	1
85	O	HN(pi)	0.01	6	6
86	O	C2(2pi)	−0.30	15	15
87	S2	HC	0.11	6	4
88	S2	C2	0.02	6	5
89	S2	C2(pi)	−0.10	6	6
90	S2	C2(2pi)	−0.15	13	11
91	S4	C2=O	−1.32	3	3
92	S4	C2=O2	−1.22	4	4
A	Based on				859
B	Goodness of fit	R^2^	0.8665		836
C	Deviation	Average	0.29		836
D	Deviation	Standard	0.39		836
E	K-fold cv	K	10.00		810
F	Goodness of fit	Q^2^	0.8404		810
G	Deviation	Average (cv)	0.31		810
H	Deviation	Standard (cv)	0.42		810

A comparison of these results with published data is difficult as the latter are either based on only a limited set of structures, on a small basis of compounds or on an entirely different approach. Nevertheless, a few numbers should provide an idea as to how classify the present result: Schultz [21] calculated an equation for the toxicity based on logP and the superdelocalizability of 197 benzene derivatives yielding in a correlation coefficient R^2^ of 0.816 and a standard deviation S of 0.34. Melagraki *et al.* [23] trained an RBF neural network to yield an equation for the toxicity calculation founded on the logP, pKa, E_LUMO_, E_HOMO_ and N_hdon_ values of 180 phenols with an R^2^ of 0.6022 and a root mean square of 0.5352. Duchowicz *et al.* [22] published the results of the QSAR calculations of 200 phenol derivatives to give a seven-parameters equation with a R^2^ of 0.7242 (R = 0.851) and an S of 0.442. Finally, Ellison *et al.* [24], who only derived a compound’s toxicity from its logP value found an equation for 87 saturated alcohols and ketones which yielded an R^2^ of 0.96 and an S of 0.20.

Tentatively, a validation test was carried out applying the leave-one-out method yielding a Q^2^ of 0.8409 and a standard deviation of again 0.42, based on 816 molecules. A tentative extention of the atom groups in Table 15 by the “pseudo atom” types as used in Table 8 for the calculation of logP (*i.e.*, “H”, “Alkane”, “Unsaturated HC” and “X(CH_2_)*_n_*”)—combined or one by one—interestingly either had no effect or even led to a deterioration of the goodness of fit.

**Figure 17:**
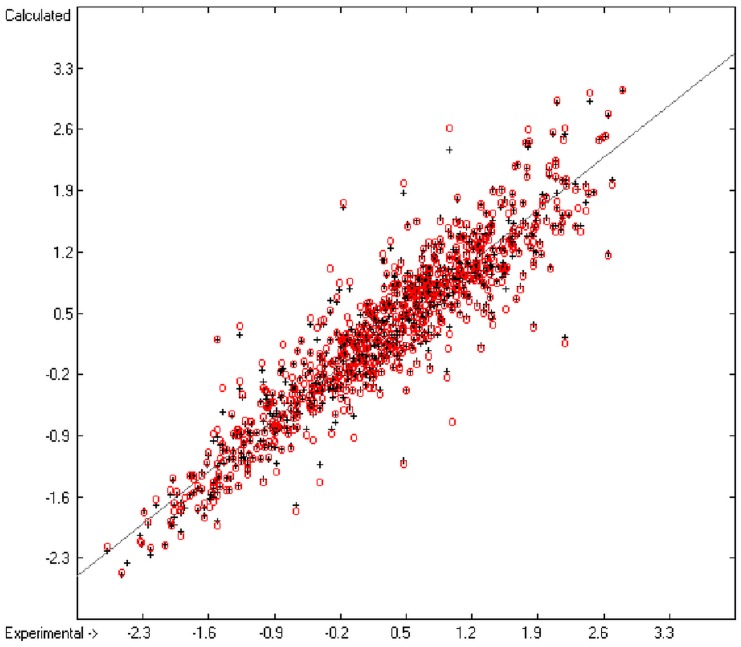
Correlation diagram of toxicity data (10-fold cross-validated: N = 810, Q^2^ = 0.8404, slope = 0.85).

**Figure 18:**
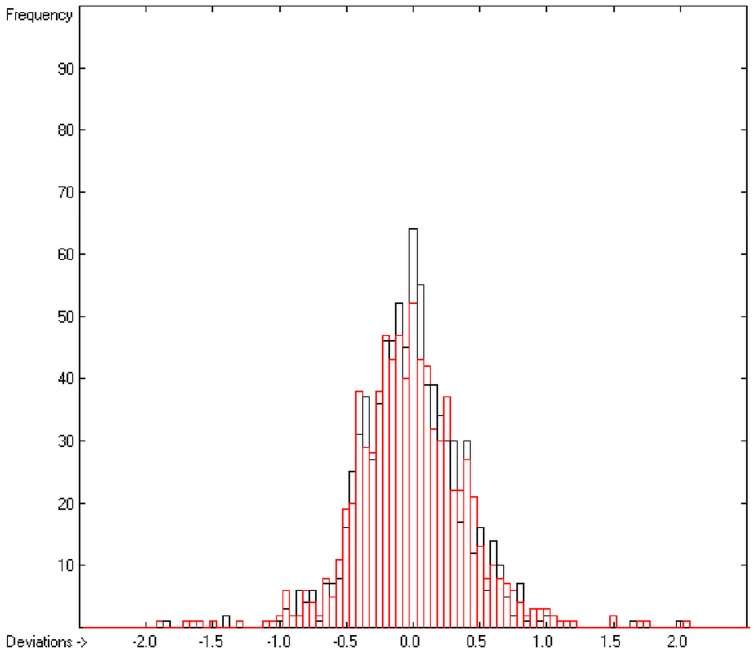
Histogram of toxicity data (S = 0.42).

Figure 17 and Figure 18 illustrate the correlation diagram and histogram of the toxicity calculations. The slope of 0.85 in Figure 17, calculated from the training set, reflects the slightly lower correlation between experimental and predicted values. (An analogous calculation of the slope using the cross-validated data yielded a slope of 0.84.).

### 3.9. Blood-Brain Barrier

**Figure 19:**
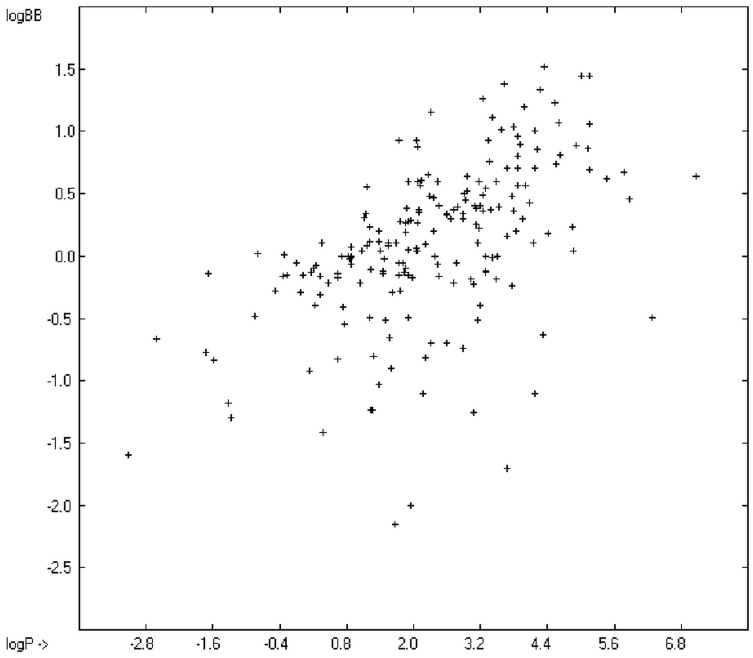
Correlation diagram of logP against logBB (N = 198, R^2^ = 0.2815).

**Figure 20:**
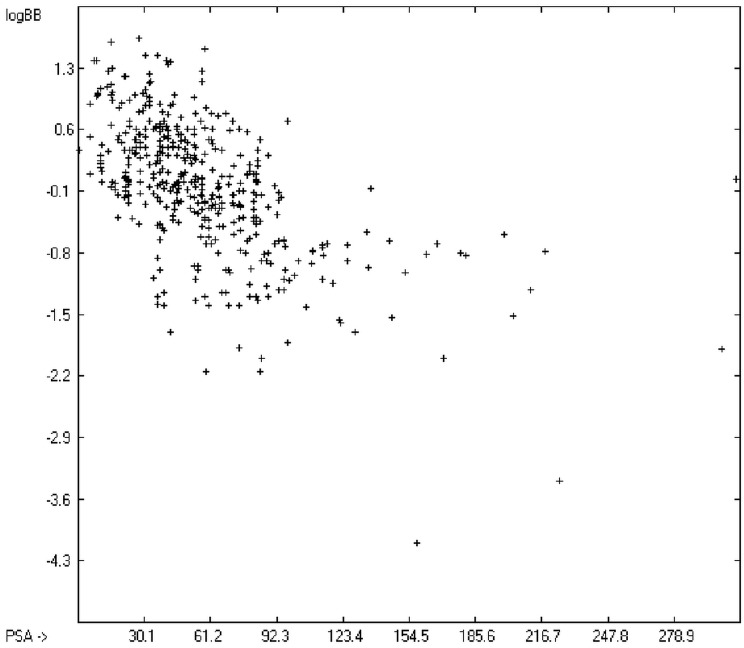
Correlation diagram of polar surface area (PSA) against logBB (N = 438, R^2^ = 0.3335).

**Figure 21:**
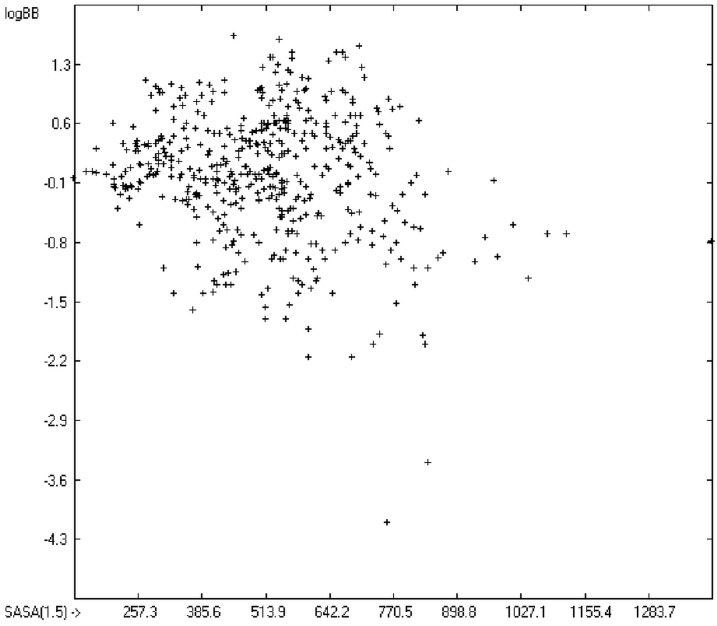
Correlation diagram of solvent-accessible surface area (SASA) against logBB (N = 493, R^2^ = 0.0334).

**Figure 22:**
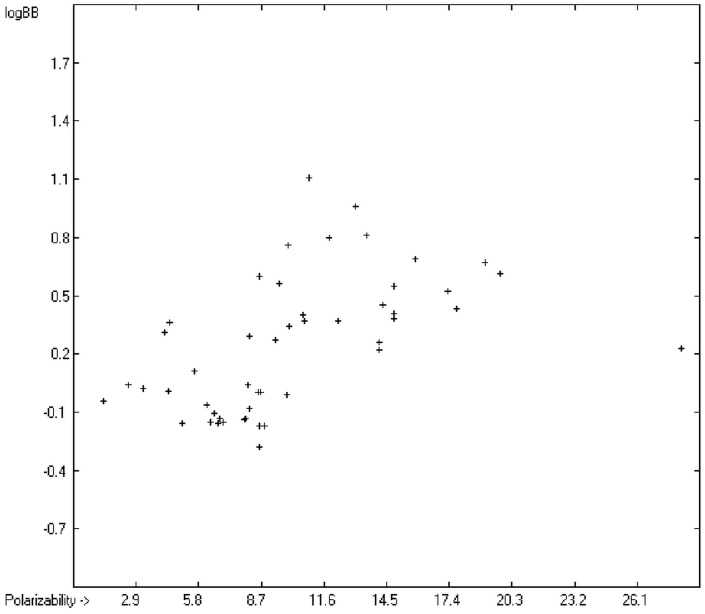
Correlation diagram of molecular polarizability against logBB (N = 49, R^2^ = 0.2717).

The blood-brain barrier is literally a “hard nut” to crack, not only for the molecules which are supposed to penetrate it but also for the theoretician who tries to find a reliable tool for the prediction of their potential to enter the brain tissue as is evident upon reviewing the many attempts to define suitable molecular descriptors to start with described in the introductory chapter. Interestingly, some of the most commonly applied and seemingly logical descriptors such as logP_O/W_, polar surface area (PSA), solvent-accessible surface area (SASA) or molecular polarizabilty exhibit no correlation to speak of with the blood-brain distribution ratio logBB, as has already been stated by Lanevskij *et al.* [39] for logP_O/W_ and as is shown in Figure 19, Figure 20, Figure 21 and Figure 22.

The experimental logBB data are collected from the references [27,28,29,30,31,32,33,34,35,36,37,38,39,40], logP data originate from the same sources as in chapter D, PSA and SASA values are calculated internally using an approximation function (see Appendix), and experimental polarizabilty data are taken from the Handbook of Chemistry and Physics [85] and Miller’s [10] publication.

It therefore seemed reasonable to abstain from any attempt to base logBB-prediction calculations on other etablished molecular descriptors and proceed with the group-additivity method as described earlier, which is very similar to H. Sun’s [12] method. While Sun applied his three-component model on only 57 compounds, yielding a correlation coefficient R^2^ of 0.897, a 7-fold cross-validated Q^2^ of 0.504 and root-mean square error of 0.259, the present calculation extended over 487 molecules and resulted in a goodness of fit R^2^ of 0.6991 for the evaluable training set of 413 molecules, and yielded a 10-fold cross-validated Q^2^ of 0.4786 and a deviation of 0.52 for the test set of 385 molecules. The large difference between R^2^ and Q^2^ is ominous and indicates the limits of the present group-additivity method. A leave-one-out cross-validation calculation produced a marginally better Q^2^ of 0.4825 but left the standard deviation unchanged. Since in general, as Sun [12] stated in his paper, a value of Q^2^ below 0.5 is regarded as at best statistically meaningful but no longer representative for a good model, the complete list of 176 atom groups and their contribution has been omitted from Table 16 presented below. It therefore only lists the result of the least-squares and 10-fold cross-validation calculations. The complete list is available in the supplementary material under the name of “LogBB Parameters Table.doc”. The associated list of results is viewable at the same location under the name of “Experimental vs Calculated LogBB Data Table.doc” and the corresponding list of compounds as SD file with the name of “Compounds List for LogBB Calculations.sdf”.

**Table 16:** Results of the logBB Calculations.

Nr	Atom Type	Neighbours	Contribution	Occurrences	Molecules
1	Const		0.21	486	486
2	C sp3	H3C	0.06	519	255
...	...	...	...	...	...
A	Based on				486
B	Goodness of fit	R^2^	0.6991		413
C	Deviation	Average	0.30		413
D	Deviation	Standard	0.39		413
E	K-fold cv	K	10.00		385
F	Goodness of fit	Q^2^	0.4786		385
G	Deviation	Average (cv)	0.40		385
H	Deviation	Standard (cv)	0.52		385

**Figure 23:**
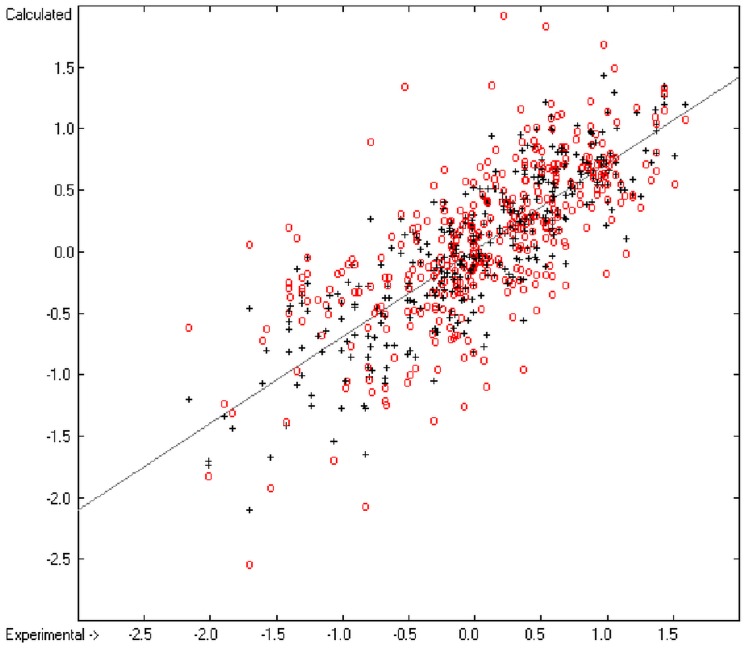
Correlation diagram of logBB data (10-fold cross-validated: N = 385; Q^2^ = 0.4786; slope = 0.70).

**Figure 24:**
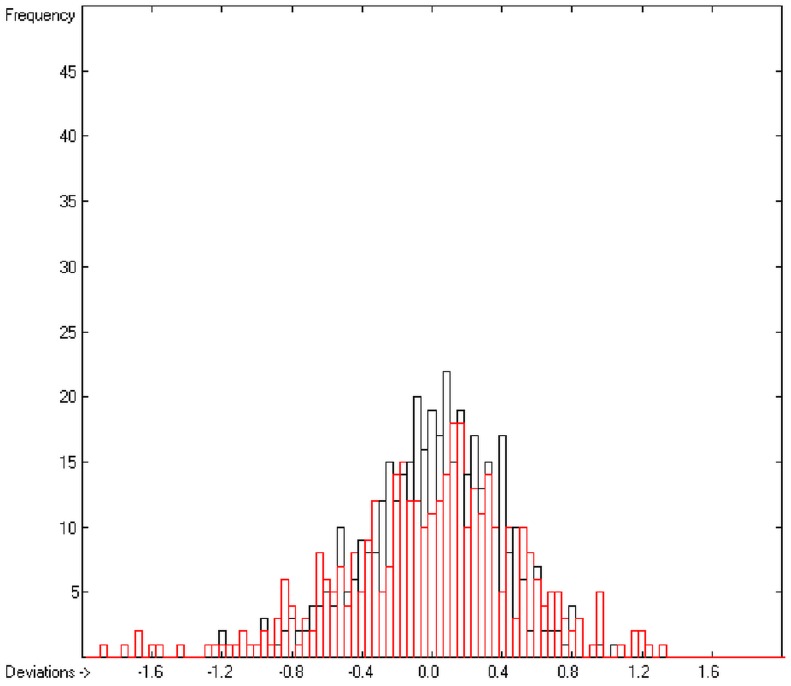
Histogram of logBB data (S = 0.53).

Figure 23 illustrates the large dispersion of the training and particularly the cross-validated data about the regression line which exhibits a slope of 0.70. The distribution of the deviations, shown in the histogram (Figure 24), nearly extends over the complete experimental values range of between −2.15 and +1.6. In conclusion, it is obvious to see that the present group-additivity model is too inaccurate for the prediction of logBB for an unlimited scope of molecular structures. On the other hand, reviewing the many publications which base their predictions either on too few examples or on models that are at best useful for only a very limited structural diversity or even rest on inappropriate parameters visualized above, it follows that a universal approach for the prediction of logBB for the complete spectrum of medicinal chemistry is still outstanding.

## 4. Conclusions

A generally applicable computer algorithm based on the well-established group-additivity method has been presented and has been applied for the calculation of the seven molecular descriptors heat of combustion, logP, logS, molar refractivity, molecular polarizability, aqueous toxicity and logBB. An eighth descriptor, the heat of formation, was calculated indirectly using the calculated value of the heat of formation. The definition of the atom groups has been set up in a way that allowed a straightforward program code of the computer algorithm except for the special groups for which, however, code development could take advantage of the information of the 3D-molecular structures stored in the molecules database. The complete algorithm, realized in ChemBrain IXL, thus enables the computation of the contributions of all the atom groups as well as all the described special groups for descriptor evaluations; their inclusion, however, is governed by their presence or absence in the respective parameters tables. Within this context it is worth mentioning that for the prediction of the refractivity, molecular polarizability and toxicity in principle a 3D geometry is not required.

The present group-additivity algorithm has shown its versatility in that it is capable of producing results at once that are in good to excellent agreement with experimental data for six of the seven title descriptors. The present study has also shown the limits of the group-additivity method as such in an area where too many unknown or incalculable factors influence the experimental data as has been exemplified for logBB.

The number of molecules in the database—at present about 20,700—which encompasses a representative collection of organic and metal-organic compounds of commercial as well as scientific relevance and which has all the referenced data stored, and the amount of compounds for which the title descriptors could be evaluated under the given constraints provides an accountable estimate of the scope of applicability of each of the presented tables of group contributions. For the heat of combustion and formation it is *ca*. 75%, for logP *ca*. 84%, for logS *ca*. 73%, for the molecular polarizability *ca*. 42%, for the refractivity *ca*. 75% and for the toxicity *ca*. 41%. These percentage numbers evidently reflect the number of experimental data available at present. There is no doubt, however, that even with a larger database of compounds for the calculation of the group contributions there is a limit to the improvement of the accuracy of the predictions on the basis of this method, not only because there is little hope that the existing experimental databases and their deficiencies will be re-examined in the laboratories but also because of influences on the results that can principally not be dealt with by this method, as there are non-neighbouring effects (e.g., gauche or cis), intramolecular charge effects or non-bonded interactions.

In view of these facts there is truth in the words which Cohen and Benson [10] stated in their closing remarks saying that the atom group additivity method is “a useful tool for making rapid property estimates or for checking the likely reliability of existing measurements”.

## Supplementary Materials

Supplementary materials can be accessed at: http://www.mdpi.com/1420-3049/20/10/18279/s1.

## Appendix

The database of ChemBrain IXL has the compounds stored as three-dimensional structures. Therefore each new input undergoes optimization of its three-dimensional form before being stored. New compounds can be added to the database either by importing them from external standard MOL or SD files or manually on a self-explanatory, easy-to-use “three-dimensional” drawing board. 3D-structure optimization is carried out within ChemBrain IXL by means of a force-field method called “steepest descent” [88], where the search direction to the energy minimum is given at each current atomic position by the first derivative of Equation (A1).

(A1) $$E=\sum E_{str}+\sum E_{ang}+\sum E_{tor}+\sum E_{vdw}+\sum E_{oop}$$

This function denotes the sum of all bond stretching (*E_str_*), bond angle (*E_ang_*), torsional angle (*E_tor_*), Van-der-Waals interaction (*E_vdw_*) and out-of-plane bending (*E_oop_*) energies. Optionally, the algorithm scans the hyper-surface for the compound’s global energy minimum.

At present, optionally, besides seven of the eight descriptors presented here four more descriptors of a compound, which can be derived directly from its structure, are calculated immediately after addition and completion of the 3D-structure optimization and then entered into the molecule’s descriptors list: molecular volume, molecular surface, polar surface area (PSA) and solvent-accessible surface area (SASA). The molecular volume is defined by the Van-der-Waals radii of the atoms and its value is approximated numerically by scanning a small but defined cube through the entire spacial box defined by the total width, length and height of the molecule and adding up those cubes which lie inside the range of any atom’s VdW radius. For fhe calculation of the molecular surface the approximation Equation (A2) is used, where A is the total molecular surface, *r*_j_ is the corresponding radius of atom *j*, *N*_j_ is the number of points evenly distributed on atom *j*’s sphere and *n*_j_ is the number of those points which are not occluded by the spheres of other atoms.

(A2) $$A=4\text{π}\sum_{j}r_{j}^{2}\frac{n_{j}}{N_{j}}$$

The calculation of SASA is based on the same function but assumes an extended radius for each atom accounting for the radius of the surrounding solvent molecules, which by default is taken as 1.5 Angstroms, approximately the value of water. For the calculation of PSA again the same function is used but the sum is limited to the VdW surfaces of the polar atoms oxygen, nitrogen, sulfur, phosphorus and hydrogen attached to the former atoms as suggested by Ertl *et al.* [89].

The present work is part of a project called ChemBrain IXL available from Neuronix Software (www.neuronix.ch, Rudolf Naef, Lupsingen, , Switzerland).
