# Supplementary material for: A Generally Applicable Computer Algorithm Based on the Group Additivity Method for the Calculation of Seven Molecular Descriptors: Heat of Combustion, LogPO/W, LogS, Refractivity, Polarizability, Toxicity and LogBB of Organic Compounds; Scope and Limits of Applicability
Source: Molecules. 2015 Oct 7;20(10):18279–351. doi: 10.3390/molecules201018279 (PMC6332030; doi:10.3390/molecules201018279)
Supplement: Supplementary file 1 [file molecules-20-18279-s001.zip › Experimental vs Calculated Heat-of-Combustion Data Table.doc]

**Experimental vs. Calculated Heat-of-Combustion Data (in kJ/mol), sorted by absolute Deviation**

| **No.** | **Molecule name** | **deltaH(c)** | **deltaH(c) calc** | **deltaH(c) test** | **Deviation** |
| --- | --- | --- | --- | --- | --- |
|  |  |  |  |  |  |
| 1 | 3-Phenylamino-2-naphthoic acid | -8291.50 | -8291.50 | -8289.50 | 0.00 |
| 2 | Butyric acid | -2185.00 | -2185.00 | -2184.20 | 0.00 |
| 3 | N-Acetamido-N'-nitroguanidine | -1988.70 | -1988.70 |  | 0.00 |
| 4 | N-Formamido-N'-nitroguanidine | -1355.70 | -1355.70 |  | 0.00 |
| 5 | Tratramide | -1783.60 | -1783.60 | -1780.40 | 0.00 |
| 6 | Caffeine | -4261.30 | -4261.30 | -4264.60 | 0.00 |
| 7 | 5-Amino-1-phenyltetrazole | -4068.70 | -4068.70 | -4068.50 | 0.00 |
| 8 | 1,3,5-Trimethylbenzene | -5193.10 | -5193.10 | -5192.00 | 0.00 |
| 9 | Diphenyldisulfide | -7509.90 | -7509.90 |  | 0.00 |
| 10 | Ethyl sulfate | -1360.69 | -1360.70 |  | 0.01 |
| 11 | 2-Methyl-1,3-pentanediol | -3787.28 | -3787.30 | -3787.30 | 0.02 |
| 12 | Dimethyldimethoxysilane | -3423.58 | -3423.60 |  | 0.02 |
| 13 | 2-n-Propylthiophene | -4779.94 | -4779.90 |  | -0.04 |
| 14 | 4-Methyl-2,6,7-trioxabicyclo[2.2.2]octane | -3218.54 | -3218.50 | -3217.90 | -0.04 |
| 15 | Guanidinium chloride | -949.26 | -949.30 |  | 0.04 |
| 16 | 2,3,4-Trimethylpentane | -5469.36 | -5469.40 | -5470.20 | 0.04 |
| 17 | 2-Methyl-1-octene | -5953.05 | -5953.10 | -5953.80 | 0.05 |
| 18 | 2,3-Dimethylpentane | -4811.49 | -4811.40 | -4810.50 | -0.09 |
| 19 | n-Butyl methyl ether | -3394.39 | -3394.30 | -3394.30 | -0.09 |
| 20 | 1,2,3,5-Tetraethylbenzene | -8462.69 | -8462.60 | -8465.50 | -0.09 |
| 21 | p-Tolualdehyde | -4173.71 | -4173.80 | -4172.60 | 0.09 |
| 22 | 2-Chlorobenzoic acid | -3087.70 | -3087.60 | -3088.80 | -0.10 |
| 23 | 2-Chloro-1,1-difluoroethylene | -971.40 | -971.30 |  | -0.10 |
| 24 | Methyl-n-propylketone | -3101.50 | -3101.40 | -3101.90 | -0.10 |
| 25 | Dimethyl glutarate | -3610.30 | -3610.40 | -3610.20 | 0.10 |
| 26 | n-Butylethylether | -4036.08 | -4036.20 | -4035.50 | 0.12 |
| 27 | 1-Heptanethiol | -5446.46 | -5446.60 | -5446.60 | 0.14 |
| 28 | n-Butylformate | -2929.77 | -2929.60 | -2932.30 | -0.17 |
| 29 | 3-Hydroxy-2-methylpropionaldehyde | -2291.31 | -2291.50 | -2291.00 | 0.19 |
| 30 | Cyclobutanonedimethylacetal | -3733.71 | -3733.90 | -3733.00 | 0.19 |
| 31 | Diethylsulfoxide | -3337.50 | -3337.30 | -3338.70 | -0.20 |
| 32 | Norleucine | -3582.20 | -3582.00 | -3580.40 | -0.20 |
| 33 | 2,2-Dimethoxybutane | -3879.68 | -3879.90 | -3879.10 | 0.22 |
| 34 | Di-n-propylether | -4035.96 | -4036.20 | -4037.10 | 0.24 |
| 35 | Tetrahydro-2H-pyran-2-one | -2651.65 | -2651.90 | -2651.70 | 0.25 |
| 36 | 1,1-Dichlorotetrafluoroethane | -567.03 | -567.30 | -565.10 | 0.27 |
| 37 | 1,3,5-Tri-t-butylbenzene | -11058.23 | -11058.50 | -11060.70 | 0.27 |
| 38 | Brucine | -12279.00 | -12278.70 | -12283.70 | -0.30 |
| 39 | 8-Methylquinoline | -5324.60 | -5324.30 | -5323.40 | -0.30 |
| 40 | m-Anthranilic acid | -3346.50 | -3346.80 | -3348.00 | 0.30 |
| 41 | Glyceryl-2-caprate | -7740.80 | -7741.10 | -7741.60 | 0.30 |
| 42 | Methyl isoamyl ketone | -4407.63 | -4407.30 | -4408.20 | -0.33 |
| 43 | Carbanilide | -6718.37 | -6718.70 | -6721.90 | 0.33 |
| 44 | Hemimellitene | -5201.55 | -5201.90 | -5201.60 | 0.35 |
| 45 | 1,4-Pentadiene | -3193.28 | -3192.90 | -3195.30 | -0.38 |
| 46 | Ethynylbenzene | -4291.72 | -4292.10 | -4292.90 | 0.38 |
| 47 | Di-n-propylsulfite | -4317.30 | -4316.90 |  | -0.40 |
| 48 | Phenaceturic acid | -4878.50 | -4878.10 | -4879.10 | -0.40 |
| 49 | 2,6-Dinitrophenol | -2723.10 | -2722.70 | -2723.10 | -0.40 |
| 50 | N-Phenylglycine | -4037.60 | -4038.00 | -4038.80 | 0.40 |
| 51 | 1,5-Dinitronaphthalene | -4834.20 | -4834.60 | -4828.70 | 0.40 |
| 52 | 2,5-Dimethylhexane | -5464.00 | -5464.40 | -5462.50 | 0.40 |
| 53 | Di-n-butylsulfide | -6102.00 | -6102.40 | -6103.30 | 0.40 |
| 54 | 2-Ethyl-1,4-dimethylbenzene | -5855.07 | -5854.60 | -5854.30 | -0.47 |
| 55 | trans-2-Butene-1,4-diol | -2317.58 | -2317.10 | -2316.50 | -0.48 |
| 56 | Serine | -1441.90 | -1441.40 | -1445.80 | -0.50 |
| 57 | 2-Nitroacetanilide | -4077.50 | -4077.00 | -4078.30 | -0.50 |
| 58 | Trimethoxymethane | -2436.30 | -2436.80 | -2437.00 | 0.50 |
| 59 | n-Butylbenzoate | -5904.59 | -5905.10 | -5905.30 | 0.51 |
| 60 | Azobutane | -5684.27 | -5684.80 | -5686.90 | 0.53 |
| 61 | 3-Chloro-1-propanol | -1861.27 | -1861.80 | -1861.20 | 0.53 |
| 62 | 2-Ethyl-2-hexenal | -4907.73 | -4907.20 | -4907.00 | -0.53 |
| 63 | 1,2-Dihydronaphthalene | -5439.33 | -5439.90 | -5437.10 | 0.57 |
| 64 | Hippurylglycine | -5216.40 | -5215.80 | -5213.90 | -0.60 |
| 65 | Diphenylsulfoxide | -6763.40 | -6764.00 | -6763.60 | 0.60 |
| 66 | Trimethylacetic acid | -2832.10 | -2831.50 | -2830.90 | -0.60 |
| 67 | Phenylalanine-N-carboxylic anhydride | -4670.80 | -4670.20 | -4670.40 | -0.60 |
| 68 | 5-Aminovaleric acid | -2937.00 | -2937.60 | -2935.50 | 0.60 |
| 69 | 3-Ethyl-2-methyl-1-pentene | -5301.99 | -5302.60 | -5304.10 | 0.61 |
| 70 | 4-t-Octylphenol | -8266.61 | -8266.00 | -8266.60 | -0.61 |
| 71 | 1,1-Dimethoxy-2-phenylcyclopropane | -6103.95 | -6104.60 | -6104.90 | 0.65 |
| 72 | 4-Ethyl-1,2-dimethylbenzene | -5853.86 | -5853.20 | -5853.50 | -0.66 |
| 73 | Chloroacetic acid ethylester | -2089.44 | -2090.10 | -2090.30 | 0.66 |
| 74 | Ethyl lactate | -2703.89 | -2703.20 | -2701.30 | -0.69 |
| 75 | Butyl propanoate | -4200.30 | -4199.60 | -4199.20 | -0.70 |
| 76 | Benzophenone | -6515.50 | -6514.80 | -6515.20 | -0.70 |
| 77 | Leucine | -3582.30 | -3583.00 | -3578.20 | 0.70 |
| 78 | 2-Methylhexane | -4811.40 | -4810.70 | -4810.80 | -0.70 |
| 79 | 2-Nitrobenzoic acid | -3071.00 | -3071.70 | -3072.10 | 0.70 |
| 80 | Indigo | -7594.90 | -7595.60 | -7602.00 | 0.70 |
| 81 | 4-Methoxypyridine | -3288.11 | -3287.40 | -3288.00 | -0.71 |
| 82 | 2,4-Dimethyl-1-pentene | -4641.58 | -4642.30 | -4641.00 | 0.72 |
| 83 | 3-Methylbutanoic acid | -2837.37 | -2838.10 | -2838.70 | 0.73 |
| 84 | 1,4-Bis(hydroxymethyl)-cyclohexane | -4853.75 | -4853.00 | -4851.60 | -0.75 |
| 85 | 5-Methylnonane | -6773.96 | -6773.20 | -6773.20 | -0.76 |
| 86 | s-Butylacrylate | -4035.74 | -4036.50 | -4036.60 | 0.76 |
| 87 | 1,2,3,4-Tetramethylbenzene | -5849.67 | -5848.90 | -5850.00 | -0.77 |
| 88 | o-Xylene | -4552.60 | -4551.80 | -4552.40 | -0.80 |
| 89 | 2,4,4-Trimethyl-1-pentene | -5292.20 | -5293.00 | -5291.60 | 0.80 |
| 90 | 2-Methyl-1-propanol | -2670.90 | -2671.70 | -2671.10 | 0.80 |
| 91 | 3,4-Lutidine | -4061.80 | -4062.60 | -4063.90 | 0.80 |
| 92 | Ethyl propionate | -2893.80 | -2894.60 | -2894.60 | 0.80 |
| 93 | DABCO | -4104.30 | -4105.10 | -4103.40 | 0.80 |
| 94 | 1,3,5-Trithiane | -3926.53 | -3925.70 |  | -0.83 |
| 95 | 2-Methylbutyraldehyde | -3127.47 | -3128.30 | -3129.10 | 0.83 |
| 96 | Allyl-t-butylsulfide | -5269.94 | -5269.10 | -5268.50 | -0.84 |
| 97 | 2-Pentenoic acid | -2666.55 | -2665.70 | -2665.70 | -0.85 |
| 98 | 2-Ethyl-1-butanol | -3982.37 | -3981.50 | -3983.20 | -0.87 |
| 99 | Ethyl ether | -2732.10 | -2731.20 | -2731.80 | -0.90 |
| 100 | Diethylketone | -3102.30 | -3101.40 | -3099.90 | -0.90 |
| 101 | Allylethylsulfone | -3595.35 | -3594.40 | -3596.00 | -0.95 |
| 102 | Cyclohexylacetate | -4598.85 | -4599.80 | -4600.90 | 0.95 |
| 103 | 2,4-Dimethyl-3-pentanone | -4405.64 | -4406.60 | -4407.70 | 0.96 |
| 104 | Progesterone | -12008.00 | -12009.00 | -12011.50 | 1.00 |
| 105 | 1-Phenyltetrazole | -3976.60 | -3977.60 | -3986.40 | 1.00 |
| 106 | 1-Chloro-2-methylpropane | -2693.66 | -2694.70 | -2693.90 | 1.04 |
| 107 | Cyclopentanethiol | -3911.85 | -3910.80 | -3911.90 | -1.05 |
| 108 | 3-Pentanol | -3314.66 | -3313.60 | -3314.00 | -1.06 |
| 109 | 4-Methyl-1-hexene | -4657.06 | -4656.00 | -4656.40 | -1.06 |
| 110 | 3-Methylthiophene | -3472.80 | -3471.70 | -3469.60 | -1.10 |
| 111 | 3-Hydroxy-2-naphthalenecarboxylic acid | -4930.40 | -4931.50 | -4932.90 | 1.10 |
| 112 | 1-Decanethiol | -7405.10 | -7404.00 | -7403.40 | -1.10 |
| 113 | t-Butylbenzene | -5860.76 | -5861.90 | -5862.30 | 1.14 |
| 114 | trans-4,4-Dimethyl-2-pentene | -4636.85 | -4635.70 | -4635.50 | -1.15 |
| 115 | 1,3-Diisopropylbenzene | -7167.06 | -7165.90 | -7166.50 | -1.16 |
| 116 | Hexamethylbenzene | -7137.92 | -7139.10 | -7139.10 | 1.18 |
| 117 | o-Terphenyl | -9266.50 | -9265.30 | -9265.00 | -1.20 |
| 118 | 2-Methylpentane | -4157.00 | -4158.20 | -4158.00 | 1.20 |
| 119 | Glyceryl-2-benzoate | -4880.50 | -4879.30 | -4878.50 | -1.20 |
| 120 | 4-Aminobutyric acid | -2283.90 | -2285.10 | -2284.20 | 1.20 |
| 121 | Benzenethiol | -3887.20 | -3886.00 |  | -1.20 |
| 122 | 1-Nitropropane | -2014.00 | -2015.20 | -2014.10 | 1.20 |
| 123 | 2-Butanol | -2662.40 | -2663.60 | -2663.50 | 1.20 |
| 124 | 1-Naphthaleneacetic acid | -5792.00 | -5793.20 | -5789.90 | 1.20 |
| 125 | Phenanthrene-9,10-dione | -6502.20 | -6503.40 | -6500.00 | 1.20 |
| 126 | 2-Ethyl-1-pentene | -4648.66 | -4647.40 | -4645.50 | -1.26 |
| 127 | N-(2-Aminoethyl)ethanolamine | -3011.03 | -3012.30 | -3015.40 | 1.27 |
| 128 | Chlorobenzene | -3112.70 | -3111.40 | -3111.50 | -1.30 |
| 129 | Pentachloroethane | -862.30 | -861.00 | -865.20 | -1.30 |
| 130 | Butane-1,4-dithiol | -4104.80 | -4106.10 | -4106.00 | 1.30 |
| 131 | Pinacolone | -3750.16 | -3751.50 | -3752.50 | 1.34 |
| 132 | 2,6-Dimethylheptan-4-one | -5709.65 | -5708.30 | -5709.30 | -1.35 |
| 133 | 2-Methylhexanoic acid | -4144.66 | -4143.30 | -4142.90 | -1.36 |
| 134 | 1-Hexen-5-one | -3595.34 | -3596.70 | -3596.90 | 1.36 |
| 135 | Vinylpropionate | -2727.49 | -2726.10 | -2724.10 | -1.39 |
| 136 | 5-Ethyl-1,3-dimethylbenzene | -5852.10 | -5853.50 | -5853.70 | 1.40 |
| 137 | 2-Iodophenol | -2979.80 | -2978.40 | -2977.10 | -1.40 |
| 138 | Tyrosine | -4428.10 | -4429.50 | -4429.50 | 1.40 |
| 139 | 2-Propanol | -2007.30 | -2008.70 | -2008.10 | 1.40 |
| 140 | Dimethoxymethane | -1947.20 | -1948.60 | -1950.90 | 1.40 |
| 141 | 2,2-Dimethoxypropane | -3225.99 | -3227.40 | -3226.00 | 1.41 |
| 142 | 2,4-Dimethylpentane | -4809.98 | -4811.40 | -4811.20 | 1.42 |
| 143 | 1,2-Diethoxyethane | -3913.35 | -3914.80 | -3913.60 | 1.45 |
| 144 | 3-Methyl-1-hexene | -4657.46 | -4656.00 | -4656.90 | -1.46 |
| 145 | 2,6-Xylenol | -4342.89 | -4341.40 | -4341.10 | -1.49 |
| 146 | 2-Quinolinecarbonitrile | -5042.29 | -5040.80 | -5039.10 | -1.49 |
| 147 | Desoxyamalic acid | -5531.20 | -5529.70 | -5528.00 | -1.50 |
| 148 | Adamantane | -6031.80 | -6033.30 | -6032.90 | 1.50 |
| 149 | Cyclooctanone | -4825.60 | -4827.10 | -4827.80 | 1.50 |
| 150 | n-Butylmethylsulfone | -3751.57 | -3750.00 | -3750.70 | -1.57 |
| 151 | Methyl cellosolve | -1909.08 | -1907.50 | -1908.20 | -1.58 |
| 152 | 3-Chloroaniline | -3221.80 | -3220.20 | -3218.40 | -1.60 |
| 153 | 4-Amino-3-azido-5-ethyl-1,2,4-triazole | -3072.30 | -3070.70 | -3071.80 | -1.60 |
| 154 | Phenol | -3055.50 | -3053.90 | -3053.90 | -1.60 |
| 155 | 1,1-Dimethylindane | -6275.60 | -6277.20 | -6278.30 | 1.60 |
| 156 | 2,3-Dimethyl-1-hexene | -5302.35 | -5304.00 | -5302.60 | 1.65 |
| 157 | Allyl chloride | -1888.15 | -1886.50 | -1887.40 | -1.65 |
| 158 | Amyl acetate | -4205.56 | -4203.90 | -4205.20 | -1.66 |
| 159 | 1-Eicosene | -13136.67 | -13135.00 | -13135.50 | -1.67 |
| 160 | 3-Methyl-cis-2-pentene | -3984.29 | -3982.60 | -3982.70 | -1.69 |
| 161 | Hexachlorobenzene | -2361.00 | -2359.30 | -2359.20 | -1.70 |
| 162 | 4-Methylpent-3-en-2-one | -3569.20 | -3570.90 | -3572.50 | 1.70 |
| 163 | Propane-1,2-dithiol | -3450.77 | -3452.50 | -3452.80 | 1.73 |
| 164 | n-Crotylacetate | -3382.64 | -3380.90 | -3380.20 | -1.74 |
| 165 | 2,6-Dimethylheptane | -6118.05 | -6116.30 | -6116.30 | -1.75 |
| 166 | 1-Isopropyl-3-methylbenzene | -5861.26 | -5859.50 | -5859.70 | -1.76 |
| 167 | 2-Ethylthiophene | -4125.71 | -4127.50 |  | 1.79 |
| 168 | 2-Methylheptane | -5465.20 | -5463.40 | -5463.30 | -1.80 |
| 169 | 2-Pentanol | -3315.40 | -3313.60 | -3314.00 | -1.80 |
| 170 | 1-Nitrobutane | -2669.50 | -2667.70 | -2667.40 | -1.80 |
| 171 | (L)-Cysteine | -2263.00 | -2261.20 | -2263.90 | -1.80 |
| 172 | Thiophene | -2830.10 | -2828.30 | -2825.70 | -1.80 |
| 173 | Pentanoic acid | -2839.20 | -2837.40 | -2836.70 | -1.80 |
| 174 | 1,1,1,3-Tetrachloropropane | -1638.00 | -1636.20 | -1635.70 | -1.80 |
| 175 | 2-Methylthiophene | -3474.30 | -3472.50 |  | -1.80 |
| 176 | Isoserine | -1438.20 | -1440.00 | -1441.40 | 1.80 |
| 177 | Durene | -5837.30 | -5839.10 | -5840.70 | 1.80 |
| 178 | Cyclohexylamine | -4074.07 | -4075.90 | -4074.90 | 1.83 |
| 179 | N-Butylpiperidine | -6089.36 | -6091.20 | -6092.00 | 1.84 |
| 180 | Butyl pentanoate | -5504.85 | -5506.70 | -5508.10 | 1.85 |
| 181 | Benzyl acetate | -4605.26 | -4603.40 | -4603.40 | -1.86 |
| 182 | 1,3-Dichlorobenzene | -2959.13 | -2961.00 | -2959.80 | 1.87 |
| 183 | Dimethylacetamide | -2583.89 | -2582.00 | -2576.00 | -1.89 |
| 184 | 3-Iodotoluene | -3834.20 | -3832.30 | -3831.60 | -1.90 |
| 185 | Ethanol | -1367.70 | -1365.80 | -1366.10 | -1.90 |
| 186 | 2-Nitroaniline | -3206.30 | -3204.40 | -3206.70 | -1.90 |
| 187 | 4-Iodophenol | -2980.30 | -2978.40 | -2978.40 | -1.90 |
| 188 | Cyclopentane | -3293.10 | -3295.00 | -3295.00 | 1.90 |
| 189 | Pyrrole-2-aldoxime | -2842.40 | -2840.50 | -2841.40 | -1.90 |
| 190 | Sarcosine | -1675.10 | -1677.00 |  | 1.90 |
| 191 | Xanthine | -2162.00 | -2160.10 | -2161.00 | -1.90 |
| 192 | Pentanamide | -3159.20 | -3161.10 | -3160.20 | 1.90 |
| 193 | trans-Cyclopentane-1,2-diol | -2909.07 | -2911.00 | -2910.00 | 1.93 |
| 194 | n-Butyl acetate | -3549.56 | -3551.50 | -3551.60 | 1.94 |
| 195 | Ethyl azide | -1769.47 | -1767.50 | -1761.20 | -1.97 |
| 196 | Nitroethylene | -1183.28 | -1181.30 |  | -1.98 |
| 197 | 3-Methylpentane | -4162.40 | -4160.40 | -4159.50 | -2.00 |
| 198 | Malic acid | -1328.80 | -1326.80 | -1326.10 | -2.00 |
| 199 | Methyl benzoate | -3960.30 | -3958.30 | -3959.30 | -2.00 |
| 200 | n-Amyl ether | -6644.00 | -6646.00 | -6648.30 | 2.00 |
| 201 | Tetrahydropyran | -3143.00 | -3141.00 | -3140.60 | -2.00 |
| 202 | Nitrobenzene | -3094.90 | -3096.90 | -3095.80 | 2.00 |
| 203 | 1,3-Dichloropropane | -1885.30 | -1887.30 | -1886.80 | 2.00 |
| 204 | Formamide | -568.57 | -570.60 | -572.70 | 2.03 |
| 205 | 1,1-Difluoroethane | -1209.85 | -1207.80 | -1208.90 | -2.05 |
| 206 | 2,4-Dimethylhexane | -5467.35 | -5465.30 | -5465.80 | -2.05 |
| 207 | Isopropylcyclohexane | -5878.70 | -5876.60 | -5874.40 | -2.10 |
| 208 | Methanol | -726.00 | -723.90 | -723.50 | -2.10 |
| 209 | 5-Diallylaminotetrazole | -4680.80 | -4678.70 | -4678.70 | -2.10 |
| 210 | 2-Nitroisobutane | -2630.00 | -2632.10 | -2632.60 | 2.10 |
| 211 | Ethyl butanoate | -3545.00 | -3547.10 | -3546.80 | 2.10 |
| 212 | n-Hexylmercaptan | -4792.00 | -4794.10 | -4794.20 | 2.10 |
| 213 | 2-Methyl-1-hexene | -4645.96 | -4648.10 | -4649.20 | 2.14 |
| 214 | 4-Octanone | -5065.25 | -5063.10 | -5063.50 | -2.15 |
| 215 | Perfluoro-n-decane | -3047.65 | -3049.80 | -3053.70 | 2.15 |
| 216 | 1,1-Dimethylcyclohexane | -5219.65 | -5217.50 | -5216.90 | -2.15 |
| 217 | 2-Butene-1-imine | -2681.36 | -2679.20 | -2679.90 | -2.16 |
| 218 | Acetaldol | -2285.56 | -2283.40 | -2283.60 | -2.16 |
| 219 | alpha-Methylstyrene | -5044.52 | -5046.70 | -5048.40 | 2.18 |
| 220 | Dimethyl adipate | -4265.10 | -4262.90 | -4261.10 | -2.20 |
| 221 | m-Xylene | -4551.60 | -4549.40 | -4548.70 | -2.20 |
| 222 | Diisopropyl sulfide | -4782.70 | -4780.50 | -4780.00 | -2.20 |
| 223 | 1,2-Dinitroethane | -1180.00 | -1177.80 | -1177.50 | -2.20 |
| 224 | 1,2-Propanediol | -1824.60 | -1826.80 | -1826.50 | 2.20 |
| 225 | Pentafluorotoluene | -3194.40 | -3196.60 | -3196.70 | 2.20 |
| 226 | 2,3-Xylenol | -4339.19 | -4341.40 | -4340.80 | 2.21 |
| 227 | 1-Isopropoxy-2-propanol | -3835.18 | -3837.40 | -3836.90 | 2.22 |
| 228 | Benzyl benzoate | -6955.85 | -6958.10 | -6959.80 | 2.25 |
| 229 | Ethyl-n-hexylether | -5343.36 | -5341.10 | -5339.90 | -2.26 |
| 230 | Pentachlorofluoroethane | -692.06 | -689.80 | -691.70 | -2.26 |
| 231 | Perfluoroethane | -485.44 | -487.70 | -492.90 | 2.26 |
| 232 | Di-n-propylsulfate | -4108.17 | -4105.90 | -4099.00 | -2.27 |
| 233 | Formaldehyde diethylacetal | -3234.59 | -3232.30 | -3232.30 | -2.29 |
| 234 | 4-Ethylphenol | -4355.70 | -4353.40 | -4353.30 | -2.30 |
| 235 | Acetylacetone (enol form) | -2685.40 | -2683.10 | -2682.40 | -2.30 |
| 236 | 1,2-Butanediol | -2481.60 | -2479.30 | -2479.40 | -2.30 |
| 237 | 3-Nitro-2-butanol | -2471.70 | -2469.40 | -2469.50 | -2.30 |
| 238 | 1,2,3-Trichloropropane | -1735.00 | -1732.70 | -1732.20 | -2.30 |
| 239 | 5-Methylcaprolactam | -4251.70 | -4254.00 | -4253.20 | 2.30 |
| 240 | Melamine | -1967.00 | -1969.30 |  | 2.30 |
| 241 | Ethyl-n-propylether | -3381.40 | -3383.70 | -3383.70 | 2.30 |
| 242 | d-Mannose | -2814.80 | -2817.10 | -2816.90 | 2.30 |
| 243 | Papaverine | -10375.00 | -10377.30 | -10375.20 | 2.30 |
| 244 | trans-1,4-Dichloro-2-butene | -2365.78 | -2368.10 | -2368.40 | 2.32 |
| 245 | trans-Decalin | -6281.32 | -6279.00 | -6279.60 | -2.32 |
| 246 | 2-Methyl-2-pentene | -3980.23 | -3977.90 | -3977.80 | -2.33 |
| 247 | Paracetamol | -4041.24 | -4038.90 | -4039.10 | -2.34 |
| 248 | 2-Ethyl-1-butene | -3991.65 | -3994.00 | -3994.00 | 2.35 |
| 249 | Butylisopropylether | -4678.94 | -4681.30 | -4681.30 | 2.36 |
| 250 | Propynal | -1667.56 | -1665.20 | -1665.40 | -2.36 |
| 251 | 2,2,3-Trimethylpentane | -5467.47 | -5465.10 | -5465.40 | -2.37 |
| 252 | 2-Chloro-1,3-propanediol | -1687.47 | -1685.10 | -1684.70 | -2.37 |
| 253 | 4-Methyl-1-pentene | -3998.72 | -4001.10 | -4001.60 | 2.38 |
| 254 | 4-Biphenylamine | -6379.59 | -6377.20 | -6377.80 | -2.39 |
| 255 | 2,2-Dimethylpentane | -4806.30 | -4808.70 | -4810.20 | 2.40 |
| 256 | 2-Naphthol | -4959.00 | -4956.60 | -4955.80 | -2.40 |
| 257 | Indene | -4795.50 | -4793.10 | -4793.70 | -2.40 |
| 258 | Benzaldehyde | -3530.30 | -3527.90 | -3529.20 | -2.40 |
| 259 | 2-Methylindole | -4891.90 | -4889.50 | -4889.00 | -2.40 |
| 260 | Angelic acid | -2656.90 | -2654.50 | -2656.40 | -2.40 |
| 261 | 5-Phenyltetrazole | -3907.50 | -3909.90 | -3909.70 | 2.40 |
| 262 | 2,6-Dimethylbenzoic acid | -4530.10 | -4527.70 | -4528.80 | -2.40 |
| 263 | Triisobutylphosphate | -7988.40 | -7990.80 | -7990.50 | 2.40 |
| 264 | 1-Methoxy-2-propanone | -2335.78 | -2338.20 | -2340.40 | 2.42 |
| 265 | Malonic acid | -861.15 | -863.60 | -861.40 | 2.45 |
| 266 | 2,3,3-Trimethylpentane | -5470.86 | -5468.40 | -5467.70 | -2.46 |
| 267 | Sulfolane | -2879.92 | -2882.40 | -2883.90 | 2.48 |
| 268 | Benzenemethanethiol | -4546.90 | -4544.40 | -4544.40 | -2.50 |
| 269 | 5-Amino-2-methyltetrazole | -1708.40 | -1705.90 |  | -2.50 |
| 270 | Ethylcarbamate | -1661.70 | -1659.20 | -1659.80 | -2.50 |
| 271 | 2-Amino-4,6-dinitrophenol | -2834.00 | -2831.50 | -2832.10 | -2.50 |
| 272 | Isopentyl isovalerate | -6153.20 | -6155.70 | -6155.20 | 2.50 |
| 273 | 2-Hydroxyethylacrylate | -2553.08 | -2555.60 | -2555.80 | 2.52 |
| 274 | Glutaraldehyde | -2747.08 | -2749.60 | -2750.20 | 2.52 |
| 275 | 1,2,4-Trimethylbenzene | -5198.28 | -5200.80 | -5201.60 | 2.52 |
| 276 | 4-Methyl-cis-2-pentene | -3991.73 | -3989.20 | -3990.00 | -2.53 |
| 277 | Di-s-butylether | -5322.87 | -5325.40 | -5324.90 | 2.53 |
| 278 | 3-Ethyl-1-hexene | -5313.75 | -5311.20 | -5310.90 | -2.55 |
| 279 | 6-Methyl-1-heptene | -5308.85 | -5306.30 | -5304.50 | -2.55 |
| 280 | 2,2-Dimethylheptane | -6115.97 | -6113.40 | -6112.40 | -2.57 |
| 281 | 4-Ethyl-1,3-dimethylbenzene | -5855.78 | -5853.20 | -5852.90 | -2.58 |
| 282 | trans-1,2-Dibenzoylethylene | -7901.79 | -7899.20 | -7900.20 | -2.59 |
| 283 | Methylpropylether | -2739.31 | -2741.90 | -2741.80 | 2.59 |
| 284 | Acenaphthene | -6223.30 | -6220.70 | -6217.90 | -2.60 |
| 285 | 3,3-Dimethylpentane | -4810.40 | -4813.00 | -4813.40 | 2.60 |
| 286 | Isopentane | -3503.40 | -3506.00 | -3505.00 | 2.60 |
| 287 | N,N-Dimethylformamide | -1943.10 | -1945.70 | -1943.30 | 2.60 |
| 288 | 2-Nitrophenol | -2885.10 | -2887.70 | -2887.90 | 2.60 |
| 289 | 2-(n-Butoxy)ethylacetate | -4728.05 | -4730.70 | -4732.00 | 2.65 |
| 290 | Cyclopropanecarboxamide | -2385.66 | -2383.00 | -2384.80 | -2.66 |
| 291 | n-Butylmethacrylate | -4680.94 | -4683.60 | -4684.40 | 2.66 |
| 292 | Hydroxyacetic acid | -695.43 | -698.10 | -696.80 | 2.67 |
| 293 | Methyl formate | -980.13 | -982.80 | -983.70 | 2.67 |
| 294 | Methylcyclohexane | -4568.48 | -4565.80 | -4566.80 | -2.68 |
| 295 | Benzylbromide | -3757.89 | -3755.20 | -3754.70 | -2.69 |
| 296 | Phenylurea | -3681.89 | -3679.20 | -3679.30 | -2.69 |
| 297 | o-Cymene | -5862.60 | -5859.90 | -5860.10 | -2.70 |
| 298 | Hydantoin | -1304.60 | -1301.90 | -1303.10 | -2.70 |
| 299 | 3-Iodophenol | -2981.10 | -2978.40 | -2978.60 | -2.70 |
| 300 | Acetaldehyde | -1167.20 | -1169.90 | -1169.40 | 2.70 |
| 301 | 3-Methyl-trans-2-pentene | -3984.20 | -3981.50 | -3981.00 | -2.70 |
| 302 | Glucose pentaacetate | -7190.90 | -7193.60 | -7194.80 | 2.70 |
| 303 | 2-Methoxy-1,3-dioxolane | -2189.18 | -2191.90 | -2193.90 | 2.72 |
| 304 | Ethylisopropylketone | -3752.86 | -3755.60 | -3756.90 | 2.74 |
| 305 | 4-Heptanone | -4412.36 | -4409.60 | -4408.80 | -2.76 |
| 306 | Ethyl crotonate | -3372.64 | -3375.40 | -3374.90 | 2.76 |
| 307 | 2,3,3,4-Tetramethylpentane | -6126.27 | -6123.50 | -6120.10 | -2.77 |
| 308 | 2-Methyl-2-butene | -3330.39 | -3327.60 | -3325.20 | -2.79 |
| 309 | 2-Methyl-2-nitro-1-propanol | -2451.70 | -2448.90 | -2445.70 | -2.80 |
| 310 | p-Anthranilic acid | -3344.00 | -3346.80 | -3348.00 | 2.80 |
| 311 | trans-Hexahydroindan | -5652.00 | -5654.80 | -5653.90 | 2.80 |
| 312 | 4-Methyl-trans-2-pentene | -3987.21 | -3984.40 | -3984.70 | -2.81 |
| 313 | 1,3-Dichloro-trans-2-butene | -2356.06 | -2358.90 | -2359.70 | 2.84 |
| 314 | Propanolamine | -2177.07 | -2174.20 | -2171.90 | -2.87 |
| 315 | Methyl-n-pentylether | -4049.68 | -4046.80 | -4046.60 | -2.88 |
| 316 | 1-Ethylcyclohexene | -5042.10 | -5039.20 | -5040.10 | -2.90 |
| 317 | Phenazine | -6105.60 | -6102.70 | -6099.30 | -2.90 |
| 318 | 3-Methyl-1-pentene | -4000.60 | -4003.50 | -4003.70 | 2.90 |
| 319 | Thiosemicarbazide | -1712.40 | -1715.30 | -1714.70 | 2.90 |
| 320 | m-Tolualdehyde | -4170.87 | -4173.80 | -4172.80 | 2.93 |
| 321 | 3,4-Dimethylhexane | -5472.54 | -5469.60 | -5468.90 | -2.94 |
| 322 | 1-Phenylethylformate | -4626.96 | -4629.90 | -4632.20 | 2.94 |
| 323 | Butylcyclopentane | -5903.95 | -5906.90 | -5904.00 | 2.95 |
| 324 | 3-Methylbiphenyl | -6916.20 | -6913.20 | -6913.10 | -3.00 |
| 325 | Diphenylcarbonate | -6143.60 | -6140.60 | -6134.50 | -3.00 |
| 326 | s-trans-1,3-Butadiene | -2541.30 | -2538.30 | -2538.90 | -3.00 |
| 327 | 1-Hexene | -4003.40 | -4000.40 | -3999.60 | -3.00 |
| 328 | 1-Propanol | -2021.20 | -2018.20 | -2018.90 | -3.00 |
| 329 | 1,2-Diiodoethane | -1368.00 | -1371.00 | -1372.40 | 3.00 |
| 330 | Diphenylsulfone | -6528.50 | -6525.50 | -6522.30 | -3.00 |
| 331 | 1,3-Dioxolane | -1700.80 | -1703.80 | -1701.70 | 3.00 |
| 332 | s-Butylamine | -3010.60 | -3013.60 | -3011.60 | 3.00 |
| 333 | 3-Methyleneheptane | -5301.93 | -5298.90 | -5300.40 | -3.03 |
| 334 | 2,2-Dimethoxypentane | -4535.38 | -4532.30 | -4531.20 | -3.08 |
| 335 | 1,1-Dimethylcyclopentane | -4586.51 | -4589.60 | -4589.90 | 3.09 |
| 336 | Di-n-butylamine | -5651.20 | -5648.10 | -5648.10 | -3.10 |
| 337 | 2-Butanone | -2445.80 | -2448.90 | -2448.10 | 3.10 |
| 338 | n-Propylcarbamate | -2308.60 | -2311.70 | -2312.20 | 3.10 |
| 339 | n-Propylformate | -2273.98 | -2277.10 | -2279.90 | 3.12 |
| 340 | 2-Methoxy-1-propanol | -2555.73 | -2552.60 | -2553.60 | -3.13 |
| 341 | 1,4-Dimethylbenzene | -4552.60 | -4549.40 | -4548.50 | -3.20 |
| 342 | Dimethyl isophthalate | -4651.50 | -4654.70 | -4653.00 | 3.20 |
| 343 | Triethoxymethane | -4365.50 | -4362.30 | -4359.70 | -3.20 |
| 344 | N,N'-Dimethylurea | -2011.70 | -2008.50 | -2004.10 | -3.20 |
| 345 | N-Methylpiperidone | -3642.70 | -3639.50 | -3633.60 | -3.20 |
| 346 | Chloroacetamide | -1044.00 | -1047.20 | -1047.80 | 3.20 |
| 347 | Diethylamine | -3035.00 | -3038.20 | -3039.70 | 3.20 |
| 348 | Ethylcyclobutane | -4019.81 | -4016.60 | -4015.70 | -3.21 |
| 349 | Phenyl-di-methoxymethane | -4951.68 | -4954.90 | -4954.20 | 3.22 |
| 350 | 2-Mercaptoethanol | -1999.05 | -2002.30 | -2002.60 | 3.25 |
| 351 | 2,3-Dimethylhexane | -5471.78 | -5468.50 | -5468.00 | -3.28 |
| 352 | 1-Octanethiol | -6102.29 | -6099.00 | -6098.90 | -3.29 |
| 353 | Di-n-propylsulfide | -4794.20 | -4797.50 | -4798.40 | 3.30 |
| 354 | 2,3-Dichloro-1-propanol | -1704.00 | -1707.30 | -1707.60 | 3.30 |
| 355 | Asparagine | -1928.50 | -1931.80 | -1931.00 | 3.30 |
| 356 | Glyceryl-1-benzoate | -4876.00 | -4879.30 | -4878.70 | 3.30 |
| 357 | Glyoxaldioxime | -1272.00 | -1275.30 | -1278.60 | 3.30 |
| 358 | Isovaleraldehyde | -3122.47 | -3125.80 | -3127.30 | 3.33 |
| 359 | Isopropylcyclopentane | -5248.26 | -5251.60 | -5249.50 | 3.34 |
| 360 | 2,4-Dimethyl-2-pentene | -4635.47 | -4632.10 | -4633.90 | -3.37 |
| 361 | Amyl formate | -3585.48 | -3582.10 | -3582.30 | -3.38 |
| 362 | Benz[a]anthracene | -8969.00 | -8972.40 | -8980.40 | 3.40 |
| 363 | 1-Naphthol | -4960.00 | -4956.60 | -4953.40 | -3.40 |
| 364 | p-Cresol | -3701.10 | -3697.70 | -3697.10 | -3.40 |
| 365 | Perseitol | -3496.80 | -3493.40 | -3494.10 | -3.40 |
| 366 | Ethylbenzene | -4563.90 | -4560.50 | -4560.80 | -3.40 |
| 367 | 1,2,2,3-Tetrachloropropane | -1594.90 | -1591.50 | -1589.70 | -3.40 |
| 368 | Proline | -2746.20 | -2749.60 | -2751.60 | 3.40 |
| 369 | Etioporphin I | -18028.00 | -18031.40 | -18040.70 | 3.40 |
| 370 | 1,2,4-Triethylbenzene | -7165.41 | -7162.00 | -7160.90 | -3.41 |
| 371 | Ethylisobutylether | -4031.28 | -4034.70 | -4033.70 | 3.42 |
| 372 | Triptane | -4808.06 | -4811.50 | -4810.50 | 3.44 |
| 373 | 2,5-Dimethyloctane | -6775.54 | -6772.10 | -6773.50 | -3.44 |
| 374 | trans-3-Decen-1-yne | -6326.36 | -6329.80 | -6333.60 | 3.44 |
| 375 | 4-Methylheptane | -5468.90 | -5465.40 | -5465.30 | -3.50 |
| 376 | Dimethyl sulfone | -1796.70 | -1793.20 | -1792.10 | -3.50 |
| 377 | 1,2-Propanediol 1-methacrylate | -3843.20 | -3839.70 | -3839.20 | -3.50 |
| 378 | epsilon-Caprolactam | -3606.50 | -3603.00 | -3602.30 | -3.50 |
| 379 | n-Butylmethylsulfide | -4142.10 | -4145.60 | -4145.50 | 3.50 |
| 380 | Azo-n-propane | -4376.28 | -4379.80 | -4381.80 | 3.52 |
| 381 | 1-Isopropyl-4-methylbenzene | -5861.85 | -5858.30 | -5859.40 | -3.55 |
| 382 | n-Propyl benzoate | -5256.15 | -5252.60 | -5252.00 | -3.55 |
| 383 | 2-Methyl-1-heptene | -5304.17 | -5300.60 | -5301.60 | -3.57 |
| 384 | 1-Iodopropane | -2115.38 | -2111.80 | -2111.50 | -3.58 |
| 385 | 2-Cyclohexene-1-one | -3335.82 | -3339.40 | -3338.90 | 3.58 |
| 386 | 3-Chlorobenzoylchloride | -3183.61 | -3187.20 | -3187.50 | 3.59 |
| 387 | 2-Bromopropane | -2052.00 | -2048.40 | -2050.10 | -3.60 |
| 388 | 1,2-Butanediamine | -3170.90 | -3174.50 | -3174.20 | 3.60 |
| 389 | Propylenediamine | -2513.50 | -2517.10 | -2520.50 | 3.60 |
| 390 | 2-Diethylaminoethanol | -4198.30 | -4201.90 | -4201.10 | 3.60 |
| 391 | Triphenylethylene | -10397.00 | -10400.60 | -10399.20 | 3.60 |
| 392 | Trimellitic anhydride | -3221.19 | -3224.80 | -3226.20 | 3.61 |
| 393 | 1-t-Butoxy-2-[2-(t-butoxy)propoxy]propane | -8992.84 | -8989.20 | -8986.30 | -3.64 |
| 394 | 1,5-Hexadiene | -3846.84 | -3843.20 | -3841.30 | -3.64 |
| 395 | 2-Methylnonane | -6772.06 | -6768.40 | -6768.10 | -3.66 |
| 396 | 3-Heptanone | -4409.96 | -4406.30 | -4408.40 | -3.66 |
| 397 | 3-Ethyl-1,2-dimethylbenzene | -5859.38 | -5855.70 | -5855.00 | -3.68 |
| 398 | Diisopentylsulfide | -7399.10 | -7395.40 | -7397.90 | -3.70 |
| 399 | 2-Ethyl-3-methl-1-butene | -4644.50 | -4648.20 | -4647.40 | 3.70 |
| 400 | 4,4'-Dimethyl-2,2'-bipyridyl | -6546.70 | -6543.00 | -6545.30 | -3.70 |
| 401 | 3,5-Dinitroaniline | -3037.00 | -3040.70 | -3041.30 | 3.70 |
| 402 | Dicyclohexylamine | -7782.40 | -7786.10 | -7785.50 | 3.70 |
| 403 | cis-Decalin | -6292.52 | -6288.80 | -6290.10 | -3.72 |
| 404 | 2,2,5-Trimethylhexane | -6110.87 | -6114.60 | -6115.30 | 3.73 |
| 405 | 2-Methyl-1-nonene | -6609.24 | -6605.50 | -6606.00 | -3.74 |
| 406 | 4-Methyl-1-heptene | -5312.25 | -5308.50 | -5308.00 | -3.75 |
| 407 | 2,2-Dimethylhexane | -5462.49 | -5458.70 | -5458.50 | -3.79 |
| 408 | 1,1,2,2-Tetraphenylethane | -13596.00 | -13592.20 | -13594.50 | -3.80 |
| 409 | 1,4-Diethylbenzene | -5863.00 | -5859.20 | -5859.60 | -3.80 |
| 410 | Lactic acid | -1344.90 | -1341.10 | -1340.60 | -3.80 |
| 411 | Capric acid t-butylperoxyester | -8828.30 | -8824.50 | -8824.50 | -3.80 |
| 412 | Citric acid | -1961.90 | -1965.70 | -1965.20 | 3.80 |
| 413 | 2-Ethylhexyl acetate | -6169.95 | -6166.10 | -6167.70 | -3.85 |
| 414 | 1-Methyl-3-propylbenzene | -5863.65 | -5859.80 | -5860.10 | -3.85 |
| 415 | 2-Methylbenzylalcohol | -4373.59 | -4369.70 | -4369.90 | -3.89 |
| 416 | 1-Nitropentane | -3324.00 | -3320.10 | -3318.80 | -3.90 |
| 417 | 2-(2-Methoxyethoxy)ethanol | -3095.00 | -3091.10 | -3092.40 | -3.90 |
| 418 | Vinylformate | -1460.00 | -1456.10 | -1456.70 | -3.90 |
| 419 | n-Hexanoic acid | -3493.80 | -3489.90 | -3489.20 | -3.90 |
| 420 | Glycylglycine | -1971.80 | -1967.90 | -1964.70 | -3.90 |
| 421 | Isopentylacetate | -4199.87 | -4203.80 | -4204.50 | 3.93 |
| 422 | Isobutylmethylether | -3388.86 | -3392.80 | -3393.50 | 3.94 |
| 423 | 2,2,4,4,6,8,8-Heptamethylnonane | -10685.85 | -10681.90 | -10682.60 | -3.95 |
| 424 | Diethyl maleate | -4068.13 | -4072.10 | -4072.50 | 3.97 |
| 425 | s-Butylbenzene | -5873.48 | -5869.50 | -5870.20 | -3.98 |
| 426 | 2-Tolualdehyde | -4181.09 | -4177.10 | -4176.00 | -3.99 |
| 427 | 1-Methylcyclohexene | -4388.30 | -4384.30 | -4383.50 | -4.00 |
| 428 | Phloroglucinol | -2634.00 | -2638.00 | -2636.00 | 4.00 |
| 429 | 1-Methylpyrrole | -3030.30 | -3034.30 | -3028.70 | 4.00 |
| 430 | 2,3-Dimethylbutane | -4157.38 | -4161.40 | -4162.00 | 4.02 |
| 431 | Methyl-n-propylsulfide | -3489.08 | -3493.10 | -3492.10 | 4.02 |
| 432 | 1-Pentene | -3351.94 | -3347.90 | -3346.60 | -4.04 |
| 433 | 2-Methylnaphthalene | -5806.46 | -5810.50 | -5811.40 | 4.04 |
| 434 | Propyl isobutanoate | -4194.06 | -4198.10 | -4198.60 | 4.04 |
| 435 | 3,3,3-Trifluoropropene | -1517.05 | -1513.00 | -1511.70 | -4.05 |
| 436 | 2-Ethyl-1,3-dimethylbenzene | -5859.76 | -5855.70 | -5856.60 | -4.06 |
| 437 | 2-Heptanone | -4410.36 | -4406.30 | -4405.50 | -4.06 |
| 438 | 3-Ethyl-1-pentene | -4660.07 | -4656.00 | -4655.30 | -4.07 |
| 439 | 4-Ethyltoluene | -5210.29 | -5206.20 | -5206.20 | -4.09 |
| 440 | 1-Heptene | -4657.00 | -4652.90 | -4652.90 | -4.10 |
| 441 | Isocumene | -5218.00 | -5213.90 | -5214.30 | -4.10 |
| 442 | 2-Piperidone | -2949.20 | -2945.10 | -2945.50 | -4.10 |
| 443 | 2-Methylbiphenyl | -6917.10 | -6913.00 | -6914.50 | -4.10 |
| 444 | Ethylene carbonate | -1171.90 | -1167.80 | -1164.90 | -4.10 |
| 445 | 4-Bromobenzoic acid | -3090.10 | -3094.20 | -3095.00 | 4.10 |
| 446 | 1-Allyl-5-allylaminotetrazole | -4680.00 | -4684.10 | -4687.60 | 4.10 |
| 447 | Butylcrotonate | -4684.53 | -4680.40 | -4682.00 | -4.13 |
| 448 | Allyl iodide | -1950.56 | -1954.70 | -1953.80 | 4.14 |
| 449 | 2-Ethylhexanal | -5089.85 | -5085.70 | -5085.70 | -4.15 |
| 450 | Propyl propanoate | -3551.26 | -3547.10 | -3548.40 | -4.16 |
| 451 | 1,8-Dinitronaphthalene | -4838.80 | -4834.60 | -4834.70 | -4.20 |
| 452 | Methylbenzene | -3909.80 | -3905.60 | -3906.10 | -4.20 |
| 453 | 1,3-Dichloro-2-propanol | -1700.00 | -1695.80 | -1695.70 | -4.20 |
| 454 | Dimethylamine | -1744.60 | -1740.40 | -1739.10 | -4.20 |
| 455 | Propionic acid | -1528.30 | -1532.50 | -1531.80 | 4.20 |
| 456 | Hypoxanthine | -2431.10 | -2435.30 | -2443.90 | 4.20 |
| 457 | Triethylamine | -4377.10 | -4381.30 | -4381.20 | 4.20 |
| 458 | Cyclobutylamine | -2867.79 | -2872.00 | -2871.80 | 4.21 |
| 459 | Methylcyanoacetate | -1991.84 | -1987.60 | -1986.40 | -4.24 |
| 460 | Ethyl-isoamylketone | -5063.95 | -5059.70 | -5060.30 | -4.25 |
| 461 | Isodurene | -5843.52 | -5847.80 | -5846.40 | 4.28 |
| 462 | 2-Methyl-1-butanol | -3328.39 | -3324.10 | -3325.00 | -4.29 |
| 463 | beta-Nitrostyrene | -4183.90 | -4179.60 |  | -4.30 |
| 464 | Furfural | -2340.80 | -2345.10 | -2341.10 | 4.30 |
| 465 | 2,4,6-Trinitro-N-methylaniline | -3562.80 | -3558.50 | -3558.90 | -4.30 |
| 466 | Sarcosine-N-carboxylic anhydride | -1686.90 | -1691.20 | -1695.70 | 4.30 |
| 467 | Salicylic acid | -3024.50 | -3028.80 | -3028.10 | 4.30 |
| 468 | Ethylcyclopropane | -3371.90 | -3376.20 | -3375.50 | 4.30 |
| 469 | 5-Nonanone | -5719.91 | -5715.60 | -5714.90 | -4.31 |
| 470 | n-Butyl-4-chlorobutanoate | -4699.92 | -4695.60 | -4696.40 | -4.32 |
| 471 | 1,1,1,2,3,3,3-Heptafluoropropane | -987.76 | -992.10 |  | 4.34 |
| 472 | 3-Butene-2-imine | -2676.76 | -2681.10 | -2681.80 | 4.34 |
| 473 | n-Propyl acetate | -2894.65 | -2899.00 | -2897.20 | 4.35 |
| 474 | 2,2,5,5-Tetramethylhexane | -6760.45 | -6764.80 | -6765.70 | 4.35 |
| 475 | 2-Bromobutane | -2705.20 | -2700.80 | -2700.20 | -4.40 |
| 476 | 1-Chloro-1-fluoroethane | -1242.40 | -1246.80 | -1248.40 | 4.40 |
| 477 | Ethyleneglycol | -1179.50 | -1183.90 | -1182.60 | 4.40 |
| 478 | 2-Hexanone | -3753.80 | -3758.20 | -3759.50 | 4.40 |
| 479 | Myristonitrile | -9107.70 | -9112.10 | -9113.80 | 4.40 |
| 480 | Dibutyldisulfide | -6702.30 | -6706.70 | -6707.80 | 4.40 |
| 481 | 1-Bromopentane | -3350.19 | -3354.60 | -3355.30 | 4.41 |
| 482 | trans-Diethylcyclopropane | -4675.26 | -4679.70 | -4681.70 | 4.44 |
| 483 | Cumene | -5218.95 | -5214.50 | -5213.50 | -4.45 |
| 484 | 2-Hydroxyacetophenone | -3952.07 | -3947.60 | -3947.30 | -4.47 |
| 485 | cis-Hexahydroindan | -5659.02 | -5663.50 | -5661.20 | 4.48 |
| 486 | Isopentyl alcohol | -3328.59 | -3324.10 | -3324.90 | -4.49 |
| 487 | Butyraldehyde | -2479.40 | -2474.90 | -2475.20 | -4.50 |
| 488 | Propyne | -1938.70 | -1934.20 | -1937.70 | -4.50 |
| 489 | 3,5-Lutidine | -4066.00 | -4061.50 | -4060.00 | -4.50 |
| 490 | Methylsuccinic acid | -2175.00 | -2170.50 | -2171.90 | -4.50 |
| 491 | Ethane-1,2-dithiol | -2796.70 | -2801.20 | -2801.20 | 4.50 |
| 492 | Formanilide | -3605.70 | -3610.20 | -3608.70 | 4.50 |
| 493 | Isooctane | -5465.21 | -5460.70 | -5461.20 | -4.51 |
| 494 | trans-2-Pentene | -3340.14 | -3335.60 | -3335.80 | -4.54 |
| 495 | 2,3,3-Trimethyl-1-butene | -4640.86 | -4645.40 | -4644.80 | 4.54 |
| 496 | 2-Fluoroethanol | -1210.53 | -1215.10 | -1215.70 | 4.57 |
| 497 | 3-Ethylpentane | -4819.69 | -4815.10 | -4815.40 | -4.59 |
| 498 | Butylcyclohexane | -6530.30 | -6525.70 | -6525.70 | -4.60 |
| 499 | 4-Phenyluracil | -4739.90 | -4744.50 | -4749.60 | 4.60 |
| 500 | Anthracene | -7062.70 | -7067.30 | -7063.50 | 4.60 |
| 501 | Methylcyclopropane | -2719.10 | -2723.70 | -2725.20 | 4.60 |
| 502 | 3-Aminoacetophenone | -4261.00 | -4265.60 | -4265.80 | 4.60 |
| 503 | Methylphenylsulfone | -4154.80 | -4159.40 | -4159.20 | 4.60 |
| 504 | Isobutylene | -2681.68 | -2686.30 | -2686.60 | 4.62 |
| 505 | 4-Methoxyphenol | -3564.98 | -3569.60 | -3569.30 | 4.62 |
| 506 | Pentaethylbenzene | -9760.54 | -9765.20 | -9765.70 | 4.66 |
| 507 | t-Nonylmercaptan | -6740.33 | -6745.00 | -6746.40 | 4.67 |
| 508 | Isobutyric acid | -2188.18 | -2183.50 | -2182.40 | -4.68 |
| 509 | 2-Ethyl-2-methyl-1,3-dioxolane | -3626.20 | -3630.90 | -3631.80 | 4.70 |
| 510 | cis-3-Decen-1-yne | -6325.10 | -6329.80 | -6330.70 | 4.70 |
| 511 | 4-Methylene-2-oxetanone | -1912.50 | -1907.80 | -1906.10 | -4.70 |
| 512 | Methylenanthate | -4867.40 | -4862.70 | -4862.40 | -4.70 |
| 513 | Trimethylamine | -2422.50 | -2427.20 | -2428.10 | 4.70 |
| 514 | t-Dodecylmercaptan | -8707.10 | -8702.40 | -8700.30 | -4.70 |
| 515 | n-Pentyl-2-mercaptan | -4135.77 | -4140.50 | -4142.40 | 4.73 |
| 516 | 2,2-Dimethyloctane | -6770.84 | -6766.10 | -6766.30 | -4.74 |
| 517 | 3,3-Dimethylhexane | -5466.84 | -5462.10 | -5461.90 | -4.74 |
| 518 | S-Propylthioacetate | -3707.25 | -3712.00 | -3717.80 | 4.75 |
| 519 | 3-Hexanone | -3758.56 | -3753.80 | -3754.50 | -4.76 |
| 520 | 5-Methyl-1-hexene | -4658.56 | -4653.80 | -4651.90 | -4.76 |
| 521 | 2,4,6-Trinitroanisol | -3284.10 | -3279.30 | -3273.80 | -4.80 |
| 522 | n-Propylnitrate | -1966.00 | -1970.80 | -1966.90 | 4.80 |
| 523 | Dimethyl disulfide | -2788.30 | -2793.10 | -2795.00 | 4.80 |
| 524 | Benzanilide | -6598.80 | -6603.60 | -6605.10 | 4.80 |
| 525 | Thioanisole | -4551.10 | -4555.90 | -4558.30 | 4.80 |
| 526 | 2,4-Dimethyloctane | -6776.01 | -6771.20 | -6771.00 | -4.81 |
| 527 | Pentafluorophenol | -2349.71 | -2344.90 | -2346.00 | -4.81 |
| 528 | Dimethyl sulfate | -1512.49 | -1517.30 | -1517.70 | 4.81 |
| 529 | Isobutyl isobutyrate | -4844.28 | -4849.10 | -4849.90 | 4.82 |
| 530 | 2-n-Hexoxyethanol | -5164.07 | -5159.20 | -5161.50 | -4.87 |
| 531 | 1,4-Dioxane | -2362.23 | -2367.10 | -2369.80 | 4.87 |
| 532 | Acetic acid | -875.12 | -880.00 | -880.00 | 4.88 |
| 533 | 3-Ethyltoluene | -5211.38 | -5206.50 | -5205.80 | -4.88 |
| 534 | 3-Methyl-1-pentanol | -3983.88 | -3979.00 | -3977.60 | -4.88 |
| 535 | 7,7-Dimethoxybicyclo[2.2.1]heptane | -5408.32 | -5413.20 | -5408.30 | 4.88 |
| 536 | Triethanolamine | -3840.60 | -3835.70 | -3836.90 | -4.90 |
| 537 | Methylchloroacetate | -1451.00 | -1446.10 | -1446.20 | -4.90 |
| 538 | 2-Aminoethoxyethanol | -2710.20 | -2705.30 | -2707.50 | -4.90 |
| 539 | Propanal | -1817.50 | -1822.40 | -1821.90 | 4.90 |
| 540 | 1-Chloronaphthalene | -5009.20 | -5014.10 | -5011.80 | 4.90 |
| 541 | 1,2,3-Triethylbenzene | -7169.14 | -7164.20 | -7163.70 | -4.94 |
| 542 | 7-Methyl-1-octene | -5963.65 | -5958.70 | -5959.50 | -4.95 |
| 543 | 4-Methylpyridine | -3420.60 | -3415.60 | -3416.50 | -5.00 |
| 544 | 1-Methylcyclopentene | -3753.80 | -3748.80 | -3750.50 | -5.00 |
| 545 | Phenylacetic acid | -3895.40 | -3890.40 | -3890.80 | -5.00 |
| 546 | cis-Stilbene | -7404.00 | -7399.00 | -7396.20 | -5.00 |
| 547 | Benzoyl chloride | -3332.60 | -3337.60 | -3335.30 | 5.00 |
| 548 | Isobutyl formate | -2923.10 | -2928.10 | -2927.40 | 5.00 |
| 549 | 1-Methyl-4-propylbenzene | -5864.82 | -5859.80 | -5859.70 | -5.02 |
| 550 | 3-Methylhexane | -4818.14 | -4813.10 | -4813.50 | -5.04 |
| 551 | 2-Ethyl-1-hexanoic acid | -4803.25 | -4798.20 | -4798.40 | -5.05 |
| 552 | 2-Phenyl-1-propanol | -5037.08 | -5032.00 | -5032.30 | -5.08 |
| 553 | Benzyl ethyl ether | -5094.30 | -5089.20 | -5089.30 | -5.10 |
| 554 | Allylamine | -2204.00 | -2198.90 | -2195.20 | -5.10 |
| 555 | o-Cresol | -3695.80 | -3700.90 | -3700.40 | 5.10 |
| 556 | 2-Methyl-1-propanal | -2468.30 | -2473.40 | -2472.50 | 5.10 |
| 557 | Dimethyl sulfite | -1723.20 | -1728.30 | -1727.60 | 5.10 |
| 558 | Isopropylamine | -2356.10 | -2361.20 | -2359.40 | 5.10 |
| 559 | 2-Naphthoic acid | -5138.00 | -5143.10 | -5143.80 | 5.10 |
| 560 | 2-Methoxyphenol | -3574.71 | -3569.60 | -3569.20 | -5.11 |
| 561 | 1-Iodohexane | -4074.36 | -4069.20 | -4068.70 | -5.16 |
| 562 | 4-Methyl-1-octanol | -5941.57 | -5936.40 | -5935.80 | -5.17 |
| 563 | t-Butylhydroperoxide | -2710.00 | -2704.80 | -2704.40 | -5.20 |
| 564 | Acetophenone | -4151.60 | -4156.80 | -4155.80 | 5.20 |
| 565 | Cyclopentanone | -2873.50 | -2878.70 | -2878.40 | 5.20 |
| 566 | Neohexane | -4150.98 | -4156.20 | -4155.20 | 5.22 |
| 567 | trans-1,4-Diethylcyclohexane | -6531.84 | -6526.60 | -6525.00 | -5.24 |
| 568 | Cyclopentanonedimethylacetal | -4312.86 | -4318.10 | -4318.80 | 5.24 |
| 569 | n-Propylmethacrylate | -4025.84 | -4031.10 | -4030.40 | 5.26 |
| 570 | Elaidic acid | -11153.20 | -11147.90 | -11149.10 | -5.30 |
| 571 | 9-Acridinamine | -6704.00 | -6698.70 | -6700.00 | -5.30 |
| 572 | Methyl-t-butylsulfide | -4127.20 | -4121.90 | -4121.00 | -5.30 |
| 573 | Pyrazine | -2287.00 | -2281.70 | -2283.00 | -5.30 |
| 574 | trans-2-Heptene | -4645.80 | -4640.50 | -4639.90 | -5.30 |
| 575 | Aspartic acid | -1602.90 | -1608.20 | -1608.30 | 5.30 |
| 576 | 1,3-Dioxane | -2342.40 | -2337.10 | -2337.90 | -5.30 |
| 577 | R134a | -872.31 | -867.00 | -868.20 | -5.31 |
| 578 | 1-Ethyl-1-methylcyclopentane | -5244.55 | -5249.90 | -5248.80 | 5.35 |
| 579 | 2-Methyl-1-butene | -3337.84 | -3343.20 | -3342.90 | 5.36 |
| 580 | Di-n-butylether | -5346.47 | -5341.10 | -5341.20 | -5.37 |
| 581 | 3,3,5-Trimethylheptane | -6779.20 | -6773.80 | -6774.00 | -5.40 |
| 582 | Chloroacetic acid | -729.00 | -723.60 | -723.00 | -5.40 |
| 583 | Anisole | -3782.90 | -3777.50 | -3776.60 | -5.40 |
| 584 | 2,2-Dimethyl-1,3-dioxane | -3607.20 | -3612.60 | -3613.60 | 5.40 |
| 585 | Bromopentafluoroethane | -569.72 | -564.30 |  | -5.42 |
| 586 | 6-Aminohexaneamide | -3964.08 | -3969.50 | -3968.40 | 5.42 |
| 587 | 2-Methyl-2-propene-1-imine | -2677.26 | -2682.70 | -2683.00 | 5.44 |
| 588 | N,N-Dimethylethanolamine | -2891.24 | -2896.70 | -2898.90 | 5.46 |
| 589 | n-Hexylformate | -4240.07 | -4234.60 | -4233.40 | -5.47 |
| 590 | Tri-n-pentylamine | -10259.09 | -10253.60 | -10254.70 | -5.49 |
| 591 | Isobutylmercaptan | -3473.50 | -3468.00 | -3468.30 | -5.50 |
| 592 | Sorbitol | -3026.90 | -3032.40 | -3032.60 | 5.50 |
| 593 | (L)-Alanine | -1619.10 | -1624.60 | -1625.10 | 5.50 |
| 594 | Ethylcyclohexane | -5226.22 | -5220.70 | -5220.60 | -5.52 |
| 595 | Allyl bromide | -1886.98 | -1892.50 | -1892.20 | 5.52 |
| 596 | 2,4-Pentanediol | -3128.64 | -3123.10 | -3122.70 | -5.54 |
| 597 | Tripropylamine | -6335.66 | -6341.20 | -6340.50 | 5.54 |
| 598 | Ethyl isobutyrate | -3540.02 | -3545.60 | -3546.00 | 5.58 |
| 599 | Myristic acid t-butylperoxyester | -11440.00 | -11434.40 | -11431.70 | -5.60 |
| 600 | Carbitol | -3738.50 | -3732.90 | -3733.00 | -5.60 |
| 601 | p-Toluidine | -4020.00 | -4014.40 | -4015.10 | -5.60 |
| 602 | 1,3-Diiodopropane | -2029.10 | -2023.50 | -2021.50 | -5.60 |
| 603 | Ethyl hydroperoxide | -1402.40 | -1408.00 | -1408.60 | 5.60 |
| 604 | Dithiodilactic acid | -4030.60 | -4025.00 | -4023.40 | -5.60 |
| 605 | 5,5-Dimethylhydantoin | -2583.70 | -2589.30 | -2590.40 | 5.60 |
| 606 | Butyrophenone | -5464.95 | -5459.30 | -5460.10 | -5.65 |
| 607 | 3-Ethylheptanal | -5748.65 | -5743.00 | -5744.30 | -5.65 |
| 608 | 1,4-Dimethyl-2,6,7-trioxabicyclo[2.2.2]octane | -3838.44 | -3844.10 | -3849.00 | 5.66 |
| 609 | 2-Methyl-1H-indene | -5428.61 | -5434.30 | -5436.20 | 5.69 |
| 610 | 3-Methylcyclopentene | -3766.50 | -3760.80 | -3761.40 | -5.70 |
| 611 | 4-Amino-3-azido-1,2,4-triazole | -1788.20 | -1782.50 | -1778.40 | -5.70 |
| 612 | 1,4-Dichlorobutane | -2534.00 | -2539.70 | -2540.20 | 5.70 |
| 613 | Ethyl-n-octylsulfide | -7413.04 | -7407.30 | -7407.40 | -5.74 |
| 614 | Ethyl-n-propyldisulfide | -4743.56 | -4749.30 | -4751.90 | 5.74 |
| 615 | cis-2-Hexene | -3994.87 | -3989.10 | -3989.10 | -5.77 |
| 616 | 2,2,3,4-Tetramethylpentane | -6122.20 | -6116.40 | -6115.60 | -5.80 |
| 617 | trans-2-Methylcyclohexanol | -4380.20 | -4374.40 | -4373.30 | -5.80 |
| 618 | Azelaic acid | -4773.90 | -4779.70 | -4778.40 | 5.80 |
| 619 | Androsterone | -11095.00 | -11100.80 | -11102.30 | 5.80 |
| 620 | Benzene | -3267.62 | -3261.80 | -3262.20 | -5.82 |
| 621 | Ethyl formate | -1618.88 | -1624.70 | -1627.60 | 5.82 |
| 622 | cis-Diethylcyclopropane | -4678.66 | -4684.50 | -4681.40 | 5.84 |
| 623 | Tetraethylmethane | -6128.78 | -6122.90 | -6123.40 | -5.88 |
| 624 | 2-Propanethiol | -2821.89 | -2816.00 | -2810.60 | -5.89 |
| 625 | Enanthic acid | -4148.30 | -4142.40 | -4141.60 | -5.90 |
| 626 | Tetraphenylethylene | -13401.00 | -13395.10 | -13395.80 | -5.90 |
| 627 | Di-n-butylsulfone | -5712.70 | -5706.80 | -5704.60 | -5.90 |
| 628 | Piperidine | -3453.90 | -3448.00 | -3445.40 | -5.90 |
| 629 | Rhamnose | -3004.90 | -2999.00 | -3000.70 | -5.90 |
| 630 | Threonine | -2084.60 | -2090.50 | -2092.80 | 5.90 |
| 631 | 1-Butanethiol | -3483.30 | -3489.20 | -3489.40 | 5.90 |
| 632 | 5-Aminohexanoic acid | -3578.47 | -3584.40 | -3584.90 | 5.93 |
| 633 | Ethyl benzoate | -4594.15 | -4600.10 | -4598.90 | 5.95 |
| 634 | 2,4,4-Trimethylhexane | -6123.97 | -6118.00 | -6118.50 | -5.97 |
| 635 | 2,3-Butyleneglycol | -2463.70 | -2469.70 | -2470.10 | 6.00 |
| 636 | Heptane | -4816.00 | -4810.00 | -4810.50 | -6.00 |
| 637 | 1,2-Dichloropropane | -1883.20 | -1889.20 | -1888.20 | 6.00 |
| 638 | 2,6-Dimethyloctane | -6776.01 | -6770.00 | -6771.30 | -6.01 |
| 639 | Allylmethylsulfone | -2936.46 | -2942.50 | -2942.70 | 6.04 |
| 640 | 1,1,1,2,3,3-Hexafluoropropane | -1176.14 | -1182.20 |  | 6.06 |
| 641 | 2,3-Dimethyl-2-butene | -3975.86 | -3969.80 | -3970.70 | -6.06 |
| 642 | Glycine | -974.14 | -980.20 | -982.70 | 6.06 |
| 643 | 1,3-Diethylbenzene | -5866.37 | -5860.30 | -5860.20 | -6.07 |
| 644 | Ethyl-t-butylsulfide | -4779.87 | -4773.80 | -4773.30 | -6.07 |
| 645 | 2-Ethyltoluene | -5213.68 | -5207.60 | -5207.30 | -6.08 |
| 646 | Isobutylbenzene | -5870.09 | -5864.00 | -5864.00 | -6.09 |
| 647 | s-Butylformate | -2916.21 | -2922.30 | -2923.50 | 6.09 |
| 648 | 2,2-Dimethyl-3-ethylpentane | -6127.20 | -6121.10 | -6119.40 | -6.10 |
| 649 | Methylamine | -1060.80 | -1054.70 | -1056.20 | -6.10 |
| 650 | Bromobenzene | -3111.90 | -3118.00 | -3120.70 | 6.10 |
| 651 | 3-Chloropropanoic acid | -1370.00 | -1376.10 | -1376.70 | 6.10 |
| 652 | Dimethylglyoxime | -2555.60 | -2561.70 | -2549.00 | 6.10 |
| 653 | 3-Methylheptane | -5472.03 | -5465.90 | -5465.80 | -6.13 |
| 654 | 2-Chloro-1,1,1,2-tetrafluoroethane | -722.96 | -716.80 |  | -6.16 |
| 655 | 1-Octene-3-yne | -5007.02 | -5013.20 | -5022.50 | 6.18 |
| 656 | 1,1,1-Trimethoxyethane | -3072.99 | -3066.80 | -3063.70 | -6.19 |
| 657 | Isoxazole | -1654.00 | -1647.80 |  | -6.20 |
| 658 | 1,8-Dimethylnaphthalene | -6463.20 | -6457.00 | -6459.20 | -6.20 |
| 659 | Acrylic acid | -1369.10 | -1375.30 | -1374.30 | 6.20 |
| 660 | Veratrole | -4286.90 | -4293.10 | -4296.30 | 6.20 |
| 661 | 3,beta-Dinitrostyrene | -4008.40 | -4014.60 |  | 6.20 |
| 662 | Diisobutylsulfide | -6093.20 | -6099.40 | -6096.50 | 6.20 |
| 663 | 2,3-Dimethyloctane | -6779.65 | -6773.40 | -6773.00 | -6.25 |
| 664 | Methylenecyclopentane | -3772.66 | -3766.40 | -3764.20 | -6.26 |
| 665 | Perfluoro-n-octane | -2415.57 | -2409.30 | -2408.60 | -6.27 |
| 666 | 3,3-Dimethyl-1-butene | -3991.36 | -3997.70 | -3997.70 | 6.34 |
| 667 | Methacrylic acid | -2025.06 | -2018.70 | -2019.50 | -6.36 |
| 668 | Indane | -4982.47 | -4976.10 | -4978.10 | -6.37 |
| 669 | n-Nonylmercaptan | -6757.89 | -6751.50 | -6751.50 | -6.39 |
| 670 | 1-t-Butyl-4-ethylbenzene | -7168.00 | -7161.60 | -7162.20 | -6.40 |
| 671 | Diethyl sulphide | -3486.10 | -3492.50 | -3492.60 | 6.40 |
| 672 | Methyl acetate | -1593.90 | -1600.30 | -1600.30 | 6.40 |
| 673 | trans-1,3-Dimethylcyclopentane | -4590.49 | -4596.90 | -4597.50 | 6.41 |
| 674 | Methylethanolamine | -2213.82 | -2207.40 | -2205.80 | -6.42 |
| 675 | 2-Nonanone | -5721.35 | -5714.90 | -5714.00 | -6.45 |
| 676 | cis-2,5-Dimethyl-3-hexene | -5287.31 | -5293.80 | -5296.00 | 6.49 |
| 677 | Cyclohexanone | -3517.60 | -3511.10 | -3509.20 | -6.50 |
| 678 | Iodobenzene | -3192.80 | -3186.30 | -3185.20 | -6.50 |
| 679 | Methyl 2-furancarboxylate | -2769.00 | -2775.50 | -2772.90 | 6.50 |
| 680 | Butylbenzene | -5873.00 | -5866.50 | -5866.50 | -6.50 |
| 681 | Resorcinol | -2852.50 | -2846.00 | -2845.90 | -6.50 |
| 682 | Diurea | -1146.60 | -1153.10 | -1140.50 | 6.50 |
| 683 | 3,5-Dinitrotoluene | -3571.30 | -3577.80 | -3578.40 | 6.50 |
| 684 | 1-Methyl-2-propylbenzene | -5867.41 | -5860.90 | -5860.80 | -6.51 |
| 685 | 1,1-Dimethoxypentane | -4549.98 | -4556.50 | -4554.60 | 6.52 |
| 686 | 8-Methyl-1-nonene | -6617.74 | -6611.20 | -6611.70 | -6.54 |
| 687 | Benzyl formate | -3989.14 | -3982.60 | -3980.80 | -6.54 |
| 688 | 4-Formylmorpholine | -2886.06 | -2879.50 | -2882.20 | -6.56 |
| 689 | Methylvinyldichlorosilane | -2551.56 | -2545.00 |  | -6.56 |
| 690 | n-Heptylacetate | -5515.48 | -5508.90 | -5510.90 | -6.58 |
| 691 | Methyl isobutanoate | -2897.21 | -2903.80 | -2905.00 | 6.59 |
| 692 | Methylvalerate | -3564.30 | -3557.70 | -3557.10 | -6.60 |
| 693 | Spiro[5,5]undecane | -6942.40 | -6935.80 | -6937.30 | -6.60 |
| 694 | N-Acetylacetamide | -2085.00 | -2078.40 | -2076.50 | -6.60 |
| 695 | t-Butylamine | -2997.60 | -2991.00 | -2991.40 | -6.60 |
| 696 | Thietane | -2666.60 | -2673.20 | -2672.90 | 6.60 |
| 697 | 2,2-Dimethylpropane | -3492.50 | -3499.10 | -3498.80 | 6.60 |
| 698 | 2-Hexanol | -3972.78 | -3966.10 | -3965.30 | -6.68 |
| 699 | Pentane | -3511.80 | -3505.10 | -3505.30 | -6.70 |
| 700 | Trimethyleneglycol | -1843.00 | -1836.30 | -1836.20 | -6.70 |
| 701 | Vitamin C | -2344.10 | -2337.40 | -2335.70 | -6.70 |
| 702 | alpha-D-Glucose | -2804.90 | -2811.60 | -2812.20 | 6.70 |
| 703 | 4-Methyloctane | -6124.57 | -6117.80 | -6117.80 | -6.77 |
| 704 | n-Undecylmercaptan | -8063.27 | -8056.50 | -8055.80 | -6.77 |
| 705 | 2-Methyl-1-pentene | -3988.80 | -3995.60 | -3994.50 | 6.80 |
| 706 | 3-Octanone | -5052.00 | -5058.80 | -5060.80 | 6.80 |
| 707 | Dipropyl disulfide | -5395.00 | -5401.80 | -5398.40 | 6.80 |
| 708 | cis-1,2-Dimethylcyclopropane | -3370.40 | -3377.20 | -3379.20 | 6.80 |
| 709 | o-Toluic acid | -3874.90 | -3881.70 | -3881.80 | 6.80 |
| 710 | Hippuric acid | -4221.30 | -4228.10 | -4227.40 | 6.80 |
| 711 | Cyclopropylaldimine | -2726.66 | -2719.80 | -2717.40 | -6.86 |
| 712 | 2,3-Dimethyl-2,3-diphenylbutane | -10188.00 | -10181.10 | -10183.10 | -6.90 |
| 713 | 1-Butanol | -2677.60 | -2670.70 | -2670.60 | -6.90 |
| 714 | 1,3,6-Trioxacyclooctane | -2880.70 | -2873.80 | -2867.50 | -6.90 |
| 715 | Ethylene | -1411.90 | -1405.00 | -1404.00 | -6.90 |
| 716 | 5-Methylisoxazole | -2283.00 | -2276.10 |  | -6.90 |
| 717 | Diethylsulfone | -3090.00 | -3096.90 | -3098.70 | 6.90 |
| 718 | 2,4-Xylenol | -4348.40 | -4341.40 | -4340.70 | -7.00 |
| 719 | 2-Amino-4,6-dimethoxypyrimidine | -3340.00 | -3333.00 |  | -7.00 |
| 720 | Diisobutylamine | -5638.10 | -5645.10 | -5644.80 | 7.00 |
| 721 | Glyceryl-2-laurate | -9043.60 | -9050.60 | -9049.40 | 7.00 |
| 722 | 2-Iodonaphthalene | -5082.09 | -5089.10 | -5088.90 | 7.01 |
| 723 | 3-Ethyl-2-methylpentane | -5474.79 | -5467.70 | -5467.00 | -7.09 |
| 724 | 1-Bromopropane | -2056.80 | -2049.70 | -2051.50 | -7.10 |
| 725 | 5-Acetamidotetrazole | -1891.20 | -1884.10 | -1882.00 | -7.10 |
| 726 | Cyclopropylamine | -2226.70 | -2233.80 | -2236.20 | 7.10 |
| 727 | Ethylnitrate | -1311.20 | -1318.30 | -1318.40 | 7.10 |
| 728 | Ethyl methyl sulfide | -2833.60 | -2840.70 | -2839.70 | 7.10 |
| 729 | Sebacic acid | -5425.00 | -5432.10 | -5432.40 | 7.10 |
| 730 | Ethylisopropylether | -3369.19 | -3376.30 | -3376.40 | 7.11 |
| 731 | cis-1,2-Dimethylcyclopentane | -4593.29 | -4600.40 | -4601.90 | 7.11 |
| 732 | Caproic aldehyde | -3786.93 | -3779.80 | -3778.90 | -7.13 |
| 733 | 5,7-Dodecadiyne | -7480.96 | -7488.10 | -7489.40 | 7.14 |
| 734 | Allyl alcohol | -1868.28 | -1861.10 | -1860.10 | -7.18 |
| 735 | Ethyl iodide | -1466.50 | -1459.30 | -1458.60 | -7.20 |
| 736 | N,N-Dimethylaniline | -4757.50 | -4750.30 | -4750.10 | -7.20 |
| 737 | Alanylglycine | -2621.70 | -2614.50 | -2612.90 | -7.20 |
| 738 | Diethyl sulfite | -3004.80 | -3012.00 | -3014.30 | 7.20 |
| 739 | Phthalic acid | -3225.70 | -3218.50 | -3216.20 | -7.20 |
| 740 | 3-Methyl-2-butanone | -3097.00 | -3104.20 | -3102.80 | 7.20 |
| 741 | Serylserine | -2898.90 | -2906.10 | -2903.30 | 7.20 |
| 742 | 13,14-Dihydroxybehenic acid | -13544.00 | -13551.20 | -13551.60 | 7.20 |
| 743 | Di-t-butyl ether | -5324.76 | -5332.00 | -5334.50 | 7.24 |
| 744 | s-Butylmethylether | -3379.75 | -3387.00 | -3387.80 | 7.25 |
| 745 | 3-Ethylhexane | -5473.96 | -5466.70 | -5467.30 | -7.26 |
| 746 | 2,2,3,3-Tetrafluoro-1-propanol | -1353.40 | -1346.10 | -1347.50 | -7.30 |
| 747 | Diethyleneglycol | -2374.70 | -2367.40 | -2369.50 | -7.30 |
| 748 | Propylenecarbonate | -1818.00 | -1810.70 | -1808.40 | -7.30 |
| 749 | Oxalyldihydrazide | -1369.70 | -1377.00 | -1382.40 | 7.30 |
| 750 | Ethyne | -1300.50 | -1307.80 | -1305.70 | 7.30 |
| 751 | Norcarane | -4433.00 | -4440.30 | -4441.60 | 7.30 |
| 752 | Dimethyl sulfide | -2181.50 | -2188.80 | -2189.50 | 7.30 |
| 753 | Ethyl methacrylate | -3371.40 | -3378.70 | -3380.00 | 7.30 |
| 754 | Xylitol | -2564.00 | -2571.30 | -2572.10 | 7.30 |
| 755 | Naphthalene | -5157.30 | -5164.60 | -5162.30 | 7.30 |
| 756 | 1,1-Dimethoxybutane | -3896.68 | -3904.00 | -3904.40 | 7.32 |
| 757 | 2,3-Dimethylbenzoic acid | -4523.66 | -4531.00 | -4531.70 | 7.34 |
| 758 | 1,4-DI-t-butylbenzene | -8470.15 | -8462.80 | -8462.30 | -7.35 |
| 759 | trans-1,2-Dimethylcyclopentane | -4587.35 | -4594.70 | -4593.60 | 7.35 |
| 760 | Nitromethane | -709.00 | -701.60 |  | -7.40 |
| 761 | Dimethyl succinate | -2957.10 | -2964.50 | -2965.20 | 7.40 |
| 762 | Norbornene | -4213.00 | -4220.40 | -4217.60 | 7.40 |
| 763 | Pentamethylbenzoic acid | -6477.34 | -6469.90 | -6469.60 | -7.44 |
| 764 | Cyclohexylformate | -3977.02 | -3984.50 | -3986.00 | 7.48 |
| 765 | 2,2,3,3-Tetramethylpentane | -6125.89 | -6118.40 | -6116.10 | -7.49 |
| 766 | 6,6-Dimethyl-1-phenyl-4,8-dioxaspiro[2.5]octane | -7784.61 | -7792.10 | -7790.60 | 7.49 |
| 767 | n-Octanoic acid | -4802.30 | -4794.80 | -4795.00 | -7.50 |
| 768 | Glyme | -2623.60 | -2631.10 | -2629.90 | 7.50 |
| 769 | Methylcyclobutane | -3354.49 | -3362.00 | -3362.20 | 7.51 |
| 770 | Linoleic acid | -10990.21 | -10982.70 | -10984.60 | -7.51 |
| 771 | 2,2-Dimethyl-1,3-dioxolane | -2968.48 | -2976.00 | -2978.80 | 7.52 |
| 772 | Urea | -632.08 | -639.60 | -642.00 | 7.52 |
| 773 | Propylcyclohexane | -5880.75 | -5873.20 | -5872.80 | -7.55 |
| 774 | Colamine | -1514.15 | -1521.70 | -1523.40 | 7.55 |
| 775 | Caprolactone | -3315.38 | -3307.80 | -3307.60 | -7.58 |
| 776 | cis-2-Pentene | -3343.20 | -3335.60 | -3336.20 | -7.60 |
| 777 | Cyclopentene | -3115.20 | -3107.60 | -3107.90 | -7.60 |
| 778 | Caprylene | -5312.90 | -5305.30 | -5306.00 | -7.60 |
| 779 | Di-n-propylsulfoxide | -4634.60 | -4642.20 | -4646.00 | 7.60 |
| 780 | Decylcyclopentane | -9829.60 | -9822.00 | -9822.40 | -7.60 |
| 781 | 3-Methyl-4-hydroxybenzaldehyde | -3828.00 | -3835.60 | -3834.80 | 7.60 |
| 782 | 3-Hydroxy-6-isopropyltoluene | -5644.38 | -5652.00 | -5652.50 | 7.62 |
| 783 | n-Propylacrylate | -3385.57 | -3393.20 | -3393.30 | 7.63 |
| 784 | Cuban | -4836.07 | -4843.70 | -4843.00 | 7.63 |
| 785 | Isobutylcyclohexane | -6534.24 | -6526.60 | -6526.40 | -7.64 |
| 786 | Propiolic acid | -1274.14 | -1266.50 | -1266.00 | -7.64 |
| 787 | 1,1-Diethylcyclohexane | -6520.83 | -6528.50 | -6527.90 | 7.67 |
| 788 | 2-Ethoxyethanol | -2556.98 | -2549.30 | -2549.20 | -7.68 |
| 789 | Formic acid | -254.81 | -262.50 | -262.90 | 7.69 |
| 790 | Tetraphenylmethane | -12943.00 | -12935.30 | -12936.60 | -7.70 |
| 791 | Cyclohexane | -3922.50 | -3914.80 | -3914.50 | -7.70 |
| 792 | trans-3-Heptene | -4646.00 | -4638.30 | -4638.10 | -7.70 |
| 793 | Acetic anhydride | -1794.20 | -1786.50 | -1786.10 | -7.70 |
| 794 | 1-Ethylcyclopentene | -4411.20 | -4403.50 | -4403.20 | -7.70 |
| 795 | Octane | -5470.20 | -5462.50 | -5461.60 | -7.70 |
| 796 | Dibenzoylperoxide | -6568.70 | -6576.40 | -6584.80 | 7.70 |
| 797 | Diethyl phthalate | -5950.82 | -5943.10 | -5943.50 | -7.72 |
| 798 | N-Methylformamide | -1261.12 | -1253.40 | -1255.50 | -7.72 |
| 799 | Isopropylthioacetate | -3703.15 | -3710.90 | -3715.80 | 7.75 |
| 800 | 5-Azabicyclo[2.1.0]pentane | -2801.76 | -2794.00 | -2791.50 | -7.76 |
| 801 | Indole | -4235.13 | -4242.90 | -4240.80 | 7.77 |
| 802 | Diisobutyl phthalate | -8556.60 | -8548.80 | -8549.40 | -7.80 |
| 803 | 1-Iodonaphthalene | -5096.90 | -5089.10 | -5087.00 | -7.80 |
| 804 | 2-Methyl-1,2-propanediamine | -3157.20 | -3149.40 | -3148.70 | -7.80 |
| 805 | 1,1,2,2-Tetrafluoroethane | -875.70 | -867.90 | -861.80 | -7.80 |
| 806 | Butyl butanoate | -4844.20 | -4852.00 | -4853.20 | 7.80 |
| 807 | Methylcaproate | -4218.00 | -4210.20 | -4209.70 | -7.80 |
| 808 | Methylglyoxime | -1915.00 | -1922.80 | -1923.70 | 7.80 |
| 809 | (Iodomethyl)benzene | -3809.47 | -3817.30 | -3818.30 | 7.83 |
| 810 | 3-Methyloctane | -6125.64 | -6117.80 | -6117.80 | -7.84 |
| 811 | 2-Norbornanonedimethylketal | -5400.92 | -5408.80 | -5403.40 | 7.88 |
| 812 | 2-Methylhexanal | -4441.09 | -4433.20 | -4435.10 | -7.89 |
| 813 | t-Butylformamide | -3201.00 | -3193.10 | -3192.90 | -7.90 |
| 814 | 1-Chlorobutane | -2704.10 | -2696.20 | -2696.20 | -7.90 |
| 815 | 1,2,3-Trichloropropene | -1578.40 | -1570.50 | -1568.90 | -7.90 |
| 816 | N,N-Diethylurea | -3310.30 | -3318.20 | -3316.70 | 7.90 |
| 817 | epsilon-Aminocaproic acid | -3582.20 | -3590.10 | -3592.50 | 7.90 |
| 818 | 1-Methylnaphthalene | -5805.88 | -5813.80 | -5815.30 | 7.92 |
| 819 | 2-Methyloctane | -6123.57 | -6115.60 | -6116.20 | -7.97 |
| 820 | 2-Vinylfuran | -3210.20 | -3218.20 | -3216.30 | 8.00 |
| 821 | Leucine anhydride | -7201.70 | -7209.70 | -7211.50 | 8.00 |
| 822 | 3-Cyclohexene-1-carboxaldehyde | -4024.41 | -4016.40 | -4014.80 | -8.01 |
| 823 | 2-Methyl-2-pentenal | -3590.94 | -3599.00 | -3598.20 | 8.06 |
| 824 | Tetraethyleneglycol | -4742.67 | -4734.60 | -4732.80 | -8.07 |
| 825 | 1,2-Diethylbenzene | -5871.39 | -5863.30 | -5862.50 | -8.09 |
| 826 | Decylcyclohexane | -10451.00 | -10442.90 | -10443.30 | -8.10 |
| 827 | 2,2,4,4-Tetramethylpentane | -6117.80 | -6109.70 | -6111.50 | -8.10 |
| 828 | DMSO | -2037.30 | -2029.20 | -2026.60 | -8.10 |
| 829 | Benzonitrile | -3632.30 | -3640.40 | -3639.90 | 8.10 |
| 830 | 2-Phenyl-1,3-dioxolane | -4703.30 | -4695.20 | -4694.40 | -8.10 |
| 831 | N-Methyl-2-pyrrolidone | -2993.70 | -3001.80 | -2999.70 | 8.10 |
| 832 | 2-Methoxypyridine | -3275.91 | -3267.80 |  | -8.11 |
| 833 | Tetrahydrofuran | -2505.40 | -2497.20 | -2495.40 | -8.20 |
| 834 | Cyclohexylbenzene | -6936.84 | -6928.60 | -6927.80 | -8.24 |
| 835 | 4-Iodobenzoic acid | -3154.20 | -3162.50 | -3162.30 | 8.30 |
| 836 | 2,5-Dihydrothiophene | -3081.10 | -3089.40 | -3090.10 | 8.30 |
| 837 | 2-Nitropropane | -1998.80 | -2007.10 | -2009.00 | 8.30 |
| 838 | 1,1-Dimethylcyclopropane | -3363.40 | -3371.70 | -3373.70 | 8.30 |
| 839 | N,N'-Di-t-butylethylenediamine | -7105.08 | -7113.40 | -7115.10 | 8.32 |
| 840 | o-Anthranilic acid | -3353.83 | -3345.50 | -3347.70 | -8.33 |
| 841 | n-Heptylformate | -4895.37 | -4887.00 | -4885.80 | -8.37 |
| 842 | Phenyldiethoxymethane | -6246.87 | -6238.50 | -6237.70 | -8.37 |
| 843 | Tetrafluoroethylene | -823.52 | -831.90 | -802.50 | 8.38 |
| 844 | Ethoxybenzene | -4427.69 | -4419.30 | -4420.10 | -8.39 |
| 845 | 6-Undecanone | -7024.60 | -7016.20 | -7015.50 | -8.40 |
| 846 | Isobutylamine | -3015.50 | -3007.10 | -3004.20 | -8.40 |
| 847 | Hexane | -4165.90 | -4157.50 | -4157.40 | -8.40 |
| 848 | 5-Methylaminotetrazole | -1704.90 | -1696.50 | -1691.50 | -8.40 |
| 849 | 2,5-Dinitroaniline | -3031.00 | -3039.40 | -3038.30 | 8.40 |
| 850 | Phthalamide | -3857.30 | -3865.70 | -3866.00 | 8.40 |
| 851 | Styrene | -4395.00 | -4403.40 | -4403.40 | 8.40 |
| 852 | Lauric peroxyacid | -7476.80 | -7485.20 | -7481.30 | 8.40 |
| 853 | n-Hexylacetate | -4860.45 | -4852.00 | -4849.90 | -8.45 |
| 854 | Di-n-propylamine | -4351.69 | -4343.20 | -4344.50 | -8.49 |
| 855 | 2,3-Dichloropropene | -1726.20 | -1717.70 | -1715.50 | -8.50 |
| 856 | 3-Mercaptopropyl-triethoxysilane | -7086.40 | -7077.90 | -7076.50 | -8.50 |
| 857 | Hydroquinone | -2854.50 | -2846.00 | -2846.80 | -8.50 |
| 858 | Ethylurea | -1966.10 | -1974.60 | -1974.60 | 8.50 |
| 859 | Lauric acid t-butylperoxyester | -10138.00 | -10129.40 | -10126.80 | -8.60 |
| 860 | alpha-Chlorohydrin | -1679.00 | -1670.40 | -1670.40 | -8.60 |
| 861 | Ethyl cyanoacetate | -2638.00 | -2629.40 | -2628.10 | -8.60 |
| 862 | 8-Hydroxyquinoline | -4461.90 | -4470.50 | -4472.00 | 8.60 |
| 863 | Ethyl acetate | -2237.90 | -2246.50 | -2246.60 | 8.60 |
| 864 | Cyclopropylbenzene | -5076.40 | -5085.00 | -5083.50 | 8.60 |
| 865 | 4-Nonanone | -5719.85 | -5711.20 | -5710.00 | -8.65 |
| 866 | Allylbenzene | -5062.09 | -5053.40 | -5053.80 | -8.69 |
| 867 | Refrigerant 115 | -555.29 | -546.60 | -548.10 | -8.69 |
| 868 | m-Cresol | -3706.40 | -3697.70 | -3698.90 | -8.70 |
| 869 | 3-Iodobenzoic acid | -3153.80 | -3162.50 | -3163.20 | 8.70 |
| 870 | Diethyl sulfate | -2792.30 | -2801.00 | -2804.80 | 8.70 |
| 871 | 2-Octanone | -5050.10 | -5058.80 | -5059.10 | 8.70 |
| 872 | trans-1,2-Dimethylcyclopropane | -3366.00 | -3374.70 | -3375.90 | 8.70 |
| 873 | Crotonaldehyde | -2294.45 | -2303.20 | -2301.50 | 8.75 |
| 874 | Thymol | -5657.00 | -5648.20 | -5647.90 | -8.80 |
| 875 | Cyclohexene | -3751.90 | -3743.10 | -3742.50 | -8.80 |
| 876 | 1,3-Diiodobenzene | -3119.60 | -3110.80 | -3108.60 | -8.80 |
| 877 | Dibenzo[b,e]pyridine | -6593.80 | -6585.00 | -6584.70 | -8.80 |
| 878 | Benzoic acid | -3229.20 | -3238.00 | -3237.80 | 8.80 |
| 879 | Pentaerythritol | -2763.70 | -2772.50 | -2773.60 | 8.80 |
| 880 | Glyceraldehyde | -1440.30 | -1449.10 | -1450.40 | 8.80 |
| 881 | Hexachlorocyclopentadiene | -1944.55 | -1953.40 | -1995.20 | 8.85 |
| 882 | Hexachloroethane | -723.81 | -732.70 | -733.30 | 8.89 |
| 883 | Edetic acid | -4465.20 | -4456.30 |  | -8.90 |
| 884 | Biuret | -938.00 | -946.90 | -945.40 | 8.90 |
| 885 | Fluoranthene | -7915.20 | -7906.20 | -7907.20 | -9.00 |
| 886 | 1-Pentanol | -3332.20 | -3323.20 | -3322.70 | -9.00 |
| 887 | DTPA | -6575.40 | -6584.40 |  | 9.00 |
| 888 | Dicyclohexyl | -7597.81 | -7588.80 | -7590.80 | -9.01 |
| 889 | 4-Methylnonane | -6779.86 | -6770.80 | -6772.10 | -9.06 |
| 890 | 2,2,3,3-Tetramethylhexane | -6780.49 | -6771.40 | -6773.00 | -9.09 |
| 891 | Vinyl acetate | -2086.00 | -2076.90 | -2076.50 | -9.10 |
| 892 | Triphenylmethane | -9934.10 | -9925.00 | -9925.20 | -9.10 |
| 893 | Ethylene-1,2-diamine | -1868.60 | -1859.50 | -1859.10 | -9.10 |
| 894 | Diisopropyl ether | -4010.40 | -4019.50 | -4018.60 | 9.10 |
| 895 | 3-Iodopropanoic acid | -1435.10 | -1444.20 | -1444.90 | 9.10 |
| 896 | Phenylalanine | -4646.30 | -4637.20 | -4637.50 | -9.10 |
| 897 | 1,2-Dibromotetrafluoroethane | -631.79 | -640.90 | -663.40 | 9.11 |
| 898 | Vinyltrimethoxysilane | -3696.92 | -3687.80 |  | -9.12 |
| 899 | Oxamic acid | -545.12 | -536.00 | -536.30 | -9.12 |
| 900 | 2-Phenyl-2,5,5-trimethyl-1,3-dioxane | -7278.74 | -7269.60 | -7270.00 | -9.14 |
| 901 | 3,9-Dodecadiyne | -7497.28 | -7488.10 | -7485.60 | -9.18 |
| 902 | 1,2-Diiodobenzene | -3120.00 | -3110.80 | -3109.30 | -9.20 |
| 903 | Benzamide | -3554.80 | -3564.00 | -3563.30 | 9.20 |
| 904 | Methylenecyclohexane | -4412.40 | -4403.20 | -4404.00 | -9.20 |
| 905 | Nitropenta | -2572.40 | -2581.60 | -2597.30 | 9.20 |
| 906 | Caprylonitrile | -5188.04 | -5197.30 | -5196.30 | 9.26 |
| 907 | 1-Decene | -6619.60 | -6610.30 | -6610.20 | -9.30 |
| 908 | Methylpelargonate | -6181.30 | -6172.00 | -6172.70 | -9.30 |
| 909 | 2-Nitrobutane | -2652.70 | -2662.00 | -2662.20 | 9.30 |
| 910 | Methoxyacetic acid | -1412.34 | -1421.70 | -1421.80 | 9.36 |
| 911 | Tetrazole | -916.91 | -926.30 | -926.80 | 9.39 |
| 912 | Ethyleneglycol diacetate | -2927.31 | -2936.70 | -2938.50 | 9.39 |
| 913 | n-Octylacetate | -6169.69 | -6160.30 | -6158.30 | -9.39 |
| 914 | Glycerol | -1654.30 | -1644.90 | -1645.20 | -9.40 |
| 915 | NTA | -2341.30 | -2331.90 |  | -9.40 |
| 916 | Propionic acid methylester | -2245.60 | -2255.00 | -2255.30 | 9.40 |
| 917 | Arabitol | -2559.80 | -2569.20 | -2569.20 | 9.40 |
| 918 | 3-Ethylheptane | -6128.69 | -6119.20 | -6119.30 | -9.49 |
| 919 | 1-Heptanol | -4637.60 | -4628.10 | -4627.70 | -9.50 |
| 920 | 2,4,6-Trinitrotoluene | -3420.20 | -3410.70 | -3407.00 | -9.50 |
| 921 | Alanine anhydride | -3300.00 | -3290.50 | -3288.80 | -9.50 |
| 922 | Furan | -2085.00 | -2094.50 | -2093.10 | 9.50 |
| 923 | 5,5'-Hydrazotetrazole | -1887.40 | -1896.90 | -1892.70 | 9.50 |
| 924 | Isoleucine | -3578.30 | -3587.80 | -3590.60 | 9.50 |
| 925 | n-Butyl-3-chlorobutanoate | -4707.02 | -4697.50 | -4696.00 | -9.52 |
| 926 | 1,3,5-Cycloheptatriene | -4042.66 | -4052.20 | -4053.10 | 9.54 |
| 927 | 3-Nonanone | -5720.75 | -5711.20 | -5711.50 | -9.55 |
| 928 | 3-Methylnonane | -6780.37 | -6770.80 | -6772.10 | -9.57 |
| 929 | 2-Cyclohexylcyclohexanone | -7194.50 | -7184.90 | -7182.80 | -9.60 |
| 930 | 1-Hexanol | -3985.20 | -3975.60 | -3976.30 | -9.60 |
| 931 | Maleic acid | -1355.20 | -1345.60 | -1346.10 | -9.60 |
| 932 | Furfurylalcohol | -2550.60 | -2541.00 | -2537.90 | -9.60 |
| 933 | Nonane | -6124.60 | -6115.00 | -6115.70 | -9.60 |
| 934 | 1,2-Dibromoethane | -1237.20 | -1246.80 | -1246.10 | 9.60 |
| 935 | Ethanethiol | -2174.60 | -2184.20 | -2184.90 | 9.60 |
| 936 | Perfluoroheptanoic acid | -1941.65 | -1951.30 | -1951.40 | 9.65 |
| 937 | cis-2-Octene | -5302.66 | -5293.00 | -5292.30 | -9.66 |
| 938 | trans-2-Octene | -5302.66 | -5293.00 | -5292.30 | -9.66 |
| 939 | 1-Hexadecanol | -10510.00 | -10500.30 | -10499.30 | -9.70 |
| 940 | Methylenecyclobutane | -3204.70 | -3195.00 | -3196.20 | -9.70 |
| 941 | Isopropanolamine | -2174.31 | -2164.60 | -2164.10 | -9.71 |
| 942 | 2-Methyloctanal | -5747.95 | -5738.20 | -5737.00 | -9.75 |
| 943 | 1,1,1,2-Tetrachloroethane | -973.90 | -983.70 | -983.40 | 9.80 |
| 944 | 4-Iodotoluene | -3822.50 | -3832.30 | -3832.90 | 9.80 |
| 945 | Di-n-propylsulfone | -4416.00 | -4406.20 | -4405.00 | -9.80 |
| 946 | Thiane | -3892.50 | -3902.30 | -3903.90 | 9.80 |
| 947 | Ethyleneglycoldiacrylate | -3917.48 | -3927.30 | -3927.00 | 9.82 |
| 948 | Cyclohexylmercaptan | -4540.45 | -4550.30 | -4550.60 | 9.85 |
| 949 | n-Dodecylmercaptan | -8718.75 | -8708.90 | -8708.80 | -9.85 |
| 950 | Pentamethylbenzene | -6486.03 | -6495.90 | -6495.90 | 9.87 |
| 951 | 2-Butylacetate | -3534.72 | -3544.60 | -3544.60 | 9.88 |
| 952 | 1,1-Dimethoxypropane | -3241.69 | -3251.60 | -3252.10 | 9.91 |
| 953 | n-Dodecylamine | -8218.38 | -8228.30 | -8227.90 | 9.92 |
| 954 | Dichloroacetaldehyde | -900.65 | -890.70 | -898.60 | -9.95 |
| 955 | 3-Methylhexanal | -4443.17 | -4433.20 | -4431.80 | -9.97 |
| 956 | Dimethoxane | -4278.63 | -4288.60 | -4288.30 | 9.97 |
| 957 | Cyclopentylacetic acid | -3920.81 | -3930.80 | -3931.50 | 9.99 |
| 958 | 1-Butyne | -2596.60 | -2586.60 | -2585.30 | -10.00 |
| 959 | 2,4-Dimethyl-3-ethylpentane | -6130.10 | -6120.10 | -6119.00 | -10.00 |
| 960 | Methylnitrate | -666.50 | -676.50 | -677.50 | 10.00 |
| 961 | Hydrocinnamyl alcohol | -5042.18 | -5032.10 | -5031.50 | -10.08 |
| 962 | Valylphenylalanine | -7604.50 | -7594.40 | -7591.40 | -10.10 |
| 963 | 1-Naphthylamine | -5285.90 | -5275.80 | -5278.70 | -10.10 |
| 964 | Decanenitrile | -6492.10 | -6502.20 | -6501.80 | 10.10 |
| 965 | Methylcaprylate | -5527.50 | -5517.30 | -5517.80 | -10.20 |
| 966 | Arabinose | -2340.40 | -2350.60 | -2346.60 | 10.20 |
| 967 | Isopropylnitrate | -1951.00 | -1961.20 | -1964.80 | 10.20 |
| 968 | D(+)-Sorbose | -2807.70 | -2817.90 | -2817.40 | 10.20 |
| 969 | AIBN | -5109.10 | -5119.30 | -5114.90 | 10.20 |
| 970 | GBL | -2010.60 | -2020.80 | -2021.60 | 10.20 |
| 971 | trans-2-Hexene | -3996.16 | -3985.90 | -3986.40 | -10.26 |
| 972 | Bis(2-chloroethyl) ether | -2408.13 | -2418.40 | -2416.80 | 10.27 |
| 973 | Cellobiose | -5642.10 | -5631.80 | -5629.30 | -10.30 |
| 974 | Formic acid methylester | -972.50 | -982.80 | -982.60 | 10.30 |
| 975 | 2,4-Xylidine | -4655.50 | -4665.80 | -4667.10 | 10.30 |
| 976 | Di(2-ethylhexyl)amine | -10889.83 | -10879.50 | -10881.40 | -10.33 |
| 977 | Methyl-t-pentylsulfide | -4787.18 | -4776.80 | -4777.90 | -10.38 |
| 978 | Mercaptoacetic acid | -1507.41 | -1497.00 | -1497.50 | -10.41 |
| 979 | 4-tert-Pentylphenol | -6318.82 | -6308.40 | -6307.70 | -10.42 |
| 980 | 2,6-Dimethyl-4-heptanol | -5934.63 | -5924.20 | -5925.00 | -10.43 |
| 981 | 2-Fluoropropane | -2062.53 | -2052.10 |  | -10.43 |
| 982 | 4-Methoxyphenylacetic acid | -4395.66 | -4406.10 | -4406.30 | 10.44 |
| 983 | Quinaldine | -5316.49 | -5306.00 | -5308.90 | -10.49 |
| 984 | 1-Chlorododecane | -7926.40 | -7915.90 | -7916.40 | -10.50 |
| 985 | Decane | -6777.90 | -6767.40 | -6767.80 | -10.50 |
| 986 | Chloroacetyl chloride | -833.72 | -823.20 | -821.20 | -10.52 |
| 987 | 2-Methyl-2-butanethiol | -4124.57 | -4135.10 | -4135.90 | 10.53 |
| 988 | Azoisopropane | -4400.58 | -4390.00 | -4385.60 | -10.58 |
| 989 | Di-n-hexylether | -7961.59 | -7951.00 | -7951.30 | -10.59 |
| 990 | Methanethiol | -1521.80 | -1532.40 | -1533.60 | 10.60 |
| 991 | 1,1-Diphenylethylene | -7396.50 | -7407.10 | -7409.70 | 10.60 |
| 992 | 2,3-Dimethyl-1,3-butadiene | -3815.00 | -3825.60 | -3829.00 | 10.60 |
| 993 | Benzo[c]phenanthrene | -8983.00 | -8972.40 | -8964.20 | -10.60 |
| 994 | trans-1,3-Pentadiene | -3189.05 | -3178.40 | -3176.20 | -10.65 |
| 995 | sec-Phenethyl alcohol | -4382.86 | -4372.20 | -4372.50 | -10.66 |
| 996 | Norbornane | -4377.44 | -4388.10 | -4389.60 | 10.66 |
| 997 | 1-Dodecene | -7925.90 | -7915.20 | -7915.50 | -10.70 |
| 998 | 2-Methyl-2-butanamine | -3656.60 | -3645.90 | -3646.30 | -10.70 |
| 999 | Pelargonic acid | -5458.00 | -5447.30 | -5446.90 | -10.70 |
| 1000 | Guanidine | -1052.10 | -1041.40 | -1036.70 | -10.70 |
| 1001 | 1-Propylamine | -2366.80 | -2356.10 | -2355.90 | -10.70 |
| 1002 | Acetol | -1625.23 | -1614.50 | -1613.10 | -10.73 |
| 1003 | 2,3-Dimethyl-1-butene | -3983.66 | -3994.40 | -3995.50 | 10.74 |
| 1004 | 2,3,4-Trimethylbenzoic acid | -5167.25 | -5178.00 | -5176.80 | 10.75 |
| 1005 | Isopropylacrylate | -3373.34 | -3384.10 | -3383.90 | 10.76 |
| 1006 | Ethyl vinyl ether | -2540.00 | -2529.20 | -2526.90 | -10.80 |
| 1007 | 2,3-Dimethyl-2,3-dinitrobutane | -3734.60 | -3723.80 | -3717.20 | -10.80 |
| 1008 | 4-Hydroxystyrene | -4184.60 | -4195.40 | -4197.00 | 10.80 |
| 1009 | 2,2-Dichloro-1,1,2-trifluoroethane | -768.23 | -757.40 | -752.30 | -10.83 |
| 1010 | Ethylamine | -1714.50 | -1703.60 | -1703.80 | -10.90 |
| 1011 | Fumaric acid | -1334.70 | -1345.60 | -1347.20 | 10.90 |
| 1012 | 2-Methyl-1-hexanol | -4640.08 | -4629.10 | -4629.70 | -10.98 |
| 1013 | Undecane | -7430.90 | -7419.90 | -7419.30 | -11.00 |
| 1014 | 4-Aminoacetophenone | -4252.20 | -4263.20 | -4263.50 | 11.00 |
| 1015 | 1,2,5,6-Tetrahydropyridine | -3287.30 | -3276.30 | -3276.10 | -11.00 |
| 1016 | 2-Ethyl-2-nitro-1,3-propanediol | -2933.90 | -2922.90 | -2918.60 | -11.00 |
| 1017 | 2,6-Dimethoxypyridine | -3756.10 | -3767.10 |  | 11.00 |
| 1018 | Diethyl succinate | -4239.26 | -4250.30 | -4249.90 | 11.04 |
| 1019 | Diethylcarbonate | -2715.20 | -2704.10 | -2710.30 | -11.10 |
| 1020 | 1-Propanethiol | -2828.20 | -2817.10 | -2815.60 | -11.10 |
| 1021 | Dimethyl malonate | -2316.60 | -2305.50 | -2304.30 | -11.10 |
| 1022 | N-Methylcaprolactam | -4308.60 | -4297.50 | -4298.50 | -11.10 |
| 1023 | Pentadecylbenzene | -13032.37 | -13043.50 | -13043.00 | 11.13 |
| 1024 | Amylbenzene | -6530.17 | -6519.00 | -6517.90 | -11.17 |
| 1025 | Coproporphyrin I tetramethylester | -20871.00 | -20859.80 | -20856.40 | -11.20 |
| 1026 | (L)-Methionine | -3564.10 | -3552.90 | -3552.20 | -11.20 |
| 1027 | 2-Iodobenzoic acid | -3173.70 | -3162.50 | -3161.00 | -11.20 |
| 1028 | N-Formylleucine | -3841.60 | -3852.80 | -3854.00 | 11.20 |
| 1029 | Crotonic acid | -2002.10 | -2013.30 | -2013.20 | 11.20 |
| 1030 | Di-n-butylsulfate | -5422.16 | -5410.90 | -5403.80 | -11.26 |
| 1031 | Saccharose | -5644.60 | -5633.30 | -5633.80 | -11.30 |
| 1032 | Di(2-pyridyl)ketone | -4668.30 | -4657.00 | -4643.70 | -11.30 |
| 1033 | Nitromesitylene | -5043.80 | -5032.50 | -5033.80 | -11.30 |
| 1034 | Cyclobutane | -2722.30 | -2711.00 | -2710.80 | -11.30 |
| 1035 | Tetrahydrofurfuryl alcohol | -2963.00 | -2974.30 | -2975.00 | 11.30 |
| 1036 | 1-Nonene | -5969.12 | -5957.80 | -5958.80 | -11.32 |
| 1037 | Diphenylamine | -6424.00 | -6412.60 | -6410.20 | -11.40 |
| 1038 | Nitromethylbenzene | -3732.10 | -3720.70 | -3719.60 | -11.40 |
| 1039 | Valine | -2921.50 | -2932.90 | -2935.80 | 11.40 |
| 1040 | 4-Chloroaniline | -3208.80 | -3220.20 | -3220.90 | 11.40 |
| 1041 | Di-(2-chloroethoxy)methane | -2907.92 | -2919.40 | -2920.90 | 11.48 |
| 1042 | Butylsilane | -4027.70 | -4016.20 |  | -11.50 |
| 1043 | 1-Nitro-2-propanone | -1600.00 | -1611.50 | -1610.70 | 11.50 |
| 1044 | D-Galactose | -2805.60 | -2817.10 | -2818.30 | 11.50 |
| 1045 | 2-Butylmercaptan | -3476.58 | -3488.10 | -3489.10 | 11.52 |
| 1046 | Ethylenedichloride | -1246.40 | -1234.80 | -1233.60 | -11.60 |
| 1047 | 2-Azidoethanol | -1597.20 | -1585.60 | -1579.00 | -11.60 |
| 1048 | Dimethyl carbonate | -1432.11 | -1420.50 | -1418.00 | -11.61 |
| 1049 | 1,1,1-Trichlorofluoroethane | -977.83 | -989.50 | -997.80 | 11.67 |
| 1050 | Ethyl-t-pentylether | -4675.51 | -4687.20 | -4687.30 | 11.69 |
| 1051 | Maleic anhydride | -1389.50 | -1377.80 | -1378.00 | -11.70 |
| 1052 | Diphenylmethane | -6927.70 | -6916.00 | -6916.10 | -11.70 |
| 1053 | N,N'-Di(2,4,6-trinitrophenyl)ethylene-1,2-dinitroamine | -6799.40 | -6811.10 |  | 11.70 |
| 1054 | 3-Hydroxy-5-isopropyltoluene | -5639.88 | -5651.60 | -5651.30 | 11.72 |
| 1055 | 2-Hydroxypropylacrylate | -3210.24 | -3198.50 | -3198.80 | -11.74 |
| 1056 | 3-Methyl-1-butyne | -3249.35 | -3237.60 | -3237.60 | -11.75 |
| 1057 | 1-Tetradecylamine | -9521.45 | -9533.20 | -9532.90 | 11.75 |
| 1058 | Diamylamine | -6964.76 | -6953.00 | -6953.00 | -11.76 |
| 1059 | Diethyleneglycol-di-n-butylether | -7719.97 | -7708.20 | -7706.00 | -11.77 |
| 1060 | 1,3-Dibromo-2-methylpropane | -2538.39 | -2550.20 | -2547.90 | 11.81 |
| 1061 | 1-Methyl-4-isopropylcyclohexene | -6357.42 | -6345.60 | -6347.70 | -11.82 |
| 1062 | Di-n-butylsulfite | -5633.66 | -5621.80 |  | -11.86 |
| 1063 | Phenylacetone | -4818.80 | -4806.90 | -4805.90 | -11.90 |
| 1064 | cis-2-Heptene | -4650.20 | -4638.30 | -4638.10 | -11.90 |
| 1065 | 1,1-Diphenylethane | -7558.60 | -7570.50 | -7571.30 | 11.90 |
| 1066 | 1-Butylamine | -3020.40 | -3008.50 | -3008.40 | -11.90 |
| 1067 | 5-Phenylaminotetrazole | -4062.90 | -4051.00 | -4052.40 | -11.90 |
| 1068 | N-Methylcyclohexylamine | -4752.05 | -4764.00 | -4765.90 | 11.95 |
| 1069 | Isoquinoline | -4686.46 | -4674.50 | -4673.40 | -11.96 |
| 1070 | Bis(2-hydroxyethyl)methylamine | -3354.23 | -3366.20 | -3367.90 | 11.97 |
| 1071 | 2-Methyl-1,3-propanediol | -2499.30 | -2487.30 | -2488.30 | -12.00 |
| 1072 | trans-3-Hexene | -3997.96 | -3985.90 | -3986.10 | -12.06 |
| 1073 | Trioxan | -1515.30 | -1503.20 | -1486.30 | -12.10 |
| 1074 | Thiohydantoin | -2106.80 | -2118.90 | -2123.30 | 12.10 |
| 1075 | 1,4-Dihydronaphthalene | -5452.03 | -5439.90 | -5440.30 | -12.13 |
| 1076 | Glutaric anhydride | -2208.43 | -2196.30 | -2197.80 | -12.13 |
| 1077 | 2,2-Dichloro-1,1,1-trifluoroethane | -726.31 | -738.50 | -740.90 | 12.19 |
| 1078 | n-Octylformate | -5551.70 | -5539.50 | -5538.30 | -12.20 |
| 1079 | Methyl methacrylate | -2724.60 | -2736.80 | -2736.70 | 12.20 |
| 1080 | 2,4,6-Trinitrophenol | -2570.00 | -2557.70 | -2557.40 | -12.30 |
| 1081 | o-Dinitrobenzene | -2944.20 | -2931.90 | -2932.90 | -12.30 |
| 1082 | Tryptophane | -5628.30 | -5616.00 | -5622.40 | -12.30 |
| 1083 | 1,1-Diethoxyethane | -3870.50 | -3882.80 | -3881.30 | 12.30 |
| 1084 | Methyl-4-formylbenzoate | -4211.94 | -4224.30 | -4224.20 | 12.36 |
| 1085 | Dibenzylsulfone | -7829.60 | -7817.20 | -7817.90 | -12.40 |
| 1086 | 1,5-Pentanediol | -3153.70 | -3141.30 | -3141.80 | -12.40 |
| 1087 | Pyridine | -2784.20 | -2771.80 | -2768.90 | -12.40 |
| 1088 | 1,2-Diphenylethane | -7562.60 | -7575.00 | -7574.90 | 12.40 |
| 1089 | Dimethyl fumarate | -2777.10 | -2789.50 | -2788.90 | 12.40 |
| 1090 | Acetylchloride | -967.15 | -979.60 | -982.20 | 12.45 |
| 1091 | Pyrrolidine | -2816.70 | -2829.20 | -2826.80 | 12.50 |
| 1092 | 1-(4-Ethylphenyl)-2-phenylethane | -8886.58 | -8874.00 | -8873.90 | -12.58 |
| 1093 | Ethylcyclopentane | -4591.90 | -4579.30 | -4579.20 | -12.60 |
| 1094 | Cyclopentylamine | -3444.50 | -3457.10 | -3456.30 | 12.60 |
| 1095 | cis-1,2-Dichloroethylene | -1123.32 | -1110.70 |  | -12.62 |
| 1096 | 2,2-Diphenyl-dimethoxymethane | -7924.16 | -7936.80 | -7939.40 | 12.64 |
| 1097 | 3-Methylundecane | -8088.35 | -8075.70 | -8074.80 | -12.65 |
| 1098 | Cyclooctatetraene | -4545.70 | -4533.00 | -4538.70 | -12.70 |
| 1099 | 2-Methyl-2-nitro-3-phenyl-1-propanol | -5449.10 | -5461.80 | -5462.00 | 12.70 |
| 1100 | 2-Chlorobutane | -2685.40 | -2698.10 | -2697.10 | 12.70 |
| 1101 | 1,1,2-Triphenylethane | -10572.00 | -10584.70 | -10587.10 | 12.70 |
| 1102 | 2,2,3,3-Tetramethylbutane | -5451.50 | -5464.20 | -5464.00 | 12.70 |
| 1103 | 2,3-Dibromobutane | -2536.39 | -2549.10 | -2541.40 | 12.71 |
| 1104 | Ethyl oleate | -12525.00 | -12512.20 | -12511.40 | -12.80 |
| 1105 | Enantholactam | -4267.20 | -4254.40 | -4255.80 | -12.80 |
| 1106 | cis-4,4-Dimethyl-2-pentene | -4650.10 | -4637.30 | -4637.40 | -12.80 |
| 1107 | Butyramide | -2495.80 | -2508.60 | -2507.60 | 12.80 |
| 1108 | 1,2-Difluoroethane | -1259.15 | -1246.30 | -1238.80 | -12.85 |
| 1109 | 1-Bromo-2-chloro-1,1,2-trifluoroethane | -806.30 | -793.40 | -788.50 | -12.90 |
| 1110 | Propylene | -2058.10 | -2045.20 | -2044.10 | -12.90 |
| 1111 | 3-Nitrobenzaldehyde | -3350.00 | -3362.90 | -3363.50 | 12.90 |
| 1112 | 2-Methyl-2-phenyl-1,3-dioxolane | -5306.30 | -5319.20 | -5319.80 | 12.90 |
| 1113 | 2-trans-Decene | -6610.84 | -6597.90 | -6597.80 | -12.94 |
| 1114 | trans-4-Octene | -5303.74 | -5290.80 | -5290.70 | -12.94 |
| 1115 | Ibuprofen | -7133.71 | -7146.70 | -7148.70 | 12.99 |
| 1116 | 1-Octanol | -5293.60 | -5280.60 | -5281.00 | -13.00 |
| 1117 | Glyceryl-1-laurate | -9035.20 | -9048.20 | -9046.30 | 13.00 |
| 1118 | n-Nonanal | -5750.22 | -5737.20 | -5738.30 | -13.02 |
| 1119 | Bis(2-ethylhexyl) adipate | -13404.14 | -13391.10 | -13390.50 | -13.04 |
| 1120 | 2-Vinylaziridine | -2775.16 | -2762.10 | -2764.30 | -13.06 |
| 1121 | n-Decylacetate | -7478.28 | -7465.20 | -7463.20 | -13.08 |
| 1122 | Butylcellosolve | -3867.38 | -3854.30 | -3854.10 | -13.08 |
| 1123 | Tetramethylene glycol | -2501.90 | -2488.80 | -2488.90 | -13.10 |
| 1124 | Tetracene | -8956.90 | -8970.00 | -8971.70 | 13.10 |
| 1125 | N,N-Dimethylurea | -2004.80 | -2017.90 | -2017.00 | 13.10 |
| 1126 | N-Methylurea | -1311.70 | -1324.80 | -1325.60 | 13.10 |
| 1127 | 2,4,6-Trinitroaniline | -2860.10 | -2873.20 | -2874.70 | 13.10 |
| 1128 | 3,5-Dimethylisoxazole | -2905.00 | -2918.10 |  | 13.10 |
| 1129 | t-Butylmercaptan | -3467.08 | -3480.20 | -3481.80 | 13.12 |
| 1130 | 1-Phenyl-4,7-dioxaspiro[2.4]heptane | -5865.03 | -5851.90 | -5854.10 | -13.13 |
| 1131 | trans-3-Octene | -5303.97 | -5290.80 | -5291.20 | -13.17 |
| 1132 | 1-Nonanol | -5946.30 | -5933.10 | -5932.80 | -13.20 |
| 1133 | Methylsalicylate | -3762.30 | -3749.10 | -3748.80 | -13.20 |
| 1134 | Phthalic anhydride | -3259.40 | -3246.20 | -3246.80 | -13.20 |
| 1135 | 2-Aminobiphenyl | -6388.00 | -6374.80 | -6376.50 | -13.20 |
| 1136 | Ethane | -1560.90 | -1547.70 | -1546.80 | -13.20 |
| 1137 | 2,6-Dinitroaniline | -3025.00 | -3038.20 | -3036.90 | 13.20 |
| 1138 | Butadiyne | -2333.91 | -2320.70 | -2314.80 | -13.21 |
| 1139 | Hexylbenzene | -7184.46 | -7171.20 | -7171.90 | -13.26 |
| 1140 | 3-Methylbenzylalcohol | -4382.99 | -4369.70 | -4369.70 | -13.29 |
| 1141 | 3-Methylpyridine | -3428.90 | -3415.60 | -3417.70 | -13.30 |
| 1142 | 2,2'-Bipyridine | -5264.50 | -5251.10 | -5247.80 | -13.40 |
| 1143 | N-Carboxymethylglycine | -1657.10 | -1670.50 | -1678.10 | 13.40 |
| 1144 | Methacrolein | -2293.00 | -2306.40 | -2307.80 | 13.40 |
| 1145 | Decanoic acid | -6086.40 | -6099.80 | -6100.00 | 13.40 |
| 1146 | 3-Methylpyrrole | -2990.44 | -2977.00 | -2972.10 | -13.44 |
| 1147 | Hexadecylbenzene | -13682.73 | -13696.20 | -13695.20 | 13.47 |
| 1148 | Nitroacetic acid methylester | -1431.10 | -1417.60 | -1415.30 | -13.50 |
| 1149 | Tartaric acid | -1150.10 | -1136.60 | -1134.20 | -13.50 |
| 1150 | 1,2-Benzenedicarboxylic diphenylester | -9388.40 | -9374.90 | -9373.20 | -13.50 |
| 1151 | 5-Methylhydantoin | -1946.00 | -1959.50 | -1961.50 | 13.50 |
| 1152 | Xylose | -2340.40 | -2353.90 | -2356.80 | 13.50 |
| 1153 | Glyceryl-1-caprate | -7727.60 | -7741.10 | -7740.20 | 13.50 |
| 1154 | 1,2-Dimethoxypropane | -3262.69 | -3276.20 | -3275.40 | 13.51 |
| 1155 | Glycylalanylphenylalanine | -7300.50 | -7286.90 | -7290.60 | -13.60 |
| 1156 | Phenylhydrazine | -3645.40 | -3631.80 | -3630.00 | -13.60 |
| 1157 | Cyclohexanol | -3737.00 | -3723.40 | -3722.60 | -13.60 |
| 1158 | Pyrocatechol | -2859.60 | -2846.00 | -2846.80 | -13.60 |
| 1159 | 2,4-Dinitro-1,3-dimethylbenzene | -4205.80 | -4219.40 | -4219.60 | 13.60 |
| 1160 | DTBP | -5339.80 | -5353.40 | -5359.90 | 13.60 |
| 1161 | cis-3-Hexene | -4001.66 | -3988.00 | -3989.20 | -13.66 |
| 1162 | 3-Butynoic acid | -1905.33 | -1919.00 | -1920.40 | 13.67 |
| 1163 | Di-n-hexyladipate | -10788.67 | -10775.00 | -10776.10 | -13.67 |
| 1164 | Cyclohexane-1,4-dicarboxylic acid | -3867.81 | -3881.50 | -3881.10 | 13.69 |
| 1165 | Glycine anhydride | -1989.10 | -1975.40 | -1975.30 | -13.70 |
| 1166 | TNX | -4068.10 | -4054.40 | -4055.10 | -13.70 |
| 1167 | Isopropyl methyl sulfide | -3480.80 | -3494.50 | -3494.10 | 13.70 |
| 1168 | Nitroethane | -1349.00 | -1362.70 | -1363.40 | 13.70 |
| 1169 | Decamethylene glycol | -6389.90 | -6403.60 | -6403.20 | 13.70 |
| 1170 | Heptadecylbenzene | -14334.70 | -14348.40 | -14347.30 | 13.70 |
| 1171 | 4,4,6,6-Tetramethyl-1,3-dioxan | -4920.35 | -4934.10 | -4937.40 | 13.75 |
| 1172 | Propyleneglycol-2-monomethylether-1-acetate | -3415.53 | -3429.30 | -3430.90 | 13.77 |
| 1173 | Dimethylether | -1461.40 | -1447.60 | -1444.70 | -13.80 |
| 1174 | 5-Guanylaminotetrazole | -1672.20 | -1686.00 | -1696.60 | 13.80 |
| 1175 | 3-Hydroxypyridine | -2550.10 | -2563.90 | -2565.00 | 13.80 |
| 1176 | 3-Nitrophenol | -2875.10 | -2888.90 | -2888.80 | 13.80 |
| 1177 | Hexanenitrile | -3878.46 | -3892.30 | -3893.50 | 13.84 |
| 1178 | 1,2-Pentadiene | -3263.78 | -3249.90 | -3241.00 | -13.88 |
| 1179 | 2,4,4-Trimethyl-2-pentene | -5292.30 | -5278.40 | -5279.20 | -13.90 |
| 1180 | 1-Hexadecene | -10539.00 | -10525.10 | -10524.50 | -13.90 |
| 1181 | 1,3,5-Trinitrobenzene | -2753.00 | -2766.90 | -2767.30 | 13.90 |
| 1182 | Methylacetoacetate | -2487.70 | -2501.60 | -2501.20 | 13.90 |
| 1183 | 2-Naphthylacetic acid | -5779.30 | -5793.20 | -5793.80 | 13.90 |
| 1184 | 2-Chloro-1,1,1-trifluoroethane | -875.24 | -861.30 | -863.80 | -13.94 |
| 1185 | 2-Hydroxypropionitrile | -1757.47 | -1743.50 | -1741.90 | -13.97 |
| 1186 | cis-3-Penten-1-yne | -3053.41 | -3067.40 | -3070.60 | 13.99 |
| 1187 | Octadecylbenzene | -14987.09 | -15001.10 | -15002.10 | 14.01 |
| 1188 | t-Butylmethylsulfone | -3731.37 | -3745.40 | -3743.70 | 14.03 |
| 1189 | Dodecane | -8086.50 | -8072.40 | -8073.50 | -14.10 |
| 1190 | Propylcyclopentane | -5245.90 | -5231.80 | -5232.40 | -14.10 |
| 1191 | Erythritol | -2094.30 | -2108.40 | -2108.80 | 14.10 |
| 1192 | Dimethylacetal | -2585.00 | -2599.10 | -2597.20 | 14.10 |
| 1193 | Tetraethylsilane | -6686.30 | -6700.40 |  | 14.10 |
| 1194 | Tridecane | -8739.00 | -8724.80 | -8726.10 | -14.20 |
| 1195 | Dimethylmaleate | -2802.60 | -2788.40 | -2788.70 | -14.20 |
| 1196 | Salicylaldoxime | -3575.70 | -3561.50 | -3560.80 | -14.20 |
| 1197 | 4-Aminohexanoic acid | -3572.70 | -3586.90 | -3589.20 | 14.20 |
| 1198 | 2,4-Dinitro-1-naphthol | -4611.20 | -4625.40 | -4626.20 | 14.20 |
| 1199 | Neopentyl alcohol | -3302.98 | -3317.20 | -3317.60 | 14.22 |
| 1200 | 1-Undecene | -7276.93 | -7262.70 | -7262.60 | -14.23 |
| 1201 | 1-Methylcyclohexanol | -4366.66 | -4380.90 | -4381.10 | 14.24 |
| 1202 | 2,2,2-Trifluoroethanol | -821.53 | -835.80 | -836.50 | 14.27 |
| 1203 | Etioporphyrin II | -18053.00 | -18038.70 | -18022.30 | -14.30 |
| 1204 | 1-Bromobutane | -2716.50 | -2702.20 | -2702.00 | -14.30 |
| 1205 | Isophthalic acid | -3204.70 | -3219.00 | -3218.50 | 14.30 |
| 1206 | 1,3-Dichlorobutane | -2527.30 | -2541.60 | -2542.70 | 14.30 |
| 1207 | 8-Methyl-1-nonanol | -6600.83 | -6586.50 | -6585.90 | -14.33 |
| 1208 | 2-Ethoxyethylacetate | -3415.76 | -3430.10 | -3432.60 | 14.34 |
| 1209 | Amylamine | -3675.38 | -3661.00 | -3660.30 | -14.38 |
| 1210 | Capraldehyde | -6404.10 | -6389.70 | -6389.30 | -14.40 |
| 1211 | Cinnamic acid | -4359.30 | -4373.70 | -4372.70 | 14.40 |
| 1212 | 4-Chlorophenol | -2917.90 | -2903.50 | -2902.70 | -14.40 |
| 1213 | Trans-2,2'-dimethyl-5,5'-azotetrazole | -3188.20 | -3173.70 | -3160.70 | -14.50 |
| 1214 | Vinylidenechloride | -1096.00 | -1110.50 |  | 14.50 |
| 1215 | Pimelic acid | -3460.20 | -3474.70 | -3476.70 | 14.50 |
| 1216 | trans-3-Penten-1-yine | -3055.08 | -3069.60 | -3071.30 | 14.52 |
| 1217 | Trifluoroacetamide | -659.17 | -673.70 | -674.40 | 14.53 |
| 1218 | Isatin | -3602.70 | -3588.10 | -3585.30 | -14.60 |
| 1219 | Succinic anhydride | -1544.90 | -1559.50 | -1561.80 | 14.60 |
| 1220 | n-Butylstearate | -13972.00 | -13986.60 | -13986.50 | 14.60 |
| 1221 | 1,2-Dichloro-1,1,2-trifluoroethane | -761.06 | -775.70 |  | 14.64 |
| 1222 | 3-(Methylmercapto)-propanal | -3110.46 | -3095.80 | -3095.10 | -14.66 |
| 1223 | Methyllactate | -2076.08 | -2061.40 | -2060.20 | -14.68 |
| 1224 | 2-Methoxytetrahydropyran | -3633.80 | -3648.50 | -3648.90 | 14.70 |
| 1225 | 2-Nitroso-1-naphthol | -4873.60 | -4858.80 | -4856.50 | -14.80 |
| 1226 | 2-Allyl-5-aminotetrazole | -2859.20 | -2844.40 |  | -14.80 |
| 1227 | Fluoroethane | -1411.83 | -1397.00 | -1395.70 | -14.83 |
| 1228 | Benzylaniline | -7079.58 | -7064.70 | -7064.10 | -14.88 |
| 1229 | n-Heptaldehyde | -4447.20 | -4432.30 | -4433.10 | -14.90 |
| 1230 | Isobutyl acetate | -3534.00 | -3548.90 | -3550.10 | 14.90 |
| 1231 | 1,3-Dinitropropane | -1815.40 | -1830.30 | -1834.50 | 14.90 |
| 1232 | Formylurea | -866.67 | -881.60 | -886.70 | 14.93 |
| 1233 | Heptylbenzene | -7838.66 | -7823.70 | -7823.80 | -14.96 |
| 1234 | Phlorol | -4368.39 | -4353.40 | -4353.50 | -14.99 |
| 1235 | Alanylphenylalanine | -6301.10 | -6286.10 | -6288.20 | -15.00 |
| 1236 | Tetrachloroethylene | -830.90 | -815.90 |  | -15.00 |
| 1237 | cis-2-Methylcyclohexanol | -4393.00 | -4378.00 | -4377.50 | -15.00 |
| 1238 | Strychnine | -11244.00 | -11259.00 | -11269.70 | 15.00 |
| 1239 | Ethylsilane | -2696.30 | -2711.30 |  | 15.00 |
| 1240 | Cumulene | -1944.40 | -1959.50 | -1963.70 | 15.10 |
| 1241 | 2-Furoic acid | -2042.30 | -2057.40 | -2058.10 | 15.10 |
| 1242 | Oxamide | -844.48 | -859.60 | -864.90 | 15.12 |
| 1243 | Allyl acetate | -2726.65 | -2741.80 | -2744.60 | 15.15 |
| 1244 | n-Decyl alcohol | -6600.69 | -6585.50 | -6585.50 | -15.19 |
| 1245 | Dodecylcyclohexane | -11760.60 | -11745.40 | -11744.80 | -15.20 |
| 1246 | Methylcyclopentane | -3938.30 | -3923.10 | -3923.50 | -15.20 |
| 1247 | Mesaconic acid | -2002.00 | -1986.80 | -1986.20 | -15.20 |
| 1248 | Glutamine | -2572.80 | -2588.00 | -2589.70 | 15.20 |
| 1249 | 6-Hydroxyhexanoic acid | -3292.76 | -3308.00 | -3307.80 | 15.24 |
| 1250 | Skatole | -4899.40 | -4884.10 | -4884.40 | -15.30 |
| 1251 | 2,5-Dimethylbenzoic acid | -4514.60 | -4529.90 | -4530.60 | 15.30 |
| 1252 | n-Butyldichloroacetate | -3283.30 | -3267.90 | -3264.00 | -15.40 |
| 1253 | 1,1,2-Trichlorotrifluoroethane | -641.60 | -626.20 | -622.70 | -15.40 |
| 1254 | Aconitic acid | -1987.10 | -1971.70 | -1968.20 | -15.40 |
| 1255 | trans-Aconitic acid | -1987.10 | -1971.70 | -1970.90 | -15.40 |
| 1256 | n-Butylurea | -3263.10 | -3278.50 | -3283.90 | 15.40 |
| 1257 | 3-Methyl-2-butanethiol | -4126.00 | -4141.50 | -4142.30 | 15.50 |
| 1258 | Mandelic acid | -3714.50 | -3699.00 | -3699.10 | -15.50 |
| 1259 | Dicyanomethane | -1654.20 | -1669.70 | -1674.40 | 15.50 |
| 1260 | 1-Allyl-5-aminotetrazole | -2841.60 | -2857.10 | -2857.30 | 15.50 |
| 1261 | 3-Mercaptopropyl-trimethoxysilane | -5167.92 | -5152.40 | -5147.60 | -15.52 |
| 1262 | 1,2,5,6-Diacetone-mannitol | -6594.51 | -6610.10 | -6614.60 | 15.59 |
| 1263 | Cryofluorane | -589.90 | -605.50 | -607.30 | 15.60 |
| 1264 | Trichloroethylene | -947.70 | -963.30 |  | 15.60 |
| 1265 | Propanamide | -1840.50 | -1856.10 | -1859.20 | 15.60 |
| 1266 | Isophthaloylchloride | -3397.70 | -3413.30 | -3423.10 | 15.60 |
| 1267 | Vinylsilane | -2524.60 | -2540.20 |  | 15.60 |
| 1268 | 2,4-Dinitrotoluene | -3560.00 | -3575.60 | -3577.20 | 15.60 |
| 1269 | 2-Hydroxybenzaldehyde | -3334.40 | -3318.70 | -3317.10 | -15.70 |
| 1270 | 1-Undecanol | -7253.70 | -7238.00 | -7237.40 | -15.70 |
| 1271 | n-Undecanoic acid | -6736.50 | -6752.20 | -6751.50 | 15.70 |
| 1272 | 2-Nitrotoluene | -3756.40 | -3740.60 | -3739.50 | -15.80 |
| 1273 | 3-Ethylphenol | -4365.90 | -4350.10 | -4349.80 | -15.80 |
| 1274 | 4-t-Butylcatechol | -5461.90 | -5446.10 | -5446.00 | -15.80 |
| 1275 | Urazine | -1102.00 | -1086.20 | -1077.10 | -15.80 |
| 1276 | n-Amyl mercaptan | -4137.80 | -4122.00 | -4122.40 | -15.80 |
| 1277 | Hexamethyleneglycol | -3778.00 | -3793.80 | -3794.00 | 15.80 |
| 1278 | 1,7-Octadiyne | -4914.69 | -4930.50 | -4938.40 | 15.81 |
| 1279 | Cyclopentanoneethyleneacetal | -4054.98 | -4070.80 | -4072.10 | 15.82 |
| 1280 | Cytosine | -2067.30 | -2083.20 | -2082.30 | 15.90 |
| 1281 | cis-3-Heptene | -4654.25 | -4638.30 | -4637.40 | -15.95 |
| 1282 | Propyleneglycol-1-monomethylether-2-acetate | -3415.53 | -3431.50 | -3431.30 | 15.97 |
| 1283 | 2-Methyl-2-nitro-1,3-propanol | -2281.80 | -2265.80 | -2268.60 | -16.00 |
| 1284 | meso-Tartaric acid | -1152.60 | -1136.60 | -1134.50 | -16.00 |
| 1285 | 1,4-Dibromobutane | -2535.69 | -2551.70 | -2555.80 | 16.01 |
| 1286 | Ethyl isovalerate | -4184.40 | -4200.50 | -4198.70 | 16.10 |
| 1287 | Fluorobenzene | -3103.90 | -3120.00 | -3119.20 | 16.10 |
| 1288 | 4-Bromotoluene | -3747.89 | -3764.00 | -3769.60 | 16.11 |
| 1289 | 1-Tridecene | -8583.82 | -8567.70 | -8568.30 | -16.12 |
| 1290 | Pentadecane | -10046.00 | -10029.80 | -10031.20 | -16.20 |
| 1291 | beta-D-Fructose | -2813.50 | -2797.30 | -2794.60 | -16.20 |
| 1292 | n-Heptylamine | -4949.70 | -4965.90 | -4963.90 | 16.20 |
| 1293 | cis-3-Octene | -5309.21 | -5293.00 | -5292.30 | -16.21 |
| 1294 | 4-Hydroxybenzaldehyde | -3303.65 | -3319.90 | -3320.90 | 16.25 |
| 1295 | Heptadecane | -11351.00 | -11334.70 | -11334.20 | -16.30 |
| 1296 | 1,3,5-Benzenetricarboxylic acid | -3209.00 | -3192.70 | -3192.60 | -16.30 |
| 1297 | 1,2-Di-(5-tetrazolyl)ethane | -2877.60 | -2861.30 | -2841.00 | -16.30 |
| 1298 | 2-Butyne | -2576.80 | -2560.50 | -2560.60 | -16.30 |
| 1299 | 5-Tetrazolylurethane | -2355.90 | -2339.60 | -2332.20 | -16.30 |
| 1300 | Vinylcyclopropane | -3235.45 | -3219.10 | -3218.70 | -16.35 |
| 1301 | 1,1,2,2-Tetrachloroethane | -972.80 | -989.20 | -1007.60 | 16.40 |
| 1302 | 2-(2-(2-Methoxyethoxy)ethoxy)ethanol | -4258.18 | -4274.60 | -4279.20 | 16.42 |
| 1303 | Isophorone diisocyanate | -6870.46 | -6886.90 | -6915.40 | 16.44 |
| 1304 | n-Undecanal | -7058.67 | -7042.20 | -7041.90 | -16.47 |
| 1305 | 1-Thiaindane | -4955.91 | -4972.40 | -4976.50 | 16.49 |
| 1306 | 1-Thiaindan | -4955.91 | -4972.40 | -4977.00 | 16.49 |
| 1307 | Methyllaurate | -8143.70 | -8127.20 | -8127.50 | -16.50 |
| 1308 | 2,4-Lutidine | -4059.60 | -4043.10 | -4044.50 | -16.50 |
| 1309 | 2,3-Lutidine | -4062.90 | -4046.40 | -4044.40 | -16.50 |
| 1310 | Succinimide | -1828.30 | -1844.80 | -1844.70 | 16.50 |
| 1311 | 2-Methylpyrrole | -2993.24 | -2976.70 | -2978.50 | -16.54 |
| 1312 | Octylbenzene | -8492.95 | -8476.40 | -8475.60 | -16.55 |
| 1313 | Isopropyl myristate | -10736.17 | -10719.60 | -10718.80 | -16.57 |
| 1314 | Acetanilide | -4226.60 | -4243.20 | -4243.70 | 16.60 |
| 1315 | Hydantoic acid | -1289.50 | -1306.10 | -1305.70 | 16.60 |
| 1316 | 4-Fluorotoluene | -3747.20 | -3763.80 | -3763.60 | 16.60 |
| 1317 | 5-Amino-1-methyltetrazole | -1696.20 | -1712.80 | -1718.30 | 16.60 |
| 1318 | 2-Methyl-1,2-propanediol | -2464.00 | -2480.60 | -2481.20 | 16.60 |
| 1319 | trans-2,5-Dimethyl-3-hexene | -5275.50 | -5292.20 | -5293.40 | 16.70 |
| 1320 | 1-Tetradecene | -9236.81 | -9220.10 | -9220.40 | -16.71 |
| 1321 | Cetane | -10699.00 | -10682.20 | -10681.70 | -16.80 |
| 1322 | Methyl caprate | -6836.90 | -6820.10 | -6821.20 | -16.80 |
| 1323 | 1,2,4-Benzenetriol | -2654.80 | -2638.00 | -2642.10 | -16.80 |
| 1324 | Trilactic acid | -3999.10 | -4015.90 | -4015.00 | 16.80 |
| 1325 | 2-Methylenecyclohexanol | -4194.94 | -4211.80 | -4211.70 | 16.86 |
| 1326 | cis-4-Octene | -5309.86 | -5293.00 | -5292.30 | -16.86 |
| 1327 | Dichloroacetyl chloride | -717.29 | -700.40 | -694.80 | -16.89 |
| 1328 | 2,5-Lutidine | -4062.20 | -4045.30 | -4046.10 | -16.90 |
| 1329 | Octanamide | -5101.60 | -5118.50 | -5118.00 | 16.90 |
| 1330 | 1-Chloro-4-(trifluoromethyl)benzene | -3208.30 | -3225.20 | -3226.10 | 16.90 |
| 1331 | n-Octylamine | -5635.40 | -5618.40 | -5618.30 | -17.00 |
| 1332 | Oleic acid | -11167.00 | -11150.00 | -11149.30 | -17.00 |
| 1333 | n-Nonylformate | -6209.01 | -6192.00 | -6190.80 | -17.01 |
| 1334 | Benzyl alcohol | -3743.00 | -3725.90 | -3727.20 | -17.10 |
| 1335 | N-Methylaniline | -4073.00 | -4055.90 | -4055.80 | -17.10 |
| 1336 | 3-Nitrotoluene | -3723.50 | -3740.60 | -3740.80 | 17.10 |
| 1337 | Hexa-2-ene-1,6-dioic acid | -2633.50 | -2650.60 | -2651.10 | 17.10 |
| 1338 | Hexa-3-ene-1,6-dioic acid | -2633.50 | -2650.60 | -2652.10 | 17.10 |
| 1339 | 3-Sulfolene | -2716.64 | -2699.50 | -2698.50 | -17.14 |
| 1340 | 2-Chloropropane | -2028.40 | -2045.60 | -2047.80 | 17.20 |
| 1341 | 2,2'-Dichlorobiphenyl | -5945.50 | -5962.70 | -5966.40 | 17.20 |
| 1342 | 2-Thiocresol | -4547.08 | -4529.80 |  | -17.28 |
| 1343 | Brassidic acid | -13775.00 | -13757.70 | -13756.70 | -17.30 |
| 1344 | 2-Chloroaniline | -3237.50 | -3220.20 | -3220.90 | -17.30 |
| 1345 | 3-Azido-1,2,4-triazole | -1515.20 | -1532.50 | -1541.30 | 17.30 |
| 1346 | 3-Methyl-2,5-furandione | -2036.36 | -2019.00 | -2017.10 | -17.36 |
| 1347 | Octamethylcyclotetrasiloxane | -7778.74 | -7796.10 |  | 17.36 |
| 1348 | Di-n-hexyl phthalate | -11177.97 | -11160.60 | -11160.80 | -17.37 |
| 1349 | 2-Methylpropane | -2868.50 | -2851.10 | -2851.20 | -17.40 |
| 1350 | m-Toluic acid | -3866.50 | -3883.90 | -3884.20 | 17.40 |
| 1351 | t-Butylacetate | -3521.50 | -3538.90 | -3538.50 | 17.40 |
| 1352 | Glutaric acid | -2152.40 | -2169.80 | -2170.30 | 17.40 |
| 1353 | Azomethane | -1794.41 | -1777.00 | -1775.40 | -17.41 |
| 1354 | Diphenyl ether | -6119.24 | -6136.70 | -6138.20 | 17.46 |
| 1355 | Hexyl bromide | -4024.60 | -4007.10 | -4006.50 | -17.50 |
| 1356 | Pyrrole | -2353.30 | -2335.80 | -2334.80 | -17.50 |
| 1357 | Itaconic acid | -1983.90 | -2001.40 | -2001.60 | 17.50 |
| 1358 | 3-Azido-5-ethyl-1,2,4-triazole | -2798.90 | -2816.40 | -2813.90 | 17.50 |
| 1359 | Dulcitol | -3017.30 | -3034.80 | -3035.90 | 17.50 |
| 1360 | 1,2-Difluorobenzene | -2960.70 | -2978.20 | -2977.30 | 17.50 |
| 1361 | t-Butylacetylene | -3868.10 | -3885.60 | -3882.70 | 17.50 |
| 1362 | Menthol | -6318.51 | -6336.10 | -6338.30 | 17.59 |
| 1363 | Trehalose | -5647.20 | -5629.60 | -5619.40 | -17.60 |
| 1364 | 1,3-Diaminobenzene | -3497.00 | -3479.40 | -3478.50 | -17.60 |
| 1365 | 1,4-Diiodobenzene | -3093.20 | -3110.80 | -3115.30 | 17.60 |
| 1366 | 2,4-Dimethylbenzoic acid | -4512.30 | -4529.90 | -4530.60 | 17.60 |
| 1367 | n-Dodecanal | -7712.28 | -7694.60 | -7692.60 | -17.68 |
| 1368 | 3-Azido-5-methyl-1,2,4-triazole | -2146.20 | -2163.90 | -2168.70 | 17.70 |
| 1369 | Chloroethylene | -1240.05 | -1257.80 | -1261.70 | 17.75 |
| 1370 | Trichloroacetaldehyde | -780.19 | -762.40 | -761.80 | -17.79 |
| 1371 | Oxalic acid dihydrate | -217.71 | -235.50 | -257.40 | 17.79 |
| 1372 | 11-Butyldocosane | -17233.00 | -17215.20 | -17213.70 | -17.80 |
| 1373 | Triethylphosphate | -4115.60 | -4097.80 | -4090.80 | -17.80 |
| 1374 | Glycylglycine-N-carboxylic acid | -1969.90 | -1952.10 | -1951.00 | -17.80 |
| 1375 | 2-trans-Dodecene | -7918.53 | -7900.70 | -7900.60 | -17.83 |
| 1376 | Aziridine | -1591.36 | -1609.20 | -1611.40 | 17.84 |
| 1377 | 2-Chloroethanol | -1191.40 | -1209.30 | -1209.60 | 17.90 |
| 1378 | 9,10-Dihydroanthracene | -7290.50 | -7308.40 | -7307.60 | 17.90 |
| 1379 | 3-Nitrobenzoic acid | -3055.00 | -3073.00 | -3072.10 | 18.00 |
| 1380 | Di-s-butylsulfide | -6106.06 | -6087.90 | -6080.60 | -18.16 |
| 1381 | Methylmyristate | -9452.50 | -9434.30 | -9433.50 | -18.20 |
| 1382 | Dialuric acid | -1316.30 | -1298.10 | -1296.20 | -18.20 |
| 1383 | 2-Chloropropanoic acid | -1396.20 | -1378.00 | -1375.80 | -18.20 |
| 1384 | N,N'-Diphenyl-p-phenylenediamine | -9545.16 | -9563.40 | -9558.10 | 18.24 |
| 1385 | 1-Pentyne | -3257.35 | -3239.10 | -3240.30 | -18.25 |
| 1386 | Levulinic acid | -2415.40 | -2433.70 | -2434.20 | 18.30 |
| 1387 | Biphenyl | -6250.10 | -6268.40 | -6267.80 | 18.30 |
| 1388 | t-Butylethylether | -4013.98 | -4032.30 | -4033.10 | 18.32 |
| 1389 | Cycloheptane | -4597.00 | -4578.60 | -4577.60 | -18.40 |
| 1390 | Thioacetic acid | -1742.60 | -1761.00 |  | 18.40 |
| 1391 | Acrylamide | -1683.00 | -1701.40 | -1700.70 | 18.40 |
| 1392 | Theobromine | -3538.70 | -3557.10 | -3556.50 | 18.40 |
| 1393 | Isobutyl-t-butylether | -5317.37 | -5335.80 | -5337.70 | 18.43 |
| 1394 | Vinylpivalate | -4043.44 | -4025.00 | -4025.60 | -18.44 |
| 1395 | Acenaphthylene | -6056.20 | -6037.70 | -6039.30 | -18.50 |
| 1396 | 2-Methoxybenzaldehyde | -4025.00 | -4043.50 | -4044.40 | 18.50 |
| 1397 | Acetaldoxime | -1430.00 | -1411.50 | -1409.60 | -18.50 |
| 1398 | 2-Methyl-2-propanol | -2644.00 | -2662.50 | -2663.00 | 18.50 |
| 1399 | alpha-Pinene | -6205.30 | -6223.80 | -6225.90 | 18.50 |
| 1400 | 5-Ethyl-5-methylhydantoin | -3227.60 | -3246.10 | -3247.50 | 18.50 |
| 1401 | Perfluorohexadecane | -4952.88 | -4971.40 | -4992.90 | 18.52 |
| 1402 | Caprylic aldehyde | -5103.30 | -5084.70 | -5084.10 | -18.60 |
| 1403 | N-Ethylaniline | -4724.20 | -4705.60 | -4704.40 | -18.60 |
| 1404 | 2,3-Butandione | -2067.10 | -2048.50 | -2046.40 | -18.60 |
| 1405 | Phthalonitrile | -4000.30 | -4018.90 | -4024.00 | 18.60 |
| 1406 | 2-Phenyl-2-propanol | -5006.20 | -5024.80 | -5023.40 | 18.60 |
| 1407 | 1-Phenyl-2-propanol | -5039.91 | -5021.30 | -5021.10 | -18.61 |
| 1408 | Isopropyl methyl ether | -2753.15 | -2734.50 | -2735.30 | -18.65 |
| 1409 | 2-Vinylpyridine | -3913.40 | -3894.70 | -3892.40 | -18.70 |
| 1410 | 1-Phenyl-1-propanol | -5040.30 | -5021.60 | -5022.00 | -18.70 |
| 1411 | 1-Chloropropane | -2025.00 | -2043.70 | -2043.60 | 18.70 |
| 1412 | Oxindole | -3978.70 | -3997.40 | -3999.30 | 18.70 |
| 1413 | (L)-Hydroxyproline | -2594.10 | -2575.30 | -2568.30 | -18.80 |
| 1414 | 2,6-Dinitrotoluene | -3556.80 | -3575.60 | -3576.50 | 18.80 |
| 1415 | 3-Quinolinecarbonitrile | -5038.09 | -5056.90 | -5058.50 | 18.81 |
| 1416 | Maltose | -5649.20 | -5630.30 | -5628.50 | -18.90 |
| 1417 | N-Acetylurea | -1493.30 | -1512.20 | -1514.70 | 18.90 |
| 1418 | Perfluorobutanoic acid | -1009.53 | -990.60 | -990.40 | -18.93 |
| 1419 | 2,6-Dinitroanisol | -3425.37 | -3444.30 | -3444.60 | 18.93 |
| 1420 | alpha-Phenylglycine | -4005.10 | -3986.10 | -3986.00 | -19.00 |
| 1421 | TRIS | -2426.40 | -2445.40 | -2447.80 | 19.00 |
| 1422 | Di-n-octylether | -10579.97 | -10560.90 | -10559.70 | -19.07 |
| 1423 | Phenethyl alcohol | -4398.68 | -4379.60 | -4378.90 | -19.08 |
| 1424 | Methyltridecylate | -8798.80 | -8779.70 | -8780.10 | -19.10 |
| 1425 | 2,4-Dinitroanisol | -3466.60 | -3447.50 | -3446.90 | -19.10 |
| 1426 | Malonamide | -1493.00 | -1512.10 | -1509.80 | 19.10 |
| 1427 | 3-Nitroacetanilide | -4059.10 | -4078.20 | -4076.00 | 19.10 |
| 1428 | Benzo[h]quinoline | -6562.00 | -6581.10 | -6580.50 | 19.10 |
| 1429 | Tri-n-octylamine | -16145.00 | -16125.80 | -16124.80 | -19.20 |
| 1430 | iso-Butylsilane | -4036.10 | -4016.90 | -4009.10 | -19.20 |
| 1431 | Butyronitrile | -2568.20 | -2587.40 | -2589.20 | 19.20 |
| 1432 | Dimethylparabanic acid | -2253.30 | -2234.10 | -2232.50 | -19.20 |
| 1433 | Pentanenitrile | -3220.70 | -3239.90 | -3235.50 | 19.20 |
| 1434 | 1-Chlorooctadecane | -11850.00 | -11830.70 | -11830.70 | -19.30 |
| 1435 | Glutamic acid | -2245.00 | -2264.30 | -2262.90 | 19.30 |
| 1436 | trans-2-Pentadecene | -9877.41 | -9858.10 | -9859.50 | -19.31 |
| 1437 | 2,3-Dihydrothiophene | -3086.80 | -3067.40 | -3070.70 | -19.40 |
| 1438 | Ethylmethylether | -2108.80 | -2089.40 | -2088.80 | -19.40 |
| 1439 | 2-Pyrrolidone | -2290.20 | -2309.60 | -2311.80 | 19.40 |
| 1440 | Uracil | -1721.30 | -1740.70 | -1741.60 | 19.40 |
| 1441 | 2-Nitro-2-propyl-1,3-propanetriol | -3596.10 | -3576.60 | -3575.40 | -19.50 |
| 1442 | 4-Nitroaniline | -3186.20 | -3205.70 | -3205.10 | 19.50 |
| 1443 | n-Decylformate | -6864.00 | -6844.40 | -6842.50 | -19.60 |
| 1444 | Suberic acid | -4110.90 | -4130.50 | -4130.90 | 19.60 |
| 1445 | Tridecanal | -8366.70 | -8347.10 | -8348.00 | -19.60 |
| 1446 | 2,4,6-Trinitrobenzaldehyde | -3052.60 | -3032.90 | -3032.90 | -19.70 |
| 1447 | Piperazine | -2961.40 | -2981.10 | -2985.50 | 19.70 |
| 1448 | Decylbenzene | -9801.14 | -9781.40 | -9782.00 | -19.74 |
| 1449 | Hydrosorbic acid | -3338.00 | -3318.20 | -3317.30 | -19.80 |
| 1450 | 2-Methyl-1,3-dioxolane | -2332.30 | -2352.10 | -2354.10 | 19.80 |
| 1451 | Nonylbenzene | -9148.44 | -9128.60 | -9128.20 | -19.84 |
| 1452 | 1-Chloro-1,1-difluoroethane | -1056.72 | -1076.60 | -1078.50 | 19.88 |
| 1453 | Benzotrifluoride | -3355.66 | -3375.60 | -3377.10 | 19.94 |
| 1454 | 10-Undecenoic acid | -6615.10 | -6595.10 | -6595.10 | -20.00 |
| 1455 | Cyclopropane | -2092.80 | -2072.80 | -2068.50 | -20.00 |
| 1456 | Di-t-butyldisulfide | -6673.94 | -6653.90 | -6645.20 | -20.04 |
| 1457 | 3-Methylsulfolane | -3513.35 | -3533.40 | -3535.00 | 20.05 |
| 1458 | Acetone | -1816.50 | -1796.40 | -1797.70 | -20.10 |
| 1459 | D-Fucose | -2978.90 | -2999.00 | -3000.70 | 20.10 |
| 1460 | Isopropylacetate | -2869.80 | -2889.90 | -2890.10 | 20.10 |
| 1461 | 2-Nitro-2-isopropyl-1,3-propanetriol | -3598.00 | -3577.80 | -3576.30 | -20.20 |
| 1462 | Barbituric acid | -1509.80 | -1489.60 | -1484.30 | -20.20 |
| 1463 | Heptyl bromide | -4679.90 | -4659.60 | -4658.10 | -20.30 |
| 1464 | 4-Nitrophenol | -2868.50 | -2888.90 | -2889.50 | 20.40 |
| 1465 | 3-Nitro-p-toluidine | -3828.90 | -3849.40 | -3851.70 | 20.50 |
| 1466 | n-Decanoic acid | -6079.30 | -6099.80 | -6099.60 | 20.50 |
| 1467 | 9,10-Dimethylphenanthrene | -8344.60 | -8365.10 | -8361.60 | 20.50 |
| 1468 | Pentafluoroethane | -698.32 | -677.80 | -673.50 | -20.52 |
| 1469 | Benzoic anhydride | -6523.00 | -6502.40 | -6502.50 | -20.60 |
| 1470 | 1-Bromooctane | -5332.60 | -5312.00 | -5310.60 | -20.60 |
| 1471 | 3-Amino-1,2,4-triazole | -1436.50 | -1415.80 | -1399.80 | -20.70 |
| 1472 | cis-1,2-Divinylcyclobutane | -4987.30 | -5008.00 | -5011.10 | 20.70 |
| 1473 | n-Hexylamine | -4292.80 | -4313.50 | -4313.50 | 20.70 |
| 1474 | 1-Fluoropropane | -2070.13 | -2049.40 | -2046.80 | -20.73 |
| 1475 | 1,5-Hexadiyne | -3604.87 | -3625.60 | -3619.80 | 20.73 |
| 1476 | Refrigerant 216 | -946.47 | -925.70 | -915.80 | -20.77 |
| 1477 | Perfluoropropane | -787.18 | -808.00 | -809.70 | 20.82 |
| 1478 | trans-1,2-Dibromocyclohexane | -3632.20 | -3611.30 | -3604.30 | -20.90 |
| 1479 | Nopinene | -6214.10 | -6235.00 | -6234.70 | 20.90 |
| 1480 | Hexanamide | -3792.50 | -3813.50 | -3813.60 | 21.00 |
| 1481 | Acetoxyacetic acid | -1553.45 | -1574.50 | -1575.00 | 21.05 |
| 1482 | Aminomethylcumulene | -2774.47 | -2753.40 | -2743.60 | -21.07 |
| 1483 | Cyclopropanecarboxylic acid | -2035.00 | -2056.10 | -2056.30 | 21.10 |
| 1484 | 1-Nitronaphthalene | -4978.50 | -4999.60 | -5001.50 | 21.10 |
| 1485 | 5,12-Dihydrotetracene | -9190.00 | -9211.10 | -9209.60 | 21.10 |
| 1486 | Cyclohexanecarbonitrile | -4279.50 | -4300.60 | -4296.50 | 21.10 |
| 1487 | Palmitic peroxyacid | -10074.00 | -10095.10 | -10098.40 | 21.10 |
| 1488 | Phenanthrene | -7048.60 | -7069.70 | -7074.40 | 21.10 |
| 1489 | n-Butyl-2-chloropropanoate | -4066.12 | -4045.00 | -4044.60 | -21.12 |
| 1490 | cis-Butenedinitrile | -2129.31 | -2150.50 | -2150.10 | 21.19 |
| 1491 | 1-Ethoxy-2-nitrobenzene | -4275.60 | -4254.40 | -4253.30 | -21.20 |
| 1492 | 2-Phenylpyrrole | -5360.50 | -5339.30 | -5338.50 | -21.20 |
| 1493 | Tropolone | -3382.00 | -3403.20 | -3404.30 | 21.20 |
| 1494 | Propane | -2221.40 | -2200.10 | -2200.80 | -21.30 |
| 1495 | 3-Nitroaniline | -3184.32 | -3205.70 | -3205.70 | 21.38 |
| 1496 | 1,2-Dibromobutane | -2571.80 | -2550.40 | -2547.40 | -21.40 |
| 1497 | 1-Butene | -2716.80 | -2695.40 | -2695.10 | -21.40 |
| 1498 | Acetamide | -1184.40 | -1205.80 | -1206.20 | 21.40 |
| 1499 | 5-Dimethylaminotetrazole | -2365.10 | -2386.50 | -2389.30 | 21.40 |
| 1500 | Cyanoethylene | -1756.40 | -1777.80 | -1773.60 | 21.40 |
| 1501 | 5,5-Dimethyl-1,3-dioxane | -3614.70 | -3636.10 | -3638.10 | 21.40 |
| 1502 | Thiourea | -1478.03 | -1456.60 | -1449.00 | -21.43 |
| 1503 | MTBE | -3369.00 | -3390.50 | -3391.80 | 21.50 |
| 1504 | N-Methyldiphenylamine | -7094.00 | -7115.50 | -7116.50 | 21.50 |
| 1505 | trans-3-Methylcyclohexanol | -4356.50 | -4378.00 | -4378.20 | 21.50 |
| 1506 | 1,4-Diphenylbutane | -8858.70 | -8880.20 | -8878.30 | 21.50 |
| 1507 | o-Toluidine | -4036.00 | -4014.40 | -4014.80 | -21.60 |
| 1508 | 1,1-Dichloroethane | -1246.80 | -1268.40 | -1277.50 | 21.60 |
| 1509 | p-Toluic acid | -3862.30 | -3883.90 | -3884.40 | 21.60 |
| 1510 | 2-Chloropropene | -1897.95 | -1876.30 | -1875.00 | -21.65 |
| 1511 | Creatinine | -2338.50 | -2360.20 | -2366.50 | 21.70 |
| 1512 | Di-n-butylperoxide | -5343.67 | -5365.40 | -5376.10 | 21.73 |
| 1513 | 2-Aminoacridine | -6715.55 | -6693.80 | -6692.20 | -21.75 |
| 1514 | Octadecane | -12009.00 | -11987.20 | -11989.10 | -21.80 |
| 1515 | 4-Acetylphenol | -3927.10 | -3948.90 | -3950.30 | 21.80 |
| 1516 | 5-Methyluracil | -2362.23 | -2384.10 | -2383.50 | 21.87 |
| 1517 | Ethyl chloride | -1413.10 | -1391.20 | -1391.20 | -21.90 |
| 1518 | p-Benzidine | -6508.00 | -6486.00 | -6481.10 | -22.00 |
| 1519 | Stearic peroxyacid | -11378.00 | -11400.00 | -11404.50 | 22.00 |
| 1520 | Propane-1,3-dithiol | -3451.30 | -3473.30 | -3474.90 | 22.00 |
| 1521 | Pyridine-3-nitrile | -3128.30 | -3150.30 | -3155.20 | 22.00 |
| 1522 | Triphenylcarbinol | -9760.90 | -9738.80 | -9740.20 | -22.10 |
| 1523 | 5-Methyl-1-phenyltetrazole | -4587.10 | -4609.20 | -4616.20 | 22.10 |
| 1524 | Diethanolamine | -2652.30 | -2674.40 | -2675.40 | 22.10 |
| 1525 | Propionic anhydride | -3113.64 | -3091.50 | -3088.30 | -22.14 |
| 1526 | 3-Ethyl-1-heptanol | -5963.47 | -5941.30 | -5940.90 | -22.17 |
| 1527 | Dicyclohexylsulfide | -8232.09 | -8209.90 | -8199.70 | -22.19 |
| 1528 | 3-Azido-5-phenyl-1,2,4-triazole | -4540.60 | -4518.40 | -4509.50 | -22.20 |
| 1529 | 2-Picoline | -3421.60 | -3399.40 | -3397.30 | -22.20 |
| 1530 | Di-t-butylsulfide | -6094.46 | -6072.20 | -6067.10 | -22.26 |
| 1531 | Nonadecane | -12662.00 | -12639.70 | -12639.70 | -22.30 |
| 1532 | 2,4,6-Trinitro-m-cresol | -3223.80 | -3201.50 | -3202.70 | -22.30 |
| 1533 | 2,4,6-Trinitrophenylhydrazine | -3112.10 | -3134.40 | -3141.40 | 22.30 |
| 1534 | Thiirane | -2012.60 | -2034.90 | -2037.70 | 22.30 |
| 1535 | cis- Cyclooctene | -5078.33 | -5056.00 | -5057.40 | -22.33 |
| 1536 | Benzylethylsulfide | -5853.20 | -5830.80 | -5828.60 | -22.40 |
| 1537 | Hydurilic acid | -2755.80 | -2733.40 | -2726.60 | -22.40 |
| 1538 | 1,3-Dibromobutane | -2527.99 | -2550.40 | -2551.70 | 22.41 |
| 1539 | Isopropenylacetate | -2726.55 | -2704.10 | -2703.90 | -22.45 |
| 1540 | Aniline | -3393.10 | -3370.60 | -3372.30 | -22.50 |
| 1541 | Trihexylamine | -12232.32 | -12209.80 | -12210.60 | -22.52 |
| 1542 | s-Butyl-t-butylether | -5306.87 | -5329.40 | -5330.60 | 22.53 |
| 1543 | Tetradecane | -9399.84 | -9377.30 | -9376.30 | -22.54 |
| 1544 | Methyl pentadecanoate | -10105.00 | -10082.40 | -10082.60 | -22.60 |
| 1545 | 4-Ethoxyaniline | -4550.70 | -4528.10 | -4525.20 | -22.60 |
| 1546 | Dimethyl oxalate | -1675.60 | -1653.00 | -1652.90 | -22.60 |
| 1547 | 2,7-Dimethylnaphthalene | -6431.60 | -6454.30 | -6455.50 | 22.70 |
| 1548 | 2,2,4,4-Tetramethyl-1,3-cyclobutanedione | -4483.20 | -4505.90 | -4507.00 | 22.70 |
| 1549 | Perylene | -9767.60 | -9790.30 | -9794.10 | 22.70 |
| 1550 | DIDP | -16407.22 | -16384.40 | -16384.20 | -22.82 |
| 1551 | Di-n-octyladipate | -13403.44 | -13380.60 | -13377.30 | -22.84 |
| 1552 | 3,3,6,6-Tetramethyl-1,7-octadiyne | -7510.54 | -7533.40 | -7536.70 | 22.86 |
| 1553 | Benzil | -6784.00 | -6761.10 | -6758.90 | -22.90 |
| 1554 | 1,3-Butyleneglycol | -2502.20 | -2479.30 | -2479.00 | -22.90 |
| 1555 | 3,3-Dimethyl-1,4-pentadiyne | -4249.18 | -4272.10 | -4279.70 | 22.92 |
| 1556 | Nitroglycerin | -1525.47 | -1502.50 | -1491.80 | -22.97 |
| 1557 | Quinoline | -4701.40 | -4678.40 | -4677.20 | -23.00 |
| 1558 | Di-n-octylamine | -10891.00 | -10867.90 | -10867.50 | -23.10 |
| 1559 | Trans-2,2'-diethyl-5,5'-azotetrazole | -4448.50 | -4471.60 | -4489.20 | 23.10 |
| 1560 | Hexamethylcyclotrisiloxane | -5858.31 | -5835.20 |  | -23.11 |
| 1561 | 1-Pentadecene | -9895.79 | -9872.60 | -9873.00 | -23.19 |
| 1562 | 1-Phenyltetrazolone | -3726.30 | -3703.10 | -3684.50 | -23.20 |
| 1563 | Dichloroacetic acid | -624.00 | -600.80 | -599.80 | -23.20 |
| 1564 | Pyrrole-2-carbaldehyde | -2575.70 | -2599.00 | -2602.30 | 23.30 |
| 1565 | trans-2-Pentenenitrile | -3044.84 | -3068.20 | -3068.10 | 23.36 |
| 1566 | Tetraethylurea | -6019.00 | -5995.60 | -5982.20 | -23.40 |
| 1567 | Myristic peroxyacid | -8766.70 | -8790.10 | -8794.90 | 23.40 |
| 1568 | Cholesterol | -16524.00 | -16547.40 | -16551.00 | 23.40 |
| 1569 | N-Nitropiperidine | -3305.97 | -3329.40 | -3331.80 | 23.43 |
| 1570 | Methyl-p-toluate | -4580.76 | -4604.20 | -4603.90 | 23.44 |
| 1571 | t-Butylmethacrylate | -4654.13 | -4677.60 | -4676.70 | 23.47 |
| 1572 | 1-Hexyl-1,2,3,4-tetrahydronaphthalene | -9553.29 | -9529.80 | -9531.60 | -23.49 |
| 1573 | Sorbic acid | -3123.00 | -3146.50 | -3144.80 | 23.50 |
| 1574 | 1,6-Hexanediamine | -4445.81 | -4469.40 | -4469.80 | 23.59 |
| 1575 | Norpempidine | -6050.10 | -6026.50 | -6021.00 | -23.60 |
| 1576 | 4-Chlorobenzoic acid | -3064.00 | -3087.60 | -3088.80 | 23.60 |
| 1577 | N-Methyl-4-nitroaniline | -3867.00 | -3890.60 | -3892.20 | 23.60 |
| 1578 | 2,2-diphenyl-1,3-dioxolane | -7700.74 | -7677.10 | -7679.80 | -23.64 |
| 1579 | Undecylbenzene | -10457.25 | -10433.60 | -10433.60 | -23.65 |
| 1580 | 1,2-Dibromododecane | -7746.54 | -7770.20 | -7774.10 | 23.66 |
| 1581 | Isoprene | -3158.20 | -3181.90 | -3183.30 | 23.70 |
| 1582 | Tetrahydrothiophene | -3248.70 | -3272.40 | -3272.50 | 23.70 |
| 1583 | Epichlorohydrin | -1761.60 | -1785.40 | -1782.30 | 23.80 |
| 1584 | 6-Aminohexanol | -4107.80 | -4131.60 | -4133.00 | 23.80 |
| 1585 | Allyl cyanide | -2406.40 | -2430.20 | -2432.10 | 23.80 |
| 1586 | Eicosane | -13316.00 | -13292.10 | -13291.10 | -23.90 |
| 1587 | Pentafluorobenzoic acid | -2552.80 | -2528.90 | -2524.40 | -23.90 |
| 1588 | Tri-n-propylphosphate | -6079.20 | -6055.20 | -6047.60 | -24.00 |
| 1589 | 3-Mercaptopropionic acid | -2173.41 | -2149.40 | -2149.90 | -24.01 |
| 1590 | trans-2-Butene | -2705.00 | -2680.90 | -2680.30 | -24.10 |
| 1591 | 4,6-Dinitroresorcinol | -2489.30 | -2513.50 | -2515.20 | 24.20 |
| 1592 | Citric acid monohydrate | -1953.10 | -1977.30 | -1980.90 | 24.20 |
| 1593 | 2,6-Diethylaniline | -5998.66 | -5974.40 | -5973.70 | -24.26 |
| 1594 | Pyruvaldehyde | -1443.00 | -1418.70 | -1418.00 | -24.30 |
| 1595 | 2,6-Lutidine | -4053.50 | -4029.10 | -4032.40 | -24.40 |
| 1596 | Cortisol | -11480.00 | -11455.60 | -11455.00 | -24.40 |
| 1597 | Diisopropylamine | -4333.80 | -4358.20 | -4364.20 | 24.40 |
| 1598 | Propanenitrile | -1910.50 | -1934.90 | -1937.10 | 24.40 |
| 1599 | 2-Chlorophenol | -2927.92 | -2903.50 | -2901.40 | -24.42 |
| 1600 | 3-Methyl-1,2-butadiene | -3214.12 | -3238.60 | -3243.40 | 24.48 |
| 1601 | 4-Nitroacetanilide | -4053.70 | -4078.20 | -4076.30 | 24.50 |
| 1602 | 2,3-Dinitroaniline | -3064.00 | -3039.40 | -3039.90 | -24.60 |
| 1603 | 1,2-Dibenzoylethane | -8041.40 | -8066.00 | -8067.90 | 24.60 |
| 1604 | Barbital | -4118.10 | -4093.50 | -4096.90 | -24.60 |
| 1605 | Neohexanoic acid | -4163.56 | -4138.90 | -4138.30 | -24.66 |
| 1606 | 3,5-Dimethylpyrrole-2-carboxylic ethylester | -4928.59 | -4953.30 | -4957.70 | 24.71 |
| 1607 | 1-Hexyne | -3916.34 | -3891.60 | -3892.10 | -24.74 |
| 1608 | Tricyanoethylene | -2551.17 | -2526.40 | -2526.50 | -24.77 |
| 1609 | cis-2-Butene-1,4-diol | -2346.28 | -2321.50 | -2323.40 | -24.78 |
| 1610 | Terephthalic acid | -3189.30 | -3214.10 | -3214.90 | 24.80 |
| 1611 | 1,1,1-Trifluoroethane | -992.88 | -1017.70 | -1015.70 | 24.82 |
| 1612 | Trichloroacetylchloride | -597.05 | -572.20 | -569.90 | -24.85 |
| 1613 | 2,2-Dichloropropane | -1879.50 | -1904.40 |  | 24.90 |
| 1614 | 2-Oxetanone | -1423.20 | -1448.10 | -1447.80 | 24.90 |
| 1615 | Thianaphthene | -4708.20 | -4733.20 | -4737.30 | 25.00 |
| 1616 | Trans-1,1'-dimethyl-5,5'-azotetrazole | -3224.00 | -3199.00 | -3177.80 | -25.00 |
| 1617 | Trichloroacetic acid | -497.50 | -472.50 | -467.30 | -25.00 |
| 1618 | 4,4'-Dinitrohydrazobenzene | -6265.10 | -6290.10 | -6301.80 | 25.00 |
| 1619 | 1-Tridecanol | -8517.80 | -8542.90 | -8543.60 | 25.10 |
| 1620 | 2,6-Dimethylnaphthalene | -6431.40 | -6456.50 | -6459.30 | 25.10 |
| 1621 | 2-Methyl-1,3-dioxane | -2960.30 | -2985.40 | -2983.20 | 25.10 |
| 1622 | 1,5-Dimethyltetrazole | -2228.20 | -2253.30 | -2259.50 | 25.10 |
| 1623 | Dimethylmaleic anhydride | -2637.20 | -2662.30 | -2663.30 | 25.10 |
| 1624 | n-Butylnonanoate | -8139.54 | -8114.40 | -8113.70 | -25.14 |
| 1625 | cis-2-Butene | -2709.40 | -2684.20 | -2683.50 | -25.20 |
| 1626 | Quinizarin | -6057.40 | -6082.60 | -6081.60 | 25.20 |
| 1627 | 4,5-Dimethylpyrrole-3-carboxylic ethylester | -4932.79 | -4958.00 | -4959.60 | 25.21 |
| 1628 | Di-isooctyl phthalate | -13797.65 | -13772.40 | -13774.30 | -25.25 |
| 1629 | 1,2-Butadiene | -2572.14 | -2597.40 | -2602.70 | 25.26 |
| 1630 | Isobutyronitrile | -2560.60 | -2585.90 | -2587.90 | 25.30 |
| 1631 | Diisopropanolamine | -3934.98 | -3960.30 | -3963.10 | 25.32 |
| 1632 | t-Butylformate | -2899.32 | -2924.70 | -2929.20 | 25.38 |
| 1633 | 11-Decylheneicosane | -20503.00 | -20477.60 | -20477.60 | -25.40 |
| 1634 | 1-Phenylindene | -7771.33 | -7796.90 | -7796.10 | 25.57 |
| 1635 | Azodicarbonamide | -1066.90 | -1041.30 | -1021.80 | -25.60 |
| 1636 | 2-Chloroethyl ethyl ether | -2549.16 | -2574.80 | -2575.20 | 25.64 |
| 1637 | Hydroxyacetonitrile | -1074.81 | -1100.50 | -1102.90 | 25.69 |
| 1638 | Dimethyl phthalate | -4680.40 | -4654.70 | -4654.60 | -25.70 |
| 1639 | Methylelaidate | -11894.00 | -11868.20 | -11868.00 | -25.80 |
| 1640 | Ethyl acrylate | -2715.00 | -2740.80 | -2741.30 | 25.80 |
| 1641 | Cyclobutanecarbonitrile | -3071.00 | -3096.80 | -3099.30 | 25.80 |
| 1642 | 4-Methylbiphenyl | -6888.60 | -6914.40 | -6915.60 | 25.80 |
| 1643 | n-Nonylamine | -6296.74 | -6270.90 | -6269.10 | -25.84 |
| 1644 | 2-Phenyl-5,5-dimethyl-1,3-dioxane | -6616.44 | -6642.30 | -6644.30 | 25.86 |
| 1645 | Butyl oleate | -13843.00 | -13817.10 | -13816.60 | -25.90 |
| 1646 | Tetramethylurea | -3420.40 | -3394.40 | -3397.80 | -26.00 |
| 1647 | Allyltrichloroacetate | -2356.00 | -2330.00 | -2323.60 | -26.00 |
| 1648 | 2,4-dichloro-1-trifluoromethylbenzene | -3048.74 | -3074.80 | -3080.60 | 26.06 |
| 1649 | Acetonitrile | -1256.33 | -1282.40 | -1278.20 | 26.07 |
| 1650 | 1-Nitroso-2-naphthol | -4884.90 | -4858.80 | -4860.30 | -26.10 |
| 1651 | Benzylmethylsulfide | -5205.10 | -5179.00 | -5177.60 | -26.10 |
| 1652 | 2-Ethylthioethanol | -3317.12 | -3291.00 | -3288.50 | -26.12 |
| 1653 | Spiro[2.2]pentane | -3270.60 | -3244.40 | -3236.40 | -26.20 |
| 1654 | 2,5-Xylidine | -4684.40 | -4658.20 | -4658.90 | -26.20 |
| 1655 | Cyclohexylisocyanate | -4128.89 | -4102.60 | -4100.20 | -26.29 |
| 1656 | 1-Bromonaphthalene | -5047.10 | -5020.80 | -5012.30 | -26.30 |
| 1657 | Thiolactic acid | -2174.60 | -2148.30 | -2142.60 | -26.30 |
| 1658 | Chloropentafluorobenzene | -2428.70 | -2402.40 | -2403.20 | -26.30 |
| 1659 | Succinic acid | -1491.00 | -1517.30 | -1516.20 | 26.30 |
| 1660 | Diethylperoxide | -2781.90 | -2755.50 | -2745.70 | -26.40 |
| 1661 | Tolan | -7250.00 | -7276.40 | -7278.10 | 26.40 |
| 1662 | Guajen | -6431.00 | -6457.50 | -6458.30 | 26.50 |
| 1663 | Butane | -2879.20 | -2852.60 | -2852.60 | -26.60 |
| 1664 | Methyloxiran | -1917.40 | -1944.00 | -1948.00 | 26.60 |
| 1665 | Hexafluoroacetone | -763.12 | -736.50 | -735.80 | -26.62 |
| 1666 | 1,1-Difluoroethylene | -1091.87 | -1118.50 | -1123.50 | 26.63 |
| 1667 | trans-2-Eicosene | -13149.28 | -13122.60 | -13122.10 | -26.68 |
| 1668 | Trihydroxyglutaric acid | -1624.50 | -1597.80 | -1595.70 | -26.70 |
| 1669 | 2,4-Dinitrophenol | -2697.20 | -2723.90 | -2722.20 | 26.70 |
| 1670 | Tetradecanoic acid | -8683.00 | -8709.70 | -8709.30 | 26.70 |
| 1671 | Di-n-Decylphthalate | -16407.12 | -16380.40 | -16382.50 | -26.72 |
| 1672 | Dimethylterephthalate | -4631.66 | -4658.40 | -4658.30 | 26.74 |
| 1673 | Succinamide | -2137.80 | -2164.60 | -2164.80 | 26.80 |
| 1674 | 5-Hydroxypentanal | -2918.60 | -2945.40 | -2943.90 | 26.80 |
| 1675 | 2-Methyl-1-dodecanol | -8570.74 | -8543.90 | -8543.20 | -26.84 |
| 1676 | 1-Pentadecanoic acid | -9335.21 | -9362.10 | -9361.70 | 26.89 |
| 1677 | m-Dinitrobenzene | -2905.00 | -2931.90 | -2932.40 | 26.90 |
| 1678 | 6-Methyluracil | -2356.90 | -2383.80 | -2388.50 | 26.90 |
| 1679 | Myristyl alcohol | -9168.40 | -9195.40 | -9195.70 | 27.00 |
| 1680 | HOX | -2193.90 | -2166.90 | -2142.90 | -27.00 |
| 1681 | N-Nitrodiethylamine | -2897.00 | -2924.00 | -2927.30 | 27.00 |
| 1682 | Perfluorocyclohexene | -2186.10 | -2159.10 |  | -27.00 |
| 1683 | Chloroacetaldehyde | -986.46 | -1013.50 | -1014.50 | 27.04 |
| 1684 | 4-Carboxyphenol | -3005.40 | -3032.50 | -3032.00 | 27.10 |
| 1685 | Dodecylbenzene | -11113.44 | -11086.20 | -11087.00 | -27.24 |
| 1686 | Di-n-butylsebacate | -10793.57 | -10766.30 | -10762.80 | -27.27 |
| 1687 | 2-Formylbenzoic acid | -3476.71 | -3504.00 | -3504.50 | 27.29 |
| 1688 | 2-Methyl-2-nitro-1-phenyl-1-propanol | -5479.30 | -5452.00 | -5446.10 | -27.30 |
| 1689 | 11-Cyclohexylheneicosane | -17652.00 | -17624.60 | -17622.70 | -27.40 |
| 1690 | Triphenylene | -8949.90 | -8977.30 | -8979.70 | 27.40 |
| 1691 | Phthalic heptylnonylester | -13799.95 | -13772.50 | -13769.60 | -27.45 |
| 1692 | 1-Triacontene | -19632.22 | -19659.70 | -19659.10 | 27.48 |
| 1693 | 1,2,4-Benzenetricarboxylic acid | -3219.90 | -3192.40 | -3190.90 | -27.50 |
| 1694 | Ethyl bromide | -1424.70 | -1397.20 | -1395.80 | -27.50 |
| 1695 | 1-Cyclohexenecarbonitrile | -4091.60 | -4119.10 | -4123.00 | 27.50 |
| 1696 | Dibenzylsulfide | -8216.35 | -8188.80 | -8187.50 | -27.55 |
| 1697 | Phenyltrimethoxymethane | -5447.98 | -5420.40 | -5415.70 | -27.58 |
| 1698 | 2-Naphthylamine | -5301.00 | -5273.40 | -5273.30 | -27.60 |
| 1699 | n-Butylchloroacetate | -3418.30 | -3390.70 | -3388.30 | -27.60 |
| 1700 | 1,3-Dithiane | -3913.94 | -3886.30 |  | -27.64 |
| 1701 | Thianthrene | -7250.00 | -7222.30 | -7182.00 | -27.70 |
| 1702 | Lauric acid | -7377.00 | -7404.70 | -7404.20 | 27.70 |
| 1703 | Bicyclo[3.1.0]hexane | -3831.34 | -3803.60 | -3802.80 | -27.74 |
| 1704 | Pentachlorophenol | -2329.70 | -2301.80 | -2290.00 | -27.90 |
| 1705 | Imidazole | -1814.10 | -1786.20 | -1779.70 | -27.90 |
| 1706 | Guanine | -2498.20 | -2526.10 | -2527.60 | 27.90 |
| 1707 | trans-Azobenzene | -6475.30 | -6503.30 | -6521.40 | 28.00 |
| 1708 | Trimethylurea | -2672.70 | -2700.70 | -2705.70 | 28.00 |
| 1709 | Tetryl | -3502.30 | -3530.40 |  | 28.10 |
| 1710 | Di-n-propyl phthalate | -7275.12 | -7246.90 | -7244.20 | -28.22 |
| 1711 | Lactose | -5652.20 | -5623.90 | -5621.50 | -28.30 |
| 1712 | Hydroxyisobutyric acid | -1974.50 | -2002.80 | -2004.00 | 28.30 |
| 1713 | 1,1-Dichloro-1-fluoroethane | -1068.96 | -1097.30 | -1106.10 | 28.34 |
| 1714 | n-Decylamine | -6951.78 | -6923.40 | -6923.20 | -28.38 |
| 1715 | Lauryl bromide | -7950.30 | -7921.90 | -7920.90 | -28.40 |
| 1716 | 1,4-Naphthoquinone | -4604.10 | -4632.50 | -4634.40 | 28.40 |
| 1717 | Methyl acrylate | -2069.40 | -2097.80 | -2098.50 | 28.40 |
| 1718 | Cyclooctane | -5265.70 | -5237.20 | -5240.70 | -28.50 |
| 1719 | m-Terphenyl | -9245.30 | -9273.80 | -9273.80 | 28.50 |
| 1720 | n-Butyltrichloroacetate | -3168.20 | -3139.60 | -3140.10 | -28.60 |
| 1721 | Phenoxyacetic acid | -3780.30 | -3751.70 | -3750.30 | -28.60 |
| 1722 | Bis(2,2,2-Trinitroethyl)urea | -2522.50 | -2551.10 | -2587.50 | 28.60 |
| 1723 | Stearic acid | -11290.80 | -11319.50 | -11319.40 | 28.70 |
| 1724 | Fluorene | -6635.00 | -6663.70 | -6664.40 | 28.70 |
| 1725 | 2-Pentyne | -3241.85 | -3213.00 | -3210.90 | -28.85 |
| 1726 | 2,5-Dimethylpyrrole-3-carboxylic ethylester | -4924.39 | -4953.30 | -4954.70 | 28.91 |
| 1727 | 1-Heptadecene | -11206.67 | -11177.60 | -11177.60 | -29.07 |
| 1728 | Terephthalaldehyde | -3764.82 | -3793.90 | -3795.90 | 29.08 |
| 1729 | D-Ribose | -2347.40 | -2376.50 | -2374.70 | 29.10 |
| 1730 | Cyclopropanecarbonitrile | -2429.40 | -2458.50 | -2457.70 | 29.10 |
| 1731 | 4-Nitroso-1-naphthol | -4831.00 | -4860.10 | -4861.80 | 29.10 |
| 1732 | 3-Nitrophenetol | -4225.30 | -4254.40 | -4254.50 | 29.10 |
| 1733 | 4-t-Butylphenol | -5625.80 | -5654.90 | -5654.30 | 29.10 |
| 1734 | Phenylglyoxylic acid | -3518.35 | -3489.20 | -3489.10 | -29.15 |
| 1735 | 2-Nitroethanol | -1210.00 | -1180.80 | -1179.40 | -29.20 |
| 1736 | 2,4-Dinitroaniline | -3010.00 | -3039.40 | -3040.90 | 29.40 |
| 1737 | 1,1,1,2,2-Pentafluoropropane | -1367.51 | -1337.90 | -1335.00 | -29.61 |
| 1738 | Ethyldiethylcarbamate | -4306.00 | -4335.80 | -4336.00 | 29.80 |
| 1739 | 1,4-Difluorobenzene | -2948.40 | -2978.20 | -2979.40 | 29.80 |
| 1740 | Neoheptanoic acid | -4824.82 | -4794.90 | -4795.30 | -29.92 |
| 1741 | 2,3-Pentadiene | -3265.37 | -3235.40 | -3229.50 | -29.97 |
| 1742 | 3-Nitrobiphenyl | -6073.50 | -6103.50 | -6104.30 | 30.00 |
| 1743 | 1-Naphthoic acid | -5110.70 | -5140.70 | -5141.80 | 30.00 |
| 1744 | 3-Fluorobenzoic acid | -3066.10 | -3096.20 | -3096.60 | 30.10 |
| 1745 | 2-Methyltetrahydrothiophene | -3893.60 | -3923.80 | -3926.60 | 30.20 |
| 1746 | 1-Pentadecanol | -9817.70 | -9847.90 | -9848.20 | 30.20 |
| 1747 | 1,3-Dithiolane | -3254.15 | -3284.40 |  | 30.25 |
| 1748 | 1,2,2-Trichloropropane | -1778.30 | -1748.00 |  | -30.30 |
| 1749 | 3,3'-Dimethylbiphenyl | -7529.90 | -7560.30 | -7562.10 | 30.40 |
| 1750 | Di-n-undecyl phthalate | -17715.71 | -17685.30 | -17687.40 | -30.41 |
| 1751 | trans-Crotononitrile | -2385.24 | -2415.70 | -2418.20 | 30.46 |
| 1752 | Oxalic acid | -242.90 | -212.40 | -213.70 | -30.50 |
| 1753 | Cortisone | -11204.00 | -11234.50 | -11234.20 | 30.50 |
| 1754 | 4-Nonylphenol | -8951.51 | -8921.00 | -8920.70 | -30.51 |
| 1755 | Neopentanoic acid | -3514.76 | -3484.00 | -3484.20 | -30.76 |
| 1756 | Tridecylbenzene | -11769.29 | -11738.50 | -11738.00 | -30.79 |
| 1757 | Tetramethylthiourea | -4246.70 | -4215.80 | -4194.50 | -30.90 |
| 1758 | S-Ethyl thioacetate | -3090.46 | -3059.50 | -3046.70 | -30.96 |
| 1759 | Diallyl phthalate | -6964.37 | -6933.40 | -6934.90 | -30.97 |
| 1760 | Benzo[f]quinoline | -6552.50 | -6583.50 | -6584.10 | 31.00 |
| 1761 | 1-Methyl-5-phenyltetrazole | -4583.70 | -4614.80 | -4629.00 | 31.10 |
| 1762 | Bicyclo(2.2.2)octane | -5005.43 | -4974.10 | -4975.40 | -31.33 |
| 1763 | 3-Butyne-2-amine | -2779.06 | -2747.70 | -2743.80 | -31.36 |
| 1764 | Chrysene | -8943.50 | -8974.90 | -8971.70 | 31.40 |
| 1765 | n-Undecylamine | -7607.24 | -7575.80 | -7575.60 | -31.44 |
| 1766 | N-Methyl-1,8-Naphthalimide | -6100.04 | -6131.50 | -6132.10 | 31.46 |
| 1767 | 1,3-Difluorobenzene | -2946.70 | -2978.20 | -2978.90 | 31.50 |
| 1768 | 1-Octadecene | -11861.52 | -11830.00 | -11830.20 | -31.52 |
| 1769 | 1,4-Phenylenediamine | -3511.00 | -3479.40 | -3473.80 | -31.60 |
| 1770 | 2-Hydroxyethylmethacrylate | -3165.14 | -3196.80 | -3198.00 | 31.66 |
| 1771 | Camphor | -5902.00 | -5933.90 | -5936.50 | 31.90 |
| 1772 | 4-Methyl-1-phenyl-2,6,7-trioxabicyclo[2.2.2]octane | -6234.02 | -6202.10 | -6196.80 | -31.92 |
| 1773 | 5-Butyldocosane | -17247.16 | -17215.20 | -17214.60 | -31.96 |
| 1774 | Pyracene | -7308.80 | -7276.80 | -7274.10 | -32.00 |
| 1775 | Pyrene | -7850.70 | -7882.70 | -7883.30 | 32.00 |
| 1776 | n-Heptyl-n-undecylphthalate | -15107.54 | -15075.40 | -15074.40 | -32.14 |
| 1777 | p-Terphenyl | -9242.80 | -9275.00 | -9276.00 | 32.20 |
| 1778 | 1,1,1-Trichloroethane | -1108.00 | -1140.20 | -1141.00 | 32.20 |
| 1779 | Adenine | -2781.10 | -2813.50 | -2835.50 | 32.40 |
| 1780 | 4-Fluorobenzoic acid | -3063.80 | -3096.20 | -3096.80 | 32.40 |
| 1781 | 1-Nonylnaphthalene | -11067.39 | -11034.80 | -11038.10 | -32.59 |
| 1782 | Glycolaldehyde | -955.39 | -988.00 | -989.60 | 32.61 |
| 1783 | Vinyl ether | -2393.35 | -2360.70 | -2349.20 | -32.65 |
| 1784 | Ethyl acetoacetate | -3180.50 | -3147.80 | -3148.40 | -32.70 |
| 1785 | Tris(hydroxymethyl)nitromethane | -2118.00 | -2085.10 | -2081.50 | -32.90 |
| 1786 | 9,10-Diphenylanthracene | -13112.40 | -13079.40 | -13080.50 | -33.00 |
| 1787 | cis,cis-1,4-Diphenylbutadiene | -8501.31 | -8534.40 | -8535.70 | 33.09 |
| 1788 | Monoacetin | -2488.20 | -2521.30 | -2522.10 | 33.10 |
| 1789 | 1-Heptadecanol | -11119.67 | -11152.80 | -11152.30 | 33.13 |
| 1790 | 4-Nitrobenzoic acid | -3042.00 | -3075.40 | -3075.00 | 33.40 |
| 1791 | Bisphenol A | -7840.00 | -7806.50 | -7806.70 | -33.50 |
| 1792 | Perfluorobutane | -1161.72 | -1128.20 | -1124.80 | -33.52 |
| 1793 | 2-Oxoglutaric acid | -1799.70 | -1766.10 | -1766.30 | -33.60 |
| 1794 | 4-Nitrotoluene | -3706.90 | -3740.60 | -3741.20 | 33.70 |
| 1795 | 1,4-Dicyano-2-butyne | -3301.18 | -3335.00 | -3346.20 | 33.82 |
| 1796 | 1-Nonadecene | -12516.49 | -12482.50 | -12482.10 | -33.99 |
| 1797 | 4,4'-Dimethylbiphenyl | -7526.10 | -7560.30 | -7558.10 | 34.20 |
| 1798 | 2,3,4,6-Tetranitroaniline | -2742.50 | -2708.20 | -2704.50 | -34.30 |
| 1799 | Di-n-nonylphthalate | -15109.74 | -15075.40 | -15074.60 | -34.34 |
| 1800 | 2-Butyl-1-decanol | -9235.54 | -9201.20 | -9201.10 | -34.34 |
| 1801 | 2-Butyl-1-nonanol | -8583.24 | -8548.80 | -8547.50 | -34.44 |
| 1802 | N-Methyl-2-propyne-1-amine | -2810.26 | -2775.80 | -2773.40 | -34.46 |
| 1803 | Raffinose | -8478.30 | -8443.70 | -8441.10 | -34.60 |
| 1804 | Hexafluorobenzene | -2445.60 | -2411.00 | -2408.00 | -34.60 |
| 1805 | Tetramethylthiacyclopropane | -4598.15 | -4632.80 | -4643.70 | 34.65 |
| 1806 | Methylthiirane | -2651.60 | -2686.30 | -2683.10 | 34.70 |
| 1807 | 2-Butyl-1-octanol | -7931.35 | -7896.30 | -7896.20 | -35.05 |
| 1808 | Trimethyl trimellitate | -5386.40 | -5351.20 | -5348.70 | -35.20 |
| 1809 | Tetradecylbenzene | -12426.32 | -12391.10 | -12391.00 | -35.22 |
| 1810 | 2,4,6-Trinitrophenetol | -3956.50 | -3921.10 | -3920.30 | -35.40 |
| 1811 | 4-Amino-3-azido-5-methyl-1,2,4-triazole | -2380.60 | -2416.00 | -2422.20 | 35.40 |
| 1812 | Adiponitrile | -3591.62 | -3627.10 | -3631.60 | 35.48 |
| 1813 | Uric acid | -1920.40 | -1884.90 | -1874.50 | -35.50 |
| 1814 | Acetoacetanilide | -5107.51 | -5143.20 | -5144.20 | 35.69 |
| 1815 | Diphenyl sulfide | -6916.90 | -6881.10 | -6872.90 | -35.80 |
| 1816 | Dipentaerythritol | -5507.00 | -5542.80 | -5547.90 | 35.80 |
| 1817 | Diacetylperoxide | -1896.40 | -1860.50 | -1844.60 | -35.90 |
| 1818 | Tiglic acid | -2621.80 | -2657.70 | -2659.60 | 35.90 |
| 1819 | Butane-1,2,4-triol | -2260.10 | -2296.10 | -2295.00 | 36.00 |
| 1820 | Oxirane | -1262.90 | -1298.90 | -1300.30 | 36.00 |
| 1821 | 1-Bromohexadecane | -10568.00 | -10531.80 | -10530.50 | -36.20 |
| 1822 | Heneicosane | -13908.24 | -13944.60 | -13943.60 | 36.36 |
| 1823 | 1-Decylnaphthalene | -11723.49 | -11687.10 | -11691.00 | -36.39 |
| 1824 | Pyrazole | -1857.60 | -1894.00 | -1913.30 | 36.40 |
| 1825 | Pyruvic acid | -1165.30 | -1128.80 | -1127.50 | -36.50 |
| 1826 | Phenylisocyanate | -3408.00 | -3444.60 | -3448.60 | 36.60 |
| 1827 | Cyclopentadiene | -2961.23 | -2924.60 | -2925.20 | -36.63 |
| 1828 | Galactaric acid | -2022.20 | -2059.00 | -2060.10 | 36.80 |
| 1829 | Succinonitrile | -2285.40 | -2322.20 | -2326.70 | 36.80 |
| 1830 | Butyric anhydride | -4433.21 | -4396.40 | -4399.10 | -36.81 |
| 1831 | n-Butyl-3-chloropropanoate | -4079.92 | -4043.10 | -4043.90 | -36.82 |
| 1832 | 1,2,3-Benzenetriol | -2674.90 | -2638.00 | -2637.50 | -36.90 |
| 1833 | 1-Pyrroline | -2648.86 | -2611.90 | -2607.30 | -36.96 |
| 1834 | Vinylethylenecarbonate | -2271.53 | -2308.50 | -2314.00 | 36.97 |
| 1835 | Anthraquinone | -6464.00 | -6501.00 | -6503.30 | 37.00 |
| 1836 | 1H-Indazole | -3764.00 | -3801.20 | -3819.60 | 37.20 |
| 1837 | Thiohydantoic acid | -2086.70 | -2124.00 | -2136.10 | 37.30 |
| 1838 | Palmitic acid | -9977.20 | -10014.60 | -10014.50 | 37.40 |
| 1839 | 3-Butyne-1-amine | -2780.06 | -2742.60 | -2739.10 | -37.46 |
| 1840 | 2,4-Dinitro-N-methylaniline | -3688.00 | -3725.50 | -3731.20 | 37.50 |
| 1841 | Hydrogenperoxide | -98.02 | -60.50 | -27.90 | -37.52 |
| 1842 | Cyclobutanoneimine | -2734.16 | -2696.60 | -2682.50 | -37.56 |
| 1843 | Lactide | -2712.50 | -2674.90 | -2672.90 | -37.60 |
| 1844 | p-Quaterphenyl | -12244.00 | -12281.60 | -12281.60 | 37.60 |
| 1845 | Docosane | -14559.42 | -14597.10 | -14596.30 | 37.68 |
| 1846 | 3-Nitrocinnamic acid | -4171.00 | -4208.70 | -4208.80 | 37.70 |
| 1847 | 2-Furanacrylic acid | -3153.00 | -3190.70 | -3192.90 | 37.70 |
| 1848 | p-Dinitrobenzene | -2894.00 | -2931.90 | -2933.30 | 37.90 |
| 1849 | Methyloleate | -11832.40 | -11870.30 | -11870.00 | 37.90 |
| 1850 | Dimethyl-2,6-naphthalinedicarboxylate | -6519.44 | -6557.40 | -6560.60 | 37.96 |
| 1851 | Benzo[b]triphenylene | -10842.00 | -10880.00 | -10881.50 | 38.00 |
| 1852 | 2-Methyl-2,3,3-trinitropentane | -3642.80 | -3680.90 | -3689.30 | 38.10 |
| 1853 | 1-Octyne | -5234.73 | -5196.50 | -5195.00 | -38.23 |
| 1854 | N,N’-Bianiline | -6658.40 | -6620.10 | -6596.10 | -38.30 |
| 1855 | 1,4-Benzoquinone | -2725.80 | -2764.10 | -2767.90 | 38.30 |
| 1856 | Hexaethylbenzene | -11027.00 | -11065.40 | -11071.40 | 38.40 |
| 1857 | n-Valeraldehyde | -3166.00 | -3127.30 | -3126.60 | -38.70 |
| 1858 | Tricosane | -15210.59 | -15249.50 | -15248.80 | 38.91 |
| 1859 | 1,3,5-Triphenylbenzene | -12238.70 | -12278.00 | -12280.60 | 39.30 |
| 1860 | Tri-n-butyl phosphate | -7980.00 | -8019.50 | -8033.70 | 39.50 |
| 1861 | 3-(1-Methyl-2-pyrrolidinyl)pyridine | -5979.20 | -6018.70 | -6026.70 | 39.50 |
| 1862 | 2,2'-Dipyrrolylmethane | -5100.26 | -5060.70 | -5048.50 | -39.56 |
| 1863 | cis-3-Methylcyclohexanol | -4334.70 | -4374.40 | -4374.70 | 39.70 |
| 1864 | 2-Methyl-1-tridecanol | -9236.24 | -9196.40 | -9196.70 | -39.84 |
| 1865 | 1-Nonadecanoic acid | -11932.08 | -11972.00 | -11970.50 | 39.92 |
| 1866 | Dichlorodimethylsilane | -2058.19 | -2018.20 | -1982.20 | -39.99 |
| 1867 | 1,5-Cyclooctadiene | -4920.80 | -4880.80 | -4879.50 | -40.00 |
| 1868 | alpha-Terpinene | -6205.30 | -6165.30 | -6162.40 | -40.00 |
| 1869 | cis-4-Methylcyclohexanol | -4338.00 | -4378.00 | -4377.50 | 40.00 |
| 1870 | 1,1,2,2-Tetramethylcyclopropane | -4635.60 | -4675.60 | -4676.70 | 40.00 |
| 1871 | Carbazole | -6110.10 | -6150.10 | -6153.70 | 40.00 |
| 1872 | Tetracosane | -15861.76 | -15902.00 | -15904.60 | 40.24 |
| 1873 | Amalic acid | -5190.80 | -5150.40 | -5135.90 | -40.40 |
| 1874 | (Chloromethyl)benzene | -3708.70 | -3749.20 | -3748.80 | 40.50 |
| 1875 | 4,4'-Dichlorobiphenyl | -5927.10 | -5967.60 | -5967.30 | 40.50 |
| 1876 | 4-Formylbenzoic acid | -3463.34 | -3504.00 | -3505.10 | 40.66 |
| 1877 | Phenanthridine | -6542.80 | -6583.50 | -6582.40 | 40.70 |
| 1878 | Epiandrosterone | -11137.00 | -11095.80 | -11096.90 | -41.20 |
| 1879 | Terebic acid | -3257.30 | -3298.70 | -3300.70 | 41.40 |
| 1880 | Pentacosane | -16512.94 | -16554.50 | -16554.30 | 41.56 |
| 1881 | 1,3-Dimethyluracil | -3096.21 | -3137.80 | -3138.90 | 41.59 |
| 1882 | 1-Docosanoic acid | -13971.00 | -13929.40 | -13928.40 | -41.60 |
| 1883 | Dibenz[ah]anthracene | -10836.00 | -10877.60 | -10885.30 | 41.60 |
| 1884 | s-Butenylacetate | -3383.74 | -3342.00 | -3337.20 | -41.74 |
| 1885 | Erucic acid | -13804.00 | -13762.10 | -13760.00 | -41.90 |
| 1886 | 4-Dimethylaminobenzaldehyde | -4977.00 | -5019.30 | -5019.90 | 42.30 |
| 1887 | 1-Bromodecane | -6574.66 | -6617.00 | -6620.20 | 42.34 |
| 1888 | 4-Nitrophenetole | -4211.90 | -4254.40 | -4253.90 | 42.50 |
| 1889 | 9,9'-Bianthracene | -13917.00 | -13874.40 | -13871.90 | -42.60 |
| 1890 | Pivalic anhydride | -5732.02 | -5689.40 | -5684.50 | -42.62 |
| 1891 | Hexacosane | -17164.11 | -17206.90 | -17205.60 | 42.79 |
| 1892 | Margaric acid | -10624.00 | -10667.10 | -10667.00 | 43.10 |
| 1893 | Methylisocyanate | -1124.58 | -1081.40 | -1074.20 | -43.18 |
| 1894 | Dibenzofuran | -5836.30 | -5879.80 | -5886.70 | 43.50 |
| 1895 | (L)-Cystine | -4255.00 | -4211.40 | -4193.70 | -43.60 |
| 1896 | trans-1,2-Divinylcyclobutane | -4964.30 | -5008.00 | -5008.30 | 43.70 |
| 1897 | 2-Azabicyclo[2.1.0]pentane | -2826.97 | -2782.90 | -2780.00 | -44.07 |
| 1898 | Heptacosane | -17815.29 | -17859.40 | -17858.00 | 44.11 |
| 1899 | Cyclononane | -5931.40 | -5887.20 | -5886.30 | -44.20 |
| 1900 | Dicyanoacetylene | -2074.40 | -2030.00 | -2025.10 | -44.40 |
| 1901 | Diethyl oxalate | -2981.30 | -2936.70 | -2933.30 | -44.60 |
| 1902 | trans-Stilbene | -7357.10 | -7401.70 | -7404.00 | 44.60 |
| 1903 | 1,4-Dimethyl-5-tetrazolone | -2011.80 | -2056.70 | -2094.30 | 44.90 |
| 1904 | 4-Amino-3-azido-5-phenyl-1,2,4-triazole | -4819.00 | -4774.00 | -4761.30 | -45.00 |
| 1905 | 1,4-Bis(methoxycarbonyl)-cyclohexane | -5277.00 | -5322.10 | -5324.20 | 45.10 |
| 1906 | Di-n-butyl phthalate | -8598.00 | -8552.90 | -8550.40 | -45.10 |
| 1907 | Androstan | -11740.00 | -11694.80 | -11693.00 | -45.20 |
| 1908 | 5-Cyanotetrazole | -1332.00 | -1286.80 | -1285.10 | -45.20 |
| 1909 | Hexafluoropropylene | -1144.12 | -1098.90 |  | -45.22 |
| 1910 | 1,1,2-Trimethylcyclopropane | -3979.90 | -4025.20 | -4027.90 | 45.30 |
| 1911 | Octacosane | -18466.46 | -18511.90 | -18511.50 | 45.44 |
| 1912 | 5-Nitro-o-toluidine | -3809.30 | -3854.90 | -3855.90 | 45.60 |
| 1913 | 7-Methylhypoxanthine | -3179.50 | -3133.80 | -3125.60 | -45.70 |
| 1914 | Ethylepoxide | -2548.50 | -2594.30 | -2593.80 | 45.80 |
| 1915 | 2-Hydroxymethyl-1,3-propanediol | -2260.60 | -2306.40 | -2309.60 | 45.80 |
| 1916 | Diphenylbutadiyne | -8243.00 | -8289.20 | -8309.10 | 46.20 |
| 1917 | (L)-Histidine | -3205.50 | -3159.00 | -3158.20 | -46.50 |
| 1918 | Dichlordiethylsilane | -3367.48 | -3414.00 | -3464.80 | 46.52 |
| 1919 | Tetraconic acid | -3332.70 | -3286.10 | -3285.40 | -46.60 |
| 1920 | 2-Nitrocinnamic acid | -4162.00 | -4208.70 | -4211.80 | 46.70 |
| 1921 | Benzimidazole | -3691.00 | -3737.70 | -3737.90 | 46.70 |
| 1922 | Nonacosane | -19117.63 | -19164.40 | -19162.60 | 46.77 |
| 1923 | Glyceryldibrassidate | -29115.00 | -29161.90 | -29164.90 | 46.90 |
| 1924 | Acetone glycerol | -3390.00 | -3437.00 | -3439.90 | 47.00 |
| 1925 | Bis-(2-hydroxyethyl)terephalate | -5527.42 | -5574.60 | -5576.80 | 47.18 |
| 1926 | Trifluoroacetic acid | -397.20 | -350.00 | -347.80 | -47.20 |
| 1927 | Protoporphyrin dimethylester | -19099.00 | -19146.20 | -19155.90 | 47.20 |
| 1928 | Bis[3-(triethoxysilyl)propyl]disulfide | -13836.78 | -13884.10 | -13919.30 | 47.32 |
| 1929 | 3-Hydroxybutyric acid | -2039.80 | -1992.30 | -1990.50 | -47.50 |
| 1930 | 2-Pyridone | -2517.62 | -2565.40 | -2565.90 | 47.78 |
| 1931 | Triacontane | -19768.81 | -19816.80 | -19820.30 | 47.99 |
| 1932 | Protoporphyrin IX | -17746.00 | -17697.90 | -17682.40 | -48.10 |
| 1933 | 1,3-Diacetin | -3349.40 | -3397.70 | -3398.50 | 48.30 |
| 1934 | 3-(2-Furanyl)-2-propenal | -3430.00 | -3478.40 | -3481.00 | 48.40 |
| 1935 | Hemimellitic acid | -3238.70 | -3190.20 | -3190.40 | -48.50 |
| 1936 | 4-Nitrosodiphenylamine | -6364.40 | -6315.80 | -6296.80 | -48.60 |
| 1937 | Vinylacetylene | -2380.00 | -2429.50 | -2430.60 | 49.50 |
| 1938 | Phenylalanyl anhydride | -9368.02 | -9318.40 | -9313.50 | -49.62 |
| 1939 | Perfluorobutene | -1316.15 | -1365.80 |  | 49.65 |
| 1940 | Tetraphenylhydrazine | -12755.00 | -12705.20 | -12647.90 | -49.80 |
| 1941 | Arachidic acid | -12574.20 | -12624.50 | -12626.00 | 50.30 |
| 1942 | 1,1,2-Trifluoroethane | -1006.78 | -1057.10 | -1070.50 | 50.32 |
| 1943 | Cyclobutene | -2589.85 | -2539.30 | -2538.60 | -50.55 |
| 1944 | 2,5-Dihydro-1H-pyrrole | -2694.86 | -2644.00 | -2642.70 | -50.86 |
| 1945 | Pyromellitic acid | -3221.80 | -3170.70 | -3168.80 | -51.10 |
| 1946 | Phenacetin | -5452.00 | -5400.70 | -5398.40 | -51.30 |
| 1947 | Benzo[k]fluoranthene | -9757.60 | -9808.90 | -9810.50 | 51.30 |
| 1948 | 4-Pyridone | -2533.20 | -2584.50 | -2584.80 | 51.30 |
| 1949 | Methyl tetryl | -4225.70 | -4174.20 |  | -51.50 |
| 1950 | 1,1,1-Trinitroethane | -1119.68 | -1171.30 | -1178.60 | 51.62 |
| 1951 | 1-Decyne | -6553.12 | -6501.40 | -6498.60 | -51.72 |
| 1952 | 2,2'-Difluorobiphenyl | -5927.90 | -5979.90 | -5980.80 | 52.00 |
| 1953 | 2-Phenylglyceric acid | -4226.60 | -4174.50 | -4172.00 | -52.10 |
| 1954 | Tetramethylsilane | -3851.90 | -3904.20 |  | 52.30 |
| 1955 | Parabanic acid | -889.70 | -837.10 | -831.90 | -52.60 |
| 1956 | Bicyclo[1.1.0]butane | -2650.25 | -2597.10 | -2592.30 | -53.15 |
| 1957 | Lophine | -10831.00 | -10777.70 | -10764.30 | -53.30 |
| 1958 | Acetone cyanohydrin | -2450.60 | -2397.30 | -2400.00 | -53.30 |
| 1959 | 1,6-Hexanedioic acid | -2768.70 | -2822.30 | -2824.10 | 53.60 |
| 1960 | Cetylmethacrylate | -12567.05 | -12513.30 | -12513.90 | -53.75 |
| 1961 | Paraldehyde | -3394.20 | -3448.00 | -3461.30 | 53.80 |
| 1962 | Perfluorocyclobutane | -1436.15 | -1382.20 | -1378.00 | -53.95 |
| 1963 | 3,5-Dimethylpyrrole-2,4-dicarboxylic ethylester | -6235.00 | -6289.00 | -6299.80 | 54.00 |
| 1964 | Inositol | -2716.66 | -2771.00 | -2772.90 | 54.34 |
| 1965 | Gluconic acid | -2489.10 | -2543.50 | -2542.20 | 54.40 |
| 1966 | Stachyose | -11325.00 | -11270.40 | -11251.60 | -54.60 |
| 1967 | 4-Nitrobiphenyl | -6048.80 | -6103.50 | -6104.10 | 54.70 |
| 1968 | Trinitroacetonitrile | -961.00 | -906.10 | -898.10 | -54.90 |
| 1969 | Glyceryltribrassidate | -42860.00 | -42915.00 | -42922.90 | 55.00 |
| 1970 | 1H-Benzotriazole | -3312.20 | -3367.60 | -3377.70 | 55.40 |
| 1971 | Hexatriacontane | -23675.80 | -23731.60 | -23735.90 | 55.80 |
| 1972 | 1,1,1-Trichlorotrifluoroethane | -554.18 | -610.20 | -614.30 | 56.02 |
| 1973 | trans-4-Methylcyclohexanol | -4317.80 | -4374.40 | -4375.60 | 56.60 |
| 1974 | Dinitromesitylene | -4922.40 | -4865.40 | -4864.90 | -57.00 |
| 1975 | Thebaine | -10221.00 | -10164.00 | -10149.90 | -57.00 |
| 1976 | Lysine | -3683.20 | -3740.40 | -3744.90 | 57.20 |
| 1977 | 5-Aminotetrazole | -1069.13 | -1011.50 | -1007.70 | -57.63 |
| 1978 | 4,4'-Difluorobiphenyl | -5927.10 | -5984.80 | -5984.70 | 57.70 |
| 1979 | Acetylenedicarboxylic acid | -1282.91 | -1225.20 | -1221.40 | -57.71 |
| 1980 | Ornithine | -3029.90 | -3087.90 | -3091.90 | 58.00 |
| 1981 | Trifluoroacetonitrile | -810.54 | -752.50 | -748.50 | -58.04 |
| 1982 | Linolenic acid | -10869.43 | -10811.00 | -10806.80 | -58.43 |
| 1983 | Glyceryltribenzoate | -11413.00 | -11353.80 | -11352.30 | -59.20 |
| 1984 | Oxetan | -1996.50 | -1937.20 | -1936.60 | -59.30 |
| 1985 | trans,trans-1,4-Diphenylbutadiene | -8475.70 | -8535.00 | -8536.60 | 59.30 |
| 1986 | N-Methylcarbazole | -6791.40 | -6850.80 | -6851.80 | 59.40 |
| 1987 | 1-Dodecanol | -7950.00 | -7890.50 | -7890.10 | -59.50 |
| 1988 | Nitrosobenzene | -3105.00 | -3165.30 | -3181.80 | 60.30 |
| 1989 | Glyceryltriacetate | -4213.60 | -4274.10 | -4276.40 | 60.50 |
| 1990 | Xanthone | -6062.20 | -6122.90 | -6131.30 | 60.70 |
| 1991 | Dipicrylamine | -5481.30 | -5420.30 | -5410.40 | -61.00 |
| 1992 | 1,2,4-Trinitrobenzene | -2828.00 | -2766.90 | -2765.10 | -61.10 |
| 1993 | 1,2-Diaminobenzene | -3543.50 | -3481.90 | -3480.50 | -61.60 |
| 1994 | 4-Nitrocinnamic acid | -4147.00 | -4208.70 | -4211.80 | 61.70 |
| 1995 | 3-(Triethoxysilyl)-1-propanamine | -6659.03 | -6597.20 | -6609.70 | -61.83 |
| 1996 | Aspirin | -3872.44 | -3935.30 | -3938.80 | 62.86 |
| 1997 | Di-n-propylmaleate | -5440.79 | -5377.10 | -5373.30 | -63.69 |
| 1998 | Diphencyprone | -7562.00 | -7498.10 | -7494.40 | -63.90 |
| 1999 | Uramil | -1586.00 | -1650.60 | -1658.80 | 64.60 |
| 2000 | Dotriacontane | -21056.00 | -21121.80 | -21125.40 | 65.80 |
| 2001 | Isatide | -7435.80 | -7369.50 | -7356.60 | -66.30 |
| 2002 | Dimethyldipropylsilane | -6673.80 | -6607.40 |  | -66.40 |
| 2003 | Pyracyclene | -6979.10 | -6910.80 | -6911.90 | -68.30 |
| 2004 | Octaethylporphyrin | -20587.00 | -20655.50 | -20668.90 | 68.50 |
| 2005 | m-Toluidine | -4083.00 | -4014.40 | -4012.40 | -68.60 |
| 2006 | Dibenzo[b,d]thiophene | -6571.60 | -6640.30 | -6652.40 | 68.70 |
| 2007 | Glyoxal | -861.47 | -792.20 | -789.30 | -69.27 |
| 2008 | DINP | -15007.54 | -15077.10 | -15080.30 | 69.56 |
| 2009 | Di-n-octyl phthalate | -13839.00 | -13768.00 | -13767.30 | -71.00 |
| 2010 | 2-Propylpiperidine | -5340.30 | -5412.90 | -5415.70 | 72.60 |
| 2011 | 2,4,6-Trinitrochlorobenzene | -2691.20 | -2616.50 | -2610.80 | -74.70 |
| 2012 | Galactonic acid | -2467.70 | -2543.20 | -2543.60 | 75.50 |
| 2013 | Carbostyril | -4397.10 | -4472.60 | -4475.50 | 75.50 |
| 2014 | Methylenecyclopropane | -2633.65 | -2556.80 | -2555.10 | -76.85 |
| 2015 | 3-Chlorobenzoic acid | -3009.70 | -3087.60 | -3088.80 | 77.90 |
| 2016 | 9,9'-Bifluorenyl | -13001.00 | -13079.60 | -13085.20 | 78.60 |
| 2017 | 9,9'-Biphenanthrene | -13803.00 | -13884.20 | -13893.20 | 81.20 |
| 2018 | Sulfuric acid | 0.00 | 84.00 |  | -84.00 |
| 2019 | Tetracyanoethylene | -2996.00 | -2902.40 | -2880.90 | -93.60 |
| 2020 | Biphenylene | -6198.80 | -6104.60 | -6103.20 | -94.20 |
| 2021 | 2,2-Dinitropropane | -1846.00 | -1941.60 | -1959.70 | 95.60 |
| 2022 | 2-Methyl-2,3,3-trinitrobutane | -2932.70 | -3030.90 |  | 98.20 |
| 2023 | Coronene | -11311.00 | -11416.30 | -11436.00 | 105.30 |
| 2024 | 4-Nitrobenzil | -6702.60 | -6596.10 | -6591.60 | -106.50 |
| 2025 | Tetrabenzonaphthalene | -12897.00 | -12785.20 | -12774.80 | -111.80 |
| 2026 | 2,4-Dinitrophenetol | -3977.50 | -4089.40 | -4093.70 | 111.90 |
| 2027 | 2,2,3,3-Tetranitrobutane | -2453.90 | -2338.00 |  | -115.90 |
| 2028 | Azulene | -5293.00 | -5176.30 | -5175.80 | -116.70 |
| 2029 | Corannulene | -9641.50 | -9513.60 | -9498.50 | -127.90 |
| 2030 | Trimyristin | -27643.70 | -27771.80 | -27778.60 | 128.10 |
| 2031 | 5,6,11,12-Tetraphenyltetracene | -21149.00 | -20985.60 | -20973.10 | -163.40 |
