# Supplementary material for: A Generally Applicable Computer Algorithm Based on the Group Additivity Method for the Calculation of Seven Molecular Descriptors: Heat of Combustion, LogPO/W, LogS, Refractivity, Polarizability, Toxicity and LogBB of Organic Compounds; Scope and Limits of Applicability
Source: Molecules. 2015 Oct 7;20(10):18279–351. doi: 10.3390/molecules201018279 (PMC6332030; doi:10.3390/molecules201018279)
Supplement: Supplementary file 1 [file molecules-20-18279-s001.zip › Experimental vs Calculated LogBB Data Table.doc]

**Experimental vs. Calculated LogBB Data, sorted by absolute Deviation**

| **No.** | **Molecule name** | **logBB** | **logBB calc** | **logBB test** | **Deviation** |
| --- | --- | --- | --- | --- | --- |
|  |  |  |  |  |  |
| 1 | Paracetamol | -0.31 | -0.31 | -0.32 | 0.00 |
| 2 | N-((2-(Difluoromethoxy)-5-(trifluoromethoxy)phenyl)methyl)-2-phenyl-3-piperidinamine | 0.88 | 0.88 |  | 0.00 |
| 3 | Antipyrine | -0.10 | -0.10 | 0.18 | 0.00 |
| 4 | Verapamil | -0.24 | -0.24 | -0.26 | 0.00 |
| 5 | Nor2chlorpromazine | 0.97 | 0.97 | 0.89 | 0.00 |
| 6 | Octane | 0.69 | 0.69 | 0.72 | 0.00 |
| 7 | Cimetidine | -1.42 | -1.42 | -1.38 | 0.00 |
| 8 | Nebivolol | 0.57 | 0.56 | 0.68 | 0.01 |
| 9 | 1,3-Dimethylxanthine | -0.35 | -0.36 | -0.44 | 0.01 |
| 10 | Progesterone | 0.20 | 0.19 | 0.09 | 0.01 |
| 11 | Imatinib | -0.10 | -0.11 | -0.25 | 0.01 |
| 12 | Cardiazole | -0.03 | -0.04 |  | 0.01 |
| 13 | CAS 202590-69-0 | -0.22 | -0.21 | -0.24 | -0.01 |
| 14 | Ro 19-4603 | -0.25 | -0.26 |  | 0.01 |
| 15 | Cyclosporin A | -0.78 | -0.77 | -0.28 | -0.01 |
| 16 | Methylbenzene | 0.37 | 0.35 | 0.35 | 0.02 |
| 17 | Perphenazine | 0.70 | 0.72 | 0.76 | -0.02 |
| 18 | Bretazenil | -0.09 | -0.07 | -0.02 | -0.02 |
| 19 | o-Xylene | 0.41 | 0.43 | 0.41 | -0.02 |
| 20 | 3-Hydroxy-3-ethyl-3-phenylpropionamide | 0.04 | 0.02 | 0.00 | 0.02 |
| 21 | n-Propyl acetate | 0.12 | 0.14 | 0.15 | -0.02 |
| 22 | Ethylene | 0.31 | 0.33 | 0.29 | -0.02 |
| 23 | Mefloquine | 0.63 | 0.65 | 0.60 | -0.02 |
| 24 | Pfizer Compound 07 | 0.63 | 0.65 | 0.62 | -0.02 |
| 25 | Imodium | 0.77 | 0.79 | 0.92 | -0.02 |
| 26 | N-((2-Methoxy-5-(1-methylethyl)phenyl)methyl)-2-phenyl-3-piperidinamine | 0.92 | 0.89 | 0.96 | 0.03 |
| 27 | 3-(4-Dimethylaminomethyl-2-pyridyl)-N-(3-nitro-2-pyrryl)-aniline | -0.28 | -0.31 | -0.33 | 0.03 |
| 28 | Aminopyrine | 0.00 | -0.03 |  | 0.03 |
| 29 | Xylocaine | 0.34 | 0.31 | 0.19 | 0.03 |
| 30 | Ranitidine | -1.23 | -1.26 |  | 0.03 |
| 31 | Ethylenedichloride | -0.14 | -0.17 | -0.15 | 0.03 |
| 32 | Terfenadine | 0.64 | 0.67 | 0.71 | -0.03 |
| 33 | Thiopental | -0.21 | -0.17 | -0.11 | -0.03 |
| 34 | Flesinoxan | -0.45 | -0.41 | -0.47 | -0.04 |
| 35 | Urea | -0.14 | -0.18 | -0.04 | 0.04 |
| 36 | Warfarin | 0.00 | -0.04 | -0.01 | 0.04 |
| 37 | Carbamazepine | -0.07 | -0.03 | -0.12 | -0.04 |
| 38 | CAS 126347-69-1 | 0.28 | 0.32 | 0.34 | -0.04 |
| 39 | Indinavir | -0.75 | -0.70 | -0.50 | -0.04 |
| 40 | Phenytoin | -0.16 | -0.21 | -0.12 | 0.05 |
| 41 | 1,4-Dimethylbenzene | 0.38 | 0.43 | 0.47 | -0.05 |
| 42 | 1-Hydroxymidazolam | -0.07 | -0.12 | -0.10 | 0.05 |
| 43 | trans-1,2-Dichloroethylene | 0.04 | -0.01 |  | 0.05 |
| 44 | 1-Pentanol | 0.20 | 0.15 | 0.17 | 0.05 |
| 45 | Pfizer Compound 17 | 0.48 | 0.43 | 0.54 | 0.05 |
| 46 | Triazolam | 0.62 | 0.67 | 0.67 | -0.05 |
| 47 | Desmethyldiazepam | 0.50 | 0.55 | 0.43 | -0.05 |
| 48 | t-Butylcyclohexane | 0.61 | 0.55 | 0.72 | 0.06 |
| 49 | Mirtazepine | 0.53 | 0.59 | 0.68 | -0.06 |
| 50 | Daunomycin | -0.80 | -0.86 |  | 0.06 |
| 51 | Vinyl ether | 0.13 | 0.07 | -0.08 | 0.06 |
| 52 | Ethyl ether | 0.00 | 0.06 | 0.01 | -0.06 |
| 53 | Cyanoethylene | -0.40 | -0.46 | -0.49 | 0.06 |
| 54 | Bupropion | 0.40 | 0.46 | 0.59 | -0.06 |
| 55 | Toliprolol | 0.28 | 0.34 | 0.34 | -0.06 |
| 56 | Zolpidem | -0.48 | -0.54 | -0.60 | 0.06 |
| 57 | Mepyramine | 0.49 | 0.55 | 0.49 | -0.06 |
| 58 | Digoxin | -1.23 | -1.17 |  | -0.06 |
| 59 | Theobromine | -0.29 | -0.36 | -0.44 | 0.07 |
| 60 | Pfizer Compound 03 | 0.89 | 0.96 | 0.88 | -0.07 |
| 61 | Theophylline | -0.29 | -0.36 | -0.46 | 0.07 |
| 62 | 5-Heptyl-5-ethylbarbituric acid | 0.02 | 0.09 | 0.14 | -0.07 |
| 63 | Oxirane | 0.01 | -0.06 | -0.10 | 0.07 |
| 64 | Beloxepin | 0.82 | 0.75 | 0.69 | 0.07 |
| 65 | Nelfinavir | -0.93 | -0.86 | -0.76 | -0.07 |
| 66 | Barbital | -0.14 | -0.21 | -0.23 | 0.07 |
| 67 | Promazine sulfoxide | -0.48 | -0.40 |  | -0.08 |
| 68 | n-Butyl acetate | 0.28 | 0.20 | 0.22 | 0.08 |
| 69 | Isopentyl alcohol | 0.04 | 0.12 | 0.13 | -0.08 |
| 70 | 1-(3-Fluoropropyl)-2-nitroimidazole | -0.24 | -0.16 |  | -0.08 |
| 71 | Methanol | 0.02 | -0.06 | 0.04 | 0.08 |
| 72 | Procaine | 0.05 | -0.03 | -0.03 | 0.08 |
| 73 | Ethosuximide | -0.30 | -0.22 | -0.20 | -0.08 |
| 74 | Propylhexedrine | 1.08 | 1.00 | 1.06 | 0.08 |
| 75 | Ethyl acetate | 0.00 | 0.08 | 0.11 | -0.08 |
| 76 | Pindolol | -0.15 | -0.23 | -0.34 | 0.08 |
| 77 | Flunitrazepam | 0.06 | 0.14 |  | -0.08 |
| 78 | 1-Butyl-3-phenylthiourea | 0.04 | 0.12 | 0.16 | -0.08 |
| 79 | Saquinavir | -0.97 | -1.05 | -1.10 | 0.08 |
| 80 | Trifluopromazine | 1.44 | 1.35 | 1.34 | 0.09 |
| 81 | Rivastigmine | 0.88 | 0.97 | 1.23 | -0.09 |
| 82 | Org 32104 | 0.52 | 0.61 | 0.66 | -0.09 |
| 83 | Allobarbital | -0.22 | -0.31 | -0.34 | 0.09 |
| 84 | 5-Ethyl-5-octylbarbituric acid | 0.24 | 0.15 | 0.18 | 0.09 |
| 85 | Ritaserin | 0.30 | 0.21 | 0.24 | 0.09 |
| 86 | Pergolide | 0.30 | 0.39 | 0.44 | -0.09 |
| 87 | Triptanol | 0.89 | 0.98 | 1.03 | -0.09 |
| 88 | Promazine | 1.23 | 1.13 | 1.18 | 0.10 |
| 89 | Corticosterone | -0.50 | -0.40 | -0.48 | -0.10 |
| 90 | Benzene | 0.37 | 0.27 | 0.30 | 0.10 |
| 91 | Pfizer Compound 24 | 0.62 | 0.52 | 0.57 | 0.10 |
| 92 | Valium | 0.52 | 0.62 | 0.65 | -0.10 |
| 93 | Amylobarbitone | 0.04 | -0.06 | -0.08 | 0.10 |
| 94 | BSP | 0.07 | -0.03 | -0.20 | 0.10 |
| 95 | 3-Nitro-2-(4-(2-pyridyl)-3-thiabutyl)amino-pyrrole | -0.66 | -0.76 | -0.62 | 0.10 |
| 96 | 3-(1-Methyl-2-pyrrolidinyl)pyridine | 0.35 | 0.24 | 0.03 | 0.11 |
| 97 | Naringenin | -0.80 | -0.69 | -0.61 | -0.11 |
| 98 | Prucalopride | -0.10 | -0.21 | -0.32 | 0.11 |
| 99 | 1-Butanol | -0.02 | 0.09 | 0.10 | -0.11 |
| 100 | PKKB11 | 0.00 | -0.11 | -0.12 | 0.11 |
| 101 | Pseudococaine | 0.60 | 0.49 | 0.44 | 0.11 |
| 102 | Scopolamine | 0.23 | 0.34 | 0.50 | -0.11 |
| 103 | Atomoxetine | 0.70 | 0.81 | 0.85 | -0.11 |
| 104 | Hexobarbital | -0.02 | -0.14 | -0.15 | 0.12 |
| 105 | Pfizer Compound 19 | 0.49 | 0.61 | 0.85 | -0.12 |
| 106 | PKKB04 | 0.00 | -0.12 | -0.13 | 0.12 |
| 107 | 5-Amyl-5-ethylbarbituric acid | 0.09 | -0.03 | -0.04 | 0.12 |
| 108 | cis-1,2-Dichloroethylene | -0.13 | -0.01 | 0.09 | -0.12 |
| 109 | Fluroxene | 0.13 | 0.25 | 0.28 | -0.12 |
| 110 | Midazolam | 0.36 | 0.24 | 0.19 | 0.12 |
| 111 | Biperiden | 0.85 | 0.73 | 0.78 | 0.12 |
| 112 | Tolbutamide | -0.70 | -0.58 |  | -0.12 |
| 113 | 1-Hexyl-4-(4-iodophenyl)piperazine | 1.01 | 1.13 | 1.14 | -0.12 |
| 114 | Cotinine | -0.25 | -0.13 | -0.05 | -0.12 |
| 115 | 4-(-3-(N'-Cyano-N''-methylguanylphenyl)-2-guanylthiazole | -1.54 | -1.67 | -1.92 | 0.13 |
| 116 | Isopropylacetate | 0.40 | 0.27 | 0.23 | 0.13 |
| 117 | Risperidal | -0.02 | -0.15 | -0.24 | 0.13 |
| 118 | Ethanol | -0.16 | -0.03 | -0.07 | -0.13 |
| 119 | Testosterone | 0.00 | 0.13 | 0.38 | -0.13 |
| 120 | t-Butylbenzene | 0.43 | 0.30 | 0.29 | 0.13 |
| 121 | 2-(4-(3-Bromo-2-pyridyl)-3-thiabutyl)amino-3-nitropyrrole | -0.67 | -0.53 | -0.51 | -0.14 |
| 122 | CAS 155428-00-5 | -0.01 | -0.15 | -0.15 | 0.14 |
| 123 | Pyrene | 0.23 | 0.37 | 1.93 | -0.14 |
| 124 | Acebutolol | -0.15 | -0.01 | -0.01 | -0.14 |
| 125 | 2-Chloro-1,1,1-trifluoroethane | 0.08 | 0.22 | 0.23 | -0.14 |
| 126 | 2-(1-Methylpiperidin-4-yl)-6-(2-phenylpyrazolo[1,5-a]pyridin-3-yl)pyridazin-3(2H)-one | 0.06 | -0.08 | -0.08 | 0.14 |
| 127 | Reminyl | 0.32 | 0.18 | 0.22 | 0.14 |
| 128 | Decane | 0.67 | 0.81 | 0.85 | -0.14 |
| 129 | Colchicine | -0.80 | -0.94 | -0.93 | 0.14 |
| 130 | SNC 121 | 0.80 | 0.65 | 0.47 | 0.15 |
| 131 | Thioridazine | 0.46 | 0.61 | 0.71 | -0.15 |
| 132 | N-(2,6-Dimethyl-4-bromophenyl)-4,5-dihydro-1H-imidazole-2-amine | -0.28 | -0.13 | -0.16 | -0.15 |
| 133 | Tacrine | -0.13 | 0.02 | -0.28 | -0.15 |
| 134 | Compound 13 | -0.23 | -0.38 | -0.46 | 0.15 |
| 135 | Styrene | 0.45 | 0.30 | 0.32 | 0.15 |
| 136 | CP 99994 | 0.87 | 0.72 | 0.77 | 0.15 |
| 137 | Ethylbenzene | 0.26 | 0.41 | 0.39 | -0.15 |
| 138 | Phencyclidine | 0.52 | 0.67 | 0.72 | -0.15 |
| 139 | Cyclobarbital | -0.30 | -0.15 | -0.10 | -0.15 |
| 140 | Thioperamide | -0.16 | -0.32 | -0.48 | 0.16 |
| 141 | Oxazepam | 0.61 | 0.45 | 0.35 | 0.16 |
| 142 | Paclitaxel | -0.60 | -0.76 | -0.86 | 0.16 |
| 143 | Caprylene | 0.74 | 0.58 | 0.56 | 0.16 |
| 144 | YH1885 | 0.67 | 0.84 | 1.12 | -0.17 |
| 145 | Phenobarbital | -0.12 | -0.29 | -0.32 | 0.17 |
| 146 | Roxatidine benzoate | -0.24 | -0.07 | -0.09 | -0.17 |
| 147 | Fluconazole | -0.22 | -0.05 | 0.67 | -0.17 |
| 148 | Levobetaxolol | 0.39 | 0.22 | 0.28 | 0.17 |
| 149 | 4-Iodo-N1,N3-bis(1-methylpropyl)-1,3-benzenediamine | 0.64 | 0.81 | 1.11 | -0.17 |
| 150 | Ethenzamide | -0.05 | -0.22 | -0.19 | 0.17 |
| 151 | Bisphenol A | -0.12 | -0.30 | -0.25 | 0.18 |
| 152 | 2-(3-(3-(1-Piperidylmethyl)phenoxy)-1-propyl)aminothiazole | 0.44 | 0.26 | 0.26 | 0.18 |
| 153 | Trifluoperazine | 1.44 | 1.26 | 1.29 | 0.18 |
| 154 | PKKB10 | 0.25 | 0.43 | 0.40 | -0.18 |
| 155 | Ropinirole | 0.25 | 0.43 | 0.41 | -0.18 |
| 156 | Heptane | 0.81 | 0.63 | 0.68 | 0.18 |
| 157 | Pentobarbital | 0.12 | -0.06 | -0.03 | 0.18 |
| 158 | Pervitin | 0.93 | 0.75 | 0.72 | 0.18 |
| 159 | Methyl acetate | -0.13 | 0.05 | 0.11 | -0.18 |
| 160 | Propofol | 0.48 | 0.30 | 0.30 | 0.18 |
| 161 | Amoxapine | 0.60 | 0.42 | 0.40 | 0.18 |
| 162 | N-((5-(1,1-dimethylethyl)-2-(trifluoromethoxy)phenyl)methyl)-2-phenyl-3-piperidinamine | 0.96 | 0.77 | 0.63 | 0.19 |
| 163 | Levocabastine | -0.70 | -0.51 | -0.44 | -0.19 |
| 164 | 1-Propanol | -0.16 | 0.03 | 0.03 | -0.19 |
| 165 | Flurbiprofen | 0.30 | 0.11 | 0.15 | 0.19 |
| 166 | Alaptide | -0.26 | -0.45 | -0.56 | 0.19 |
| 167 | DM44 | -1.00 | -0.80 |  | -0.20 |
| 168 | 2-Methylheptane | 0.86 | 0.66 | 0.68 | 0.20 |
| 169 | Dopa | -0.77 | -0.97 | -1.14 | 0.20 |
| 170 | Epiquinine | -0.01 | 0.19 | 0.18 | -0.20 |
| 171 | 1,1-Dichloroethane | -0.28 | -0.07 |  | -0.21 |
| 172 | m-Xylene | 0.22 | 0.43 | 0.41 | -0.21 |
| 173 | Naproxen | 0.10 | -0.11 | 0.42 | 0.21 |
| 174 | Lormetazepam | 0.47 | 0.68 | 0.84 | -0.21 |
| 175 | Norchlorpromazine | 1.37 | 1.15 | 1.10 | 0.22 |
| 176 | Glibenclamide | -0.90 | -0.68 |  | -0.22 |
| 177 | Aspirin | -0.50 | -0.28 | -0.23 | -0.22 |
| 178 | BMS-184111 | 0.74 | 0.52 |  | 0.22 |
| 179 | 1-Nonene | 0.86 | 0.64 | 0.71 | 0.22 |
| 180 | Hydroxyzine | 0.39 | 0.17 | 0.13 | 0.22 |
| 181 | BPSP | -0.01 | 0.21 | 0.21 | -0.22 |
| 182 | Nornicotine | 0.32 | 0.10 | 0.04 | 0.22 |
| 183 | Lamisil | 0.09 | 0.31 |  | -0.22 |
| 184 | 1,1,2-Trichloroethane | -0.10 | -0.32 |  | 0.22 |
| 185 | 3-(3-(1-Piperidylmethyl)phenoxy-1-propanol | -0.02 | 0.20 | 0.21 | -0.22 |
| 186 | Hexane | 0.80 | 0.57 | 0.54 | 0.23 |
| 187 | Asenapin | 1.03 | 0.80 | 0.80 | 0.23 |
| 188 | Caffeine | -0.06 | -0.29 | -0.31 | 0.23 |
| 189 | Fentanyl | 0.56 | 0.33 | 0.44 | 0.23 |
| 190 | 2-Methyl-1-propanol | -0.17 | 0.06 | 0.05 | -0.23 |
| 191 | N-((5-(1,1-Dimethylethyl)-2-methoxyphenyl)methyl)-2-phenylpiperidinamine | 0.98 | 0.75 | 0.64 | 0.23 |
| 192 | Nonane | 0.52 | 0.75 | 0.82 | -0.23 |
| 193 | 2',3'-Dideoxy-3'-(hydroxymethyl)cytidine | -0.79 | -1.02 | -1.04 | 0.23 |
| 194 | 4-((4-Chlorophenyl)amino)-6-methyl-2-oxo-3-cyclohexene-1-carboxylic acid, methyl ester | -0.96 | -0.73 | -1.05 | -0.23 |
| 195 | Chlorpromazine | 1.06 | 1.29 | 1.50 | -0.23 |
| 196 | Accutane | -0.49 | -0.26 | -0.18 | -0.23 |
| 197 | 13-cis-Retinoic acid | -0.49 | -0.26 | -0.29 | -0.23 |
| 198 | 4-Hydroxymidazolam | -0.10 | 0.14 | 0.34 | -0.24 |
| 199 | Loxapine | 0.80 | 0.56 | 0.39 | 0.24 |
| 200 | Methadone | 0.90 | 0.66 | 0.65 | 0.24 |
| 201 | Clozapine | 0.70 | 0.46 | 0.38 | 0.24 |
| 202 | Methohexital | -0.06 | -0.30 | -0.37 | 0.24 |
| 203 | 5-Ethyl-5-propylbarbituric acid | 0.09 | -0.15 | -0.10 | 0.24 |
| 204 | ORG30526 | 0.39 | 0.63 | 0.59 | -0.24 |
| 205 | PKKB05 | 1.44 | 1.20 | 1.15 | 0.24 |
| 206 | 2-Hydroxydesmethylimipramine | 0.53 | 0.28 | 0.24 | 0.25 |
| 207 | U69593 | 0.60 | 0.85 | 0.67 | -0.25 |
| 208 | Pentane | 0.76 | 0.51 | 0.54 | 0.25 |
| 209 | SB-222200 | 0.30 | 0.05 | -0.26 | 0.25 |
| 210 | Sabeluzole | 0.78 | 1.03 | 0.98 | -0.25 |
| 211 | Benzodiazepine 34 | -1.34 | -1.08 | -0.97 | -0.26 |
| 212 | 1-Decene | 0.96 | 0.70 | 0.72 | 0.26 |
| 213 | t-Butylethylether | 0.22 | 0.48 | 0.64 | -0.26 |
| 214 | Norverapamil | -0.64 | -0.38 | -0.21 | -0.26 |
| 215 | Methyl-n-propylketone | -0.01 | 0.25 | 0.31 | -0.26 |
| 216 | Icotidine | -2.00 | -1.74 | -1.82 | -0.26 |
| 217 | 2-Methyloctane | 0.98 | 0.72 | 0.77 | 0.26 |
| 218 | Pentazocine | 0.55 | 0.28 | 0.20 | 0.26 |
| 219 | 5-Ethyl-5-methylbarbituric acid | 0.00 | -0.27 | -0.27 | 0.27 |
| 220 | 2-Methylnonane | 1.05 | 0.78 | 0.75 | 0.27 |
| 221 | 4-Benzyl-2-(4-(5-dimethylaminomethyl-2-furanyl)-3-thiabutyl)amino-3-nitropyrrole | -0.73 | -0.46 | -0.45 | -0.27 |
| 222 | 2-Butanone | -0.08 | 0.19 | 0.22 | -0.27 |
| 223 | N-Desmethylasenapine | 0.39 | 0.66 | 0.74 | -0.27 |
| 224 | Northioridazine | 0.75 | 0.47 | 0.34 | 0.28 |
| 225 | Isobutyl acetate | 0.45 | 0.17 | 0.17 | 0.28 |
| 226 | Pethidine | 0.60 | 0.32 | 0.25 | 0.28 |
| 227 | Butobarbitone | 0.19 | -0.09 | -0.06 | 0.28 |
| 228 | Acetone | -0.15 | 0.13 | 0.16 | -0.28 |
| 229 | Cyclopropane | 0.11 | 0.39 | 0.41 | -0.28 |
| 230 | 2-Methyl-2-propanol | 0.11 | 0.39 | 0.42 | -0.28 |
| 231 | 6-Methyl-4-((3-nitrophenyl)amino)-2-oxo-3-cyclohexene-1-carboxylic acid, methyl ester | -1.00 | -1.28 |  | 0.28 |
| 232 | Cidoxepin | 1.00 | 0.72 | 0.73 | 0.28 |
| 233 | Nalidixic acid | -0.66 | -0.94 | -1.24 | 0.28 |
| 234 | 2-(5-(5-Dimethylaminomethyl-2-furanyl)-1-aza-4-thiapentyl)-5-(2-naphthylmethyl)-4-pyrimidone | -1.30 | -1.01 | -0.32 | -0.29 |
| 235 | Amyl acetate | 0.55 | 0.26 | 0.26 | 0.29 |
| 236 | 9-Hydroxyrisperidone | -0.67 | -0.38 | -0.21 | -0.29 |
| 237 | N,N-Dimethyl-2-pyridineethanamine | -0.06 | 0.23 | 0.25 | -0.29 |
| 238 | PKKB14 | 0.39 | 0.68 | 0.70 | -0.29 |
| 239 | Doxylamine | 0.64 | 0.34 | 0.29 | 0.30 |
| 240 | 2-(1,2-Benzisoxazol-3-yl)-alpha-2-propenylbenzeneamine | 0.00 | 0.30 | 0.06 | -0.30 |
| 241 | 3-Methylhexane | 0.90 | 0.60 | 0.61 | 0.30 |
| 242 | Escitalopram | 0.10 | 0.40 | 0.62 | -0.30 |
| 243 | Etoposide | -2.00 | -1.70 |  | -0.30 |
| 244 | DM49 | -0.17 | -0.48 | -0.71 | 0.31 |
| 245 | Quinalbarbitone | 0.20 | -0.11 | -0.10 | 0.31 |
| 246 | Carteolol | -0.40 | -0.09 | -0.21 | -0.31 |
| 247 | 2-Propanol | -0.15 | 0.16 | 0.14 | -0.31 |
| 248 | Codeine | 0.55 | 0.24 | 0.12 | 0.31 |
| 249 | Clonidine | 0.11 | -0.20 | -0.15 | 0.31 |
| 250 | 1-(8-Fluorooctyl)-2-nitroimidazole | -0.17 | 0.14 |  | -0.31 |
| 251 | FFMZ | -0.09 | -0.40 | -0.54 | 0.31 |
| 252 | Isopentylacetate | 0.55 | 0.23 | 0.21 | 0.32 |
| 253 | PKKB08 | -0.34 | -0.02 | -0.12 | -0.32 |
| 254 | Vinblastine | -0.07 | -0.39 | -0.86 | 0.32 |
| 255 | 2-Guanyl-4-(3-aminophenyl)thiazole | -1.15 | -0.82 | -0.68 | -0.33 |
| 256 | Flumazenil | -0.29 | -0.62 | -0.73 | 0.33 |
| 257 | Hexethal | 0.36 | 0.03 | 0.05 | 0.33 |
| 258 | Compound 5 | -0.31 | -0.64 | -0.67 | 0.33 |
| 259 | Pfizer Compound 22 | 0.32 | 0.65 | 0.77 | -0.33 |
| 260 | Stavudine | -0.48 | -0.81 | -1.00 | 0.33 |
| 261 | 1-(4-Iodophenyl)-4-(1-methylpropyl)piperazine | 1.38 | 1.04 | 1.05 | 0.34 |
| 262 | Sarcolysinum | -0.50 | -0.84 | -1.06 | 0.34 |
| 263 | Bremazocine | 0.60 | 0.26 | 0.16 | 0.34 |
| 264 | t-Amyl methyl ether | 0.17 | 0.51 | 0.54 | -0.34 |
| 265 | Propranolol | 0.64 | 0.30 | 0.32 | 0.34 |
| 266 | Imipramine | 1.07 | 0.73 | 0.77 | 0.34 |
| 267 | Propylene | -0.06 | 0.28 | 0.30 | -0.34 |
| 268 | Mianserin | 0.99 | 0.64 | 0.62 | 0.35 |
| 269 | 1,2,4-Trimethylbenzene | 0.16 | 0.51 | 0.60 | -0.35 |
| 270 | Ibuprofen | -0.18 | 0.17 | 0.24 | -0.35 |
| 271 | Benzedrine | 0.93 | 0.57 | 0.47 | 0.36 |
| 272 | 1,2,4-Trimethylcyclohexane | 1.02 | 0.66 | 0.70 | 0.36 |
| 273 | N-(2,4-Dichlorophenyl)-2-imidazoline-2-amine | 0.16 | -0.20 | -0.21 | 0.36 |
| 274 | Methylcyclohexane | 0.96 | 0.60 | 0.61 | 0.36 |
| 275 | PKKB07 | -0.67 | -1.03 | -1.21 | 0.36 |
| 276 | 2-Guanyl-4-phenylthiazole | -0.18 | -0.54 | -0.61 | 0.36 |
| 277 | Tolonidine | -0.65 | -0.28 | -0.27 | -0.37 |
| 278 | Roxatidine acetate | -0.46 | -0.09 | -0.14 | -0.37 |
| 279 | Dexsotalol | -0.28 | -0.65 |  | 0.37 |
| 280 | Mesoridazine | -0.19 | 0.19 |  | -0.38 |
| 281 | Venlafaxine | 0.60 | 0.98 | 1.00 | -0.38 |
| 282 | 2-(4-Bromo-2,6-dichloroanilino)-2-imidazoline | 0.41 | 0.03 | 0.02 | 0.38 |
| 283 | 2-Propylpentanoic acid | -0.22 | 0.16 | 0.25 | -0.38 |
| 284 | t-Pentyl alcohol | 0.07 | 0.45 | 0.47 | -0.38 |
| 285 | Desmethylclobazam | 0.24 | -0.14 | -0.23 | 0.38 |
| 286 | Fluoromisonidazole | -0.01 | -0.39 |  | 0.38 |
| 287 | Fluoroethylfumazenil | -0.14 | -0.52 | -0.68 | 0.38 |
| 288 | Benzodiazepine 35 | -1.82 | -1.44 | -1.31 | -0.38 |
| 289 | Methylcyclopentane | 0.93 | 0.54 | 0.51 | 0.39 |
| 290 | Maropitant | 0.46 | 0.85 | 0.91 | -0.39 |
| 291 | N-(2,6-Dibromophenyl)-4,5-dihydro-1H-imidazole-2-amine | 0.33 | -0.06 | -0.13 | 0.39 |
| 292 | 3-(5-Dimethylaminomethyl-2-furanyl)-N-(3-nitro-2-pyrryl)-aniline | -0.27 | -0.66 | -0.96 | 0.39 |
| 293 | Desmonomethylpromazine | 0.59 | 0.99 | 1.09 | -0.40 |
| 294 | Pfizer Compound 20 | 0.11 | 0.51 | 0.74 | -0.40 |
| 295 | s-trans-1,3-Butadiene | -0.17 | 0.23 | 0.25 | -0.40 |
| 296 | 1-Isobutyl-4-(4-iodophenyl)piperazine | 1.38 | 0.98 | 0.81 | 0.40 |
| 297 | Sertraline | 1.60 | 1.20 | 1.08 | 0.40 |
| 298 | Flavonol | -1.70 | -2.10 | -2.54 | 0.40 |
| 299 | Ribavirin | -0.67 | -1.07 | -1.10 | 0.40 |
| 300 | 2-(3-(3-(1-Piperidylmethyl)phenoxy)-1-propyl)aminopyridine | 0.69 | 0.28 | 0.08 | 0.41 |
| 301 | 2-(2,4,6-Tribromoanilino)-2-imidazoline | 0.58 | 0.17 | -0.11 | 0.41 |
| 302 | Carbamazepine epoxide | -0.35 | 0.06 | 0.26 | -0.41 |
| 303 | Quinine | 0.60 | 0.19 | -0.19 | 0.41 |
| 304 | Paraxanthine | 0.06 | -0.36 | -0.46 | 0.42 |
| 305 | Salicyluric acid | -0.44 | -0.86 | -0.94 | 0.42 |
| 306 | PKKB01 | -0.24 | 0.18 | 0.11 | -0.42 |
| 307 | N-Methylspiroperidol | 0.46 | 0.04 | 0.07 | 0.42 |
| 308 | Adriamycin | -0.83 | -1.25 |  | 0.42 |
| 309 | Clobazam | 0.35 | -0.07 | -0.23 | 0.42 |
| 310 | Morphine | -0.41 | 0.02 | -0.09 | -0.43 |
| 311 | 2-Methylpentane | 0.97 | 0.54 | 0.51 | 0.43 |
| 312 | Quetiapine | 0.70 | 0.27 | 0.05 | 0.43 |
| 313 | trans-1,2-Dimethylcyclohexane | 1.07 | 0.63 | 0.63 | 0.44 |
| 314 | 4-Iodo-alpha,alpha'-dipiperidino-m-xylene | 0.98 | 1.43 | 1.69 | -0.45 |
| 315 | Liarozole | -1.00 | -0.55 | -0.16 | -0.45 |
| 316 | Prazosin | -0.90 | -0.45 | -0.10 | -0.45 |
| 317 | Benzocaine | 0.27 | -0.18 | -0.24 | 0.45 |
| 318 | PK11195 | 0.48 | 0.03 | -0.18 | 0.45 |
| 319 | Pfizer Compound 04 | 0.37 | 0.82 | 0.90 | -0.45 |
| 320 | 4-Benzyl-3-nitro-2-(4-(2-pyridyl)-3-thiabutyl)amino-pyrrole | -0.12 | -0.58 | -0.68 | 0.46 |
| 321 | N-((2-(1-Methylethoxy)-5-(trifluoromethoxy)phenyl)methyl)-2-phenyl-3-piperidinamine | 0.41 | 0.87 | 1.00 | -0.46 |
| 322 | Esomeprazole | -0.82 | -1.28 |  | 0.46 |
| 323 | Alprazolam | 0.04 | 0.51 | 0.69 | -0.47 |
| 324 | 3-Methylpentane | 1.01 | 0.54 | 0.55 | 0.47 |
| 325 | Rolipram | 0.61 | 0.14 | 0.08 | 0.47 |
| 326 | Daidzein | -0.15 | -0.62 | -0.70 | 0.47 |
| 327 | Pfizer Compound 21 | 0.85 | 0.38 | 0.20 | 0.47 |
| 328 | 1,2,3,4-Tetrahydroquinoline | 0.65 | 0.18 | 0.18 | 0.47 |
| 329 | Cortisol | -0.90 | -0.43 | -0.32 | -0.47 |
| 330 | Maprotiline | 1.30 | 0.82 | 0.71 | 0.48 |
| 331 | 2-(4-(5-Dimethylaminomethyl-2-furanyl)-3-thiabutyl)amino-3-nitropyrrole | -1.12 | -0.64 | -0.43 | -0.48 |
| 332 | Butamben | 0.42 | -0.06 | -0.16 | 0.48 |
| 333 | 1-Chloro-4-(trifluoromethyl)benzene | 0.17 | 0.65 | 0.83 | -0.48 |
| 334 | Lupitidine | -1.06 | -1.54 | -1.69 | 0.48 |
| 335 | Zalcitabine | -1.18 | -0.69 | -0.39 | -0.48 |
| 336 | Donepezil | 0.89 | 0.40 | 0.36 | 0.49 |
| 337 | Alfentanil | 0.00 | -0.50 |  | 0.50 |
| 338 | Pfizer Compound 01 | 0.48 | 0.99 | 1.01 | -0.51 |
| 339 | Trazodone | 0.30 | -0.21 | -0.53 | 0.51 |
| 340 | Betahistine | -0.42 | 0.09 | 0.20 | -0.51 |
| 341 | N-(2,4,6-Trichlorophenyl)-4,5-dihydro-1H-imidazole-2-amine | 0.47 | -0.04 | -0.26 | 0.51 |
| 342 | Lubeluzole | 0.59 | 1.10 | 1.21 | -0.51 |
| 343 | Didanosine | -1.30 | -0.78 | -0.56 | -0.52 |
| 344 | 4-Fluorophenylalanine | -0.80 | -0.28 |  | -0.52 |
| 345 | Spiperone | 0.26 | -0.26 | -0.32 | 0.52 |
| 346 | Levorphanol | 0.00 | 0.52 | 0.56 | -0.52 |
| 347 | 2-Guanyl-4-methylthiazole | -0.04 | -0.56 | -0.72 | 0.52 |
| 348 | Sorbitol | -1.60 | -1.07 | -0.72 | -0.53 |
| 349 | Hyoscyamine | -0.06 | 0.47 | 0.58 | -0.53 |
| 350 | Temelastine | -1.88 | -1.34 | -1.23 | -0.54 |
| 351 | Cyclohexane | 1.11 | 0.57 | 0.55 | 0.54 |
| 352 | Practolol | -0.55 | -0.01 | 0.06 | -0.54 |
| 353 | 4-(2-(Dipropylamino)ethyl)-1,3-dihydro-7-hydroxy-2H-indol-2-one | -0.43 | 0.12 | 0.09 | -0.55 |
| 354 | PKKB09 | -0.43 | 0.12 | 0.13 | -0.55 |
| 355 | Buspirone | 0.48 | -0.07 | -0.47 | 0.55 |
| 356 | 2-(2-Aminoethyl)imidazo[1,2-a]pyridine | -1.40 | -0.82 | -0.49 | -0.58 |
| 357 | Bromoperidol | 1.38 | 0.80 | 0.66 | 0.58 |
| 358 | BESP | -0.43 | 0.15 | 0.31 | -0.58 |
| 359 | Compound 17 | -1.00 | -0.42 | -0.40 | -0.58 |
| 360 | Alprenolol | -0.23 | 0.35 | 0.41 | -0.58 |
| 361 | 2-(2-Methyl-4-chlorophenylamino)-2-imidazoline | -0.87 | -0.28 | -0.32 | -0.59 |
| 362 | Paspertin | -0.70 | -0.11 | -0.05 | -0.59 |
| 363 | Pfizer Compound 15 | 0.36 | -0.23 | -0.14 | 0.59 |
| 364 | Fluoxetine | 0.36 | 0.95 | 1.17 | -0.59 |
| 365 | Desmethyldesipramine | 1.01 | 0.41 | 0.43 | 0.60 |
| 366 | Primidone | -0.07 | -0.67 | -1.25 | 0.60 |
| 367 | Dizocilpine | 1.11 | 0.50 | 0.40 | 0.61 |
| 368 | 6-(2-Phenylpyrazolo[1,5-a]pyridin-3-yl)-2-(2-(1-piperidinyl)ethyl))-3(2H)-pyridazinone | 0.38 | -0.23 | -0.30 | 0.61 |
| 369 | Haloperidol | 1.34 | 0.73 | 0.59 | 0.61 |
| 370 | Desipramine | 1.20 | 0.59 | 0.45 | 0.61 |
| 371 | PKKB02 | -0.46 | 0.16 | 0.19 | -0.62 |
| 372 | Pfizer Compound 16 | -0.22 | 0.40 | 0.34 | -0.62 |
| 373 | Naltrindole | 0.60 | -0.03 | -0.34 | 0.63 |
| 374 | Salicylic acid | -1.10 | -0.46 | -0.51 | -0.64 |
| 375 | Zanapezil | 1.14 | 0.50 | 0.46 | 0.64 |
| 376 | Darifenacin | -0.62 | 0.03 | 0.13 | -0.65 |
| 377 | Phenylbutazone | -0.52 | 0.14 | 1.35 | -0.66 |
| 378 | 2-[[2-Amino-4-(2-fluoroethyl)phenyl]thio]-N,N-dimethylbenzenamine | 0.55 | 1.22 | 1.84 | -0.67 |
| 379 | Propyl 4-aminobenzoate | 0.55 | -0.12 | -0.18 | 0.67 |
| 380 | Levosalbutamol | -1.03 | -0.35 | -0.17 | -0.68 |
| 381 | Neohexane | 1.04 | 0.34 | 0.27 | 0.70 |
| 382 | DM5 | -0.95 | -0.25 | -0.10 | -0.70 |
| 383 | Oxycodone | -0.30 | 0.40 | 0.54 | -0.70 |
| 384 | Cytisine | -1.09 | -0.36 | -0.30 | -0.73 |
| 385 | Triflumethazine | 1.52 | 0.78 | 0.55 | 0.74 |
| 386 | PKKB15 | 1.20 | 0.46 | 0.47 | 0.74 |
| 387 | FG-8205 | -0.30 | -1.05 | -1.37 | 0.75 |
| 388 | 4-(3-Acetylaminophenyl)-2-guanylthiazole | -1.57 | -0.81 | -0.62 | -0.76 |
| 389 | Glycyrrhetinic acid | -1.40 | -0.63 | 0.20 | -0.77 |
| 390 | Indometacin | -1.26 | -0.48 | -0.40 | -0.78 |
| 391 | Fluvastatin | 0.10 | -0.68 | -1.09 | 0.78 |
| 392 | Terbutylchloroambucil | 1.00 | 0.21 | -0.17 | 0.79 |
| 393 | Lovastatin acid | 0.70 | -0.10 | -0.27 | 0.80 |
| 394 | Zolantidine | 0.14 | 0.94 | 1.36 | -0.80 |
| 395 | Pfizer Compound 23 | 1.26 | 0.45 | 0.36 | 0.81 |
| 396 | Atenolol | -0.92 | -0.11 | -0.06 | -0.81 |
| 397 | BMSP | -0.55 | 0.27 | 0.31 | -0.82 |
| 398 | Nevirapine | 0.00 | -0.82 | -0.82 | 0.82 |
| 399 | 2-Thiazolylethylamine | -1.40 | -0.57 | -0.29 | -0.83 |
| 400 | Tiotidine | -0.82 | -1.65 | -2.07 | 0.83 |
| 401 | Gentisic acid | 0.08 | -0.77 | -0.88 | 0.85 |
| 402 | 4-Phenyl-2-thiazoleethanamine | -1.30 | -0.42 | -0.29 | -0.88 |
| 403 | Apaxifylline | -1.40 | -0.48 | -0.25 | -0.92 |
| 404 | 2-(2,4-Dimethylphenylimino)imidazolidine | -1.30 | -0.36 | -0.21 | -0.94 |
| 405 | Bunitrolol | 0.38 | -0.56 | -0.96 | 0.94 |
| 406 | N-(2-Methylphenyl)-2-imidazolidinimine | -1.39 | -0.44 | -0.37 | -0.95 |
| 407 | N-(4-(3-Bromo-2-pyridyl)-3-thiabutyl)-N'-cyano-N''-ethylguanine | -2.15 | -1.20 | -0.61 | -0.95 |
| 408 | PKKB13 | -1.26 | -0.26 | -0.17 | -1.00 |
| 409 | Metoprolol | 1.15 | 0.10 | -0.01 | 1.05 |
| 410 | Motilium | -0.78 | 0.27 | 0.90 | -1.05 |
| 411 | Etodolac | -1.34 | -0.14 | 0.12 | -1.20 |
| 412 | p-Phenylbenzoic acid | -1.26 | -0.05 | -0.04 | -1.21 |
| 413 | Chloroambucil | -1.70 | -0.46 | 0.06 | -1.24 |
