# Supplementary material for: A Generally Applicable Computer Algorithm Based on the Group Additivity Method for the Calculation of Seven Molecular Descriptors: Heat of Combustion, LogPO/W, LogS, Refractivity, Polarizability, Toxicity and LogBB of Organic Compounds; Scope and Limits of Applicability
Source: Molecules. 2015 Oct 7;20(10):18279–351. doi: 10.3390/molecules201018279 (PMC6332030; doi:10.3390/molecules201018279)
Supplement: Supplementary file 1 [file molecules-20-18279-s001.zip › Experimental vs Calculated LogP Data Table.doc]

**Experimental vs. Calculated LogP Data, sorted by absolute Deviation**

| **No.** | **Molecule name** | **logP** | **logP calc** | **logP test** | **Deviation** |
| --- | --- | --- | --- | --- | --- |
|  |  |  |  |  |  |
| 1 | Azinphos-methyl | 2.75 | 2.75 |  | 0.00 |
| 2 | Biperiden | 4.25 | 4.25 | 4.26 | 0.00 |
| 3 | 4-Cyanobenzenesulfonamide | 0.23 | 0.23 | 0.19 | 0.00 |
| 4 | Dexindoprofen | 2.77 | 2.77 | 2.76 | 0.00 |
| 5 | 1-(4-Pyridinyl)ethanone | 0.54 | 0.54 | 0.58 | 0.00 |
| 6 | 4-Ethylaniline | 1.96 | 1.96 | 1.94 | 0.00 |
| 7 | 3-(4-Pyridyl)propanol | 0.58 | 0.58 | 0.61 | 0.00 |
| 8 | Gentisic acid | 1.18 | 1.18 | 1.12 | 0.00 |
| 9 | 2,3,4'-Trichlorobiphenyl | 5.42 | 5.42 | 5.36 | 0.00 |
| 10 | 2-Phenylquinoline | 3.90 | 3.90 | 3.97 | 0.00 |
| 11 | 1,4-Dimethylbenzene | 3.15 | 3.15 | 3.19 | 0.00 |
| 12 | Ethyl bromide | 1.61 | 1.61 | 1.60 | 0.00 |
| 13 | 4-Methylbiphenyl | 4.63 | 4.63 | 4.67 | 0.00 |
| 14 | 4,5-Dichloroveratrole | 3.11 | 3.11 | 3.09 | 0.00 |
| 15 | 6-Isopropyl-3-methylphenyl N-methylcarbamate | 2.84 | 2.84 | 2.80 | 0.00 |
| 16 | Hexafluoro-2-propanol | 1.66 | 1.66 | 1.59 | 0.00 |
| 17 | Fenthion | 4.09 | 4.09 | 4.13 | 0.00 |
| 18 | 6-Methylquinoline | 2.57 | 2.57 | 2.59 | 0.00 |
| 19 | Endrin | 5.20 | 5.20 | 5.21 | 0.00 |
| 20 | 2,4-Dichloro-beta-nitrostyrene | 3.26 | 3.26 | 3.35 | 0.00 |
| 21 | Mepindolol | 2.30 | 2.30 | 2.28 | 0.00 |
| 22 | 2,2',3,3',5,5'-Hexachlorobiphenyl | 7.07 | 7.07 | 7.01 | 0.00 |
| 23 | Trifluopromazine | 5.19 | 5.19 | 5.18 | 0.00 |
| 24 | trans-Stilbene | 4.81 | 4.81 | 4.85 | 0.00 |
| 25 | 2,3,5,6-Tetrachlorobiphenyl | 5.96 | 5.97 | 5.95 | -0.01 |
| 26 | Propofol | 3.79 | 3.78 | 3.78 | 0.01 |
| 27 | 2,4,5-Trimethylphenyl N-methylcarbamate | 2.52 | 2.51 | 2.54 | 0.01 |
| 28 | Benzothiazole | 2.02 | 2.01 | 1.91 | 0.01 |
| 29 | 1-Phenylbutane-1,3-dione | 2.52 | 2.51 | 2.55 | 0.01 |
| 30 | 2-Methylanthracene | 5.00 | 5.01 | 4.97 | -0.01 |
| 31 | Metosulam | 2.50 | 2.49 |  | 0.01 |
| 32 | Phenthoate | 3.69 | 3.68 | 3.68 | 0.01 |
| 33 | 4-Nitrotoluene | 2.42 | 2.41 | 2.45 | 0.01 |
| 34 | 3-Methylthiophenoxyacetic acid | 1.90 | 1.89 | 1.89 | 0.01 |
| 35 | 1,1,2-Trichloroethane | 1.89 | 1.88 | 1.92 | 0.01 |
| 36 | Oxacillin | 2.38 | 2.37 | 2.51 | 0.01 |
| 37 | Dexchlorpheniramine | 3.39 | 3.40 | 3.38 | -0.01 |
| 38 | 4-Chloro-3-nitrophenoxyacetic acid | 1.85 | 1.84 | 1.86 | 0.01 |
| 39 | Phenylthioacetic acid | 1.91 | 1.92 | 1.96 | -0.01 |
| 40 | 4-Chloroaniline | 1.83 | 1.82 | 1.85 | 0.01 |
| 41 | 4-Acetylanisole | 1.82 | 1.81 | 1.76 | 0.01 |
| 42 | 3-Isopropyl-4-hydroxyacetanilide | 2.20 | 2.21 | 2.22 | -0.01 |
| 43 | 4-Hexylresorcinol | 3.45 | 3.46 | 3.40 | -0.01 |
| 44 | Propanal | 0.59 | 0.60 | 0.58 | -0.01 |
| 45 | 2-Aminoquinazolin-4-one | 0.60 | 0.61 | 0.68 | -0.01 |
| 46 | Acetaldoxime | -0.18 | -0.17 | -0.17 | -0.01 |
| 47 | 2-(3-Pyridyl)ethylamine | 0.00 | 0.01 | 0.00 | -0.01 |
| 48 | 4-(Acetylamino)benzamide | 0.01 | 0.02 | 0.04 | -0.01 |
| 49 | 1-Ethyl-1-hydroxyurea | -0.10 | -0.11 |  | 0.01 |
| 50 | 3-Ethoxyphenyl N-methylcarbamate | 1.75 | 1.74 | 1.75 | 0.01 |
| 51 | Iodoacetamide | -0.19 | -0.20 |  | 0.01 |
| 52 | Enalapril | 0.16 | 0.17 | 0.37 | -0.01 |
| 53 | 2,6-Dimethyl-N-nitrosomorpholine | 0.32 | 0.33 | 0.35 | -0.01 |
| 54 | Sulfisoxazole | 1.01 | 1.02 |  | -0.01 |
| 55 | DDD | 6.22 | 6.21 | 6.17 | 0.01 |
| 56 | 2-Ethyltoluene | 3.53 | 3.54 | 3.56 | -0.01 |
| 57 | 3-Chlorophenyl N-methylcarbamate | 2.03 | 2.04 | 2.02 | -0.01 |
| 58 | Trichloromethylthiobenzene | 3.78 | 3.79 |  | -0.01 |
| 59 | 4-(3-Pyridyl)butanol | 0.92 | 0.93 | 0.96 | -0.01 |
| 60 | Methylhydrazine | -1.05 | -1.06 |  | 0.01 |
| 61 | 3-(1-Methyl-2-pyrrolidinyl)pyridine | 1.17 | 1.16 | 1.12 | 0.01 |
| 62 | 4-Aminosalicylic acid 2-tolylester | 3.14 | 3.13 | 3.12 | 0.01 |
| 63 | 2-t-Butylphenyl N-methylcarbamate | 2.65 | 2.66 | 2.67 | -0.01 |
| 64 | Ethylbenzene | 3.15 | 3.16 | 3.15 | -0.01 |
| 65 | 2-Butylthiophenyl N-methylcarbamate | 2.98 | 2.99 | 2.96 | -0.01 |
| 66 | 2,2',5,6'-Tetrachlorobiphenyl | 5.98 | 5.97 | 5.97 | 0.01 |
| 67 | 2,3',4',5'-Tetrachlorobiphenyl | 5.98 | 5.97 | 5.93 | 0.01 |
| 68 | 1,8-Dimethylnaphthalene | 4.26 | 4.27 | 4.20 | -0.01 |
| 69 | 2,4,4'-Tribromodiphenyl ether | 5.80 | 5.81 | 5.82 | -0.01 |
| 70 | 2,3,3',4,6-Pentachlorobiphenyl | 6.51 | 6.52 | 6.48 | -0.01 |
| 71 | Prosulfuron | 2.80 | 2.78 | 2.75 | 0.02 |
| 72 | Phenylisothiocyanate | 3.28 | 3.26 | 3.26 | 0.02 |
| 73 | Ibuprofen | 3.51 | 3.49 | 3.51 | 0.02 |
| 74 | Prometryn | 3.51 | 3.49 |  | 0.02 |
| 75 | Phlorol | 2.47 | 2.45 | 2.46 | 0.02 |
| 76 | 1-Methoxy-3-phenylpropane | 2.70 | 2.68 | 2.62 | 0.02 |
| 77 | 3-Ethylphenyl N-methylcarbamate | 2.20 | 2.18 | 2.18 | 0.02 |
| 78 | 4-Phenylpyridine | 2.45 | 2.43 | 2.45 | 0.02 |
| 79 | 4-Bromophenol | 2.43 | 2.41 | 2.43 | 0.02 |
| 80 | 4-Chloro-2-trifluoromethylbenzimidazole | 2.93 | 2.91 | 2.89 | 0.02 |
| 81 | Benzophenone | 3.18 | 3.16 | 3.16 | 0.02 |
| 82 | 1,2,4-Trifluorobenzene | 2.41 | 2.39 | 2.43 | 0.02 |
| 83 | Chlorbromuron | 3.09 | 3.11 |  | -0.02 |
| 84 | Ketoprofen | 3.16 | 3.14 | 3.15 | 0.02 |
| 85 | 3-Carboxyphenylacetic acid | 1.14 | 1.12 | 1.13 | 0.02 |
| 86 | 3-Nitrophenyl N-methylcarbamate | 1.39 | 1.41 | 1.40 | -0.02 |
| 87 | Hydroxyacetic acid | -1.11 | -1.09 | -1.08 | -0.02 |
| 88 | trans-1-Propenylbenzene | 3.35 | 3.33 | 3.45 | 0.02 |
| 89 | Valine | -2.10 | -2.08 | -2.09 | -0.02 |
| 90 | Viagra | 1.90 | 1.92 | 2.14 | -0.02 |
| 91 | 2,3-Xylenol | 2.42 | 2.44 | 2.42 | -0.02 |
| 92 | 2-Methylbenzylalcohol | 1.58 | 1.56 | 1.53 | 0.02 |
| 93 | 1,8-Dichloronaphthalene | 4.19 | 4.21 | 4.19 | -0.02 |
| 94 | Triethylamine | 1.44 | 1.46 | 1.49 | -0.02 |
| 95 | 4-Amino-3-morpholino-6-isopropyl-1,2,4-triazine-5-one | 0.49 | 0.47 | 0.59 | 0.02 |
| 96 | 4-Methyl-N-nitrosopiperidine | 1.05 | 1.03 | 1.01 | 0.02 |
| 97 | 4-Aminopyrimidine | -0.25 | -0.23 | -0.06 | -0.02 |
| 98 | Aminopyrine | 0.85 | 0.87 | 0.85 | -0.02 |
| 99 | Piperidine | 0.85 | 0.87 | 0.93 | -0.02 |
| 100 | N-Methyl-N-nitrosourea | -0.10 | -0.12 | -0.10 | 0.02 |
| 101 | 2-Heptanone | 1.98 | 2.00 | 2.00 | -0.02 |
| 102 | 3-Iodophenyl N-methylcarbamate | 2.52 | 2.50 | 2.46 | 0.02 |
| 103 | Chrysene | 5.73 | 5.75 | 5.84 | -0.02 |
| 104 | Ioversol | -2.98 | -3.00 | -2.99 | 0.02 |
| 105 | Mefoxine | -0.02 | -0.04 | -0.17 | 0.02 |
| 106 | 2-(2-Pyridyl)ethylamine | 0.08 | 0.06 | 0.00 | 0.02 |
| 107 | 1-Naphthylamine | 2.25 | 2.23 | 2.18 | 0.02 |
| 108 | 3-Methoxyformanilide | 1.25 | 1.27 | 1.26 | -0.02 |
| 109 | Vitamin M | -2.50 | -2.48 |  | -0.02 |
| 110 | Threonine | -3.50 | -3.48 | -3.55 | -0.02 |
| 111 | 3-Pyridinemethanamine | -0.32 | -0.34 | -0.34 | 0.02 |
| 112 | 6-Cyanoquinoxaline | 1.01 | 1.03 | 1.05 | -0.02 |
| 113 | Benzedrine | 1.76 | 1.78 | 1.76 | -0.02 |
| 114 | N,N-Dimethyl-N'-(4-nitrophenyl)urea | 1.51 | 1.53 | 1.57 | -0.02 |
| 115 | Acetylthiobenzene | 2.23 | 2.21 | 2.16 | 0.02 |
| 116 | 3-Pyridinepropanol | 0.60 | 0.58 | 0.57 | 0.02 |
| 117 | 3-Nitrobenzaldehyde | 1.47 | 1.45 | 1.42 | 0.02 |
| 118 | Ranitidine | 1.28 | 1.30 | 1.42 | -0.02 |
| 119 | 1-Hydroxy-2,3,4,5,6-pentachlorocyclohexane | 2.54 | 2.56 | 2.58 | -0.02 |
| 120 | 4-Aminosalicylic acid 4-bromophenylester | 3.46 | 3.44 | 3.46 | 0.02 |
| 121 | 4-Propionylphenyl N-methylcarbamate | 1.55 | 1.57 | 1.57 | -0.02 |
| 122 | Furan | 1.34 | 1.36 | 1.39 | -0.02 |
| 123 | Trazodone | 2.90 | 2.88 | 3.01 | 0.02 |
| 124 | 1-Methylnaphthalene | 3.87 | 3.89 | 3.92 | -0.02 |
| 125 | beta-Ethyl-beta-nitrostyrene | 2.86 | 2.84 |  | 0.02 |
| 126 | Phenyltrifluoromethylsulfone | 2.68 | 2.70 | 2.84 | -0.02 |
| 127 | Hexabromobenzene | 6.07 | 6.05 | 6.08 | 0.02 |
| 128 | 2-Naphthol | 2.70 | 2.72 | 2.70 | -0.02 |
| 129 | 2,4,4'-Trichlorodiphenyl ether | 5.53 | 5.51 | 5.55 | 0.02 |
| 130 | 2,2,6,6-Tetramethyl-N-nitrosopiperidine | 2.49 | 2.51 | 2.49 | -0.02 |
| 131 | Pentafluorobenzene | 2.53 | 2.55 | 2.58 | -0.02 |
| 132 | 2,2',4,5,5'-PCB | 6.50 | 6.52 | 6.48 | -0.02 |
| 133 | Fenpropimorph | 4.93 | 4.90 | 4.88 | 0.03 |
| 134 | 2,3,3',4',5,5'-Hexachlorobiphenyl | 7.10 | 7.07 | 7.02 | 0.03 |
| 135 | 2,3,3',4,5',6-Hexachlorobiphenyl | 7.10 | 7.07 | 7.08 | 0.03 |
| 136 | 2,2',6,6'-Tetrachlorobiphenyl | 5.94 | 5.97 | 5.93 | -0.03 |
| 137 | 1-Phenyl-2-nitropropene | 2.52 | 2.49 |  | 0.03 |
| 138 | Bifenox | 4.48 | 4.51 | 4.46 | -0.03 |
| 139 | Trifluoroacetanilide | 2.21 | 2.18 | 2.21 | 0.03 |
| 140 | Phthalic anhydride | 1.43 | 1.40 | 1.49 | 0.03 |
| 141 | 4-Bromophenyl N-methylcarbamate | 2.17 | 2.14 | 2.17 | 0.03 |
| 142 | Benzylbromide | 2.92 | 2.89 | 2.86 | 0.03 |
| 143 | Benzimidazole-2-thione | 1.66 | 1.63 | 1.62 | 0.03 |
| 144 | 2-Phenoxyethanol | 1.16 | 1.13 | 1.14 | 0.03 |
| 145 | Levosalbutamol | 1.40 | 1.37 | 1.34 | 0.03 |
| 146 | 3-Chloroanisole | 2.60 | 2.63 | 2.59 | -0.03 |
| 147 | Alprazolam | 4.90 | 4.87 | 5.13 | 0.03 |
| 148 | o-Xylene | 3.12 | 3.15 | 3.18 | -0.03 |
| 149 | Tramadol | 2.63 | 2.60 | 2.63 | 0.03 |
| 150 | 2,3-Dichloropyridine | 2.11 | 2.08 | 2.13 | 0.03 |
| 151 | Anisole | 2.11 | 2.08 | 2.11 | 0.03 |
| 152 | Ethyleneglycol | -1.36 | -1.33 | -1.29 | -0.03 |
| 153 | Phenyl benzoate | 3.59 | 3.56 | 3.62 | 0.03 |
| 154 | 4-Methylbenzylalcohol | 1.59 | 1.56 | 1.53 | 0.03 |
| 155 | Diuron | 2.68 | 2.71 | 2.63 | -0.03 |
| 156 | Ioxynil | 3.43 | 3.46 | 3.43 | -0.03 |
| 157 | Propanil | 3.07 | 3.04 | 3.03 | 0.03 |
| 158 | 2,4-Dichlorodiphenyl ether | 4.93 | 4.96 | 5.01 | -0.03 |
| 159 | 3-Methyl-4-nitroquinoline-1-oxide | 1.06 | 1.03 | 1.00 | 0.03 |
| 160 | Propyne | 0.94 | 0.97 | 1.01 | -0.03 |
| 161 | 2-Bromopropanoic acid | 0.92 | 0.89 | 1.13 | 0.03 |
| 162 | Pyridine-4-nitrile | 0.46 | 0.49 | 0.47 | -0.03 |
| 163 | Allylamine | 0.03 | 0.06 | 0.06 | -0.03 |
| 164 | 4-Methyl-2-pentanol | 1.53 | 1.50 | 1.52 | 0.03 |
| 165 | 4-Nitroanisole | 2.03 | 2.00 | 1.99 | 0.03 |
| 166 | Methimazole | -0.34 | -0.31 |  | -0.03 |
| 167 | Cefmetazole | -0.60 | -0.63 | -0.74 | 0.03 |
| 168 | 4,5-Dibromo-1,2,3-triazole | 2.24 | 2.27 |  | -0.03 |
| 169 | 9-Acridinamine | 2.74 | 2.77 | 2.73 | -0.03 |
| 170 | 4,5-Dichloro-2-trifluoromethylbenzimidazole | 3.49 | 3.46 | 3.42 | 0.03 |
| 171 | 4,6-Dichloro-2-trifluoromethylbenzimidazole | 3.49 | 3.46 | 3.44 | 0.03 |
| 172 | 4-Chlorophenyl N-methylcarbamate | 2.01 | 2.04 | 2.03 | -0.03 |
| 173 | 3-Fluorophenoxyacetic acid | 1.48 | 1.45 | 1.46 | 0.03 |
| 174 | N,N-Dimethyl-N'-(4-tolyl)urea | 1.98 | 1.95 | 1.95 | 0.03 |
| 175 | 4-(4-Pyridyl)butanol | 0.90 | 0.93 | 0.94 | -0.03 |
| 176 | 3-Pentanol | 1.21 | 1.18 | 1.17 | 0.03 |
| 177 | Cathine | 0.67 | 0.70 | 0.68 | -0.03 |
| 178 | Fenbufen | 3.20 | 3.17 | 3.12 | 0.03 |
| 179 | Azelaic acid | 1.57 | 1.60 | 1.61 | -0.03 |
| 180 | Quinoxaline | 1.32 | 1.35 | 1.33 | -0.03 |
| 181 | Desmethyldiazepam | 2.93 | 2.90 | 2.84 | 0.03 |
| 182 | 3-Chloropyridine | 1.33 | 1.36 | 1.37 | -0.03 |
| 183 | Fluoranthene | 5.16 | 5.13 | 5.26 | 0.03 |
| 184 | Phenoxyacetic acid | 1.34 | 1.37 | 1.38 | -0.03 |
| 185 | 3-Bromoquinoline | 2.91 | 2.88 | 2.86 | 0.03 |
| 186 | 3-Methyl-4-phenyl-1,2,5-oxadiazole | 2.59 | 2.62 | 2.69 | -0.03 |
| 187 | Quinaldine | 2.59 | 2.62 | 2.66 | -0.03 |
| 188 | 2-Methylnaphthalene | 3.86 | 3.89 | 3.96 | -0.03 |
| 189 | 8-Methylquinoline | 2.60 | 2.57 | 2.53 | 0.03 |
| 190 | Ethyl phenylacetate | 2.42 | 2.45 | 2.42 | -0.03 |
| 191 | 1,3,5-Trichloro-2-nitrobenzene | 3.69 | 3.72 | 3.74 | -0.03 |
| 192 | 1-Methyl-1-hydroxypentachlorocyclohexane | 2.94 | 2.97 | 2.95 | -0.03 |
| 193 | 3-Ethoxyphenol | 1.98 | 2.01 | 2.01 | -0.03 |
| 194 | Bevantolol | 3.00 | 3.03 | 2.98 | -0.03 |
| 195 | Cyclopropylbenzene | 3.27 | 3.30 | 3.40 | -0.03 |
| 196 | 2,2',3,3',4,6'-Hexachlorobiphenyl | 7.04 | 7.07 | 7.03 | -0.03 |
| 197 | Cinoxate | 2.29 | 2.32 | 2.23 | -0.03 |
| 198 | Tris-BP | 4.39 | 4.35 | 5.10 | 0.04 |
| 199 | 1,2-Dimethylnaphthalene | 4.31 | 4.27 | 4.29 | 0.04 |
| 200 | 2,6-Dimethylnaphthalene | 4.31 | 4.27 | 4.32 | 0.04 |
| 201 | 2-Iodoaniline | 2.32 | 2.28 | 2.26 | 0.04 |
| 202 | 2-Chlorobenzoic acid | 2.05 | 2.01 | 2.04 | 0.04 |
| 203 | Valium | 2.99 | 2.95 | 2.88 | 0.04 |
| 204 | 2-Chlorodiphenyl ether | 4.45 | 4.41 | 4.43 | 0.04 |
| 205 | Clotrimazole | 5.20 | 5.16 | 5.09 | 0.04 |
| 206 | 3-Nitrotoluene | 2.45 | 2.41 | 2.41 | 0.04 |
| 207 | 6-Methoxyquinoline | 2.20 | 2.16 | 2.17 | 0.04 |
| 208 | Bitertanol | 4.16 | 4.12 | 4.10 | 0.04 |
| 209 | s-Butylpropylamine | 1.91 | 1.87 | 1.89 | 0.04 |
| 210 | Bromacil | 2.11 | 2.15 | 2.17 | -0.04 |
| 211 | Hydrocinnamyl alcohol | 1.88 | 1.92 | 1.89 | -0.04 |
| 212 | 1-Acetoxy-2,3,4,5,6-pentachlorocyclohexane | 3.40 | 3.44 | 3.41 | -0.04 |
| 213 | 3-Methylbenzylalcohol | 1.60 | 1.56 | 1.60 | 0.04 |
| 214 | 4-Fluorophenoxyacetic acid | 1.41 | 1.45 | 1.45 | -0.04 |
| 215 | 3-Fluoro-5-iodophenoxyacetic acid | 2.42 | 2.46 | 2.47 | -0.04 |
| 216 | 5-t-Butyl-4-hydroxy-2-methylacetanilide | 2.67 | 2.71 | 2.74 | -0.04 |
| 217 | Parathion | 3.83 | 3.79 | 3.79 | 0.04 |
| 218 | Aldosterone | 1.08 | 1.04 | 1.09 | 0.04 |
| 219 | Methyl acrylate | 0.80 | 0.84 | 0.86 | -0.04 |
| 220 | 3-Dimethylaminophenol | 1.57 | 1.53 | 1.46 | 0.04 |
| 221 | Toliprolol | 1.93 | 1.97 | 1.96 | -0.04 |
| 222 | 4-Pyridinemethanamine | -0.38 | -0.34 | -0.34 | -0.04 |
| 223 | Isoleucine | -1.69 | -1.73 | -1.79 | 0.04 |
| 224 | 1-Phenyl-1-propanone | 2.19 | 2.23 | 2.24 | -0.04 |
| 225 | 1H-Purine | -0.37 | -0.33 | -0.46 | -0.04 |
| 226 | Purine | -0.37 | -0.33 | -0.32 | -0.04 |
| 227 | Phthiocol | 1.20 | 1.24 | 1.14 | -0.04 |
| 228 | Sulfathiazole | 0.05 | 0.09 | 0.02 | -0.04 |
| 229 | Benz[a]anthracene | 5.79 | 5.75 | 5.92 | 0.04 |
| 230 | 4-Iodophenyl N-methylcarbamate | 2.46 | 2.50 | 2.51 | -0.04 |
| 231 | 4-Formylphenoxyacetic acid | 0.79 | 0.75 | 0.76 | 0.04 |
| 232 | Formic acid | -0.54 | -0.50 | -0.57 | -0.04 |
| 233 | Cefuroxime | -0.16 | -0.12 | -0.07 | -0.04 |
| 234 | Chloroacetic acid | 0.22 | 0.18 | 0.19 | 0.04 |
| 235 | Bufexamac | 1.47 | 1.51 | 1.56 | -0.04 |
| 236 | Flumequine | 1.72 | 1.76 | 1.87 | -0.04 |
| 237 | 3-Methylbenzenesulfonamide | 0.85 | 0.89 | 0.91 | -0.04 |
| 238 | 2-Methyl-1-propanol | 0.65 | 0.61 | 0.60 | 0.04 |
| 239 | 3-(4-Pyridyl)propylamine | 0.40 | 0.36 | 0.38 | 0.04 |
| 240 | Fluorobenzene | 2.27 | 2.23 | 2.21 | 0.04 |
| 241 | Phthalic acid | 0.73 | 0.77 | 0.79 | -0.04 |
| 242 | Methylbenzene | 2.73 | 2.77 | 2.82 | -0.04 |
| 243 | Tinidazole | 0.23 | 0.27 | 0.15 | -0.04 |
| 244 | GBL | 0.64 | 0.60 | 0.65 | 0.04 |
| 245 | 3-Methyl-N-nitrosopiperidine | 0.99 | 1.03 | 1.04 | -0.04 |
| 246 | Tranylcypromine | 1.49 | 1.53 | 1.54 | -0.04 |
| 247 | 2-Pyridinecarbonitrile | 0.50 | 0.54 | 0.50 | -0.04 |
| 248 | Neurontin | -1.25 | -1.21 | -1.27 | -0.04 |
| 249 | 1,4-Pentadiene | 2.48 | 2.44 | 2.44 | 0.04 |
| 250 | Orphenadrine | 3.77 | 3.81 | 3.84 | -0.04 |
| 251 | Pipamperone | 2.02 | 2.06 | 1.99 | -0.04 |
| 252 | Guanine | -0.91 | -0.95 | -0.94 | 0.04 |
| 253 | 2-Tolunitrile | 2.21 | 2.17 | 2.17 | 0.04 |
| 254 | 4-Phenoxybenzoic acid | 3.21 | 3.17 | 3.13 | 0.04 |
| 255 | Ethyl propionate | 1.21 | 1.17 | 1.23 | 0.04 |
| 256 | 4-Chloro-3-cyanophenoxyacetic acid | 1.56 | 1.60 | 1.56 | -0.04 |
| 257 | 7-Bromoquinoline | 2.92 | 2.88 | 2.90 | 0.04 |
| 258 | Benzo[ghi]perylene | 6.63 | 6.59 | 6.46 | 0.04 |
| 259 | 2,6-Dichloroanisole | 3.14 | 3.18 | 3.14 | -0.04 |
| 260 | 2,3',4,4',5-Pentachlorobiphenyl | 6.57 | 6.52 | 6.49 | 0.05 |
| 261 | Isodecyl diphenyl phosphate | 5.70 | 5.65 | 5.60 | 0.05 |
| 262 | 2,2',3,3',6,6'-Hexachlorobiphenyl | 7.12 | 7.07 | 7.03 | 0.05 |
| 263 | 2,2',3,4',5,5'-Hexachlorobiphenyl | 7.12 | 7.07 | 7.01 | 0.05 |
| 264 | Methylcyclopentane | 3.37 | 3.32 | 3.39 | 0.05 |
| 265 | 2-Methylphenoxyacetic acid methylester | 2.08 | 2.03 | 2.08 | 0.05 |
| 266 | Bentazone | 2.83 | 2.78 | 2.79 | 0.05 |
| 267 | Carbomycin | 3.04 | 2.99 | 2.99 | 0.05 |
| 268 | 3-Ethylphenol | 2.50 | 2.45 | 2.45 | 0.05 |
| 269 | 4-Ethylphenol | 2.50 | 2.45 | 2.42 | 0.05 |
| 270 | Desipramine | 4.00 | 3.95 | 3.99 | 0.05 |
| 271 | 4-Ethylphenyl N-methylcarbamate | 2.23 | 2.18 | 2.18 | 0.05 |
| 272 | 3-Methylpyridine | 1.20 | 1.15 | 1.13 | 0.05 |
| 273 | 6-Bromoquinoline | 2.83 | 2.88 | 2.86 | -0.05 |
| 274 | 4-Propylbenzenesulfonamide | 1.64 | 1.59 | 1.59 | 0.05 |
| 275 | 3,4-Dichloronitrobenzene | 3.12 | 3.17 | 3.20 | -0.05 |
| 276 | 4-Chlorobenzaldehyde | 2.13 | 2.08 | 2.08 | 0.05 |
| 277 | Methylcyclohexane | 3.88 | 3.83 | 3.88 | 0.05 |
| 278 | Trimethylamine | 0.16 | 0.11 | 0.09 | 0.05 |
| 279 | 2-Pyridinepropanol | 0.58 | 0.63 | 0.67 | -0.05 |
| 280 | Benzohydroxamic acid | 0.26 | 0.21 | 0.21 | 0.05 |
| 281 | Isopropanolamine | -0.96 | -1.01 | -1.02 | 0.05 |
| 282 | 2-Methylbenzenesulfonamide | 0.84 | 0.89 | 0.84 | -0.05 |
| 283 | 2-Naphthylamine | 2.28 | 2.23 | 2.19 | 0.05 |
| 284 | Quatrimycin | -1.47 | -1.52 | -1.59 | 0.05 |
| 285 | 1-Propanol | 0.34 | 0.29 | 0.30 | 0.05 |
| 286 | Benzaldehyde | 1.48 | 1.53 | 1.52 | -0.05 |
| 287 | 2-(3-Hydroxypropyl)benzimidazole | 1.25 | 1.20 | 1.18 | 0.05 |
| 288 | Nitromethylbenzene | 1.75 | 1.80 | 1.83 | -0.05 |
| 289 | Vitamin B6 | -0.50 | -0.55 | -0.58 | 0.05 |
| 290 | 1,1,1-Trichloroethane | 2.49 | 2.44 | 2.36 | 0.05 |
| 291 | Benzylmethylamine | 1.52 | 1.57 | 1.54 | -0.05 |
| 292 | 5-(2-Pyridyl)pentanol | 1.28 | 1.33 | 1.37 | -0.05 |
| 293 | 4-Fluorophenol | 1.79 | 1.84 | 1.83 | -0.05 |
| 294 | 3-Fluoroaniline | 1.30 | 1.35 | 1.38 | -0.05 |
| 295 | m-Xylene | 3.20 | 3.15 | 3.18 | 0.05 |
| 296 | N-Propylaniline | 2.45 | 2.40 | 2.43 | 0.05 |
| 297 | 1,2-Dimethylindole | 2.82 | 2.87 | 2.84 | -0.05 |
| 298 | Anise alcohol | 1.10 | 1.15 | 1.18 | -0.05 |
| 299 | Vitamin C | -1.85 | -1.90 | -1.94 | 0.05 |
| 300 | 2,2',3,4,4',5-Hexachlorodiphenyl ether | 7.11 | 7.16 | 7.13 | -0.05 |
| 301 | 2,3',4,4',5,5'-Hexachlorodiphenyl ether | 7.11 | 7.16 | 7.17 | -0.05 |
| 302 | 2',3,3',4,4',5,6-Heptabromodiphenyl ether | 8.36 | 8.41 | 8.44 | -0.05 |
| 303 | Benzalmalonodinitrile | 2.12 | 2.07 | 2.04 | 0.05 |
| 304 | 1,2-Dibromoethane | 1.96 | 2.01 | 2.09 | -0.05 |
| 305 | 2,4,6-Trimethylphenol | 2.73 | 2.78 | 2.82 | -0.05 |
| 306 | 6-Chloroquinoline | 2.73 | 2.78 | 2.78 | -0.05 |
| 307 | Methylthiopentachlorocyclohexane | 3.75 | 3.80 | 3.52 | -0.05 |
| 308 | Cymperator | 6.05 | 6.10 | 6.13 | -0.05 |
| 309 | 3-Trifluoromethylphenoxyacetic acid | 2.36 | 2.30 | 2.32 | 0.06 |
| 310 | 4-Iodoaniline | 2.34 | 2.28 | 2.27 | 0.06 |
| 311 | Diphenyl sulfide | 4.45 | 4.51 | 4.41 | -0.06 |
| 312 | 4-Methylacetophenone | 2.28 | 2.22 | 2.24 | 0.06 |
| 313 | s-trans-1,3-Butadiene | 1.99 | 2.05 | 2.05 | -0.06 |
| 314 | 2,3-Dichloroanisole | 3.24 | 3.18 | 3.18 | 0.06 |
| 315 | 1-(2-CF3-phenylhydrazono)-1-cyanoacetic acid methylester | 3.72 | 3.66 | 3.69 | 0.06 |
| 316 | Cyanazine | 2.22 | 2.16 | 2.12 | 0.06 |
| 317 | Phosmet | 2.78 | 2.84 |  | -0.06 |
| 318 | Neburon | 3.80 | 3.86 | 3.78 | -0.06 |
| 319 | 2-Hydroxybenzaldehyde | 1.70 | 1.64 | 1.59 | 0.06 |
| 320 | Benzeneethanamine | 1.41 | 1.35 | 1.33 | 0.06 |
| 321 | 1,1,1,3,3,3-Hexafluoro-2-phenyl-2-propanol | 3.41 | 3.35 | 3.32 | 0.06 |
| 322 | 3,4-Diphenyl-1,2,5-oxadiazole | 3.84 | 3.90 | 4.03 | -0.06 |
| 323 | 1,4-Diiodobenzene | 4.11 | 4.17 | 4.17 | -0.06 |
| 324 | Hexamethylbenzene | 4.61 | 4.67 | 4.71 | -0.06 |
| 325 | 2-Chloroaniline | 1.88 | 1.82 | 1.84 | 0.06 |
| 326 | 3-Chloroaniline | 1.88 | 1.82 | 1.83 | 0.06 |
| 327 | 1,2-Difluorobenzene | 2.37 | 2.31 | 2.33 | 0.06 |
| 328 | Ethyl 2-picolinate | 0.87 | 0.81 | 0.80 | 0.06 |
| 329 | Triazamate | 1.62 | 1.56 | 1.55 | 0.06 |
| 330 | 3-Methylpentane | 3.60 | 3.54 | 3.59 | 0.06 |
| 331 | Bromazepam | 1.65 | 1.71 | 1.74 | -0.06 |
| 332 | Oxamyl | -0.47 | -0.41 | -0.34 | -0.06 |
| 333 | Crotonic acid | 0.72 | 0.66 | 0.69 | 0.06 |
| 334 | Acetylacetone | 0.40 | 0.34 | 0.33 | 0.06 |
| 335 | 4-Cyanoformanilide | 1.08 | 1.02 | 1.00 | 0.06 |
| 336 | N-Nitrosodibutylamine | 1.92 | 1.98 | 2.02 | -0.06 |
| 337 | Benzylpenicillin | 1.83 | 1.77 | 1.83 | 0.06 |
| 338 | 2-Amino-5-phenylvaleric acid | -0.36 | -0.42 | -0.50 | 0.06 |
| 339 | 4-Hydroxybenzamide | 0.25 | 0.19 | 0.14 | 0.06 |
| 340 | Corticosterone | 1.94 | 2.00 | 2.04 | -0.06 |
| 341 | t-Pentylcarbamate | 0.94 | 1.00 | 1.07 | -0.06 |
| 342 | Enprofylline | 0.33 | 0.39 | 0.34 | -0.06 |
| 343 | Sulfaphenazole | 1.52 | 1.46 | 1.67 | 0.06 |
| 344 | Benzamide | 0.64 | 0.58 | 0.61 | 0.06 |
| 345 | Benzo[f]quinoline | 3.25 | 3.19 | 3.15 | 0.06 |
| 346 | Diphenylamine | 3.50 | 3.56 | 3.62 | -0.06 |
| 347 | 2,2',6-Trichlorobiphenyl | 5.48 | 5.42 | 5.37 | 0.06 |
| 348 | Peonol | 1.98 | 1.92 | 1.93 | 0.06 |
| 349 | 4-Phenylbutyric acid methylester | 2.77 | 2.83 | 2.83 | -0.06 |
| 350 | XMC | 2.23 | 2.17 | 2.15 | 0.06 |
| 351 | 4-Amino-3-iodobenzoic acid | 1.53 | 1.59 | 1.59 | -0.06 |
| 352 | 4-Nitrophenyl N-methylcarbamate | 1.47 | 1.41 | 1.43 | 0.06 |
| 353 | Prednisone | 1.47 | 1.41 | 1.38 | 0.06 |
| 354 | 2-Iodophenoxyacetic acid | 2.44 | 2.38 | 2.40 | 0.06 |
| 355 | Dibenzo[ai]anthracene | 6.81 | 6.87 | 6.98 | -0.06 |
| 356 | 1,3-Benzodioxole | 2.08 | 2.14 | 2.13 | -0.06 |
| 357 | 5-Phenylvaleric acid | 2.92 | 2.86 | 2.89 | 0.06 |
| 358 | 2-Bromophenol | 2.35 | 2.41 | 2.43 | -0.06 |
| 359 | 9-Methylanthracene | 5.07 | 5.01 | 5.09 | 0.06 |
| 360 | Diethylstilbestrol | 5.07 | 5.01 | 4.93 | 0.06 |
| 361 | Pethidine | 2.45 | 2.51 | 2.47 | -0.06 |
| 362 | 2,4,4',6-Tetrachlorobiphenyl | 6.03 | 5.97 | 5.93 | 0.06 |
| 363 | Trifluoperazine | 5.03 | 4.97 | 5.02 | 0.06 |
| 364 | Sulforidazine | 4.45 | 4.39 | 4.36 | 0.06 |
| 365 | 2,3',4,4'-Tetrachlorodiphenyl ether | 6.13 | 6.06 | 6.00 | 0.07 |
| 366 | Propham | 2.60 | 2.53 | 2.47 | 0.07 |
| 367 | 1,2,3,4-Tetramethylbenzene | 3.98 | 3.91 | 3.91 | 0.07 |
| 368 | 4-Methylpyridine | 1.22 | 1.15 | 1.17 | 0.07 |
| 369 | 3-Nitrobenzylalcohol | 1.21 | 1.14 | 1.16 | 0.07 |
| 370 | Chlorpyrifos-methyl | 4.31 | 4.38 | 4.30 | -0.07 |
| 371 | 2,4-Dinitrophenol | 1.67 | 1.60 | 1.62 | 0.07 |
| 372 | 2-Nitroethylbenzene | 2.08 | 2.15 | 2.21 | -0.07 |
| 373 | 4-Methylformanilide | 1.61 | 1.68 | 1.66 | -0.07 |
| 374 | 2,2',4,4'-Tetrabromodiphenyl ether | 6.39 | 6.46 | 6.47 | -0.07 |
| 375 | 4-Nitropyrazole | 0.59 | 0.52 |  | 0.07 |
| 376 | 2-Hydroxyacetophenone | 1.92 | 1.99 | 2.00 | -0.07 |
| 377 | Cefixime | 0.11 | 0.18 | 0.01 | -0.07 |
| 378 | Metronidazol | -0.10 | -0.03 | 0.05 | -0.07 |
| 379 | 4-Cyano-2-methylpyridine | 0.81 | 0.88 | 0.95 | -0.07 |
| 380 | 4-Ethylbenzenesulfonamide | 1.31 | 1.24 | 1.24 | 0.07 |
| 381 | 4-Methylbenzenesulfonamide | 0.82 | 0.89 | 0.89 | -0.07 |
| 382 | 2,6-Dibromo-4-nitrophenol | 3.05 | 2.98 | 2.98 | 0.07 |
| 383 | 5-Fluorouracil | -0.95 | -1.02 |  | 0.07 |
| 384 | 2-Fluoroethanol | -0.92 | -0.85 | -0.79 | -0.07 |
| 385 | Dichloroacetic acid | 0.92 | 0.85 | 0.84 | 0.07 |
| 386 | Pyrimidine | -0.40 | -0.47 | -0.49 | 0.07 |
| 387 | 2-Nitrophenoxyacetic acid | 1.22 | 1.29 | 1.29 | -0.07 |
| 388 | 3-Methylphenoxyacetic acid | 1.78 | 1.71 | 1.67 | 0.07 |
| 389 | 4-Methoxyphenoxyacetic acid | 1.23 | 1.30 | 1.29 | -0.07 |
| 390 | Butyraldehyde | 0.88 | 0.95 | 0.93 | -0.07 |
| 391 | 4-CPA | 1.99 | 1.92 | 1.94 | 0.07 |
| 392 | Thioanisole | 2.74 | 2.67 | 2.78 | 0.07 |
| 393 | n-Propyl acetate | 1.24 | 1.17 | 1.16 | 0.07 |
| 394 | Bayleton | 3.26 | 3.33 | 3.35 | -0.07 |
| 395 | Thiourea | -1.02 | -1.09 | -1.07 | 0.07 |
| 396 | 2-s-Butylphenyl N-methylcarbamate | 2.78 | 2.85 | 2.82 | -0.07 |
| 397 | 6-Aminoquinoline | 1.28 | 1.35 | 1.36 | -0.07 |
| 398 | Amiodarone | 7.80 | 7.87 | 7.77 | -0.07 |
| 399 | 9-Anthracenol | 3.61 | 3.68 | 3.62 | -0.07 |
| 400 | 2-Iodobenzoic acid | 2.40 | 2.47 | 2.50 | -0.07 |
| 401 | 1-Methylphenanthrene | 5.08 | 5.01 | 5.10 | 0.07 |
| 402 | 2,4,5-Trichlorodiphenyl ether | 5.58 | 5.51 | 5.45 | 0.07 |
| 403 | 4,5,6-Trichloro-2-trifluoromethylbenzimidazole | 4.08 | 4.01 | 4.00 | 0.07 |
| 404 | N,N-Dimethyl-N'-(4-bromophenyl)urea | 2.19 | 2.26 | 2.25 | -0.07 |
| 405 | 2,3,3',5',6-Pentachlorobiphenyl | 6.45 | 6.52 | 6.47 | -0.07 |
| 406 | 2,4,4',5-Tetrachlorodiphenyl ether | 5.99 | 6.06 | 6.05 | -0.07 |
| 407 | (L)-Arginine | -1.65 | -1.58 |  | -0.07 |
| 408 | 2,2',4,5,5'-Pentabromobiphenyl | 7.10 | 7.02 | 7.01 | 0.08 |
| 409 | 2,3,6-Trimethylnaphthalene | 4.73 | 4.65 | 4.63 | 0.08 |
| 410 | 2,2',3,3',5,6'-Hexachlorobiphenyl | 7.15 | 7.07 | 7.03 | 0.08 |
| 411 | Norflurazon | 2.30 | 2.22 | 2.02 | 0.08 |
| 412 | Ethynylbenzene | 2.53 | 2.45 | 2.64 | 0.08 |
| 413 | Flunarizine | 5.78 | 5.70 | 5.70 | 0.08 |
| 414 | beta-Nitrostyrene | 2.24 | 2.16 | 2.15 | 0.08 |
| 415 | Cyclooctane | 4.45 | 4.37 | 4.41 | 0.08 |
| 416 | 1-Butyne | 1.44 | 1.36 | 1.38 | 0.08 |
| 417 | Trichloroacetic acid | 1.33 | 1.41 | 1.44 | -0.08 |
| 418 | 2,4-Dichloronitrobenzene | 3.09 | 3.17 | 3.15 | -0.08 |
| 419 | 3,5-Dichloronitrobenzene | 3.09 | 3.17 | 3.16 | -0.08 |
| 420 | Xylylcarb | 2.09 | 2.17 | 2.20 | -0.08 |
| 421 | Dazomet | 1.40 | 1.32 |  | 0.08 |
| 422 | 2,6-Xylenol | 2.36 | 2.44 | 2.45 | -0.08 |
| 423 | 4-Chlorophenol | 2.39 | 2.31 | 2.29 | 0.08 |
| 424 | 3-Methoxyphenoxyacetic acid | 1.38 | 1.30 | 1.25 | 0.08 |
| 425 | 2,3,4,6-Tetrachlorophenol | 3.88 | 3.96 | 3.96 | -0.08 |
| 426 | 2,3,5,6-Tetrachlorophenol | 3.88 | 3.96 | 3.96 | -0.08 |
| 427 | 3-Nitrophenoxyacetic acid | 1.37 | 1.29 | 1.32 | 0.08 |
| 428 | N-Nitrosopiperidine | 0.63 | 0.71 | 0.73 | -0.08 |
| 429 | 3-(2-Pyridyl)propylamine | 0.49 | 0.41 | 0.43 | 0.08 |
| 430 | 3-Nitrobenzenesulfonamide | 0.55 | 0.47 | 0.55 | 0.08 |
| 431 | N-Nitrosodipropylamine | 1.36 | 1.28 | 1.25 | 0.08 |
| 432 | 2-Pyridinemethanamine | -0.21 | -0.29 | -0.31 | 0.08 |
| 433 | 2-Bromophenoxyacetic acid | 2.10 | 2.02 | 2.02 | 0.08 |
| 434 | 4-Phenyl-1-butanol | 2.35 | 2.27 | 2.28 | 0.08 |
| 435 | N,N-Dimethylbenzenesulfonamide | 1.35 | 1.27 | 1.55 | 0.08 |
| 436 | 1,2-Dimethyl-3-nitrobenzene | 2.83 | 2.75 | 2.77 | 0.08 |
| 437 | 1-Naphthylmethylisothiocyanate | 4.42 | 4.50 | 4.51 | -0.08 |
| 438 | (L)-Alanine | -2.83 | -2.75 | -2.74 | -0.08 |
| 439 | Lomefloxacin | -0.80 | -0.88 | -0.94 | 0.08 |
| 440 | 3-Ureidophenoxyacetic acid | 0.26 | 0.18 | 0.16 | 0.08 |
| 441 | 3,5-Dinitro-4-methylbenzamide | 0.68 | 0.76 | 0.74 | -0.08 |
| 442 | 3-(3-Pyridyl)propylamine | 0.44 | 0.36 | 0.35 | 0.08 |
| 443 | 4-Amino-3-fluorobenzoic acid | 0.58 | 0.66 | 0.69 | -0.08 |
| 444 | 4-Bromopyridine | 1.54 | 1.46 | 1.49 | 0.08 |
| 445 | Bupropion | 3.21 | 3.29 | 3.23 | -0.08 |
| 446 | Methylpentachlorocyclohexane | 4.04 | 3.96 | 3.99 | 0.08 |
| 447 | Phenylthiocyanate | 2.54 | 2.46 |  | 0.08 |
| 448 | Ametryn | 2.98 | 3.06 |  | -0.08 |
| 449 | Cyclohexanol | 1.23 | 1.31 | 1.33 | -0.08 |
| 450 | 3-Propionylphenyl N-methylcarbamate | 1.49 | 1.57 | 1.51 | -0.08 |
| 451 | Triazolam | 5.50 | 5.42 | 5.32 | 0.08 |
| 452 | Isosorbide | -1.31 | -1.39 | -1.25 | 0.08 |
| 453 | 4-Chloroacetophenone | 2.35 | 2.43 | 2.41 | -0.08 |
| 454 | 3-Bromonitrobenzene | 2.64 | 2.72 | 2.74 | -0.08 |
| 455 | 4,4'-Dimethylbiphenyl | 5.09 | 5.01 | 4.94 | 0.08 |
| 456 | Methoxytetrachlorocyclohexane | 2.99 | 3.07 | 3.04 | -0.08 |
| 457 | 2,2',3,4,4'-Pentabromodiphenyl ether | 7.03 | 7.11 | 7.10 | -0.08 |
| 458 | p-Terphenyl | 6.03 | 6.11 | 6.20 | -0.08 |
| 459 | 2,3,4,4',6-Pentachlorobiphenyl | 6.44 | 6.52 | 6.51 | -0.08 |
| 460 | 2,2',3,4,4'-Pentachlorobiphenyl | 6.61 | 6.52 | 6.57 | 0.09 |
| 461 | Trihexyphenidyl | 4.49 | 4.40 | 4.38 | 0.09 |
| 462 | 3,4,5-Trimethylphenol | 2.87 | 2.78 | 2.78 | 0.09 |
| 463 | Tolmetin | 2.79 | 2.70 | 2.57 | 0.09 |
| 464 | Warfarin | 3.54 | 3.45 | 3.41 | 0.09 |
| 465 | Durene | 4.00 | 3.91 | 3.94 | 0.09 |
| 466 | Iodobenzene | 3.25 | 3.16 | 3.15 | 0.09 |
| 467 | Hexafluorobenzene | 2.54 | 2.63 | 2.90 | -0.09 |
| 468 | 4-Nitroquinoline | 2.06 | 2.15 | 2.14 | -0.09 |
| 469 | Cyclohexane | 3.44 | 3.35 | 3.39 | 0.09 |
| 470 | 2,6-Lutidine | 1.68 | 1.59 | 1.61 | 0.09 |
| 471 | 3-Fluorophenol | 1.93 | 1.84 | 1.86 | 0.09 |
| 472 | 2-Chloropropane | 1.90 | 1.81 | 1.84 | 0.09 |
| 473 | 2,4-Xylenol | 2.35 | 2.44 | 2.45 | -0.09 |
| 474 | 3,5-Xylenol | 2.35 | 2.44 | 2.46 | -0.09 |
| 475 | Triphenyl phosphate | 3.90 | 3.81 | 3.83 | 0.09 |
| 476 | 2,5-Xylidine | 1.86 | 1.95 | 1.90 | -0.09 |
| 477 | 2-Picoline | 1.11 | 1.20 | 1.22 | -0.09 |
| 478 | 3,4-Xylidine | 1.86 | 1.95 | 1.90 | -0.09 |
| 479 | 3-Bromophenylacetic acid | 2.37 | 2.46 | 2.47 | -0.09 |
| 480 | 4-Ethyltoluene | 3.63 | 3.54 | 3.56 | 0.09 |
| 481 | Metolachlor | 3.13 | 3.04 | 3.00 | 0.09 |
| 482 | cis-1,2-Dichloroethylene | 1.86 | 1.77 | 1.99 | 0.09 |
| 483 | Ornidazole | 0.60 | 0.51 | 0.49 | 0.09 |
| 484 | Mercaptoacetic acid | 0.09 | 0.18 | 0.17 | -0.09 |
| 485 | 2-Chloroethanol | 0.03 | -0.06 | -0.04 | 0.09 |
| 486 | Chlorotoluron | 2.41 | 2.50 | 2.56 | -0.09 |
| 487 | Benzylamine | 1.09 | 1.00 | 0.98 | 0.09 |
| 488 | Butyric acid | 0.79 | 0.88 | 0.90 | -0.09 |
| 489 | Fludrocortisone | 1.67 | 1.76 |  | -0.09 |
| 490 | Isobutyric acid | 0.94 | 0.85 | 0.85 | 0.09 |
| 491 | N-nitroso-morpholine | -0.44 | -0.53 | -0.58 | 0.09 |
| 492 | 4-Chloro-2-nitrotoluene | 3.05 | 2.96 | 2.95 | 0.09 |
| 493 | N-Methyl-2-quinolone | 1.45 | 1.54 | 1.62 | -0.09 |
| 494 | 4-Methylsulfonylbenzoic acid | 0.67 | 0.58 | 0.67 | 0.09 |
| 495 | Chlorsulfuron | 1.79 | 1.70 | 1.66 | 0.09 |
| 496 | 2-Cyano-6-methylpyridine | 0.84 | 0.93 | 0.97 | -0.09 |
| 497 | 3-Fluorophenyl N-methylcarbamate | 1.48 | 1.57 | 1.53 | -0.09 |
| 498 | Ethyl acetate | 0.73 | 0.82 | 0.79 | -0.09 |
| 499 | 2-Fluoroaniline | 1.26 | 1.35 | 1.32 | -0.09 |
| 500 | Chlorobutanol | 2.03 | 2.12 | 2.28 | -0.09 |
| 501 | 1,1-Dichloroethane | 1.79 | 1.88 | 1.88 | -0.09 |
| 502 | Carbaryl | 2.36 | 2.45 | 2.48 | -0.09 |
| 503 | 4-Phenylbutyric acid | 2.42 | 2.51 | 2.53 | -0.09 |
| 504 | 4-(Methylthio)phenyl N-methylcarbamate | 1.92 | 2.01 | 1.93 | -0.09 |
| 505 | 1,4-Dimethylnaphthalene | 4.37 | 4.27 | 4.20 | 0.10 |
| 506 | 2,3',4,4',5',6-Hexachlorobiphenyl | 7.17 | 7.07 | 7.03 | 0.10 |
| 507 | Bunolol | 2.40 | 2.30 | 2.24 | 0.10 |
| 508 | Hemimellitene | 3.63 | 3.53 | 3.57 | 0.10 |
| 509 | 2-Ethylnaphthalene | 4.38 | 4.28 | 4.28 | 0.10 |
| 510 | Haloperidol | 4.30 | 4.20 | 4.19 | 0.10 |
| 511 | 7-Methylquinoline | 2.47 | 2.57 | 2.55 | -0.10 |
| 512 | 3-Benzamidophenoxyacetic acid | 1.99 | 2.09 | 2.06 | -0.10 |
| 513 | Dosulepin | 4.49 | 4.59 | 4.55 | -0.10 |
| 514 | 2,2'-Dichlorobiphenyl | 4.97 | 4.87 | 4.89 | 0.10 |
| 515 | Oxyphenbutazone | 2.72 | 2.62 | 2.61 | 0.10 |
| 516 | Sulfaguanidine | -1.22 | -1.12 |  | -0.10 |
| 517 | 4-t-Butyl-N-nitrosopiperidine | 1.96 | 1.86 | 1.85 | 0.10 |
| 518 | m-Tolyl N,N-dimethylcarbamate | 2.05 | 2.15 | 2.18 | -0.10 |
| 519 | Triptanol | 4.93 | 4.83 | 4.82 | 0.10 |
| 520 | Allylpropylamine | 1.33 | 1.43 | 1.39 | -0.10 |
| 521 | 2,5-Xylenol | 2.34 | 2.44 | 2.48 | -0.10 |
| 522 | Clarithromycin | 3.16 | 3.06 | 3.11 | 0.10 |
| 523 | 4-Amino-6-isopentyl-3-methylthio-1,2,4-triazin-5-one | 1.85 | 1.95 | 1.86 | -0.10 |
| 524 | N-Nitrosodiethylamine | 0.48 | 0.58 | 0.58 | -0.10 |
| 525 | Vinylidenechloride | 2.13 | 2.23 | 2.22 | -0.10 |
| 526 | Sulfamethoxazole | 0.88 | 0.98 | 1.01 | -0.10 |
| 527 | Thioacetamide | -0.26 | -0.36 | -0.32 | 0.10 |
| 528 | 3-Hydroxyacetophenone | 1.39 | 1.49 | 1.50 | -0.10 |
| 529 | 4-(2-Pyridyl)butylamine | 0.86 | 0.76 | 0.69 | 0.10 |
| 530 | N-Ethyl-N'-phenylurea | 1.64 | 1.74 | 1.78 | -0.10 |
| 531 | 3-Pyridinemethanol | -0.02 | -0.12 | -0.11 | 0.10 |
| 532 | 2,6-Dichloropyridine | 2.15 | 2.25 | 2.33 | -0.10 |
| 533 | 4-Hydroxybenzenesulfonamide | 0.06 | 0.16 | 0.17 | -0.10 |
| 534 | 3-Hydroxy-N-Nitrosopiperidine | -0.47 | -0.37 | -0.36 | -0.10 |
| 535 | 2-Chloro-4-nitrophenol | 2.33 | 2.23 | 2.23 | 0.10 |
| 536 | 3-Mercaptopropionic acid | 0.43 | 0.53 | 0.58 | -0.10 |
| 537 | Dinoseb | 3.56 | 3.46 | 3.47 | 0.10 |
| 538 | 3-Cyanophenoxyacetic acid | 0.95 | 1.05 | 1.07 | -0.10 |
| 539 | 1-Methyl-1-hydroxyurea | -0.46 | -0.56 | -0.67 | 0.10 |
| 540 | Cyclopropane | 1.72 | 1.82 | 1.83 | -0.10 |
| 541 | 4-Hydroxy-3-methoxybenzaldehyde | 0.97 | 1.07 | 1.05 | -0.10 |
| 542 | 2-Chlorophenoxyacetic acid | 2.02 | 1.92 | 1.94 | 0.10 |
| 543 | Propylene | 1.77 | 1.67 | 1.69 | 0.10 |
| 544 | 2-Chloronitrobenzene | 2.52 | 2.62 | 2.65 | -0.10 |
| 545 | Phenylacetaldehyde | 1.78 | 1.88 | 1.86 | -0.10 |
| 546 | 3-Ethyl-4-hydroxyacetanilide | 1.79 | 1.89 | 1.88 | -0.10 |
| 547 | 1-Hydroxyadamantane | 2.14 | 2.24 | 2.37 | -0.10 |
| 548 | 4-Bromoacetophenone | 2.43 | 2.53 | 2.55 | -0.10 |
| 549 | 2,2',3,4,4',6'-Hexachlorobiphenyl | 6.97 | 7.07 | 7.01 | -0.10 |
| 550 | 2,2',3,4,5,6-Hexachlorobiphenyl | 6.97 | 7.07 | 7.03 | -0.10 |
| 551 | 2,3,3',4,4'-Pentachlorodiphenyl ether | 6.51 | 6.61 | 6.62 | -0.10 |
| 552 | 2,3',4,5',6-Pentachlorobiphenyl | 6.42 | 6.52 | 6.51 | -0.10 |
| 553 | 1,5-Dimethylnaphthalene | 4.38 | 4.27 | 4.38 | 0.11 |
| 554 | Dibenzo[b,d]thiophene | 4.38 | 4.27 | 4.30 | 0.11 |
| 555 | 2,3,5,6-Tetrachloronitrobenzene | 4.38 | 4.27 | 4.26 | 0.11 |
| 556 | Tolan | 4.78 | 4.89 | 4.98 | -0.11 |
| 557 | 1-Ethylnaphthalene | 4.39 | 4.28 | 4.36 | 0.11 |
| 558 | 4-Acetyl-7-chloroquinoline | 2.62 | 2.51 | 2.52 | 0.11 |
| 559 | 2,4-Dichlorophenoxyacetic acid | 2.58 | 2.47 | 2.48 | 0.11 |
| 560 | 2-(Methylthio)quinoxaline | 2.79 | 2.68 | 2.61 | 0.11 |
| 561 | Benzylphenylether | 3.79 | 3.68 | 3.63 | 0.11 |
| 562 | 2,3-Dichlorophenyl N-methylcarbamate | 2.48 | 2.59 | 2.59 | -0.11 |
| 563 | Mepyramine | 3.27 | 3.16 | 3.12 | 0.11 |
| 564 | 2,4-Dimethoxyamphetamine | 1.75 | 1.64 | 1.60 | 0.11 |
| 565 | 2,6-Dichlorophenol | 2.75 | 2.86 | 2.90 | -0.11 |
| 566 | 3-Bromophenyl N-methylcarbamate | 2.25 | 2.14 | 2.17 | 0.11 |
| 567 | 2,6-Dichlorobiphenyl | 4.98 | 4.87 | 4.89 | 0.11 |
| 568 | Phenanthrene | 4.52 | 4.63 | 4.78 | -0.11 |
| 569 | 2,2',3-Trichlorobiphenyl | 5.31 | 5.42 | 5.36 | -0.11 |
| 570 | Tolclofos-methyl | 4.56 | 4.67 | 4.66 | -0.11 |
| 571 | 3-Methoxyphenol | 1.58 | 1.69 | 1.71 | -0.11 |
| 572 | Thioridazine | 5.90 | 5.79 | 5.78 | 0.11 |
| 573 | 5-(4-Pyridyl)pentanol | 1.39 | 1.28 | 1.22 | 0.11 |
| 574 | Guanidinoacetic acid | -1.11 | -1.22 |  | 0.11 |
| 575 | Cefacetrile | -0.45 | -0.34 | -0.18 | -0.11 |
| 576 | Mandelic acid | 0.62 | 0.73 | 0.77 | -0.11 |
| 577 | 2,4,5-T | 3.13 | 3.02 | 3.02 | 0.11 |
| 578 | 2-Isopropylphenol | 2.88 | 2.77 | 2.77 | 0.11 |
| 579 | Diflubenzuron | 3.88 | 3.77 | 3.81 | 0.11 |
| 580 | 4-Ethoxyphenyl N-methylcarbamate | 1.63 | 1.74 | 1.75 | -0.11 |
| 581 | 5-Bromouracil | -0.21 | -0.10 | -0.04 | -0.11 |
| 582 | Isopropylamine | 0.26 | 0.15 | 0.15 | 0.11 |
| 583 | 2-(3-Pyridyl)ethanol | 0.12 | 0.23 | 0.27 | -0.11 |
| 584 | Dalapon | 0.78 | 0.89 | 0.90 | -0.11 |
| 585 | N-Phenylhydroxylamine | 0.79 | 0.90 | 0.90 | -0.11 |
| 586 | 2-Bromoethanol | 0.23 | 0.34 | 0.35 | -0.11 |
| 587 | Allyl alcohol | 0.17 | 0.28 | 0.29 | -0.11 |
| 588 | 2,6-Diisopropylaniline | 3.18 | 3.29 | 3.29 | -0.11 |
| 589 | 3-Acetylpyridine | 0.43 | 0.54 | 0.53 | -0.11 |
| 590 | 4-Nitrobenzaldehyde | 1.56 | 1.45 | 1.44 | 0.11 |
| 591 | 1,4-Cyclohexadiene | 2.30 | 2.19 | 2.26 | 0.11 |
| 592 | 2-Trifluoromethylphenol | 2.80 | 2.69 | 2.68 | 0.11 |
| 593 | Tilmicosin | 3.80 | 3.69 | 3.57 | 0.11 |
| 594 | 3-Chlorophenoxyacetic acid | 2.03 | 1.92 | 1.94 | 0.11 |
| 595 | Ethoxybenzene | 2.51 | 2.40 | 2.36 | 0.11 |
| 596 | 1,2,4,7,8-Pentachlorodibenzofuran | 6.26 | 6.37 | 6.37 | -0.11 |
| 597 | beta-HCH | 3.78 | 3.89 | 3.94 | -0.11 |
| 598 | Pentachloroethane | 3.22 | 3.11 | 3.12 | 0.11 |
| 599 | Progabide | 2.97 | 2.86 | 2.74 | 0.11 |
| 600 | DIDP | 7.70 | 7.59 | 7.47 | 0.11 |
| 601 | 2-Nitrotoluene | 2.30 | 2.41 | 2.44 | -0.11 |
| 602 | 4-Methoxy-beta-nitrostyrene | 2.20 | 2.09 | 2.09 | 0.11 |
| 603 | N-Ethylaniline | 2.16 | 2.05 | 2.04 | 0.11 |
| 604 | 1,2,3-Trichloro-4-nitrobenzene | 3.61 | 3.72 | 3.69 | -0.11 |
| 605 | Bisoprolol | 2.15 | 2.26 | 2.40 | -0.11 |
| 606 | Cinnamaldehyde | 1.90 | 2.01 | 2.04 | -0.11 |
| 607 | 2,3,6-Trimethylphenol | 2.67 | 2.78 | 2.82 | -0.11 |
| 608 | 1,3,5-Trimethylbenzene | 3.42 | 3.53 | 3.55 | -0.11 |
| 609 | 2,3,3',5,6-Pentachlorobiphenyl | 6.41 | 6.52 | 6.57 | -0.11 |
| 610 | 2,2',4,4'-Tetrachlorodiphenyl ether | 5.95 | 6.06 | 6.09 | -0.11 |
| 611 | 2,3',4,4',5'-Pentachlorobiphenyl | 6.64 | 6.52 | 6.57 | 0.12 |
| 612 | 2,2',3,4,5,5'-Hexachlorobiphenyl | 7.19 | 7.07 | 7.01 | 0.12 |
| 613 | 2,3,3',4'-Tetrachlorobiphenyl | 5.85 | 5.97 | 6.02 | -0.12 |
| 614 | 2,3,5,6-Tetrachloroanisole | 4.40 | 4.28 | 4.29 | 0.12 |
| 615 | 1-Naphthol | 2.84 | 2.72 | 2.75 | 0.12 |
| 616 | 1-(3-CF3-phenylhydrazono)-1-cyanoacetic acid methylester | 3.78 | 3.66 | 3.58 | 0.12 |
| 617 | 4-Bromophenylisothiocyanate | 4.03 | 3.91 | 3.91 | 0.12 |
| 618 | 4-Nitrobenzylalcohol | 1.26 | 1.14 | 1.13 | 0.12 |
| 619 | alpha-Estradiol | 4.01 | 3.89 | 3.85 | 0.12 |
| 620 | 2,3-Dichlorobiphenyl | 4.99 | 4.87 | 4.82 | 0.12 |
| 621 | Meloxicam | 3.01 | 3.13 | 2.89 | -0.12 |
| 622 | 3-Methyl-4-methylthiophenyl N-methylcarbamate | 2.47 | 2.35 | 2.29 | 0.12 |
| 623 | Etoposide | 1.97 | 1.85 | 1.86 | 0.12 |
| 624 | p-Tolyl N,N-dimethylcarbamate | 2.03 | 2.15 | 2.15 | -0.12 |
| 625 | 2,3-Dichloronitrobenzene | 3.05 | 3.17 | 3.19 | -0.12 |
| 626 | 3-Methoxyphenyl N-methylcarbamate | 1.30 | 1.42 | 1.43 | -0.12 |
| 627 | 2,5-Dihydrofuran | 0.46 | 0.58 | 0.67 | -0.12 |
| 628 | 3-Amino-4-chlorophenoxyacetic acid | 1.16 | 1.04 | 1.01 | 0.12 |
| 629 | N,N'-Dinitrosopiperazine | -0.85 | -0.97 | -0.97 | 0.12 |
| 630 | Sulfometuron-methyl | 1.40 | 1.28 | 1.16 | 0.12 |
| 631 | 4-(2-Pyridyl)butanol | 0.86 | 0.98 | 0.95 | -0.12 |
| 632 | Ethyl formate | 0.26 | 0.14 | 0.23 | 0.12 |
| 633 | Hydracrylic acid | -0.62 | -0.74 | -0.74 | 0.12 |
| 634 | Methyl methacrylate | 1.38 | 1.26 | 1.24 | 0.12 |
| 635 | Procarbazine | 0.06 | 0.18 |  | -0.12 |
| 636 | Nicosulfuron | 0.50 | 0.62 | 0.65 | -0.12 |
| 637 | 4-Amino-2-nitrobenzoic acid | 0.38 | 0.50 | 0.49 | -0.12 |
| 638 | 4-Propoylpyridine | 0.77 | 0.89 | 0.86 | -0.12 |
| 639 | 2-Iodophenol | 2.65 | 2.77 | 2.78 | -0.12 |
| 640 | 2-Methylfuran | 1.85 | 1.73 | 1.84 | 0.12 |
| 641 | Fluometuron | 2.42 | 2.54 | 2.51 | -0.12 |
| 642 | 4-Cyanophenoxyacetic acid | 0.93 | 1.05 | 1.08 | -0.12 |
| 643 | 3-Amino-4-phenyl-1,2,5-oxadiazole | 1.81 | 1.69 | 1.55 | 0.12 |
| 644 | Phenyl N,N-dimethylcarbamate | 1.69 | 1.81 | 1.87 | -0.12 |
| 645 | 1,2,3,4-Tetrahydroquinoline | 2.29 | 2.17 | 2.16 | 0.12 |
| 646 | 2,3',4-Trichlorobiphenyl | 5.54 | 5.42 | 5.46 | 0.12 |
| 647 | Tralkoxydim | 4.46 | 4.58 | 4.67 | -0.12 |
| 648 | o-Cresol | 1.98 | 2.10 | 2.07 | -0.12 |
| 649 | 3-Acetylphenoxyacetic acid | 0.98 | 1.10 | 1.07 | -0.12 |
| 650 | m-Cresol | 1.98 | 2.10 | 2.11 | -0.12 |
| 651 | Oxprenolol | 2.10 | 2.22 | 2.18 | -0.12 |
| 652 | 3-Trifluoromethylphenylacetic acid | 2.62 | 2.74 | 2.73 | -0.12 |
| 653 | 3,5-Dipropyl-4-hydroxyacetanilide | 3.16 | 3.28 | 3.23 | -0.12 |
| 654 | 1-Naphthylisothiocyanate | 4.34 | 4.22 | 4.24 | 0.12 |
| 655 | 2,2',5,5'-Tetrachlorobiphenyl | 6.09 | 5.97 | 5.95 | 0.12 |
| 656 | 2-Naphthylisothiocyanate | 4.34 | 4.22 | 4.26 | 0.12 |
| 657 | 2,2',3,4,4',6-Hexachlorobiphenyl | 6.95 | 7.07 | 7.05 | -0.12 |
| 658 | 2,6-Di-s-butylphenol | 4.36 | 4.48 | 4.42 | -0.12 |
| 659 | 2,3',4,4',6-Pentachlorobiphenyl | 6.40 | 6.52 | 6.51 | -0.12 |
| 660 | 2,3,3',4,4'-Pentachlorobiphenyl | 6.65 | 6.52 | 6.48 | 0.13 |
| 661 | Guajen | 4.40 | 4.27 | 4.33 | 0.13 |
| 662 | 2,3,3',4',5',6-Hexachlorobiphenyl | 7.20 | 7.07 | 7.01 | 0.13 |
| 663 | 2-n-Propylphenol | 2.93 | 2.80 | 2.78 | 0.13 |
| 664 | 2,3,4,4'-Tetrachlorobiphenyl | 5.84 | 5.97 | 5.93 | -0.13 |
| 665 | Acenaphthene | 3.92 | 4.05 | 4.09 | -0.13 |
| 666 | Isodurene | 4.04 | 3.91 | 3.98 | 0.13 |
| 667 | 3-Methyl-2-butanol | 1.28 | 1.15 | 1.16 | 0.13 |
| 668 | Caproic aldehyde | 1.78 | 1.65 | 1.68 | 0.13 |
| 669 | alpha-Methylstyrene | 3.48 | 3.61 | 3.66 | -0.13 |
| 670 | Arabinose | -3.02 | -2.89 | -2.82 | -0.13 |
| 671 | d-Lyxose | -3.02 | -2.89 | -2.82 | -0.13 |
| 672 | Xylose | -3.02 | -2.89 | -2.87 | -0.13 |
| 673 | 2,2',5,5'-Tetrabromobiphenyl | 6.50 | 6.37 | 6.38 | 0.13 |
| 674 | 2-Chloroanisole | 2.50 | 2.63 | 2.67 | -0.13 |
| 675 | Anthracene | 4.50 | 4.63 | 4.65 | -0.13 |
| 676 | 1-Isopropylbenzotriazole | 1.98 | 1.85 | 1.89 | 0.13 |
| 677 | Trimeprazine | 4.71 | 4.58 | 4.58 | 0.13 |
| 678 | epsilon-Aminocaproic acid | -1.95 | -1.82 | -1.77 | -0.13 |
| 679 | Levomoprolol | 1.69 | 1.56 | 1.55 | 0.13 |
| 680 | 5-(3-Pyridyl)pentanol | 1.41 | 1.28 | 1.25 | 0.13 |
| 681 | Bunitrolol | 1.91 | 1.78 | 1.75 | 0.13 |
| 682 | 2-Hydroxyphenoxyacetic acid | 0.85 | 0.98 | 0.98 | -0.13 |
| 683 | 3-Nitropyridine | 0.60 | 0.73 | 0.74 | -0.13 |
| 684 | 4-Iodophenol | 2.90 | 2.77 | 2.78 | 0.13 |
| 685 | Mexiletine | 2.15 | 2.02 | 2.02 | 0.13 |
| 686 | N-Methylindol-2,3-dione | 0.58 | 0.45 | 0.53 | 0.13 |
| 687 | 2-Methylquinoxaline | 1.61 | 1.74 | 1.72 | -0.13 |
| 688 | Pyridine-3-nitrile | 0.36 | 0.49 | 0.58 | -0.13 |
| 689 | 1,4-Dimethylquinoxalin-2,3-dione | 0.08 | 0.21 | 0.31 | -0.13 |
| 690 | 1,2-Dichlorobenzene | 3.38 | 3.25 | 3.29 | 0.13 |
| 691 | 2-Methyl-beta-nitrostyrene | 2.63 | 2.50 | 2.52 | 0.13 |
| 692 | Hydrochlorothiazide | -0.03 | -0.16 | -0.17 | 0.13 |
| 693 | 2-Pyridinemethanol | 0.06 | -0.07 | -0.04 | 0.13 |
| 694 | 2-(4-Pyridyl)ethanol | 0.10 | 0.23 | 0.24 | -0.13 |
| 695 | Ethyl ether | 0.89 | 1.02 | 1.02 | -0.13 |
| 696 | 2-Chloro-6-nitrotoluene | 3.09 | 2.96 | 2.96 | 0.13 |
| 697 | 4-Anisaldehyde | 1.59 | 1.46 | 1.50 | 0.13 |
| 698 | 3-Phenylpropylamine | 1.83 | 1.70 | 1.70 | 0.13 |
| 699 | 3-Nitroquinoline-1-oxide | 0.56 | 0.69 | 0.67 | -0.13 |
| 700 | 4-Acetylformanilide | 0.94 | 1.07 | 1.07 | -0.13 |
| 701 | Metolcarb | 1.70 | 1.83 | 1.83 | -0.13 |
| 702 | 2-Fluorophenol | 1.71 | 1.84 | 1.87 | -0.13 |
| 703 | 4-Chloro-2-methylphenol | 2.78 | 2.65 | 2.63 | 0.13 |
| 704 | p-Cresol | 1.97 | 2.10 | 2.14 | -0.13 |
| 705 | Dapsone | 0.97 | 1.10 | 1.11 | -0.13 |
| 706 | Prazepam | 3.72 | 3.85 | 3.79 | -0.13 |
| 707 | 2,4'-Dibromodiphenyl ether | 5.03 | 5.16 | 5.15 | -0.13 |
| 708 | 2,4-Dibromodiphenyl ether | 5.03 | 5.16 | 5.19 | -0.13 |
| 709 | 2,6-Dibromodiphenyl ether | 5.03 | 5.16 | 5.15 | -0.13 |
| 710 | 3,4'-Dibromodiphenyl ether | 5.03 | 5.16 | 5.19 | -0.13 |
| 711 | 3,4-Dibromodiphenyl ether | 5.03 | 5.16 | 5.19 | -0.13 |
| 712 | Bis(4-bromophenyl)ether | 5.03 | 5.16 | 5.19 | -0.13 |
| 713 | (3-Bromopropyl)benzene | 3.72 | 3.59 | 3.63 | 0.13 |
| 714 | 2,3,4',5-Tetrachlorobiphenyl | 6.10 | 5.97 | 5.95 | 0.13 |
| 715 | 2,3,4',5,6-Pentachlorobiphenyl | 6.39 | 6.52 | 6.52 | -0.13 |
| 716 | DDT | 6.91 | 6.77 | 6.89 | 0.14 |
| 717 | Butachlor | 4.50 | 4.36 | 4.47 | 0.14 |
| 718 | Clofibrate | 3.65 | 3.51 | 3.53 | 0.14 |
| 719 | 2,2',4,4',5,5'-Hexabromodiphenyl ether | 7.62 | 7.76 | 7.78 | -0.14 |
| 720 | Chlorobenzene | 2.84 | 2.70 | 2.74 | 0.14 |
| 721 | Sulfinpyrazone | 2.30 | 2.16 | 1.89 | 0.14 |
| 722 | 5-Chlorovanillin | 1.76 | 1.62 | 1.61 | 0.14 |
| 723 | 6-Chlorovanillin | 1.76 | 1.62 | 1.61 | 0.14 |
| 724 | N-(4-Methoxyphenyl)sulfanilamide | 1.51 | 1.37 | 1.36 | 0.14 |
| 725 | 1,4-Dichloro-2-nitrobenzene | 3.03 | 3.17 | 3.20 | -0.14 |
| 726 | 2,5-Dimethylphenyl N-methylcarbamate | 2.03 | 2.17 | 2.18 | -0.14 |
| 727 | 3-Methyl-4-hydroxybenzaldehyde | 1.21 | 1.07 | 1.06 | 0.14 |
| 728 | Ethyldimethylamine | 0.70 | 0.56 | 0.58 | 0.14 |
| 729 | 2,3-Xylidine | 1.81 | 1.95 | 1.96 | -0.14 |
| 730 | 2-Ethylpyridine | 1.69 | 1.55 | 1.59 | 0.14 |
| 731 | Phenylurea | 0.82 | 0.96 | 0.95 | -0.14 |
| 732 | 3-Chloro-beta-nitrostyrene | 2.57 | 2.71 | 2.69 | -0.14 |
| 733 | Carbofuran | 2.32 | 2.46 | 2.45 | -0.14 |
| 734 | Ciprofloxacin | -1.08 | -1.22 | -1.17 | 0.14 |
| 735 | Ethylmethylamine | 0.15 | 0.29 | 0.27 | -0.14 |
| 736 | N-Ethylbromoacetamide | 0.34 | 0.48 | 0.43 | -0.14 |
| 737 | 1-Phenylhydrazono-1-cyanoacetic acid methylester | 2.59 | 2.73 | 2.74 | -0.14 |
| 738 | Cumene | 3.66 | 3.52 | 3.56 | 0.14 |
| 739 | 3-Methoxyphenyl N,N-dimethylcarbamate | 1.60 | 1.74 | 1.75 | -0.14 |
| 740 | 4-Acetylphenol | 1.35 | 1.49 | 1.48 | -0.14 |
| 741 | 6-t-Butyl-3-methylphenyl N-methylcarbamate | 3.14 | 3.00 | 2.96 | 0.14 |
| 742 | Desmycosin | 1.63 | 1.49 | 1.51 | 0.14 |
| 743 | Trichlormethiazide | 0.56 | 0.42 | 0.35 | 0.14 |
| 744 | 1,2,3,4,7-Pentachlorodibenzo-p-dioxin | 6.60 | 6.46 | 6.44 | 0.14 |
| 745 | 1-Bromopropane | 2.10 | 1.96 | 1.98 | 0.14 |
| 746 | 4-Chloro-beta-nitrostyrene | 2.85 | 2.71 | 2.69 | 0.14 |
| 747 | Perazine | 3.90 | 4.04 | 3.90 | -0.14 |
| 748 | Triflusulfuron-methyl | 3.40 | 3.54 | 3.53 | -0.14 |
| 749 | 3-Bromopyridine | 1.60 | 1.46 | 1.49 | 0.14 |
| 750 | Pentoxyfylline | 0.72 | 0.58 | 0.54 | 0.14 |
| 751 | Cinnamic acid | 2.08 | 1.94 | 1.95 | 0.14 |
| 752 | 2-s-Butylphenyl N,N-dimethylcarbamate | 3.31 | 3.17 | 3.17 | 0.14 |
| 753 | 3-Iodonitrobenzene | 2.94 | 3.08 | 3.08 | -0.14 |
| 754 | Pirimicarb | 1.70 | 1.84 | 1.97 | -0.14 |
| 755 | Quinuclidine | 1.20 | 1.34 | 1.34 | -0.14 |
| 756 | 4-Iodoacetanilide | 2.46 | 2.60 | 2.60 | -0.14 |
| 757 | Benzyl acetate | 1.96 | 2.10 | 2.12 | -0.14 |
| 758 | Cycloheptane | 4.00 | 3.86 | 3.90 | 0.14 |
| 759 | 7-Trifluoromethylquinoline | 3.02 | 3.16 | 3.17 | -0.14 |
| 760 | 4-Pentylpyrazole | 2.96 | 2.82 | 2.79 | 0.14 |
| 761 | 3-Methylphenanthrene | 5.15 | 5.01 | 4.93 | 0.14 |
| 762 | 3,5-Dibromo-4-hydroxybenzonitrile | 2.60 | 2.74 | 2.72 | -0.14 |
| 763 | 2,2',3,4-Tetrachlorobiphenyl | 6.11 | 5.97 | 5.97 | 0.14 |
| 764 | Benzyl ethyl ether | 2.16 | 2.30 | 2.34 | -0.14 |
| 765 | 4-Chloro-6-nitro-m-cresol | 2.93 | 3.07 | 3.08 | -0.14 |
| 766 | Indane | 3.18 | 3.32 | 3.41 | -0.14 |
| 767 | 2,2',3,4',5,6-Hexachlorobiphenyl | 6.93 | 7.07 | 7.05 | -0.14 |
| 768 | 2,2',3,3',4-Pentabromodiphenyl ether | 6.97 | 7.11 | 7.13 | -0.14 |
| 769 | 2,2',4,4',5-Pentabromodiphenyl ether | 6.97 | 7.11 | 7.13 | -0.14 |
| 770 | 2,3,4,4',6-Pentabromodiphenyl ether | 6.97 | 7.11 | 7.10 | -0.14 |
| 771 | 3,3',4,4',5-Pentabromodiphenyl ether | 6.97 | 7.11 | 7.10 | -0.14 |
| 772 | Pimozide | 6.30 | 6.44 | 6.38 | -0.14 |
| 773 | Benzo[k]fluoranthene | 6.11 | 6.25 | 6.19 | -0.14 |
| 774 | 1,3-Dimethylnaphthalene | 4.42 | 4.27 | 4.33 | 0.15 |
| 775 | 2,2',3,4',5'-Pentachlorobiphenyl | 6.67 | 6.52 | 6.49 | 0.15 |
| 776 | Propazine | 2.93 | 2.78 | 2.71 | 0.15 |
| 777 | Phenylbutazone | 3.16 | 3.01 | 3.18 | 0.15 |
| 778 | 2-Methyl-8-quinolinol | 2.33 | 2.18 | 2.18 | 0.15 |
| 779 | 2,5-Dichlorophenyl N-methylcarbamate | 2.44 | 2.59 | 2.62 | -0.15 |
| 780 | Harmalol | 2.19 | 2.34 | 2.26 | -0.15 |
| 781 | (S)-(+)-Nirvanol | 1.53 | 1.38 | 1.44 | 0.15 |
| 782 | 2,3'-Dichlorobiphenyl | 5.02 | 4.87 | 4.89 | 0.15 |
| 783 | 2-s-Butylphenol | 3.27 | 3.12 | 3.10 | 0.15 |
| 784 | 3-Phenylphenol | 3.23 | 3.38 | 3.32 | -0.15 |
| 785 | Dexamethasone | 2.01 | 1.86 |  | 0.15 |
| 786 | 2-Acetylphenoxyacetic acid | 1.25 | 1.10 | 1.07 | 0.15 |
| 787 | Fomesafen | 3.00 | 3.15 | 3.17 | -0.15 |
| 788 | 4-Bromophenylacetic acid | 2.31 | 2.46 | 2.47 | -0.15 |
| 789 | Lacidipine | 5.56 | 5.71 | 5.77 | -0.15 |
| 790 | Tetrabromo-2-trifluoromethylbenzimidazole | 4.81 | 4.96 | 4.98 | -0.15 |
| 791 | Thiabendazole | 1.94 | 1.79 | 1.61 | 0.15 |
| 792 | Thiamphenicol | -0.27 | -0.42 | -0.43 | 0.15 |
| 793 | Ethylcarbamate | -0.15 | -0.30 | -0.29 | 0.15 |
| 794 | 4-Nitroacetanilide | 1.66 | 1.51 | 1.48 | 0.15 |
| 795 | Benzo[j]fluoranthene | 6.40 | 6.25 | 6.33 | 0.15 |
| 796 | Tetracene | 5.90 | 5.75 | 5.85 | 0.15 |
| 797 | Procainamide | 0.88 | 0.73 | 0.76 | 0.15 |
| 798 | 2-Aminoacridine | 2.62 | 2.77 | 2.73 | -0.15 |
| 799 | 2-Phenylpyridine | 2.63 | 2.48 | 2.44 | 0.15 |
| 800 | Nitrazepam | 2.12 | 2.27 | 2.24 | -0.15 |
| 801 | 2-Azacycloheptanethione | 0.75 | 0.90 | 1.00 | -0.15 |
| 802 | Ethylamine | -0.13 | -0.28 | -0.26 | 0.15 |
| 803 | 4-(4-Pyridyl)butylamine | 0.86 | 0.71 | 0.71 | 0.15 |
| 804 | 4-Methylphenoxyacetic acid | 1.86 | 1.71 | 1.73 | 0.15 |
| 805 | Benzimidazole | 1.32 | 1.17 | 1.14 | 0.15 |
| 806 | 2,3,3'-Trichlorobiphenyl | 5.57 | 5.42 | 5.46 | 0.15 |
| 807 | Dimethyl phthalate | 1.56 | 1.41 | 1.37 | 0.15 |
| 808 | Carbamazepine | 2.45 | 2.60 | 2.57 | -0.15 |
| 809 | 4-Chloroanisole | 2.78 | 2.63 | 2.60 | 0.15 |
| 810 | Propylisopropylacetamide | 1.48 | 1.63 | 1.68 | -0.15 |
| 811 | 2,3-Dimethoxyamphetamine | 1.49 | 1.64 | 1.54 | -0.15 |
| 812 | 2-Phenethylisothiocyanate | 3.74 | 3.89 | 3.82 | -0.15 |
| 813 | 5-Chloroquinoxaline | 1.75 | 1.90 | 1.89 | -0.15 |
| 814 | 2,2',3,4,4',5'-Hexachlorodiphenyl ether | 7.01 | 7.16 | 7.13 | -0.15 |
| 815 | Mephenamic acid | 4.20 | 4.05 | 4.08 | 0.15 |
| 816 | beta-Phenylethylacetate | 2.30 | 2.45 | 2.45 | -0.15 |
| 817 | 2,3,3',4,4',5'-Hexabromodiphenyl ether | 7.91 | 7.76 | 7.77 | 0.15 |
| 818 | (2-Bromoethyl)benzene | 3.09 | 3.24 | 3.28 | -0.15 |
| 819 | N-Nitrosodiphenylamine | 3.13 | 3.28 | 3.28 | -0.15 |
| 820 | 2,2',3,4,5'-Pentachlorobiphenyl | 6.37 | 6.52 | 6.49 | -0.15 |
| 821 | Benzo[a]fluorene | 5.68 | 5.52 | 5.47 | 0.16 |
| 822 | 1-Nitronaphthalene | 3.19 | 3.03 | 3.01 | 0.16 |
| 823 | 2,2',3,5'-Tetrachlorobiphenyl | 5.81 | 5.97 | 6.02 | -0.16 |
| 824 | 2,6-Dichloro-4-nitrophenol | 2.94 | 2.78 | 2.77 | 0.16 |
| 825 | 4-Cumylphenyl diphenyl phosphate | 6.10 | 6.26 | 6.21 | -0.16 |
| 826 | 4-Aminosalicylic acid 2-methoxyphenylester | 2.88 | 2.72 | 2.70 | 0.16 |
| 827 | N-Nitrosotriethylurea | 1.54 | 1.38 | 1.39 | 0.16 |
| 828 | 1,2,3,4,7-Pentachlorodibenzofuran | 6.53 | 6.37 | 6.29 | 0.16 |
| 829 | 8-Hydroxyquinoline | 2.00 | 1.84 | 1.85 | 0.16 |
| 830 | N,N-Dimethyl-3-methoxybenzamide | 1.00 | 1.16 | 1.14 | -0.16 |
| 831 | Flurbiprofen | 3.99 | 3.83 | 3.80 | 0.16 |
| 832 | 4-Benzoylpyridine | 1.98 | 1.82 | 1.84 | 0.16 |
| 833 | 2,4-Dibromophenol | 3.22 | 3.06 | 3.04 | 0.16 |
| 834 | Phenytoin | 2.47 | 2.31 | 2.29 | 0.16 |
| 835 | Allyl bromide | 1.79 | 1.95 | 1.96 | -0.16 |
| 836 | 3,4-Methylenedioxybenzylalcohol | 1.05 | 1.21 | 1.26 | -0.16 |
| 837 | Diethyl sulphide | 1.95 | 1.79 | 1.83 | 0.16 |
| 838 | Monolinuron | 2.30 | 2.46 |  | -0.16 |
| 839 | 3-Methylquinazolin-4-one | 0.69 | 0.53 | 0.61 | 0.16 |
| 840 | Leucine | -1.57 | -1.73 | -1.73 | 0.16 |
| 841 | Methylamine | -0.57 | -0.73 | -0.73 | 0.16 |
| 842 | 3-Iodophenol | 2.93 | 2.77 | 2.78 | 0.16 |
| 843 | 3-phenylpropionic acid methylester | 2.32 | 2.48 | 2.53 | -0.16 |
| 844 | Naproxen | 3.18 | 3.02 | 3.03 | 0.16 |
| 845 | alpha-Hydroxybutyric acid | -0.36 | -0.20 | -0.21 | -0.16 |
| 846 | Diethylamine | 0.58 | 0.74 | 0.73 | -0.16 |
| 847 | Thifensulfuron | 0.02 | 0.18 | 0.21 | -0.16 |
| 848 | 3-Acetaminpyridine | 0.41 | 0.25 | 0.23 | 0.16 |
| 849 | 1,2-Dimethyl-4-nitrobenzene | 2.91 | 2.75 | 2.76 | 0.16 |
| 850 | 3-Nitroanisol | 2.16 | 2.00 | 1.98 | 0.16 |
| 851 | 4-Methyl-beta-nitrostyrene | 2.66 | 2.50 | 2.59 | 0.16 |
| 852 | 4-Aminoquinoline-1-oxide | 0.05 | -0.11 | -0.04 | 0.16 |
| 853 | N-Nitrosothiomorpholine | 0.40 | 0.24 | 0.25 | 0.16 |
| 854 | Pentanoic acid | 1.39 | 1.23 | 1.22 | 0.16 |
| 855 | 1-Methylbenzotriazole | 1.13 | 0.97 | 0.82 | 0.16 |
| 856 | 2-(2-Pyridyl)ethanol | 0.12 | 0.28 | 0.30 | -0.16 |
| 857 | 2-Methylisoniazid | -0.37 | -0.53 | -0.50 | 0.16 |
| 858 | 4-Butoxyphenol | 2.87 | 2.71 | 2.70 | 0.16 |
| 859 | 3-Ethyl-1-hydroxyurea | -0.76 | -0.92 | -0.94 | 0.16 |
| 860 | 4-Bromobenzenesulfonamide | 1.36 | 1.20 | 1.19 | 0.16 |
| 861 | 2-Chlorophenol | 2.15 | 2.31 | 2.34 | -0.16 |
| 862 | Sulindac | 3.60 | 3.44 | 3.35 | 0.16 |
| 863 | 2-Nitroaniline | 1.85 | 1.69 | 1.70 | 0.16 |
| 864 | 4-Cyanophenol | 1.60 | 1.44 | 1.45 | 0.16 |
| 865 | Ethyl acrylate | 1.32 | 1.16 | 1.14 | 0.16 |
| 866 | 3-Chloronitrobenzene | 2.46 | 2.62 | 2.65 | -0.16 |
| 867 | Cyclopentane | 3.00 | 2.84 | 2.86 | 0.16 |
| 868 | N,N-Dimethyl-N'-(3-chlorophenyl)urea | 2.00 | 2.16 | 2.16 | -0.16 |
| 869 | 2-Chloroquinoxaline | 2.23 | 2.07 | 1.95 | 0.16 |
| 870 | Moracizine | 2.98 | 2.82 | 2.84 | 0.16 |
| 871 | 2-Phenylphenoxyacetic acid | 2.83 | 2.99 | 2.95 | -0.16 |
| 872 | Naphthalene | 3.35 | 3.51 | 3.49 | -0.16 |
| 873 | Clomethiazole | 2.12 | 2.28 | 2.47 | -0.16 |
| 874 | 1,1,2,2-Tetrachloroethane | 2.39 | 2.55 | 2.60 | -0.16 |
| 875 | 2,2',3,3',5,6-Hexachlorobiphenyl | 6.91 | 7.07 | 7.05 | -0.16 |
| 876 | Pyrazinamide | -1.88 | -2.05 | -2.02 | 0.17 |
| 877 | 1,7-Dimethylnaphthalene | 4.44 | 4.27 | 4.29 | 0.17 |
| 878 | Isocumene | 3.72 | 3.55 | 3.58 | 0.17 |
| 879 | 1,2,4-Trimethylbenzene | 3.70 | 3.53 | 3.56 | 0.17 |
| 880 | Pyrene | 4.88 | 5.05 | 5.15 | -0.17 |
| 881 | 4-Chloro-3-iodophenoxyacetic acid | 3.10 | 2.93 | 2.95 | 0.17 |
| 882 | 4-Bromo-3,5-dimethoxyphenethylamine | 2.03 | 1.86 | 1.83 | 0.17 |
| 883 | Tribenuron-methyl | 1.50 | 1.33 | 1.15 | 0.17 |
| 884 | 1,2,3-Trichloropropane | 1.98 | 1.81 | 1.83 | 0.17 |
| 885 | 3,3-Dimethyl-2-butanol | 1.48 | 1.31 | 1.28 | 0.17 |
| 886 | Bromoacetic acid | 0.41 | 0.58 | 0.57 | -0.17 |
| 887 | 4-Nitrobenzenesulfonamide | 0.64 | 0.47 | 0.40 | 0.17 |
| 888 | Benzyl alcohol | 1.05 | 1.22 | 1.20 | -0.17 |
| 889 | Methylhippurate | 0.82 | 0.65 | 0.63 | 0.17 |
| 890 | 3-Chlorobenzylalcohol | 1.94 | 1.77 | 1.80 | 0.17 |
| 891 | 3-(Dimethylamino)phenyl N-methylcarbamate | 1.43 | 1.26 | 1.31 | 0.17 |
| 892 | 4-Nitroformanilide | 1.43 | 1.26 | 1.12 | 0.17 |
| 893 | Triprolidine | 3.92 | 3.75 | 3.77 | 0.17 |
| 894 | 1,4-Benzoquinone | 0.20 | 0.03 | 0.07 | 0.17 |
| 895 | Dihydro-2(3H)-thiophenone | 0.60 | 0.43 |  | 0.17 |
| 896 | 6-Chloro-4-nitroquinoline-1-oxide | 1.41 | 1.24 | 1.25 | 0.17 |
| 897 | 4-Amino-3-bromobenzoic acid | 1.40 | 1.23 | 1.24 | 0.17 |
| 898 | Acrylic acid | 0.35 | 0.52 | 0.53 | -0.17 |
| 899 | 4-(3-Pyridyl)butylamine | 0.88 | 0.71 | 0.71 | 0.17 |
| 900 | Sulfadimethoxine | 1.63 | 1.80 | 1.83 | -0.17 |
| 901 | 2-Hydroxybenzonitrile | 1.61 | 1.44 | 1.44 | 0.17 |
| 902 | Chloroacetamide | -0.53 | -0.70 | -0.72 | 0.17 |
| 903 | 4-Tolyl N-methylcarbamate | 1.66 | 1.83 | 1.80 | -0.17 |
| 904 | Formoterol | 1.91 | 2.08 | 2.06 | -0.17 |
| 905 | Metribuzin | 1.58 | 1.41 | 1.36 | 0.17 |
| 906 | Benzanilide | 2.70 | 2.87 | 2.93 | -0.17 |
| 907 | Dodecane | 6.80 | 6.63 | 6.65 | 0.17 |
| 908 | N-Phenylpiperidine | 2.78 | 2.95 | 2.92 | -0.17 |
| 909 | 4-Bromonitrobenzene | 2.55 | 2.72 | 2.74 | -0.17 |
| 910 | 2,3,3',4,4',5-Hexabromodiphenyl ether | 7.93 | 7.76 | 7.78 | 0.17 |
| 911 | Methotrimeprazine | 4.68 | 4.51 | 4.40 | 0.17 |
| 912 | Imazaquin | 1.86 | 2.03 | 2.13 | -0.17 |
| 913 | n-Octanoic acid | 2.11 | 2.28 | 2.33 | -0.17 |
| 914 | 2,2',3,4,4',5'-Hexachlorobiphenyl | 7.25 | 7.07 | 7.05 | 0.18 |
| 915 | 2,2',3,4,4',5-Hexachlorobiphenyl | 7.25 | 7.07 | 7.03 | 0.18 |
| 916 | 2,3,3',4',5,6-Hexachlorobiphenyl | 7.25 | 7.07 | 7.05 | 0.18 |
| 917 | 2,3,3',4,4',6-Hexachlorobiphenyl | 7.25 | 7.07 | 7.05 | 0.18 |
| 918 | Benzo[b]furan | 2.67 | 2.49 | 2.45 | 0.18 |
| 919 | 4-Methyl-8-quinolinol | 2.36 | 2.18 | 2.16 | 0.18 |
| 920 | Vitavax | 2.14 | 2.32 | 2.37 | -0.18 |
| 921 | 4-Phenylphenol | 3.20 | 3.38 | 3.42 | -0.18 |
| 922 | 3,5-Dimethyl-N-nitrosopiperidine | 1.53 | 1.35 | 1.35 | 0.18 |
| 923 | 3-Nitroquinoline | 1.97 | 2.15 | 2.19 | -0.18 |
| 924 | Ethyl 2-furancarboxylate | 1.52 | 1.34 | 1.26 | 0.18 |
| 925 | o-Anthranilic acid | 1.26 | 1.08 | 1.11 | 0.18 |
| 926 | 4-Aminosalicylic acid | 0.87 | 0.69 | 0.67 | 0.18 |
| 927 | 1,2-Dichloropropane | 1.99 | 1.81 | 1.79 | 0.18 |
| 928 | N,N-Diethyl-N'-methyl-N'-nitrosourea | 1.11 | 0.93 | 0.87 | 0.18 |
| 929 | Sulfameter | 0.41 | 0.23 | 0.22 | 0.18 |
| 930 | Thiazole | 0.44 | 0.26 | 0.31 | 0.18 |
| 931 | Barbituric acid | -1.44 | -1.26 | -1.32 | -0.18 |
| 932 | 4-Hydroxymethylpyridine | 0.06 | -0.12 | -0.12 | 0.18 |
| 933 | 1,4-Dioxane | -0.27 | -0.09 | 0.03 | -0.18 |
| 934 | 3-Aminopyridine | 0.11 | -0.07 | -0.04 | 0.18 |
| 935 | alpha-Bromobutyric acid | 1.42 | 1.24 | 1.27 | 0.18 |
| 936 | Proxyphylline | -0.77 | -0.59 | -0.46 | -0.18 |
| 937 | 1,2,3,7,8-Pentachlorodibenzo-p-dioxin | 6.64 | 6.46 | 6.46 | 0.18 |
| 938 | 1,2,4,7,8-Pentachlorodibenzo-p-dioxin | 6.64 | 6.46 | 6.44 | 0.18 |
| 939 | Pyrazon | 1.14 | 0.96 | 0.89 | 0.18 |
| 940 | Devrinol | 3.36 | 3.54 | 3.54 | -0.18 |
| 941 | Oxycarboxin | 0.74 | 0.92 | 0.88 | -0.18 |
| 942 | 1,1,1,2-Tetrachloroethane | 2.62 | 2.44 | 2.43 | 0.18 |
| 943 | 3-Nitroaniline | 1.37 | 1.19 | 1.20 | 0.18 |
| 944 | 4-Nitroaniline | 1.37 | 1.19 | 1.23 | 0.18 |
| 945 | 2,2',5-Trichlorobiphenyl | 5.60 | 5.42 | 5.36 | 0.18 |
| 946 | 3-Bromoaniline | 2.10 | 1.92 | 1.91 | 0.18 |
| 947 | 3-Chlorodibenzofuran | 4.35 | 4.17 | 4.21 | 0.18 |
| 948 | 2,2,2-Trichloroethanol | 1.35 | 1.17 | 1.09 | 0.18 |
| 949 | Indol-3-ylacetic acid | 1.41 | 1.59 | 1.55 | -0.18 |
| 950 | Indene | 2.92 | 3.10 | 3.07 | -0.18 |
| 951 | o-Toluidine | 1.43 | 1.61 | 1.59 | -0.18 |
| 952 | 3-Bromo-4-chlorophenoxyacetic acid | 2.75 | 2.57 | 2.60 | 0.18 |
| 953 | 3-Methylstyrene | 3.35 | 3.53 | 3.56 | -0.18 |
| 954 | 4-Methylstyrene | 3.35 | 3.53 | 3.56 | -0.18 |
| 955 | 2,2',4,4',5,6'-Hexachlorobiphenyl | 6.89 | 7.07 | 7.12 | -0.18 |
| 956 | PCNB | 4.64 | 4.82 | 4.84 | -0.18 |
| 957 | Cetane | 8.86 | 8.67 | 8.69 | 0.19 |
| 958 | Voltaren | 4.51 | 4.32 | 4.28 | 0.19 |
| 959 | Bromobenzene | 2.99 | 2.80 | 2.78 | 0.19 |
| 960 | 1-Methylfluorene | 4.97 | 4.78 | 4.82 | 0.19 |
| 961 | Hexachlorobutadiene | 4.78 | 4.97 | 5.00 | -0.19 |
| 962 | 4-Isopropylaniline | 2.47 | 2.28 | 2.30 | 0.19 |
| 963 | Linuron | 3.20 | 3.01 |  | 0.19 |
| 964 | 3-Phenylphenoxyacetic acid | 3.18 | 2.99 | 2.97 | 0.19 |
| 965 | 1,2-Dibromobenzene | 3.64 | 3.45 | 3.45 | 0.19 |
| 966 | 5-Methyl-8-quinolinol | 2.37 | 2.18 | 2.18 | 0.19 |
| 967 | 17a-Hydroxyprogesterone | 3.17 | 3.36 | 3.32 | -0.19 |
| 968 | 3-Ethoxy-4-hydroxybenzaldehyde | 1.58 | 1.39 | 1.38 | 0.19 |
| 969 | 7-Azaindole | 1.82 | 1.63 | 1.60 | 0.19 |
| 970 | N-(2-Methoxyphenyl)sulfanilamide | 1.56 | 1.37 | 1.41 | 0.19 |
| 971 | 3-Chlorobenzenesulfonamide | 1.29 | 1.10 | 1.05 | 0.19 |
| 972 | 4'-Hydroxypropiophenone | 2.03 | 1.84 | 1.87 | 0.19 |
| 973 | Butyrophenone | 2.77 | 2.58 | 2.56 | 0.19 |
| 974 | Carbutamide | 1.01 | 0.82 | 0.88 | 0.19 |
| 975 | 3-Chlorophenol | 2.50 | 2.31 | 2.29 | 0.19 |
| 976 | 3-Ethylphenoxyacetic acid | 2.25 | 2.06 | 2.03 | 0.19 |
| 977 | 2-Fluorophenoxyacetic acid | 1.26 | 1.45 | 1.47 | -0.19 |
| 978 | Ethylurea | -0.74 | -0.55 | -0.54 | -0.19 |
| 979 | 3-Phenylpropyldimethylamine | 2.73 | 2.54 | 2.48 | 0.19 |
| 980 | 4-Nitrophenoxyacetic acid | 1.48 | 1.29 | 1.27 | 0.19 |
| 981 | 4,5-Dimethyl-8-quinolinol | 2.71 | 2.52 | 2.50 | 0.19 |
| 982 | 4-Chlorobenzylalcohol | 1.96 | 1.77 | 1.77 | 0.19 |
| 983 | 1,4-Dichlorobenzene | 3.44 | 3.25 | 3.27 | 0.19 |
| 984 | 2,6-Dimethyl-N,N'-dinitrosopiperazine | 0.08 | -0.11 | -0.14 | 0.19 |
| 985 | 3-(4-pyridyl)propionamide | -0.25 | -0.06 | -0.10 | -0.19 |
| 986 | Epichlorohydrin | 0.45 | 0.64 | 0.69 | -0.19 |
| 987 | Deprenyl | 2.90 | 2.71 | 2.67 | 0.19 |
| 988 | N-Nitrosodiisopropylamine | 1.63 | 1.44 | 1.39 | 0.19 |
| 989 | Fluconazole | 0.50 | 0.69 | 0.78 | -0.19 |
| 990 | 4,beta-Dinitrostyrene | 1.89 | 2.08 | 2.12 | -0.19 |
| 991 | Formanilide | 1.15 | 1.34 | 1.34 | -0.19 |
| 992 | 5-Aminoquinoline | 1.16 | 1.35 | 1.39 | -0.19 |
| 993 | Methadone | 3.93 | 4.12 | 4.07 | -0.19 |
| 994 | Fenchlorphos | 5.07 | 4.88 | 4.87 | 0.19 |
| 995 | Diltiazem | 2.70 | 2.89 | 2.82 | -0.19 |
| 996 | Thymol | 3.30 | 3.11 | 3.10 | 0.19 |
| 997 | Amoxicillin | -1.71 | -1.90 | -1.87 | 0.19 |
| 998 | Benzylthiocyanate | 1.99 | 2.18 |  | -0.19 |
| 999 | Diclobenil | 2.74 | 2.93 | 3.00 | -0.19 |
| 1000 | 2,4',5-Trichlorodiphenyl ether | 5.70 | 5.51 | 5.55 | 0.19 |
| 1001 | Chlordane | 6.20 | 6.01 | 5.85 | 0.19 |
| 1002 | Octachloronaphthalene | 7.70 | 7.51 | 7.46 | 0.19 |
| 1003 | (Chloromethyl)benzene | 2.30 | 2.49 | 2.52 | -0.19 |
| 1004 | Fenitrothion | 3.30 | 3.49 | 3.58 | -0.19 |
| 1005 | Tridecane | 7.33 | 7.14 | 7.18 | 0.19 |
| 1006 | Decane | 5.80 | 5.61 | 5.65 | 0.19 |
| 1007 | Undecane | 6.31 | 6.12 | 6.15 | 0.19 |
| 1008 | 3-p-Anisoyl-3-bromoacrylic acid | 1.74 | 1.93 | 1.95 | -0.19 |
| 1009 | 1,3-Dimethoxybenzene | 2.21 | 2.01 | 2.01 | 0.20 |
| 1010 | Allyl phenyl ether | 2.94 | 2.74 | 2.75 | 0.20 |
| 1011 | 2,3,4-Trichlorophenol | 3.61 | 3.41 | 3.45 | 0.20 |
| 1012 | Trichloroethylene | 2.61 | 2.41 | 2.41 | 0.20 |
| 1013 | 6-Chloroquinoxaline | 2.10 | 1.90 | 1.91 | 0.20 |
| 1014 | Betamethasone | 2.06 | 1.86 |  | 0.20 |
| 1015 | Styrene | 2.95 | 3.15 | 3.25 | -0.20 |
| 1016 | N,N-Dimethyl-4-methoxybenzamide | 0.96 | 1.16 | 1.21 | -0.20 |
| 1017 | 2-Naphthoxyacetic acid | 2.53 | 2.33 | 2.29 | 0.20 |
| 1018 | 3-Cyanophenyl N-methylcarbamate | 0.97 | 1.17 | 1.13 | -0.20 |
| 1019 | 4-Fluoroacetanilide | 1.47 | 1.67 | 1.71 | -0.20 |
| 1020 | Acenaphthylene | 4.03 | 3.83 | 3.89 | 0.20 |
| 1021 | Benzo[a]pyrene | 5.97 | 6.17 | 6.28 | -0.20 |
| 1022 | Methyl cellosolve | -0.77 | -0.57 | -0.56 | -0.20 |
| 1023 | 2-Hydroxybenzamide | 0.89 | 0.69 | 0.72 | 0.20 |
| 1024 | 1,7-Phenanthroline | 2.51 | 2.31 | 2.29 | 0.20 |
| 1025 | 4-Pentoxyphenol | 3.26 | 3.06 | 3.03 | 0.20 |
| 1026 | Prometon | 2.99 | 3.19 | 3.22 | -0.20 |
| 1027 | 3-Bromophenoxyacetic acid | 2.22 | 2.02 | 2.04 | 0.20 |
| 1028 | Quinoline | 2.03 | 2.23 | 2.27 | -0.20 |
| 1029 | 4-Trifluoromethylbenzamide | 1.71 | 1.51 | 1.57 | 0.20 |
| 1030 | 3-Hydroxybenzamide | 0.39 | 0.19 | 0.21 | 0.20 |
| 1031 | Acetaldehyde | 0.45 | 0.25 | 0.28 | 0.20 |
| 1032 | 1,3-Dimethyl-2-nitrobenzene | 2.95 | 2.75 | 2.72 | 0.20 |
| 1033 | 3-Chloro-5-fluorophenoxyacetic acid | 2.20 | 2.00 | 1.98 | 0.20 |
| 1034 | 3-(3-pyridyl)propionamide | -0.26 | -0.06 | -0.06 | -0.20 |
| 1035 | Propionic acid | 0.33 | 0.53 | 0.53 | -0.20 |
| 1036 | 4-Amino-2-fluorobenzoic acid | 0.46 | 0.66 | 0.62 | -0.20 |
| 1037 | 5,8-Dihydroxy-4-methylquinoline | 1.59 | 1.79 | 1.79 | -0.20 |
| 1038 | 5-Nitroquinoline-1-oxide | 0.49 | 0.69 | 0.65 | -0.20 |
| 1039 | 3-Methoxysalicylaldehyde | 1.37 | 1.57 | 1.66 | -0.20 |
| 1040 | 2,4,4'-Trichlorobiphenyl | 5.62 | 5.42 | 5.46 | 0.20 |
| 1041 | 2-Chlorodibenzofuran | 4.37 | 4.17 | 4.09 | 0.20 |
| 1042 | Benzylaniline | 3.13 | 3.33 | 3.33 | -0.20 |
| 1043 | 1-Isopropyl-4-methylbenzene | 4.10 | 3.90 | 3.91 | 0.20 |
| 1044 | o-Cymene | 4.10 | 3.90 | 3.91 | 0.20 |
| 1045 | 4-Fluoroaniline | 1.15 | 1.35 | 1.38 | -0.20 |
| 1046 | 4-Amino-6-phenyl-3-(methylthio)-1,2,4-triazin-5-one | 1.66 | 1.86 | 2.00 | -0.20 |
| 1047 | 2,4-Dichlorophenol | 3.06 | 2.86 | 2.88 | 0.20 |
| 1048 | Dopa | -1.70 | -1.90 | -1.96 | 0.20 |
| 1049 | Tetrahydropyran | 0.95 | 1.15 | 1.23 | -0.20 |
| 1050 | 1-(2-Hydroxyphenyl)-1-propanone | 2.54 | 2.34 | 2.35 | 0.20 |
| 1051 | Hexachlorocyclopentadiene | 5.04 | 4.84 | 4.78 | 0.20 |
| 1052 | Clonazepam | 3.02 | 2.82 | 2.83 | 0.20 |
| 1053 | 4-Ethoxyphenol | 1.81 | 2.01 | 2.01 | -0.20 |
| 1054 | 2,3,3',5'-Tetrachlorobiphenyl | 6.17 | 5.97 | 5.97 | 0.20 |
| 1055 | 2,2',3,4',5,6'-Hexachlorobiphenyl | 6.87 | 7.07 | 7.03 | -0.20 |
| 1056 | 2,2',3,4',5-Pentachlorobiphenyl | 6.32 | 6.52 | 6.48 | -0.20 |
| 1057 | Triflumethazine | 4.36 | 4.15 | 4.24 | 0.21 |
| 1058 | 2-Chlorobiphenyl | 4.53 | 4.32 | 4.26 | 0.21 |
| 1059 | 2,3,4',6-Tetrachlorobiphenyl | 5.76 | 5.97 | 5.97 | -0.21 |
| 1060 | Terbutryn | 3.74 | 3.53 |  | 0.21 |
| 1061 | 7-Methoxyquinoline | 2.37 | 2.16 | 2.16 | 0.21 |
| 1062 | Vinclozolin | 3.10 | 2.89 | 2.83 | 0.21 |
| 1063 | 5-(2-Pyridyl)pentylamine | 1.32 | 1.11 | 1.13 | 0.21 |
| 1064 | N,N’-Bianiline | 2.94 | 3.15 | 3.39 | -0.21 |
| 1065 | Bumetanide | 1.96 | 2.17 | 2.22 | -0.21 |
| 1066 | 3,4-Xylenol | 2.23 | 2.44 | 2.48 | -0.21 |
| 1067 | Phenanthrene-9,10-dione | 2.52 | 2.31 | 2.32 | 0.21 |
| 1068 | 2,2',3,4,4',5,6'-Heptachlorodiphenyl ether | 7.50 | 7.71 | 7.72 | -0.21 |
| 1069 | 4-Acetylphenyl N-methylcarbamate | 1.01 | 1.22 | 1.24 | -0.21 |
| 1070 | Ethylenethiourea | -0.66 | -0.45 | -0.39 | -0.21 |
| 1071 | 4-Methoxyphenyl N,N-dimethylcarbamate | 1.53 | 1.74 | 1.71 | -0.21 |
| 1072 | Serotonin | 0.53 | 0.74 | 0.81 | -0.21 |
| 1073 | Methyl formate | 0.03 | -0.18 | -0.21 | 0.21 |
| 1074 | Butyramide | -0.21 | 0.00 | -0.02 | -0.21 |
| 1075 | 2,2,2-Trifluoroethanol | 0.32 | 0.53 | 0.58 | -0.21 |
| 1076 | 1-(2-Cl-5-CF3-phenylhydrazono)-1-cyanoacetic acid methylester | 4.42 | 4.21 | 4.19 | 0.21 |
| 1077 | Pyridine | 0.60 | 0.81 | 0.83 | -0.21 |
| 1078 | 3-Phenyl-1-iodopropane | 3.90 | 3.69 |  | 0.21 |
| 1079 | N-Methyl-p-toluidine | 2.15 | 1.94 | 1.99 | 0.21 |
| 1080 | 2,6-Dimethyl-N-nitrosopiperidine | 1.36 | 1.57 | 1.60 | -0.21 |
| 1081 | Phenethyl alcohol | 1.36 | 1.57 | 1.60 | -0.21 |
| 1082 | m-Toluidine | 1.40 | 1.61 | 1.63 | -0.21 |
| 1083 | Isobutylene | 2.34 | 2.13 | 2.16 | 0.21 |
| 1084 | 3-Isopropylphenoxyacetic acid | 2.59 | 2.38 | 2.35 | 0.21 |
| 1085 | 3,4-Dichlorobenzenesulfonamide | 1.44 | 1.65 | 1.72 | -0.21 |
| 1086 | 3,4-Dichlorophenyl N-methylcarbamate | 2.80 | 2.59 | 2.56 | 0.21 |
| 1087 | 4-Nitrophenetole | 2.53 | 2.32 | 2.30 | 0.21 |
| 1088 | 1-Bromo-2-nitrobenzene | 2.51 | 2.72 | 2.72 | -0.21 |
| 1089 | N,N-Dimethyl-N',N'-diphenylurea | 2.80 | 3.01 | 3.07 | -0.21 |
| 1090 | 2,2',3,3'-Tetrachlorobiphenyl | 6.18 | 5.97 | 6.00 | 0.21 |
| 1091 | 4-Methyl-2-nitroaniline | 1.82 | 2.03 | 2.03 | -0.21 |
| 1092 | 2,2',3,4',5',6-Hexachlorobiphenyl | 6.86 | 7.07 | 7.06 | -0.21 |
| 1093 | 2,2',3,4',6-Pentachlorobiphenyl | 6.31 | 6.52 | 6.54 | -0.21 |
| 1094 | 2,3,4,5,6-Pentachlorobiphenyl | 6.74 | 6.52 | 6.57 | 0.22 |
| 1095 | 1,2,3,7-Tetrachlorodibenzofuran | 6.04 | 5.82 | 5.84 | 0.22 |
| 1096 | 2,2',4,4',6,6'-Hexachlorobiphenyl | 7.29 | 7.07 | 7.03 | 0.22 |
| 1097 | 1,2,4-Trichlorobenzene | 4.02 | 3.80 | 3.80 | 0.22 |
| 1098 | Disulfoton | 4.02 | 3.80 | 3.97 | 0.22 |
| 1099 | 2,2',4,6-Tetrachlorobiphenyl | 5.75 | 5.97 | 5.93 | -0.22 |
| 1100 | 2,3,4,5-Tetrachloroanisole | 4.50 | 4.28 | 4.29 | 0.22 |
| 1101 | 4-Isothiocyanatodiphenylether | 4.75 | 4.97 | 4.96 | -0.22 |
| 1102 | Nitrobenzene | 1.85 | 2.07 | 2.11 | -0.22 |
| 1103 | 3-Bromophenol | 2.63 | 2.41 | 2.43 | 0.22 |
| 1104 | Naloxone | 2.09 | 1.87 | 1.86 | 0.22 |
| 1105 | 4-Biphenylisothiocyanate | 4.66 | 4.88 | 4.92 | -0.22 |
| 1106 | N-Methylaniline | 1.82 | 1.60 | 1.62 | 0.22 |
| 1107 | 2,3-Dimethylphenyl N-methylcarbamate | 1.95 | 2.17 | 2.20 | -0.22 |
| 1108 | 4-Methoxyphenyl N-methylcarbamate | 1.20 | 1.42 | 1.44 | -0.22 |
| 1109 | 4-Cyanophenyl N-methylcarbamate | 0.95 | 1.17 | 1.18 | -0.22 |
| 1110 | Thioacetanilide | 1.71 | 1.93 | 1.92 | -0.22 |
| 1111 | Carbendazim | 1.52 | 1.30 | 1.20 | 0.22 |
| 1112 | Isoniazid | -1.14 | -0.92 | -0.89 | -0.22 |
| 1113 | 2-Ethylaniline | 1.74 | 1.96 | 1.97 | -0.22 |
| 1114 | Aminopyrazine | -0.07 | -0.29 | -0.26 | 0.22 |
| 1115 | 3-Hydroxyphenoxyacetic acid | 0.76 | 0.98 | 1.00 | -0.22 |
| 1116 | Isocarboxazid | 1.49 | 1.27 |  | 0.22 |
| 1117 | N-(3,5-Dibromo-2-pyridyl)sulfanilamide | 2.74 | 2.52 | 2.63 | 0.22 |
| 1118 | 4-Cyanobenzamide | 0.48 | 0.26 | 0.24 | 0.22 |
| 1119 | 3-Hydroxypicolinic acid | -1.27 | -1.49 |  | 0.22 |
| 1120 | t-Butylamine | 0.40 | 0.62 | 0.60 | -0.22 |
| 1121 | 1-Nitropropane | 0.65 | 0.87 | 0.97 | -0.22 |
| 1122 | Dimethylamine | -0.38 | -0.16 | -0.13 | -0.22 |
| 1123 | N-Nitroso-4-piperidone | -0.47 | -0.25 | -0.25 | -0.22 |
| 1124 | Dimethoate | 0.78 | 1.00 | 1.15 | -0.22 |
| 1125 | Tetracosane | 12.53 | 12.75 | 12.85 | -0.22 |
| 1126 | 2-Methoxyisoniazid | -0.10 | -0.32 | -0.37 | 0.22 |
| 1127 | Bromoacetamide | -0.52 | -0.30 | -0.26 | -0.22 |
| 1128 | 5-(4-Pyridyl)pentamide | 0.42 | 0.64 | 0.64 | -0.22 |
| 1129 | N-p-Tolylsulfanilamide | 1.56 | 1.78 | 1.87 | -0.22 |
| 1130 | Ethyl chloride | 1.43 | 1.21 | 1.21 | 0.22 |
| 1131 | Dichlorvos | 1.42 | 1.20 | 1.13 | 0.22 |
| 1132 | N-Methyl-o-toluidine | 2.16 | 1.94 | 1.91 | 0.22 |
| 1133 | 2-Butanol | 0.61 | 0.83 | 0.83 | -0.22 |
| 1134 | 2-Bromopropane | 2.14 | 1.92 | 1.85 | 0.22 |
| 1135 | 3,5-Diethyl-4-hydroxyacetanilide | 2.36 | 2.58 | 2.62 | -0.22 |
| 1136 | N,N-Dimethyl-2-anisidine | 1.63 | 1.85 | 1.77 | -0.22 |
| 1137 | p-Toluidine | 1.39 | 1.61 | 1.63 | -0.22 |
| 1138 | Trimethoprim | 0.91 | 1.13 | 1.06 | -0.22 |
| 1139 | 4-Phenylbutyramide | 1.41 | 1.63 | 1.63 | -0.22 |
| 1140 | 2-Bromotoluene | 2.92 | 3.14 | 3.12 | -0.22 |
| 1141 | 1-s-Butylbenzotriazole | 2.31 | 2.09 | 2.06 | 0.22 |
| 1142 | Monuron | 1.94 | 2.16 | 2.16 | -0.22 |
| 1143 | Glycine | -3.00 | -3.22 | -3.25 | 0.22 |
| 1144 | 2,2',3,5,5',6-Hexachlorobiphenyl | 6.85 | 7.07 | 7.08 | -0.22 |
| 1145 | 1,2,4-Tribromobenzene | 4.32 | 4.10 | 4.13 | 0.22 |
| 1146 | Fluorene | 4.18 | 4.40 | 4.45 | -0.22 |
| 1147 | 2,4',6-Tribromodiphenyl ether | 5.59 | 5.81 | 5.82 | -0.22 |
| 1148 | Chlorazine | 3.24 | 3.46 | 3.59 | -0.22 |
| 1149 | Bromophos | 5.21 | 4.98 | 4.95 | 0.23 |
| 1150 | 2,3,3',4,5,6-Hexachlorobiphenyl | 7.30 | 7.07 | 7.05 | 0.23 |
| 1151 | 4,5,7-Trichloro-2-trifluoromethylbenzimidazole | 3.78 | 4.01 | 4.13 | -0.23 |
| 1152 | 4-Chloro-2,5-dimethylphenyl N-methylcarbamate | 2.95 | 2.72 | 2.74 | 0.23 |
| 1153 | 2,4'-Dichlorobiphenyl | 5.10 | 4.87 | 4.83 | 0.23 |
| 1154 | 2,2,2-Trifluoroethylacetate | 1.18 | 1.41 | 1.42 | -0.23 |
| 1155 | Levobetaxolol | 2.81 | 2.58 | 2.55 | 0.23 |
| 1156 | Phorate | 3.56 | 3.33 | 3.39 | 0.23 |
| 1157 | Stugeron | 5.77 | 5.54 | 5.63 | 0.23 |
| 1158 | Methyl N-methylcarbamate | -0.06 | -0.29 | -0.27 | 0.23 |
| 1159 | 8-Fluoro-4-nitroquinoline-1-oxide | 1.00 | 0.77 | 0.73 | 0.23 |
| 1160 | Benzoylhydrazine | 0.19 | 0.42 | 0.39 | -0.23 |
| 1161 | Carbostyril | 1.26 | 1.49 | 1.55 | -0.23 |
| 1162 | N-Methylmorpholine | -0.33 | -0.10 | -0.07 | -0.23 |
| 1163 | 1-Pentyne | 1.98 | 1.75 | 1.67 | 0.23 |
| 1164 | Diphenhydramine | 3.27 | 3.50 | 3.62 | -0.23 |
| 1165 | Trifluoroacetamide | 0.12 | -0.11 | -0.11 | 0.23 |
| 1166 | Methylphenylsulfoxide | 0.55 | 0.78 | 0.82 | -0.23 |
| 1167 | Triforine | 2.20 | 1.97 | 1.68 | 0.23 |
| 1168 | Latamoxef | -0.58 | -0.81 |  | 0.23 |
| 1169 | Mercaptodimethur | 2.92 | 2.69 | 2.59 | 0.23 |
| 1170 | 4-Nitrophenol | 1.91 | 1.68 | 1.69 | 0.23 |
| 1171 | Phenylglycidyl ether | 1.60 | 1.83 | 1.84 | -0.23 |
| 1172 | 2,6-Dinitrotoluene | 2.10 | 2.33 | 2.31 | -0.23 |
| 1173 | 4-Isothiocyanatophenylbenzoate | 4.90 | 4.67 | 4.75 | 0.23 |
| 1174 | 4-Acetylphenoxyacetic acid | 0.87 | 1.10 | 1.10 | -0.23 |
| 1175 | (L)-Methionine | -1.87 | -2.10 | -2.12 | 0.23 |
| 1176 | 4-Chloronitrobenzene | 2.39 | 2.62 | 2.61 | -0.23 |
| 1177 | N,N'-Dibutyrylurea | 1.40 | 1.63 | 1.55 | -0.23 |
| 1178 | 2-Methylphenanthrene | 5.24 | 5.01 | 4.97 | 0.23 |
| 1179 | 2,2',4,4',5-Pentachlorodiphenyl ether | 6.38 | 6.61 | 6.59 | -0.23 |
| 1180 | 2',3,4-Tribromodiphenyl ether | 5.58 | 5.81 | 5.80 | -0.23 |
| 1181 | 2,2',4-Tribromodiphenyl ether | 5.58 | 5.81 | 5.82 | -0.23 |
| 1182 | 2,4,6-Tribromodiphenyl ether | 5.58 | 5.81 | 5.85 | -0.23 |
| 1183 | 3,3',4-Tribromodiphenyl ether | 5.58 | 5.81 | 5.85 | -0.23 |
| 1184 | 3,4,4'-Tribromodiphenyl ether | 5.58 | 5.81 | 5.82 | -0.23 |
| 1185 | Astemizole | 5.80 | 5.56 | 5.55 | 0.24 |
| 1186 | 2,3,4,4',5,6-Hexachlorobiphenyl | 7.31 | 7.07 | 7.03 | 0.24 |
| 1187 | 2,5-Dimethoxyamphetamine | 1.88 | 1.64 | 1.60 | 0.24 |
| 1188 | 4-Chlorophenylacetic acid | 2.12 | 2.36 | 2.34 | -0.24 |
| 1189 | 4-Amino-6-isopropyl-3-propylthio-1,2,4-triazin-5-one | 2.12 | 1.88 | 1.82 | 0.24 |
| 1190 | Dibenz[aj]anthracene | 7.11 | 6.87 | 6.92 | 0.24 |
| 1191 | Fenoprop | 3.80 | 3.56 | 3.56 | 0.24 |
| 1192 | m-Anthranilic acid | 0.34 | 0.58 | 0.52 | -0.24 |
| 1193 | 3-Chlorotoluene | 3.28 | 3.04 | 3.07 | 0.24 |
| 1194 | 4-Carbamidomethylpyridine | -0.65 | -0.41 | -0.38 | -0.24 |
| 1195 | 2-Methylpropane | 2.76 | 2.52 | 2.58 | 0.24 |
| 1196 | N-(2-Chloroethyl)-N-nitrosourea | 0.57 | 0.33 | 0.33 | 0.24 |
| 1197 | 1-Butanol | 0.88 | 0.64 | 0.63 | 0.24 |
| 1198 | 3-Methoxyphenylacetic acid | 1.50 | 1.74 | 1.76 | -0.24 |
| 1199 | alpha-Chloroisovalerylurea | 1.00 | 0.76 | 0.79 | 0.24 |
| 1200 | Caffeine | -0.07 | -0.31 | -0.26 | 0.24 |
| 1201 | Biphenyl | 4.01 | 4.25 | 4.21 | -0.24 |
| 1202 | s-Butylamine | 0.74 | 0.50 | 0.50 | 0.24 |
| 1203 | Clotiazepam | 3.49 | 3.25 |  | 0.24 |
| 1204 | Isoxazole | 0.08 | 0.32 | 0.35 | -0.24 |
| 1205 | 1,2-Diaminobenzene | 0.15 | 0.39 | 0.38 | -0.24 |
| 1206 | 5-(3-Pyridyl)pentamide | 0.40 | 0.64 | 0.64 | -0.24 |
| 1207 | 4-Methoxyformanilide | 1.03 | 1.27 | 1.32 | -0.24 |
| 1208 | Prostaglandin F2A | 2.28 | 2.52 | 2.51 | -0.24 |
| 1209 | Tropolone | 0.53 | 0.77 | 0.87 | -0.24 |
| 1210 | 1-(2-Pyridinyl)ethanone | 0.83 | 0.59 | 0.62 | 0.24 |
| 1211 | Diclofop-methyl | 4.80 | 5.04 | 5.01 | -0.24 |
| 1212 | Butyranilide | 2.05 | 2.29 | 2.33 | -0.24 |
| 1213 | N-(2-Chloroethyl)-N'-(4-hydroxycyclohexyl)-N-nitrosourea | 1.05 | 1.29 | 1.36 | -0.24 |
| 1214 | Benzenesulfonamide | 0.31 | 0.55 | 0.54 | -0.24 |
| 1215 | Colamine | -1.31 | -1.55 | -1.55 | 0.24 |
| 1216 | Testosterone | 3.32 | 3.56 | 3.56 | -0.24 |
| 1217 | 4-Chloroquinoline-1-oxide | 1.08 | 1.32 | 1.40 | -0.24 |
| 1218 | 1-(2,4-Dinitrophenylhydrazono)-1-cyanoacetone | 3.41 | 3.17 | 3.16 | 0.24 |
| 1219 | 2,5-Dinitrophenol | 1.86 | 2.10 | 2.14 | -0.24 |
| 1220 | 3-Iodophenoxyacetic acid | 2.62 | 2.38 | 2.39 | 0.24 |
| 1221 | Flutamide | 3.35 | 3.11 | 3.05 | 0.24 |
| 1222 | Nimesulide | 2.60 | 2.36 | 2.28 | 0.24 |
| 1223 | 3-Fluorophenylacetic acid | 1.65 | 1.89 | 1.90 | -0.24 |
| 1224 | 2-Chloroquinoline | 2.71 | 2.95 | 2.96 | -0.24 |
| 1225 | Terazosin | 2.29 | 2.05 | 1.96 | 0.24 |
| 1226 | Isobutyranilide | 2.02 | 2.26 | 2.30 | -0.24 |
| 1227 | 1,2-Diphenylethane | 4.79 | 5.03 | 5.22 | -0.24 |
| 1228 | Benzo[b]fluorene | 5.77 | 5.52 | 5.66 | 0.25 |
| 1229 | Chlorpyrifos | 5.27 | 5.02 | 4.97 | 0.25 |
| 1230 | 1,4,5-Trimethylnaphthalene | 4.90 | 4.65 | 4.70 | 0.25 |
| 1231 | p-Cresyl diphenyl phosphate | 4.40 | 4.15 | 4.05 | 0.25 |
| 1232 | 2,2',3,3',4,4'-Hexachlorobiphenyl | 7.32 | 7.07 | 7.12 | 0.25 |
| 1233 | 2,2',3,4'-Tetrachlorobiphenyl | 5.72 | 5.97 | 5.98 | -0.25 |
| 1234 | Flupentixol | 4.51 | 4.76 | 4.84 | -0.25 |
| 1235 | (Ethylthio)benzene | 3.20 | 2.95 | 2.88 | 0.25 |
| 1236 | Azatadine | 3.59 | 3.84 | 3.91 | -0.25 |
| 1237 | delta-HCH | 4.14 | 3.89 | 3.87 | 0.25 |
| 1238 | Isoproturon | 2.87 | 2.62 | 2.57 | 0.25 |
| 1239 | Phenyl-4-tolylcarbinol | 3.13 | 3.38 | 3.30 | -0.25 |
| 1240 | 4-Propylpyridine | 2.10 | 1.85 | 1.89 | 0.25 |
| 1241 | Dibenzo[b,e]pyridine | 3.40 | 3.65 | 3.63 | -0.25 |
| 1242 | 3-Methylcholanthrene | 6.42 | 6.67 | 6.66 | -0.25 |
| 1243 | Azathioprine | 1.07 | 0.82 | 0.45 | 0.25 |
| 1244 | 1-Propanethiol | 1.81 | 1.56 | 1.54 | 0.25 |
| 1245 | 2-Methoxyaniline | 0.95 | 1.20 | 1.22 | -0.25 |
| 1246 | 4-Methoxyaniline | 0.95 | 1.20 | 1.20 | -0.25 |
| 1247 | 2,2',3,4,4',5,5'-Heptachlorodiphenyl ether | 7.46 | 7.71 | 7.67 | -0.25 |
| 1248 | 3-Fluorobenzamide | 0.91 | 0.66 | 0.68 | 0.25 |
| 1249 | 4-Fluorobenzamide | 0.91 | 0.66 | 0.68 | 0.25 |
| 1250 | Methomyl | 0.60 | 0.85 | 1.02 | -0.25 |
| 1251 | 2-Hydroxybenzophenone | 3.52 | 3.27 | 3.32 | 0.25 |
| 1252 | 4-Nitropyridine | 0.48 | 0.73 | 0.73 | -0.25 |
| 1253 | Phenyl N-methylcarbamate | 1.24 | 1.49 | 1.52 | -0.25 |
| 1254 | N-Nitrosodimethylamine | -0.57 | -0.32 | -0.34 | -0.25 |
| 1255 | 2-Azacyclooctanethione | 1.00 | 1.25 | 1.30 | -0.25 |
| 1256 | 4-Cyclopropyl-2-butanone | 1.50 | 1.75 | 1.74 | -0.25 |
| 1257 | Ethanol | -0.31 | -0.06 | -0.05 | -0.25 |
| 1258 | 2-Azacyclopentathione | -0.05 | 0.20 | 0.24 | -0.25 |
| 1259 | 2-Thiouracil | -0.28 | -0.53 | -0.53 | 0.25 |
| 1260 | 1-Nitrobutane | 1.47 | 1.22 | 1.23 | 0.25 |
| 1261 | 2-Chloropyridine | 1.28 | 1.53 | 1.53 | -0.25 |
| 1262 | 3-Phenylpropionamide | 1.03 | 1.28 | 1.22 | -0.25 |
| 1263 | Atenolol | 0.16 | 0.41 | 0.42 | -0.25 |
| 1264 | 2,3,4,5-Tetrachlorophenol | 4.21 | 3.96 | 3.94 | 0.25 |
| 1265 | 2,4,6-Tribromophenol | 3.96 | 3.71 | 3.73 | 0.25 |
| 1266 | trans-3-Phenyl-2-propen-1-ol | 1.95 | 1.70 | 1.71 | 0.25 |
| 1267 | 3-Acetylaminoquinoline | 1.92 | 1.67 | 1.71 | 0.25 |
| 1268 | 2,3,6-Trichlorobiphenyl | 5.67 | 5.42 | 5.37 | 0.25 |
| 1269 | Phenacemide | 0.87 | 1.12 | 1.07 | -0.25 |
| 1270 | 4-Aminosalicylic acid 4-tolylester | 3.38 | 3.13 | 3.12 | 0.25 |
| 1271 | Acetophenone | 1.63 | 1.88 | 1.87 | -0.25 |
| 1272 | 2-Ethylphenyl N-methylcarbamate | 1.93 | 2.18 | 2.16 | -0.25 |
| 1273 | 4-Trifluoromethylaniline | 1.95 | 2.20 | 2.16 | -0.25 |
| 1274 | Bremazocine | 3.20 | 3.45 | 3.55 | -0.25 |
| 1275 | 1,2,4-Trichloro-5-nitrobenzene | 3.47 | 3.72 | 3.70 | -0.25 |
| 1276 | 2,2',3,3',4,6-Hexachlorobiphenyl | 6.82 | 7.07 | 7.08 | -0.25 |
| 1277 | 2,2',4,4',6-Pentabromodiphenyl ether | 6.86 | 7.11 | 7.10 | -0.25 |
| 1278 | Heptylbenzene | 5.37 | 5.11 | 5.15 | 0.26 |
| 1279 | 3-Chlorobiphenyl | 4.58 | 4.32 | 4.36 | 0.26 |
| 1280 | 1-(4-trifluoromethylthiophenylhydrazono)-1-cyanoacetone | 5.04 | 4.78 | 4.78 | 0.26 |
| 1281 | 2-Chloro-1,4-naphthoquinone | 2.15 | 1.89 | 1.68 | 0.26 |
| 1282 | Phenindione | 2.90 | 2.64 | 2.60 | 0.26 |
| 1283 | Atrazine | 2.61 | 2.35 | 2.27 | 0.26 |
| 1284 | Cinnamanilide | 3.61 | 3.35 | 3.26 | 0.26 |
| 1285 | 4-Fluoronitrobenzene | 1.89 | 2.15 | 2.17 | -0.26 |
| 1286 | Tiapride | 0.90 | 1.16 | 1.25 | -0.26 |
| 1287 | 2-Chlorosyringaldehyde | 1.81 | 1.55 | 1.52 | 0.26 |
| 1288 | 3-Methylbenzamide | 1.18 | 0.92 | 0.93 | 0.26 |
| 1289 | Phenylcyclopropylcarbinol | 1.95 | 2.21 | 2.21 | -0.26 |
| 1290 | Piracetam | -1.54 | -1.28 | -1.26 | -0.26 |
| 1291 | n-Butyl acetate | 1.78 | 1.52 | 1.56 | 0.26 |
| 1292 | 3-Cyanobenzamide | 0.52 | 0.26 | 0.29 | 0.26 |
| 1293 | Chloramphenicol | 1.14 | 0.88 | 0.91 | 0.26 |
| 1294 | Lincomycin | 0.20 | -0.06 |  | 0.26 |
| 1295 | 3-(2-pyridyl)propionamide | -0.27 | -0.01 | -0.05 | -0.26 |
| 1296 | (L)-Cysteine | -2.49 | -2.75 | -2.83 | 0.26 |
| 1297 | Pronamide | 3.26 | 3.00 | 2.92 | 0.26 |
| 1298 | Triphenylene | 5.49 | 5.75 | 5.84 | -0.26 |
| 1299 | n-Butylurea | 0.41 | 0.15 | 0.16 | 0.26 |
| 1300 | Phenol | 1.50 | 1.76 | 1.80 | -0.26 |
| 1301 | N,N-Diallylurea | 0.64 | 0.90 | 0.87 | -0.26 |
| 1302 | Trovafloxacin | 0.15 | 0.41 | 0.78 | -0.26 |
| 1303 | 2-Methyl-N,N'-dinitrosopiperazine | -0.28 | -0.54 | -0.57 | 0.26 |
| 1304 | 3-Methyl-4-hydroxyacetanilide | 1.28 | 1.54 | 1.54 | -0.26 |
| 1305 | Ethirimol | 2.22 | 1.96 | 1.98 | 0.26 |
| 1306 | 3-Cyanophenol | 1.70 | 1.44 | 1.40 | 0.26 |
| 1307 | 3-Trifluoromethylphenol | 2.95 | 2.69 | 2.77 | 0.26 |
| 1308 | 4,7-Phenanthroline | 2.05 | 2.31 | 2.32 | -0.26 |
| 1309 | beta-Resorcylic acid | 1.44 | 1.18 | 1.20 | 0.26 |
| 1310 | 3,beta-Dinitrostyrene | 1.82 | 2.08 | 2.03 | -0.26 |
| 1311 | Simazine | 2.18 | 1.92 | 1.87 | 0.26 |
| 1312 | Phenacetin | 1.58 | 1.84 | 1.85 | -0.26 |
| 1313 | 4-Chlorobenzenesulfonamide | 0.84 | 1.10 | 1.02 | -0.26 |
| 1314 | Nitrapyrin | 3.41 | 3.15 | 3.10 | 0.26 |
| 1315 | 4-Iodophenoxyacetic acid | 2.64 | 2.38 | 2.40 | 0.26 |
| 1316 | Benzene | 2.13 | 2.39 | 2.43 | -0.26 |
| 1317 | 4-Aminosalicylic acid 4-chlorophenylester | 3.60 | 3.34 | 3.37 | 0.26 |
| 1318 | Promecarb | 3.10 | 2.84 | 2.84 | 0.26 |
| 1319 | 2,3',4',5-Tetrachlorobiphenyl | 6.23 | 5.97 | 5.92 | 0.26 |
| 1320 | Cyhalothrin | 7.00 | 6.73 | 6.53 | 0.27 |
| 1321 | Pentachlorobenzene | 5.17 | 4.90 | 4.88 | 0.27 |
| 1322 | 3,3',4,4',5-Pentachlorodiphenyl ether | 6.88 | 6.61 | 6.59 | 0.27 |
| 1323 | 3-t-Butylphenyl N-methylcarbamate | 2.93 | 2.66 | 2.66 | 0.27 |
| 1324 | 3-Chlorophenylacetic acid | 2.09 | 2.36 | 2.34 | -0.27 |
| 1325 | Thianaphthene | 3.12 | 2.85 | 2.83 | 0.27 |
| 1326 | Celiprolol | 1.92 | 2.19 | 2.22 | -0.27 |
| 1327 | m-Anisidine | 0.93 | 1.20 | 1.24 | -0.27 |
| 1328 | 2-Chloro-beta-nitrostyrene | 2.44 | 2.71 | 2.80 | -0.27 |
| 1329 | Pentamethylbenzene | 4.56 | 4.29 | 4.33 | 0.27 |
| 1330 | 5-Methyluracil | -0.62 | -0.35 | -0.35 | -0.27 |
| 1331 | Bis(2-chloroethyl) ether | 1.29 | 1.02 | 1.02 | 0.27 |
| 1332 | Anabasine | 0.97 | 1.24 | 1.26 | -0.27 |
| 1333 | 3-Nitrobenzamide | 0.77 | 0.50 | 0.46 | 0.27 |
| 1334 | Acebutolol | 1.77 | 1.50 | 1.46 | 0.27 |
| 1335 | Fensulfothion | 2.23 | 2.50 | 2.72 | -0.27 |
| 1336 | 8-Sulfonamidoquinoline | 0.36 | 0.63 | 0.69 | -0.27 |
| 1337 | 2-Amino-4-pyrimidone | -0.99 | -1.26 | -1.32 | 0.27 |
| 1338 | Phenoxyacetamide | 0.76 | 0.49 | 0.53 | 0.27 |
| 1339 | 4-Amino-3-methylthio-6-propyl-1,2,4-triazine-5-one | 1.01 | 1.28 | 1.25 | -0.27 |
| 1340 | Ethylenedichloride | 1.48 | 1.21 | 1.23 | 0.27 |
| 1341 | 2-Methylphenoxyacetic acid | 1.98 | 1.71 | 1.67 | 0.27 |
| 1342 | Heptachlor | 5.27 | 5.54 | 5.59 | -0.27 |
| 1343 | Azanidazole | 0.85 | 0.58 | 0.57 | 0.27 |
| 1344 | Glycerol | -1.79 | -2.06 | -2.06 | 0.27 |
| 1345 | Allylethylamine | 0.81 | 1.08 | 1.06 | -0.27 |
| 1346 | 3-(Trifluoromethylsulfonyl)phenoxyacetic acid | 2.19 | 1.92 | 1.84 | 0.27 |
| 1347 | 4-Isothiocyanatodiphenylamine | 4.94 | 4.67 | 4.70 | 0.27 |
| 1348 | Tavron | 4.56 | 4.83 | 4.87 | -0.27 |
| 1349 | Benzo[e]pyrene | 6.44 | 6.17 | 6.17 | 0.27 |
| 1350 | Benzonitrile | 1.56 | 1.83 | 1.78 | -0.27 |
| 1351 | 4-Chloroacetanilide | 1.87 | 2.14 | 2.12 | -0.27 |
| 1352 | 1,3,5-Cycloheptatriene | 2.63 | 2.36 | 2.33 | 0.27 |
| 1353 | 2-Hexanone | 1.38 | 1.65 | 1.69 | -0.27 |
| 1354 | 4-Isopropyl-3-methylphenyl N-methylcarbamate | 3.11 | 2.84 | 2.83 | 0.27 |
| 1355 | 2,2-Dimethylpropane | 3.11 | 2.84 | 2.84 | 0.27 |
| 1356 | Dithianon | 2.84 | 2.57 | 2.30 | 0.27 |
| 1357 | Benzo[ghi]fluoranthene | 5.78 | 5.51 | 5.47 | 0.27 |
| 1358 | 1,2,4,5-Tetrachlorobenzene | 4.63 | 4.35 | 4.38 | 0.28 |
| 1359 | 1-(3-Chlorophenylhydrazono)-1-cyanoacetic acid methylester | 3.56 | 3.28 | 3.29 | 0.28 |
| 1360 | Tripropylamine | 2.79 | 2.51 | 2.49 | 0.28 |
| 1361 | Guanabenz | 3.02 | 2.74 | 2.55 | 0.28 |
| 1362 | Hexachlorobenzene | 5.73 | 5.45 | 5.44 | 0.28 |
| 1363 | 2,4,6-Trichlorophenol | 3.69 | 3.41 | 3.39 | 0.28 |
| 1364 | 4-Bromotoluene | 3.42 | 3.14 | 3.11 | 0.28 |
| 1365 | 4-Chloro-8-quinolinol | 2.67 | 2.39 | 2.39 | 0.28 |
| 1366 | 2,4-Dichlorobiphenyl | 5.15 | 4.87 | 4.92 | 0.28 |
| 1367 | 3,4'-Dichlorobiphenyl | 5.15 | 4.87 | 4.83 | 0.28 |
| 1368 | Rivastigmine | 2.10 | 2.38 | 2.33 | -0.28 |
| 1369 | 3-Aminoquinoline | 1.63 | 1.35 | 1.35 | 0.28 |
| 1370 | 4-Aminoquinoline | 1.63 | 1.35 | 1.36 | 0.28 |
| 1371 | 4-Phenyl-N-nitrosopiperidine | 2.59 | 2.31 | 2.27 | 0.28 |
| 1372 | 5-Methoxyindole | 2.06 | 1.78 | 1.76 | 0.28 |
| 1373 | Ethane | 1.81 | 1.53 | 1.53 | 0.28 |
| 1374 | 3-Nitrophenylacetic acid | 1.45 | 1.73 | 1.70 | -0.28 |
| 1375 | 1,3-Dichlorobenzene | 3.53 | 3.25 | 3.29 | 0.28 |
| 1376 | Dimethylether | 0.10 | 0.38 | 0.42 | -0.28 |
| 1377 | 2,3-Dihydro-1,4-benzodioxin | 2.01 | 1.73 | 1.76 | 0.28 |
| 1378 | Chlorthalidone | 0.24 | 0.52 |  | -0.28 |
| 1379 | Trimellitic anhydride | 0.99 | 0.71 | 0.65 | 0.28 |
| 1380 | p-Anthranilic acid | 0.86 | 0.58 | 0.59 | 0.28 |
| 1381 | 1,3-Cyclohexadiene | 2.47 | 2.19 | 2.19 | 0.28 |
| 1382 | 5-Formyluracil | -1.03 | -1.31 | -1.28 | 0.28 |
| 1383 | Practolol | 0.79 | 1.07 | 1.04 | -0.28 |
| 1384 | 2,beta-Dinitrostyrene | 1.80 | 2.08 | 2.12 | -0.28 |
| 1385 | Pervitin | 2.07 | 2.35 | 2.33 | -0.28 |
| 1386 | 3,5-Dimethyl-4-hydroxyacetanilide | 1.60 | 1.88 | 1.87 | -0.28 |
| 1387 | 3-Methoxy-beta-nitrostyrene | 2.37 | 2.09 | 2.08 | 0.28 |
| 1388 | Dicamba | 2.21 | 2.49 | 2.46 | -0.28 |
| 1389 | Permethrin | 6.50 | 6.22 | 6.15 | 0.28 |
| 1390 | 2,2',3,4,5',6-Hexachlorobiphenyl | 6.79 | 7.07 | 7.06 | -0.28 |
| 1391 | Phenothiazine | 3.78 | 4.06 | 4.16 | -0.28 |
| 1392 | 1,2,3,4-Tetrachlorobenzene | 4.64 | 4.35 | 4.35 | 0.29 |
| 1393 | Bifonazole | 4.77 | 4.48 | 4.46 | 0.29 |
| 1394 | 4-Chlorobiphenyl | 4.61 | 4.32 | 4.26 | 0.29 |
| 1395 | 2-Methylindole | 2.53 | 2.82 | 2.78 | -0.29 |
| 1396 | 3-Bromotoluene | 3.43 | 3.14 | 3.18 | 0.29 |
| 1397 | 4-Phenylbut-3-en-2-one | 2.07 | 2.36 | 2.35 | -0.29 |
| 1398 | 2,5-Dichlorobiphenyl | 5.16 | 4.87 | 4.90 | 0.29 |
| 1399 | 2-Phenylphenol | 3.09 | 3.38 | 3.34 | -0.29 |
| 1400 | 4-Methylphenylacetic acid | 1.86 | 2.15 | 2.15 | -0.29 |
| 1401 | 5-Nitroquinoline | 1.86 | 2.15 | 2.14 | -0.29 |
| 1402 | o-Tolyl N,N-dimethylcarbamate | 1.86 | 2.15 | 2.06 | -0.29 |
| 1403 | Indole | 2.14 | 1.85 | 1.85 | 0.29 |
| 1404 | Methylparathion | 2.86 | 3.15 | 3.24 | -0.29 |
| 1405 | Nalidixic acid | 1.59 | 1.30 | 1.24 | 0.29 |
| 1406 | 4-Chlorotoluene | 3.33 | 3.04 | 3.07 | 0.29 |
| 1407 | Carbophenothion | 5.33 | 5.04 | 5.03 | 0.29 |
| 1408 | 5-Methoxy-8-quinolinol | 2.06 | 1.77 | 1.79 | 0.29 |
| 1409 | Ephedrine | 1.56 | 1.27 | 1.29 | 0.29 |
| 1410 | Tyrosine | -1.80 | -1.51 | -1.47 | -0.29 |
| 1411 | 2-Ethoxyethanol | -0.54 | -0.25 | -0.19 | -0.29 |
| 1412 | Nimorazole | 0.07 | -0.22 | -0.21 | 0.29 |
| 1413 | Secnidazole | 0.22 | 0.51 | 0.51 | -0.29 |
| 1414 | 2-Carbamidomethylpyridine | -0.65 | -0.36 | -0.34 | -0.29 |
| 1415 | 4,6-Dinitroquinoline-1-oxide | 0.90 | 0.61 | 0.60 | 0.29 |
| 1416 | 2,4,6-Trichloroanisole | 4.02 | 3.73 | 3.71 | 0.29 |
| 1417 | Quinazolin-4-one | 0.77 | 0.48 | 0.57 | 0.29 |
| 1418 | Salicylanilide | 3.27 | 2.98 | 3.01 | 0.29 |
| 1419 | Scopolamine | 1.24 | 1.53 | 1.57 | -0.29 |
| 1420 | Ethyl iodide | 2.00 | 1.71 |  | 0.29 |
| 1421 | 2-Nitrobenzaldehyde | 1.74 | 1.45 | 1.47 | 0.29 |
| 1422 | Di-n-propylamine | 1.73 | 1.44 | 1.41 | 0.29 |
| 1423 | 4-Fluorophenyl N-methylcarbamate | 1.28 | 1.57 | 1.60 | -0.29 |
| 1424 | 2,3',5'-Trichlorobiphenyl | 5.71 | 5.42 | 5.44 | 0.29 |
| 1425 | 2,4,6-Trichlorobiphenyl | 5.71 | 5.42 | 5.38 | 0.29 |
| 1426 | Benzotrifluoride | 2.79 | 3.08 | 3.07 | -0.29 |
| 1427 | Flupyrsulfuron-methyl | 1.30 | 1.59 |  | -0.29 |
| 1428 | 4-Trifluoromethylphenylacetic acid | 2.45 | 2.74 | 2.77 | -0.29 |
| 1429 | Atomoxetine | 3.70 | 3.99 | 4.01 | -0.29 |
| 1430 | Promazine | 4.55 | 4.26 | 4.24 | 0.29 |
| 1431 | Tetrazepam | 2.76 | 3.05 | 3.21 | -0.29 |
| 1432 | 2,2',4,4',6-Pentachlorobiphenyl | 6.23 | 6.52 | 6.51 | -0.29 |
| 1433 | 3,3',4,4'-Tetrachlorodiphenyl ether | 6.36 | 6.06 | 6.06 | 0.30 |
| 1434 | 2,3,3',5,5',6-Hexachlorobiphenyl | 7.37 | 7.07 | 7.03 | 0.30 |
| 1435 | 1,3-Dibromobenzene | 3.75 | 3.45 | 3.47 | 0.30 |
| 1436 | 3-Propylphenoxyacetic acid | 2.71 | 2.41 | 2.43 | 0.30 |
| 1437 | Phenserine | 4.19 | 3.89 | 3.76 | 0.30 |
| 1438 | 3-Methoxycarbonylphenyl N-methylcarbamate | 1.42 | 1.12 | 1.10 | 0.30 |
| 1439 | Methyl phenylacetate | 1.83 | 2.13 | 2.13 | -0.30 |
| 1440 | Diisopropyl phthalate | 2.83 | 3.13 | 3.11 | -0.30 |
| 1441 | Aminocarb | 1.90 | 1.60 | 1.50 | 0.30 |
| 1442 | 2,3',4,4'-Tetrabromodiphenyl ether | 6.16 | 6.46 | 6.47 | -0.30 |
| 1443 | 2,3',4,6-Tetrabromodiphenyl ether | 6.16 | 6.46 | 6.47 | -0.30 |
| 1444 | 3-Carbamidomethylpyridine | -0.71 | -0.41 | -0.42 | -0.30 |
| 1445 | 3-Hydroxy-beta-nitrostyrene | 2.07 | 1.77 | 1.70 | 0.30 |
| 1446 | 4-Hydroxybenzophenone | 3.07 | 2.77 | 2.82 | 0.30 |
| 1447 | Uracil | -1.07 | -0.77 | -0.82 | -0.30 |
| 1448 | o-Dinitrobenzene | 1.69 | 1.99 | 1.97 | -0.30 |
| 1449 | Benzalmalonodiamide | -0.13 | -0.43 | -0.50 | 0.30 |
| 1450 | 5-Chlorouracil | -0.35 | -0.05 | 0.00 | -0.30 |
| 1451 | Temafloxacin | -0.20 | 0.10 | 0.26 | -0.30 |
| 1452 | 5-Nitroimidazole | -0.16 | 0.14 | 0.24 | -0.30 |
| 1453 | Sulfaperine | 0.34 | 0.64 | 0.59 | -0.30 |
| 1454 | 1-Aminoacridine | 2.47 | 2.77 | 2.79 | -0.30 |
| 1455 | Chlorguanide | 2.53 | 2.23 | 2.02 | 0.30 |
| 1456 | 2,3,4-Trichloroanisole | 4.03 | 3.73 | 3.75 | 0.30 |
| 1457 | 2,3-Dichloronaphthalene | 4.51 | 4.21 | 4.26 | 0.30 |
| 1458 | 4-Aminosalicylic acid 2-bromophenylester | 3.74 | 3.44 | 3.46 | 0.30 |
| 1459 | 5-(2-Pyridyl)pentamide | 0.39 | 0.69 | 0.66 | -0.30 |
| 1460 | 6-Nitroquinoline-1-oxide | 0.39 | 0.69 | 0.70 | -0.30 |
| 1461 | 5,6-Dichlorovanillin | 2.47 | 2.17 | 2.13 | 0.30 |
| 1462 | Tryptophane | -1.04 | -1.34 | -1.30 | 0.30 |
| 1463 | Clofibric acid | 2.57 | 2.87 | 2.89 | -0.30 |
| 1464 | 2-Methoxybenzoic acid | 1.59 | 1.89 | 1.82 | -0.30 |
| 1465 | N-Methylformanilide | 1.09 | 1.39 | 1.45 | -0.30 |
| 1466 | Triasulfuron | 1.60 | 1.90 | 2.02 | -0.30 |
| 1467 | Terbufos | 4.48 | 4.18 | 4.01 | 0.30 |
| 1468 | 9,10-Dimethylanthracene | 5.69 | 5.39 | 5.37 | 0.30 |
| 1469 | 1,2,3,5-Tetrachlorobenzene | 4.66 | 4.35 | 4.32 | 0.31 |
| 1470 | p-Menthadiene | 4.38 | 4.07 | 4.15 | 0.31 |
| 1471 | Coronene | 6.70 | 7.01 | 6.99 | -0.31 |
| 1472 | 2,4,5-Trichlorophenol | 3.72 | 3.41 | 3.39 | 0.31 |
| 1473 | Phenylalanine | -1.43 | -1.12 | -1.17 | -0.31 |
| 1474 | 6-Nitroquinoline | 1.84 | 2.15 | 2.14 | -0.31 |
| 1475 | 2-Cyanophenyl N-methylcarbamate | 0.86 | 1.17 | 1.22 | -0.31 |
| 1476 | Diallylamine | 1.11 | 1.42 | 1.47 | -0.31 |
| 1477 | Styrene oxide | 1.61 | 1.92 | 1.99 | -0.31 |
| 1478 | Isoquinoline | 2.08 | 1.77 | 1.77 | 0.31 |
| 1479 | Isobutylpropylamine | 2.07 | 1.76 | 1.73 | 0.31 |
| 1480 | 3-Cyanophenylacetic acid | 1.18 | 1.49 | 1.55 | -0.31 |
| 1481 | 4,6-Dimethylpyrimidine | 0.62 | 0.31 | 0.33 | 0.31 |
| 1482 | 2-Aminophenol | 0.57 | 0.88 | 0.91 | -0.31 |
| 1483 | Phenylurethane | 2.30 | 1.99 | 2.00 | 0.31 |
| 1484 | 2-Chloroisoniazid | 0.11 | -0.20 | -0.40 | 0.31 |
| 1485 | Acrylamide | -0.67 | -0.36 | -0.38 | -0.31 |
| 1486 | Di-n-propylether | 2.03 | 1.72 | 1.78 | 0.31 |
| 1487 | n-Butylethylether | 2.03 | 1.72 | 1.78 | 0.31 |
| 1488 | Phenobarbital | 1.47 | 1.78 | 1.74 | -0.31 |
| 1489 | Pyrazine | -0.22 | -0.53 | -0.45 | 0.31 |
| 1490 | Allylbenzene | 3.23 | 3.54 | 3.53 | -0.31 |
| 1491 | Diethylketone | 0.99 | 1.30 | 1.30 | -0.31 |
| 1492 | 4-Nitroacetophenone | 1.49 | 1.80 | 1.80 | -0.31 |
| 1493 | 4-Isopropylphenoxyacetic acid | 2.69 | 2.38 | 2.39 | 0.31 |
| 1494 | Methyl isobutyl ketone | 1.31 | 1.62 | 1.61 | -0.31 |
| 1495 | Diphenylsulfoxide | 2.06 | 2.37 | 2.44 | -0.31 |
| 1496 | 2-Trifluoromethylbenzimidazole | 2.67 | 2.36 | 2.36 | 0.31 |
| 1497 | 2-Methylbenzalmalonodinitrile | 2.10 | 2.41 | 2.57 | -0.31 |
| 1498 | Diethion | 5.07 | 4.76 | 4.80 | 0.31 |
| 1499 | 2,4,6-Tribromobiphenyl | 6.03 | 5.72 | 5.76 | 0.31 |
| 1500 | 2,2',3,3',4,5'-Hexachlorobiphenyl | 7.39 | 7.07 | 7.05 | 0.32 |
| 1501 | Methaqualone | 2.50 | 2.82 | 2.93 | -0.32 |
| 1502 | 2,6-Dinitroaniline | 1.79 | 2.11 | 2.16 | -0.32 |
| 1503 | 3,4-Dichloroaniline | 2.69 | 2.37 | 2.32 | 0.32 |
| 1504 | Phenyl-2-tolylcarbinol | 3.06 | 3.38 | 3.41 | -0.32 |
| 1505 | 4-Methylphenylisothiocyanate | 3.92 | 3.60 | 3.56 | 0.32 |
| 1506 | 2,5-Dichloropyridine | 2.40 | 2.08 | 2.05 | 0.32 |
| 1507 | Captopril | 1.02 | 0.70 | 0.56 | 0.32 |
| 1508 | Aldicarb | 1.13 | 0.81 | 0.83 | 0.32 |
| 1509 | 4,6-Dinitro-o-cresol | 2.12 | 2.44 | 2.45 | -0.32 |
| 1510 | Propane | 2.36 | 2.04 | 2.10 | 0.32 |
| 1511 | 2,6-Dichlorodiphenyl ether | 4.64 | 4.96 | 4.95 | -0.32 |
| 1512 | 3-Acetylphenyl N-methylcarbamate | 0.90 | 1.22 | 1.20 | -0.32 |
| 1513 | trans-1,2-Dichloroethylene | 2.09 | 1.77 | 1.64 | 0.32 |
| 1514 | trans-1,3-Dichloropropene | 1.41 | 1.73 | 1.73 | -0.32 |
| 1515 | 2-Methylimidazoline | 0.52 | 0.20 | 0.13 | 0.32 |
| 1516 | 4-Aminobenzamide | 0.02 | -0.30 | -0.32 | 0.32 |
| 1517 | 4-Methoxyphenylacetic acid | 1.42 | 1.74 | 1.73 | -0.32 |
| 1518 | Actidione | 0.55 | 0.87 | 0.88 | -0.32 |
| 1519 | Hydroflumethiazide | 0.54 | 0.22 | -0.01 | 0.32 |
| 1520 | 4-Nitrobenzamide | 0.82 | 0.50 | 0.52 | 0.32 |
| 1521 | Methyl acetate | 0.18 | 0.50 | 0.54 | -0.32 |
| 1522 | Ftorafur | -0.27 | 0.05 |  | -0.32 |
| 1523 | 4-Amino-6-isopropyl-3-isopropylthio-1,2,4-triazin-5-one | 2.06 | 1.74 | 1.51 | 0.32 |
| 1524 | 2-Methoxyphenoxyacetic acid | 0.98 | 1.30 | 1.32 | -0.32 |
| 1525 | Ethyl acetoacetate | 0.24 | 0.56 | 0.52 | -0.32 |
| 1526 | Butylbenzene | 4.26 | 3.94 | 4.02 | 0.32 |
| 1527 | 2-Fluorophenyl N-methylcarbamate | 1.25 | 1.57 | 1.60 | -0.32 |
| 1528 | 3-Nitrophenol | 2.00 | 1.68 | 1.70 | 0.32 |
| 1529 | N,N'-Dimethyl-N-phenylurea | 1.02 | 1.34 | 1.32 | -0.32 |
| 1530 | Aciclovir | -1.56 | -1.88 | -2.01 | 0.32 |
| 1531 | 8-Methoxyquinoline | 1.84 | 2.16 | 2.14 | -0.32 |
| 1532 | 3-phenylpropionic acid | 1.84 | 2.16 | 2.14 | -0.32 |
| 1533 | Trifluoroacetophenone | 2.15 | 2.47 | 2.48 | -0.32 |
| 1534 | 2,2',4,4'-Tetrachlorobiphenyl | 6.29 | 5.97 | 6.00 | 0.32 |
| 1535 | 3-Isopropoxyphenyl N-methylcarbamate | 1.96 | 2.28 | 2.23 | -0.32 |
| 1536 | 2,2',3,4',6,6'-Hexachlorobiphenyl | 6.75 | 7.07 | 7.12 | -0.32 |
| 1537 | 2,3,3',4',6-Pentachlorobiphenyl | 6.20 | 6.52 | 6.48 | -0.32 |
| 1538 | Eicosane | 10.39 | 10.71 | 10.77 | -0.32 |
| 1539 | Hexane | 3.90 | 3.57 | 3.61 | 0.33 |
| 1540 | Mecoprop | 3.13 | 2.80 | 2.75 | 0.33 |
| 1541 | Simvastin | 4.68 | 5.01 | 4.95 | -0.33 |
| 1542 | 1-(3,4,5-Trichlorophenylhydrazono)-1-cyanoacetic acid ethylester | 5.03 | 4.70 | 4.65 | 0.33 |
| 1543 | 2-Phenylthiophene | 3.74 | 3.41 | 3.38 | 0.33 |
| 1544 | Cytarabine | -2.51 | -2.84 | -2.92 | 0.33 |
| 1545 | Thioxanthone | 3.99 | 3.66 | 3.67 | 0.33 |
| 1546 | 7-Nitroquinoline | 1.82 | 2.15 | 2.14 | -0.33 |
| 1547 | Urea | -1.66 | -1.33 | -1.30 | -0.33 |
| 1548 | Debrisoquine | 0.85 | 1.18 | 1.33 | -0.33 |
| 1549 | 4-Nitroquinoline-1-oxide | 1.02 | 0.69 | 0.65 | 0.33 |
| 1550 | Propionanilide | 1.61 | 1.94 | 1.96 | -0.33 |
| 1551 | Pentane | 3.39 | 3.06 | 3.09 | 0.33 |
| 1552 | Butylpropylamine | 2.12 | 1.79 | 1.84 | 0.33 |
| 1553 | Prednisolone | 1.62 | 1.29 | 1.24 | 0.33 |
| 1554 | 5-Methyl-4-nitroquinoline-1-oxide | 1.36 | 1.03 | 1.10 | 0.33 |
| 1555 | Skatole | 2.60 | 2.27 | 2.29 | 0.33 |
| 1556 | 4-Hydroxyphenoxyacetic acid | 0.65 | 0.98 | 0.99 | -0.33 |
| 1557 | Metalaxyl | 1.65 | 1.98 | 1.94 | -0.33 |
| 1558 | 2-Nitroethanol | -0.42 | -0.75 | -0.78 | 0.33 |
| 1559 | N-Nitrosotrimethylurea | 0.36 | 0.03 | -0.01 | 0.33 |
| 1560 | Mefruside | 1.54 | 1.21 | 1.28 | 0.33 |
| 1561 | 3-Acetamidophenoxyacetic acid | 0.48 | 0.81 | 0.81 | -0.33 |
| 1562 | 7-Nitroquinoline-1-oxide | 0.36 | 0.69 | 0.65 | -0.33 |
| 1563 | 2,4',6-Trichlorobiphenyl | 5.75 | 5.42 | 5.43 | 0.33 |
| 1564 | 4-Chlorodiphenyl ether | 4.08 | 4.41 | 4.37 | -0.33 |
| 1565 | Griseofulvin | 2.18 | 2.51 | 2.61 | -0.33 |
| 1566 | 2-Ethoxyphenol | 1.68 | 2.01 | 1.98 | -0.33 |
| 1567 | 2,2',3,4,4'-Pentachlorodiphenyl ether | 6.28 | 6.61 | 6.60 | -0.33 |
| 1568 | 1-(3,5-Dichlorophenylhydrazono)-1-cyanoacetic acid ethylester | 3.82 | 4.15 | 4.19 | -0.33 |
| 1569 | 3,3',4,4',5,5'-Hexachlorobiphenyl | 7.41 | 7.07 | 7.03 | 0.34 |
| 1570 | 1,2,3-Trichlorobenzene | 4.14 | 3.80 | 3.83 | 0.34 |
| 1571 | Butane | 2.89 | 2.55 | 2.56 | 0.34 |
| 1572 | 2,3-Dimethylbutane | 3.85 | 3.51 | 3.54 | 0.34 |
| 1573 | 3,4-Dichlorophenoxyacetic acid | 2.81 | 2.47 | 2.46 | 0.34 |
| 1574 | p-Dibromobenzene | 3.79 | 3.45 | 3.44 | 0.34 |
| 1575 | 3-Cyanobenzoic acid | 1.48 | 1.14 | 1.15 | 0.34 |
| 1576 | Cortisone | 1.47 | 1.13 | 1.08 | 0.34 |
| 1577 | 1-(3-Cl-phenylhydrazono)-1-cyanoacetic acid ethylester | 3.94 | 3.60 | 3.55 | 0.34 |
| 1578 | Flunitrazepam | 2.06 | 2.40 | 2.39 | -0.34 |
| 1579 | n-Hexanoic acid | 1.92 | 1.58 | 1.59 | 0.34 |
| 1580 | 2-(2,4,6-Tribromophenoxy)ethanol | 3.42 | 3.08 | 3.03 | 0.34 |
| 1581 | Digitoxin | 2.83 | 3.17 | 3.23 | -0.34 |
| 1582 | 5-(4-Pyridyl)pentylamine | 1.40 | 1.06 | 1.05 | 0.34 |
| 1583 | 3-Ethyltoluene | 3.88 | 3.54 | 3.56 | 0.34 |
| 1584 | Clobazam | 2.12 | 2.46 | 2.45 | -0.34 |
| 1585 | Methyl benzoate | 2.12 | 1.78 | 1.82 | 0.34 |
| 1586 | Ronidazole | -0.38 | -0.72 | -0.86 | 0.34 |
| 1587 | 4-Nitrophenylacetic acid | 1.39 | 1.73 | 1.75 | -0.34 |
| 1588 | Cephamandole | 0.50 | 0.16 | 0.31 | 0.34 |
| 1589 | 4-Acetaminpyridine | 0.59 | 0.25 | 0.29 | 0.34 |
| 1590 | n-Propylformate | 0.83 | 0.49 | 0.45 | 0.34 |
| 1591 | sec-Phenethyl alcohol | 1.42 | 1.76 | 1.73 | -0.34 |
| 1592 | Butylmethylamine | 1.33 | 0.99 | 0.98 | 0.34 |
| 1593 | 4,5-Dinitroquinoline-1-oxide | 0.95 | 0.61 | 0.56 | 0.34 |
| 1594 | Propachlor | 2.18 | 2.52 | 2.55 | -0.34 |
| 1595 | Nitroethane | 0.18 | 0.52 | 0.59 | -0.34 |
| 1596 | 3-(Methylthio)aniline | 1.45 | 1.79 | 1.71 | -0.34 |
| 1597 | Tetrahydrofuran | 0.46 | 0.80 | 0.86 | -0.34 |
| 1598 | Camphorquinone | 1.52 | 1.18 | 1.15 | 0.34 |
| 1599 | 3-Hydroxybenzylalcohol | 0.49 | 0.83 | 0.81 | -0.34 |
| 1600 | 4-Bromoaniline | 2.26 | 1.92 | 1.91 | 0.34 |
| 1601 | 2,2',4-Trichlorobiphenyl | 5.76 | 5.42 | 5.46 | 0.34 |
| 1602 | 2,3',5-Trichlorobiphenyl | 5.76 | 5.42 | 5.41 | 0.34 |
| 1603 | Phenylformate | 1.26 | 1.60 | 1.67 | -0.34 |
| 1604 | 2,5-Dichlorophenol | 3.20 | 2.86 | 2.86 | 0.34 |
| 1605 | 4-Fluorophenylacetic acid | 1.55 | 1.89 | 1.90 | -0.34 |
| 1606 | Lindane | 3.55 | 3.89 | 3.93 | -0.34 |
| 1607 | 2,2',3,3',4,4'-Hexachlorodiphenyl ether | 6.82 | 7.16 | 7.14 | -0.34 |
| 1608 | 4-Phenylbutylamine | 2.39 | 2.05 | 2.07 | 0.34 |
| 1609 | Phenylvinylketone | 1.88 | 2.22 | 2.24 | -0.34 |
| 1610 | 2,3',4,4'-Tetrachlorobiphenyl | 6.31 | 5.97 | 5.93 | 0.34 |
| 1611 | Lidoflazine | 5.60 | 5.94 | 5.80 | -0.34 |
| 1612 | 2,3,4,5-Tetrachloronitrobenzene | 3.93 | 4.27 | 4.27 | -0.34 |
| 1613 | N-(3,4-Dichlorophenyl)-N'-phenylurea | 4.70 | 4.35 | 4.41 | 0.35 |
| 1614 | Tetradecane | 8.00 | 7.65 | 7.69 | 0.35 |
| 1615 | 1,2,3,4-Tetrachlorodibenzofuran | 6.17 | 5.82 | 5.85 | 0.35 |
| 1616 | Tetrachloroethylene | 3.40 | 3.05 | 2.98 | 0.35 |
| 1617 | N,N-Dimethyl-p-toluidine | 2.61 | 2.26 | 2.31 | 0.35 |
| 1618 | 4-Aminosalicylic acid 4-methoxyphenylester | 3.07 | 2.72 | 2.70 | 0.35 |
| 1619 | N-(3,5-Dichloro-2-pyridyl)sulfanilamide | 1.97 | 2.32 | 2.44 | -0.35 |
| 1620 | 3,4,5-Trichloroveratrole | 4.01 | 3.66 | 3.64 | 0.35 |
| 1621 | 4-s-Butylphenyl N-methylcarbamate | 3.20 | 2.85 | 2.84 | 0.35 |
| 1622 | Niflumic acid | 4.43 | 4.08 | 4.10 | 0.35 |
| 1623 | Perylene | 5.82 | 6.17 | 6.29 | -0.35 |
| 1624 | Physostigmine | 1.58 | 1.93 | 2.20 | -0.35 |
| 1625 | 4-Methoxyphenol | 1.34 | 1.69 | 1.66 | -0.35 |
| 1626 | 5-(3-Pyridyl)pentylamine | 1.41 | 1.06 | 1.06 | 0.35 |
| 1627 | Cinnamamide | 1.41 | 1.06 | 1.07 | 0.35 |
| 1628 | Thiophanate-methyl | 1.40 | 1.05 | 0.79 | 0.35 |
| 1629 | N-Phenylmorpholine | 1.36 | 1.71 | 1.77 | -0.35 |
| 1630 | 4-Hydroxy-beta-nitrostyrene | 2.12 | 1.77 | 1.74 | 0.35 |
| 1631 | 4-Methoxybenzamide | 0.86 | 0.51 | 0.49 | 0.35 |
| 1632 | t-Pentyl alcohol | 0.89 | 1.24 | 1.27 | -0.35 |
| 1633 | Acetic acid | -0.17 | 0.18 | 0.19 | -0.35 |
| 1634 | 5-t-Butyl-3-methylphenyl N-methylcarbamate | 3.35 | 3.00 | 3.00 | 0.35 |
| 1635 | Phentermine | 1.90 | 2.25 | 2.26 | -0.35 |
| 1636 | 2-Aminopyrimidine | -0.22 | 0.13 | 0.05 | -0.35 |
| 1637 | 4-Pyridinamine | 0.28 | -0.07 | -0.03 | 0.35 |
| 1638 | 2,7-Dichloronaphthalene | 4.56 | 4.21 | 4.19 | 0.35 |
| 1639 | 5-Methylquinoxaline | 2.04 | 1.69 | 1.68 | 0.35 |
| 1640 | trans-3-Phenyl-2-propenenitrile | 1.96 | 2.31 | 2.29 | -0.35 |
| 1641 | N-Hydroxy-N'-propylurea | -0.22 | -0.57 | -0.62 | 0.35 |
| 1642 | 2,4-Dinitrotoluene | 1.98 | 2.33 | 2.34 | -0.35 |
| 1643 | Diphenyl ether | 4.21 | 3.86 | 3.93 | 0.35 |
| 1644 | 2,3',4,5-Tetrachlorobiphenyl | 6.32 | 5.97 | 5.93 | 0.35 |
| 1645 | Azulene | 3.20 | 3.55 | 3.61 | -0.35 |
| 1646 | 2,3,3',4,5,5'-Hexachlorobiphenyl | 7.43 | 7.07 | 7.03 | 0.36 |
| 1647 | Methyl trans-cinnamate | 2.62 | 2.26 | 2.24 | 0.36 |
| 1648 | 1,2,3,4,6,7-Hexachloronaphthalene | 6.77 | 6.41 | 6.39 | 0.36 |
| 1649 | Rimsulfuron | 1.30 | 0.94 | 0.78 | 0.36 |
| 1650 | Phenyl aminosalicylate | 3.15 | 2.79 | 2.79 | 0.36 |
| 1651 | 1-Propylbenzotriazole | 2.13 | 1.77 | 1.63 | 0.36 |
| 1652 | Theophylline | 0.00 | -0.36 | -0.39 | 0.36 |
| 1653 | 2-Methoxybenzamide | 0.87 | 0.51 | 0.55 | 0.36 |
| 1654 | Ethylene | 1.13 | 1.49 | 1.50 | -0.36 |
| 1655 | Terbutaline | 1.40 | 1.76 | 1.73 | -0.36 |
| 1656 | Furfural | 0.41 | 0.77 | 0.87 | -0.36 |
| 1657 | 3-Acetylphenyl N,N-dimethylcarbamate | 1.18 | 1.54 | 1.56 | -0.36 |
| 1658 | 2-Methoxyquinoxaline | 2.31 | 1.95 | 1.92 | 0.36 |
| 1659 | (3-Chloropropyl)benzene | 3.55 | 3.19 | 3.18 | 0.36 |
| 1660 | Estriol | 2.45 | 2.81 | 2.78 | -0.36 |
| 1661 | 5-Hexyn-2-one | 0.58 | 0.94 | 1.02 | -0.36 |
| 1662 | 1,1,1-Trifluoro-2-propanol | 0.71 | 1.07 | 1.11 | -0.36 |
| 1663 | 2,4-DB | 3.53 | 3.17 | 3.12 | 0.36 |
| 1664 | 3,4,4'-Trichlorobiphenyl | 5.78 | 5.42 | 5.46 | 0.36 |
| 1665 | 2-Chlorobenzenesulfonamide | 0.74 | 1.10 | 1.12 | -0.36 |
| 1666 | Kentanserin | 3.01 | 2.65 | 2.69 | 0.36 |
| 1667 | Triclosan | 4.76 | 5.12 | 5.09 | -0.36 |
| 1668 | Mescalin | 0.78 | 1.14 | 1.10 | -0.36 |
| 1669 | 3,4-Dichlorotoluene | 3.95 | 3.59 | 3.59 | 0.36 |
| 1670 | Diphenylacetic acid | 3.05 | 3.41 | 3.37 | -0.36 |
| 1671 | Ethyldiisopropylamin | 2.68 | 2.32 | 2.27 | 0.36 |
| 1672 | Dolasteron | 2.42 | 2.78 | 2.62 | -0.36 |
| 1673 | 2,2',3,4,6,6'-Hexachlorobiphenyl | 6.71 | 7.07 | 7.02 | -0.36 |
| 1674 | Exaltol | 4.82 | 4.46 | 4.49 | 0.36 |
| 1675 | Octadecane | 9.32 | 9.69 | 9.79 | -0.37 |
| 1676 | Heptachlor epoxide | 5.40 | 5.03 | 4.96 | 0.37 |
| 1677 | 2,2',4,4',5,6'-Hexabromodiphenyl ether | 7.39 | 7.76 | 7.75 | -0.37 |
| 1678 | Medazepam | 4.05 | 3.68 | 3.85 | 0.37 |
| 1679 | 1-Butanethiol | 2.28 | 1.91 | 1.93 | 0.37 |
| 1680 | Pentachloropyridine | 3.53 | 3.90 | 3.95 | -0.37 |
| 1681 | Clopyralid | 1.07 | 1.44 | 1.48 | -0.37 |
| 1682 | 2-Methoxyphenol | 1.32 | 1.69 | 1.66 | -0.37 |
| 1683 | Lysine | -2.82 | -3.19 | -3.28 | 0.37 |
| 1684 | 4-(3-Pyridyl)butanamide | -0.08 | 0.29 | 0.32 | -0.37 |
| 1685 | Dexfenfluramine | 3.36 | 3.73 | 3.70 | -0.37 |
| 1686 | Pyrrole | 0.75 | 0.38 | 0.43 | 0.37 |
| 1687 | Methyl nicotinate | 0.81 | 0.44 | 0.40 | 0.37 |
| 1688 | 2-Methylpyrazine | 0.23 | -0.14 | -0.18 | 0.37 |
| 1689 | Aniline | 0.90 | 1.27 | 1.30 | -0.37 |
| 1690 | 1,2,3,5,7,8-Hexachloronaphthalene | 7.33 | 6.96 | 6.93 | 0.37 |
| 1691 | Benzohydrol | 2.67 | 3.04 | 3.08 | -0.37 |
| 1692 | Diethanolamine | -1.43 | -1.80 | -1.81 | 0.37 |
| 1693 | 2,4',5-Trichlorobiphenyl | 5.79 | 5.42 | 5.46 | 0.37 |
| 1694 | 2-Bromoaniline | 2.29 | 1.92 | 1.94 | 0.37 |
| 1695 | 2-Tolyl N-methylcarbamate | 1.46 | 1.83 | 1.85 | -0.37 |
| 1696 | Dibenz[ah]anthracene | 6.50 | 6.87 | 6.85 | -0.37 |
| 1697 | 2-Amino-5-methylpyridine | 1.02 | 1.39 | 1.44 | -0.37 |
| 1698 | 2-Bromophenyl N-methylcarbamate | 1.77 | 2.14 | 2.12 | -0.37 |
| 1699 | N,N-Dimethylphenoxyacetamide | 0.77 | 1.14 | 1.14 | -0.37 |
| 1700 | Ethiofencarb | 2.04 | 2.41 | 2.40 | -0.37 |
| 1701 | Chlorpromazine | 5.19 | 4.81 | 4.90 | 0.38 |
| 1702 | 2-t-Butylphenol | 3.31 | 2.93 | 2.94 | 0.38 |
| 1703 | 4-t-Butylphenol | 3.31 | 2.93 | 2.89 | 0.38 |
| 1704 | 1,3,6,8-Tetrachlorodibenzo-p-dioxin | 6.29 | 5.91 | 5.89 | 0.38 |
| 1705 | 3-Chlorobenzamide | 1.51 | 1.13 | 1.15 | 0.38 |
| 1706 | 4-Chlorobenzamide | 1.51 | 1.13 | 1.15 | 0.38 |
| 1707 | 2,5-Dichloroaniline | 2.75 | 2.37 | 2.38 | 0.38 |
| 1708 | 4-Methoxycarbonylphenyl N-methylcarbamate | 1.50 | 1.12 | 1.09 | 0.38 |
| 1709 | Cefatrizine | -2.24 | -1.86 |  | -0.38 |
| 1710 | 2,2'-Dichlorodiisopropyl ether | 2.48 | 2.10 | 2.04 | 0.38 |
| 1711 | Picolinic acid | -1.98 | -1.60 | -1.42 | -0.38 |
| 1712 | 5-Chloro-6-nitro-2-trifluoromethylbenzimidazole | 3.21 | 2.83 | 2.84 | 0.38 |
| 1713 | 2-Acetamido-1,4-naphthoquinone | 1.29 | 0.91 | 0.84 | 0.38 |
| 1714 | 4-(4-Pyridyl)butanamide | -0.09 | 0.29 | 0.26 | -0.38 |
| 1715 | Pirenzepine | 0.60 | 0.98 | 1.01 | -0.38 |
| 1716 | Xylocaine | 2.90 | 2.52 | 2.48 | 0.38 |
| 1717 | Butylcellosolve | 0.83 | 0.45 | 0.37 | 0.38 |
| 1718 | Sulphadiazine | -0.08 | 0.30 | 0.25 | -0.38 |
| 1719 | 1,2,4,5-Tetrabromobenzene | 5.13 | 4.75 | 4.73 | 0.38 |
| 1720 | 4-t-Butyl-3-methylphenyl N-methylcarbamate | 3.38 | 3.00 | 2.97 | 0.38 |
| 1721 | Floxuridine | -1.38 | -1.76 |  | 0.38 |
| 1722 | 4-Acetanisidine | 1.14 | 1.52 | 1.49 | -0.38 |
| 1723 | 4-Amino-3-methylbenzoic acid | 0.54 | 0.92 | 0.92 | -0.38 |
| 1724 | 3-Nitroacetophenone | 1.42 | 1.80 | 1.79 | -0.38 |
| 1725 | Tetroxoprim | 0.56 | 0.94 | 0.97 | -0.38 |
| 1726 | Thiophene | 1.81 | 1.43 | 1.16 | 0.38 |
| 1727 | 1,5-Hexadiene | 2.45 | 2.83 | 2.83 | -0.38 |
| 1728 | Quazepam | 4.03 | 4.41 | 4.54 | -0.38 |
| 1729 | 4,5,6-Trichloroguaiacol | 3.72 | 3.34 | 3.31 | 0.38 |
| 1730 | 4-Aminosalicylic acid 2-chlorophenylester | 3.72 | 3.34 | 3.37 | 0.38 |
| 1731 | 3-Nitrophenylacetate | 1.82 | 2.20 | 2.22 | -0.38 |
| 1732 | Pseudococaine | 2.09 | 2.47 | 2.46 | -0.38 |
| 1733 | Decabromodiphenyl ether | 9.97 | 10.36 | 10.29 | -0.39 |
| 1734 | Heptadecane | 8.79 | 9.18 | 9.32 | -0.39 |
| 1735 | 1,3,5-Trichlorobenzene | 4.19 | 3.80 | 3.81 | 0.39 |
| 1736 | t-Butylbenzene | 4.11 | 3.72 | 3.75 | 0.39 |
| 1737 | 4-Amino-3-chlorobenzoic acid | 1.52 | 1.13 | 1.07 | 0.39 |
| 1738 | 2-Tolualdehyde | 2.26 | 1.87 | 1.86 | 0.39 |
| 1739 | 2-Nitrophenyl N-methylcarbamate | 1.02 | 1.41 | 1.41 | -0.39 |
| 1740 | 7-Methyl-4-nitroquinoline-1-oxide | 1.42 | 1.03 | 1.06 | 0.39 |
| 1741 | Phenazine | 2.84 | 3.23 | 3.19 | -0.39 |
| 1742 | Imazapyr | 0.22 | 0.61 | 0.65 | -0.39 |
| 1743 | Methanol | -0.77 | -0.38 | -0.39 | -0.39 |
| 1744 | N,N,N',N'-Tertramethylethylenediamine | 0.30 | -0.09 | -0.11 | 0.39 |
| 1745 | Flucloxacillin | 2.61 | 3.00 | 3.12 | -0.39 |
| 1746 | Vitamin B3 | -0.37 | -0.76 | -0.80 | 0.39 |
| 1747 | 4-s-Butylphenoxyacetic acid | 3.12 | 2.73 | 2.72 | 0.39 |
| 1748 | Fenarimol | 3.60 | 3.21 | 3.12 | 0.39 |
| 1749 | 5-Allyl-5-butylbarbituric acid | 1.35 | 0.96 | 0.89 | 0.39 |
| 1750 | Methyl-n-propylketone | 0.91 | 1.30 | 1.33 | -0.39 |
| 1751 | 3,3',5-Trichlorobiphenyl | 5.81 | 5.42 | 5.43 | 0.39 |
| 1752 | 4-Methylanisole | 2.81 | 2.42 | 2.40 | 0.39 |
| 1753 | N,N-Dimethylaniline | 2.31 | 1.92 | 1.92 | 0.39 |
| 1754 | Nadolol | 0.71 | 1.10 | 1.10 | -0.39 |
| 1755 | 2-Fluorophenylacetic acid | 1.50 | 1.89 | 1.87 | -0.39 |
| 1756 | Propranolol | 2.98 | 2.59 | 2.60 | 0.39 |
| 1757 | 2,2',4,5,5'-Pentachlorodiphenyl ether | 6.22 | 6.61 | 6.55 | -0.39 |
| 1758 | 2,3,3',4,5-Pentachlorobiphenyl | 6.92 | 6.52 | 6.46 | 0.40 |
| 1759 | Carbazole | 3.72 | 3.32 | 3.29 | 0.40 |
| 1760 | 4-Propylphenol | 3.20 | 2.80 | 2.77 | 0.40 |
| 1761 | 5-Nitro-2-trifluoromethylbenzimidazole | 2.68 | 2.28 | 2.26 | 0.40 |
| 1762 | 1-(2,6-Cl2-4-CF3-phenylhydrazono)-1-cyanoacetic acid methylester | 4.36 | 4.76 | 4.77 | -0.40 |
| 1763 | 4-t-Butylphenyl N-methylcarbamate | 3.06 | 2.66 | 2.59 | 0.40 |
| 1764 | 4-Diethylaminobenzylalcohol | 2.29 | 1.89 | 1.92 | 0.40 |
| 1765 | 2-Chloro-5-nitro-beta-nitrostyrene | 2.23 | 2.63 | 2.67 | -0.40 |
| 1766 | 4-Aminosalicylic acid 4-fluorophenylester | 3.27 | 2.87 | 2.85 | 0.40 |
| 1767 | 4-Iodobenzamide | 1.99 | 1.59 | 1.53 | 0.40 |
| 1768 | Norfloxacin | -1.03 | -1.43 | -1.60 | 0.40 |
| 1769 | Ipronidazole | 1.06 | 1.46 | 1.45 | -0.40 |
| 1770 | 6-Methyl-4-nitroquinoline-1-oxide | 1.43 | 1.03 | 0.99 | 0.40 |
| 1771 | Formaldehyde diethylacetal | 0.84 | 1.24 | 1.31 | -0.40 |
| 1772 | N-Methyl-N-phenylthiourea | 0.85 | 1.25 | 1.35 | -0.40 |
| 1773 | 1,4-Dimethoxy-2,3,5,6-tetrachlorocyclohexane | 3.15 | 2.75 | 2.71 | 0.40 |
| 1774 | Oxirane | -0.30 | 0.10 | 0.19 | -0.40 |
| 1775 | 3,4-Diaminobenzoic acid | -0.70 | -0.30 | -0.30 | -0.40 |
| 1776 | N-Phenylglycine | 0.62 | 1.02 | 1.07 | -0.40 |
| 1777 | 2-Chlorophenyl N-methylcarbamate | 1.64 | 2.04 | 2.08 | -0.40 |
| 1778 | Allylisopropylacetamide | 1.14 | 1.54 | 1.54 | -0.40 |
| 1779 | 4-Aminosalicylic acid 3-bromophenylester | 3.84 | 3.44 | 3.39 | 0.40 |
| 1780 | Phenylacetic acid | 1.41 | 1.81 | 1.85 | -0.40 |
| 1781 | 3,3'-Dichlorobenzidine | 3.51 | 3.11 | 3.05 | 0.40 |
| 1782 | 2,2',3,4,4',6'-Hexachlorodiphenyl ether | 6.76 | 7.16 | 7.12 | -0.40 |
| 1783 | Imipramine | 4.62 | 4.22 | 4.20 | 0.40 |
| 1784 | 1,2,7,8-Tetrachlorodibenzofuran | 6.23 | 5.82 | 5.84 | 0.41 |
| 1785 | 1-(4-CF3S-phenylhydrazono)-1-cyanoacetic acid methylester | 4.27 | 4.68 | 4.79 | -0.41 |
| 1786 | Eradicane | 3.21 | 2.80 | 2.81 | 0.41 |
| 1787 | Gitoxin | 1.68 | 2.09 | 2.14 | -0.41 |
| 1788 | 2,3-Dichloroaniline | 2.78 | 2.37 | 2.35 | 0.41 |
| 1789 | Pheneticillin | 2.28 | 1.87 | 1.87 | 0.41 |
| 1790 | 2-Octanone | 2.76 | 2.35 | 2.34 | 0.41 |
| 1791 | Ethyl trans-cinnamate | 2.99 | 2.58 | 2.60 | 0.41 |
| 1792 | 1,2,3-Trimethoxybenzene | 1.53 | 1.94 | 1.88 | -0.41 |
| 1793 | 4-(Acetylamino)benzoic acid | 1.31 | 0.90 | 0.90 | 0.41 |
| 1794 | 1-Propylamine | 0.48 | 0.07 | 0.06 | 0.41 |
| 1795 | Nopinene | 4.16 | 3.75 | 3.76 | 0.41 |
| 1796 | 1-Pentanol | 1.40 | 0.99 | 0.97 | 0.41 |
| 1797 | Nitromide | 0.83 | 0.42 | 0.38 | 0.41 |
| 1798 | Quinoline-1-oxide | 0.36 | 0.77 | 0.74 | -0.41 |
| 1799 | Benzoic acid | 1.87 | 1.46 | 1.46 | 0.41 |
| 1800 | Primidone | 0.91 | 1.32 | 2.02 | -0.41 |
| 1801 | 4-Cyano-N-formylstyrylamine | 1.58 | 1.17 | 1.01 | 0.41 |
| 1802 | 2,3,3',4',5,5',6-Heptachlorobiphenyl | 7.21 | 7.62 | 7.58 | -0.41 |
| 1803 | 2,3,3',4,4',5',6-Heptachlorobiphenyl | 7.21 | 7.62 | 7.62 | -0.41 |
| 1804 | 2,3,3',4,5,5',6-Heptachlorobiphenyl | 7.21 | 7.62 | 7.60 | -0.41 |
| 1805 | 2-Nitrobenzoic acid | 1.47 | 1.88 | 1.92 | -0.41 |
| 1806 | 2-Methyl-4-nitroquinoline-1-oxide | 1.25 | 1.66 |  | -0.41 |
| 1807 | 2-Nitrophenol | 1.77 | 2.18 | 2.19 | -0.41 |
| 1808 | 4-Ethylphenylacetate | 2.56 | 2.97 | 2.98 | -0.41 |
| 1809 | Veratrole | 1.60 | 2.01 | 2.00 | -0.41 |
| 1810 | 2,2',4,5'-Tetrachlorobiphenyl | 6.38 | 5.97 | 5.97 | 0.41 |
| 1811 | Pyrimethamine | 2.87 | 3.28 | 3.29 | -0.41 |
| 1812 | 1,3,5-Tribromobenzene | 4.51 | 4.10 | 4.12 | 0.41 |
| 1813 | 2,2',4,5',6-Pentachlorobiphenyl | 6.11 | 6.52 | 6.49 | -0.41 |
| 1814 | 2,4,6-Trichlorodibenzofuran | 5.69 | 5.27 | 5.19 | 0.42 |
| 1815 | 2,4,8-Trichlorodibenzofuran | 5.69 | 5.27 | 5.25 | 0.42 |
| 1816 | Clomicalm | 5.19 | 4.77 | 4.79 | 0.42 |
| 1817 | 3-Butylphenoxyacetic acid | 3.18 | 2.76 | 2.77 | 0.42 |
| 1818 | Dichlorprop | 3.43 | 3.01 | 3.02 | 0.42 |
| 1819 | 4-Cyanobenzoic acid | 1.56 | 1.14 | 1.20 | 0.42 |
| 1820 | 1,2,3,7,8-Pentachlorodibenzofuran | 6.79 | 6.37 | 6.40 | 0.42 |
| 1821 | 3,4-Dichlorobiphenyl | 5.29 | 4.87 | 4.92 | 0.42 |
| 1822 | 4-Aminosalicylic acid 2-fluorophenylester | 3.29 | 2.87 | 2.85 | 0.42 |
| 1823 | BCNU | 1.53 | 1.11 | 1.10 | 0.42 |
| 1824 | 2,6-Dichlorosyringaldehyde | 2.52 | 2.10 | 2.08 | 0.42 |
| 1825 | Quinmerac | 0.78 | 1.20 | 2.21 | -0.42 |
| 1826 | Pyridine-N-oxide | -1.69 | -1.27 | -0.78 | -0.42 |
| 1827 | 9-Propyladenine | 0.74 | 0.32 | 0.42 | 0.42 |
| 1828 | Inosine | -2.08 | -2.50 | -2.56 | 0.42 |
| 1829 | 3-Bromobenzamide | 1.65 | 1.23 | 1.26 | 0.42 |
| 1830 | 4-Butoxyphenyl N-methylcarbamate | 2.86 | 2.44 | 2.45 | 0.42 |
| 1831 | Barban | 2.68 | 3.10 | 3.32 | -0.42 |
| 1832 | Diethyl phthalate | 2.47 | 2.05 | 2.03 | 0.42 |
| 1833 | 4-Nonylphenol | 4.48 | 4.90 | 4.96 | -0.42 |
| 1834 | 2,2',4,4',5,5'-Hexachlorobiphenyl | 7.50 | 7.07 | 7.02 | 0.43 |
| 1835 | 2,3',4,4',5,5'-Hexachlorobiphenyl | 7.50 | 7.07 | 7.08 | 0.43 |
| 1836 | MCPA | 2.69 | 2.26 | 2.28 | 0.43 |
| 1837 | Levopropicillin | 2.65 | 2.22 | 2.17 | 0.43 |
| 1838 | 4-Octylphenol | 4.12 | 4.55 | 4.59 | -0.43 |
| 1839 | Terbacil | 1.89 | 2.32 | 2.46 | -0.43 |
| 1840 | 3,3'-Dichlorobiphenyl | 5.30 | 4.87 | 4.83 | 0.43 |
| 1841 | Butyl thiocyanate | 2.03 | 1.60 |  | 0.43 |
| 1842 | Chloropropham | 3.51 | 3.08 | 3.11 | 0.43 |
| 1843 | 3-Carboxyphenol | 1.50 | 1.07 | 1.09 | 0.43 |
| 1844 | 3-Carboxyphenoxyacetic acid | 1.11 | 0.68 | 0.68 | 0.43 |
| 1845 | Hydroxyurea | -1.27 | -1.70 | -1.78 | 0.43 |
| 1846 | Pentachlorobenzimidazole | 4.53 | 4.96 |  | -0.43 |
| 1847 | Nedocromil | 2.22 | 1.79 | 1.68 | 0.43 |
| 1848 | Ethyl vinyl ether | 1.04 | 1.47 | 1.46 | -0.43 |
| 1849 | Perphenazine | 4.20 | 3.77 | 3.75 | 0.43 |
| 1850 | 2-Propanol | 0.05 | 0.48 | 0.47 | -0.43 |
| 1851 | 4-Bromophenoxyacetic acid | 2.45 | 2.02 | 2.04 | 0.43 |
| 1852 | 3-Methoxybenzamide | 0.94 | 0.51 | 0.51 | 0.43 |
| 1853 | N-Nitrosopyrrolidone | -0.19 | 0.24 | 0.32 | -0.43 |
| 1854 | Methyl isonicotinate | 0.87 | 0.44 | 0.46 | 0.43 |
| 1855 | Phenylpropylether | 3.18 | 2.75 | 2.79 | 0.43 |
| 1856 | Oxasulfuron | 1.10 | 1.53 | 1.55 | -0.43 |
| 1857 | Indane-1,3-dione | 0.61 | 1.04 | 1.04 | -0.43 |
| 1858 | Carbenicillin | 1.13 | 1.56 | 1.65 | -0.43 |
| 1859 | Acetanilide | 1.16 | 1.59 | 1.63 | -0.43 |
| 1860 | 4-Methoxy-N,N-dimethylaniline | 1.42 | 1.85 | 1.85 | -0.43 |
| 1861 | 2-Methyl-N-nitrosopiperidine | 0.71 | 1.14 | 1.17 | -0.43 |
| 1862 | 3,4,5-Trichloroguaiacol | 3.77 | 3.34 | 3.32 | 0.43 |
| 1863 | Cortexolone | 2.52 | 2.09 | 2.01 | 0.43 |
| 1864 | Hyoscyamine | 1.83 | 2.26 | 2.26 | -0.43 |
| 1865 | 4-Nitrophenylisothiocyanate | 3.62 | 3.18 | 3.11 | 0.44 |
| 1866 | Alprenolol | 3.10 | 2.66 | 2.64 | 0.44 |
| 1867 | Oxazepam | 2.17 | 2.61 | 2.88 | -0.44 |
| 1868 | 8-Aminoquinoline | 1.79 | 1.35 | 1.30 | 0.44 |
| 1869 | 2,2',3,3',5,5',6,6'-Octachlorobiphenyl | 7.73 | 8.17 | 8.11 | -0.44 |
| 1870 | 1-Nitropentane | 2.01 | 1.57 | 1.52 | 0.44 |
| 1871 | Creatinine | -1.76 | -1.32 | -1.15 | -0.44 |
| 1872 | 1,3-Dichloropropane | 2.00 | 1.56 | 1.58 | 0.44 |
| 1873 | 1-Bromobutane | 2.75 | 2.31 | 2.29 | 0.44 |
| 1874 | 1-Phenyl-2-butanol | 2.02 | 2.46 | 2.42 | -0.44 |
| 1875 | Butyldimethylamine | 1.70 | 1.26 | 1.25 | 0.44 |
| 1876 | Fluoroacetamide | -1.05 | -1.49 | -1.57 | 0.44 |
| 1877 | 6-Azauracil | -0.59 | -0.15 | -0.21 | -0.44 |
| 1878 | Succinic acid | -0.59 | -0.15 | -0.13 | -0.44 |
| 1879 | Adenine | -0.09 | -0.53 | -0.54 | 0.44 |
| 1880 | 2,3,4-Trichlorobiphenyl | 5.86 | 5.42 | 5.38 | 0.44 |
| 1881 | 2-Chloro-1-ethylbenzene | 2.95 | 3.39 | 3.39 | -0.44 |
| 1882 | 3,5-Dichlorophenyl N-methylcarbamate | 3.03 | 2.59 | 2.57 | 0.44 |
| 1883 | 2,2',4,4',5,5'-Hexachlorodiphenyl ether | 6.72 | 7.16 | 7.13 | -0.44 |
| 1884 | Pentadecane | 7.72 | 8.16 | 8.26 | -0.44 |
| 1885 | 2,3,4,5-Tetrachlorobiphenyl | 6.41 | 5.97 | 5.95 | 0.44 |
| 1886 | 2,2',3,5,5'-Pentachlorobiphenyl | 6.97 | 6.52 | 6.52 | 0.45 |
| 1887 | Epiquinine | 3.44 | 2.99 | 2.96 | 0.45 |
| 1888 | Indinavir | 2.92 | 2.47 | 2.40 | 0.45 |
| 1889 | Cyclohexene | 2.86 | 2.41 | 2.46 | 0.45 |
| 1890 | 1-Benzylimidazole | 1.60 | 1.15 | 1.16 | 0.45 |
| 1891 | 2-Aminobiphenyl | 3.34 | 2.89 | 2.85 | 0.45 |
| 1892 | N,N-Dimethyl-2-methoxybenzamide | 0.71 | 1.16 | 1.19 | -0.45 |
| 1893 | N-Nitrosodiethanolamine | -1.51 | -1.96 | -1.96 | 0.45 |
| 1894 | 2-(Hydroxymethyl)-1-methyl-5-nitroimidazole | -0.03 | -0.48 | -0.49 | 0.45 |
| 1895 | 4-Aminobenzenesulfonamide | -0.78 | -0.33 | -0.37 | -0.45 |
| 1896 | Bromocyclohexane | 3.20 | 2.75 | 2.75 | 0.45 |
| 1897 | Procaine | 1.92 | 1.47 | 1.38 | 0.45 |
| 1898 | 3-Fluorobenzalmalonodiamide | 0.10 | -0.35 | -0.35 | 0.45 |
| 1899 | 1,2-Dichloronaphthalene | 4.66 | 4.21 | 4.19 | 0.45 |
| 1900 | 1,4-Dichloronaphthalene | 4.66 | 4.21 | 4.23 | 0.45 |
| 1901 | 1-(4-Cl-2-CF3-phenylhydrazono)-1-cyanoacetic acid methylester | 4.66 | 4.21 | 4.16 | 0.45 |
| 1902 | Diphenylmethylamine | 3.16 | 2.71 | 2.65 | 0.45 |
| 1903 | 2,3',4'-Trichlorobiphenyl | 5.87 | 5.42 | 5.43 | 0.45 |
| 1904 | 4-Chloro-m-cresol | 3.10 | 2.65 | 2.66 | 0.45 |
| 1905 | Dexamisole | 1.94 | 2.39 | 2.56 | -0.45 |
| 1906 | Dyrene | 3.00 | 3.45 | 3.75 | -0.45 |
| 1907 | 1,2,3,4,6,7,8-Heptachlorodibenzofuran | 7.92 | 7.47 | 7.45 | 0.45 |
| 1908 | 8-Chloroquinoline | 2.33 | 2.78 | 2.80 | -0.45 |
| 1909 | Methoxychlor | 5.08 | 5.53 | 5.48 | -0.45 |
| 1910 | 2,2',4,6'-Tetrachlorobiphenyl | 5.51 | 5.97 | 5.97 | -0.46 |
| 1911 | Diphenylsulfone | 2.40 | 2.86 | 2.89 | -0.46 |
| 1912 | Lomustine | 2.83 | 2.37 | 2.34 | 0.46 |
| 1913 | trans-2-Butene | 2.31 | 1.85 | 1.85 | 0.46 |
| 1914 | Pentobarbital | 1.40 | 0.94 | 0.90 | 0.46 |
| 1915 | Midazolam | 3.27 | 3.73 | 3.91 | -0.46 |
| 1916 | Tris(2-chloroethyl) phosphate | 1.48 | 1.02 | 1.28 | 0.46 |
| 1917 | Isopentyl alcohol | 1.42 | 0.96 | 0.99 | 0.46 |
| 1918 | 1,1-Diethoxyethane | 0.84 | 1.30 | 1.33 | -0.46 |
| 1919 | Ketorolac | 2.10 | 2.56 | 2.50 | -0.46 |
| 1920 | 2-Isopropylphenoxyacetic acid | 2.84 | 2.38 | 2.39 | 0.46 |
| 1921 | Mandelic acid ethylester | 0.91 | 1.37 | 1.41 | -0.46 |
| 1922 | Paraldehyde | 0.67 | 1.13 | 1.34 | -0.46 |
| 1923 | 1-Phenyl-2-acetamidocyclopropane | 1.43 | 1.89 | 1.92 | -0.46 |
| 1924 | 1-Acetoxynaphthalene | 2.78 | 3.24 | 3.24 | -0.46 |
| 1925 | 2-Isopropoxyphenol | 2.09 | 2.55 | 2.57 | -0.46 |
| 1926 | 3-(Trifluoromethylthio)phenoxyacetic acid | 2.86 | 3.32 | 3.45 | -0.46 |
| 1927 | 2,2',3,5,6-Pentachlorobiphenyl | 6.06 | 6.52 | 6.51 | -0.46 |
| 1928 | 1,2,3,5-Tetrachloronaphthalene | 5.78 | 5.31 | 5.33 | 0.47 |
| 1929 | 1,2,7,8-Tetrachlorodibenzo-p-dioxin | 6.38 | 5.91 | 5.90 | 0.47 |
| 1930 | Tranexamic acid | -1.87 | -1.40 | -1.30 | -0.47 |
| 1931 | Neohexane | 3.82 | 3.35 | 3.35 | 0.47 |
| 1932 | 4-Ethoxybenzamide | 1.30 | 0.83 | 0.83 | 0.47 |
| 1933 | 2,2',4,4',6,6'-Hexabromobiphenyl | 7.20 | 7.67 | 7.67 | -0.47 |
| 1934 | 2-Butyne | 1.46 | 1.93 | 2.12 | -0.47 |
| 1935 | N-Hydroxyacetamide | -1.54 | -1.07 | -0.99 | -0.47 |
| 1936 | 3-Hydroxyacetanilide | 0.73 | 1.20 | 1.21 | -0.47 |
| 1937 | p-Toluic acid | 2.27 | 1.80 | 1.80 | 0.47 |
| 1938 | Phenylthiourea | 0.73 | 1.20 | 1.10 | -0.47 |
| 1939 | 4,5-Dichloroguaiacol | 3.26 | 2.79 | 2.78 | 0.47 |
| 1940 | Trichlorosyringol | 3.74 | 3.27 | 3.22 | 0.47 |
| 1941 | Benzo[b]fluoranthene | 5.78 | 6.25 | 6.34 | -0.47 |
| 1942 | Trimethadione | -0.37 | 0.10 | 0.23 | -0.47 |
| 1943 | Piperonal | 1.05 | 1.52 | 1.61 | -0.47 |
| 1944 | Allobarbital | 1.07 | 0.60 | 0.55 | 0.47 |
| 1945 | Amantadine | 2.44 | 1.97 | 1.90 | 0.47 |
| 1946 | Buprenorphine | 4.82 | 5.29 | 5.39 | -0.47 |
| 1947 | MCPB | 3.43 | 2.96 | 2.93 | 0.47 |
| 1948 | 2-Aminopyridine | 0.58 | 1.05 | 1.10 | -0.47 |
| 1949 | 1,6-Hexanedioic acid | 0.08 | 0.55 | 0.53 | -0.47 |
| 1950 | 1-Butene | 1.59 | 2.06 | 2.10 | -0.47 |
| 1951 | Hexachloroethane | 4.14 | 3.67 | 3.59 | 0.47 |
| 1952 | 2,6-Dimethoxyphenol | 1.15 | 1.62 | 1.58 | -0.47 |
| 1953 | 3,4-Dichlorophenol | 3.33 | 2.86 | 2.86 | 0.47 |
| 1954 | Promethazine | 4.81 | 4.34 | 4.26 | 0.47 |
| 1955 | Temazepam | 2.19 | 2.66 | 2.92 | -0.47 |
| 1956 | 2,3,8-Trichlorodibenzofuran | 5.75 | 5.27 | 5.29 | 0.48 |
| 1957 | 2,2',3,3',4,4',5,5'-Octachlorodiphenyl ether | 7.78 | 8.26 | 8.21 | -0.48 |
| 1958 | 2-Nonanone | 3.18 | 2.70 | 2.62 | 0.48 |
| 1959 | 1,3,7,9-Tetrachlorodibenzo-p-dioxin | 6.39 | 5.91 | 5.91 | 0.48 |
| 1960 | 2-Chloronaphthalene | 4.14 | 3.66 | 3.71 | 0.48 |
| 1961 | 5-Chloro-2-trifluoromethylbenzimidazole | 3.39 | 2.91 | 2.92 | 0.48 |
| 1962 | 2-(Acetylamino)benzoic acid | 1.88 | 1.40 | 1.42 | 0.48 |
| 1963 | 2-Pyrimidone | -1.62 | -1.14 | -1.15 | -0.48 |
| 1964 | 3-t-Butyl-4-hydroxyacetanilide | 2.85 | 2.37 | 2.33 | 0.48 |
| 1965 | 1-Chloropropane | 2.04 | 1.56 | 1.56 | 0.48 |
| 1966 | 2-Hydroxyacetanilide | 0.72 | 1.20 | 1.21 | -0.48 |
| 1967 | 2-Chlorotoluene | 3.52 | 3.04 | 3.07 | 0.48 |
| 1968 | Zofenopril | 3.50 | 3.02 |  | 0.48 |
| 1969 | 1-Methyluracil | -1.20 | -0.72 | -0.68 | -0.48 |
| 1970 | Methicillin | 1.30 | 1.78 | 1.76 | -0.48 |
| 1971 | Phenylacetamide | 0.45 | 0.93 | 0.87 | -0.48 |
| 1972 | Enanthic acid | 2.41 | 1.93 | 1.92 | 0.48 |
| 1973 | 2,4,5-Trichlorobiphenyl | 5.90 | 5.42 | 5.38 | 0.48 |
| 1974 | Alclofenac | 2.47 | 2.95 | 2.92 | -0.48 |
| 1975 | Norethindrone | 2.97 | 3.45 | 3.57 | -0.48 |
| 1976 | 2,2',3,3',6-Pentachlorobiphenyl | 6.04 | 6.52 | 6.57 | -0.48 |
| 1977 | Progesterone | 3.87 | 4.35 | 4.36 | -0.48 |
| 1978 | Chloroxylenol | 3.48 | 2.99 | 2.99 | 0.49 |
| 1979 | 5,6-Dibromo-2-trifluoromethylbenzimidazole | 4.15 | 3.66 | 3.62 | 0.49 |
| 1980 | Disopyramide | 2.37 | 2.86 | 2.82 | -0.49 |
| 1981 | Cloxacillin | 2.43 | 2.92 | 2.99 | -0.49 |
| 1982 | 4-Aminoacridine | 3.26 | 2.77 | 2.73 | 0.49 |
| 1983 | Isobutylamine | 0.88 | 0.39 | 0.42 | 0.49 |
| 1984 | 3-Methylbenzalmalonodiamide | 0.40 | -0.09 | -0.10 | 0.49 |
| 1985 | N,N'-Diacetylurea | -0.68 | -0.19 | -0.17 | -0.49 |
| 1986 | Pefloxacin | 0.27 | 0.76 | 0.74 | -0.49 |
| 1987 | Finasteride | 3.03 | 3.52 | 3.52 | -0.49 |
| 1988 | Sulfapyridine | -0.02 | 0.47 |  | -0.49 |
| 1989 | 5-Methylindole | 2.68 | 2.19 | 2.16 | 0.49 |
| 1990 | Monocrotophos | -0.20 | -0.69 | -0.72 | 0.49 |
| 1991 | 2,2',3,4,4',5,6-Heptachlorobiphenyl | 7.13 | 7.62 | 7.60 | -0.49 |
| 1992 | Pyrocatechol | 0.88 | 1.37 | 1.42 | -0.49 |
| 1993 | 3,6-Acridinediamine | 1.40 | 1.89 | 1.91 | -0.49 |
| 1994 | N,N-Diethylaniline | 3.31 | 2.82 | 2.79 | 0.49 |
| 1995 | 4,4'-Diisothiocyanatobiphenyl | 5.50 | 5.99 | 5.89 | -0.49 |
| 1996 | Isoxicam | 2.83 | 3.32 | 3.54 | -0.49 |
| 1997 | Octachlorodibenzo-p-dioxin | 8.60 | 8.11 | 8.06 | 0.49 |
| 1998 | 1,3,5,8-Tetrachloronaphthalene | 5.81 | 5.31 | 5.29 | 0.50 |
| 1999 | Diphenylmethane | 4.14 | 4.64 | 4.65 | -0.50 |
| 2000 | 2,3,3',4,4',5-Hexachlorobiphenyl | 7.57 | 7.07 | 7.02 | 0.50 |
| 2001 | Dibenzofuran | 4.12 | 3.62 | 3.67 | 0.50 |
| 2002 | n-Butyl acrylate | 2.36 | 1.86 | 1.90 | 0.50 |
| 2003 | 5,6-Dimethylbenzimidazole | 2.35 | 1.85 | 1.82 | 0.50 |
| 2004 | 2-Ethoxyphenyl N-methylcarbamate | 1.24 | 1.74 | 1.75 | -0.50 |
| 2005 | m-Dinitrobenzene | 1.49 | 1.99 | 2.00 | -0.50 |
| 2006 | p-Dinitrobenzene | 1.49 | 1.99 | 2.03 | -0.50 |
| 2007 | 5-Bromoindole | 3.00 | 2.50 | 2.48 | 0.50 |
| 2008 | 2-Propylpentanoic acid | 2.75 | 2.25 | 2.24 | 0.50 |
| 2009 | 6-Mercaptopurine | -0.18 | 0.32 |  | -0.50 |
| 2010 | Diallyl phthalate | 3.23 | 2.73 | 2.69 | 0.50 |
| 2011 | 1,2,3,4,5,6,8-Heptachloronaphthalene | 7.46 | 6.96 | 6.94 | 0.50 |
| 2012 | 3,3',4,4'-Tetrabromodiphenyl ether | 6.96 | 6.46 | 6.45 | 0.50 |
| 2013 | Piroxicam | 3.06 | 3.56 | 3.78 | -0.50 |
| 2014 | Quinazolin-2,4-dione | 0.20 | 0.70 | 0.66 | -0.50 |
| 2015 | Labetalol | 3.09 | 2.59 | 2.55 | 0.50 |
| 2016 | 2-(Methylthio)phenyl N-methylcarbamate | 1.51 | 2.01 | 2.11 | -0.50 |
| 2017 | 4-(Methylthio)phenol | 1.78 | 2.28 | 2.29 | -0.50 |
| 2018 | Metipranolol | 2.28 | 2.78 | 2.79 | -0.50 |
| 2019 | Bisphenol A | 3.32 | 3.82 | 3.87 | -0.50 |
| 2020 | 2,2',4,4',6-Pentachlorodiphenyl ether | 6.11 | 6.61 | 6.60 | -0.50 |
| 2021 | 1-(3,4-Dichlorophenylhydrazono)-1-cyanoacetic acid ethylester | 4.66 | 4.15 | 4.19 | 0.51 |
| 2022 | Quinine | 3.50 | 2.99 | 2.96 | 0.51 |
| 2023 | DDE | 6.96 | 6.45 | 6.40 | 0.51 |
| 2024 | 1-(2,4,5-Trichlorophenylhydrazono)-1-cyanoacetic acid ethylester | 5.21 | 4.70 | 4.74 | 0.51 |
| 2025 | 3-Nitrobenzoic acid | 1.89 | 1.38 | 1.37 | 0.51 |
| 2026 | 4-Nitrobenzoic acid | 1.89 | 1.38 | 1.37 | 0.51 |
| 2027 | 4-Carboxyphenol | 1.58 | 1.07 | 1.04 | 0.51 |
| 2028 | 1-(4-Carboxybutyl)benzotriazole | 0.93 | 1.44 | 1.68 | -0.51 |
| 2029 | Trichloroacetamide | 1.04 | 0.53 | 0.44 | 0.51 |
| 2030 | 3-Methoxybenzalmalonodiamide | 0.01 | -0.50 | -0.47 | 0.51 |
| 2031 | 4-Amino-3-hydroxybenzoic acid | -0.32 | 0.19 | 0.20 | -0.51 |
| 2032 | Cyclohexylamine | 1.49 | 0.98 | 0.93 | 0.51 |
| 2033 | 2-Isopropylphenylacetate | 2.78 | 3.29 | 3.30 | -0.51 |
| 2034 | 4-Butylbenzenesulfonamide | 2.45 | 1.94 | 1.85 | 0.51 |
| 2035 | 3-Chlorophenylacetate | 2.32 | 2.83 | 2.85 | -0.51 |
| 2036 | 4-Aminosalicylic acid 3-tolylester | 3.64 | 3.13 | 3.13 | 0.51 |
| 2037 | 4-Tolylacetate | 2.11 | 2.62 | 2.65 | -0.51 |
| 2038 | o-Cresylacetate | 2.11 | 2.62 | 2.65 | -0.51 |
| 2039 | 5-Phenyl-2-pentanone | 2.42 | 2.93 | 2.95 | -0.51 |
| 2040 | 1-Phenethylisothiocyanate | 3.46 | 3.97 | 4.08 | -0.51 |
| 2041 | 3,3',5,5'-Tetrachlorobiphenyl | 6.48 | 5.97 | 5.95 | 0.51 |
| 2042 | Lauric acid | 4.20 | 3.68 | 3.62 | 0.52 |
| 2043 | Chloroambucil | 3.70 | 3.18 | 3.05 | 0.52 |
| 2044 | 4-Butylaniline | 3.18 | 2.66 | 2.65 | 0.52 |
| 2045 | 2,2',3,3',4,4',5,5'-Octachlorobiphenyl | 7.65 | 8.17 | 8.11 | -0.52 |
| 2046 | Fomepizole | 0.90 | 1.42 | 1.45 | -0.52 |
| 2047 | Ethinyl Estradiol | 3.42 | 3.94 | 4.06 | -0.52 |
| 2048 | Mebendazole | 2.83 | 2.31 | 2.21 | 0.52 |
| 2049 | Piperazine | -1.17 | -0.65 | -0.49 | -0.52 |
| 2050 | 2-Phenoxyaniline | 2.46 | 2.98 | 3.01 | -0.52 |
| 2051 | N-Methyl-4-nitroaniline | 2.04 | 1.52 | 1.51 | 0.52 |
| 2052 | 2-Methylimidazole | 0.49 | -0.03 | -0.08 | 0.52 |
| 2053 | 2,4,6-Trinitrotoluene | 1.73 | 2.25 | 2.26 | -0.52 |
| 2054 | Di-n-propyl phthalate | 3.27 | 2.75 | 2.71 | 0.52 |
| 2055 | 4-Hydroxy-N-Nitrosopiperidine | -0.89 | -0.37 | -0.37 | -0.52 |
| 2056 | 4-Cyanopyrazole | 0.24 | 0.76 | 0.81 | -0.52 |
| 2057 | 2-Methyl-2-propanol | 0.37 | 0.89 | 0.89 | -0.52 |
| 2058 | Piretanide | 1.50 | 2.02 | 2.05 | -0.52 |
| 2059 | 2,3,5,6-Tetramethyl-1,4-benzoquinone | 2.23 | 1.71 | 1.56 | 0.52 |
| 2060 | 3-Butoxyphenyl N-methylcarbamate | 2.96 | 2.44 | 2.40 | 0.52 |
| 2061 | 2,6-Dihydroxybenzoic acid | 2.20 | 1.68 | 1.72 | 0.52 |
| 2062 | 2-Methoxybenzylalcohol | 1.13 | 1.65 | 1.66 | -0.52 |
| 2063 | Bupranolol | 2.97 | 3.49 | 3.55 | -0.52 |
| 2064 | Thyreoliberin | -2.47 | -2.99 | -3.11 | 0.52 |
| 2065 | 2-Anthracenylisothiocyanate | 5.70 | 5.18 | 5.11 | 0.52 |
| 2066 | Nonylbenzene | 6.41 | 5.89 | 5.78 | 0.52 |
| 2067 | Nortriptyline | 4.04 | 4.56 | 4.63 | -0.52 |
| 2068 | Trifluoromethylthiobenzene | 3.57 | 4.10 | 4.24 | -0.53 |
| 2069 | 2,3,3',4,4',5'-Hexachlorobiphenyl | 7.60 | 7.07 | 7.03 | 0.53 |
| 2070 | Terfenadine | 7.10 | 6.57 | 6.42 | 0.53 |
| 2071 | Alachlor | 3.52 | 2.99 | 3.06 | 0.53 |
| 2072 | 4-Aminosalicylic acid 3-methoxyphenylester | 3.25 | 2.72 | 2.70 | 0.53 |
| 2073 | s-Butylbenzene | 4.44 | 3.91 | 3.92 | 0.53 |
| 2074 | Clenbuterol | 2.94 | 2.41 | 2.38 | 0.53 |
| 2075 | 3,4,5,6-Tetrachloroguaiacol | 4.42 | 3.89 | 3.89 | 0.53 |
| 2076 | 3,5-Dichloroaniline | 2.90 | 2.37 | 2.38 | 0.53 |
| 2077 | Bromophos-ethyl | 6.15 | 5.62 | 5.56 | 0.53 |
| 2078 | Metoprolol | 2.34 | 1.81 | 1.77 | 0.53 |
| 2079 | 4-Fluorobenzoic acid | 2.07 | 1.54 | 1.56 | 0.53 |
| 2080 | Benzenethiol | 2.52 | 1.99 |  | 0.53 |
| 2081 | 4-Bromobenzamide | 1.76 | 1.23 | 1.22 | 0.53 |
| 2082 | Malic acid | -1.26 | -0.73 | -0.66 | -0.53 |
| 2083 | m-Diacetamidobenzene | 0.50 | 1.03 | 1.03 | -0.53 |
| 2084 | 5,6-Dichloro-2-trifluoromethylbenzimidazole | 3.99 | 3.46 | 3.43 | 0.53 |
| 2085 | 4-Bromobiphenyl | 4.95 | 4.42 | 4.38 | 0.53 |
| 2086 | 1-(3-Chlorophenylhydrazono)-1-cyanoacetone | 3.91 | 3.38 | 3.40 | 0.53 |
| 2087 | m-Cresylacetate | 2.09 | 2.62 | 2.65 | -0.53 |
| 2088 | Ropivacaine | 2.90 | 3.43 | 3.41 | -0.53 |
| 2089 | Thiobencarb | 3.40 | 3.93 | 4.06 | -0.53 |
| 2090 | N,N-Dimethyl-m-toluidine | 2.80 | 2.26 | 2.23 | 0.54 |
| 2091 | Hydantoin | -1.69 | -1.15 | -1.04 | -0.54 |
| 2092 | Di-n-butylamine | 2.68 | 2.14 | 2.17 | 0.54 |
| 2093 | Ganciclovir | -2.07 | -2.61 | -2.70 | 0.54 |
| 2094 | 3,5-Dichlorobiphenyl | 5.41 | 4.87 | 4.81 | 0.54 |
| 2095 | Ethyl benzoate | 2.64 | 2.10 | 2.10 | 0.54 |
| 2096 | DMSO | -1.35 | -0.81 | -0.55 | -0.54 |
| 2097 | Rosamicin | 2.19 | 2.73 | 2.68 | -0.54 |
| 2098 | 2-Amino-1,7-dihydro-6H-purine-6-thione | -0.07 | -0.61 | -0.83 | 0.54 |
| 2099 | 2-Iodophenyl N-methylcarbamate | 1.96 | 2.50 | 2.51 | -0.54 |
| 2100 | N-Hydroxy-N'-butylurea | 0.32 | -0.22 | -0.25 | 0.54 |
| 2101 | 2-Methoxyacetanilide | 0.98 | 1.52 | 1.47 | -0.54 |
| 2102 | Dimethyl sulfide | 1.77 | 1.23 | 1.16 | 0.54 |
| 2103 | 9,10-Dihydroanthracene | 4.25 | 4.79 | 4.92 | -0.54 |
| 2104 | N-(2-Norbornyl)-N'-(2-chloroethyl)-N'-nitrosourea | 2.98 | 2.44 | 2.45 | 0.54 |
| 2105 | 4-Hydroxyphenylacetic acid | 1.96 | 1.42 | 1.42 | 0.54 |
| 2106 | 2,3,3',4,4',5,6-Heptachlorobiphenyl | 7.08 | 7.62 | 7.56 | -0.54 |
| 2107 | 1,3,6,8-Tetrachlorodibenzofuran | 6.37 | 5.82 | 5.74 | 0.55 |
| 2108 | Fluvastatin | 4.17 | 4.72 | 4.72 | -0.55 |
| 2109 | 4-Iodobenzoic acid | 3.02 | 2.47 | 2.50 | 0.55 |
| 2110 | Triallate | 4.29 | 4.84 | 4.98 | -0.55 |
| 2111 | 2,3,4,7,8-Pentachlorodibenzofuran | 6.92 | 6.37 | 6.29 | 0.55 |
| 2112 | 4-Aminosalicylic acid 3-fluorophenylester | 3.42 | 2.87 | 2.81 | 0.55 |
| 2113 | 4-Isopropylbenzamide | 2.14 | 1.59 | 1.58 | 0.55 |
| 2114 | 2,3,3',4,4',5,5',6-Octachlorobiphenyl | 7.62 | 8.17 | 8.13 | -0.55 |
| 2115 | Broxuridine | -0.29 | -0.84 | -0.98 | 0.55 |
| 2116 | Imidazole | -0.08 | -0.63 | -0.67 | 0.55 |
| 2117 | 1-Butylamine | 0.97 | 0.42 | 0.41 | 0.55 |
| 2118 | Cefoperazone | -0.74 | -1.29 | -1.49 | 0.55 |
| 2119 | Sulfamerazine | 0.14 | 0.69 | 0.72 | -0.55 |
| 2120 | 5-Chloro-2-methylthiobenzimidazole | 3.22 | 2.67 | 2.51 | 0.55 |
| 2121 | 3-Phenylpropylfluoride | 2.95 | 2.40 | 2.31 | 0.55 |
| 2122 | Trimetrexate | 2.55 | 3.10 | 3.11 | -0.55 |
| 2123 | 4-Cyclopentylphenoxyacetic acid | 3.41 | 2.86 | 2.91 | 0.55 |
| 2124 | 1-Hexene | 3.39 | 2.84 | 2.82 | 0.55 |
| 2125 | 2-Ethylphenylacetate | 2.42 | 2.97 | 2.91 | -0.55 |
| 2126 | Benzyl butyl phthalate | 4.59 | 4.03 | 4.04 | 0.56 |
| 2127 | 5-Bromo-2-trifluoromethylbenzimidazole | 3.57 | 3.01 | 3.03 | 0.56 |
| 2128 | n-Propyl benzoate | 3.01 | 2.45 | 2.48 | 0.56 |
| 2129 | 3-Trifluoromethylbenzoic acid | 2.95 | 2.39 | 2.37 | 0.56 |
| 2130 | Chlorimuron-ethyl | 2.70 | 2.14 | 2.10 | 0.56 |
| 2131 | Acetamide | -1.26 | -0.70 | -0.70 | -0.56 |
| 2132 | Lawsone | 1.38 | 0.82 | 0.76 | 0.56 |
| 2133 | 1-Phenyltetrazole | 1.09 | 0.53 | 0.40 | 0.56 |
| 2134 | 8-Methyl-4-nitroquinoline-1-oxide | 1.59 | 1.03 | 0.99 | 0.56 |
| 2135 | Ethyl nicotinate | 1.32 | 0.76 | 0.74 | 0.56 |
| 2136 | 4-Cyanonitrobenzene | 1.19 | 1.75 | 1.74 | -0.56 |
| 2137 | Famotidine | -0.81 | -1.37 |  | 0.56 |
| 2138 | Benzalacetophenone | 3.08 | 3.64 | 3.64 | -0.56 |
| 2139 | 4-Aminosalicylic acid 3-chlorophenylester | 3.90 | 3.34 | 3.34 | 0.56 |
| 2140 | Penbutolol | 4.15 | 3.59 | 3.61 | 0.56 |
| 2141 | Dicloxacillin | 2.91 | 3.47 | 3.61 | -0.56 |
| 2142 | Papaverine | 2.95 | 3.51 | 3.47 | -0.56 |
| 2143 | 2,2',3,5,6,6'-Hexachlorobiphenyl | 6.51 | 7.07 | 7.03 | -0.56 |
| 2144 | Ethalfluralin | 4.92 | 4.35 | 4.32 | 0.57 |
| 2145 | 1,3,7,8-Tetrachlorodibenzofuran | 6.39 | 5.82 | 5.81 | 0.57 |
| 2146 | 1,3,7,9-Tetrachlorodibenzofuran | 6.39 | 5.82 | 5.85 | 0.57 |
| 2147 | 4-Anisic acid | 1.96 | 1.39 | 1.35 | 0.57 |
| 2148 | Methyl 4-hydroxybenzoate | 1.96 | 1.39 | 1.41 | 0.57 |
| 2149 | Methylsalicylate | 2.46 | 1.89 | 1.84 | 0.57 |
| 2150 | Kepone | 5.44 | 4.87 | 3.66 | 0.57 |
| 2151 | 3-Hydroxyphenylacetic acid | 0.85 | 1.42 | 1.40 | -0.57 |
| 2152 | Amylbenzene | 4.90 | 4.33 | 4.32 | 0.57 |
| 2153 | Chloramben | 1.11 | 1.68 | 1.70 | -0.57 |
| 2154 | m-Toluic acid | 2.37 | 1.80 | 1.77 | 0.57 |
| 2155 | Thiobenzamide | 1.49 | 0.92 | 0.84 | 0.57 |
| 2156 | 2,4-Diaminobenzoic acid | -1.14 | -1.71 | -1.76 | 0.57 |
| 2157 | 4-Amino-2-bromobenzoic acid | 0.66 | 1.23 | 1.21 | -0.57 |
| 2158 | Propyl N-methylcarbamate | 0.95 | 0.38 | 0.32 | 0.57 |
| 2159 | Bendroflumethiazide | 1.19 | 1.76 | 2.19 | -0.57 |
| 2160 | Antipyrine | 0.23 | 0.80 | 1.04 | -0.57 |
| 2161 | Pyrazin-2-one | -1.49 | -2.06 | -2.12 | 0.57 |
| 2162 | Loratadine | 5.20 | 4.63 | 4.44 | 0.57 |
| 2163 | Resorcinol | 0.80 | 1.37 | 1.39 | -0.57 |
| 2164 | Exalgin | 1.07 | 1.64 | 1.67 | -0.57 |
| 2165 | D-Ribose | -2.32 | -2.89 | -2.99 | 0.57 |
| 2166 | 3-Iodoaniline | 2.86 | 2.28 | 2.29 | 0.58 |
| 2167 | 1-Chloronaphthalene | 4.24 | 3.66 | 3.71 | 0.58 |
| 2168 | Chloroprene | 2.03 | 2.61 | 2.65 | -0.58 |
| 2169 | Heptane | 4.66 | 4.08 | 4.12 | 0.58 |
| 2170 | 9-Butyladenine | 1.25 | 0.67 | 0.60 | 0.58 |
| 2171 | Stavudine | -0.81 | -0.23 | -0.15 | -0.58 |
| 2172 | Trimethylphosphate | -0.52 | 0.06 | 0.09 | -0.58 |
| 2173 | 3-Nitrobenzonitrile | 1.17 | 1.75 | 1.79 | -0.58 |
| 2174 | 3-Aminoacridine | 2.19 | 2.77 | 2.76 | -0.58 |
| 2175 | 4-Cyclohexylphenoxyacetic acid | 3.79 | 3.21 | 3.24 | 0.58 |
| 2176 | 4-Hydroxybenzylalcohol | 0.25 | 0.83 | 0.85 | -0.58 |
| 2177 | Diisopropyl ether | 1.52 | 2.10 | 2.09 | -0.58 |
| 2178 | 4-Ethoxyphenylacetate | 1.95 | 2.53 | 2.55 | -0.58 |
| 2179 | 1,2,3,4-Tetrachloronaphthalene | 5.90 | 5.31 | 5.32 | 0.59 |
| 2180 | N,N-Dimethyl-o-toluidine | 2.85 | 2.26 | 2.19 | 0.59 |
| 2181 | Diazinon | 3.81 | 3.22 | 2.84 | 0.59 |
| 2182 | 1-Hexyne | 2.73 | 2.14 | 2.05 | 0.59 |
| 2183 | Pindolol | 1.92 | 1.33 | 1.28 | 0.59 |
| 2184 | 2-Ethylphenoxyacetic acid | 2.65 | 2.06 | 2.06 | 0.59 |
| 2185 | 4,7-Dichloro-2-trifluoromethylbenzimidazole | 2.87 | 3.46 | 3.58 | -0.59 |
| 2186 | Sulfasalazine | 3.61 | 3.02 | 2.69 | 0.59 |
| 2187 | Neopentyl alcohol | 1.36 | 0.77 | 0.77 | 0.59 |
| 2188 | Cetamolol | 1.36 | 0.77 | 0.70 | 0.59 |
| 2189 | 6-Methyl-1,4-naphthoquinone | 2.10 | 1.51 | 1.46 | 0.59 |
| 2190 | 4-Aminoacetophenone | 0.41 | 1.00 | 1.02 | -0.59 |
| 2191 | Baclofen | -0.96 | -0.37 | -0.33 | -0.59 |
| 2192 | 2-s-Butylphenoxyacetic acid | 3.32 | 2.73 | 2.74 | 0.59 |
| 2193 | 2-(Methylthio)aniline | 1.20 | 1.79 | 1.78 | -0.59 |
| 2194 | Buspirone | 2.30 | 1.71 | 1.61 | 0.59 |
| 2195 | Iohexol | -3.05 | -2.46 | -2.39 | -0.59 |
| 2196 | Dinitramine | 4.30 | 3.71 | 3.78 | 0.59 |
| 2197 | 2,2',3,3',4,5,5'-Heptachlorobiphenyl | 7.03 | 7.62 | 7.57 | -0.59 |
| 2198 | Benzyl benzoate | 3.97 | 3.38 | 3.42 | 0.59 |
| 2199 | Octane | 5.18 | 4.59 | 4.61 | 0.59 |
| 2200 | 1,3,7-Trichloronaphthalene | 5.35 | 4.76 | 4.76 | 0.59 |
| 2201 | Tetrachloro-2-trifluoromethylbenzimidazole | 3.97 | 4.56 | 4.68 | -0.59 |
| 2202 | Lovastatin | 4.26 | 4.85 | 4.82 | -0.59 |
| 2203 | 1,2,3,5,8-Pentachloronaphthalene | 6.46 | 5.86 | 5.84 | 0.60 |
| 2204 | 6-Hydroxy-1-methyl-4-phenylquinazolin-2-one | 1.72 | 2.32 | 2.32 | -0.60 |
| 2205 | 2-Fluorophenylacetate | 1.76 | 2.36 | 2.37 | -0.60 |
| 2206 | 3-(Dimethylamino)benzamide | 0.95 | 0.35 | 0.27 | 0.60 |
| 2207 | Propynal | -0.01 | 0.59 | 0.62 | -0.60 |
| 2208 | Dimethoxymethane | 0.00 | 0.60 | 0.69 | -0.60 |
| 2209 | Cortisol | 1.61 | 1.01 | 0.96 | 0.60 |
| 2210 | Cromoglicic acid | 1.60 | 1.00 | 0.88 | 0.60 |
| 2211 | Pentylcarbamate | 1.35 | 0.75 | 0.70 | 0.60 |
| 2212 | 2-Pyridone | -0.58 | 0.02 | 0.00 | -0.60 |
| 2213 | Fleroxacin | -0.55 | 0.05 | 0.50 | -0.60 |
| 2214 | 2-Pyrrolidone | 0.46 | -0.14 | -0.12 | 0.60 |
| 2215 | n-Decylamine | 1.92 | 2.52 | 2.53 | -0.60 |
| 2216 | 2-Hydroxybenzylalcohol | 0.73 | 1.33 | 1.35 | -0.60 |
| 2217 | 2-Aminoquinoline | 1.87 | 2.47 | 2.42 | -0.60 |
| 2218 | 2,2',3,5',6-Pentachlorobiphenyl | 5.92 | 6.52 | 6.48 | -0.60 |
| 2219 | 4-Isothiocyanatobenzophenone | 4.88 | 4.27 | 4.23 | 0.61 |
| 2220 | 2-Methoxyphenyl N-methylcarbamate | 0.81 | 1.42 | 1.44 | -0.61 |
| 2221 | O,O-Diethyl-O-phenylphosphate | 1.34 | 1.95 | 1.99 | -0.61 |
| 2222 | 3-Fluorobenzoic acid | 2.15 | 1.54 | 1.56 | 0.61 |
| 2223 | N,N-Dimethylbenzamide | 0.62 | 1.23 | 1.28 | -0.61 |
| 2224 | Sethoxydim | 4.38 | 3.77 | 3.55 | 0.61 |
| 2225 | 2,4-Diaminophenol | -0.61 | 0.00 | 0.00 | -0.61 |
| 2226 | N-Phenylsulfamide | 0.40 | 1.01 | 1.49 | -0.61 |
| 2227 | Methyloxiran | 0.03 | 0.64 | 0.68 | -0.61 |
| 2228 | 2-Acetylphenyl N,N-dimethylcarbamate | 0.93 | 1.54 | 1.46 | -0.61 |
| 2229 | 4-Amino-2-methylbenzoic acid | 0.31 | 0.92 | 0.93 | -0.61 |
| 2230 | Semustine | 3.30 | 2.69 | 2.68 | 0.61 |
| 2231 | 1,4-Naphthoquinone | 1.78 | 1.17 | 1.18 | 0.61 |
| 2232 | Chlorprothixene | 6.03 | 5.42 | 5.27 | 0.61 |
| 2233 | 17-Methyltestosterone | 3.36 | 3.97 | 3.96 | -0.61 |
| 2234 | Pentachlorophenol | 5.12 | 4.51 | 4.50 | 0.61 |
| 2235 | Clofazimine | 7.48 | 6.86 | 6.46 | 0.62 |
| 2236 | 2,3,7,8-Tetrachlorodibenzo-p-dioxin | 6.53 | 5.91 | 5.93 | 0.62 |
| 2237 | 3-Fluorophenylacetate | 1.74 | 2.36 | 2.36 | -0.62 |
| 2238 | Pentachloroanisole | 5.45 | 4.83 | 4.83 | 0.62 |
| 2239 | Cyclohexanone | 0.81 | 1.43 | 1.39 | -0.62 |
| 2240 | Salicylic acid | 2.19 | 1.57 | 1.58 | 0.62 |
| 2241 | Zalcitabine | -1.30 | -0.68 | -0.72 | -0.62 |
| 2242 | 4-Phenoxyaniline | 2.36 | 2.98 | 2.98 | -0.62 |
| 2243 | 5-Dimethylamino-1,3,6-trimethyluracil | 0.99 | 0.37 | 0.24 | 0.62 |
| 2244 | Chloral hydrate | 0.99 | 0.37 | 0.17 | 0.62 |
| 2245 | Vinyl acetate | 0.73 | 1.35 | 1.35 | -0.62 |
| 2246 | 1-Hexen-5-one | 1.02 | 1.64 | 1.66 | -0.62 |
| 2247 | 1-(2-Methylsulfonyl-4-nitrophenylhydrazono)-1-cyanoacetic acid ethylester | 3.21 | 2.59 | 2.53 | 0.62 |
| 2248 | Phenylacetonitrile | 1.56 | 2.18 | 2.20 | -0.62 |
| 2249 | Chloroquine | 4.69 | 4.06 | 4.09 | 0.63 |
| 2250 | 2,3,5,6-Tetrachloroaniline | 4.10 | 3.47 | 3.46 | 0.63 |
| 2251 | 1-(3,4-Dichlorophenylhydrazono)-1-cyanoacetone | 4.56 | 3.93 | 3.96 | 0.63 |
| 2252 | Isobutylbenzene | 4.54 | 3.91 | 3.87 | 0.63 |
| 2253 | 3-Methoxybenzoic acid | 2.02 | 1.39 | 1.36 | 0.63 |
| 2254 | a-D-Arabinopyranose | -3.02 | -2.39 | -2.36 | -0.63 |
| 2255 | Molinate | 3.21 | 2.58 | 2.42 | 0.63 |
| 2256 | Strophantin | -1.70 | -1.07 | -0.99 | -0.63 |
| 2257 | Hypoxanthine | -1.31 | -0.68 | -0.62 | -0.63 |
| 2258 | 2-Cyanophenylacetate | 1.33 | 1.96 | 1.94 | -0.63 |
| 2259 | Hymecromone | 1.58 | 2.21 | 2.28 | -0.63 |
| 2260 | 4-Aminoacetanilide | 0.08 | 0.71 | 0.74 | -0.63 |
| 2261 | 2-Nitrobenzenesulfonamide | 0.34 | 0.97 | 0.90 | -0.63 |
| 2262 | Benzyl methyl ether | 1.35 | 1.98 | 1.99 | -0.63 |
| 2263 | 3-Aminobenzamide | 0.33 | -0.30 | -0.35 | 0.63 |
| 2264 | Flumazenil | 1.64 | 1.01 | 0.87 | 0.63 |
| 2265 | 2-Phenylimidazole | 1.88 | 1.25 | 1.18 | 0.63 |
| 2266 | Fenuron | 0.98 | 1.61 | 1.61 | -0.63 |
| 2267 | N-(3-Bromo-2-pyridyl)sulfanilamide | 1.24 | 1.87 | 1.96 | -0.63 |
| 2268 | 1-(2-Chlorophenylhydrazono)-1-cyanoacetone | 4.01 | 3.38 | 3.36 | 0.63 |
| 2269 | 2,2',3,4,5,5',6-Heptachlorobiphenyl | 6.99 | 7.62 | 7.59 | -0.63 |
| 2270 | 4-Amino-2-chlorobenzoic acid | 0.50 | 1.13 | 1.12 | -0.63 |
| 2271 | 4-Chlorobenzoic acid | 2.65 | 2.01 | 2.03 | 0.64 |
| 2272 | Ketoconazole | 4.34 | 3.70 | 3.38 | 0.64 |
| 2273 | cis-Benzaldehyde oxime | 1.75 | 1.11 | 0.93 | 0.64 |
| 2274 | 6,7-Dimethyl-1,4-naphthoquinone | 2.49 | 1.85 | 1.87 | 0.64 |
| 2275 | Bupirimate | 2.70 | 2.06 |  | 0.64 |
| 2276 | Clonidine | 1.57 | 2.21 | 2.25 | -0.64 |
| 2277 | Cyanoethylene | 0.25 | 0.89 | 0.91 | -0.64 |
| 2278 | 1-(2-NO2-5-CF3-phenylhydrazono)-1-cyanoacetic acid methylester | 3.44 | 4.08 | 4.14 | -0.64 |
| 2279 | 5-Chloro-1-phenyltetrazole | 1.48 | 2.12 |  | -0.64 |
| 2280 | Etofylline | -0.49 | -1.13 | -1.25 | 0.64 |
| 2281 | 3,4-Dimethoxyamphetamine | 1.00 | 1.64 | 1.66 | -0.64 |
| 2282 | 4-Methyl-5-methoxy-8-quinolinol | 2.75 | 2.11 | 2.13 | 0.64 |
| 2283 | Ribavirin | -2.60 | -3.24 | -3.26 | 0.64 |
| 2284 | Fentanyl | 3.89 | 4.53 | 4.52 | -0.64 |
| 2285 | 2,4,5-Trichlorotoluene | 4.78 | 4.14 | 4.12 | 0.64 |
| 2286 | 4,4'-Dibromobiphenyl | 5.72 | 5.07 | 5.09 | 0.65 |
| 2287 | Carvedilol | 4.14 | 3.49 | 3.37 | 0.65 |
| 2288 | 5-Bromosalicylic acid | 2.87 | 2.22 | 2.28 | 0.65 |
| 2289 | Bromoperidol | 3.65 | 4.30 | 4.24 | -0.65 |
| 2290 | Levopropoxyphene | 4.40 | 5.05 | 5.03 | -0.65 |
| 2291 | 3,5-Dichloropyridine | 2.56 | 1.91 | 1.89 | 0.65 |
| 2292 | Indometacin | 3.10 | 3.75 | 3.75 | -0.65 |
| 2293 | Tetrachloroveratrole | 4.86 | 4.21 | 4.19 | 0.65 |
| 2294 | 8-Nitroquinoline-1-oxide | 0.04 | 0.69 | 0.75 | -0.65 |
| 2295 | 2-Chlorophenylacetate | 2.18 | 2.83 | 2.83 | -0.65 |
| 2296 | 2,4-Dichlorotoluene | 4.24 | 3.59 | 3.57 | 0.65 |
| 2297 | 2-Nitrophenylacetate | 1.55 | 2.20 | 2.22 | -0.65 |
| 2298 | 1H-Indazole | 1.82 | 2.47 | 2.46 | -0.65 |
| 2299 | Decylbenzene | 6.94 | 6.28 | 6.24 | 0.66 |
| 2300 | 3-Iodobenzoic acid | 3.13 | 2.47 | 2.48 | 0.66 |
| 2301 | 1-(4-Cl-2-CH3-phenylhydrazono)-1-cyanoacetic acid methylester | 4.28 | 3.62 | 3.59 | 0.66 |
| 2302 | o-Toluic acid | 2.46 | 1.80 | 1.81 | 0.66 |
| 2303 | Quinoline-4-one | 0.58 | 1.24 | 1.31 | -0.66 |
| 2304 | Ampicillin | -2.17 | -1.51 | -1.49 | -0.66 |
| 2305 | 2-Butanone | 0.29 | 0.95 | 0.98 | -0.66 |
| 2306 | 9H-Fluoren-9-one | 3.58 | 2.92 | 2.98 | 0.66 |
| 2307 | 2,2',3,3',4,4',5-Heptachlorobiphenyl | 6.96 | 7.62 | 7.57 | -0.66 |
| 2308 | 3-Hydroxyphenylacetate | 1.23 | 1.89 | 1.85 | -0.66 |
| 2309 | 8-Trifluoromethylquinoline | 2.50 | 3.16 | 3.18 | -0.66 |
| 2310 | 3,3',4,4'-Tetrachlorobiphenyl | 6.63 | 5.97 | 6.02 | 0.66 |
| 2311 | Fenvalerate | 6.20 | 6.86 | 6.78 | -0.66 |
| 2312 | 2,6-Diphenylpyridine | 4.82 | 4.15 | 3.97 | 0.67 |
| 2313 | 3-Chlorobenzoic acid | 2.68 | 2.01 | 2.04 | 0.67 |
| 2314 | 4-Phenylbutyronitrile | 2.21 | 2.88 | 2.93 | -0.67 |
| 2315 | 1-(3,5-Dichlorophenylhydrazono)-1-cyanoacetic acid methylester | 4.50 | 3.83 | 3.80 | 0.67 |
| 2316 | Altretamine | 2.73 | 2.06 | 1.59 | 0.67 |
| 2317 | 4-Methoxyphenylacetate | 1.54 | 2.21 | 2.23 | -0.67 |
| 2318 | Ethyl 4-pyridinecarboxylate | 1.43 | 0.76 | 0.78 | 0.67 |
| 2319 | 2,3-Butyleneglycol | -0.92 | -0.25 | -0.19 | -0.67 |
| 2320 | Mephenesin | 1.41 | 0.74 | 0.74 | 0.67 |
| 2321 | Benzocaine | 1.89 | 1.22 | 1.23 | 0.67 |
| 2322 | Enantholactam | 0.24 | 0.91 | 0.96 | -0.67 |
| 2323 | 4-(2-Pyridyl)pentamide | -0.01 | 0.66 | 0.69 | -0.67 |
| 2324 | Dibenzo-p-dioxin | 4.38 | 3.71 | 3.74 | 0.67 |
| 2325 | 1,3-Dimethyluracil | 0.00 | -0.67 | -0.70 | 0.67 |
| 2326 | 4-Amino-2-ethoxybenzoic acid | 0.16 | 0.83 | 0.84 | -0.67 |
| 2327 | 2,2',4,4',5,6'-Hexachlorodiphenyl ether | 6.49 | 7.16 | 7.14 | -0.67 |
| 2328 | p-Benzidine | 1.34 | 2.01 | 1.95 | -0.67 |
| 2329 | 1,2,4-Triiodobenzene | 5.85 | 5.18 | 5.25 | 0.67 |
| 2330 | 1,3,5-Triiodobenzene | 5.85 | 5.18 | 5.15 | 0.67 |
| 2331 | 2-Decanone | 3.73 | 3.05 | 3.04 | 0.68 |
| 2332 | Hydroxyzine | 3.55 | 2.87 | 2.79 | 0.68 |
| 2333 | Benzalcyanoacetamide | 1.50 | 0.82 | 0.81 | 0.68 |
| 2334 | Spironolactone | 2.78 | 3.46 | 3.80 | -0.68 |
| 2335 | Amylamine | 1.45 | 0.77 | 0.78 | 0.68 |
| 2336 | Epivir | -0.93 | -0.25 |  | -0.68 |
| 2337 | 8-Azaguanine | -0.71 | -0.03 | 0.00 | -0.68 |
| 2338 | Methylacetamide | -1.05 | -0.37 | -0.34 | -0.68 |
| 2339 | Acetone oxime | 0.20 | 0.88 | 1.07 | -0.68 |
| 2340 | 1-Naphthoic acid | 3.10 | 2.42 | 2.38 | 0.68 |
| 2341 | 1,2,3-Triiodobenzene | 5.86 | 5.18 | 5.12 | 0.68 |
| 2342 | 2,2',4,4',5-Pentachlorobiphenyl | 7.21 | 6.52 | 6.49 | 0.69 |
| 2343 | 1-Hexanol | 2.03 | 1.34 | 1.35 | 0.69 |
| 2344 | 2,2',3,4,4',5,6,6'-Octachlorobiphenyl | 7.48 | 8.17 | 8.16 | -0.69 |
| 2345 | Oxamniquine | 2.24 | 1.55 | 1.58 | 0.69 |
| 2346 | 5-Allyl-5-ethylbarbituric acid | 0.95 | 0.26 | 0.23 | 0.69 |
| 2347 | 2,4,6-Trinitrophenol | 1.33 | 2.02 | 2.04 | -0.69 |
| 2348 | Fluchloralin | 4.63 | 3.94 | 3.83 | 0.69 |
| 2349 | Pebulate | 3.84 | 3.15 | 2.96 | 0.69 |
| 2350 | Vernolate | 3.84 | 3.15 | 3.09 | 0.69 |
| 2351 | 3-Acetamido-4-chlorophenoxyacetic acid | 0.67 | 1.36 | 1.37 | -0.69 |
| 2352 | cis-1,3-Dichloropropene | 1.76 | 2.45 | 2.53 | -0.69 |
| 2353 | 2,7-Dichlorodibenzofuran | 5.41 | 4.72 | 4.73 | 0.69 |
| 2354 | Retinol | 5.68 | 4.98 | 4.87 | 0.70 |
| 2355 | Remoxipride | 2.13 | 2.83 | 2.87 | -0.70 |
| 2356 | 2,2',3,3',4,5',6-Heptachlorobiphenyl | 6.92 | 7.62 | 7.57 | -0.70 |
| 2357 | 2,2',3,4,4',5',6-Heptachlorobiphenyl | 6.92 | 7.62 | 7.57 | -0.70 |
| 2358 | 2,2',3,4,4',5,6'-Heptachlorobiphenyl | 6.92 | 7.62 | 7.58 | -0.70 |
| 2359 | Benzo[b]triphenylene | 6.17 | 6.87 | 6.99 | -0.70 |
| 2360 | 2,6-Dichlorotoluene | 4.29 | 3.59 | 3.60 | 0.70 |
| 2361 | Butralin | 4.54 | 3.84 | 3.69 | 0.70 |
| 2362 | Carfecillin | 2.96 | 3.66 | 3.57 | -0.70 |
| 2363 | 2,4,4',5-Tetrachlorobiphenyl | 6.67 | 5.97 | 5.92 | 0.70 |
| 2364 | 9-Acetylcarbazole | 3.45 | 2.75 | 2.68 | 0.70 |
| 2365 | 2,3,7,8-Tetrachlorodibenzofuran | 6.53 | 5.82 | 5.84 | 0.71 |
| 2366 | 7,12-Dimethylbenz[a]anthracene | 5.80 | 6.51 | 6.61 | -0.71 |
| 2367 | 1-Bromopentane | 3.37 | 2.66 | 2.68 | 0.71 |
| 2368 | 4,4'-Dichlorobiphenyl | 5.58 | 4.87 | 4.81 | 0.71 |
| 2369 | alpha-Hydroxybenzylpenicillin | 1.40 | 0.69 | 0.64 | 0.71 |
| 2370 | Procymidone | 3.00 | 2.29 | 2.29 | 0.71 |
| 2371 | Cytosine | -1.73 | -1.02 | -0.93 | -0.71 |
| 2372 | 7-Dimethylaminoquinoline | 2.71 | 2.00 | 2.04 | 0.71 |
| 2373 | 3-Aminophenol | 0.17 | 0.88 | 0.91 | -0.71 |
| 2374 | Morpholine | -1.08 | -0.37 | -0.32 | -0.71 |
| 2375 | Benzylisothiocyanate | 2.83 | 3.54 | 3.64 | -0.71 |
| 2376 | Butylate | 4.15 | 3.44 | 3.27 | 0.71 |
| 2377 | Ketamine | 2.18 | 2.89 | 2.93 | -0.71 |
| 2378 | 4-Nitrophenylacetate | 1.49 | 2.20 | 2.22 | -0.71 |
| 2379 | 2,3-Dichlorodibenzofuran | 5.43 | 4.72 | 4.69 | 0.71 |
| 2380 | Tetrachlorocatechol | 4.29 | 3.57 | 3.54 | 0.72 |
| 2381 | Diisobutyl phthalate | 4.11 | 3.39 | 3.38 | 0.72 |
| 2382 | 3,5-Dinitrophenol | 2.32 | 1.60 | 1.58 | 0.72 |
| 2383 | Ergonovine | 1.67 | 0.95 | 0.90 | 0.72 |
| 2384 | Methyl levulinate | -0.13 | 0.59 | 0.59 | -0.72 |
| 2385 | Dimethylacetamide | -0.77 | -0.05 | -0.08 | -0.72 |
| 2386 | Butyronitrile | 0.53 | 1.25 | 1.23 | -0.72 |
| 2387 | Citric acid | -1.72 | -1.00 | -1.05 | -0.72 |
| 2388 | Ethyl 4-hydroxybenzoate | 2.43 | 1.71 | 1.71 | 0.72 |
| 2389 | Benalaxyl | 3.40 | 4.12 | 4.18 | -0.72 |
| 2390 | 3,6-Dichlorodibenzofuran | 5.44 | 4.72 | 4.73 | 0.72 |
| 2391 | 1-Chlorobutane | 2.64 | 1.91 | 1.88 | 0.73 |
| 2392 | 3,4-Dihydro-2H-1-benzopyran-2-one | 1.67 | 2.40 | 2.34 | -0.73 |
| 2393 | 3,4,5-Trichlorocatechol | 3.75 | 3.02 | 2.99 | 0.73 |
| 2394 | Nitroethylene | 0.01 | 0.74 | 0.83 | -0.73 |
| 2395 | Thifensulfuron-methyl | 1.23 | 0.50 | 0.17 | 0.73 |
| 2396 | Barbital | 0.65 | -0.08 | -0.06 | 0.73 |
| 2397 | Probarbital | 0.97 | 0.24 | 0.20 | 0.73 |
| 2398 | 2,2',3,4,4',5,5'-Heptachlorobiphenyl | 6.89 | 7.62 | 7.57 | -0.73 |
| 2399 | 1,3,5-Trinitrobenzene | 1.18 | 1.91 | 1.89 | -0.73 |
| 2400 | 2-Bromophenylacetate | 2.20 | 2.93 | 2.94 | -0.73 |
| 2401 | Penconazole | 3.40 | 4.14 | 4.17 | -0.74 |
| 2402 | Phenolphthalol | 3.27 | 4.01 | 4.04 | -0.74 |
| 2403 | 2,2',3,3',4,4',5,6'-Octachlorobiphenyl | 7.43 | 8.17 | 8.11 | -0.74 |
| 2404 | 2,2',3,3',4,5,5',6-Octachlorobiphenyl | 7.43 | 8.17 | 8.13 | -0.74 |
| 2405 | Erythromycin | 2.54 | 1.80 | 1.63 | 0.74 |
| 2406 | 4-Amino-6-methyl-3-(methylthio)-1,2,4-triazin-5-one | -0.16 | 0.58 | 0.66 | -0.74 |
| 2407 | Propanenitrile | 0.16 | 0.90 | 0.92 | -0.74 |
| 2408 | 4-Methyl-N-nitrosopiperazine | 0.20 | -0.54 | -0.59 | 0.74 |
| 2409 | 2-Iodophenylacetate | 2.55 | 3.29 | 3.31 | -0.74 |
| 2410 | 4-Dimethylamino-beta-nitrostyrene | 2.67 | 1.93 | 1.81 | 0.74 |
| 2411 | 2,2',3,3',4,4',5,5',6-Nonachlorodiphenyl ether | 8.07 | 8.81 | 8.79 | -0.74 |
| 2412 | 1-Pentylbenzotriazole | 3.22 | 2.47 | 2.33 | 0.75 |
| 2413 | 1-(3,5-Dichlorophenylhydrazono)-1-cyanoacetone | 4.68 | 3.93 | 3.88 | 0.75 |
| 2414 | 1H-Benzotriazole | 1.34 | 2.09 | 2.47 | -0.75 |
| 2415 | 1-(2,4-Dinitrophenylhydrazono)-1-cyanoacetic acid ethylester | 4.14 | 3.39 | 3.38 | 0.75 |
| 2416 | 8-Nitroquinoline | 1.40 | 2.15 | 2.15 | -0.75 |
| 2417 | Hexylcarbamate | 1.85 | 1.10 | 1.11 | 0.75 |
| 2418 | 3-Allyloxy-4-aminobenzoic acid | 0.42 | 1.17 | 1.17 | -0.75 |
| 2419 | (L)-Histidine | -2.52 | -3.27 | -3.41 | 0.75 |
| 2420 | epsilon-Caprolactam | -0.19 | 0.56 | 0.55 | -0.75 |
| 2421 | Clozapine | 3.90 | 3.15 | 3.06 | 0.75 |
| 2422 | 1-(4-Chlorophenylhydrazono)-1-cyanoacetone | 4.13 | 3.38 | 3.42 | 0.75 |
| 2423 | 3,5-Dichlorophenol | 3.61 | 2.86 | 2.84 | 0.75 |
| 2424 | 4-Bromobenzoic acid | 2.86 | 2.11 | 2.12 | 0.75 |
| 2425 | Phenoxymethylpenicillin | 2.09 | 1.33 | 1.28 | 0.76 |
| 2426 | Tacrine | 3.32 | 2.56 | 2.55 | 0.76 |
| 2427 | 1-Heptene | 3.99 | 3.23 | 3.27 | 0.76 |
| 2428 | 4-(Methylthio)benzoic acid | 2.74 | 1.98 | 1.92 | 0.76 |
| 2429 | Fluoxetine | 3.82 | 4.58 | 4.64 | -0.76 |
| 2430 | Triethanolamine | -1.59 | -2.35 | -2.30 | 0.76 |
| 2431 | 2-Acetaminpyridine | 0.61 | 1.37 | 1.38 | -0.76 |
| 2432 | 3-Bromobenzoic acid | 2.87 | 2.11 | 2.08 | 0.76 |
| 2433 | 1-(2-Cl-5-CF3-phenylhydrazono)-1-cyanoacetone | 5.08 | 4.31 | 4.22 | 0.77 |
| 2434 | Aspartic acid | -3.70 | -2.93 | -2.80 | -0.77 |
| 2435 | 9-Pentyladenine | 1.79 | 1.02 | 0.95 | 0.77 |
| 2436 | N-Maleoyl-3-aminopropionic acid ethylester | 0.41 | -0.36 | -0.28 | 0.77 |
| 2437 | Dexsotalol | -0.47 | 0.30 | 0.48 | -0.77 |
| 2438 | 2,4,5-Trichloroaniline | 3.69 | 2.92 | 2.90 | 0.77 |
| 2439 | 4-Amino-2-iodobenzoic acid | 0.82 | 1.59 | 1.62 | -0.77 |
| 2440 | 2,6-Dinitrophenol | 1.33 | 2.10 | 2.12 | -0.77 |
| 2441 | 1-(2,4,5-Trichlorophenylhydrazono)-1-cyanoacetic acid methylester | 5.15 | 4.38 | 4.33 | 0.77 |
| 2442 | 2,2',3,3',5,5',6-Heptachlorobiphenyl | 6.85 | 7.62 | 7.59 | -0.77 |
| 2443 | Tamoxifen | 7.10 | 6.32 | 6.27 | 0.78 |
| 2444 | Diphenyl 2-ethylhexyl phosphate | 5.73 | 4.95 | 4.95 | 0.78 |
| 2445 | Isophthalamide | -0.21 | -0.99 | -1.08 | 0.78 |
| 2446 | Rufinamide | 0.90 | 0.12 | 0.01 | 0.78 |
| 2447 | Triphenylamine | 5.74 | 4.96 | 4.97 | 0.78 |
| 2448 | Bensulfuron-methyl | 2.45 | 1.67 | 1.59 | 0.78 |
| 2449 | 2,2',3,4',5,5',6-Heptachlorobiphenyl | 6.84 | 7.62 | 7.59 | -0.78 |
| 2450 | Hydroquinone | 0.59 | 1.37 | 1.38 | -0.78 |
| 2451 | Saccharose | -3.70 | -4.48 | -4.66 | 0.78 |
| 2452 | Hexyl bromide | 3.80 | 3.01 | 3.01 | 0.79 |
| 2453 | 2-t-Butylphenoxyacetic acid | 3.33 | 2.54 | 2.50 | 0.79 |
| 2454 | 4-(Dimethylamino)benzamide | 1.14 | 0.35 | 0.35 | 0.79 |
| 2455 | Aldrin | 6.50 | 5.71 | 5.57 | 0.79 |
| 2456 | Aprobarbital | 1.37 | 0.58 | 0.52 | 0.79 |
| 2457 | Di-n-butylether | 3.21 | 2.42 | 2.34 | 0.79 |
| 2458 | 2,5-Dimethyl-4-hydroxyacetanilide | 1.09 | 1.88 | 1.89 | -0.79 |
| 2459 | Coumarin | 1.39 | 2.18 | 2.26 | -0.79 |
| 2460 | 1-Chlorodibenzo-p-dioxin | 5.05 | 4.26 | 4.17 | 0.79 |
| 2461 | Ethyl isothiocyanate | 1.47 | 2.26 | 2.28 | -0.79 |
| 2462 | 4-Amino-2-butoxybenzoic acid | 1.24 | 2.03 | 2.08 | -0.79 |
| 2463 | Phenylacetate | 1.49 | 2.28 | 2.31 | -0.79 |
| 2464 | 2,3,4,5-Tetrachloroaniline | 4.27 | 3.47 | 3.46 | 0.80 |
| 2465 | Arbutin | -1.35 | -0.55 | -0.59 | -0.80 |
| 2466 | Tolbutamide | 2.34 | 1.54 | 1.53 | 0.80 |
| 2467 | Octylbenzene | 6.30 | 5.50 | 5.50 | 0.80 |
| 2468 | Cefaclor | -1.79 | -0.99 | -0.70 | -0.80 |
| 2469 | Methylphenylsulfone | 0.47 | 1.27 | 1.37 | -0.80 |
| 2470 | Levofloxacin | -0.28 | 0.52 | 0.67 | -0.80 |
| 2471 | Benzotrichloride | 2.92 | 3.72 | 3.77 | -0.80 |
| 2472 | Hexylbenzene | 5.52 | 4.72 | 4.71 | 0.80 |
| 2473 | Quinoclamine | 2.12 | 1.31 | 1.13 | 0.81 |
| 2474 | Chlorothalonil | 2.90 | 3.71 | 3.81 | -0.81 |
| 2475 | Sulfadimidine | 0.27 | 1.08 | 1.08 | -0.81 |
| 2476 | 4-Amino-3,5,6-trichloro-2-pyridinecarboxlic acid | 0.30 | 1.11 | 1.09 | -0.81 |
| 2477 | 2,2',3,3',4,5,6,6'-Octachlorobiphenyl | 7.35 | 8.17 | 8.13 | -0.82 |
| 2478 | Tolazoline | 2.65 | 1.83 | 1.86 | 0.82 |
| 2479 | Phloroglucinol | 0.16 | 0.98 | 1.04 | -0.82 |
| 2480 | 2,3-Dimethyl-4-hydroxyacetanilide | 1.06 | 1.88 | 1.87 | -0.82 |
| 2481 | Tetrahydrocannabinol | 6.97 | 6.14 | 6.07 | 0.83 |
| 2482 | Benzil | 3.38 | 2.55 | 2.43 | 0.83 |
| 2483 | Digoxin | 1.26 | 2.09 | 2.14 | -0.83 |
| 2484 | 4-Amino-2-pentoxybenzoic acid | 1.55 | 2.38 | 2.40 | -0.83 |
| 2485 | 2-Phenoxybenzoic acid | 2.84 | 3.67 | 3.68 | -0.83 |
| 2486 | Lormetazepam | 2.38 | 3.21 | 3.47 | -0.83 |
| 2487 | 2-Acetoxyanisole | 1.38 | 2.21 | 2.21 | -0.83 |
| 2488 | 9-(1-(hydroxymethyl)butyl)adenine | 0.66 | -0.17 | -0.29 | 0.83 |
| 2489 | Octachlorostyrene | 6.29 | 7.12 | 7.13 | -0.83 |
| 2490 | 2-Amino-4-picoline | 0.56 | 1.39 | 1.44 | -0.83 |
| 2491 | Hydrocinnamonitrile | 1.70 | 2.53 | 2.53 | -0.83 |
| 2492 | 4-Benzylphenylisothiocyanate | 4.40 | 5.23 | 5.31 | -0.83 |
| 2493 | Propiconazole | 3.50 | 4.34 | 5.15 | -0.84 |
| 2494 | Acetone | -0.24 | 0.60 | 0.59 | -0.84 |
| 2495 | Hexobarbital | 1.49 | 0.65 | 0.68 | 0.84 |
| 2496 | 4-Aminophenol | 0.04 | 0.88 | 0.89 | -0.84 |
| 2497 | 1-(3,4,5-Trichlorophenylhydrazono)-1-cyanoacetic acid methylester | 5.22 | 4.38 | 4.39 | 0.84 |
| 2498 | 2,2',3,4',5,6,6'-Heptachlorobiphenyl | 6.78 | 7.62 | 7.57 | -0.84 |
| 2499 | 1,2,4,6,7,9-Hexachlorodibenzo-p-dioxin | 7.85 | 7.01 | 6.98 | 0.84 |
| 2500 | 2,2',3,3',4,4',5,6,6'-Nonachlorobiphenyl | 7.88 | 8.72 | 8.68 | -0.84 |
| 2501 | Mesoridazine | 3.05 | 3.90 | 4.16 | -0.85 |
| 2502 | 5-Nitrosalicylic acid | 2.34 | 1.49 | 1.48 | 0.85 |
| 2503 | 9-Carboxy-9,10-dihydroanthracene | 2.67 | 3.52 | 3.47 | -0.85 |
| 2504 | 4-Amino-3-methylthio-1,2,4-triazin-5-one | 0.38 | -0.47 | -0.62 | 0.85 |
| 2505 | Chlorfenvinphos | 3.10 | 3.95 | 4.21 | -0.85 |
| 2506 | Codeine | 1.19 | 2.05 | 2.02 | -0.86 |
| 2507 | Aspirin | 1.23 | 2.09 | 2.16 | -0.86 |
| 2508 | Dialifor | 4.69 | 3.83 | 3.41 | 0.86 |
| 2509 | Paracetamol | 0.34 | 1.20 | 1.21 | -0.86 |
| 2510 | 1-Fluoropentane | 2.33 | 1.47 | 1.37 | 0.86 |
| 2511 | Amlodipine | 3.74 | 2.88 | 2.62 | 0.86 |
| 2512 | Vidarabine | -1.05 | -1.91 | -2.13 | 0.86 |
| 2513 | Uric acid | -1.11 | -0.24 | -0.14 | -0.87 |
| 2514 | Chlorpropamide | 2.27 | 1.40 | 1.44 | 0.87 |
| 2515 | Ornithine | -4.41 | -3.54 | -3.43 | -0.87 |
| 2516 | 3-Aminobenzenesulfonamide | -1.20 | -0.33 | -0.25 | -0.87 |
| 2517 | Pipracil | 0.50 | -0.37 | -0.43 | 0.87 |
| 2518 | Pyrazole | 0.13 | 1.00 | 1.03 | -0.87 |
| 2519 | Ninhydrin | 0.65 | -0.22 | -0.60 | 0.87 |
| 2520 | 2-Phenoxyanisole | 2.92 | 3.79 | 3.77 | -0.87 |
| 2521 | Sulfisomidine | -0.30 | 0.57 | 0.98 | -0.87 |
| 2522 | 1,3,5,7-Tetrachloronaphthalene | 6.19 | 5.31 | 5.33 | 0.88 |
| 2523 | p-Isopropylphenyl diphenyl phosphate | 5.70 | 4.82 | 4.56 | 0.88 |
| 2524 | Estrone | 3.13 | 4.01 | 4.03 | -0.88 |
| 2525 | Uridine | -1.71 | -2.59 | -2.58 | 0.88 |
| 2526 | N,N-Diethylbenzamide | 1.25 | 2.13 | 2.15 | -0.88 |
| 2527 | Guanosine | -1.89 | -2.77 | -2.84 | 0.88 |
| 2528 | 2-Methyl-4-hydroxyacetanilide | 0.66 | 1.54 | 1.52 | -0.88 |
| 2529 | Enilconazole | 3.82 | 2.94 | 2.84 | 0.88 |
| 2530 | 4-Amino-2-isopentoxybenzoic acid | 1.47 | 2.35 | 2.31 | -0.88 |
| 2531 | 6-Chloro-1-methyl-4-phenylquinazolin-2-one | 2.38 | 3.26 | 3.39 | -0.88 |
| 2532 | alpha,alpha-Diphenylpropionic acid | 2.69 | 3.57 | 3.65 | -0.88 |
| 2533 | 1,2,3-Tribromobenzene | 4.98 | 4.10 | 4.12 | 0.88 |
| 2534 | Tri-n-butyl phosphate | 4.01 | 3.12 | 3.35 | 0.89 |
| 2535 | Isophthalic acid | 1.66 | 0.77 | 0.75 | 0.89 |
| 2536 | Clindamycin | 2.16 | 1.27 |  | 0.89 |
| 2537 | Fusaric acid | 0.68 | -0.21 | -0.51 | 0.89 |
| 2538 | 4-Amino-2-methoxybenzoic acid | -0.38 | 0.51 | 0.52 | -0.89 |
| 2539 | Benomyl | 2.12 | 1.23 | 1.14 | 0.89 |
| 2540 | 3-Aminobenzylalcohol | -0.55 | 0.34 | 0.36 | -0.89 |
| 2541 | Acetonitrile | -0.34 | 0.55 | 0.64 | -0.89 |
| 2542 | 2,2',3,3',4,5',6'-Heptachlorobiphenyl | 6.73 | 7.62 | 7.60 | -0.89 |
| 2543 | Fenpropathrin | 6.00 | 5.10 | 5.00 | 0.90 |
| 2544 | Iopromide | -2.05 | -1.15 | -1.03 | -0.90 |
| 2545 | Nebivolol | 4.04 | 3.14 | 3.05 | 0.90 |
| 2546 | alpha-Pinene | 4.44 | 3.54 | 3.53 | 0.90 |
| 2547 | Didanosine | -1.24 | -0.34 | -0.22 | -0.90 |
| 2548 | Nevirapine | 2.41 | 1.51 | 1.43 | 0.90 |
| 2549 | 4-Sulfamidobenzoic acid propylester | 1.75 | 0.85 | 0.75 | 0.90 |
| 2550 | 7-Chloro-1-methyl-4-phenylquinazolin-2-one | 2.36 | 3.26 | 3.37 | -0.90 |
| 2551 | Enoxacin | 2.30 | 1.39 | 1.21 | 0.91 |
| 2552 | Paspertin | 2.62 | 1.71 | 1.70 | 0.91 |
| 2553 | Mirex | 5.28 | 6.19 | 7.22 | -0.91 |
| 2554 | Dibutyl phenyl phosphate | 4.27 | 3.35 | 3.47 | 0.92 |
| 2555 | 1-Methyl-4-phenylquinazolin-2-one | 1.79 | 2.71 | 2.83 | -0.92 |
| 2556 | 4-Isothiocyanatodiphenylsulfoxide | 4.40 | 3.48 | 3.18 | 0.92 |
| 2557 | 6-Fluoro-1-methyl-4-phenylquinazolin-2-one | 1.87 | 2.79 | 2.84 | -0.92 |
| 2558 | Fenoxycarb | 4.30 | 3.38 | 3.30 | 0.92 |
| 2559 | 2,3,5-Trimethyl-4-hydroxyacetanilide | 1.30 | 2.22 | 2.20 | -0.92 |
| 2560 | Carazolol | 3.73 | 2.80 | 2.77 | 0.93 |
| 2561 | Marcoumar | 3.62 | 4.55 | 4.60 | -0.93 |
| 2562 | Buquinolate | 2.18 | 3.11 | 3.23 | -0.93 |
| 2563 | 2-Nitroresorcinol | 1.36 | 2.29 | 2.33 | -0.93 |
| 2564 | Emodepside | 4.90 | 5.83 | 6.21 | -0.93 |
| 2565 | 2,8-Dichlorodibenzofuran | 5.65 | 4.72 | 4.76 | 0.93 |
| 2566 | 1-(4-CF3SO2-phenylhydrazono)-1-cyanoacetic acid methylester | 4.22 | 3.28 | 3.13 | 0.94 |
| 2567 | N-Methyl-N-benzylaniline | 4.22 | 3.28 | 3.18 | 0.94 |
| 2568 | n-Hexylamine | 2.06 | 1.12 | 1.16 | 0.94 |
| 2569 | Methylphenobarbital | 1.84 | 0.90 | 0.84 | 0.94 |
| 2570 | 5-Butyl-N-methylbarbituric acid | 1.10 | 0.16 | 0.19 | 0.94 |
| 2571 | 4-Hexylpyridine | 3.84 | 2.90 | 2.90 | 0.94 |
| 2572 | 2,2',3,3',4,4',6-Heptachlorobiphenyl | 6.68 | 7.62 | 7.58 | -0.94 |
| 2573 | Di-n-hexyl phthalate | 5.80 | 4.85 | 4.89 | 0.95 |
| 2574 | 1,3,7-Trichlorodibenzo-p-dioxin | 6.31 | 5.36 | 5.36 | 0.95 |
| 2575 | Caprylene | 4.57 | 3.62 | 3.63 | 0.95 |
| 2576 | 4-Butoxybenzamide | 2.48 | 1.53 | 1.56 | 0.95 |
| 2577 | Acetylsulfanilamide | -0.96 | -0.01 | -0.01 | -0.95 |
| 2578 | 4-Carboxyphenylisothiocyanate | 3.52 | 2.57 | 2.54 | 0.95 |
| 2579 | Flufenamic acid | 5.25 | 4.30 | 4.27 | 0.95 |
| 2580 | 2,7-Dichlorodibenzo-p-dioxin | 5.77 | 4.81 | 4.72 | 0.96 |
| 2581 | 2,8-Dichlorodibenzo-p-dioxin | 5.77 | 4.81 | 4.80 | 0.96 |
| 2582 | Quinclorac | 3.60 | 2.64 | 2.65 | 0.96 |
| 2583 | 2,2',3,3',4,4',6,6'-Octachlorobiphenyl | 7.21 | 8.17 | 8.13 | -0.96 |
| 2584 | 2,2',3,3',4,5,5',6'-Octachlorobiphenyl | 7.21 | 8.17 | 8.11 | -0.96 |
| 2585 | 2,2',3,4,4',5,5',6-Octachlorobiphenyl | 7.21 | 8.17 | 8.13 | -0.96 |
| 2586 | Diflunisal | 4.32 | 3.35 | 3.34 | 0.97 |
| 2587 | Morphine | 0.76 | 1.73 | 1.72 | -0.97 |
| 2588 | 2,2',3,3',4,6,6'-Heptachlorobiphenyl | 6.65 | 7.62 | 7.67 | -0.97 |
| 2589 | 2,2',3,4,4',6,6'-Heptachlorobiphenyl | 6.65 | 7.62 | 7.57 | -0.97 |
| 2590 | 3,5-Diiodosalicylic acid | 4.56 | 3.59 | 3.57 | 0.97 |
| 2591 | 1,2,4-Trichlorodibenzo-p-dioxin | 6.34 | 5.36 | 5.27 | 0.98 |
| 2592 | Benorylate | 2.15 | 3.13 | 3.11 | -0.98 |
| 2593 | Preforan | 3.65 | 4.63 | 4.62 | -0.98 |
| 2594 | 4-Amino-2-propoxybenzoic acid | 0.70 | 1.68 | 1.73 | -0.98 |
| 2595 | Propylparaben | 3.04 | 2.06 | 2.07 | 0.98 |
| 2596 | Meprobamate | 0.70 | -0.28 | -0.44 | 0.98 |
| 2597 | Crotoxyphos | 3.30 | 2.32 | 2.18 | 0.98 |
| 2598 | 2,3,6-Trimethyl-4-hydroxyacetanilide | 1.24 | 2.22 | 2.23 | -0.98 |
| 2599 | 2,3-Dichlorodibenzo-p-dioxin | 5.80 | 4.81 | 4.81 | 0.99 |
| 2600 | N-Phenylanthranilic acid | 4.36 | 3.37 | 3.40 | 0.99 |
| 2601 | Memantine | 3.28 | 2.29 | 2.24 | 0.99 |
| 2602 | Formamidoxime | -1.64 | -0.65 | -0.39 | -0.99 |
| 2603 | 2,2',3,3',4,4',5,5',6,6'-Decachlorobiphenyl | 8.27 | 9.27 | 9.32 | -1.00 |
| 2604 | Benefin | 5.29 | 4.29 | 4.20 | 1.00 |
| 2605 | 4-Nitromethylhippuric acid | 0.98 | -0.02 | -0.15 | 1.00 |
| 2606 | 6-Azauridine | -2.14 | -3.14 | -3.30 | 1.00 |
| 2607 | Heptyl bromide | 4.36 | 3.36 | 3.35 | 1.00 |
| 2608 | Bendiocarb | 1.70 | 2.70 | 3.28 | -1.00 |
| 2609 | 1,2,3,5,7-Pentachloronaphthalene | 6.87 | 5.86 | 5.82 | 1.01 |
| 2610 | 5-Azauracil | -1.87 | -0.86 | -0.77 | -1.01 |
| 2611 | Cefotaxime | -1.36 | -0.34 | -0.07 | -1.02 |
| 2612 | Mevinphos | 1.20 | 0.18 | 0.11 | 1.02 |
| 2613 | Probenecid | 3.21 | 2.18 | 2.00 | 1.03 |
| 2614 | 2-Ethylhexylamine | 2.82 | 1.79 | 1.80 | 1.03 |
| 2615 | Doxycycline | 0.42 | -0.61 | -0.93 | 1.03 |
| 2616 | 5-Ethyl-6-azauracil | 0.22 | 1.25 | 1.29 | -1.03 |
| 2617 | 1-Heptanol | 2.72 | 1.69 | 1.73 | 1.03 |
| 2618 | Cinnolin-4-one | 0.82 | 1.86 | 2.09 | -1.04 |
| 2619 | 2,4,6-Tribromoresorcinol | 4.37 | 3.32 | 3.31 | 1.05 |
| 2620 | Trifluralin | 5.34 | 4.29 | 4.29 | 1.05 |
| 2621 | Glipizide | 1.91 | 0.86 | 0.79 | 1.05 |
| 2622 | p-Nonylphenyl diphenyl phosphate | 5.90 | 6.95 | 7.33 | -1.05 |
| 2623 | 6-Acetylmorphine | 1.55 | 2.61 | 2.74 | -1.06 |
| 2624 | Pentafluorophenol | 3.23 | 2.16 | 2.06 | 1.07 |
| 2625 | Pentazocine | 3.31 | 4.38 | 4.41 | -1.07 |
| 2626 | 2,4,6-Trichloropurine | 3.90 | 2.83 |  | 1.07 |
| 2627 | Phenylglycerol | 0.70 | -0.37 | -0.34 | 1.07 |
| 2628 | n-Octylamine | 2.90 | 1.82 | 1.82 | 1.08 |
| 2629 | Triflumizole | 1.40 | 2.48 | 2.73 | -1.08 |
| 2630 | 2-Methylbenzaldoxime | 2.53 | 1.45 | 1.14 | 1.08 |
| 2631 | 2,6-Dimethyl-4-hydroxyacetanilide | 0.80 | 1.88 | 1.86 | -1.08 |
| 2632 | 13-cis-Retinoic acid | 6.30 | 5.22 | 5.07 | 1.08 |
| 2633 | Chlorothiazide | -0.24 | -1.33 | -1.46 | 1.09 |
| 2634 | 2,3,7-Trichlorodibenzo-p-dioxin | 6.46 | 5.36 | 5.36 | 1.10 |
| 2635 | n-Heptylamine | 2.57 | 1.47 | 1.46 | 1.10 |
| 2636 | 1-Octanol | 3.15 | 2.04 | 2.10 | 1.11 |
| 2637 | Quinalbarbitone | 2.39 | 1.28 | 1.27 | 1.11 |
| 2638 | Decanoic acid | 4.09 | 2.98 | 2.97 | 1.11 |
| 2639 | Colchicine | 1.30 | 2.42 | 2.56 | -1.12 |
| 2640 | Xamoterol | 0.61 | -0.51 | -0.63 | 1.12 |
| 2641 | Adriamycin | 0.65 | -0.47 | -0.66 | 1.12 |
| 2642 | 2,3,5,6-Tetramethyl-4-hydroxyacetanilide | 1.44 | 2.56 | 2.55 | -1.12 |
| 2643 | Amylobarbitone | 2.07 | 0.94 | 0.96 | 1.13 |
| 2644 | 1,2,3,4,6-Pentachloronaphthalene | 7.00 | 5.86 | 5.82 | 1.14 |
| 2645 | Saccharin | 0.91 | -0.23 | -0.30 | 1.14 |
| 2646 | 1-Nonene | 5.15 | 4.01 | 4.00 | 1.14 |
| 2647 | Sorbitol | -3.10 | -4.25 | -4.31 | 1.15 |
| 2648 | Butyl 4-hydroxybenzoate | 3.57 | 2.41 | 2.42 | 1.16 |
| 2649 | Hydromorphone | 0.90 | 2.07 | 2.12 | -1.17 |
| 2650 | 1,2,3,4,5,7-Hexachloronaphthalene | 7.58 | 6.41 | 6.38 | 1.17 |
| 2651 | Fluridone | 2.98 | 4.15 | 4.11 | -1.17 |
| 2652 | 1-Bromooctane | 4.89 | 3.71 | 3.73 | 1.18 |
| 2653 | Esomeprazole | 2.23 | 1.04 | 0.63 | 1.19 |
| 2654 | 4-Pyrimidone | -1.38 | -0.19 | 0.18 | -1.19 |
| 2655 | Fluazuron | 5.10 | 6.29 | 6.41 | -1.19 |
| 2656 | 2-Chlorodibenzo-p-dioxin | 5.45 | 4.26 | 4.24 | 1.19 |
| 2657 | Decachlorodiphenyl ether | 8.16 | 9.36 | 9.33 | -1.20 |
| 2658 | Chloroethylene | 2.79 | 1.59 | 1.46 | 1.20 |
| 2659 | 2,2',3,3',5,6,6'-Heptachlorobiphenyl | 6.41 | 7.62 | 7.57 | -1.21 |
| 2660 | 2,2',3,3',4,4',5,5',6-Nonachlorobiphenyl | 7.51 | 8.72 | 8.70 | -1.21 |
| 2661 | 6-Aminonicotinamide | 0.70 | -0.52 | -0.55 | 1.22 |
| 2662 | Frusemide | 2.56 | 1.33 | 1.15 | 1.23 |
| 2663 | 3-Hydroxypicolinamide | 0.65 | -0.60 | -0.66 | 1.25 |
| 2664 | Etorphine | 2.79 | 4.04 | 4.15 | -1.25 |
| 2665 | Methohexital | 2.80 | 1.54 | 1.09 | 1.26 |
| 2666 | Di-n-butyl phthalate | 4.72 | 3.45 | 3.47 | 1.27 |
| 2667 | 1,2,3,4-Tetrachlorodibenzo-p-dioxin | 7.18 | 5.91 | 5.91 | 1.27 |
| 2668 | Butobarbitone | 1.89 | 0.62 | 0.62 | 1.27 |
| 2669 | 5-Amyl-5-ethylbarbituric acid | 2.24 | 0.97 | 1.02 | 1.27 |
| 2670 | Cefadroxil | -0.40 | -1.68 | -1.84 | 1.28 |
| 2671 | Elaidic acid | 6.86 | 5.56 | 5.31 | 1.30 |
| 2672 | Tetrahydro-2H-pyran-2-one | -0.35 | 0.95 | 0.99 | -1.30 |
| 2673 | Helenalin | 0.87 | 2.19 | 2.30 | -1.32 |
| 2674 | 2,3-Butandione | -1.34 | -0.01 | -0.02 | -1.33 |
| 2675 | Roxithromycin | 3.79 | 2.45 | 1.25 | 1.34 |
| 2676 | 8-Azaadenine | -0.96 | 0.39 | 0.81 | -1.35 |
| 2677 | Sulfamethizole | 0.54 | 1.90 | 3.12 | -1.36 |
| 2678 | 4-Hydroxybenzoic acid heptylester | 4.83 | 3.46 | 3.47 | 1.37 |
| 2679 | Verapamil | 3.79 | 5.18 | 5.30 | -1.39 |
| 2680 | Cefalotine | -0.20 | 1.24 | 1.59 | -1.44 |
| 2681 | Pendimethalin | 5.18 | 3.70 | 3.70 | 1.48 |
| 2682 | Dicofol | 4.28 | 5.78 | 5.96 | -1.50 |
| 2683 | Heroine | 1.59 | 3.13 | 3.23 | -1.54 |
| 2684 | Azithromycin | 4.02 | 2.47 | 2.00 | 1.55 |
| 2685 | 3-Amino-1,2,4-triazole | -0.87 | 0.79 | 1.12 | -1.66 |
| 2686 | Tetracaine | 3.73 | 2.05 | 1.99 | 1.68 |
| 2687 | DEHP | 7.88 | 6.19 | 6.14 | 1.69 |
| 2688 | Di-isooctyl phthalate | 8.00 | 6.19 | 5.95 | 1.81 |
| 2689 | n-Decyl alcohol | 4.57 | 2.74 | 2.67 | 1.83 |
| 2690 | Lauryl bromide | 6.98 | 5.11 | 5.10 | 1.87 |
| 2691 | 1-Nonanol | 4.26 | 2.39 | 2.38 | 1.87 |
| 2692 | Cefalexin | 0.65 | -1.29 | -1.45 | 1.94 |
| 2693 | Profluralin | 6.34 | 4.39 | 4.31 | 1.95 |
| 2694 | Oryzalin | 3.73 | 1.76 | 1.64 | 1.97 |
| 2695 | 1-Bromodecane | 6.43 | 4.41 | 4.45 | 2.02 |
| 2696 | Oleic acid | 7.64 | 5.56 | 5.57 | 2.08 |
| 2697 | Stearic acid | 8.23 | 5.78 | 5.93 | 2.45 |
