# Supplementary material for: A Generally Applicable Computer Algorithm Based on the Group Additivity Method for the Calculation of Seven Molecular Descriptors: Heat of Combustion, LogPO/W, LogS, Refractivity, Polarizability, Toxicity and LogBB of Organic Compounds; Scope and Limits of Applicability
Source: Molecules. 2015 Oct 7;20(10):18279–351. doi: 10.3390/molecules201018279 (PMC6332030; doi:10.3390/molecules201018279)
Supplement: Supplementary file 1 [file molecules-20-18279-s001.zip › Experimental vs Calculated LogS Data Table.doc]

**Experimental vs. Calculated LogS Data, sorted by absolute Deviation**

| **No.** | **Molecule name** | **logS** | **logS calc** | **logS test** | **Deviation** |
| --- | --- | --- | --- | --- | --- |
|  |  |  |  |  |  |
| 1 | 3-Methyl-3-hexanol | -1.00 | -1.00 | -0.91 | 0.00 |
| 2 | Pentobarbital | -2.52 | -2.52 | -2.51 | 0.00 |
| 3 | 3-Thiophenecarboxylic acid | -1.47 | -1.47 | -1.42 | 0.00 |
| 4 | 2-Nitroanisol | -1.96 | -1.96 | -1.98 | 0.00 |
| 5 | Methyl methacrylate | -0.80 | -0.80 | -0.84 | 0.00 |
| 6 | Cyclohexylacetate | -1.67 | -1.67 | -1.68 | 0.00 |
| 7 | 2-Nitrotoluene | -2.33 | -2.33 | -2.33 | 0.00 |
| 8 | 1,8-Cineole | -1.64 | -1.64 | -1.77 | 0.00 |
| 9 | Cyfluthrin | -7.34 | -7.34 | -7.32 | 0.00 |
| 10 | Fluorometholone | -4.10 | -4.10 | -4.08 | 0.00 |
| 11 | 2,2',3,4,5,5',6-Heptachlorobiphenyl | -8.94 | -8.94 | -8.90 | 0.00 |
| 12 | 1,2,3,4-Tetrachlorobenzene | -4.57 | -4.56 | -4.53 | -0.01 |
| 13 | 1,2,3-Tribromobenzene | -5.04 | -5.05 | -5.00 | 0.01 |
| 14 | Triptanol | -4.46 | -4.45 | -4.45 | -0.01 |
| 15 | Diallate | -4.08 | -4.09 | -4.12 | 0.01 |
| 16 | 4-Hexylresorcinol | -2.59 | -2.58 | -2.61 | -0.01 |
| 17 | Diethylacetic acid | -0.81 | -0.80 | -0.80 | -0.01 |
| 18 | Ketoprofen | -3.70 | -3.71 | -3.67 | 0.01 |
| 19 | Saccharose | 0.79 | 0.78 | 1.22 | 0.01 |
| 20 | 1,4-Difluorobenzene | -1.97 | -1.98 | -2.01 | 0.01 |
| 21 | Diisopropyl sulfide | -2.24 | -2.25 |  | 0.01 |
| 22 | Chlorpyrifos | -5.49 | -5.50 | -5.61 | 0.01 |
| 23 | 1,1,1-Trichloroethane | -2.00 | -1.99 | -1.93 | -0.01 |
| 24 | 2-Naphthol | -2.28 | -2.29 | -2.29 | 0.01 |
| 25 | 2,4-Dimethyl-2-pentanol | -0.92 | -0.93 | -0.87 | 0.01 |
| 26 | 4-Aminophenol | -0.80 | -0.81 | -0.81 | 0.01 |
| 27 | 2,4-Dimethyl-3-pentanone | -1.30 | -1.31 | -1.37 | 0.01 |
| 28 | Molecule665 | -3.32 | -3.33 | -3.26 | 0.01 |
| 29 | Molecule1047 | -2.09 | -2.10 | -2.10 | 0.01 |
| 30 | p-Terphenyl | -7.11 | -7.12 | -7.06 | 0.01 |
| 31 | Diethylstilbestrol | -4.35 | -4.34 | -4.26 | -0.01 |
| 32 | Triflumethazine | -4.15 | -4.14 | -4.10 | -0.01 |
| 33 | 9-Methylanthracene | -5.89 | -5.90 | -5.89 | 0.01 |
| 34 | alpha-Santonin | -3.09 | -3.07 | -3.07 | -0.02 |
| 35 | 2,2',4,6,6'-Pentachlorobiphenyl | -7.32 | -7.34 | -7.33 | 0.02 |
| 36 | 4-Aminoacetophenone | -1.61 | -1.59 | -1.55 | -0.02 |
| 37 | Hyocholic acid | -4.35 | -4.33 | -4.37 | -0.02 |
| 38 | Molecule1401 | -4.35 | -4.33 | -4.19 | -0.02 |
| 39 | Phenoxyacetic acid | -1.10 | -1.08 | -1.04 | -0.02 |
| 40 | Procaine | -1.40 | -1.42 | -1.45 | 0.02 |
| 41 | Benzaldehyde | -1.19 | -1.21 | -1.16 | 0.02 |
| 42 | Thiamylal | -3.46 | -3.48 | -3.38 | 0.02 |
| 43 | 2-Methyl-1-butanol | -0.47 | -0.45 | -0.45 | -0.02 |
| 44 | Ethyl ether | -0.09 | -0.07 | -0.13 | -0.02 |
| 45 | Camphoronic acid | -0.29 | -0.31 | -0.47 | 0.02 |
| 46 | 1,3-Difluorobenzene | -2.00 | -1.98 | -2.00 | -0.02 |
| 47 | Ethyl iodide | -1.60 | -1.62 | -1.39 | 0.02 |
| 48 | Ioxynil | -3.61 | -3.59 | -3.73 | -0.02 |
| 49 | Linalol | -1.99 | -2.01 | -2.07 | 0.02 |
| 50 | Molecule559 | -4.13 | -4.15 | -4.13 | 0.02 |
| 51 | Nitrofen | -5.46 | -5.48 | -5.31 | 0.02 |
| 52 | Methotrimeprazine | -4.22 | -4.19 | -4.15 | -0.03 |
| 53 | Diethyl phthalate | -2.35 | -2.38 | -2.41 | 0.03 |
| 54 | Molecule428 | -3.90 | -3.87 | -3.93 | -0.03 |
| 55 | n-Hexylamine | -1.10 | -1.07 | -1.05 | -0.03 |
| 56 | 2-Heptanone | -1.42 | -1.45 | -1.44 | 0.03 |
| 57 | Molecule1050 | -1.58 | -1.55 | -1.55 | -0.03 |
| 58 | Molecule645 | -3.68 | -3.71 | -3.73 | 0.03 |
| 59 | 2,2',4,5'-Tetrachlorobiphenyl | -6.57 | -6.54 | -6.53 | -0.03 |
| 60 | Antipyrine | -0.56 | -0.53 | -0.49 | -0.03 |
| 61 | n-Propylformate | -0.49 | -0.46 | -0.47 | -0.03 |
| 62 | 2-Methylbenzimidazole | -1.96 | -1.99 | -1.95 | 0.03 |
| 63 | o-Anthranilic acid | -1.52 | -1.49 | -1.48 | -0.03 |
| 64 | Dimethoxymethane | 0.48 | 0.51 | 0.58 | -0.03 |
| 65 | o-Cymene | -3.76 | -3.73 | -3.71 | -0.03 |
| 66 | MTBE | -0.24 | -0.27 | -0.36 | 0.03 |
| 67 | 2,2',4,4'-Tetrachlorobiphenyl | -6.51 | -6.54 | -6.57 | 0.03 |
| 68 | Benzotrifluoride | -2.51 | -2.54 | -2.38 | 0.03 |
| 69 | 2-Hexanol | -0.89 | -0.92 | -0.86 | 0.03 |
| 70 | N-Ethylaniline | -1.70 | -1.67 | -1.64 | -0.03 |
| 71 | Monuron | -2.89 | -2.86 | -2.86 | -0.03 |
| 72 | 1,2-Dibromopropane | -2.15 | -2.18 |  | 0.03 |
| 73 | Bromacil | -2.52 | -2.55 |  | 0.03 |
| 74 | Kebuzone | -3.27 | -3.30 | -3.38 | 0.03 |
| 75 | DDE | -6.90 | -6.86 | -6.82 | -0.04 |
| 76 | Devrinol | -3.57 | -3.53 | -3.53 | -0.04 |
| 77 | Bromobenzene | -2.55 | -2.59 | -2.55 | 0.04 |
| 78 | trans-3-Pentenenitrile | -0.96 | -0.92 | -0.92 | -0.04 |
| 79 | Cantharidic acid | -0.27 | -0.23 | -0.10 | -0.04 |
| 80 | Butyric acid | -0.19 | -0.23 | -0.23 | 0.04 |
| 81 | 1-Isopropyl-4-methylbenzene | -3.77 | -3.73 | -3.75 | -0.04 |
| 82 | Cumene | -3.27 | -3.23 | -3.22 | -0.04 |
| 83 | 3-Chloronitrobenzene | -2.77 | -2.73 | -2.64 | -0.04 |
| 84 | Molecule1395 | -1.73 | -1.77 | -1.73 | 0.04 |
| 85 | Ethyl acrylate | -0.74 | -0.78 | -0.74 | 0.04 |
| 86 | Molecule1435 | -0.89 | -0.93 | -0.94 | 0.04 |
| 87 | Morpholine | 1.06 | 1.10 | 1.08 | -0.04 |
| 88 | 2,3-Dimethyl-1,3-butadiene | -2.40 | -2.36 | -2.41 | -0.04 |
| 89 | Prasterone | -4.12 | -4.16 | -4.17 | 0.04 |
| 90 | Molecule1066 | -2.36 | -2.32 | -2.29 | -0.04 |
| 91 | Pencycuron | -5.91 | -5.95 | -5.87 | 0.04 |
| 92 | Guajen | -4.72 | -4.68 | -4.64 | -0.04 |
| 93 | Teflubenzuron | -7.28 | -7.32 | -7.46 | 0.04 |
| 94 | 1,1,3-Trimethylcyclopentane | -4.48 | -4.52 | -4.44 | 0.04 |
| 95 | Propylcyclopentane | -4.74 | -4.69 | -4.66 | -0.05 |
| 96 | Chavicine | -3.46 | -3.41 | -3.41 | -0.05 |
| 97 | Molecule945 | -0.73 | -0.68 | -0.52 | -0.05 |
| 98 | Androsterone | -4.16 | -4.21 | -4.10 | 0.05 |
| 99 | Neopentyl alcohol | -0.40 | -0.35 | -0.32 | -0.05 |
| 100 | 4-Methyl-2-pentanol | -0.80 | -0.85 | -0.87 | 0.05 |
| 101 | Amylobarbitone | -2.57 | -2.52 | -2.38 | -0.05 |
| 102 | 1,1-Diethoxyethane | -0.43 | -0.38 | -0.50 | -0.05 |
| 103 | (L)-Methionine | -0.42 | -0.37 | -0.31 | -0.05 |
| 104 | Ethyl propionate | -0.66 | -0.61 | -0.61 | -0.05 |
| 105 | 3-Methyl-1-pentanol | -0.72 | -0.77 | -0.77 | 0.05 |
| 106 | Levosalbutamol | -1.22 | -1.27 | -1.03 | 0.05 |
| 107 | Camphor | -1.99 | -2.04 | -2.06 | 0.05 |
| 108 | Methyl isoamyl ketone | -1.33 | -1.38 | -1.40 | 0.05 |
| 109 | 2,3',4,4',5-Pentachlorobiphenyl | -7.39 | -7.34 | -7.36 | -0.05 |
| 110 | 1,3,5-Cycloheptatriene | -2.15 | -2.20 | -2.22 | 0.05 |
| 111 | 2,3-Dichloronitrobenzene | -3.48 | -3.53 | -3.52 | 0.05 |
| 112 | MCPA | -2.23 | -2.28 | -2.27 | 0.05 |
| 113 | Trifluoperazine | -4.52 | -4.47 | -4.47 | -0.05 |
| 114 | 1,4-Diethylbenzene | -3.75 | -3.80 | -3.87 | 0.05 |
| 115 | 1-Methylphenanthrene | -5.85 | -5.90 | -5.82 | 0.05 |
| 116 | Epitiostanol | -5.41 | -5.35 |  | -0.06 |
| 117 | Benzo[f]quinoline | -3.36 | -3.42 | -3.37 | 0.06 |
| 118 | 1,3,5-Trimethylbenzene | -3.40 | -3.46 | -3.46 | 0.06 |
| 119 | 1-Bromo-3-chloropropane | -1.85 | -1.79 | -1.84 | -0.06 |
| 120 | Molecule540 | 0.52 | 0.46 | 0.07 | 0.06 |
| 121 | Dulcin | -2.17 | -2.23 | -2.27 | 0.06 |
| 122 | Dimethyl sulfide | -0.45 | -0.39 | -0.49 | -0.06 |
| 123 | 3-Nitrobenzoic acid | -1.68 | -1.74 | -1.72 | 0.06 |
| 124 | 1,1,2-Trichloroethane | -1.48 | -1.42 | -1.42 | -0.06 |
| 125 | Trimazosin | -3.64 | -3.70 | -3.84 | 0.06 |
| 126 | Glafenine | -4.57 | -4.51 | -4.59 | -0.06 |
| 127 | Cloprop | -2.22 | -2.28 | -2.25 | 0.06 |
| 128 | Molecule376 | -2.22 | -2.28 | -2.35 | 0.06 |
| 129 | 1,5-Dimethylnaphthalene | -4.74 | -4.68 | -4.66 | -0.06 |
| 130 | 2-Methylphenanthrene | -5.84 | -5.90 | -5.89 | 0.06 |
| 131 | Benzo[ghi]perylene | -9.03 | -8.96 | -9.11 | -0.07 |
| 132 | 1,2,3,5-Tetrachlorobenzene | -4.63 | -4.56 | -4.52 | -0.07 |
| 133 | Lysodren | -6.51 | -6.44 | -6.45 | -0.07 |
| 134 | Isocumene | -3.37 | -3.30 | -3.29 | -0.07 |
| 135 | Dicapthon | -4.31 | -4.24 | -4.24 | -0.07 |
| 136 | Benzo[b]fluoranthene | -8.23 | -8.16 | -7.95 | -0.07 |
| 137 | Sparsomycin | -1.98 | -1.91 |  | -0.07 |
| 138 | 2-Ethylhexylamine | -1.71 | -1.64 | -1.32 | -0.07 |
| 139 | Ethyl bromide | -1.09 | -1.16 | -1.29 | 0.07 |
| 140 | Chlortetracycline | -2.88 | -2.81 | -2.85 | -0.07 |
| 141 | Isopentyl alcohol | -0.52 | -0.45 | -0.43 | -0.07 |
| 142 | 1-Naphthol | -2.22 | -2.29 | -2.27 | 0.07 |
| 143 | Isoprene | -2.03 | -1.96 | -1.99 | -0.07 |
| 144 | 2,2',3,5'-Tetrachlorobiphenyl | -6.47 | -6.54 | -6.45 | 0.07 |
| 145 | 2-Furoic acid | -0.48 | -0.55 | -0.60 | 0.07 |
| 146 | Alachlor | -3.26 | -3.19 | -3.05 | -0.07 |
| 147 | 4,5-Dichloroguaiacol | -2.53 | -2.60 | -2.61 | 0.07 |
| 148 | Testosterone | -4.09 | -4.16 | -4.18 | 0.07 |
| 149 | Acenaphthene | -4.63 | -4.70 | -4.71 | 0.07 |
| 150 | Chloroxuron | -4.89 | -4.81 | -4.83 | -0.08 |
| 151 | Hiltachlor | -4.19 | -4.11 | -4.19 | -0.08 |
| 152 | 2,4-Dinitrotoluene | -2.82 | -2.90 | -2.92 | 0.08 |
| 153 | Androstane-3b,11b-diol-17-one | -3.59 | -3.67 | -3.67 | 0.08 |
| 154 | 2,2-Dimethyl-3-pentanol | -1.15 | -1.07 | -1.12 | -0.08 |
| 155 | Ethylisopropylether | -0.55 | -0.47 | -0.49 | -0.08 |
| 156 | 2-Chlorobenzoic acid | -1.89 | -1.97 | -1.94 | 0.08 |
| 157 | 1-Pentanol | -0.60 | -0.52 | -0.55 | -0.08 |
| 158 | 2-Methyl-2-hexanol | -1.08 | -1.00 | -1.02 | -0.08 |
| 159 | Indol-3-ylacetic acid | -2.07 | -1.99 | -2.06 | -0.08 |
| 160 | 1,3-Dichlorobenzene | -3.04 | -2.96 | -2.96 | -0.08 |
| 161 | Diethyl succinate | -0.96 | -1.04 | -1.06 | 0.08 |
| 162 | 2,3-Dimethyl-3-pentanol | -0.85 | -0.93 | -0.89 | 0.08 |
| 163 | 5-Ethyl-5-methylbarbituric acid | -1.23 | -1.31 | -1.29 | 0.08 |
| 164 | Propachlor | -2.48 | -2.56 | -2.54 | 0.08 |
| 165 | Fluconazole | -1.80 | -1.88 | -0.37 | 0.08 |
| 166 | Naphthalene | -3.60 | -3.68 | -3.72 | 0.08 |
| 167 | Thioanisole | -2.39 | -2.47 | -2.49 | 0.08 |
| 168 | Pregnenolone | -4.65 | -4.73 | -4.64 | 0.08 |
| 169 | 6-Aminopurine | -2.12 | -2.03 | -1.83 | -0.09 |
| 170 | Molecule747 | -2.12 | -2.03 | -2.14 | -0.09 |
| 171 | Molecule1353 | -4.58 | -4.49 | -4.60 | -0.09 |
| 172 | Pentylcyclopentane | -6.08 | -5.99 | -5.83 | -0.09 |
| 173 | XMC | -2.58 | -2.49 | -2.54 | -0.09 |
| 174 | 1,4-Cyclohexadiene | -1.97 | -1.88 | -1.88 | -0.09 |
| 175 | Ampyrone | -0.62 | -0.71 | -0.79 | 0.09 |
| 176 | Isopropyl formate | -0.63 | -0.54 | -0.51 | -0.09 |
| 177 | Ethanethiol | -0.60 | -0.51 |  | -0.09 |
| 178 | 1,2-Dichlorobenzene | -3.05 | -2.96 | -2.91 | -0.09 |
| 179 | 2-Aminophenol | -0.72 | -0.81 | -0.80 | 0.09 |
| 180 | Methylcyclopentane | -3.30 | -3.39 | -3.39 | 0.09 |
| 181 | 2,2',3,4',6-Pentachlorobiphenyl | -7.43 | -7.34 | -7.26 | -0.09 |
| 182 | 2,2',3,4,6-Pentachlorobiphenyl | -7.43 | -7.34 | -7.28 | -0.09 |
| 183 | Hexestrol | -4.43 | -4.34 | -4.31 | -0.09 |
| 184 | Medrogestone | -5.27 | -5.36 | -5.36 | 0.09 |
| 185 | Kepone | -5.26 | -5.16 | -4.95 | -0.10 |
| 186 | Heptachlor | -6.32 | -6.42 | -6.42 | 0.10 |
| 187 | Molecule1087 | -1.57 | -1.67 | -1.58 | 0.10 |
| 188 | Fludrocortisone | -3.43 | -3.33 | -3.26 | -0.10 |
| 189 | Citric acid | 0.51 | 0.61 | 0.62 | -0.10 |
| 190 | Ethylenethiourea | -0.71 | -0.61 | -0.59 | -0.10 |
| 191 | Propionanilide | -1.92 | -2.02 | -1.97 | 0.10 |
| 192 | Diethyl malonate | -0.82 | -0.72 | -0.76 | -0.10 |
| 193 | Atropic acid | -2.06 | -1.96 | -1.87 | -0.10 |
| 194 | Dimethyl disulfide | -1.44 | -1.54 |  | 0.10 |
| 195 | Pelletierine | -0.45 | -0.55 | -0.60 | 0.10 |
| 196 | Molecule299 | -4.52 | -4.42 | -4.39 | -0.10 |
| 197 | 2,6-Dinitrotoluene | -3.00 | -2.90 | -2.89 | -0.10 |
| 198 | N-Methylaniline | -1.28 | -1.38 | -1.37 | 0.10 |
| 199 | Norethindrone | -4.57 | -4.47 | -4.37 | -0.10 |
| 200 | Diphenylacetic acid | -3.22 | -3.32 | -3.34 | 0.10 |
| 201 | Cortisol | -2.97 | -3.07 | -3.11 | 0.10 |
| 202 | 2,2,5-Trimethylhexane | -5.05 | -5.15 | -5.18 | 0.10 |
| 203 | PCNB | -5.82 | -5.93 | -5.93 | 0.11 |
| 204 | t-Butylbenzene | -3.66 | -3.55 | -3.60 | -0.11 |
| 205 | N-Nitrosodiphenylamine | -3.75 | -3.86 | -4.57 | 0.11 |
| 206 | Ethylbenzene | -2.77 | -2.88 | -2.84 | 0.11 |
| 207 | Isoquinoline | -1.45 | -1.34 | -1.31 | -0.11 |
| 208 | 3-Nitrotoluene | -2.44 | -2.33 | -2.32 | -0.11 |
| 209 | Chloroacetamide | -0.02 | 0.09 | 0.20 | -0.11 |
| 210 | Vinylidenechloride | -1.64 | -1.75 | -1.76 | 0.11 |
| 211 | Anisomycin | -1.61 | -1.50 | -1.51 | -0.11 |
| 212 | n-Propyl acetate | -0.72 | -0.61 | -0.62 | -0.11 |
| 213 | 1,4-Pentadiene | -2.09 | -1.98 | -2.02 | -0.11 |
| 214 | Dulcitol | 1.09 | 0.98 | 0.98 | 0.11 |
| 215 | Propylene | -1.08 | -0.97 | -0.97 | -0.11 |
| 216 | cis-3,7-Dimethyl-2,6-octadienal | -2.06 | -1.95 | -1.85 | -0.11 |
| 217 | Propyl propanoate | -0.82 | -0.93 | -0.96 | 0.11 |
| 218 | Phenyl N-methylcarbamate | -1.80 | -1.69 | -1.69 | -0.11 |
| 219 | 2,3,3-Trimethyl-2-butanol | -0.72 | -0.83 | -0.88 | 0.11 |
| 220 | 1-Butyne | -1.24 | -1.35 | -1.54 | 0.11 |
| 221 | 1,2-Dibromo-3-chloropropane | -2.38 | -2.49 | -2.65 | 0.11 |
| 222 | Diflubenzuron | -6.02 | -5.90 | -5.90 | -0.12 |
| 223 | Bromocyclohexane | -2.30 | -2.18 | -2.10 | -0.12 |
| 224 | Thymol | -2.22 | -2.34 | -2.39 | 0.12 |
| 225 | Molecule1422 | -1.27 | -1.15 | -1.17 | -0.12 |
| 226 | 2,4-Dimethyl-3-pentanol | -1.22 | -1.10 | -1.11 | -0.12 |
| 227 | 2,4,6-Trimethylphenol | -2.05 | -2.17 | -2.25 | 0.12 |
| 228 | Molecule966 | -4.16 | -4.04 | -4.20 | -0.12 |
| 229 | Isoleucine | -0.59 | -0.47 | -0.51 | -0.12 |
| 230 | Carbostyril | -2.14 | -2.02 | -2.06 | -0.12 |
| 231 | Atrazine | -3.85 | -3.73 | -3.85 | -0.12 |
| 232 | 3-Hexanol | -0.80 | -0.92 | -0.93 | 0.12 |
| 233 | Molecule1204 | -1.56 | -1.44 | -1.46 | -0.12 |
| 234 | Molecule571 | -4.21 | -4.33 | -4.23 | 0.12 |
| 235 | Pseudococaine | -2.23 | -2.35 | -2.44 | 0.12 |
| 236 | Coumaphos | -5.38 | -5.26 | -5.37 | -0.12 |
| 237 | Molecule460 | -5.38 | -5.26 | -5.44 | -0.12 |
| 238 | 1,1,1,2-Tetrachloroethane | -2.18 | -2.30 | -2.27 | 0.12 |
| 239 | Chlorpyrifos-methyl | -4.82 | -4.94 | -5.28 | 0.12 |
| 240 | 2,2',3,4',5'-Pentachlorobiphenyl | -7.21 | -7.34 | -7.33 | 0.13 |
| 241 | 2,2',3,4,5-Pentachlorobiphenyl | -7.21 | -7.34 | -7.28 | 0.13 |
| 242 | Heptabarbital | -3.00 | -3.13 | -3.09 | 0.13 |
| 243 | Nitrobenzene | -1.80 | -1.93 | -1.92 | 0.13 |
| 244 | 1-Methyluracil | -0.80 | -0.67 | -0.66 | -0.13 |
| 245 | 3-Methyl-2-pentanol | -0.72 | -0.85 | -0.85 | 0.13 |
| 246 | 3,3-Dimethyl-2-butanol | -0.62 | -0.75 | -0.78 | 0.13 |
| 247 | Triallate | -4.88 | -4.75 | -4.86 | -0.13 |
| 248 | Isopropyl methyl ether | -0.06 | -0.19 | -0.20 | 0.13 |
| 249 | 1-Pentyne | -1.64 | -1.77 | -1.83 | 0.13 |
| 250 | Fluorobenzene | -1.80 | -1.67 | -1.69 | -0.13 |
| 251 | N-Methylmorpholine | 1.00 | 1.13 | 1.17 | -0.13 |
| 252 | Mecoprop | -2.55 | -2.68 | -2.67 | 0.13 |
| 253 | Molecule827 | -2.59 | -2.72 | -2.72 | 0.13 |
| 254 | Hexachloroethane | -3.67 | -3.80 | -3.90 | 0.13 |
| 255 | Pyrene | -6.19 | -6.32 | -6.30 | 0.13 |
| 256 | 2,2',3,3',4,4'-Hexachlorobiphenyl | -8.01 | -8.14 | -8.08 | 0.13 |
| 257 | 2,2',3,3',4,5,5',6,6'-Nonachlorobiphenyl | -10.41 | -10.54 | -10.48 | 0.13 |
| 258 | Diuron | -3.80 | -3.66 | -3.64 | -0.14 |
| 259 | Chloramben | -2.47 | -2.61 | -2.58 | 0.14 |
| 260 | 4-Hydroxybenzaldehyde | -0.96 | -0.82 | -0.75 | -0.14 |
| 261 | Molecule1110 | -0.96 | -0.82 |  | -0.14 |
| 262 | m-Xylene | -2.82 | -2.96 | -2.99 | 0.14 |
| 263 | Molecule1035 | -0.80 | -0.66 | -0.63 | -0.14 |
| 264 | Trimethylamine | 0.84 | 0.98 | 1.03 | -0.14 |
| 265 | Chlordimeform | -2.86 | -3.00 | -3.05 | 0.14 |
| 266 | Molecule1042 | -0.30 | -0.44 | -0.41 | 0.14 |
| 267 | Isopropylacetate | -0.55 | -0.69 | -0.69 | 0.14 |
| 268 | Isobutyl acrylate | -1.21 | -1.35 | -1.38 | 0.14 |
| 269 | Bentranil | -4.61 | -4.47 | -4.49 | -0.14 |
| 270 | Phenanthrene | -5.26 | -5.40 | -5.30 | 0.14 |
| 271 | Benzo[a]pyrene | -8.19 | -8.04 | -7.90 | -0.15 |
| 272 | Molecule240 | -5.46 | -5.31 | -5.28 | -0.15 |
| 273 | Molecule857 | -6.78 | -6.93 | -7.01 | 0.15 |
| 274 | p-Bromoacetanilide | -3.08 | -2.93 | -2.97 | -0.15 |
| 275 | 4-Heptanone | -1.30 | -1.45 | -1.47 | 0.15 |
| 276 | 1,2,4-Trimethylbenzene | -3.31 | -3.46 | -3.54 | 0.15 |
| 277 | 1-Bromo-2-chloroethane | -1.32 | -1.47 | -1.44 | 0.15 |
| 278 | 2-Methyl-3-pentanol | -0.70 | -0.85 | -0.88 | 0.15 |
| 279 | Molecule1040 | -0.66 | -0.51 | -0.49 | -0.15 |
| 280 | 3-Ethyl-3-pentanol | -0.85 | -1.00 | -1.01 | 0.15 |
| 281 | Thiamphenicol | -2.15 | -2.00 | -2.33 | -0.15 |
| 282 | Valine | -0.30 | -0.15 | -0.09 | -0.15 |
| 283 | cis-1,4-Dimethylcyclohexane | -4.47 | -4.62 | -4.57 | 0.15 |
| 284 | Allyl bromide | -1.50 | -1.65 | -1.60 | 0.15 |
| 285 | Skatole | -2.42 | -2.26 | -2.27 | -0.16 |
| 286 | 1,7-Phenanthroline | -2.68 | -2.84 | -2.89 | 0.16 |
| 287 | Karbutilate | -2.93 | -3.09 | -3.41 | 0.16 |
| 288 | Molecule647 | -2.68 | -2.84 | -2.79 | 0.16 |
| 289 | Benzylamine | -1.53 | -1.37 | -1.36 | -0.16 |
| 290 | 4-Nitrotoluene | -2.49 | -2.33 | -2.31 | -0.16 |
| 291 | o-Xylene | -2.80 | -2.96 | -2.99 | 0.16 |
| 292 | 4-Heptanol | -1.40 | -1.24 | -1.25 | -0.16 |
| 293 | N-Methyl-N-nitrosourea | -0.85 | -1.01 |  | 0.16 |
| 294 | Betamethasone | -3.64 | -3.48 | -3.47 | -0.16 |
| 295 | Glycerol | 1.12 | 1.28 | 1.31 | -0.16 |
| 296 | Molecule1192 | -5.37 | -5.21 | -5.11 | -0.16 |
| 297 | 1-Naphthaleneacetic acid | -2.65 | -2.81 | -2.86 | 0.16 |
| 298 | Dimethyl phthalate | -1.66 | -1.82 | -1.88 | 0.16 |
| 299 | Molecule995 | -1.59 | -1.43 | -1.47 | -0.16 |
| 300 | 1-(Chloromethyl)-4-fluorobenzene | -2.54 | -2.38 | -2.32 | -0.16 |
| 301 | Benzo[j]fluoranthene | -8.00 | -8.16 | -8.15 | 0.16 |
| 302 | Dibenzo[b,d]thiophene | -4.38 | -4.22 | -4.14 | -0.16 |
| 303 | Triamcinolone acetonide | -4.32 | -4.48 | -4.61 | 0.16 |
| 304 | Molecule465 | -4.23 | -4.06 | -4.00 | -0.17 |
| 305 | 9,10-Dimethylanthracene | -6.57 | -6.40 | -6.38 | -0.17 |
| 306 | Molecule1090 | -4.24 | -4.07 | -4.09 | -0.17 |
| 307 | 2-Ethyltoluene | -3.21 | -3.38 | -3.45 | 0.17 |
| 308 | Molecule518 | -3.77 | -3.60 | -3.60 | -0.17 |
| 309 | Butyric acid methylester | -0.82 | -0.65 | -0.67 | -0.17 |
| 310 | N-Methylpiperidine | 0.23 | 0.06 | 0.08 | 0.17 |
| 311 | 2-Methyl-1-propanol | 0.04 | -0.13 | -0.12 | 0.17 |
| 312 | 3,3-Dimethyl-1-butanol | -0.50 | -0.67 | -0.70 | 0.17 |
| 313 | 1-Hexyne | -2.36 | -2.19 | -2.21 | -0.17 |
| 314 | Cyclopentane | -2.64 | -2.81 | -2.72 | 0.17 |
| 315 | Styrene oxide | -1.60 | -1.43 | -1.42 | -0.17 |
| 316 | 2,4-Dichlorophenoxyacetic acid | -2.51 | -2.68 | -2.68 | 0.17 |
| 317 | Molecule378 | -4.18 | -4.01 | -3.93 | -0.17 |
| 318 | 1,2,4-Trichlorobenzene | -3.59 | -3.76 | -3.72 | 0.17 |
| 319 | Cortisone | -3.11 | -3.28 | -3.32 | 0.17 |
| 320 | DDT | -7.15 | -7.32 | -7.22 | 0.17 |
| 321 | 2,2',3,4,4',5'-Hexachlorobiphenyl | -8.32 | -8.14 | -8.09 | -0.18 |
| 322 | (1,1-Dimethylpropyl)benzene | -4.15 | -3.97 | -4.03 | -0.18 |
| 323 | (2,2-Dimethylpropyl)benzene | -4.15 | -3.97 | -4.02 | -0.18 |
| 324 | Dibenz[ah]anthracene | -8.66 | -8.84 | -8.81 | 0.18 |
| 325 | 1,1-Dichloroethane | -1.29 | -1.11 | -1.13 | -0.18 |
| 326 | Methocarbamol | -0.99 | -1.17 | -1.14 | 0.18 |
| 327 | 2-Chloronitrobenzene | -2.55 | -2.73 | -2.73 | 0.18 |
| 328 | Pirimicarb | -1.95 | -1.77 | -1.09 | -0.18 |
| 329 | Oxycarboxin | -2.43 | -2.25 | -2.32 | -0.18 |
| 330 | Meconin | -1.89 | -1.71 | -1.85 | -0.18 |
| 331 | Tyramine | -1.12 | -1.30 | -1.27 | 0.18 |
| 332 | Phenylbutazone | -3.81 | -3.63 | -3.67 | -0.18 |
| 333 | 3-Bromochlorobenzene | -3.21 | -3.39 | -3.38 | 0.18 |
| 334 | Neburon | -4.77 | -4.59 | -4.70 | -0.18 |
| 335 | 3,4,5,6-Tetrachloroguaiacol | -4.02 | -4.20 | -4.24 | 0.18 |
| 336 | 1,3,5-Trinitrobenzene | -2.89 | -3.07 | -3.06 | 0.18 |
| 337 | Benzo[a]fluorene | -6.68 | -6.86 | -6.83 | 0.18 |
| 338 | Fluometuron | -3.43 | -3.24 | -3.12 | -0.19 |
| 339 | Butabarbital | -2.39 | -2.20 | -2.14 | -0.19 |
| 340 | Molecule1704 | -3.37 | -3.18 | -3.24 | -0.19 |
| 341 | Megestrol acetate | -5.35 | -5.16 | -5.15 | -0.19 |
| 342 | Mecarbam | -2.52 | -2.71 | -2.85 | 0.19 |
| 343 | 1,4-Dimethylbenzene | -2.77 | -2.96 | -2.96 | 0.19 |
| 344 | Di-n-propylamine | -0.46 | -0.27 | -0.26 | -0.19 |
| 345 | Molecule454 | -3.79 | -3.98 | -4.11 | 0.19 |
| 346 | n-Hexanoic acid | -1.06 | -0.87 | -0.87 | -0.19 |
| 347 | Propionic acid methylester | -0.14 | -0.33 | -0.35 | 0.19 |
| 348 | 4-Chloronitrobenzene | -2.92 | -2.73 | -2.64 | -0.19 |
| 349 | Fenchone | -1.85 | -2.04 | -2.00 | 0.19 |
| 350 | Methylcyclohexane | -3.85 | -4.04 | -3.99 | 0.19 |
| 351 | 2-Methyl-2-pentanol | -0.49 | -0.68 | -0.75 | 0.19 |
| 352 | Fenthion | -4.57 | -4.38 | -4.42 | -0.19 |
| 353 | Molecule853 | -4.47 | -4.66 | -4.83 | 0.19 |
| 354 | Molecule955 | -2.24 | -2.05 | -2.02 | -0.19 |
| 355 | Barban | -4.37 | -4.18 | -4.13 | -0.19 |
| 356 | Molecule1354 | -4.37 | -4.18 | -4.22 | -0.19 |
| 357 | Dimefuron | -4.33 | -4.52 | -4.58 | 0.19 |
| 358 | Triazolam | -4.08 | -4.27 | -2.47 | 0.19 |
| 359 | 1-Aminoacridine | -4.22 | -4.02 | -3.99 | -0.20 |
| 360 | Chlorotoluron | -3.46 | -3.26 | -3.25 | -0.20 |
| 361 | 4-Bromotoluene | -3.19 | -2.99 | -2.98 | -0.20 |
| 362 | Cycloate | -3.40 | -3.20 | -3.14 | -0.20 |
| 363 | Isoprocarb | -2.86 | -2.66 | -2.63 | -0.20 |
| 364 | 1-Bromo-2-fluorobenzene | -2.70 | -2.90 | -2.90 | 0.20 |
| 365 | 2,3-Dimethyl-2-butanol | -0.41 | -0.61 | -0.62 | 0.20 |
| 366 | Parabanic acid | -0.40 | -0.20 | -0.20 | -0.20 |
| 367 | 1-Butanol | 0.00 | -0.20 | -0.19 | 0.20 |
| 368 | 1-Bromo-2-chlorobenzene | -3.19 | -3.39 | -3.37 | 0.20 |
| 369 | Fenitrothion | -4.04 | -3.84 | -3.86 | -0.20 |
| 370 | Molecule555 | -4.90 | -4.69 | -4.74 | -0.21 |
| 371 | Molecule556 | -4.90 | -4.69 | -4.52 | -0.21 |
| 372 | Diphenylamine | -3.51 | -3.30 | -3.30 | -0.21 |
| 373 | Thiopropazate | -4.70 | -4.49 | -4.51 | -0.21 |
| 374 | Hexachlorobutadiene | -4.91 | -4.70 | -4.68 | -0.21 |
| 375 | 2-Chloroiodobenzene | -3.54 | -3.33 | -3.26 | -0.21 |
| 376 | 2-Aminobutyric acid | 0.31 | 0.10 | 0.07 | 0.21 |
| 377 | Methyl nicotinate | -0.46 | -0.25 | -0.04 | -0.21 |
| 378 | m-Dinitrobenzene | -2.29 | -2.50 | -2.45 | 0.21 |
| 379 | N-Nitrosopiperidine | -0.17 | 0.04 | 0.23 | -0.21 |
| 380 | Furan | -0.82 | -0.61 | -0.59 | -0.21 |
| 381 | Morin | -3.08 | -3.29 | -3.41 | 0.21 |
| 382 | 5-Methyl-2-hexanol | -1.38 | -1.17 | -1.19 | -0.21 |
| 383 | Benzophenone | -3.12 | -3.33 | -3.34 | 0.21 |
| 384 | Chloropropham | -3.38 | -3.17 | -3.15 | -0.21 |
| 385 | Pethidine | -1.89 | -2.10 | -2.05 | 0.21 |
| 386 | Formetanate | -2.34 | -2.13 | -2.19 | -0.21 |
| 387 | Dapsone | -2.82 | -2.61 | -2.51 | -0.21 |
| 388 | Trifluopromazine | -5.30 | -5.09 | -5.02 | -0.21 |
| 389 | Etryptamine | -2.57 | -2.78 | -2.81 | 0.21 |
| 390 | 2,6-Dimethylnaphthalene | -4.89 | -4.68 | -4.61 | -0.21 |
| 391 | Molecule238 | -5.64 | -5.85 | -5.86 | 0.21 |
| 392 | Chlorobenzene | -2.38 | -2.16 | -2.10 | -0.22 |
| 393 | Siduron | -4.11 | -3.89 | -3.82 | -0.22 |
| 394 | 1,2-Dichloropropane | -1.60 | -1.38 | -1.43 | -0.22 |
| 395 | Desipramine | -3.66 | -3.88 | -3.64 | 0.22 |
| 396 | Salicylanilide | -3.59 | -3.37 | -3.25 | -0.22 |
| 397 | 3-Chloroiodobenzene | -3.55 | -3.33 | -3.52 | -0.22 |
| 398 | Carbofuran | -2.80 | -2.58 | -2.59 | -0.22 |
| 399 | Benzamide | -0.96 | -1.18 | -1.18 | 0.22 |
| 400 | Aminopyrine | -0.63 | -0.85 | -1.03 | 0.22 |
| 401 | 1-Nitropropane | -0.80 | -1.02 | -1.44 | 0.22 |
| 402 | Cinchocaine | -3.70 | -3.48 | -3.55 | -0.22 |
| 403 | Cinchonidine | -3.07 | -3.29 | -3.30 | 0.22 |
| 404 | 1,2,4,5-Tetrafluorobenzene | -2.38 | -2.60 | -2.64 | 0.22 |
| 405 | Butallylonal | -2.65 | -2.87 |  | 0.22 |
| 406 | Molecule385 | -2.83 | -2.61 | -2.51 | -0.22 |
| 407 | Molecule815 | -2.25 | -2.47 | -2.55 | 0.22 |
| 408 | Fluorene | -4.91 | -5.14 | -5.23 | 0.23 |
| 409 | Vegiben 2E | -3.26 | -3.03 | -3.00 | -0.23 |
| 410 | Chlordane | -6.86 | -7.09 | -7.14 | 0.23 |
| 411 | Diallyl phthalate | -3.13 | -3.36 | -3.41 | 0.23 |
| 412 | Molecule1216 | -1.60 | -1.37 | -1.31 | -0.23 |
| 413 | 1-Bromo-3-fluorobenzene | -2.67 | -2.90 | -2.84 | 0.23 |
| 414 | Hydantoin | -0.40 | -0.17 | -0.13 | -0.23 |
| 415 | Diisopropyl ether | -1.10 | -0.87 | -0.80 | -0.23 |
| 416 | 3-Heptanol | -1.47 | -1.24 | -1.27 | -0.23 |
| 417 | 3-Methyl-2-heptanol | -1.72 | -1.49 | -1.51 | -0.23 |
| 418 | 2,5-Xylenol | -1.54 | -1.77 | -1.77 | 0.23 |
| 419 | Molecule1627 | -1.46 | -1.23 | -1.38 | -0.23 |
| 420 | Biphenyl | -4.31 | -4.54 | -4.50 | 0.23 |
| 421 | 1,2,3-Trichloropropane | -1.92 | -1.69 | -1.60 | -0.23 |
| 422 | Piperophos | -4.15 | -3.92 | -3.93 | -0.23 |
| 423 | Vitavax | -3.14 | -3.37 | -3.40 | 0.23 |
| 424 | Chloroxylenol | -2.80 | -2.57 | -2.49 | -0.23 |
| 425 | 4-Fluoroacetanilide | -1.78 | -2.01 | -2.03 | 0.23 |
| 426 | Styrene | -2.82 | -3.05 | -3.02 | 0.23 |
| 427 | 1,2,3-Trichlorobenzene | -4.00 | -3.76 | -3.79 | -0.24 |
| 428 | 1-Phenyl-1-propanone | -1.83 | -2.07 | -2.11 | 0.24 |
| 429 | 1-Bromo-4-chlorobenzene | -3.63 | -3.39 | -3.34 | -0.24 |
| 430 | Pyrazon | -2.87 | -3.11 | -3.18 | 0.24 |
| 431 | Haloperidol | -4.43 | -4.67 | -4.58 | 0.24 |
| 432 | Pinacolone | -0.72 | -0.96 | -0.99 | 0.24 |
| 433 | Isooctane | -4.74 | -4.50 | -4.46 | -0.24 |
| 434 | 6-Methyluracil | -1.26 | -1.50 | -1.53 | 0.24 |
| 435 | Glycine | 0.52 | 0.76 | 0.88 | -0.24 |
| 436 | Methylparathion | -3.68 | -3.44 | -3.45 | -0.24 |
| 437 | s-Butylbenzene | -3.89 | -3.65 | -3.70 | -0.24 |
| 438 | Methapyrilene | -2.64 | -2.40 | -2.10 | -0.24 |
| 439 | Barbital | -1.39 | -1.63 | -1.71 | 0.24 |
| 440 | Niflumic acid | -4.17 | -4.41 | -4.41 | 0.24 |
| 441 | Molecule601 | -4.21 | -4.45 | -4.36 | 0.24 |
| 442 | Prednisolone | -3.21 | -3.45 | -3.54 | 0.24 |
| 443 | Molecule442 | -4.11 | -4.35 | -4.56 | 0.24 |
| 444 | p-Dibromobenzene | -4.07 | -3.82 | -3.89 | -0.25 |
| 445 | Iodobenzene | -2.78 | -2.53 | -2.50 | -0.25 |
| 446 | Phenytoin | -3.99 | -3.74 | -3.67 | -0.25 |
| 447 | 4-t-Butylphenol | -2.41 | -2.16 | -2.12 | -0.25 |
| 448 | Molecule661 | -3.36 | -3.61 | -3.79 | 0.25 |
| 449 | Methylbenzene | -2.21 | -2.46 | -2.45 | 0.25 |
| 450 | Ethyl acetate | -0.04 | -0.29 | -0.29 | 0.25 |
| 451 | Phenylalanine | -1.23 | -1.48 | -1.50 | 0.25 |
| 452 | Triethylamine | -0.14 | 0.11 | 0.04 | -0.25 |
| 453 | 4-Anisaldehyde | -1.49 | -1.24 | -1.22 | -0.25 |
| 454 | 1-Bromopropane | -1.73 | -1.48 | -1.52 | -0.25 |
| 455 | Amygdalin | -0.77 | -1.02 | -1.10 | 0.25 |
| 456 | Camphoric acid | -1.42 | -1.17 | -1.19 | -0.25 |
| 457 | 2,4,6-Trinitrotoluene | -3.22 | -3.47 | -3.46 | 0.25 |
| 458 | 17-Methyltestosterone | -3.99 | -4.24 | -4.17 | 0.25 |
| 459 | 1,4,5-Trimethylnaphthalene | -4.92 | -5.18 | -5.10 | 0.26 |
| 460 | Carvacrol | -2.08 | -2.34 | -2.39 | 0.26 |
| 461 | Benzoin | -2.85 | -3.11 | -3.02 | 0.26 |
| 462 | Methyl benzoate | -1.85 | -1.59 | -1.56 | -0.26 |
| 463 | 2-Methyl-5-(1-methylethenyl)cyclohexanone | -2.18 | -2.44 | -2.42 | 0.26 |
| 464 | Ethylenedichloride | -1.06 | -0.80 | -0.73 | -0.26 |
| 465 | Hemimellitene | -3.20 | -3.46 | -3.54 | 0.26 |
| 466 | 2,2',5,6'-Tetrachlorobiphenyl | -6.80 | -6.54 | -6.48 | -0.26 |
| 467 | 4-Pyrimidone | 0.59 | 0.33 | 0.28 | 0.26 |
| 468 | Hexobarbital | -2.74 | -2.48 | -2.36 | -0.26 |
| 469 | Butyraldehyde | -0.01 | -0.27 | -0.28 | 0.26 |
| 470 | n-Valeraldehyde | -0.85 | -0.59 | -0.58 | -0.26 |
| 471 | cis-1,2-Dichloroethylene | -1.30 | -1.56 | -1.89 | 0.26 |
| 472 | Trichloroethylene | -1.96 | -2.22 | -2.17 | 0.26 |
| 473 | Molecule758 | -4.68 | -4.94 | -5.09 | 0.26 |
| 474 | Molecule580 | -4.05 | -4.31 | -4.50 | 0.26 |
| 475 | 2,6-Dichlorobiphenyl | -5.21 | -4.94 | -4.93 | -0.27 |
| 476 | 2,3,4-Trimethylpentane | -4.80 | -4.53 | -4.52 | -0.27 |
| 477 | Carbaryl | -3.28 | -3.01 | -2.95 | -0.27 |
| 478 | 2,3',5-Trichlorobiphenyl | -6.01 | -5.74 | -5.70 | -0.27 |
| 479 | Menthone | -2.49 | -2.22 | -2.21 | -0.27 |
| 480 | Molecule639 | -3.93 | -3.66 | -3.53 | -0.27 |
| 481 | 4-Ethyltoluene | -3.11 | -3.38 | -3.40 | 0.27 |
| 482 | Molecule440 | -2.08 | -1.81 | -1.27 | -0.27 |
| 483 | Ethyl-n-propylether | -0.66 | -0.39 | -0.39 | -0.27 |
| 484 | Monocrotophos | 0.65 | 0.38 | 0.14 | 0.27 |
| 485 | Pyrrole | -0.17 | -0.44 | -0.55 | 0.27 |
| 486 | Hexamethylbenzene | -5.23 | -4.96 | -4.91 | -0.27 |
| 487 | Ethyl vinyl ether | -0.85 | -0.58 | -0.48 | -0.27 |
| 488 | 2-Chloroanisole | -2.46 | -2.19 | -2.13 | -0.27 |
| 489 | Flutriafol | -3.37 | -3.64 | -3.57 | 0.27 |
| 490 | Hexachlorocyclopentadiene | -5.18 | -5.45 | -5.51 | 0.27 |
| 491 | Pacazine | -4.74 | -4.47 | -4.36 | -0.27 |
| 492 | Molecule1386 | -4.95 | -4.68 | -4.49 | -0.27 |
| 493 | Fenarimol | -4.38 | -4.65 | -4.63 | 0.27 |
| 494 | 2,2',3,3',4,5-Hexachlorobiphenyl | -8.42 | -8.14 | -8.07 | -0.28 |
| 495 | 2,2',5-Trichlorobiphenyl | -6.02 | -5.74 | -5.64 | -0.28 |
| 496 | p-Menthadiene | -4.00 | -3.72 | -3.68 | -0.28 |
| 497 | 2-Chlorophenoxyacetic acid | -2.16 | -1.88 | -1.89 | -0.28 |
| 498 | 3-Methyl-3-heptanol | -1.60 | -1.32 | -1.28 | -0.28 |
| 499 | Gentisin | -2.93 | -3.21 | -3.25 | 0.28 |
| 500 | Neohexane | -3.55 | -3.27 | -3.29 | -0.28 |
| 501 | 2-Octanone | -2.05 | -1.77 | -1.74 | -0.28 |
| 502 | Chloroacetonitrile | -0.09 | -0.37 | -0.36 | 0.28 |
| 503 | Methylpropylether | -0.39 | -0.11 | -0.14 | -0.28 |
| 504 | Methyl acrylate | -0.22 | -0.50 | -0.51 | 0.28 |
| 505 | 2-Nitrophenol | -1.74 | -2.02 | -1.99 | 0.28 |
| 506 | Paracetamol | -1.03 | -1.31 | -1.27 | 0.28 |
| 507 | 1,5-Hexadiene | -2.68 | -2.40 | -2.42 | -0.28 |
| 508 | 2,2',3,3',4,4',5,5',6,6'-Decachlorobiphenyl | -11.62 | -11.34 | -11.31 | -0.28 |
| 509 | 1,3-Dibromobenzene | -3.54 | -3.82 | -3.82 | 0.28 |
| 510 | 2,2',3,3',4,4',5,5',6-Nonachlorobiphenyl | -10.26 | -10.54 | -10.48 | 0.28 |
| 511 | Pentachlorobenzene | -5.65 | -5.36 | -5.31 | -0.29 |
| 512 | Herban | -3.01 | -2.72 | -2.63 | -0.29 |
| 513 | 2,2',3,3',4-Pentachlorobiphenyl | -7.05 | -7.34 | -7.35 | 0.29 |
| 514 | 2,3,3',4,4'-Pentachlorobiphenyl | -7.05 | -7.34 | -7.28 | 0.29 |
| 515 | Metolcarb | -1.80 | -2.09 | -2.04 | 0.29 |
| 516 | 1-Nitronaphthalene | -3.54 | -3.25 | -3.27 | -0.29 |
| 517 | 2-Nitroaniline | -1.96 | -2.25 | -2.25 | 0.29 |
| 518 | Molecule635 | -3.04 | -2.75 | -2.68 | -0.29 |
| 519 | Ethyl formate | 0.15 | -0.14 | -0.22 | 0.29 |
| 520 | Pentyl propanoate | -1.28 | -1.57 | -1.60 | 0.29 |
| 521 | Cyclooctanol | -1.29 | -1.58 | -1.54 | 0.29 |
| 522 | 1,2,3,5-Tetrafluorobenzene | -2.31 | -2.60 | -2.50 | 0.29 |
| 523 | 3,5-Diiodosalicylic acid | -3.31 | -3.60 | -3.73 | 0.29 |
| 524 | 2,6-Diethylaniline | -2.35 | -2.64 | -2.65 | 0.29 |
| 525 | 4-Fluoroiodobenzene | -3.13 | -2.84 | -2.85 | -0.29 |
| 526 | Aminocarb | -2.36 | -2.07 | -2.05 | -0.29 |
| 527 | Coumachlor | -5.84 | -5.55 | -5.38 | -0.29 |
| 528 | Molecule1193 | -5.18 | -4.89 | -4.84 | -0.29 |
| 529 | Cholanthrene | -7.85 | -8.14 | -8.19 | 0.29 |
| 530 | Propyl 4-aminobenzoate | -2.33 | -2.03 | -2.11 | -0.30 |
| 531 | 3,3'-Dichlorobenzidine | -4.92 | -4.62 | -4.53 | -0.30 |
| 532 | Trichloroacetonitrile | -2.17 | -1.87 | -1.80 | -0.30 |
| 533 | 1,1,2,2-Tetrachloroethane | -1.74 | -2.04 | -2.08 | 0.30 |
| 534 | Isobutyl formate | -1.01 | -0.71 | -0.71 | -0.30 |
| 535 | Chlorpromazine | -5.01 | -4.71 | -4.64 | -0.30 |
| 536 | 3-Methyl-3-pentanol | -0.38 | -0.68 | -0.66 | 0.30 |
| 537 | Fensulfothion | -2.30 | -2.60 |  | 0.30 |
| 538 | 3-Hexanone | -0.83 | -1.13 | -1.15 | 0.30 |
| 539 | Lindane | -4.60 | -4.90 | -4.91 | 0.30 |
| 540 | Nifedipine | -4.76 | -5.06 | -5.09 | 0.30 |
| 541 | 2,4-Dichlorobiphenyl | -5.25 | -4.94 | -4.90 | -0.31 |
| 542 | 2,4-Dimethylpentane | -4.26 | -3.95 | -3.94 | -0.31 |
| 543 | Cyclohexanecarboxylic acid | -1.45 | -1.14 | -1.15 | -0.31 |
| 544 | 3-Methylpentane | -3.68 | -3.37 | -3.33 | -0.31 |
| 545 | Isopentyl salicylate | -3.16 | -2.85 | -2.87 | -0.31 |
| 546 | Molecule1144 | -1.90 | -1.59 | -1.66 | -0.31 |
| 547 | cis-4-Methylcyclohexanol | -0.88 | -1.19 | -1.20 | 0.31 |
| 548 | s-cis-Butadiene | -1.87 | -1.56 | -1.48 | -0.31 |
| 549 | n-Butyl acetate | -1.24 | -0.93 | -0.89 | -0.31 |
| 550 | 2-Pentanol | -0.29 | -0.60 | -0.59 | 0.31 |
| 551 | 2-Heptanol | -1.55 | -1.24 | -1.19 | -0.31 |
| 552 | Thiophene | -1.45 | -1.76 | -1.89 | 0.31 |
| 553 | Glyceryltriacetate | -0.60 | -0.91 | -1.00 | 0.31 |
| 554 | 1,4-Dichlorobenzene | -3.27 | -2.96 | -2.91 | -0.31 |
| 555 | Molecule1473 | -0.39 | -0.70 | -0.71 | 0.31 |
| 556 | 3,3-Dimethylpentane | -4.23 | -3.92 | -3.92 | -0.31 |
| 557 | 3-Aminoacetophenone | -1.28 | -1.59 | -1.59 | 0.31 |
| 558 | Prochlorperazine | -4.40 | -4.09 | -3.95 | -0.31 |
| 559 | Hyoscyamine | -1.91 | -2.22 | -2.21 | 0.31 |
| 560 | Molecule1170 | -5.28 | -4.97 | -4.91 | -0.31 |
| 561 | 2-Ethylnaphthalene | -4.29 | -4.60 | -4.68 | 0.31 |
| 562 | 4-Allyl anisole | -2.92 | -2.60 | -2.61 | -0.32 |
| 563 | Molecule802 | -1.65 | -1.33 | -1.33 | -0.32 |
| 564 | Pentachloroethane | -2.60 | -2.92 | -3.01 | 0.32 |
| 565 | Benzene | -1.64 | -1.96 | -2.01 | 0.32 |
| 566 | Acetonitrile | 0.26 | -0.06 | -0.13 | 0.32 |
| 567 | p-Benzidine | -2.70 | -3.02 | -3.15 | 0.32 |
| 568 | (Chloromethyl)benzene | -2.39 | -2.07 | -2.04 | -0.32 |
| 569 | Cidoxepin | -3.40 | -3.72 | -3.66 | 0.32 |
| 570 | 1,2-Dibromobenzene | -3.50 | -3.82 | -3.82 | 0.32 |
| 571 | 2,3,3',4,4',5-Hexachlorobiphenyl | -7.82 | -8.14 | -8.13 | 0.32 |
| 572 | 2,2'-Dichlorobiphenyl | -5.27 | -4.94 | -4.93 | -0.33 |
| 573 | 2,3-Dimethylpentane | -4.28 | -3.95 | -3.94 | -0.33 |
| 574 | Benzo[k]fluoranthene | -8.49 | -8.16 | -8.08 | -0.33 |
| 575 | Molecule625 | -3.97 | -3.64 | -3.59 | -0.33 |
| 576 | Probarbital | -2.21 | -1.88 | -1.95 | -0.33 |
| 577 | Nimetazepam | -3.80 | -4.13 | -4.08 | 0.33 |
| 578 | Phlorol | -1.36 | -1.69 | -1.74 | 0.33 |
| 579 | 3-Methylthiophene | -2.39 | -2.06 | -1.89 | -0.33 |
| 580 | Piperonal | -1.63 | -1.30 | -1.21 | -0.33 |
| 581 | Leucine | -0.80 | -0.47 | -0.42 | -0.33 |
| 582 | Diethylsulfone | 0.04 | -0.29 | -0.58 | 0.33 |
| 583 | Ethohexadiol | -0.54 | -0.87 | -0.93 | 0.33 |
| 584 | Butylcellosolve | -0.42 | -0.09 | -0.12 | -0.33 |
| 585 | 3-Methyl-2-butanol | -0.20 | -0.53 | -0.55 | 0.33 |
| 586 | 4-Amino-3,5,6-trichloro-2-pyridinecarboxlic acid | -2.75 | -2.42 | -2.20 | -0.33 |
| 587 | 2-Hexanone | -0.80 | -1.13 | -1.12 | 0.33 |
| 588 | Carvone | -2.06 | -2.39 | -2.37 | 0.33 |
| 589 | Metoxuron | -2.56 | -2.89 | -2.83 | 0.33 |
| 590 | 3,4-Dichloronitrobenzene | -3.20 | -3.53 | -3.54 | 0.33 |
| 591 | 2,4'-Dichlorobiphenyl | -5.28 | -4.94 | -4.95 | -0.34 |
| 592 | Ethinyl Estradiol | -4.30 | -4.64 | -4.78 | 0.34 |
| 593 | Nitrazepam | -3.80 | -4.14 | -3.91 | 0.34 |
| 594 | Iprodione | -4.38 | -4.72 | -5.14 | 0.34 |
| 595 | Butylbenzene | -4.06 | -3.72 | -3.66 | -0.34 |
| 596 | Heptachlor epoxide | -6.29 | -5.95 | -5.93 | -0.34 |
| 597 | Tetrachloroethylene | -2.54 | -2.88 | -2.83 | 0.34 |
| 598 | Imipramine | -4.19 | -3.85 | -3.79 | -0.34 |
| 599 | 2-Chloropropane | -1.41 | -1.07 | -1.09 | -0.34 |
| 600 | 2-Methyl-1-pentanol | -1.11 | -0.77 | -0.79 | -0.34 |
| 601 | 4-Chloroacetanilide | -2.84 | -2.50 | -2.46 | -0.34 |
| 602 | 4-Chloroaniline | -1.66 | -2.00 | -2.01 | 0.34 |
| 603 | 2,3-Xylenol | -1.43 | -1.77 | -1.73 | 0.34 |
| 604 | 4-Methylbenzylalcohol | -1.20 | -1.54 | -1.51 | 0.34 |
| 605 | Fenfuram | -3.30 | -2.96 | -2.87 | -0.34 |
| 606 | 2-Phenoxyethanol | -0.71 | -1.05 | -1.04 | 0.34 |
| 607 | Di-n-butylsebacate | -3.90 | -4.24 | -4.28 | 0.34 |
| 608 | Amitraz | -5.47 | -5.81 | -6.13 | 0.34 |
| 609 | 2,4-DB | -3.67 | -3.32 | -3.30 | -0.35 |
| 610 | 2,3-Dimethylbutane | -3.65 | -3.30 | -3.32 | -0.35 |
| 611 | Molecule1089 | -2.05 | -2.40 | -2.41 | 0.35 |
| 612 | Ethyl butanoate | -1.28 | -0.93 | -0.93 | -0.35 |
| 613 | Isobutyl acetate | -1.21 | -0.86 | -0.85 | -0.35 |
| 614 | Trietazine | -4.06 | -3.71 | -3.86 | -0.35 |
| 615 | 1-Iodopropane | -2.29 | -1.94 | -1.95 | -0.35 |
| 616 | Terbacil | -2.48 | -2.83 | -2.94 | 0.35 |
| 617 | cis-1,2-Dimethylcyclohexane | -4.27 | -4.62 | -4.59 | 0.35 |
| 618 | Molecule669 | -2.62 | -2.97 | -2.92 | 0.35 |
| 619 | Atovaquone | -5.93 | -6.28 | -6.16 | 0.35 |
| 620 | Phenacetin | -2.37 | -2.01 | -1.99 | -0.36 |
| 621 | Metiazinic acid | -3.94 | -4.30 | -4.25 | 0.36 |
| 622 | Indane | -3.04 | -3.40 | -3.45 | 0.36 |
| 623 | Oxadiazon | -5.69 | -5.33 | -5.00 | -0.36 |
| 624 | Methyl isobutyl ketone | -1.42 | -1.06 | -1.08 | -0.36 |
| 625 | Opianic acid | -1.92 | -1.56 | -1.56 | -0.36 |
| 626 | Diethyl disulfide | -2.42 | -2.06 |  | -0.36 |
| 627 | Cyclohexane | -3.10 | -3.46 | -3.39 | 0.36 |
| 628 | 3-Pentanol | -0.24 | -0.60 | -0.59 | 0.36 |
| 629 | Molecule1197 | -1.63 | -1.27 | -1.32 | -0.36 |
| 630 | Coumarin | -1.89 | -2.25 | -2.31 | 0.36 |
| 631 | Molecule917 | -4.61 | -4.25 | -4.09 | -0.36 |
| 632 | 3,4-Xylenol | -1.41 | -1.77 | -1.78 | 0.36 |
| 633 | Molecule1055 | -1.91 | -2.27 | -2.28 | 0.36 |
| 634 | Estrone | -3.96 | -4.32 | -4.30 | 0.36 |
| 635 | Thiopental | -3.36 | -2.99 | -2.95 | -0.37 |
| 636 | 2-Methylpentane | -3.74 | -3.37 | -3.33 | -0.37 |
| 637 | Acetanilide | -1.33 | -1.70 | -1.69 | 0.37 |
| 638 | 2,2-Dimethyl-1-butanol | -1.04 | -0.67 | -0.68 | -0.37 |
| 639 | Salicin | -0.85 | -1.22 | -1.23 | 0.37 |
| 640 | 4-Methyl-1-pentanol | -1.14 | -0.77 | -0.77 | -0.37 |
| 641 | Tripelennamine | -2.64 | -2.27 | -2.15 | -0.37 |
| 642 | 1,4-Benzoquinone | -0.99 | -0.62 | -0.57 | -0.37 |
| 643 | 3,5-Xylenol | -1.40 | -1.77 | -1.77 | 0.37 |
| 644 | Molecule788 | -0.53 | -0.90 | -0.94 | 0.37 |
| 645 | Sufentanil | -3.71 | -4.08 | -4.14 | 0.37 |
| 646 | Molecule806 | -2.98 | -2.61 | -2.55 | -0.37 |
| 647 | Steroid01 | -3.85 | -3.47 | -3.51 | -0.38 |
| 648 | 4-Iodoacetanilide | -3.25 | -2.87 | -2.79 | -0.38 |
| 649 | 3,5-Dibromo-4-hydroxybenzonitrile | -3.33 | -3.71 | -3.81 | 0.38 |
| 650 | 1,3-Dibromopropane | -2.08 | -2.46 | -2.55 | 0.38 |
| 651 | Cycloheptanol | -0.88 | -1.26 | -1.23 | 0.38 |
| 652 | Allyl chloride | -1.36 | -0.98 | -0.95 | -0.38 |
| 653 | Tetrahydropyran | -0.03 | -0.41 | -0.35 | 0.38 |
| 654 | 2-Ethylthiophene | -2.59 | -2.21 | -1.85 | -0.38 |
| 655 | Benzoic acid | -1.55 | -1.17 | -1.11 | -0.38 |
| 656 | Nitrapyrin | -3.76 | -3.38 | -3.40 | -0.38 |
| 657 | Triphenylene | -6.74 | -7.12 | -7.12 | 0.38 |
| 658 | Endrin | -6.29 | -5.90 | -5.81 | -0.39 |
| 659 | Diethion | -5.54 | -5.15 | -5.08 | -0.39 |
| 660 | 1,3-Dimethylnaphthalene | -4.29 | -4.68 | -4.64 | 0.39 |
| 661 | Thiometon | -3.09 | -2.70 | -2.85 | -0.39 |
| 662 | Bromophos | -6.09 | -5.70 | -5.75 | -0.39 |
| 663 | Strychnine | -3.32 | -2.93 | -2.98 | -0.39 |
| 664 | Promazine | -4.30 | -3.91 | -3.81 | -0.39 |
| 665 | Pentane | -3.18 | -2.79 | -2.78 | -0.39 |
| 666 | Caproic aldehyde | -1.30 | -0.91 | -0.90 | -0.39 |
| 667 | 2-Propanol | 0.43 | 0.04 | 0.01 | 0.39 |
| 668 | Methacrylic acid | 0.01 | -0.38 | -0.37 | 0.39 |
| 669 | Methylvalerate | -1.36 | -0.97 | -1.00 | -0.39 |
| 670 | 2-Bromopropane | -1.59 | -1.20 |  | -0.39 |
| 671 | 3-Methyl-2-pentanone | -0.67 | -1.06 | -1.08 | 0.39 |
| 672 | p-Toluidine | -1.21 | -1.60 | -1.57 | 0.39 |
| 673 | 2-Iodobenzoic acid | -2.73 | -2.34 | -2.34 | -0.39 |
| 674 | beta-HCH | -4.51 | -4.90 | -5.19 | 0.39 |
| 675 | Dienochlor | -7.28 | -7.68 | -9.38 | 0.40 |
| 676 | Perphenazine | -4.16 | -3.76 | -3.76 | -0.40 |
| 677 | 2,4',6-Trichlorobiphenyl | -6.14 | -5.74 | -5.78 | -0.40 |
| 678 | 2,4,6-Trichlorobiphenyl | -6.14 | -5.74 | -5.64 | -0.40 |
| 679 | Benzilic acid | -2.21 | -2.61 | -2.60 | 0.40 |
| 680 | 2-Methyl-2-heptanol | -1.72 | -1.32 | -1.32 | -0.40 |
| 681 | 2-Ethyl-1-butanol | -1.17 | -0.77 | -0.75 | -0.40 |
| 682 | Hydrocinnamyl alcohol | -1.38 | -1.78 | -1.81 | 0.40 |
| 683 | 1-Hexanol | -1.24 | -0.84 | -0.79 | -0.40 |
| 684 | Hexane | -3.84 | -3.44 | -3.44 | -0.40 |
| 685 | 3-phenylpropionic acid | -1.41 | -1.81 | -1.76 | 0.40 |
| 686 | 3-Chloropropanenitrile | -0.29 | -0.69 | -0.72 | 0.40 |
| 687 | Fenpropathrin | -6.03 | -5.63 | -5.66 | -0.40 |
| 688 | Molecule529 | -5.03 | -4.63 | -4.49 | -0.40 |
| 689 | 1-Heptyne | -3.01 | -2.61 | -2.67 | -0.40 |
| 690 | Promethazine | -4.26 | -3.86 | -3.79 | -0.40 |
| 691 | Molecule668 | -2.86 | -3.26 | -3.43 | 0.40 |
| 692 | 2-Chlorobiphenyl | -4.54 | -4.14 | -4.08 | -0.40 |
| 693 | 2-Methylnaphthalene | -3.77 | -4.18 | -4.26 | 0.41 |
| 694 | Anilofos | -4.43 | -4.84 | -4.87 | 0.41 |
| 695 | 4-CPA | -2.29 | -1.88 | -1.83 | -0.41 |
| 696 | Hydroquinone | -0.17 | -0.58 | -0.61 | 0.41 |
| 697 | 2-Methylheptane | -5.08 | -4.67 | -4.66 | -0.41 |
| 698 | tau-Fluvalinate | -8.00 | -8.41 | -8.59 | 0.41 |
| 699 | Molecule1174 | -4.79 | -5.20 | -5.04 | 0.41 |
| 700 | Desmedipham | -4.63 | -4.22 | -4.05 | -0.41 |
| 701 | Carbophenothion | -5.74 | -6.15 | -6.45 | 0.41 |
| 702 | Flufenamic acid | -4.36 | -4.77 | -4.79 | 0.41 |
| 703 | 2,2',4,4',5,5'-Hexachlorobiphenyl | -8.56 | -8.14 | -8.09 | -0.42 |
| 704 | 1-Methylfluorene | -5.22 | -5.64 | -5.67 | 0.42 |
| 705 | Molecule1284 | -4.63 | -5.05 | -5.06 | 0.42 |
| 706 | 3-Octanol | -1.98 | -1.56 | -1.52 | -0.42 |
| 707 | 2-Undecanol | -2.94 | -2.52 | -2.45 | -0.42 |
| 708 | 3-Nitroaniline | -2.19 | -1.77 | -1.68 | -0.42 |
| 709 | 4-Nitroacetanilide | -2.69 | -2.27 | -2.21 | -0.42 |
| 710 | Succinimide | 0.30 | -0.12 | -0.10 | 0.42 |
| 711 | 2-Nitropropane | -0.62 | -1.04 | -1.44 | 0.42 |
| 712 | 4-Methylbiphenyl | -4.62 | -5.04 | -5.00 | 0.42 |
| 713 | beta-D-Fructose | 0.64 | 1.06 | 0.98 | -0.42 |
| 714 | Molecule1138 | 0.64 | 1.06 | 1.06 | -0.42 |
| 715 | 2-Propylpentanoic acid | -1.86 | -1.44 | -1.44 | -0.42 |
| 716 | Ethylene | -0.40 | -0.82 | -0.91 | 0.42 |
| 717 | 1,2-Dihydroxy-9,10-anthracenedione | -2.78 | -3.20 | -3.14 | 0.42 |
| 718 | Molecule1194 | -4.46 | -4.89 | -4.89 | 0.43 |
| 719 | Norflurazon | -4.04 | -4.47 | -4.70 | 0.43 |
| 720 | Butane | -2.57 | -2.14 | -2.15 | -0.43 |
| 721 | Bis(2-chloroethyl) ether | -1.12 | -0.69 | -0.56 | -0.43 |
| 722 | Diethyl sulphide | -1.34 | -0.91 | -0.90 | -0.43 |
| 723 | 4-Acetanisidine | -1.30 | -1.73 | -1.74 | 0.43 |
| 724 | Propazine | -4.43 | -4.00 | -4.08 | -0.43 |
| 725 | Etoposide | -3.57 | -4.00 | -4.13 | 0.43 |
| 726 | Fenothiocarb | -3.93 | -3.50 | -3.28 | -0.43 |
| 727 | D-Quercitol | -0.17 | 0.26 | 0.56 | -0.43 |
| 728 | Thiofanox | -1.62 | -1.19 | -0.88 | -0.43 |
| 729 | Carbetamide | -1.83 | -2.26 | -2.27 | 0.43 |
| 730 | Danazol | -5.51 | -5.94 | -6.34 | 0.43 |
| 731 | 1-Ethylnaphthalene | -4.17 | -4.60 | -4.68 | 0.43 |
| 732 | 3,4-Dichloroaniline | -3.24 | -2.80 | -2.75 | -0.44 |
| 733 | 1-Bromonaphthalene | -4.35 | -3.91 | -3.90 | -0.44 |
| 734 | Pebulate | -3.35 | -2.91 | -2.83 | -0.44 |
| 735 | N,N’-Bianiline | -2.92 | -3.36 | -4.96 | 0.44 |
| 736 | Indole | -1.52 | -1.96 | -2.00 | 0.44 |
| 737 | Formic acid methylester | 0.58 | 0.14 | 0.08 | 0.44 |
| 738 | t-Pentyl alcohol | 0.08 | -0.36 | -0.38 | 0.44 |
| 739 | Pyracarbolid | -2.56 | -3.00 | -3.05 | 0.44 |
| 740 | Isobutyric acid | 0.28 | -0.16 | -0.16 | 0.44 |
| 741 | Molecule702 | -2.15 | -1.71 | -1.53 | -0.44 |
| 742 | 2-Hydroxybenzaldehyde | -0.86 | -1.30 | -1.30 | 0.44 |
| 743 | 2,2-Dimethylpentane | -4.36 | -3.92 | -3.92 | -0.44 |
| 744 | Heptane | -4.53 | -4.09 | -4.05 | -0.44 |
| 745 | 5,6,7,8-Tetrahydro-2-naphthalenol | -1.99 | -2.43 | -2.37 | 0.44 |
| 746 | Ethylcyclohexane | -4.25 | -4.69 | -4.63 | 0.44 |
| 747 | Fluoranthene | -6.00 | -6.44 | -6.57 | 0.44 |
| 748 | Fenchlorphos | -5.72 | -5.27 | -5.23 | -0.45 |
| 749 | Ethyl benzoate | -2.32 | -1.87 | -1.84 | -0.45 |
| 750 | Molecule759 | -5.07 | -4.62 | -4.59 | -0.45 |
| 751 | Xylocaine | -1.76 | -2.21 | -2.28 | 0.45 |
| 752 | Vitamin B3 | 0.61 | 0.16 | 0.08 | 0.45 |
| 753 | Propane | -1.94 | -1.49 | -1.49 | -0.45 |
| 754 | 1-Chloronaphthalene | -3.93 | -3.48 | -3.45 | -0.45 |
| 755 | Molecule1032 | 0.10 | -0.35 | -0.44 | 0.45 |
| 756 | Chlorazine | -4.41 | -3.96 | -3.87 | -0.45 |
| 757 | 4-Nitroanisole | -2.41 | -1.96 | -1.92 | -0.45 |
| 758 | 6-Hydroxyquinoline | -2.16 | -1.71 | -1.67 | -0.45 |
| 759 | Molecule836 | -2.38 | -1.93 |  | -0.45 |
| 760 | Phenylsalicylate | -3.15 | -3.60 | -3.63 | 0.45 |
| 761 | Delmadinone acetate | -4.95 | -5.40 | -5.36 | 0.45 |
| 762 | 2,2',3,3',5,6-Hexachlorobiphenyl | -8.60 | -8.14 | -8.13 | -0.46 |
| 763 | 2,2',3,4',5,5'-Hexachlorobiphenyl | -8.60 | -8.14 | -8.08 | -0.46 |
| 764 | Flurbiprofen | -4.49 | -4.03 | -3.88 | -0.46 |
| 765 | Isopentane | -3.18 | -2.72 | -2.70 | -0.46 |
| 766 | Propylthiouracil | -2.15 | -2.61 | -2.62 | 0.46 |
| 767 | Anisole | -1.85 | -1.39 | -1.41 | -0.46 |
| 768 | n-Heptylamine | -1.85 | -1.39 | -1.51 | -0.46 |
| 769 | Molecule554 | -4.71 | -5.17 | -5.28 | 0.46 |
| 770 | 2,2',5,5'-Tetrachlorobiphenyl | -7.00 | -6.54 | -6.48 | -0.46 |
| 771 | Pentamethylbenzene | -4.00 | -4.46 | -4.49 | 0.46 |
| 772 | Permethrin | -6.29 | -6.75 | -6.81 | 0.46 |
| 773 | 2,6-Xylenol | -1.31 | -1.77 | -1.73 | 0.46 |
| 774 | Methylthiouracil | -2.43 | -1.97 | -1.96 | -0.46 |
| 775 | Fenuron | -1.60 | -2.06 | -2.02 | 0.46 |
| 776 | Phthalonitrile | -2.38 | -1.92 | -1.75 | -0.46 |
| 777 | Noscapine | -3.14 | -3.60 | -3.70 | 0.46 |
| 778 | Molecule946 | -3.84 | -3.38 | -3.39 | -0.46 |
| 779 | 1,2-Dibromoethane | -1.68 | -2.14 | -2.41 | 0.46 |
| 780 | 2-Methylcyclohexanone | -0.94 | -1.40 | -1.38 | 0.46 |
| 781 | 2,2',3,4,4'-Pentachlorobiphenyl | -7.80 | -7.34 | -7.32 | -0.46 |
| 782 | Phenmedipham | -4.80 | -4.34 | -4.33 | -0.46 |
| 783 | 2,2',3,4,5,5'-Hexachlorobiphenyl | -7.68 | -8.14 | -8.13 | 0.46 |
| 784 | 2,4,4'-Trichlorobiphenyl | -6.21 | -5.74 | -5.70 | -0.47 |
| 785 | 3-Methylcyclohexanone | -1.87 | -1.40 | -1.37 | -0.47 |
| 786 | 1,3-Dichloro-2-propanol | -0.11 | -0.58 | -0.63 | 0.47 |
| 787 | Cytosine | -1.14 | -0.67 | -0.50 | -0.47 |
| 788 | Molecule1158 | -0.74 | -1.21 | -1.43 | 0.47 |
| 789 | Ethyne | 0.29 | -0.18 | -0.40 | 0.47 |
| 790 | Acetophenone | -1.28 | -1.75 | -1.79 | 0.47 |
| 791 | n-Heptaldehyde | -1.70 | -1.23 | -1.17 | -0.47 |
| 792 | Piperazine | 1.07 | 1.54 | 1.71 | -0.47 |
| 793 | Molecule1211 | -0.83 | -1.30 | -1.28 | 0.47 |
| 794 | Glutethimide | -2.34 | -2.81 | -2.77 | 0.47 |
| 795 | Isobutylbenzene | -4.12 | -3.65 | -3.66 | -0.47 |
| 796 | Molecule27 | -3.12 | -2.65 |  | -0.47 |
| 797 | Osthole | -4.31 | -3.84 | -3.87 | -0.47 |
| 798 | 17a-Hydroxyprogesterone | -4.71 | -4.23 | -4.24 | -0.48 |
| 799 | 1-Methylnaphthalene | -3.70 | -4.18 | -4.15 | 0.48 |
| 800 | PKKB04 | -3.19 | -3.67 | -3.77 | 0.48 |
| 801 | 2-Chloroaniline | -1.52 | -2.00 | -1.98 | 0.48 |
| 802 | n-Propyl benzoate | -2.67 | -2.19 | -2.16 | -0.48 |
| 803 | Nitroethane | -0.22 | -0.70 | -0.46 | 0.48 |
| 804 | 1H-Indazole | -2.16 | -2.64 |  | 0.48 |
| 805 | 2-Methylpropane | -2.55 | -2.07 | -2.09 | -0.48 |
| 806 | 2,3,3',4,4',6-Hexachlorobiphenyl | -7.66 | -8.14 | -8.08 | 0.48 |
| 807 | Oxyphenbutazone | -3.73 | -3.24 | -3.17 | -0.49 |
| 808 | 2-Bromonaphthalene | -4.40 | -3.91 | -3.76 | -0.49 |
| 809 | Isoborneol | -2.32 | -1.83 | -1.85 | -0.49 |
| 810 | o-Toluic acid | -2.06 | -1.57 | -1.55 | -0.49 |
| 811 | Furfural | -0.10 | -0.59 | -0.86 | 0.49 |
| 812 | Isopentyl formate | -1.52 | -1.03 | -0.95 | -0.49 |
| 813 | 2-Hydroxybenzamide | -1.76 | -1.27 | -1.19 | -0.49 |
| 814 | Crotonaldehyde | 0.32 | -0.17 | -0.22 | 0.49 |
| 815 | Molecule761 | -4.47 | -3.98 | -3.82 | -0.49 |
| 816 | 5-Methylcytosine | -1.46 | -0.97 | -0.90 | -0.49 |
| 817 | 3-Methylheptane | -5.16 | -4.67 | -4.63 | -0.49 |
| 818 | Hydrocinchonine | -2.63 | -3.12 | -3.15 | 0.49 |
| 819 | Molecule1221 | -0.88 | -1.37 | -1.44 | 0.49 |
| 820 | 2-Nonanone | -2.58 | -2.09 | -2.10 | -0.49 |
| 821 | Diphenamid | -2.98 | -3.47 | -3.48 | 0.49 |
| 822 | Molecule1343 | -4.00 | -4.49 | -4.53 | 0.49 |
| 823 | Nonane | -5.88 | -5.39 | -5.26 | -0.49 |
| 824 | Steroid17 | -4.30 | -3.80 | -3.73 | -0.50 |
| 825 | Octane | -5.24 | -4.74 | -4.67 | -0.50 |
| 826 | Dinoseb | -3.38 | -3.88 | -3.97 | 0.50 |
| 827 | 1,3-Dichloropropane | -1.62 | -1.12 | -1.15 | -0.50 |
| 828 | Di-n-butylether | -1.85 | -1.35 | -1.36 | -0.50 |
| 829 | Cinmetacin | -5.54 | -5.04 | -4.97 | -0.50 |
| 830 | Ethyl valerate | -1.75 | -1.25 | -1.28 | -0.50 |
| 831 | 1-Propanol | 0.62 | 0.12 | 0.11 | 0.50 |
| 832 | Isoniazid | 0.01 | 0.51 |  | -0.50 |
| 833 | Cyclohexanol | -0.44 | -0.94 | -0.95 | 0.50 |
| 834 | 1-Hexen-3-ol | -0.59 | -1.09 | -1.13 | 0.50 |
| 835 | Molecule633 | -1.87 | -2.37 | -2.71 | 0.50 |
| 836 | 5-Nonanone | -2.59 | -2.09 | -2.11 | -0.50 |
| 837 | Aspirin | -1.72 | -2.22 | -2.10 | 0.50 |
| 838 | Molecule366 | -6.36 | -6.86 | -6.95 | 0.50 |
| 839 | Amylbenzene | -4.64 | -4.14 | -4.18 | -0.50 |
| 840 | 2,2',3,3',6,6'-Hexachlorobiphenyl | -8.65 | -8.14 | -8.15 | -0.51 |
| 841 | 2,2',3,4',5',6-Hexachlorobiphenyl | -8.65 | -8.14 | -8.10 | -0.51 |
| 842 | Dibenzo[b,e]pyridine | -3.67 | -4.18 | -4.08 | 0.51 |
| 843 | Isoproturon | -3.54 | -3.03 | -3.00 | -0.51 |
| 844 | 2,4',5-Trichlorobiphenyl | -6.25 | -5.74 | -5.73 | -0.51 |
| 845 | Triptane | -4.36 | -3.85 | -3.82 | -0.51 |
| 846 | cis-2,5-Dimethylpiperazine | 0.49 | 1.00 | 1.29 | -0.51 |
| 847 | 5-Allyl-5-ethylbarbituric acid | -1.61 | -2.12 | -2.13 | 0.51 |
| 848 | Ancymidol | -2.60 | -2.09 | -1.79 | -0.51 |
| 849 | Progesterone | -4.43 | -4.94 | -4.89 | 0.51 |
| 850 | Menthol | -2.53 | -2.01 | -2.02 | -0.52 |
| 851 | 2,3,4'-Trichlorobiphenyl | -6.26 | -5.74 | -5.73 | -0.52 |
| 852 | Chlorothalonil | -5.64 | -5.12 | -5.03 | -0.52 |
| 853 | 4-Chlorotoluene | -3.08 | -2.56 | -2.50 | -0.52 |
| 854 | 2,4,5-T | -2.96 | -3.48 | -3.48 | 0.52 |
| 855 | Ethane | -1.36 | -0.84 | -0.84 | -0.52 |
| 856 | Propyne | -0.41 | -0.93 | -1.13 | 0.52 |
| 857 | Molecule1131 | -2.94 | -2.42 | -2.18 | -0.52 |
| 858 | Phthalic anhydride | -1.39 | -1.91 | -1.83 | 0.52 |
| 859 | 1,2-Diethylbenzene | -3.28 | -3.80 | -3.81 | 0.52 |
| 860 | 2,4,5-Trichlorobiphenyl | -6.27 | -5.74 | -5.68 | -0.53 |
| 861 | 3-Bromotoluene | -3.52 | -2.99 | -2.98 | -0.53 |
| 862 | 4-Phenylphenol | -3.48 | -2.95 | -2.91 | -0.53 |
| 863 | Molecule1589 | -2.36 | -1.83 | -1.25 | -0.53 |
| 864 | 2-Octanol | -2.09 | -1.56 | -1.50 | -0.53 |
| 865 | Di-n-butylamine | -1.44 | -0.91 | -0.86 | -0.53 |
| 866 | Propanal | 0.58 | 0.05 | 0.01 | 0.53 |
| 867 | Methyl acetate | 0.52 | -0.01 | -0.08 | 0.53 |
| 868 | Molecule1441 | -1.52 | -0.99 | -1.04 | -0.53 |
| 869 | 3-Hexyne | -1.99 | -2.52 | -2.85 | 0.53 |
| 870 | Molecule777 | 0.74 | 1.27 | 1.30 | -0.53 |
| 871 | Stanolone | -4.74 | -4.21 | -4.18 | -0.53 |
| 872 | 3-Nitrophenol | -1.01 | -1.54 | -1.49 | 0.53 |
| 873 | Diethylketone | -0.28 | -0.81 | -0.84 | 0.53 |
| 874 | Bisphenol A | -2.82 | -3.35 | -3.31 | 0.53 |
| 875 | Dichlorophene | -3.95 | -4.48 | -4.51 | 0.53 |
| 876 | 1,4-Dimethylnaphthalene | -4.14 | -4.68 | -4.69 | 0.54 |
| 877 | Hydrastine | -4.11 | -3.57 | -3.50 | -0.54 |
| 878 | Tefluthrin | -7.32 | -6.78 | -6.66 | -0.54 |
| 879 | Cyclopentene | -2.10 | -1.56 | -1.61 | -0.54 |
| 880 | 1,6-Hexanedioic acid | -0.82 | -0.28 | -0.27 | -0.54 |
| 881 | Dimethyl carbate | -1.20 | -1.74 | -1.76 | 0.54 |
| 882 | 5-Methyluracil | -1.52 | -0.98 | -0.99 | -0.54 |
| 883 | a-D-Arabinopyranose | 0.39 | 0.93 | 0.99 | -0.54 |
| 884 | 4-Penten-1-ol | -0.15 | -0.69 | -0.74 | 0.54 |
| 885 | Corticosterone | -3.24 | -3.78 | -3.76 | 0.54 |
| 886 | Tetralin | -4.37 | -3.82 | -3.87 | -0.55 |
| 887 | 2,3',4'-Trichlorobiphenyl | -6.29 | -5.74 | -5.73 | -0.55 |
| 888 | 2,3,6-Trichlorobiphenyl | -6.29 | -5.74 | -5.64 | -0.55 |
| 889 | 1,2,4-Tribromobenzene | -4.50 | -5.05 | -5.17 | 0.55 |
| 890 | 1-Butene | -1.94 | -1.39 | -1.36 | -0.55 |
| 891 | Allobarbital | -2.06 | -2.61 | -2.66 | 0.55 |
| 892 | 1-(1-Methylethoxy)propane | -1.34 | -0.79 | -0.74 | -0.55 |
| 893 | Ephedrine | -0.42 | -0.97 | -0.91 | 0.55 |
| 894 | Cyclohexanone | -0.60 | -1.15 | -1.15 | 0.55 |
| 895 | 2,2',4,5,5'-PCB | -7.89 | -7.34 | -7.30 | -0.55 |
| 896 | 1,3,5-Tribromobenzene | -5.60 | -5.05 | -4.93 | -0.55 |
| 897 | Coronene | -9.33 | -9.88 | -9.70 | 0.55 |
| 898 | Di-isooctyl phthalate | -6.64 | -6.08 | -6.16 | -0.56 |
| 899 | Methyl carbamate | 0.97 | 0.41 | 0.42 | 0.56 |
| 900 | Succinic acid | -0.20 | 0.36 | 0.42 | -0.56 |
| 901 | Salicylic acid | -1.82 | -1.26 | -1.31 | -0.56 |
| 902 | 1-Pentadecanoic acid | -4.31 | -3.75 | -3.63 | -0.56 |
| 903 | n-Butyl methyl ether | -0.99 | -0.43 | -0.50 | -0.56 |
| 904 | Molecule1447 | -1.55 | -0.99 | -1.01 | -0.56 |
| 905 | 4-Aminoacetanilide | -0.98 | -1.54 | -1.47 | 0.56 |
| 906 | Adrenosterone | -3.48 | -4.04 | -4.08 | 0.56 |
| 907 | Metolachlor | -2.73 | -3.29 | -3.50 | 0.56 |
| 908 | 2-Phenylphenol | -2.39 | -2.95 | -2.95 | 0.56 |
| 909 | Epiquinine | -2.76 | -3.32 | -3.33 | 0.56 |
| 910 | Digitoxin | -5.29 | -5.85 | -5.99 | 0.56 |
| 911 | 2,2',3,4',5,6'-Hexachlorobiphenyl | -8.71 | -8.14 | -8.08 | -0.57 |
| 912 | 2,2',4,4',6,6'-Hexachlorobiphenyl | -8.71 | -8.14 | -8.13 | -0.57 |
| 913 | Molecule159 | -6.89 | -6.32 | -6.28 | -0.57 |
| 914 | 2-Chlorobutane | -1.96 | -1.39 | -1.39 | -0.57 |
| 915 | m-Toluic acid | -2.14 | -1.57 | -1.55 | -0.57 |
| 916 | 1-Bromobutane | -2.37 | -1.80 | -1.76 | -0.57 |
| 917 | Ethyl chloride | -1.06 | -0.49 | -0.46 | -0.57 |
| 918 | Benzohydrol | -2.55 | -3.12 | -3.09 | 0.57 |
| 919 | Molecule809 | -2.32 | -2.89 | -2.87 | 0.57 |
| 920 | 3,5-Diiodotyrosine | -2.86 | -3.43 | -4.00 | 0.57 |
| 921 | Deoxycorticosterone | -3.75 | -4.32 | -4.35 | 0.57 |
| 922 | Phorate | -4.11 | -3.53 |  | -0.58 |
| 923 | trans-Geraniol | -2.46 | -1.88 | -1.95 | -0.58 |
| 924 | 11-Aminoundecanoic acid | -2.70 | -2.12 | -1.80 | -0.58 |
| 925 | N,N-Dimethylaniline | -1.92 | -1.34 | -1.18 | -0.58 |
| 926 | Molecule1471 | -1.60 | -1.02 | -1.02 | -0.58 |
| 927 | N-Acetylurea | -0.90 | -0.32 | -0.29 | -0.58 |
| 928 | 2,4-Xylenol | -1.19 | -1.77 | -1.73 | 0.58 |
| 929 | Methyl 4-hydroxybenzoate | -1.78 | -1.20 | -1.15 | -0.58 |
| 930 | Serine | -0.02 | 0.56 | 0.39 | -0.58 |
| 931 | 2,3,4,5,6-Pentachlorobiphenyl | -7.92 | -7.34 | -7.29 | -0.58 |
| 932 | 2,2',3,3',4,4',5,5'-Octachlorobiphenyl | -9.16 | -9.74 | -9.70 | 0.58 |
| 933 | Ethyl trans-cinnamate | -3.00 | -2.41 | -2.40 | -0.59 |
| 934 | Molecule502 | -1.50 | -0.91 | -0.52 | -0.59 |
| 935 | n-Butylformate | -1.37 | -0.78 | -0.83 | -0.59 |
| 936 | 3-Chloroanisole | -2.78 | -2.19 | -2.22 | -0.59 |
| 937 | 4-Chloroanisole | -2.78 | -2.19 | -2.22 | -0.59 |
| 938 | Molecule991 | -3.26 | -2.67 | -2.68 | -0.59 |
| 939 | Molecule379 | -4.18 | -3.59 | -3.51 | -0.59 |
| 940 | Butylate | -3.68 | -3.09 | -3.01 | -0.59 |
| 941 | 2,2',3,3',5,5',6,6'-Octachlorobiphenyl | -9.15 | -9.74 | -9.69 | 0.59 |
| 942 | Pericyazine | -3.98 | -4.57 | -4.61 | 0.59 |
| 943 | Cycloheptane | -3.52 | -4.11 | -4.06 | 0.59 |
| 944 | Molecule1348 | -5.51 | -4.91 | -4.88 | -0.60 |
| 945 | Butyl 4-hydroxybenzoate | -2.72 | -2.12 | -2.10 | -0.60 |
| 946 | 4-Chloro-m-cresol | -1.57 | -2.17 | -2.14 | 0.60 |
| 947 | 4-Nitroaniline | -2.37 | -1.77 | -1.75 | -0.60 |
| 948 | Tetrahydro-3-furanol | 1.05 | 0.45 | 0.51 | 0.60 |
| 949 | 2-Methyltetrahydrofuran | 0.11 | -0.49 | -0.52 | 0.60 |
| 950 | Phenylacetic acid | -0.89 | -1.49 | -1.52 | 0.60 |
| 951 | Cyanazine | -3.15 | -3.75 | -4.22 | 0.60 |
| 952 | o-Dinitrobenzene | -3.10 | -2.50 | -2.45 | -0.60 |
| 953 | 1-Naphthylamine | -1.92 | -2.52 | -2.51 | 0.60 |
| 954 | Valium | -3.76 | -4.36 | -4.10 | 0.60 |
| 955 | Molecule86 | -5.96 | -6.56 | -6.57 | 0.60 |
| 956 | Mephenamic acid | -3.78 | -4.39 | -4.42 | 0.61 |
| 957 | Cyclooctane | -4.15 | -4.76 | -4.71 | 0.61 |
| 958 | Metharbital | -2.23 | -1.62 | -1.63 | -0.61 |
| 959 | Thiourea | 0.32 | -0.29 | -0.30 | 0.61 |
| 960 | Dichlormid | -1.62 | -2.23 | -2.24 | 0.61 |
| 961 | Thebanone | -1.87 | -2.48 | -2.55 | 0.61 |
| 962 | p-Anthranilic acid | -0.40 | -1.01 | -1.04 | 0.61 |
| 963 | Cyclohexene | -2.59 | -1.98 | -2.03 | -0.61 |
| 964 | Benzocaine | -2.32 | -1.71 | -1.70 | -0.61 |
| 965 | Molecule760 | -4.91 | -4.30 | -4.10 | -0.61 |
| 966 | Molecule755 | -6.52 | -5.90 | -5.91 | -0.62 |
| 967 | Altretamine | -3.36 | -2.74 | -0.56 | -0.62 |
| 968 | Mirex | -6.80 | -7.42 | -8.05 | 0.62 |
| 969 | Hippuric acid | -1.68 | -1.06 | -1.03 | -0.62 |
| 970 | 2,3,4,5-Tetrachlorobiphenyl | -7.16 | -6.54 | -6.53 | -0.62 |
| 971 | 3-Methyl-2-butanone | -0.12 | -0.74 | -0.78 | 0.62 |
| 972 | 2-Propylpiperidine | -1.50 | -0.88 | -0.85 | -0.62 |
| 973 | Oxalic acid | 0.38 | 1.00 | 1.08 | -0.62 |
| 974 | Aldosterone | -3.85 | -3.23 | -3.24 | -0.62 |
| 975 | 3-Chlorobenzoic acid | -2.59 | -1.97 | -1.97 | -0.62 |
| 976 | sec-Phenethyl alcohol | -0.92 | -1.54 | -1.50 | 0.62 |
| 977 | 1,2-Diaminobenzene | -0.42 | -1.04 | -1.00 | 0.62 |
| 978 | Methyl-n-propylketone | -0.19 | -0.81 | -0.80 | 0.62 |
| 979 | Propanil | -3.00 | -3.62 | -3.67 | 0.62 |
| 980 | Phenylthiourea | -1.77 | -2.39 | -2.29 | 0.62 |
| 981 | 1-Octyne | -3.66 | -3.03 | -3.18 | -0.63 |
| 982 | 4-Carboxyphenol | -1.41 | -0.78 | -0.72 | -0.63 |
| 983 | 3-Chloroaniline | -1.37 | -2.00 | -2.01 | 0.63 |
| 984 | Butobarbitone | -1.64 | -2.27 | -2.35 | 0.63 |
| 985 | Durene | -4.59 | -3.96 | -4.01 | -0.63 |
| 986 | Dichlorprop | -2.45 | -3.08 | -3.08 | 0.63 |
| 987 | Molecule811 | -2.70 | -3.33 | -3.36 | 0.63 |
| 988 | Molecule757 | -5.89 | -5.26 | -5.27 | -0.63 |
| 989 | 2,2',3,3',4,4',6-Heptachlorobiphenyl | -8.30 | -8.94 | -8.93 | 0.64 |
| 990 | Pentylcarbamate | -1.47 | -0.83 | -0.82 | -0.64 |
| 991 | Phenanthridine | -2.78 | -3.42 | -3.32 | 0.64 |
| 992 | Amyl acetate | -1.89 | -1.25 | -1.29 | -0.64 |
| 993 | Molecule820 | -1.16 | -1.80 | -1.80 | 0.64 |
| 994 | Cyclobarbital | -2.17 | -2.81 | -2.82 | 0.64 |
| 995 | Molecule1077 | -3.02 | -2.38 | -2.46 | -0.64 |
| 996 | Molecule829 | -2.77 | -2.13 | -2.08 | -0.64 |
| 997 | Benzonitrile | -1.00 | -1.64 | -1.65 | 0.64 |
| 998 | Molecule283 | -2.10 | -2.74 | -2.74 | 0.64 |
| 999 | Acenaphthylene | -3.96 | -4.60 | -4.73 | 0.64 |
| 1000 | Hexylbenzene | -5.21 | -4.56 | -4.62 | -0.65 |
| 1001 | Diphenyl ether | -3.96 | -3.31 | -3.31 | -0.65 |
| 1002 | 4-Allyl-2-methoxyphenol | -1.56 | -2.21 | -2.27 | 0.65 |
| 1003 | Tetrahydrofuran | 0.56 | -0.09 | -0.01 | 0.65 |
| 1004 | 1,1-Dichlorobutane | -2.40 | -1.75 | -1.78 | -0.65 |
| 1005 | 4-Hydroxypyridine | 1.02 | 0.37 | 0.32 | 0.65 |
| 1006 | Azelaic acid | -1.89 | -1.24 | -1.16 | -0.65 |
| 1007 | 2-Ethylhexanal | -2.13 | -1.48 | -1.51 | -0.65 |
| 1008 | 1-Heptanol | -1.81 | -1.16 | -1.19 | -0.65 |
| 1009 | Benefin | -5.53 | -4.88 | -5.00 | -0.65 |
| 1010 | Quinalbarbitone | -2.36 | -3.01 | -3.02 | 0.65 |
| 1011 | Chloroethylene | -1.75 | -1.09 | -1.01 | -0.66 |
| 1012 | 1-Chloropropane | -1.47 | -0.81 | -0.81 | -0.66 |
| 1013 | Ethanol | 1.10 | 0.44 | 0.47 | 0.66 |
| 1014 | Parathion | -4.66 | -4.00 | -4.04 | -0.66 |
| 1015 | Propanenitrile | 0.28 | -0.38 | -0.44 | 0.66 |
| 1016 | 2-Chloronaphthalene | -4.14 | -3.48 | -3.34 | -0.66 |
| 1017 | 1,4-Phenylenediamine | -0.38 | -1.04 | -1.01 | 0.66 |
| 1018 | Methylsalicylate | -2.34 | -1.68 | -1.64 | -0.66 |
| 1019 | Ethoxybenzene | -2.33 | -1.67 | -1.65 | -0.66 |
| 1020 | Aprobarbital | -1.71 | -2.37 | -2.38 | 0.66 |
| 1021 | Phenyl benzoate | -2.85 | -3.51 | -3.44 | 0.66 |
| 1022 | Paspertin | -3.18 | -2.51 | -2.67 | -0.67 |
| 1023 | Talbutal | -2.02 | -2.69 | -2.69 | 0.67 |
| 1024 | Propyl butanoate | -1.92 | -1.25 | -1.28 | -0.67 |
| 1025 | Tropic acid | -0.93 | -1.60 | -1.63 | 0.67 |
| 1026 | p-Cresol | -0.70 | -1.37 | -1.30 | 0.67 |
| 1027 | Vitamin K3 | -3.03 | -2.36 | -2.39 | -0.67 |
| 1028 | Phenylethanolamine | -0.48 | -1.15 | -1.20 | 0.67 |
| 1029 | Indometacin | -4.62 | -5.30 | -5.21 | 0.68 |
| 1030 | Diphenic acid | -2.28 | -2.96 | -2.95 | 0.68 |
| 1031 | Meprobamate | -1.67 | -0.99 | -1.09 | -0.68 |
| 1032 | Diethylacetaldehyde | -1.52 | -0.84 | -0.82 | -0.68 |
| 1033 | 4-Androstene-3,17-dione | -3.69 | -4.37 | -4.37 | 0.68 |
| 1034 | Molecule631 | -1.20 | -1.88 | -1.98 | 0.68 |
| 1035 | Tetradecane | -7.96 | -8.64 | -8.88 | 0.68 |
| 1036 | Pentachlorophenol | -4.28 | -4.97 | -4.96 | 0.69 |
| 1037 | Xanthotoxin | -3.66 | -2.97 | -2.98 | -0.69 |
| 1038 | Doxycycline | -2.87 | -2.18 | -2.14 | -0.69 |
| 1039 | Demeclocycline | -2.52 | -3.21 | -3.09 | 0.69 |
| 1040 | Propynal | 0.57 | -0.12 | -0.13 | 0.69 |
| 1041 | Reposal | -2.64 | -3.33 | -3.30 | 0.69 |
| 1042 | m-Cresol | -0.68 | -1.37 | -1.40 | 0.69 |
| 1043 | Molecule1004 | -2.86 | -3.55 | -3.73 | 0.69 |
| 1044 | 1,2,4,5-Tetrabromobenzene | -6.98 | -6.28 | -6.09 | -0.70 |
| 1045 | 1-Iodobutane | -2.96 | -2.26 | -2.18 | -0.70 |
| 1046 | Molecule803 | -2.35 | -1.65 | -1.60 | -0.70 |
| 1047 | 1-Iodonaphthalene | -4.55 | -3.85 | -3.76 | -0.70 |
| 1048 | 4-Chloroiodobenzene | -4.03 | -3.33 | -3.52 | -0.70 |
| 1049 | Acetylacetone | 0.22 | -0.48 | -0.51 | 0.70 |
| 1050 | Tryptophane | -1.28 | -1.98 | -1.98 | 0.70 |
| 1051 | Molecule781 | -0.06 | -0.76 | -0.77 | 0.70 |
| 1052 | Isobutyl bromide | -2.43 | -1.73 | -1.64 | -0.70 |
| 1053 | Cyanoethylene | 0.15 | -0.55 | -0.64 | 0.70 |
| 1054 | 2-Ethyl-1-hexanol | -2.11 | -1.41 | -1.45 | -0.70 |
| 1055 | 2,3,4-Trichlorophenol | -2.67 | -3.37 | -3.41 | 0.70 |
| 1056 | 2,3,5-Trichlorophenol | -2.67 | -3.37 | -3.32 | 0.70 |
| 1057 | Molecule479 | -6.62 | -7.32 | -7.40 | 0.70 |
| 1058 | Dienestrol | -4.95 | -4.24 | -4.11 | -0.71 |
| 1059 | 2,3',4',5-Tetrachlorobiphenyl | -7.25 | -6.54 | -6.49 | -0.71 |
| 1060 | Methylcaproate | -2.00 | -1.29 | -1.33 | -0.71 |
| 1061 | 2-Chlorophenol | -1.06 | -1.77 | -1.78 | 0.71 |
| 1062 | 2-Butanol | 0.43 | -0.28 | -0.28 | 0.71 |
| 1063 | 8-Hydroxyquinoline | -2.42 | -1.71 | -1.66 | -0.71 |
| 1064 | Methylprednisolone | -2.99 | -3.70 | -3.81 | 0.71 |
| 1065 | Molecule241 | -5.43 | -4.72 | -4.61 | -0.71 |
| 1066 | Quinonamid | -5.03 | -4.31 | -4.16 | -0.72 |
| 1067 | 1,3,5-Trichlorobenzene | -4.48 | -3.76 | -3.71 | -0.72 |
| 1068 | 4-Aminopropiophenone | -2.63 | -1.91 | -1.99 | -0.72 |
| 1069 | Molecule756 | -6.30 | -5.58 | -5.62 | -0.72 |
| 1070 | Phenethyl alcohol | -0.74 | -1.46 | -1.49 | 0.72 |
| 1071 | Ethylcarbamate | 0.85 | 0.13 | 0.15 | 0.72 |
| 1072 | Benazolin | -2.61 | -3.33 | -3.90 | 0.72 |
| 1073 | 2,2',3,5,5',6-Hexachlorobiphenyl | -7.42 | -8.14 | -8.16 | 0.72 |
| 1074 | 3-Methylcholanthrene | -7.92 | -8.64 | -8.76 | 0.72 |
| 1075 | Molecule87 | -7.92 | -8.64 | -8.59 | 0.72 |
| 1076 | alpha-Estradiol | -4.84 | -4.11 | -4.09 | -0.73 |
| 1077 | Phenothrin | -5.24 | -5.97 | -5.89 | 0.73 |
| 1078 | Ethiofencarb | -2.09 | -2.82 | -2.89 | 0.73 |
| 1079 | Butamben | -3.08 | -2.35 | -2.33 | -0.73 |
| 1080 | 2,4-Dimethylquinoline | -1.94 | -2.67 | -2.66 | 0.73 |
| 1081 | Molecule638 | -1.94 | -2.67 | -2.62 | 0.73 |
| 1082 | Pyrimidine | 1.10 | 0.37 | 0.22 | 0.73 |
| 1083 | Threonine | -0.09 | 0.64 | 0.75 | -0.73 |
| 1084 | 2,3-Dihydro-1H-indole | -1.04 | -1.77 | -1.81 | 0.73 |
| 1085 | Hymexazol | -0.07 | -0.80 | -1.29 | 0.73 |
| 1086 | Contergan | -2.68 | -1.95 | -2.03 | -0.73 |
| 1087 | Molecule328 | -2.64 | -3.37 | -3.38 | 0.73 |
| 1088 | Lovastatin | -6.01 | -5.27 | -5.36 | -0.74 |
| 1089 | Molecule1183 | -4.85 | -4.11 | -4.02 | -0.74 |
| 1090 | Di-n-butyl phthalate | -4.40 | -3.66 | -3.69 | -0.74 |
| 1091 | Ethyl hexanoate | -2.31 | -1.57 | -1.55 | -0.74 |
| 1092 | Exalgin | -0.95 | -1.69 | -1.59 | 0.74 |
| 1093 | 2,2',3,3'-Tetrachlorobiphenyl | -7.28 | -6.54 | -6.53 | -0.74 |
| 1094 | Methyloxiran | -0.59 | 0.15 | 0.14 | -0.74 |
| 1095 | 2,3-Dichlorobutane | -2.70 | -1.96 | -1.99 | -0.74 |
| 1096 | Isopentylacetate | -1.92 | -1.18 | -1.16 | -0.74 |
| 1097 | Benzyl alcohol | -0.40 | -1.14 | -1.14 | 0.74 |
| 1098 | 3-Chlorobiphenyl | -4.88 | -4.14 | -4.10 | -0.74 |
| 1099 | Molecule821 | -1.74 | -0.99 | -0.85 | -0.75 |
| 1100 | o-Toluidine | -0.85 | -1.60 | -1.57 | 0.75 |
| 1101 | m-Toluidine | -0.85 | -1.60 | -1.56 | 0.75 |
| 1102 | Molecule573 | -1.60 | -2.35 | -2.42 | 0.75 |
| 1103 | o-Cresol | -0.62 | -1.37 | -1.38 | 0.75 |
| 1104 | cis-Decalin | -5.19 | -5.94 | -5.98 | 0.75 |
| 1105 | 2,2',3,4,4',5-Hexachlorobiphenyl | -7.39 | -8.14 | -8.15 | 0.75 |
| 1106 | Benzo[e]pyrene | -8.80 | -8.04 | -8.09 | -0.76 |
| 1107 | Equilenin | -5.24 | -4.48 | -4.45 | -0.76 |
| 1108 | DDD | -7.20 | -6.44 | -6.42 | -0.76 |
| 1109 | 10-Undecenoic acid | -3.40 | -2.64 | -2.59 | -0.76 |
| 1110 | 1,2-Diphenylethane | -4.62 | -5.38 | -5.41 | 0.76 |
| 1111 | Molecule1102 | -2.33 | -1.57 |  | -0.76 |
| 1112 | Procymidone | -4.80 | -4.04 | -3.74 | -0.76 |
| 1113 | Propylgallate | -1.78 | -1.02 | -1.00 | -0.76 |
| 1114 | 3-Pyridinemethanol | 0.96 | 0.20 | 0.18 | 0.76 |
| 1115 | Isophorone | -1.06 | -1.82 | -1.87 | 0.76 |
| 1116 | 2-Bromotoluene | -2.23 | -2.99 | -2.98 | 0.76 |
| 1117 | Hexylcarbamate | -1.92 | -1.15 | -1.19 | -0.77 |
| 1118 | Cinnamic acid | -2.48 | -1.71 | -1.65 | -0.77 |
| 1119 | 2,6-Dichlorophenol | -1.79 | -2.57 | -2.55 | 0.78 |
| 1120 | Pyridine | 0.76 | -0.02 | -0.07 | 0.78 |
| 1121 | Arecoline | 0.81 | 0.03 | -0.04 | 0.78 |
| 1122 | Urea | 0.96 | 0.18 | 0.08 | 0.78 |
| 1123 | Cycloheptene | -3.18 | -2.40 | -2.34 | -0.78 |
| 1124 | Gibberellic acid | -1.84 | -2.62 | -2.69 | 0.78 |
| 1125 | Molecule923 | -1.42 | -2.20 | -2.22 | 0.78 |
| 1126 | Molecule405 | -4.53 | -5.31 | -5.31 | 0.78 |
| 1127 | Molecule421 | -4.53 | -5.31 | -5.36 | 0.78 |
| 1128 | Diosgenin | -7.32 | -6.53 | -6.62 | -0.79 |
| 1129 | 1-Nonyne | -4.24 | -3.45 | -3.41 | -0.79 |
| 1130 | Molecule1296 | -2.41 | -1.62 | -1.60 | -0.79 |
| 1131 | Aniline | -0.41 | -1.20 | -1.21 | 0.79 |
| 1132 | Diphenylmethane | -4.17 | -4.96 | -4.92 | 0.79 |
| 1133 | Triethylphosphate | 0.43 | 1.22 | 1.47 | -0.79 |
| 1134 | n-Octanoic acid | -2.30 | -1.51 | -1.46 | -0.79 |
| 1135 | 4-Bromoiodobenzene | -4.56 | -3.76 | -4.00 | -0.80 |
| 1136 | 2,3,5,6-Tetrachlorophenol | -3.37 | -4.17 | -4.15 | 0.80 |
| 1137 | Anethole | -3.13 | -2.33 | -2.33 | -0.80 |
| 1138 | Uracil | -1.48 | -0.68 | -0.67 | -0.80 |
| 1139 | Pyrrolidine | 1.15 | 0.35 | 0.26 | 0.80 |
| 1140 | Erythritol | 0.70 | 1.50 | 1.42 | -0.80 |
| 1141 | 4-Nitrophenol | -0.74 | -1.54 | -1.55 | 0.80 |
| 1142 | Quinoline | -1.30 | -2.10 | -2.06 | 0.80 |
| 1143 | Trifluralin | -5.68 | -4.88 | -4.87 | -0.80 |
| 1144 | Molecule1357 | -4.57 | -3.76 | -3.59 | -0.81 |
| 1145 | Cyhalothrin | -8.18 | -7.37 | -7.18 | -0.81 |
| 1146 | Caprylic aldehyde | -2.36 | -1.55 | -1.43 | -0.81 |
| 1147 | Acetic acid | 1.22 | 0.41 | 0.39 | 0.81 |
| 1148 | 2,6-Lutidine | 0.45 | -0.36 | -0.35 | 0.81 |
| 1149 | 1,2-Diethoxyethane | -0.77 | 0.04 | 0.06 | -0.81 |
| 1150 | Chlorfenac | -3.08 | -3.89 | -3.89 | 0.81 |
| 1151 | Ethyl oenanthate | -2.71 | -1.89 | -1.82 | -0.82 |
| 1152 | Parethoxycaine | -2.71 | -1.89 | -1.95 | -0.82 |
| 1153 | Molecule1301 | -1.24 | -0.42 | -0.44 | -0.82 |
| 1154 | 2-Methyl-1-pentene | -3.03 | -2.21 | -2.23 | -0.82 |
| 1155 | Abietic acid | -3.80 | -4.62 | -4.56 | 0.82 |
| 1156 | Abate | -6.24 | -7.06 | -7.62 | 0.82 |
| 1157 | Chloroacetic acid | 0.93 | 0.10 | 0.10 | 0.83 |
| 1158 | 3-Hydroxypyridine | -0.46 | 0.37 | 0.33 | -0.83 |
| 1159 | Molecule632 | -2.54 | -1.71 | -1.66 | -0.83 |
| 1160 | Hematein | -2.70 | -1.86 | -1.63 | -0.84 |
| 1161 | 2-Nitroacetanilide | -1.91 | -2.75 | -2.79 | 0.84 |
| 1162 | alpha-Chloralose | -1.84 | -1.00 | -0.90 | -0.84 |
| 1163 | Niclosamide | -4.70 | -5.54 | -5.57 | 0.84 |
| 1164 | Isopentyl bromide | -2.89 | -2.05 | -2.07 | -0.84 |
| 1165 | 2,2',6,6'-Tetrachlorobiphenyl | -7.39 | -6.54 | -6.54 | -0.85 |
| 1166 | Isosparteine | -1.89 | -1.04 | -0.79 | -0.85 |
| 1167 | Dimethylacetamide | 1.11 | 0.26 | 0.20 | 0.85 |
| 1168 | Methanol | 1.57 | 0.72 | 0.69 | 0.85 |
| 1169 | 3,3'-Dichlorobiphenyl | -5.80 | -4.94 | -4.93 | -0.86 |
| 1170 | 2-Nonanol | -2.74 | -1.88 | -1.81 | -0.86 |
| 1171 | Molecule1123 | -3.89 | -4.75 | -4.74 | 0.86 |
| 1172 | Triclosan | -4.46 | -5.32 | -5.26 | 0.86 |
| 1173 | Griseofulvin | -4.61 | -3.74 | -3.78 | -0.87 |
| 1174 | 1-Pentene | -2.68 | -1.81 | -1.84 | -0.87 |
| 1175 | Ethyl 4-hydroxybenzoate | -2.35 | -1.48 | -1.49 | -0.87 |
| 1176 | Carbanilide | -3.15 | -4.02 | -3.99 | 0.87 |
| 1177 | DEHP | -6.96 | -6.08 | -6.11 | -0.88 |
| 1178 | Molecule805 | -3.17 | -2.29 | -2.19 | -0.88 |
| 1179 | Molecule719 | -1.54 | -0.66 | -0.48 | -0.88 |
| 1180 | Molecule801 | -1.89 | -1.01 | -0.85 | -0.88 |
| 1181 | n-Hexylacetate | -2.46 | -1.57 | -1.57 | -0.89 |
| 1182 | 2,3-Dichloro-2-methylbutane | -2.69 | -1.80 | -1.92 | -0.89 |
| 1183 | p-Dinitrobenzene | -3.39 | -2.50 | -2.36 | -0.89 |
| 1184 | 1-Methylcyclohexene | -3.27 | -2.38 | -2.37 | -0.89 |
| 1185 | alpha-D-Glucose | 0.74 | 1.63 | 1.64 | -0.89 |
| 1186 | Metalaxyl | -1.60 | -2.49 | -2.51 | 0.89 |
| 1187 | 2-Decanone | -3.31 | -2.41 | -2.43 | -0.90 |
| 1188 | 1-Chlorobutane | -2.03 | -1.13 | -1.11 | -0.90 |
| 1189 | Dopa | -1.60 | -0.70 | -0.66 | -0.90 |
| 1190 | Molecule1554 | -6.01 | -6.91 | -7.92 | 0.90 |
| 1191 | Brucine | -2.09 | -2.99 | -3.29 | 0.90 |
| 1192 | Molecule854 | -5.47 | -4.56 | -4.55 | -0.91 |
| 1193 | 3-Trifluoromethylaniline | -1.47 | -2.38 | -2.49 | 0.91 |
| 1194 | Glutamine | -0.55 | 0.36 | 0.30 | -0.91 |
| 1195 | 1-Octanol | -2.39 | -1.48 | -1.47 | -0.91 |
| 1196 | Di-n-propylether | -1.62 | -0.71 | -0.74 | -0.91 |
| 1197 | Codeine | -1.52 | -2.43 | -2.56 | 0.91 |
| 1198 | Fenoxycarb | -4.70 | -3.78 | -3.73 | -0.92 |
| 1199 | Rotenone | -4.42 | -5.34 | -5.56 | 0.92 |
| 1200 | Pelargonic acid | -2.75 | -1.83 | -1.77 | -0.92 |
| 1201 | Molecule1481 | -2.52 | -3.44 | -3.42 | 0.92 |
| 1202 | Phenylhydrazine | 0.07 | -0.85 |  | 0.92 |
| 1203 | 3-(1-Methyl-2-pyrrolidinyl)pyridine | 0.79 | -0.13 | 0.01 | 0.92 |
| 1204 | 2-Chloropentane | -2.63 | -1.71 | -1.72 | -0.92 |
| 1205 | 3-Chloropentane | -2.63 | -1.71 | -1.72 | -0.92 |
| 1206 | Lenacil | -4.59 | -3.67 | -3.52 | -0.92 |
| 1207 | 3-Iodopropanoic acid | -0.43 | -1.35 | -1.67 | 0.92 |
| 1208 | Deoxycholic acid | -3.95 | -4.87 | -4.88 | 0.92 |
| 1209 | Benzylurea | -0.95 | -1.87 | -1.96 | 0.92 |
| 1210 | Khellin | -2.40 | -3.32 | -3.72 | 0.92 |
| 1211 | Fluridone | -4.44 | -5.36 | -5.43 | 0.92 |
| 1212 | 2-Hydroxyacetanilide | -2.24 | -1.31 | -1.28 | -0.93 |
| 1213 | Vinclozolin | -4.93 | -4.00 | -3.88 | -0.93 |
| 1214 | Inositol | 0.35 | 1.28 | 1.63 | -0.93 |
| 1215 | 3-Iodobenzoic acid | -3.27 | -2.34 | -2.24 | -0.93 |
| 1216 | Glycocholic acid | -5.15 | -4.22 | -4.05 | -0.93 |
| 1217 | Chrysene | -8.06 | -7.12 | -7.23 | -0.94 |
| 1218 | 1-Chloro-2-methylpropane | -2.00 | -1.06 | -1.11 | -0.94 |
| 1219 | Molecule775 | -1.50 | -0.56 | -0.55 | -0.94 |
| 1220 | 2-Methyl-1-butene | -2.73 | -1.79 | -1.75 | -0.94 |
| 1221 | 2-Hydroxybenzylalcohol | -0.29 | -1.23 | -1.28 | 0.94 |
| 1222 | Asparagine | -0.74 | 0.20 | 0.36 | -0.94 |
| 1223 | Dicofol | -5.67 | -6.61 | -6.65 | 0.94 |
| 1224 | Anthracene | -6.35 | -5.40 | -5.30 | -0.95 |
| 1225 | 1-Bromopentane | -3.07 | -2.12 | -2.05 | -0.95 |
| 1226 | Chloramphenicol | -2.11 | -3.06 | -3.29 | 0.95 |
| 1227 | Ibuprofen | -3.99 | -3.03 | -2.95 | -0.96 |
| 1228 | Molecule1553 | -6.70 | -5.74 | -4.70 | -0.96 |
| 1229 | Molecule990 | -3.95 | -2.99 | -2.94 | -0.96 |
| 1230 | Eriodictyol | -3.62 | -2.66 | -2.50 | -0.96 |
| 1231 | Isobutylene | -2.33 | -1.37 | -1.34 | -0.96 |
| 1232 | Aldrin | -7.33 | -6.37 | -6.22 | -0.96 |
| 1233 | 2-Chlorotoluene | -3.52 | -2.56 | -2.55 | -0.96 |
| 1234 | Glutaric acid | 1.00 | 0.04 | 0.07 | 0.96 |
| 1235 | 2-Methoxyphenol | -1.96 | -1.00 | -0.96 | -0.96 |
| 1236 | Cholic acid | -3.37 | -4.33 | -4.35 | 0.96 |
| 1237 | Ethisterone | -5.66 | -4.69 | -4.60 | -0.97 |
| 1238 | Disulfoton | -4.23 | -3.26 | -3.07 | -0.97 |
| 1239 | n-Butylbenzoate | -3.48 | -2.51 | -2.55 | -0.97 |
| 1240 | Pycene | -7.87 | -8.84 | -9.00 | 0.97 |
| 1241 | Hydrobenzoin | -1.93 | -2.90 | -2.90 | 0.97 |
| 1242 | 2,3-Lutidine | 0.38 | -0.59 | -0.60 | 0.97 |
| 1243 | 2,4-Lutidine | 0.38 | -0.59 | -0.60 | 0.97 |
| 1244 | Phenol | 0.00 | -0.97 | -1.01 | 0.97 |
| 1245 | Stearic acid | -5.68 | -4.71 | -4.69 | -0.97 |
| 1246 | Dexivacaine | -1.55 | -2.53 | -2.65 | 0.98 |
| 1247 | Iodofenphos | -6.62 | -5.64 | -5.55 | -0.98 |
| 1248 | Cymperator | -8.02 | -7.03 | -6.99 | -0.99 |
| 1249 | 2,5-Lutidine | 0.40 | -0.59 | -0.59 | 0.99 |
| 1250 | 3-Methyl-1-butene | -2.73 | -1.74 | -1.74 | -0.99 |
| 1251 | Molecule1097 | -2.27 | -3.26 | -3.17 | 0.99 |
| 1252 | 1,2,4,5-Tetrachlorobenzene | -5.56 | -4.56 | -4.55 | -1.00 |
| 1253 | Molecule660 | -4.55 | -3.55 | -3.48 | -1.00 |
| 1254 | Diclobenil | -4.24 | -3.24 | -3.17 | -1.00 |
| 1255 | cis-2-Pentene | -2.54 | -1.54 | -1.61 | -1.00 |
| 1256 | 1-Hexene | -3.23 | -2.23 | -2.25 | -1.00 |
| 1257 | Triamcinolone | -3.69 | -2.69 | -2.55 | -1.00 |
| 1258 | Molecule408 | -2.62 | -3.62 | -3.80 | 1.00 |
| 1259 | Equilin | -5.28 | -4.27 | -4.31 | -1.01 |
| 1260 | Molecule794 | 0.59 | -0.42 | -0.67 | 1.01 |
| 1261 | 2-Butanone | 0.52 | -0.49 | -0.51 | 1.01 |
| 1262 | Molecule712 | -0.84 | 0.17 | 0.19 | -1.01 |
| 1263 | 2,2',3,4,4',5',6-Heptachlorobiphenyl | -7.92 | -8.94 | -8.97 | 1.02 |
| 1264 | 2,2',3,4,4',5,6-Heptachlorobiphenyl | -7.92 | -8.94 | -8.93 | 1.02 |
| 1265 | 2,4-Dichlorophenol | -1.55 | -2.57 | -2.56 | 1.02 |
| 1266 | Molecule1339 | -2.92 | -1.90 | -1.95 | -1.02 |
| 1267 | 2,3,4,5-Tetrachlorophenol | -3.15 | -4.17 | -4.17 | 1.02 |
| 1268 | Ocotoic acid | -1.93 | -0.91 | -0.85 | -1.02 |
| 1269 | 2-Ethylpyridine | 0.51 | -0.51 | -0.55 | 1.02 |
| 1270 | Methyldymron | -3.35 | -4.37 | -4.58 | 1.02 |
| 1271 | 1-Butanethiol | -2.18 | -1.15 |  | -1.03 |
| 1272 | p-Toluic acid | -2.60 | -1.57 | -1.49 | -1.03 |
| 1273 | Di-n-propylsulfide | -2.58 | -1.55 | -1.30 | -1.03 |
| 1274 | 2-Ethyl-2-hexenal | -2.46 | -1.43 | -1.44 | -1.03 |
| 1275 | Molecule175 | -6.59 | -7.62 | -7.74 | 1.03 |
| 1276 | 2,4,6-Trichlorophenol | -2.34 | -3.37 | -3.36 | 1.03 |
| 1277 | Benzyl butyl phthalate | -5.64 | -4.60 | -4.57 | -1.04 |
| 1278 | Molecule637 | -5.40 | -4.36 | -4.06 | -1.04 |
| 1279 | 6-Chrysenamine | -6.20 | -5.16 | -4.84 | -1.04 |
| 1280 | Molecule984 | -2.30 | -3.34 | -3.47 | 1.04 |
| 1281 | 2-Methyl-2-butene | -2.56 | -1.52 | -1.56 | -1.04 |
| 1282 | 1,1,1-Trifluoro-2-propanol | 0.30 | -0.74 | -0.60 | 1.04 |
| 1283 | Piperidine | 1.07 | 0.03 | -0.01 | 1.04 |
| 1284 | Tetramethylurea | 0.94 | -0.10 | -0.29 | 1.04 |
| 1285 | n-Octylamine | -2.75 | -1.71 | -1.66 | -1.04 |
| 1286 | Acrylamide | 0.96 | -0.09 | -0.12 | 1.05 |
| 1287 | N-Methyl-2-pyrrolidone | 1.00 | -0.05 | -0.04 | 1.05 |
| 1288 | 6-Methylchrysene | -6.57 | -7.62 | -7.67 | 1.05 |
| 1289 | Murocholic acid | -3.82 | -4.87 | -4.85 | 1.05 |
| 1290 | 2-Methylanthracene | -6.96 | -5.90 | -5.82 | -1.06 |
| 1291 | Alloxan | -1.25 | -0.19 | -0.17 | -1.06 |
| 1292 | 4-Nitrobenzoic acid | -2.80 | -1.74 | -1.75 | -1.06 |
| 1293 | Molecule644 | -4.75 | -3.69 | -3.52 | -1.06 |
| 1294 | 4-Chlorobiphenyl | -5.20 | -4.14 | -4.10 | -1.06 |
| 1295 | 2,3,4,6-Tetrachlorophenol | -3.10 | -4.17 | -4.13 | 1.07 |
| 1296 | Molecule501 | -1.16 | -0.09 |  | -1.07 |
| 1297 | 3-Chlorophenol | -0.70 | -1.77 | -1.73 | 1.07 |
| 1298 | 4-Chlorophenol | -0.70 | -1.77 | -1.72 | 1.07 |
| 1299 | Naproxen | -4.16 | -3.09 | -3.06 | -1.07 |
| 1300 | n-Undecanoic acid | -3.55 | -2.47 | -2.53 | -1.08 |
| 1301 | 4-Methyl-1-pentene | -3.24 | -2.16 | -2.23 | -1.08 |
| 1302 | 2-Hydroxypyridine | 1.02 | -0.06 | -0.01 | 1.08 |
| 1303 | 1-Heptene | -3.73 | -2.65 | -2.68 | -1.08 |
| 1304 | Ethalfluralin | -6.12 | -5.03 | -4.77 | -1.09 |
| 1305 | Dimetan | -0.85 | -1.94 | -2.05 | 1.09 |
| 1306 | Oxipurinol | -1.99 | -0.90 | -0.39 | -1.09 |
| 1307 | Molecule804 | -3.06 | -1.97 | -1.97 | -1.09 |
| 1308 | Simazine | -4.55 | -3.46 | -3.21 | -1.09 |
| 1309 | Molecule1033 | -0.35 | -1.45 | -1.45 | 1.10 |
| 1310 | Sorbic acid | -1.77 | -0.67 | -0.65 | -1.10 |
| 1311 | 2-Chloroacetanilide | -1.40 | -2.50 | -2.55 | 1.10 |
| 1312 | Dicamba | -1.70 | -2.80 | -2.78 | 1.10 |
| 1313 | 7,12-Dimethylbenz[a]anthracene | -7.02 | -8.12 | -8.25 | 1.10 |
| 1314 | Di-n-octyl phthalate | -5.11 | -6.22 | -6.12 | 1.11 |
| 1315 | Methoxychlor | -6.89 | -5.78 | -5.68 | -1.11 |
| 1316 | Dibenzofuran | -4.60 | -3.49 | -3.49 | -1.11 |
| 1317 | 2-Thiouracil | -2.26 | -1.15 | -1.00 | -1.11 |
| 1318 | Cellobiose | -0.24 | 0.87 | 0.97 | -1.11 |
| 1319 | N,N-Diethylaniline | -3.03 | -1.92 | -1.87 | -1.11 |
| 1320 | 4-Bromophenol | -1.09 | -2.20 | -2.21 | 1.11 |
| 1321 | Molecule174 | -7.01 | -8.12 | -7.93 | 1.11 |
| 1322 | Amikacin | -0.50 | 0.62 | 2.03 | -1.12 |
| 1323 | 9-Anthracenol | -4.73 | -3.61 | -3.36 | -1.12 |
| 1324 | Methaqualone | -2.92 | -4.05 | -4.54 | 1.13 |
| 1325 | N-Methylurea | 1.13 | 0.00 | 0.00 | 1.13 |
| 1326 | Methylpelargonate | -3.38 | -2.25 | -2.22 | -1.13 |
| 1327 | Phthalic acid | -2.11 | -0.98 | -0.97 | -1.13 |
| 1328 | Diisobutyl phthalate | -4.66 | -3.52 | -3.56 | -1.14 |
| 1329 | Molecule1000 | -3.21 | -4.35 | -5.21 | 1.14 |
| 1330 | Molecule666 | -4.87 | -3.72 | -3.52 | -1.15 |
| 1331 | Naepaine | -3.27 | -2.12 | -2.07 | -1.15 |
| 1332 | Molecule1037 | -2.62 | -1.47 | -1.51 | -1.15 |
| 1333 | Phthalimide | -2.61 | -1.46 | -1.38 | -1.15 |
| 1334 | Prostaglandin E2 | -2.47 | -3.62 | -3.70 | 1.15 |
| 1335 | Molecule646 | -3.67 | -2.51 | -2.43 | -1.16 |
| 1336 | 2-Pyrrolidone | 1.07 | -0.09 | -0.16 | 1.16 |
| 1337 | Gallic acid | -1.16 | 0.00 | 0.14 | -1.16 |
| 1338 | 2,4,5-Trichlorophenol | -2.21 | -3.37 | -3.32 | 1.16 |
| 1339 | Scopolamine | -0.48 | -1.65 | -1.87 | 1.17 |
| 1340 | Benzo[b]fluorene | -8.04 | -6.86 | -6.87 | -1.18 |
| 1341 | Acetamide | 1.58 | 0.40 | 0.47 | 1.18 |
| 1342 | Ethyl octanoate | -3.39 | -2.21 | -2.17 | -1.18 |
| 1343 | 3,4-Lutidine | 0.36 | -0.82 | -0.92 | 1.18 |
| 1344 | Molecule1206 | -5.19 | -4.01 | -4.03 | -1.18 |
| 1345 | Antazoline | -2.60 | -3.78 | -4.37 | 1.18 |
| 1346 | Di-n-hexyl phthalate | -6.14 | -4.94 | -4.97 | -1.20 |
| 1347 | Phenallymal | -2.18 | -3.38 | -3.35 | 1.20 |
| 1348 | Pyrocatechol | 0.62 | -0.58 | -0.61 | 1.20 |
| 1349 | 3,5-Lutidine | 0.38 | -0.82 | -0.85 | 1.20 |
| 1350 | 1-Nonanol | -3.01 | -1.80 | -1.80 | -1.21 |
| 1351 | 2-(Methylamino)benzoic acid | -2.88 | -1.67 | -1.65 | -1.21 |
| 1352 | Molecule902 | -2.28 | -1.06 | -0.42 | -1.22 |
| 1353 | 2,2'-Bipyridine | -1.42 | -0.20 | -0.11 | -1.22 |
| 1354 | Molecule383 | -5.36 | -4.14 | -4.07 | -1.22 |
| 1355 | 3,5-Dichlorophenol | -1.34 | -2.57 | -2.55 | 1.23 |
| 1356 | Ursodiol | -3.64 | -4.87 | -4.89 | 1.23 |
| 1357 | Digoxin | -4.08 | -5.31 | -5.62 | 1.23 |
| 1358 | Molecule1182 | -4.11 | -2.87 | -2.89 | -1.24 |
| 1359 | Bromopropylate | -4.93 | -6.17 | -6.27 | 1.24 |
| 1360 | Vanillic acid | -2.05 | -0.81 | -0.81 | -1.24 |
| 1361 | Ethyl caprate | -4.10 | -2.85 | -2.89 | -1.25 |
| 1362 | 2,3',4,4'-Tetrachlorobiphenyl | -7.80 | -6.54 | -6.53 | -1.26 |
| 1363 | Ethyl nonanoate | -3.80 | -2.53 | -2.56 | -1.27 |
| 1364 | Morphine | -3.28 | -2.01 | -1.82 | -1.27 |
| 1365 | 2,3-Dichlorophenol | -1.30 | -2.57 | -2.59 | 1.27 |
| 1366 | Fenbufen | -5.06 | -3.78 | -3.71 | -1.28 |
| 1367 | 2-Chloro-2-methylbutane | -2.51 | -1.23 | -1.25 | -1.28 |
| 1368 | 1-Chloropentane | -2.73 | -1.45 | -1.36 | -1.28 |
| 1369 | Propiconazole | -3.49 | -4.77 | -5.27 | 1.28 |
| 1370 | Molecule989 | -4.60 | -3.31 | -3.31 | -1.29 |
| 1371 | n-Decanoic acid | -3.44 | -2.15 | -2.07 | -1.29 |
| 1372 | Molecule739 | -7.42 | -6.12 | -5.70 | -1.30 |
| 1373 | n-Nonanal | -3.17 | -1.87 | -1.86 | -1.30 |
| 1374 | Butoxycaine | -3.84 | -2.53 | -2.59 | -1.31 |
| 1375 | Tributylamine | -3.12 | -1.81 | -1.77 | -1.31 |
| 1376 | 3,4-Dichlorophenol | -1.25 | -2.57 | -2.53 | 1.32 |
| 1377 | Etomidate | -4.74 | -3.42 | -3.24 | -1.32 |
| 1378 | Liothyronine | -5.22 | -6.55 | -7.33 | 1.33 |
| 1379 | 2-Aminoanthracene | -5.17 | -3.84 | -3.75 | -1.33 |
| 1380 | Molecule1003 | -2.10 | -3.44 | -3.66 | 1.34 |
| 1381 | 4-Chlorobenzoic acid | -3.31 | -1.97 | -1.97 | -1.34 |
| 1382 | 1-Chlorohexane | -3.12 | -1.77 | -1.75 | -1.35 |
| 1383 | Caprylene | -4.44 | -3.07 | -3.00 | -1.37 |
| 1384 | Hexyl bromide | -3.81 | -2.44 | -2.46 | -1.37 |
| 1385 | Estriol | -4.95 | -3.57 | -3.29 | -1.38 |
| 1386 | Molecule667 | -5.15 | -3.77 | -3.60 | -1.38 |
| 1387 | GBL | 1.07 | -0.31 | -0.29 | 1.38 |
| 1388 | Actidione | -1.13 | -2.51 | -2.54 | 1.38 |
| 1389 | Resorcinol | 0.81 | -0.58 | -0.61 | 1.39 |
| 1390 | Ethyl Biscoumacetate | -3.66 | -5.08 | -5.29 | 1.42 |
| 1391 | Tripropylamine | -2.28 | -0.85 | -0.81 | -1.43 |
| 1392 | cis-2-Heptene | -3.82 | -2.38 | -2.40 | -1.44 |
| 1393 | Clonazepam | -3.50 | -4.94 | -5.28 | 1.44 |
| 1394 | 3,4-Dichlorobiphenyl | -6.39 | -4.94 | -4.83 | -1.45 |
| 1395 | Molecule731 | 0.96 | -0.49 | -0.68 | 1.45 |
| 1396 | beta-Aminobutyric acid | 1.08 | -0.38 | -0.61 | 1.46 |
| 1397 | Methylcaprylate | -3.39 | -1.93 | -1.90 | -1.46 |
| 1398 | Tetracene | -8.60 | -7.12 | -6.95 | -1.48 |
| 1399 | Tyrosine | -2.57 | -1.09 | -1.00 | -1.48 |
| 1400 | Methyllaurate | -4.69 | -3.21 | -3.31 | -1.48 |
| 1401 | Carbazole | -4.97 | -3.48 | -3.50 | -1.49 |
| 1402 | Glycine anhydride | -0.83 | 0.66 | 0.75 | -1.49 |
| 1403 | n-Decyl alcohol | -3.63 | -2.12 | -2.11 | -1.51 |
| 1404 | Molecule1036 | -3.30 | -1.79 | -1.79 | -1.51 |
| 1405 | 4,6-Dinitro-o-cresol | -1.46 | -2.99 | -3.12 | 1.53 |
| 1406 | Cetane | -8.40 | -9.94 | -10.22 | 1.54 |
| 1407 | 1-Nonene | -5.05 | -3.49 | -3.43 | -1.56 |
| 1408 | 1-Iodoheptane | -4.81 | -3.22 | -2.94 | -1.59 |
| 1409 | 1-Decene | -5.51 | -3.91 | -3.82 | -1.60 |
| 1410 | Molecule1134 | -2.90 | -4.51 | -4.58 | 1.61 |
| 1411 | Norbormide | -3.93 | -5.54 | -5.85 | 1.61 |
| 1412 | 4,4'-Dichlorobiphenyl | -6.56 | -4.94 | -4.88 | -1.62 |
| 1413 | Nalidixic acid | -3.37 | -1.75 | -1.57 | -1.62 |
| 1414 | Cytisine | 0.36 | -1.27 | -1.65 | 1.63 |
| 1415 | Heptyl bromide | -4.43 | -2.76 | -2.83 | -1.67 |
| 1416 | Anthraquinone | -5.19 | -3.50 | -3.44 | -1.69 |
| 1417 | Molecule1132 | -7.32 | -5.59 | -5.39 | -1.73 |
| 1418 | Molecule988 | -5.40 | -3.63 | -3.61 | -1.77 |
| 1419 | Vitamin B2 | -3.68 | -1.88 | -1.00 | -1.80 |
| 1420 | Phenobarbital | -2.34 | -4.15 | -4.23 | 1.81 |
| 1421 | Isopropalin | -6.49 | -4.67 | -4.63 | -1.82 |
| 1422 | Lauric acid | -4.62 | -2.79 | -2.71 | -1.83 |
| 1423 | Molecule751 | -2.93 | -1.08 | -1.04 | -1.85 |
| 1424 | Dibenzo-p-dioxin | -5.31 | -3.46 | -3.32 | -1.85 |
| 1425 | Tetradecanoic acid | -5.33 | -3.43 | -3.41 | -1.90 |
| 1426 | 1-Chloroheptane | -3.99 | -2.09 | -2.07 | -1.90 |
| 1427 | 1-Dodecanol | -4.67 | -2.76 | -2.67 | -1.91 |
| 1428 | Phthalamide | -2.92 | -1.00 | -0.80 | -1.92 |
| 1429 | Molecule1173 | -6.70 | -4.75 | -4.73 | -1.95 |
| 1430 | 1-Bromooctane | -5.06 | -3.08 | -2.99 | -1.98 |
| 1431 | Methyl caprate | -4.63 | -2.57 | -2.50 | -2.06 |
| 1432 | Fluotrimazol | -8.40 | -6.33 | -5.03 | -2.07 |
| 1433 | Quatrimycin | -3.12 | -1.05 | -0.70 | -2.07 |
| 1434 | Oxytetracycline | -3.14 | -0.99 | -0.91 | -2.15 |
| 1435 | Etofenprox | -8.60 | -6.28 | -5.97 | -2.32 |
| 1436 | Coumatetralyl | -2.84 | -5.18 | -5.32 | 2.34 |
| 1437 | Raffinose | -0.41 | 1.94 | 2.66 | -2.35 |
| 1438 | 1-Pentadecanol | -6.35 | -3.72 | -3.75 | -2.63 |
| 1439 | Pyrazinamide | -0.91 | 1.73 | 2.10 | -2.64 |
| 1440 | Myristyl alcohol | -6.05 | -3.40 | -3.39 | - |

2.65;1441;Palmitic acid;-6.81;-4.07;-4.08;-2.74
