# Supplementary material for: A Generally Applicable Computer Algorithm Based on the Group Additivity Method for the Calculation of Seven Molecular Descriptors: Heat of Combustion, LogPO/W, LogS, Refractivity, Polarizability, Toxicity and LogBB of Organic Compounds; Scope and Limits of Applicability
Source: Molecules. 2015 Oct 7;20(10):18279–351. doi: 10.3390/molecules201018279 (PMC6332030; doi:10.3390/molecules201018279)
Supplement: Supplementary file 1 [file molecules-20-18279-s001.zip › Experimental vs Calculated Polarizability Data Table.doc]

**Experimental vs. Calculated Molecular Polarizability Data, sorted by a bsolute Deviation**

| **No.** | **Molecule name** | **Polarizability** | **Polarizability calc** | **Polarizability test** | **Deviation** |
| --- | --- | --- | --- | --- | --- |
|  |  |  |  |  |  |
| 1 | Iodobenzene | 15.50 | 15.50 | 15.49 | 0.00 |
| 2 | Acetaldehyde | 4.59 | 4.59 | 4.51 | 0.00 |
| 3 | 1-Methyl-1H-pyrazole | 8.99 | 8.99 |  | 0.00 |
| 4 | 1,4-Dioxane | 8.60 | 8.60 | 8.65 | 0.00 |
| 5 | Imidazole | 7.19 | 7.19 | 11.32 | 0.00 |
| 6 | 2-Chloroethanol | 7.10 | 7.10 | 7.06 | 0.00 |
| 7 | 1,5-Dimethylpyrazole | 10.72 | 10.72 |  | 0.00 |
| 8 | 1-Fluorononane | 17.34 | 17.33 | 17.32 | 0.01 |
| 9 | Diphenyl sulfide | 23.79 | 23.78 |  | 0.01 |
| 10 | 1-Heptyne | 12.80 | 12.79 | 12.80 | 0.01 |
| 11 | 1-Ethyl-5-methylpyrazole | 12.50 | 12.49 |  | 0.01 |
| 12 | Crotonaldehyde | 8.50 | 8.49 | 8.51 | 0.01 |
| 13 | Ethane | 4.47 | 4.46 | 4.47 | 0.01 |
| 14 | Pyrrole | 7.93 | 7.92 | 10.65 | 0.01 |
| 15 | Butyraldehyde | 8.20 | 8.19 | 8.19 | 0.01 |
| 16 | Propanal | 6.38 | 6.39 | 6.39 | -0.01 |
| 17 | Diethylamine | 10.20 | 10.21 |  | -0.01 |
| 18 | 1,2-Dichlorobenzene | 14.27 | 14.28 | 14.25 | -0.01 |
| 19 | 1-Butyne | 7.41 | 7.39 | 7.36 | 0.02 |
| 20 | Trichloroacetonitrile | 10.42 | 10.44 |  | -0.02 |
| 21 | 1,4-Dichlorobutane | 12.00 | 12.02 | 11.91 | -0.02 |
| 22 | 1-Formylnaphthalene | 19.75 | 19.77 | 19.67 | -0.02 |
| 23 | Propanenitrile | 6.24 | 6.26 | 6.26 | -0.02 |
| 24 | Acetonitrile | 4.48 | 4.46 | 4.47 | 0.02 |
| 25 | Hydrazine | 3.46 | 3.44 |  | 0.02 |
| 26 | 2,3-Dimethylquinoxaline | 18.70 | 18.68 |  | 0.02 |
| 27 | m-Xylene | 14.20 | 14.22 | 14.17 | -0.02 |
| 28 | trans-Chlorobromoethylene | 9.28 | 9.30 |  | -0.02 |
| 29 | 4-Chlorobutanoic acid | 10.69 | 10.71 | 10.74 | -0.02 |
| 30 | Tetracene | 32.27 | 32.24 | 32.21 | 0.03 |
| 31 | 1-Naphthoic acid ethylester | 23.97 | 23.94 | 24.00 | 0.03 |
| 32 | Propionic acid | 6.90 | 6.93 | 6.94 | -0.03 |
| 33 | Acetic acid | 5.10 | 5.13 | 5.10 | -0.03 |
| 34 | Methanol | 3.32 | 3.35 | 3.34 | -0.03 |
| 35 | Chloroethylene | 6.41 | 6.38 | 6.42 | 0.03 |
| 36 | Propane | 6.29 | 6.26 | 6.27 | 0.03 |
| 37 | t-Butylbenzene | 17.80 | 17.77 | 17.77 | 0.03 |
| 38 | 1-Propylamine | 7.70 | 7.73 | 7.75 | -0.03 |
| 39 | 1,3-Dichlorobenzene | 14.32 | 14.28 | 14.29 | 0.04 |
| 40 | Benzene | 10.74 | 10.70 | 10.72 | 0.04 |
| 41 | Benzonitrile | 12.50 | 12.46 | 12.40 | 0.04 |
| 42 | Isoquinoline | 17.00 | 17.04 | 16.96 | -0.04 |
| 43 | Succinonitrile | 8.10 | 8.06 | 8.15 | 0.04 |
| 44 | Pyrazine | 8.98 | 9.02 | 9.05 | -0.04 |
| 45 | 2-Methylpropane | 8.14 | 8.18 | 8.19 | -0.04 |
| 46 | Isobutylene | 8.29 | 8.25 | 8.24 | 0.04 |
| 47 | Ethanol | 5.08 | 5.12 | 5.11 | -0.04 |
| 48 | Ethyl chloride | 6.40 | 6.44 | 6.45 | -0.04 |
| 49 | Styrene | 14.41 | 14.45 | 14.41 | -0.04 |
| 50 | 1-Chloro-2-methoxyethane | 8.71 | 8.66 | 8.62 | 0.05 |
| 51 | 2-Chloro-1-propanol | 8.89 | 8.84 | 8.80 | 0.05 |
| 52 | trans-2-Methyl-1,3-pentadiene | 12.10 | 12.15 | 12.24 | -0.05 |
| 53 | 1-Fluorodecane | 19.18 | 19.13 | 19.17 | 0.05 |
| 54 | 3-Methylheptane | 15.44 | 15.38 | 15.60 | 0.06 |
| 55 | 1,3-Difluorobenzene | 10.30 | 10.36 | 10.31 | -0.06 |
| 56 | 2-Chloro-1-butanol | 10.70 | 10.64 | 10.65 | 0.06 |
| 57 | Ethylbenzene | 14.20 | 14.26 | 14.26 | -0.06 |
| 58 | 1-Bromo-4-fluorobenzene | 13.40 | 13.34 | 13.29 | 0.06 |
| 59 | 3-Chloro-1-propanol | 8.84 | 8.90 | 9.00 | -0.06 |
| 60 | Acetophenone | 15.00 | 14.94 | 15.06 | 0.06 |
| 61 | 1-Pentyne | 9.12 | 9.19 | 9.17 | -0.07 |
| 62 | 1-Fluoroundecane | 21.00 | 20.93 | 20.99 | 0.07 |
| 63 | 1-Fluoroheptane | 13.66 | 13.73 | 13.70 | -0.07 |
| 64 | trans-1,2-Dichloroethylene | 8.15 | 8.22 | 8.23 | -0.07 |
| 65 | 1-Fluorooctane | 15.46 | 15.53 | 15.52 | -0.07 |
| 66 | 4-Chloropentanoic acid | 12.53 | 12.45 | 12.41 | 0.08 |
| 67 | N-Methylformamide | 5.91 | 5.83 |  | 0.08 |
| 68 | 2-Methyl-1-propanol | 8.92 | 8.84 | 8.84 | 0.08 |
| 69 | Ethyleneglycol | 5.70 | 5.78 | 5.81 | -0.08 |
| 70 | Methacrolein | 8.30 | 8.38 | 8.19 | -0.08 |
| 71 | 1,4-Dichlorobenzene | 14.20 | 14.28 | 14.29 | -0.08 |
| 72 | 2-Ethylnaphthalene | 21.36 | 21.44 | 21.35 | -0.08 |
| 73 | 1-Hexyne | 10.90 | 10.99 | 10.97 | -0.09 |
| 74 | 2-Chloronaphthalene | 19.58 | 19.67 | 19.82 | -0.09 |
| 75 | Chrysene | 32.15 | 32.24 | 31.99 | -0.09 |
| 76 | Anthracene | 24.97 | 25.06 | 25.25 | -0.09 |
| 77 | 1-Fluorododecane | 22.83 | 22.73 | 22.72 | 0.10 |
| 78 | 1-Iodopropane | 11.50 | 11.60 |  | -0.10 |
| 79 | Hexafluorobenzene | 9.58 | 9.68 | 9.83 | -0.10 |
| 80 | Methyl cellosolve | 7.44 | 7.34 | 7.45 | 0.10 |
| 81 | 1,3,5-Trinitrobenzene | 16.53 | 16.43 | 16.36 | 0.10 |
| 82 | 1-Iodobutane | 13.30 | 13.40 |  | -0.10 |
| 83 | 4-Nitroaniline | 14.10 | 13.99 | 13.95 | 0.11 |
| 84 | 1,3,5-Tri-t-butylbenzene | 31.80 | 31.91 | 32.12 | -0.11 |
| 85 | Bromobenzene | 13.62 | 13.51 | 13.59 | 0.11 |
| 86 | 1,5-Hexadiyne | 10.21 | 10.32 | 10.35 | -0.11 |
| 87 | cis-Chlorobromoethylene | 9.19 | 9.30 |  | -0.11 |
| 88 | 2-Decyn-1-ol | 18.99 | 19.10 | 19.16 | -0.11 |
| 89 | 5-Decyn-1-ol | 18.99 | 19.10 | 18.99 | -0.11 |
| 90 | 3-Chloropentanoic acid | 12.57 | 12.45 | 12.43 | 0.12 |
| 91 | 1-Bromo-2-chloroethane | 9.50 | 9.38 | 9.42 | 0.12 |
| 92 | Ethyl bromide | 7.28 | 7.40 | 7.42 | -0.12 |
| 93 | 2-Methylnaphthalene | 19.52 | 19.64 | 19.65 | -0.12 |
| 94 | Ethyl 2-chlorobutanoate | 14.16 | 14.28 | 14.25 | -0.12 |
| 95 | Ethyl butanoate | 12.23 | 12.36 | 12.37 | -0.13 |
| 96 | 1-Fluorohexane | 11.80 | 11.93 | 11.96 | -0.13 |
| 97 | Isobutyronitrile | 8.05 | 8.18 | 8.11 | -0.13 |
| 98 | Bromoethylene | 7.59 | 7.46 |  | 0.13 |
| 99 | 2-Chloroethyl ethyl ether | 10.56 | 10.43 | 10.58 | 0.13 |
| 100 | Pentane | 9.99 | 9.86 | 9.88 | 0.13 |
| 101 | trans-2-Butene | 8.49 | 8.36 | 8.26 | 0.13 |
| 102 | Ethyl benzoate | 16.90 | 16.76 | 16.77 | 0.14 |
| 103 | 9-Decyn-1-ol | 18.99 | 18.85 | 18.88 | 0.14 |
| 104 | Butane | 8.20 | 8.06 | 8.01 | 0.14 |
| 105 | Ethyl acetate | 8.62 | 8.76 | 8.80 | -0.14 |
| 106 | Diethyl succinate | 16.80 | 16.66 | 16.54 | 0.14 |
| 107 | 1-Bromobutane | 10.86 | 11.00 | 10.98 | -0.14 |
| 108 | 1-Heptene | 13.51 | 13.65 | 13.68 | -0.14 |
| 109 | Ethyl acetoacetate | 12.90 | 13.04 | 13.05 | -0.14 |
| 110 | Methylcyclohexylether | 13.40 | 13.54 |  | -0.14 |
| 111 | Isopropylcyclohexane | 17.20 | 17.06 | 17.06 | 0.14 |
| 112 | Isooctane | 15.44 | 15.29 | 15.27 | 0.15 |
| 113 | Heptane | 13.61 | 13.46 | 13.41 | 0.15 |
| 114 | 1,1-Difluoro-1,2,2-trichloroethane | 10.20 | 10.35 |  | -0.15 |
| 115 | 3-Chlorobutanoic acid | 10.80 | 10.65 | 10.61 | 0.15 |
| 116 | 2-Propanol | 6.97 | 6.82 | 6.77 | 0.15 |
| 117 | 1-Propanol | 6.77 | 6.92 | 6.90 | -0.15 |
| 118 | Methylamine | 4.01 | 4.16 | 4.26 | -0.15 |
| 119 | 1-Cyano-1,3-butadiene | 10.50 | 10.35 | 10.17 | 0.15 |
| 120 | Butyric acid | 8.58 | 8.73 | 8.82 | -0.15 |
| 121 | Ethyl propionate | 10.41 | 10.56 | 10.57 | -0.15 |
| 122 | Ethyl 3-chlorobutanoate | 14.13 | 14.28 | 14.28 | -0.15 |
| 123 | trans-Crotononitrile | 8.20 | 8.36 | 8.37 | -0.16 |
| 124 | 1,3,5-Trimethylbenzene | 16.14 | 15.98 | 15.95 | 0.16 |
| 125 | Hexyl bromide | 14.44 | 14.60 | 14.62 | -0.16 |
| 126 | Cryofluorane | 8.50 | 8.66 | 9.15 | -0.16 |
| 127 | 1-Chloropentane | 12.00 | 11.84 | 11.74 | 0.16 |
| 128 | Methylbenzene | 12.30 | 12.46 | 12.43 | -0.16 |
| 129 | 4-Iodotoluene | 17.10 | 17.26 | 17.33 | -0.16 |
| 130 | Triethylamine | 13.38 | 13.21 |  | 0.17 |
| 131 | 4-Fluoroiodobenzene | 15.50 | 15.33 | 15.29 | 0.17 |
| 132 | Acetylacetone (enol form) | 10.50 | 10.67 |  | -0.17 |
| 133 | 2-Ethoxyethanol | 9.28 | 9.11 | 9.13 | 0.17 |
| 134 | Naphthalene | 17.70 | 17.88 | 17.89 | -0.18 |
| 135 | 1-Bromooctane | 18.02 | 18.20 | 18.22 | -0.18 |
| 136 | t-Butyl cyanide | 9.59 | 9.77 | 9.78 | -0.18 |
| 137 | Cumene | 16.00 | 16.18 | 16.11 | -0.18 |
| 138 | 2-Chlorobutanoic acid methylester | 12.33 | 12.51 | 12.46 | -0.18 |
| 139 | Butyric acid methylester | 10.41 | 10.59 | 10.60 | -0.18 |
| 140 | Methyl acetate | 6.81 | 6.99 | 7.03 | -0.18 |
| 141 | Oxirane | 4.43 | 4.61 | 4.55 | -0.18 |
| 142 | 1-Fluoropentane | 9.95 | 10.13 | 10.16 | -0.18 |
| 143 | 1-Bromononane | 19.81 | 20.00 | 20.06 | -0.19 |
| 144 | Propylene | 6.26 | 6.45 | 6.46 | -0.19 |
| 145 | Chlorobenzene | 12.30 | 12.49 | 12.52 | -0.19 |
| 146 | cis-1,2-Dichloroethylene | 8.03 | 8.22 | 8.29 | -0.19 |
| 147 | 1-Bromopropane | 9.40 | 9.20 | 9.18 | 0.20 |
| 148 | Ethyl iodide | 10.00 | 9.80 |  | 0.20 |
| 149 | 1-Hexene | 11.65 | 11.85 | 11.88 | -0.20 |
| 150 | Methyl 3-chlorobutanoate | 12.31 | 12.51 | 12.56 | -0.20 |
| 151 | alpha-Methylstyrene | 16.05 | 16.25 | 16.25 | -0.20 |
| 152 | Propynal | 6.38 | 6.58 | 6.56 | -0.20 |
| 153 | Ethylene-1,2-diamine | 7.20 | 7.40 | 7.69 | -0.20 |
| 154 | s-cis-Butadiene | 8.64 | 8.44 | 8.35 | 0.20 |
| 155 | s-trans-1,3-Butadiene | 8.64 | 8.44 | 8.43 | 0.20 |
| 156 | 1-Bromodecane | 21.60 | 21.80 | 21.73 | -0.20 |
| 157 | Acetic anhydride | 8.90 | 9.11 | 9.52 | -0.21 |
| 158 | Ethoxybenzene | 14.90 | 15.11 | 15.12 | -0.21 |
| 159 | 1-Chloro-2-fluoroethane | 6.50 | 6.71 | 6.77 | -0.21 |
| 160 | Ethyne | 3.33 | 3.54 | 3.71 | -0.21 |
| 161 | trans-2,3-Epoxybutane | 8.22 | 8.01 | 7.60 | 0.21 |
| 162 | 2-Chlorobutanoic acid | 10.87 | 10.65 | 10.65 | 0.22 |
| 163 | Pentafluorobenzene | 9.63 | 9.85 | 9.92 | -0.22 |
| 164 | Acetamide | 5.67 | 5.89 | 5.86 | -0.22 |
| 165 | 4-Methylene-2-oxetanone | 8.00 | 8.22 |  | -0.22 |
| 166 | 4-(4-Bromophenoxy)toluene | 26.60 | 26.38 | 26.33 | 0.22 |
| 167 | Lauryl bromide | 25.18 | 25.40 | 25.33 | -0.22 |
| 168 | Fluorobenzene | 10.30 | 10.53 | 10.57 | -0.23 |
| 169 | Fluoroethane | 4.96 | 4.73 | 4.70 | 0.23 |
| 170 | Ethyl 4-chlorobutanoate | 14.11 | 14.34 | 14.44 | -0.23 |
| 171 | Pentachloroethane | 14.00 | 14.23 |  | -0.23 |
| 172 | 1-Fluorotetradecane | 26.57 | 26.33 | 26.29 | 0.24 |
| 173 | 1-Naphthylamine | 19.50 | 19.26 | 19.37 | 0.24 |
| 174 | 2-Chloropentanoic acid | 12.69 | 12.45 | 12.40 | 0.24 |
| 175 | Anisole | 13.10 | 13.34 | 13.17 | -0.24 |
| 176 | Hexane | 11.90 | 11.66 | 11.64 | 0.24 |
| 177 | 4-Methylquinoline | 18.65 | 18.41 | 18.35 | 0.24 |
| 178 | Methylcyclohexane | 13.10 | 13.34 | 13.37 | -0.24 |
| 179 | Decane | 19.10 | 18.86 | 18.75 | 0.24 |
| 180 | 2,3-Dimethyl-1,3-butadiene | 11.80 | 12.04 | 12.03 | -0.24 |
| 181 | 2-Ethyl-1,3-butadiene | 11.80 | 12.04 | 12.11 | -0.24 |
| 182 | N,N-Diethylaniline | 18.52 | 18.76 | 18.95 | -0.24 |
| 183 | 2-Naphthoic acid ethylester | 24.19 | 23.94 | 23.83 | 0.25 |
| 184 | 2-Methylacrylonitrile | 8.00 | 8.25 | 8.30 | -0.25 |
| 185 | Isoprene | 9.99 | 10.24 | 10.28 | -0.25 |
| 186 | Pyridine | 10.11 | 9.86 | 9.79 | 0.25 |
| 187 | 1-Ethylnaphthalene | 21.19 | 21.44 | 21.35 | -0.25 |
| 188 | N,N-Dimethylformamide | 7.81 | 7.55 |  | 0.26 |
| 189 | 1,1,1-Trichloroethane | 10.70 | 10.44 |  | 0.26 |
| 190 | 3-Chloro-1-butanol | 10.38 | 10.64 | 10.66 | -0.26 |
| 191 | Tripropylamine | 18.87 | 18.61 |  | 0.26 |
| 192 | Propionic acid methylester | 8.53 | 8.79 | 8.83 | -0.26 |
| 193 | Fluorene | 21.68 | 21.94 | 22.07 | -0.26 |
| 194 | 1-Bromohexadecane | 32.34 | 32.60 | 32.70 | -0.26 |
| 195 | 1-Iodonaphthalene | 22.41 | 22.68 | 22.86 | -0.27 |
| 196 | 2-Iodonaphthalene | 22.95 | 22.68 | 22.63 | 0.27 |
| 197 | 1-Chloro-1,3-butadiene | 10.00 | 10.28 | 10.29 | -0.28 |
| 198 | 1-Butene | 7.97 | 8.25 | 8.21 | -0.28 |
| 199 | Ethylene | 4.26 | 4.54 | 4.51 | -0.28 |
| 200 | Ethyl ether | 8.73 | 8.45 | 8.42 | 0.28 |
| 201 | 1-Bromooctadecane | 35.92 | 36.20 | 36.21 | -0.28 |
| 202 | Dodecane | 22.75 | 22.46 | 22.44 | 0.29 |
| 203 | Quinoxaline | 15.13 | 15.42 | 14.39 | -0.29 |
| 204 | 2-Formylnaphthalene | 20.06 | 19.77 | 19.73 | 0.29 |
| 205 | 1-Methylnaphthalene | 19.35 | 19.64 | 19.61 | -0.29 |
| 206 | 1-Bromopentane | 13.10 | 12.80 | 12.77 | 0.30 |
| 207 | Methyl 4-chlorobutanoate | 12.27 | 12.57 | 12.60 | -0.30 |
| 208 | Nonane | 17.36 | 17.06 | 17.12 | 0.30 |
| 209 | Anthraquinone | 25.41 | 25.10 | 25.05 | 0.31 |
| 210 | Nitrobenzene | 12.92 | 12.61 | 12.55 | 0.31 |
| 211 | 2,6-Dichloroaniline | 15.98 | 15.66 | 15.61 | 0.32 |
| 212 | cis-2-Pentene | 9.84 | 10.16 | 10.19 | -0.32 |
| 213 | trans-2-Pentene | 9.84 | 10.16 | 10.24 | -0.32 |
| 214 | 5-Methyluracil | 11.23 | 11.55 |  | -0.32 |
| 215 | 2-Phenyl-3-methyl-2-butene | 19.64 | 19.96 | 19.55 | -0.32 |
| 216 | 1,2,3,4-Tetrafluorobenzene | 9.69 | 10.02 | 10.13 | -0.33 |
| 217 | 1,2,4,5-Tetrafluorobenzene | 9.69 | 10.02 | 10.09 | -0.33 |
| 218 | Dimethylether | 5.24 | 4.91 | 4.84 | 0.33 |
| 219 | Formamide | 4.08 | 4.41 |  | -0.33 |
| 220 | 1,2-Diaminobenzene | 13.80 | 13.46 | 13.47 | 0.34 |
| 221 | 1,3-Diaminobenzene | 13.80 | 13.46 | 13.45 | 0.34 |
| 222 | 1,4-Phenylenediamine | 13.80 | 13.46 | 13.42 | 0.34 |
| 223 | Butyronitrile | 8.40 | 8.06 | 8.07 | 0.34 |
| 224 | 1,4-DI-t-butylbenzene | 24.50 | 24.84 | 24.97 | -0.34 |
| 225 | Uracil | 10.09 | 9.75 |  | 0.34 |
| 226 | Chloroacetonitrile | 6.10 | 6.44 | 6.51 | -0.34 |
| 227 | 4-Bromoaniline | 14.55 | 14.89 | 14.82 | -0.34 |
| 228 | trans-1,3-Pentadiene | 10.00 | 10.35 | 10.42 | -0.35 |
| 229 | Formic acid | 3.40 | 3.75 | 3.90 | -0.35 |
| 230 | 3-Methyl-1,3-pentadiene | 11.80 | 12.15 | 12.20 | -0.35 |
| 231 | 1-Bromonaphthalene | 20.34 | 20.69 | 20.66 | -0.35 |
| 232 | Quinoline | 17.00 | 16.65 | 16.64 | 0.35 |
| 233 | 3-Aminobutyronitrile | 9.17 | 9.53 | 9.60 | -0.36 |
| 234 | 1,2-Dibromoethane | 10.70 | 10.34 | 10.19 | 0.36 |
| 235 | Cyclopropane | 5.66 | 6.02 | 5.89 | -0.36 |
| 236 | 4-Fluoronitrobenzene | 12.80 | 12.44 | 12.42 | 0.36 |
| 237 | Hexamethylbenzene | 20.90 | 21.26 | 21.49 | -0.36 |
| 238 | Phenanthrene | 24.70 | 25.06 | 25.15 | -0.36 |
| 239 | Quinaldine | 18.65 | 18.28 |  | 0.37 |
| 240 | p-Toluidine | 13.47 | 13.84 | 13.82 | -0.37 |
| 241 | Undecane | 21.03 | 20.66 | 20.61 | 0.37 |
| 242 | N-Ethylacetamide | 9.45 | 9.08 | 8.94 | 0.37 |
| 243 | Bis(4-bromophenyl)ether | 27.80 | 27.43 | 27.34 | 0.37 |
| 244 | 4-Chloroaniline | 13.50 | 13.87 | 13.87 | -0.37 |
| 245 | 1-Chloronaphthalene | 19.30 | 19.67 | 19.82 | -0.37 |
| 246 | Methylpropylether | 8.86 | 8.48 | 8.47 | 0.38 |
| 247 | Phenol | 11.10 | 11.48 | 11.72 | -0.38 |
| 248 | N,N-Dimethylaniline | 14.84 | 15.22 |  | -0.38 |
| 249 | Diethylacetamide | 12.96 | 12.57 |  | 0.39 |
| 250 | 1,1-Dichloroethane | 8.64 | 8.25 |  | 0.39 |
| 251 | Isoquinaldine | 18.28 | 18.67 |  | -0.39 |
| 252 | 3-(Dimethylamino)butyronitrile | 12.87 | 13.27 |  | -0.40 |
| 253 | Pentamethylbenzene | 19.10 | 19.50 | 19.55 | -0.40 |
| 254 | 4-Fluoroaniline | 11.51 | 11.91 | 11.99 | -0.40 |
| 255 | 1-Pentene | 9.65 | 10.05 | 10.02 | -0.40 |
| 256 | Heptyl bromide | 16.80 | 16.40 | 16.37 | 0.40 |
| 257 | Acetoacetic acid ethylester Enolform | 12.90 | 12.49 |  | 0.41 |
| 258 | Ethylenedichloride | 8.00 | 8.42 | 8.65 | -0.42 |
| 259 | Cyclohexane | 11.00 | 11.42 | 11.44 | -0.42 |
| 260 | 2,6-Xylidine | 16.02 | 15.60 | 15.61 | 0.42 |
| 261 | Cyclohexanol | 11.56 | 11.98 |  | -0.42 |
| 262 | 4-Bromodiphenyl ether | 24.20 | 24.62 | 24.70 | -0.42 |
| 263 | N-Ethylaniline | 14.84 | 15.27 | 15.45 | -0.43 |
| 264 | Ethyl-n-propylether | 10.68 | 10.25 | 10.19 | 0.43 |
| 265 | 2,2-Dimethylpropane | 10.20 | 9.77 | 9.73 | 0.43 |
| 266 | Bis(4-tolyl) ether | 24.90 | 25.33 | 25.42 | -0.43 |
| 267 | Durene | 17.30 | 17.74 | 17.81 | -0.44 |
| 268 | 9,9'-Bifluorenyl | 42.81 | 43.26 | 43.35 | -0.45 |
| 269 | 9-Bromoanthracene | 28.32 | 27.87 | 27.81 | 0.45 |
| 270 | Di-n-propylether | 12.50 | 12.05 | 12.00 | 0.45 |
| 271 | 1,3,5-Trifluorobenzene | 9.74 | 10.19 | 10.24 | -0.45 |
| 272 | 4-Nitroanisole | 15.70 | 15.25 | 15.24 | 0.45 |
| 273 | 2-Naphthylamine | 19.73 | 19.26 | 19.28 | 0.47 |
| 274 | 4-Bromotoluene | 14.80 | 15.27 | 15.25 | -0.47 |
| 275 | Dicyanomethane | 5.79 | 6.26 | 6.37 | -0.47 |
| 276 | Cyclopentane | 9.15 | 9.62 | 9.63 | -0.47 |
| 277 | 2,4-Dichloroaniline | 15.18 | 15.66 | 15.80 | -0.48 |
| 278 | N-Methylaniline | 13.01 | 13.50 | 13.66 | -0.49 |
| 279 | 9-Chloroanthracene | 27.35 | 26.85 | 27.12 | 0.50 |
| 280 | Ethyl formate | 6.88 | 7.38 | 7.52 | -0.50 |
| 281 | Methylacetamide | 7.82 | 7.31 | 6.99 | 0.51 |
| 282 | Chloroacetone | 8.40 | 8.92 | 9.01 | -0.52 |
| 283 | Di-n-propylamine | 13.29 | 13.81 |  | -0.52 |
| 284 | 2-Methoxyaniline | 14.20 | 14.72 | 14.77 | -0.52 |
| 285 | Allyl alcohol | 7.65 | 7.11 | 7.07 | 0.54 |
| 286 | Dimethylamine | 7.21 | 6.67 |  | 0.54 |
| 287 | Paraldehyde | 17.90 | 17.36 |  | 0.54 |
| 288 | Pentanenitrile | 10.40 | 9.86 | 9.76 | 0.54 |
| 289 | 4-Chlorotoluene | 13.70 | 14.25 | 14.27 | -0.55 |
| 290 | Cyanogen | 5.01 | 4.46 | 4.35 | 0.55 |
| 291 | Acenaphthene | 20.61 | 21.16 | 21.20 | -0.55 |
| 292 | 2-Butanone | 8.19 | 8.74 | 8.76 | -0.55 |
| 293 | Acetone | 6.39 | 6.94 | 6.81 | -0.55 |
| 294 | 1,2-Difluorobenzene | 9.80 | 10.36 | 10.35 | -0.56 |
| 295 | 1,4-Difluorobenzene | 9.80 | 10.36 | 10.33 | -0.56 |
| 296 | Methyl formate | 5.05 | 5.61 | 5.85 | -0.56 |
| 297 | Propyne | 6.18 | 5.59 | 5.59 | 0.59 |
| 298 | 4-Fluorotoluene | 11.70 | 12.29 | 12.32 | -0.59 |
| 299 | Methyl-n-propylketone | 9.93 | 10.54 | 10.66 | -0.61 |
| 300 | Diethylketone | 9.93 | 10.54 | 10.54 | -0.61 |
| 301 | Benz[a]anthracene | 32.86 | 32.24 | 32.26 | 0.62 |
| 302 | Octane | 15.90 | 15.26 | 15.27 | 0.64 |
| 303 | Ethyl sorbate | 17.20 | 16.56 | 16.33 | 0.64 |
| 304 | 3,5-Xylidine | 16.25 | 15.60 | 15.69 | 0.65 |
| 305 | Pyrene | 28.22 | 28.88 | 28.95 | -0.66 |
| 306 | o-Xylene | 14.90 | 14.22 | 14.29 | 0.68 |
| 307 | 1,4-Dimethylbenzene | 14.90 | 14.22 | 14.17 | 0.68 |
| 308 | 1-Chloro-2-methyl-1-propene | 10.80 | 10.09 | 9.99 | 0.71 |
| 309 | Amyl acetate | 14.90 | 14.16 | 14.13 | 0.74 |
| 310 | 2-Methyl-1,3-dioxolane | 9.44 | 10.19 |  | -0.75 |
| 311 | Dixanthylene | 45.27 | 46.02 | 46.87 | -0.75 |
| 312 | Ethylcyclohexane | 15.90 | 15.14 | 15.09 | 0.76 |
| 313 | 3,5-Dichloroaniline | 16.45 | 15.66 | 15.55 | 0.79 |
| 314 | 1,1,1-Trifluoroethane | 4.40 | 5.19 |  | -0.79 |
| 315 | 4-Nitrotoluene | 13.56 | 14.37 | 14.47 | -0.81 |
| 316 | 2,6-Dinitrotoluene | 15.45 | 16.28 | 16.51 | -0.83 |
| 317 | 1,1-Diethoxyethane | 13.20 | 14.03 |  | -0.83 |
| 318 | 2,4-Dimethyl-3-pentanone | 13.50 | 14.38 | 14.92 | -0.88 |
| 319 | Perfluoroethane | 6.82 | 5.92 |  | 0.90 |
| 320 | Aniline | 11.17 | 12.08 | 12.17 | -0.91 |
| 321 | 1-Chloro-2-methylpropane | 11.10 | 10.16 | 10.06 | 0.94 |
| 322 | 4-Tolunitrile | 13.28 | 14.22 | 14.33 | -0.94 |
| 323 | 1-Butylamine | 10.50 | 9.53 | 9.38 | 0.97 |
| 324 | 4-Nitrophenoxybenzene | 24.70 | 23.72 | 23.50 | 0.98 |
| 325 | Refrigerant 115 | 6.30 | 7.29 |  | -0.99 |
| 326 | Fluoranthene | 28.34 | 27.32 | 27.25 | 1.02 |
| 327 | Cyclohexene | 10.70 | 11.72 | 11.75 | -1.02 |
| 328 | Benzamide | 12.86 | 13.89 | 14.31 | -1.03 |
| 329 | Benzo[b]fluorene | 30.21 | 29.12 | 29.03 | 1.09 |
| 330 | 1,4-Benzoquinone | 14.50 | 13.38 | 13.09 | 1.12 |
| 331 | Octafluoronaphthalene | 17.64 | 16.52 | 15.81 | 1.12 |
| 332 | Truxene | 45.55 | 44.42 | 43.57 | 1.13 |
| 333 | 1,2-Diethoxyethane | 11.30 | 12.44 | 12.79 | -1.14 |
| 334 | t-Butylcyclohexane | 19.80 | 18.65 | 18.60 | 1.15 |
| 335 | Triphenylene | 31.07 | 32.24 | 32.39 | -1.17 |
| 336 | Coronene | 42.50 | 43.70 | 44.55 | -1.20 |
| 337 | Benzo[b]naphtho[2,3-d]furan | 29.89 | 28.67 | 28.50 | 1.22 |
| 338 | 2,3-Butandione | 8.20 | 9.42 | 9.73 | -1.22 |
| 339 | 1,4-Pentadiene | 11.49 | 10.24 | 10.20 | 1.25 |
| 340 | 1-Chlorobutane | 11.30 | 10.04 | 9.95 | 1.26 |
| 341 | Amyl formate | 14.20 | 12.78 | 12.26 | 1.42 |
| 342 | Dodecahydrotriphenyl | 29.89 | 31.34 | 31.75 | -1.45 |
| 343 | 1-Chloro-1,1-difluoroethane | 8.05 | 6.56 | 6.28 | 1.49 |
| 344 | 9-Anthracenecarbonitrile | 28.32 | 26.82 | 26.73 | 1.50 |
| 345 | Di-n-butylether | 17.20 | 15.65 | 15.61 | 1.55 |
| 346 | Cyclopentadiene | 8.64 | 10.22 | 10.55 | -1.58 |
| 347 | Phenazine | 23.43 | 21.82 | 19.57 | 1.61 |
| 348 | 2,5-Dimethyl-1,4-benzoquinone | 18.80 | 16.98 | 16.17 | 1.82 |
| 349 | Dibenz[ah]anthracene | 41.31 | 39.42 | 39.09 | 1.89 |
| 350 | Dibenzo[b,e]pyridine | 25.49 | 23.44 | 22.31 | 2.05 |
| 351 | Dibenzo[a,c]phenazine | 33.42 | 36.18 | 38.01 | -2.76 |
