# Supplementary material for: A Generally Applicable Computer Algorithm Based on the Group Additivity Method for the Calculation of Seven Molecular Descriptors: Heat of Combustion, LogPO/W, LogS, Refractivity, Polarizability, Toxicity and LogBB of Organic Compounds; Scope and Limits of Applicability
Source: Molecules. 2015 Oct 7;20(10):18279–351. doi: 10.3390/molecules201018279 (PMC6332030; doi:10.3390/molecules201018279)
Supplement: Supplementary file 1 [file molecules-20-18279-s001.zip › Experimental vs Calculated Refractivity Data Table.doc]

**Experimental vs. Calculated Refractivity Data, sorted by absolute Deviation**

| **No.** | **Molecule name** | **Refractivity** | **Refractivity calc** | **Refractivity test** | **Deviation** |
| --- | --- | --- | --- | --- | --- |
|  |  |  |  |  |  |
| 1 | 1,1,1,2-Tetrachloropropane | 35.49 | 35.49 | 35.48 | 0.00 |
| 2 | 1,1,2-Tribromoethane | 34.51 | 34.51 | 34.57 | 0.00 |
| 3 | Glyme | 24.00 | 24.00 | 24.00 | 0.00 |
| 4 | 1-(2-Furanyl)-1-propanone | 33.91 | 33.91 | 33.91 | 0.00 |
| 5 | 1-(4-Methylphenyl)-1-propanone | 45.96 | 45.96 | 45.98 | 0.00 |
| 6 | 1-Chloro-4-iodobutane | 38.40 | 38.40 | 38.39 | 0.00 |
| 7 | 1-Iodooctane | 52.07 | 52.07 | 52.01 | 0.00 |
| 8 | 1-Piperazineethanamine | 38.47 | 38.47 | 38.49 | 0.00 |
| 9 | 1-Piperidinecarboxaldehyde | 31.67 | 31.67 | 31.50 | 0.00 |
| 10 | 1-Propylamine | 19.40 | 19.40 | 19.41 | 0.00 |
| 11 | 1-Pyrrolidineethanol | 33.04 | 33.04 | 33.01 | 0.00 |
| 12 | 2,3-Dimethyl-2-butanol | 31.24 | 31.24 | 31.28 | 0.00 |
| 13 | 2,3-Dimethyloctane | 48.26 | 48.26 | 48.26 | 0.00 |
| 14 | 2-(2-(2-Butoxyethoxy)ethoxy)ethanol | 54.85 | 54.85 | 54.79 | 0.00 |
| 15 | 2-Bromopentane | 33.05 | 33.05 | 33.06 | 0.00 |
| 16 | 2-Chloroquinoline | 46.95 | 46.95 |  | 0.00 |
| 17 | 2-Ethylnaphthalene | 53.86 | 53.86 | 53.90 | 0.00 |
| 18 | 2-Methoxy-1-propanol | 23.65 | 23.65 | 23.69 | 0.00 |
| 19 | 2-Methoxybenzenemethanamine | 41.42 | 41.42 | 41.42 | 0.00 |
| 20 | 2-Methyl-1-pentanol | 31.18 | 31.18 | 31.19 | 0.00 |
| 21 | 2-Methylbutyric acid | 26.81 | 26.81 | 26.81 | 0.00 |
| 22 | 2-Octanamine | 42.52 | 42.52 | 42.55 | 0.00 |
| 23 | 3,3-Dimethyloxetane | 24.84 | 24.84 | 24.84 | 0.00 |
| 24 | 3-Buten-1-ol | 21.77 | 21.77 | 21.78 | 0.00 |
| 25 | 3-Penten-2-ol | 26.61 | 26.61 | 26.61 | 0.00 |
| 26 | 4,4-Dimethyl-1-pentyne | 32.52 | 32.52 | 32.57 | 0.00 |
| 27 | 4-Chloronitrobenzene | 37.95 | 37.95 | 37.91 | 0.00 |
| 28 | 5-Methyl-1-hexyne | 32.47 | 32.47 | 32.50 | 0.00 |
| 29 | 5-Methylnonane | 48.29 | 48.29 | 48.29 | 0.00 |
| 30 | beta-Vetivone | 67.53 | 67.53 | 67.51 | 0.00 |
| 31 | 4-Bromo-1,2-butadiene | 28.58 | 28.58 | 28.59 | 0.00 |
| 32 | Chloroacetonitrile | 16.02 | 16.02 | 16.08 | 0.00 |
| 33 | cis-2-Methylcyclohexanol | 33.66 | 33.66 | 33.66 | 0.00 |
| 34 | Cyclohexane | 27.72 | 27.72 | 27.72 | 0.00 |
| 35 | Dibutyl adipate | 70.40 | 70.40 | 70.36 | 0.00 |
| 36 | Dimethyldiphenoxysilane | 71.56 | 71.56 | 71.59 | 0.00 |
| 37 | 3-(Triethoxysilyl)-1-propanamine | 59.24 | 59.24 | 59.23 | 0.00 |
| 38 | Glycerol 1,3-dinitrate | 33.45 | 33.45 | 33.48 | 0.00 |
| 39 | 2,4,6,8,10,12-Hexamethylcyclohexasiloxane | 85.86 | 85.86 | 85.80 | 0.00 |
| 40 | Hexanenitrile | 29.69 | 29.69 | 29.69 | 0.00 |
| 41 | Menthyl isovalerate | 70.96 | 70.96 | 70.93 | 0.00 |
| 42 | Methylcyanoacetate | 22.23 | 22.23 | 22.26 | 0.00 |
| 43 | N-Nitrosopiperidine | 31.22 | 31.22 | 31.23 | 0.00 |
| 44 | Propyl 3-methylbutanoate | 40.85 | 40.85 | 40.84 | 0.00 |
| 45 | Trichloroacetaldehyde diethylacetal | 47.80 | 47.80 | 47.82 | 0.00 |
| 46 | Triethylamine | 33.79 | 33.79 | 33.78 | 0.00 |
| 47 | Ethyl laurate | 68.61 | 68.60 | 68.71 | 0.01 |
| 48 | 3-Methyl-1,2-butadiene | 25.34 | 25.33 | 25.33 | 0.01 |
| 49 | 1,6-Dichlorohexane | 39.45 | 39.46 | 39.46 | -0.01 |
| 50 | 1-Bromo-4-chlorobutane | 33.21 | 33.22 | 33.24 | -0.01 |
| 51 | 1-Bromooctane | 46.90 | 46.89 | 46.88 | 0.01 |
| 52 | 1-Chloro-2-methyl-1-propene | 25.06 | 25.07 | 24.98 | -0.01 |
| 53 | 1-Dodecyne | 55.61 | 55.60 | 55.62 | 0.01 |
| 54 | 1-Iodo-2,4-dimethylbenzene | 48.81 | 48.82 | 48.85 | -0.01 |
| 55 | 2,3,4-Trimethylhexane | 43.62 | 43.61 | 43.61 | 0.01 |
| 56 | 2,4-Dimethyl-1-pentene | 34.18 | 34.17 | 34.18 | 0.01 |
| 57 | 2,5-Dimethyl-2-hexene | 39.04 | 39.03 | 39.03 | 0.01 |
| 58 | 2-Chloro-2,4,4-trimethylpentane | 43.99 | 43.98 | 44.04 | 0.01 |
| 59 | 2-Chloropyridine | 29.20 | 29.19 |  | 0.01 |
| 60 | Isophorol | 42.94 | 42.93 | 42.90 | 0.01 |
| 61 | 3-Pyridinemethanamine | 32.48 | 32.49 | 32.46 | -0.01 |
| 62 | 4-(1-Ethylpropyl)pyridine | 47.53 | 47.52 | 47.53 | 0.01 |
| 63 | 4-Morpholineethanamine | 36.80 | 36.81 | 36.82 | -0.01 |
| 64 | 4-Pyridineethanamine | 37.10 | 37.11 | 37.11 | -0.01 |
| 65 | Acetylcyanide | 16.28 | 16.29 | 16.34 | -0.01 |
| 66 | alpha-Methyl-4-morpholineethanol | 39.37 | 39.36 | 39.40 | 0.01 |
| 67 | alpha-Naphthaleneazide | 53.00 | 53.01 | 53.03 | -0.01 |
| 68 | Benzophenone | 56.69 | 56.68 | 56.65 | 0.01 |
| 69 | Cyclopentanecarboxylic acid | 29.32 | 29.31 | 29.31 | 0.01 |
| 70 | Dichlorodimethylsilane | 29.65 | 29.66 | 29.71 | -0.01 |
| 71 | Diethoxymethylphenylsilane | 60.85 | 60.84 | 60.80 | 0.01 |
| 72 | Ethyl trifluoroacetate | 22.80 | 22.81 | 22.81 | -0.01 |
| 73 | Isobutryic anhydride | 40.76 | 40.75 | 40.63 | 0.01 |
| 74 | Isobutyl nitrate | 28.62 | 28.63 | 28.64 | -0.01 |
| 75 | Isopentyl isopentanoate | 50.05 | 50.06 | 50.05 | -0.01 |
| 76 | Isopropyl butanoate | 36.23 | 36.24 | 36.22 | -0.01 |
| 77 | Methacrylic acid | 21.97 | 21.96 | 21.92 | 0.01 |
| 78 | Diethyl methylphosphonate | 36.23 | 36.24 | 36.10 | -0.01 |
| 79 | Methyl 5-bromopentanoate | 39.42 | 39.43 | 39.42 | -0.01 |
| 80 | Methyl heptafluorobutanoate | 28.32 | 28.31 | 28.32 | 0.01 |
| 81 | n-Butylmethacrylate | 40.61 | 40.62 | 40.66 | -0.01 |
| 82 | n-Decylacetate | 59.35 | 59.36 | 59.34 | -0.01 |
| 83 | N-Ethyl-2-methylaniline | 45.13 | 45.14 | 45.09 | -0.01 |
| 84 | n-Heptylformate | 41.03 | 41.04 | 41.03 | -0.01 |
| 85 | n-Propylnitrate | 24.03 | 24.04 | 24.03 | -0.01 |
| 86 | Oxiranecarboxaldehyde | 16.21 | 16.22 | 16.18 | -0.01 |
| 87 | Pelletierine | 40.81 | 40.82 | 40.78 | -0.01 |
| 88 | Phenyldichlorofluoromethane | 41.29 | 41.28 | 41.28 | 0.01 |
| 89 | s-Butylformate | 27.15 | 27.16 | 27.14 | -0.01 |
| 90 | Tetrahydrofurfuryl alcohol | 26.18 | 26.17 | 26.13 | 0.01 |
| 91 | trans-2-Hexene | 29.87 | 29.88 | 29.88 | -0.01 |
| 92 | Vinylcyclopentane | 32.10 | 32.11 | 32.11 | -0.01 |
| 93 | 2,2,2-Trifluoroethanol | 13.13 | 13.14 | 13.15 | -0.01 |
| 94 | Dimethylacetal | 23.79 | 23.80 | 23.81 | -0.01 |
| 95 | Vinylacetylene | 18.43 | 18.44 | 18.47 | -0.01 |
| 96 | 2-Bromoethanol | 20.55 | 20.54 | 20.52 | 0.01 |
| 97 | 2-Ethyl-1-butanol | 31.19 | 31.18 | 31.16 | 0.01 |
| 98 | 2-Iodoethanol | 25.73 | 25.72 | 25.74 | 0.01 |
| 99 | 2-Nitro-1-butanol | 27.65 | 27.64 | 27.67 | 0.01 |
| 100 | 3-Aminopropionitrile | 19.26 | 19.25 | 19.26 | 0.01 |
| 101 | Isovaleronitrile | 25.05 | 25.04 | 25.05 | 0.01 |
| 102 | 3-Methylpentane | 29.80 | 29.81 | 29.85 | -0.01 |
| 103 | cis-1,3-Dichloro-2-butene | 30.24 | 30.23 | 30.21 | 0.01 |
| 104 | Cyclopentanecarbonitrile | 27.55 | 27.54 | 27.55 | 0.01 |
| 105 | Diethylperoxide | 24.83 | 24.82 | 24.84 | 0.01 |
| 106 | Diethyl sulfate | 31.81 | 31.80 | 31.83 | 0.01 |
| 107 | Ethylene-1,2-diamine | 18.21 | 18.20 | 18.22 | 0.01 |
| 108 | Isobutyl acetate | 31.62 | 31.61 | 31.59 | 0.01 |
| 109 | Methanesulfonylchloride | 21.08 | 21.07 | 20.98 | 0.01 |
| 110 | N-Nitrosopyrrolidine | 26.59 | 26.60 | 26.58 | -0.01 |
| 111 | Propyl isothiocyanate | 30.86 | 30.87 | 30.92 | -0.01 |
| 112 | 1(3H)-Isobenzofuranone | 35.95 | 35.94 | 35.95 | 0.01 |
| 113 | 1,2-Dimethylcyclopentene | 32.27 | 32.26 | 32.26 | 0.01 |
| 114 | 1-Nonylnaphthalene | 86.19 | 86.20 | 86.23 | -0.01 |
| 115 | 1-Undecyne | 50.97 | 50.98 | 51.00 | -0.01 |
| 116 | 2-(1-Iodoethyl)-1,3-dioxolane-4-methanol | 45.54 | 45.55 | 45.49 | -0.01 |
| 117 | 2-Methyl-1-hexanol | 35.79 | 35.80 | 35.80 | -0.01 |
| 118 | 3-Bromopentane | 33.06 | 33.05 | 33.08 | 0.01 |
| 119 | Benzoyl chloride | 37.15 | 37.16 | 37.17 | -0.01 |
| 120 | beta-Terpineol | 47.33 | 47.34 | 47.33 | -0.01 |
| 121 | cis-1,2-Di-t-butylethene | 48.41 | 48.40 | 48.42 | 0.01 |
| 122 | cis-3-Octene | 39.13 | 39.12 | 39.12 | 0.01 |
| 123 | Dodecamethylcyclohexasiloxane | 111.89 | 111.90 | 112.08 | -0.01 |
| 124 | Ethyl octanoate | 50.11 | 50.12 | 50.11 | -0.01 |
| 125 | Hexadecylbenzene | 100.79 | 100.78 | 100.79 | 0.01 |
| 126 | Propyl 3-chloropropanoate | 36.44 | 36.45 | 36.47 | -0.01 |
| 127 | Triethyleneglycol | 36.01 | 36.02 | 36.04 | -0.01 |
| 128 | 1-Bromodecane | 56.15 | 56.13 | 56.15 | 0.02 |
| 129 | 1-Decylnaphthalene | 90.84 | 90.82 | 90.87 | 0.02 |
| 130 | 1-Fluorooctane | 39.02 | 39.04 | 39.05 | -0.02 |
| 131 | 1-Methyl-4-(phenylthio)benzene | 64.25 | 64.27 | 64.37 | -0.02 |
| 132 | 2-Ethyl-1-hexanol | 40.44 | 40.42 | 40.41 | 0.02 |
| 133 | 3-Nonanol | 45.07 | 45.05 | 45.05 | 0.02 |
| 134 | 5-(Chloromethyl)-1,3-benzodioxole | 42.42 | 42.44 | 42.44 | -0.02 |
| 135 | 6-Methyl-2-heptanone | 39.49 | 39.51 | 39.51 | -0.02 |
| 136 | Brassidic acid | 105.44 | 105.42 | 105.42 | 0.02 |
| 137 | cis-5-Methyl-2-hexene | 34.45 | 34.47 | 34.48 | -0.02 |
| 138 | Diethyl maleate | 42.70 | 42.72 | 42.70 | -0.02 |
| 139 | Diphenylmethylsilane | 65.28 | 65.30 | 65.34 | -0.02 |
| 140 | Ethyl 1-piperidinepropanoate | 51.97 | 51.95 | 51.93 | 0.02 |
| 141 | Heptadecylbenzene | 105.42 | 105.40 | 105.41 | 0.02 |
| 142 | Methyl 2-octynoate | 44.44 | 44.42 | 44.47 | 0.02 |
| 143 | Pentadecylbenzene | 96.14 | 96.16 | 96.16 | -0.02 |
| 144 | trans-5-Undecene | 53.00 | 52.98 | 53.05 | 0.02 |
| 145 | Methyl trichloroacetate | 32.50 | 32.48 | 32.51 | 0.02 |
| 146 | Triisopropylphosphite | 56.74 | 56.76 | 56.85 | -0.02 |
| 147 | Vinylethoxydimethylsilane | 39.82 | 39.80 | 39.80 | 0.02 |
| 148 | Methylcyclopentylether | 29.42 | 29.40 | 29.40 | 0.02 |
| 149 | Benzophenone-N-methylimine | 63.74 | 63.76 | 63.77 | -0.02 |
| 150 | 1,1,3-Trichloropropane | 30.44 | 30.46 | 30.44 | -0.02 |
| 151 | 1,2-Dibromoethane | 27.00 | 26.98 | 27.02 | 0.02 |
| 152 | 1,2-Dichloro-1-fluoroethane | 21.12 | 21.10 | 21.11 | 0.02 |
| 153 | 1-Hexen-3-ol | 31.01 | 30.99 | 30.98 | 0.02 |
| 154 | N-Methylpiperidine | 31.75 | 31.73 | 31.74 | 0.02 |
| 155 | 1-Penten-3-yne | 23.98 | 23.96 | 23.99 | 0.02 |
| 156 | 2-(Chloromethyl)-2-methyloxirane | 25.05 | 25.07 | 25.10 | -0.02 |
| 157 | 2-Methyl-2-butanamine | 28.61 | 28.59 | 28.59 | 0.02 |
| 158 | 3-Butyn-1-ol | 19.99 | 20.01 | 19.99 | -0.02 |
| 159 | 3-Methylsulfolane | 31.93 | 31.91 | 31.94 | 0.02 |
| 160 | 4-Amino-1-butanol | 25.37 | 25.39 | 25.39 | -0.02 |
| 161 | Diethylmethylamine | 29.25 | 29.23 | 29.25 | 0.02 |
| 162 | Ethyl-n-propylether | 26.96 | 26.94 | 26.94 | 0.02 |
| 163 | Ethyl propionate | 27.00 | 27.02 | 27.00 | -0.02 |
| 164 | Thietane | 21.75 | 21.73 | 21.75 | 0.02 |
| 165 | Pentanoic acid | 26.86 | 26.84 | 26.83 | 0.02 |
| 166 | 3-Methylbutanaloxime | 29.64 | 29.66 | 29.82 | -0.02 |
| 167 | 1,2-Dimethylcyclohexene | 36.86 | 36.88 | 36.98 | -0.02 |
| 168 | 1,2-Propanediol diacetate | 38.06 | 38.04 | 37.99 | 0.02 |
| 169 | 1,8-Dichlorooctane | 48.68 | 48.70 | 48.68 | -0.02 |
| 170 | 1-Iodoheptane | 47.43 | 47.45 | 47.47 | -0.02 |
| 171 | 2,3-Dimethylheptane | 43.66 | 43.64 | 43.64 | 0.02 |
| 172 | 2,4-Dimethyl-3-isopropylpentane | 48.18 | 48.20 | 48.20 | -0.02 |
| 173 | 2-Hexanethiol | 37.72 | 37.74 | 37.84 | -0.02 |
| 174 | 3-Iodotoluene | 44.06 | 44.04 | 44.02 | 0.02 |
| 175 | 3-Methyl-2-hexanol | 35.76 | 35.78 | 35.78 | -0.02 |
| 176 | 6-Methyl-2-heptanol | 40.42 | 40.40 | 40.40 | 0.02 |
| 177 | Allylcyclopentane | 36.71 | 36.73 | 36.74 | -0.02 |
| 178 | Carvenone | 46.60 | 46.58 | 46.57 | 0.02 |
| 179 | Di-n-butylamine | 42.44 | 42.46 | 42.46 | -0.02 |
| 180 | Ethyl 2-acetylhexanoate | 50.53 | 50.55 | 50.56 | -0.02 |
| 181 | Hexyl methacrylate | 49.88 | 49.86 | 49.87 | 0.02 |
| 182 | 3-Methylbutanoic acid | 26.79 | 26.81 | 26.81 | -0.02 |
| 183 | Methyl crotonate | 27.06 | 27.04 | 27.02 | 0.02 |
| 184 | n-Butyltrichloroacetate | 46.38 | 46.36 | 46.33 | 0.02 |
| 185 | s-Butyl ethyl ether | 31.56 | 31.54 | 31.54 | 0.02 |
| 186 | 2,4,6,8,10-Pentamethylcyclopentasiloxane | 71.57 | 71.55 | 71.50 | 0.02 |
| 187 | Diethyl dibutylmalonate | 75.02 | 75.04 | 75.11 | -0.02 |
| 188 | Octamethyltrisiloxane | 67.44 | 67.46 | 67.49 | -0.02 |
| 189 | Octadecyltrichlorosilane | 108.02 | 107.99 | 107.98 | 0.03 |
| 190 | 1-Iodo-2,2-dimethylpropane | 38.26 | 38.23 | 38.21 | 0.03 |
| 191 | 1-Tridecene | 62.01 | 61.98 | 61.99 | 0.03 |
| 192 | 2,2-Dimethyl-3-pentanol | 35.80 | 35.83 | 35.84 | -0.03 |
| 193 | 2-Phenoxy-1-propanol | 43.80 | 43.83 | 43.79 | -0.03 |
| 194 | 3-Bromo-3-methylpentane | 37.66 | 37.69 | 37.81 | -0.03 |
| 195 | Cyclohexaneacetic acid | 38.58 | 38.55 | 38.55 | 0.03 |
| 196 | Ethyl 2-methylbenzoate | 47.33 | 47.30 | 47.33 | 0.03 |
| 197 | Isobutyl methacrylate | 40.62 | 40.59 | 40.58 | 0.03 |
| 198 | Amyl acetate | 36.23 | 36.26 | 36.25 | -0.03 |
| 199 | Octylcyanide | 43.58 | 43.55 | 43.55 | 0.03 |
| 200 | Pentyl salicylate | 58.15 | 58.12 | 58.09 | 0.03 |
| 201 | trans-3-Octene | 39.09 | 39.12 | 39.12 | -0.03 |
| 202 | N-Benzyl-2-methylaniline | 65.29 | 65.26 | 65.28 | 0.03 |
| 203 | 1-Chloro-2-butanol | 26.79 | 26.76 | 26.75 | 0.03 |
| 204 | 3-Methoxy-1-butanol | 28.24 | 28.27 | 28.27 | -0.03 |
| 205 | Butyric acid | 22.19 | 22.22 | 22.19 | -0.03 |
| 206 | Cyclopentane | 23.13 | 23.10 | 23.10 | 0.03 |
| 207 | Ethyl chlorofluoroacetate | 27.36 | 27.33 | 27.33 | 0.03 |
| 208 | Fumaric acid dichloride | 31.97 | 32.00 | 32.06 | -0.03 |
| 209 | Methyl chlorocarbonate | 18.18 | 18.15 | 18.18 | 0.03 |
| 210 | t-Butylnitrite | 26.77 | 26.74 | 26.74 | 0.03 |
| 211 | Tiglic aldehyde | 25.83 | 25.86 | 25.88 | -0.03 |
| 212 | (2-Bromoethyl)cyclohexane | 44.71 | 44.74 | 44.76 | -0.03 |
| 213 | (Bromomethyl)trimethylsilane | 38.09 | 38.06 |  | 0.03 |
| 214 | 1,1,1,3,5,7,7,7-Octamethyltetrasiloxane | 77.46 | 77.43 | 77.46 | 0.03 |
| 215 | Dimethyl phthalate | 49.09 | 49.06 | 49.08 | 0.03 |
| 216 | 1,3-Diisopropylbenzene | 54.65 | 54.68 | 54.72 | -0.03 |
| 217 | 1,4-Dichlorobutane | 30.25 | 30.22 | 30.20 | 0.03 |
| 218 | 1,8-Dichloronaphthalene | 53.81 | 53.78 | 53.82 | 0.03 |
| 219 | 1-Bromo-4-chlorobenzene | 38.88 | 38.85 | 38.83 | 0.03 |
| 220 | Heptyl bromide | 42.24 | 42.27 | 42.26 | -0.03 |
| 221 | Hexyl bromide | 37.62 | 37.65 | 37.67 | -0.03 |
| 222 | 1-Chloro-2-nitro-4-(trifluoromethyl)benzene | 43.11 | 43.14 | 43.19 | -0.03 |
| 223 | 1-Chloropropane | 20.82 | 20.79 | 20.79 | 0.03 |
| 224 | Acetylhexane | 37.42 | 37.39 | 37.39 | 0.03 |
| 225 | 1-Dodecene | 57.33 | 57.36 | 57.45 | -0.03 |
| 226 | 1-Fluorodecane | 48.31 | 48.28 | 48.28 | 0.03 |
| 227 | 1-Fluoroheptane | 34.39 | 34.42 | 34.42 | -0.03 |
| 228 | 1-Naphthol | 46.23 | 46.20 | 46.14 | 0.03 |
| 229 | 1-Phenylnaphthalene | 69.33 | 69.36 | 69.40 | -0.03 |
| 230 | 2,2,4-Trimethyl-3-pentanol | 40.45 | 40.42 | 40.42 | 0.03 |
| 231 | 2,3-Dibromobutane | 36.29 | 36.26 | 36.26 | 0.03 |
| 232 | 2-(2-Chloroethoxy)ethanol | 28.53 | 28.50 | 28.47 | 0.03 |
| 233 | 2-(2-Pyridyl)ethanol | 35.23 | 35.20 | 35.22 | 0.03 |
| 234 | 2-Bromo-1-phenyl-1-propanone | 49.04 | 49.01 | 49.02 | 0.03 |
| 235 | Isobutyronitrile | 20.39 | 20.42 | 20.47 | -0.03 |
| 236 | 2-Iodooctane | 52.35 | 52.32 | 52.32 | 0.03 |
| 237 | 2-Iodotoluene | 44.01 | 44.04 | 44.10 | -0.03 |
| 238 | 2-Methyl-2-propenol | 21.68 | 21.71 | 21.71 | -0.03 |
| 239 | 3-Methyl-1-butyne | 23.26 | 23.23 | 23.29 | 0.03 |
| 240 | 3-Methyl-2-oxobutanoic acid | 27.30 | 27.27 | 27.26 | 0.03 |
| 241 | 3-Methyl-2-pentyl-2-cyclopenten-1-one | 51.23 | 51.20 | 51.22 | 0.03 |
| 242 | 3-Methyl-6-isopropyl-2-cyclohexen-1-ol | 47.44 | 47.47 | 47.46 | -0.03 |
| 243 | 3-Methylbutyl nitrate | 33.28 | 33.25 | 33.23 | 0.03 |
| 244 | 4-Decanol | 49.70 | 49.67 | 49.67 | 0.03 |
| 245 | 4-Penten-1-ol | 26.36 | 26.39 | 26.38 | -0.03 |
| 246 | 1,2,2-Trimethylpropylacetate | 40.85 | 40.88 | 40.90 | -0.03 |
| 247 | alpha-Irone | 65.09 | 65.12 | 65.12 | -0.03 |
| 248 | Benzylbromide | 39.32 | 39.29 | 39.31 | 0.03 |
| 249 | Diethyleneglycol monobutyl ether | 43.92 | 43.89 | 43.89 | 0.03 |
| 250 | Carbitol acetate | 44.29 | 44.32 | 44.32 | -0.03 |
| 251 | cis-1-Ethyl-2-methylcyclopentane | 36.87 | 36.90 | 36.90 | -0.03 |
| 252 | cis-3-Methyl-2-heptene | 39.09 | 39.06 | 39.08 | 0.03 |
| 253 | cis-4-Methyl-2-hexene | 34.44 | 34.47 | 34.45 | -0.03 |
| 254 | DTBP | 43.45 | 43.42 | 43.44 | 0.03 |
| 255 | Dodecamethylpentasiloxane | 104.79 | 104.76 | 104.85 | 0.03 |
| 256 | Ethyl 3-cyclohexene-1-carboxylate | 43.42 | 43.39 | 43.39 | 0.03 |
| 257 | Ethyl 4-methyl-3-oxopentanoate | 41.28 | 41.31 | 41.30 | -0.03 |
| 258 | Ethyl isothiocyanate | 26.22 | 26.25 | 26.25 | -0.03 |
| 259 | Hexyl hexanoate | 59.39 | 59.36 | 59.34 | 0.03 |
| 260 | Isobutyldimethylamine | 33.85 | 33.88 | 33.97 | -0.03 |
| 261 | Isopropyl propanoate | 31.59 | 31.62 | 31.61 | -0.03 |
| 262 | Isopropylacetate | 26.97 | 27.00 | 27.02 | -0.03 |
| 263 | Isopropylcyclohexane | 41.55 | 41.52 | 41.52 | 0.03 |
| 264 | Butyl butanoate | 40.85 | 40.88 | 40.93 | -0.03 |
| 265 | n-Hexylamine | 33.29 | 33.26 | 33.28 | 0.03 |
| 266 | N-Phenylpiperidine | 52.42 | 52.39 | 52.42 | 0.03 |
| 267 | 3-(1-Methyl-2-pyrrolidinyl)pyridine | 49.49 | 49.46 | 49.44 | 0.03 |
| 268 | Nitrocyclohexane | 33.42 | 33.39 | 33.38 | 0.03 |
| 269 | Octahydroindolizine | 38.84 | 38.81 | 38.76 | 0.03 |
| 270 | Oleyl alcohol | 86.72 | 86.69 | 86.66 | 0.03 |
| 271 | Pentyl hexanoate | 54.77 | 54.74 | 54.75 | 0.03 |
| 272 | Pentyl pentanoate | 50.09 | 50.12 | 50.12 | -0.03 |
| 273 | Propyl trichloroacetate | 41.77 | 41.74 | 41.71 | 0.03 |
| 274 | Pyridazine | 22.13 | 22.16 |  | -0.03 |
| 275 | Benzenethiol | 34.47 | 34.50 | 34.58 | -0.03 |
| 276 | trans-5-Decene | 48.39 | 48.36 | 48.34 | 0.03 |
| 277 | Trimethoxyborine | 24.86 | 24.89 | 24.93 | -0.03 |
| 278 | 1,3-Bis(dichloromethyl)tetramethyldisiloxane | 68.07 | 68.11 |  | -0.04 |
| 279 | Dodecyloxirane | 66.34 | 66.38 | 66.38 | -0.04 |
| 280 | Elaidic acid | 86.90 | 86.94 | 86.96 | -0.04 |
| 281 | Ethyl ricinoleate | 97.75 | 97.71 | 97.68 | 0.04 |
| 282 | Dicyclohexyl | 53.22 | 53.26 | 53.26 | -0.04 |
| 283 | 1,2,3-Tribromobutane | 44.03 | 44.07 | 44.01 | -0.04 |
| 284 | 1,2,3-Trichloropropane | 30.43 | 30.39 | 30.40 | 0.04 |
| 285 | 1,2-Diisobutylhydrazine | 46.34 | 46.30 | 46.32 | 0.04 |
| 286 | 1,3-Diethylbenzene | 45.54 | 45.50 | 45.48 | 0.04 |
| 287 | 1,4-Diethylbenzene | 45.54 | 45.50 | 45.48 | 0.04 |
| 288 | 1-(Bromomethyl)-3-methylbenzene | 44.03 | 44.07 | 44.09 | -0.04 |
| 289 | 1-(Chloromethyl)-3-methylbenzene | 41.11 | 41.07 | 41.08 | 0.04 |
| 290 | 1-Chloro-4-ethylbenzene | 40.72 | 40.76 | 40.75 | -0.04 |
| 291 | 1-Decyne | 46.32 | 46.36 | 46.38 | -0.04 |
| 292 | Isobutyl iodide | 33.52 | 33.56 | 33.59 | -0.04 |
| 293 | Caprylonitrile | 38.97 | 38.93 | 38.99 | 0.04 |
| 294 | 1-Undecene | 52.70 | 52.74 | 52.75 | -0.04 |
| 295 | 2,2-Dimethyl-1-butanol | 31.27 | 31.23 | 31.24 | 0.04 |
| 296 | 2,3-Dimethylbutane | 29.82 | 29.78 | 29.78 | 0.04 |
| 297 | 2,5-Dibromothiophene | 40.13 | 40.09 |  | 0.04 |
| 298 | 2,5-Dimethylthiophene | 34.23 | 34.27 | 34.23 | -0.04 |
| 299 | 2-(Methylthio)aniline | 44.24 | 44.20 | 44.19 | 0.04 |
| 300 | 2-Ethylhexyl acetate | 50.05 | 50.09 | 50.14 | -0.04 |
| 301 | 2-Hexenoic acid | 31.54 | 31.50 | 31.51 | 0.04 |
| 302 | 2-Isopropylnaphthalene | 58.49 | 58.45 | 58.50 | 0.04 |
| 303 | 2-Butylmercaptan | 28.46 | 28.50 | 28.53 | -0.04 |
| 304 | 2-Methylbutyl acrylate | 40.61 | 40.65 | 40.63 | -0.04 |
| 305 | 3,3-Dimethylheptane | 43.68 | 43.72 | 43.71 | -0.04 |
| 306 | 3,5-Lutidine | 33.81 | 33.85 | 33.89 | -0.04 |
| 307 | 3,6-Dimethyl-3-octanol | 49.68 | 49.72 | 49.69 | -0.04 |
| 308 | 3-Bromopropyne | 21.87 | 21.83 | 21.90 | 0.04 |
| 309 | 3-Chloroaniline | 35.43 | 35.39 | 35.38 | 0.04 |
| 310 | 3-Chloroanisole | 37.84 | 37.88 | 37.92 | -0.04 |
| 311 | 3-Pentanamine | 28.62 | 28.66 | 28.66 | -0.04 |
| 312 | 4-Methoxybenzenemethanamine | 41.38 | 41.42 | 41.45 | -0.04 |
| 313 | Methyl isobutyl ketone | 30.23 | 30.27 | 30.29 | -0.04 |
| 314 | Acetic anhydride | 22.37 | 22.33 | 22.33 | 0.04 |
| 315 | Acrylic acid | 17.44 | 17.40 | 17.42 | 0.04 |
| 316 | alpha-Methylcyclohexanemethanol | 38.24 | 38.28 | 38.28 | -0.04 |
| 317 | Cycloheptanol | 33.65 | 33.69 | 33.68 | -0.04 |
| 318 | Cyclohexaneethanol | 38.34 | 38.30 | 38.29 | 0.04 |
| 319 | Diethylcarbonate | 28.53 | 28.49 | 28.38 | 0.04 |
| 320 | Ethyl trans-2-methyl-2-butenoate | 36.28 | 36.24 | 36.25 | 0.04 |
| 321 | Ethyl undecanoate | 63.94 | 63.98 | 63.96 | -0.04 |
| 322 | Fulvene | 27.50 | 27.54 | 27.55 | -0.04 |
| 323 | Isopentyl propanoate | 40.81 | 40.85 | 40.86 | -0.04 |
| 324 | Methylcyclohexylether | 34.06 | 34.02 | 34.00 | 0.04 |
| 325 | Morpholine | 23.62 | 23.58 | 23.57 | 0.04 |
| 326 | N,N,N',N'-Tertramethylethylenediamine | 38.02 | 37.98 | 38.08 | 0.04 |
| 327 | N,N-Dimethyl-1,2-ethanediamine | 28.13 | 28.09 | 28.12 | 0.04 |
| 328 | N,N-Dimethylformamide | 20.01 | 20.05 | 19.84 | -0.04 |
| 329 | 1-Iodohexane | 42.79 | 42.83 | 42.84 | -0.04 |
| 330 | Neopentanoic acid | 31.52 | 31.48 | 31.46 | 0.04 |
| 331 | 3-Chloropropyne | 18.87 | 18.83 | 18.79 | 0.04 |
| 332 | Propyl 4-toluenesulfonate | 55.07 | 55.11 | 55.11 | -0.04 |
| 333 | Quinaldine | 46.93 | 46.97 | 46.99 | -0.04 |
| 334 | Thiamorpholine | 29.69 | 29.73 | 29.74 | -0.04 |
| 335 | Triethylphosphite | 43.00 | 42.96 | 42.89 | 0.04 |
| 336 | 1-Bromo-6-fluorohexane | 37.57 | 37.61 | 37.63 | -0.04 |
| 337 | Formic acid | 8.56 | 8.52 | 8.50 | 0.04 |
| 338 | Propargyl alcohol | 15.35 | 15.39 | 15.40 | -0.04 |
| 339 | 1-Chloro-4-fluorobutane | 25.33 | 25.37 | 25.31 | -0.04 |
| 340 | 2,6-Dimethyl-4-ethylpyridine | 43.49 | 43.53 | 43.41 | -0.04 |
| 341 | 1-Chloro-2-fluoroethane | 16.17 | 16.13 | 16.12 | 0.04 |
| 342 | 3-Butyn-2-one | 19.06 | 19.10 | 19.09 | -0.04 |
| 343 | 3-Methylpentanoic acid | 31.47 | 31.43 | 31.44 | 0.04 |
| 344 | 5-Chloropentanoic acid | 31.61 | 31.65 | 31.65 | -0.04 |
| 345 | trans-2-Pentene | 25.22 | 25.26 | 25.27 | -0.04 |
| 346 | 1,3-Bis(bromomethyl)tetramethyldisiloxane | 64.41 | 64.45 |  | -0.04 |
| 347 | 1-Bromononane | 51.55 | 51.51 | 51.58 | 0.04 |
| 348 | 1-Iodononane | 56.73 | 56.69 | 56.70 | 0.04 |
| 349 | 2-(1-Ethylpropyl)pyridine | 47.62 | 47.66 | 47.65 | -0.04 |
| 350 | Benzylsalicylate | 64.42 | 64.38 | 64.36 | 0.04 |
| 351 | Di-n-butylsulfite | 50.51 | 50.55 | 50.59 | -0.04 |
| 352 | alpha,alpha-Dichloroacetophenone | 46.19 | 46.23 | 46.17 | -0.04 |
| 353 | Dimethyldiphenylsilane | 70.04 | 70.00 | 70.16 | 0.04 |
| 354 | Tetradecamethylhexasiloxane | 123.45 | 123.41 | 123.41 | 0.04 |
| 355 | Tetradecylbenzene | 91.50 | 91.54 | 91.53 | -0.04 |
| 356 | Tetraethyleneglycol | 46.94 | 46.98 | 46.93 | -0.04 |
| 357 | Tris(4-bromophenyl)amine | 103.16 | 103.20 |  | -0.04 |
| 358 | 1,1,1,3,5,5,5-Heptamethyltrisiloxane | 63.17 | 63.12 | 63.15 | 0.05 |
| 359 | 1,5-Dichloropentane | 34.89 | 34.84 | 34.82 | 0.05 |
| 360 | 1-Bromo-2-methylbutane | 32.95 | 33.00 | 33.00 | -0.05 |
| 361 | 1-Bromopentane | 32.98 | 33.03 | 33.04 | -0.05 |
| 362 | 1-Butylnaphthalene | 63.15 | 63.10 | 63.10 | 0.05 |
| 363 | 1-Octadecene | 85.13 | 85.08 | 85.11 | 0.05 |
| 364 | 1-Pentadecene | 71.27 | 71.22 | 71.23 | 0.05 |
| 365 | 1-Phenoxy-2-propanol | 43.78 | 43.83 | 43.84 | -0.05 |
| 366 | 2,3,3-Trimethylhexane | 43.64 | 43.69 | 43.69 | -0.05 |
| 367 | 2,3,5-Trimethylhexane | 43.56 | 43.61 | 43.64 | -0.05 |
| 368 | 2,3-Dihydro-1-methyl-1H-indene | 43.30 | 43.35 | 43.33 | -0.05 |
| 369 | 2-Bromothiophene | 32.53 | 32.48 |  | 0.05 |
| 370 | 2-Chlorohexane | 34.68 | 34.63 | 34.64 | 0.05 |
| 371 | 2-Ethylhexanal | 39.66 | 39.71 | 39.68 | -0.05 |
| 372 | 4,5-Dihydro-1H-pyrazole | 19.51 | 19.56 | 19.69 | -0.05 |
| 373 | 2-Tridecanone | 62.59 | 62.64 | 62.64 | -0.05 |
| 374 | 3-Ethyl-1-pentene | 34.28 | 34.23 | 34.21 | 0.05 |
| 375 | 3-Methyl-1-phenyl-1-butanone | 50.34 | 50.39 | 50.40 | -0.05 |
| 376 | 4-Chloro-N-methylaniline | 40.51 | 40.46 | 40.42 | 0.05 |
| 377 | 5-Ethyl-1,3-dimethylbenzene | 45.71 | 45.66 | 45.63 | 0.05 |
| 378 | Butyl citrate | 92.16 | 92.11 | 92.03 | 0.05 |
| 379 | Methyl caprate | 54.67 | 54.72 | 54.71 | -0.05 |
| 380 | cis-1,2-Dimethylcyclohexane | 36.85 | 36.90 | 36.92 | -0.05 |
| 381 | Cyclohexyl methacrylate | 47.67 | 47.72 | 47.75 | -0.05 |
| 382 | Decyl decanoate | 96.37 | 96.32 | 96.30 | 0.05 |
| 383 | Diethyl 1,1-cyclobutanedicarboxylate | 49.77 | 49.82 | 49.83 | -0.05 |
| 384 | Diisobutyl carbonate | 46.96 | 46.91 | 46.92 | 0.05 |
| 385 | Dimethylphosphite | 22.40 | 22.45 | 22.47 | -0.05 |
| 386 | Ethyl ethoxyacetate | 33.31 | 33.36 | 33.33 | -0.05 |
| 387 | Cetane | 76.09 | 76.04 | 76.06 | 0.05 |
| 388 | Isobutyl acrylate | 36.08 | 36.03 | 36.01 | 0.05 |
| 389 | Isopentyl butanoate | 45.42 | 45.47 | 45.47 | -0.05 |
| 390 | Isopentyliodide | 38.13 | 38.18 | 38.12 | -0.05 |
| 391 | 1,3,5-Trimethylbenzene | 40.99 | 41.04 | 41.01 | -0.05 |
| 392 | Methylthiirane | 22.06 | 22.01 | 22.02 | 0.05 |
| 393 | N,N'-Diethyl-1,2-ethanediamine | 36.55 | 36.60 | 36.56 | -0.05 |
| 394 | t-Pentyl alcohol | 26.70 | 26.65 | 26.66 | 0.05 |
| 395 | Tetrahydro-3-methyl-2H-thiopyran | 35.61 | 35.56 | 35.58 | 0.05 |
| 396 | Tridecanenitrile | 62.08 | 62.03 | 62.03 | 0.05 |
| 397 | Triglyme | 45.97 | 45.92 | 45.98 | 0.05 |
| 398 | Triisopentylamine | 75.33 | 75.28 | 75.30 | 0.05 |
| 399 | Cyclopentyl acetate | 34.07 | 34.12 | 34.16 | -0.05 |
| 400 | Oxirane | 11.01 | 10.96 | 10.95 | 0.05 |
| 401 | 1,1-Dipropoxyethane | 42.37 | 42.32 | 42.38 | 0.05 |
| 402 | (Trichloromethyl)oxirane | 30.33 | 30.28 | 30.24 | 0.05 |
| 403 | Ethylenedichloride | 21.03 | 20.98 | 21.00 | 0.05 |
| 404 | 1-Nitropentane | 30.96 | 30.91 | 30.91 | 0.05 |
| 405 | 2,3-Butyleneglycol | 23.25 | 23.30 | 23.28 | -0.05 |
| 406 | 2-Methyl-3-butyn-2-ol | 24.74 | 24.69 | 24.72 | 0.05 |
| 407 | 2-Methyl-3-pentanol | 31.21 | 31.16 | 31.18 | 0.05 |
| 408 | 2-Methylbutyronitrile | 25.09 | 25.04 | 25.06 | 0.05 |
| 409 | 2-Methylcyclopentanone | 28.10 | 28.15 | 28.17 | -0.05 |
| 410 | 2-Methylpentanoic acid | 31.38 | 31.43 | 31.45 | -0.05 |
| 411 | 4-Methylvaleronitrile | 29.71 | 29.66 | 29.68 | 0.05 |
| 412 | 6-Oxabicyclo[3.1.0]hexane | 22.71 | 22.66 | 22.66 | 0.05 |
| 413 | Bromocyclopentane | 30.98 | 30.93 | 30.96 | 0.05 |
| 414 | Ethyl isocrotonate | 31.73 | 31.68 | 31.66 | 0.05 |
| 415 | Methyl methacrylate | 26.69 | 26.74 | 26.76 | -0.05 |
| 416 | Amyl formate | 31.75 | 31.80 | 31.78 | -0.05 |
| 417 | Nitroethane | 17.10 | 17.05 | 17.06 | 0.05 |
| 418 | Pentane | 25.27 | 25.22 | 25.22 | 0.05 |
| 419 | Propyl chlorocarbonate | 27.46 | 27.41 | 27.43 | 0.05 |
| 420 | Oxetan | 15.63 | 15.58 | 15.59 | 0.05 |
| 421 | 1,10-Undecadiyne | 48.97 | 49.02 | 48.98 | -0.05 |
| 422 | 1,3-Bis(chloromethyl)tetramethyldisiloxane | 58.30 | 58.25 |  | 0.05 |
| 423 | 1-Decene | 48.07 | 48.12 | 48.10 | -0.05 |
| 424 | 1-Ethyl-4-nitrobenzene | 42.74 | 42.69 | 42.69 | 0.05 |
| 425 | 1-Octen-3-ol | 40.18 | 40.23 | 40.23 | -0.05 |
| 426 | 3,4,5-Trimethylheptane | 48.18 | 48.23 | 48.23 | -0.05 |
| 427 | 3,4-Dibromoheptane | 50.07 | 50.12 | 50.16 | -0.05 |
| 428 | 3-Methylcyclohexanone | 32.72 | 32.77 | 32.81 | -0.05 |
| 429 | 6-Methyl-5-hepten-2-ol | 40.36 | 40.41 | 40.46 | -0.05 |
| 430 | Acetophenone | 36.51 | 36.56 | 36.55 | -0.05 |
| 431 | Benzyl formate | 38.01 | 38.06 | 38.04 | -0.05 |
| 432 | Ethyl nonanoate | 54.69 | 54.74 | 54.74 | -0.05 |
| 433 | Tetramethyl silicate | 33.51 | 33.56 | 33.59 | -0.05 |
| 434 | n-Hexylformate | 36.37 | 36.42 | 36.42 | -0.05 |
| 435 | trans-4-Undecene | 52.93 | 52.98 | 52.98 | -0.05 |
| 436 | trans-Azobenzene | 63.27 | 63.22 | 63.54 | 0.05 |
| 437 | Coprostane | 118.27 | 118.21 | 118.19 | 0.06 |
| 438 | 1,1,2-Tribromobutane | 43.83 | 43.77 | 43.86 | 0.06 |
| 439 | 1,2,4-Triethylbenzene | 54.84 | 54.90 | 54.90 | -0.06 |
| 440 | Benzo[d]isoxazole | 32.70 | 32.76 | 32.80 | -0.06 |
| 441 | 1,3-Bis(dimethylamino)-2-propanol | 44.01 | 43.95 | 44.05 | 0.06 |
| 442 | 1,3-Divinyl-1,1,3,3-tetramethyldisiloxane | 57.23 | 57.29 | 57.33 | -0.06 |
| 443 | 1-Bromo-2,2-dimethylpropane | 32.99 | 33.05 | 33.07 | -0.06 |
| 444 | 1-Octyne | 37.06 | 37.12 | 37.11 | -0.06 |
| 445 | 2,3-Dimethylpentane | 34.34 | 34.40 | 34.40 | -0.06 |
| 446 | 2-Ethylhexyl acrylate | 54.45 | 54.51 | 54.50 | -0.06 |
| 447 | 3,4-Dimethylhexane | 39.08 | 39.02 | 39.04 | 0.06 |
| 448 | 3-Methyl-3-heptanol | 40.45 | 40.51 | 40.59 | -0.06 |
| 449 | m-Toluidine | 35.45 | 35.51 | 35.51 | -0.06 |
| 450 | 4-Methyl-1-hexene | 34.29 | 34.23 | 34.25 | 0.06 |
| 451 | 6,6-Dimethylbicyclo(3.1.1)heptan-2-one | 39.94 | 39.88 | 39.87 | 0.06 |
| 452 | Acenaphthene | 51.84 | 51.90 | 51.92 | -0.06 |
| 453 | Cyclohexyl isothiocyanate | 42.69 | 42.63 | 42.69 | 0.06 |
| 454 | Diphenyl phosphonate | 61.69 | 61.75 | 61.92 | -0.06 |
| 455 | N,N-Bis(2-hydroxyethyl)butylamine | 45.83 | 45.77 | 45.74 | 0.06 |
| 456 | n-Pentyl-2-mercaptan | 33.06 | 33.12 | 33.22 | -0.06 |
| 457 | (Methylsulfonyl)ethene | 24.15 | 24.21 | 24.23 | -0.06 |
| 458 | 1,1,2,2-Tetrachloro-1,2-difluoroethane | 30.90 | 30.96 | 30.96 | -0.06 |
| 459 | 1,3-Dichloro-trans-2-butene | 30.17 | 30.23 | 30.23 | -0.06 |
| 460 | 1,3-Dimethyl-1H-pyrazole | 28.23 | 28.29 | 28.31 | -0.06 |
| 461 | 1-Fluorohexane | 29.74 | 29.80 | 29.79 | -0.06 |
| 462 | 2-Butenoyl chloride | 26.26 | 26.32 | 26.32 | -0.06 |
| 463 | 2-Chloroethyl chloroformate | 27.66 | 27.60 | 27.59 | 0.06 |
| 464 | 2-Methyl-1,2-propanediol | 23.46 | 23.40 | 23.35 | 0.06 |
| 465 | 2-Methyl-2-pentene | 29.76 | 29.82 | 29.87 | -0.06 |
| 466 | 3,3-Dimethyl-1-butene | 29.60 | 29.66 | 29.67 | -0.06 |
| 467 | 3-Chloro-1-butene | 25.25 | 25.19 | 25.20 | 0.06 |
| 468 | 3-Methoxypropanenitrile | 22.21 | 22.15 | 22.15 | 0.06 |
| 469 | 3-Methylpyridazine | 27.14 | 27.08 |  | 0.06 |
| 470 | cis-2-Butene-1,4-diol | 23.32 | 23.38 | 23.44 | -0.06 |
| 471 | Isocrotonic acid | 22.32 | 22.26 | 22.23 | 0.06 |
| 472 | Methoxyacetic acid | 19.24 | 19.30 | 19.31 | -0.06 |
| 473 | Methylacetamide | 19.73 | 19.67 | 19.64 | 0.06 |
| 474 | 1-Chlorobutane | 25.47 | 25.41 | 25.42 | 0.06 |
| 475 | Pyrrole | 20.64 | 20.70 | 20.77 | -0.06 |
| 476 | Pentanenitrile | 25.01 | 25.07 | 25.06 | -0.06 |
| 477 | 2-Oxetanone | 15.60 | 15.66 | 15.66 | -0.06 |
| 478 | 1,1,2-Trimethylcyclohexane | 41.63 | 41.57 | 41.61 | 0.06 |
| 479 | 1,2,3,4-Tetrahydro-1,1,6-trimethylnaphthalene | 57.48 | 57.42 | 57.44 | 0.06 |
| 480 | 1,2,3,4-Tetramethylcyclohexane | 46.14 | 46.08 | 46.08 | 0.06 |
| 481 | 1,2-Dichloropentane | 34.76 | 34.82 | 34.82 | -0.06 |
| 482 | 1,3-Dichloropropane | 25.54 | 25.60 | 25.62 | -0.06 |
| 483 | 1,3-Dinitropropane | 27.42 | 27.36 | 27.34 | 0.06 |
| 484 | 1,4-Diisopropylbenzene | 54.74 | 54.68 | 54.70 | 0.06 |
| 485 | 1,9-Decadiene | 47.86 | 47.92 | 47.90 | -0.06 |
| 486 | 1-Acetyl-3-methylpiperidine | 40.91 | 40.85 | 40.74 | 0.06 |
| 487 | 1-Butylamine | 24.08 | 24.02 | 24.04 | 0.06 |
| 488 | Arachic alcohol | 95.83 | 95.89 | 95.89 | -0.06 |
| 489 | 1-Hexadecene | 75.90 | 75.84 | 75.85 | 0.06 |
| 490 | 1-Iodopentane | 38.15 | 38.21 | 38.24 | -0.06 |
| 491 | 1-Methyl-3-propylbenzene | 45.56 | 45.50 | 45.52 | 0.06 |
| 492 | 2,2-Dimethoxy-N-methylethanamine | 31.92 | 31.86 | 31.94 | 0.06 |
| 493 | 2,3-Dibromoheptane | 50.06 | 50.12 | 50.18 | -0.06 |
| 494 | 2,4-Dimethylthiazole | 32.00 | 32.06 |  | -0.06 |
| 495 | 2,5-Lutidine | 33.93 | 33.99 | 34.02 | -0.06 |
| 496 | 2,6-Dimethylmorpholine | 32.92 | 32.86 | 32.78 | 0.06 |
| 497 | s-Butylamine | 24.10 | 24.04 | 24.06 | 0.06 |
| 498 | 2-Bromopropene | 23.13 | 23.19 | 23.19 | -0.06 |
| 499 | 2-Chlorobenzaldehyde | 36.74 | 36.80 | 36.79 | -0.06 |
| 500 | 2-Fluoro-1-phenylethanone | 36.46 | 36.52 | 36.56 | -0.06 |
| 501 | 2-Methyl-1-undecanol | 58.96 | 58.90 | 58.98 | 0.06 |
| 502 | 3,4-Dimethoxybenzenemethanol | 45.83 | 45.89 | 45.92 | -0.06 |
| 503 | 3,4-Dimethylacetophenone | 46.18 | 46.12 | 46.14 | 0.06 |
| 504 | 3,7-Dimethyl-1-octene | 48.12 | 48.06 | 48.05 | 0.06 |
| 505 | 3-Heptanol | 35.75 | 35.81 | 35.81 | -0.06 |
| 506 | 3-Methoxyphenol | 35.02 | 34.96 | 34.97 | 0.06 |
| 507 | Isopentylamine | 28.67 | 28.61 | 28.62 | 0.06 |
| 508 | 3-Methyl-3-hexanol | 35.95 | 35.89 | 35.94 | 0.06 |
| 509 | 3-Phenoxybenzaldehyde | 58.75 | 58.69 | 58.71 | 0.06 |
| 510 | 4,4-Dimethylheptane | 43.78 | 43.72 | 43.73 | 0.06 |
| 511 | 4-Allyl-2-methoxyphenyl acetate | 58.07 | 58.13 | 58.13 | -0.06 |
| 512 | alpha-Pinene | 43.96 | 44.02 | 44.02 | -0.06 |
| 513 | DEHP | 113.78 | 113.72 | 113.74 | 0.06 |
| 514 | Bis(3-chloropropyl) ether | 41.24 | 41.18 | 41.17 | 0.06 |
| 515 | Butylisothiocyanate | 35.55 | 35.49 | 35.55 | 0.06 |
| 516 | cis-1,2-Dichlorocyclohexane | 37.24 | 37.30 | 37.30 | -0.06 |
| 517 | Cyclopentylacetic acid | 33.87 | 33.93 | 33.93 | -0.06 |
| 518 | 2,3-Dimercapto-1-propanol | 32.93 | 32.87 | 33.00 | 0.06 |
| 519 | Eicosamethylnonasiloxane | 179.30 | 179.36 | 179.39 | -0.06 |
| 520 | Ethoxyacetic acid | 23.88 | 23.94 | 23.95 | -0.06 |
| 521 | Ethyl 2,3-dibromopropanoate | 42.60 | 42.66 | 42.68 | -0.06 |
| 522 | Ethyl 2-bromo-3-methylbutanoate | 44.00 | 44.06 | 44.04 | -0.06 |
| 523 | Ethyl benzoate | 42.46 | 42.52 | 42.48 | -0.06 |
| 524 | Dihydro-2(3H)-thiophenone | 26.45 | 26.51 | 26.51 | -0.06 |
| 525 | Heptylbenzene | 59.14 | 59.20 | 59.21 | -0.06 |
| 526 | Isohexanoic acid | 31.49 | 31.43 | 31.40 | 0.06 |
| 527 | 2-Propanethiol | 23.94 | 23.88 | 23.87 | 0.06 |
| 528 | Isopropylphenylether | 42.40 | 42.46 | 42.45 | -0.06 |
| 529 | Menthol | 47.40 | 47.46 | 47.45 | -0.06 |
| 530 | Methoxyacetonitrile | 17.47 | 17.53 | 17.58 | -0.06 |
| 531 | Monobutyl succinate | 42.44 | 42.50 | 42.49 | -0.06 |
| 532 | Nikethamide | 51.53 | 51.47 | 51.52 | 0.06 |
| 533 | Styrene oxide | 35.62 | 35.68 | 35.69 | -0.06 |
| 534 | trans-3,4-Dimethyl-2-pentene | 34.35 | 34.41 | 34.45 | -0.06 |
| 535 | Tridecylbenzene | 86.86 | 86.92 | 86.91 | -0.06 |
| 536 | 1-Cyclohexen-1-ylbenzene | 52.37 | 52.44 | 52.52 | -0.07 |
| 537 | 1-Pentadecyne | 69.53 | 69.46 | 69.48 | 0.07 |
| 538 | 3-Methylheptane | 39.12 | 39.05 | 39.05 | 0.07 |
| 539 | 4-Methylheptane | 39.12 | 39.05 | 39.05 | 0.07 |
| 540 | Lauryl bromide | 65.44 | 65.37 | 65.47 | 0.07 |
| 541 | trans-4-Octene | 39.05 | 39.12 | 39.14 | -0.07 |
| 542 | 2-Nitropropane | 21.72 | 21.65 | 21.57 | 0.07 |
| 543 | Ethyl 2-chloropropanoate | 31.88 | 31.81 | 31.80 | 0.07 |
| 544 | 1,1-Dichloropropane | 25.72 | 25.65 | 25.65 | 0.07 |
| 545 | Tetrahydrofurfurylamine | 28.15 | 28.22 | 28.23 | -0.07 |
| 546 | Diisobutylsulfite | 50.56 | 50.49 | 50.51 | 0.07 |
| 547 | 1,1,1,2,3,3,3-Heptachloropropane | 50.14 | 50.21 | 50.18 | -0.07 |
| 548 | 1,1,1-Trichloroethane | 26.15 | 26.08 | 26.07 | 0.07 |
| 549 | 1,1,2,2-Tetrachloroethane | 30.63 | 30.70 | 30.78 | -0.07 |
| 550 | 1,1-Dimethylcyclohexane | 37.05 | 36.98 | 37.04 | 0.07 |
| 551 | 1,10-Dichlorodecane | 58.01 | 57.94 | 57.96 | 0.07 |
| 552 | 1,2,4-Tribromobutane | 43.98 | 44.05 | 44.08 | -0.07 |
| 553 | 1,2-Cycloheptanedione | 33.19 | 33.26 | 33.26 | -0.07 |
| 554 | 1,2-Dibromotetrafluoroethane | 26.75 | 26.82 |  | -0.07 |
| 555 | 1,3-Dibromo-2,2-dimethylpropane | 40.93 | 40.86 | 40.85 | 0.07 |
| 556 | 1,3-Dichlorobutane | 30.27 | 30.20 | 30.20 | 0.07 |
| 557 | 1,7-Heptanediol | 37.27 | 37.20 | 37.20 | 0.07 |
| 558 | 1-Bromo-2-methoxyethane | 25.42 | 25.49 | 25.52 | -0.07 |
| 559 | 1-Heptyne | 32.43 | 32.50 | 32.55 | -0.07 |
| 560 | 1-Piperazineethanol | 36.49 | 36.42 | 36.39 | 0.07 |
| 561 | 1-Piperidinepropanenitrile | 40.83 | 40.76 | 40.75 | 0.07 |
| 562 | 2,3-Dichloro-1-propanol | 27.02 | 26.95 | 26.91 | 0.07 |
| 563 | 2,4,4-Trimethyl-1-pentene | 38.77 | 38.84 | 38.86 | -0.07 |
| 564 | 2,4,4-Trimethyl-2-pentene | 39.01 | 39.08 | 39.12 | -0.07 |
| 565 | 2,4,4-Trimethylcyclopentanone | 37.34 | 37.41 | 37.44 | -0.07 |
| 566 | 2-Bromobutane | 28.50 | 28.43 | 28.43 | 0.07 |
| 567 | 2-Bromodecanoic acid | 57.70 | 57.77 | 57.77 | -0.07 |
| 568 | 2-Ethoxyethylacetate | 33.29 | 33.36 | 33.36 | -0.07 |
| 569 | 2-Methyl-1-butanethiol | 32.88 | 32.81 | 32.81 | 0.07 |
| 570 | 2-Methyl-1-hexene | 34.27 | 34.20 | 34.19 | 0.07 |
| 571 | 2-Methyltetrahydrofuran | 24.73 | 24.80 | 24.82 | -0.07 |
| 572 | 2-Pentanone oxime | 29.60 | 29.67 | 29.49 | -0.07 |
| 573 | 2-Tolualdehyde | 36.85 | 36.92 | 36.91 | -0.07 |
| 574 | 3,4-Dimethyl-1-pentene | 34.27 | 34.20 | 34.20 | 0.07 |
| 575 | 3-Chloro-2-(chloromethyl)-1-propene | 29.89 | 29.96 | 29.95 | -0.07 |
| 576 | 3-Methylbiphenyl | 56.31 | 56.38 | 56.41 | -0.07 |
| 577 | alpha-Ionol | 61.35 | 61.42 | 61.48 | -0.07 |
| 578 | 4-Heptyl bromide | 42.36 | 42.29 | 42.29 | 0.07 |
| 579 | 4-Bromotoluene | 38.90 | 38.97 | 38.94 | -0.07 |
| 580 | 4-Methyl-1,3-dioxane | 26.26 | 26.33 | 26.31 | -0.07 |
| 581 | 4-Methyl-1-pentene | 29.54 | 29.61 | 29.64 | -0.07 |
| 582 | 4-Methyl-4-heptanol | 40.44 | 40.51 | 40.52 | -0.07 |
| 583 | 4-Octanone | 39.61 | 39.54 | 39.54 | 0.07 |
| 584 | 5-Ethyl-2-picoline | 38.54 | 38.61 | 38.67 | -0.07 |
| 585 | 5-Methyl-5-hexen-2-one | 34.59 | 34.66 | 34.66 | -0.07 |
| 586 | Chloromethylacetate | 22.47 | 22.54 | 22.50 | -0.07 |
| 587 | Acetic acid | 13.05 | 12.98 | 12.98 | 0.07 |
| 588 | Acetoxyacetic acid ethylester | 33.37 | 33.44 | 33.44 | -0.07 |
| 589 | Allyldimethylamine | 29.16 | 29.09 | 29.17 | 0.07 |
| 590 | Aniline | 30.80 | 30.73 | 30.76 | 0.07 |
| 591 | Bis(chloromethyl)ether | 22.67 | 22.60 | 22.58 | 0.07 |
| 592 | cis-3,3,5-Trimethylcyclohexanol | 42.85 | 42.92 | 42.92 | -0.07 |
| 593 | cis-5-Undecene | 52.91 | 52.98 | 52.96 | -0.07 |
| 594 | N,N-Dimethylethanolamine | 25.97 | 26.04 | 26.10 | -0.07 |
| 595 | Diallylsulfoxide | 38.05 | 37.98 |  | 0.07 |
| 596 | Diethyl tartrate | 45.45 | 45.38 | 45.32 | 0.07 |
| 597 | Diethylacetic acid | 31.36 | 31.43 | 31.45 | -0.07 |
| 598 | Dodecane | 57.63 | 57.56 | 57.56 | 0.07 |
| 599 | Ethyl caprate | 59.29 | 59.36 | 59.35 | -0.07 |
| 600 | Ethylene glycol diformate | 24.45 | 24.52 | 24.50 | -0.07 |
| 601 | Ethylene | 10.99 | 10.92 | 10.96 | 0.07 |
| 602 | 1,3-Diacetin | 39.34 | 39.41 | 39.45 | -0.07 |
| 603 | Isoxazole | 16.54 | 16.61 | 16.66 | -0.07 |
| 604 | Meparfynol | 29.24 | 29.31 | 29.26 | -0.07 |
| 605 | Methyl 2-hydroxy-5-methylbenzoate | 44.33 | 44.40 | 44.36 | -0.07 |
| 606 | Dimethoxymethane | 19.12 | 19.19 | 19.19 | -0.07 |
| 607 | Methylenecyclohexane | 32.15 | 32.08 | 32.09 | 0.07 |
| 608 | N-Methyl-o-toluidine | 40.65 | 40.58 | 40.72 | 0.07 |
| 609 | N-Methylformamide | 15.15 | 15.08 | 15.13 | 0.07 |
| 610 | n-Propyl benzoate | 47.21 | 47.14 | 47.10 | 0.07 |
| 611 | Octane | 39.15 | 39.08 | 39.08 | 0.07 |
| 612 | Propanenitrile | 15.76 | 15.83 | 15.83 | -0.07 |
| 613 | Spiro[5,5]undecane | 48.65 | 48.72 | 48.73 | -0.07 |
| 614 | Tetrahydro-2H-pyran-2-methanol | 30.86 | 30.79 | 30.79 | 0.07 |
| 615 | Tetramethylurea | 32.20 | 32.13 | 32.17 | 0.07 |
| 616 | Trichloroacetic acid | 27.77 | 27.70 | 27.68 | 0.07 |
| 617 | Trichloroethylene | 25.37 | 25.44 | 25.42 | -0.07 |
| 618 | Trimethylvinylsilane | 34.55 | 34.48 | 34.39 | 0.07 |
| 619 | 1-Hexylnaphthalene | 72.27 | 72.34 | 72.37 | -0.07 |
| 620 | Bis(2-ethylhexyl) phosphite | 87.18 | 87.11 | 87.13 | 0.07 |
| 621 | Decamethyltetrasiloxane | 86.18 | 86.11 | 86.12 | 0.07 |
| 622 | Nonadecylbenzene | 114.71 | 114.64 | 114.63 | 0.07 |
| 623 | Tri-n-pentylamine | 75.30 | 75.37 | 75.36 | -0.07 |
| 624 | Methyl 2-methylacetoacetate | 31.97 | 32.05 | 32.06 | -0.08 |
| 625 | (4-Methylphenyl)trimethylsilane | 54.82 | 54.90 | 54.97 | -0.08 |
| 626 | 1,2,3,4,5,6,7,8-Octahydroanthracene | 59.98 | 60.06 | 60.12 | -0.08 |
| 627 | 1,2-Dibromoheptane | 50.18 | 50.10 | 50.10 | 0.08 |
| 628 | 1,3-Dimethylcyclohexene | 36.99 | 36.91 | 36.90 | 0.08 |
| 629 | 1-Bromo-2-chlorobenzene | 38.93 | 38.85 | 38.83 | 0.08 |
| 630 | 3-Bromochlorobenzene | 38.93 | 38.85 | 38.83 | 0.08 |
| 631 | 1-Chloro-2,2-dimethylpropane | 30.13 | 30.05 | 30.06 | 0.08 |
| 632 | 1-Chloro-4-isopropylbenzene | 45.43 | 45.35 | 45.39 | 0.08 |
| 633 | 1-Chloropentane | 30.11 | 30.03 | 30.02 | 0.08 |
| 634 | Butyronitrile | 20.37 | 20.45 | 20.44 | -0.08 |
| 635 | 1-Fluoropentane | 25.10 | 25.18 | 25.25 | -0.08 |
| 636 | 1-Heptadecene | 80.54 | 80.46 | 80.45 | 0.08 |
| 637 | 1-Naphthalenethiol | 52.18 | 52.26 | 52.38 | -0.08 |
| 638 | Isooctane | 38.99 | 39.07 | 39.06 | -0.08 |
| 639 | 2,3-Dimethyl-3-pentanol | 35.94 | 35.86 | 35.85 | 0.08 |
| 640 | Glycidyl acrylate | 30.94 | 31.02 | 31.04 | -0.08 |
| 641 | 2,4-Lutidine | 33.91 | 33.99 | 34.02 | -0.08 |
| 642 | 2,5,5-Trimethylheptane | 48.39 | 48.31 | 48.31 | 0.08 |
| 643 | 1-(2-Pyridinyl)ethanone | 34.21 | 34.29 | 34.28 | -0.08 |
| 644 | 2-Methyl-1-octene | 43.36 | 43.44 | 43.41 | -0.08 |
| 645 | 2-Methyl-2-octanol | 45.21 | 45.13 | 45.11 | 0.08 |
| 646 | 3,3-Diethoxy-1-propyne | 35.82 | 35.74 | 35.78 | 0.08 |
| 647 | Ethyl 3,5-dinitrobenzoate | 55.78 | 55.70 | 55.67 | 0.08 |
| 648 | 3-Decen-2-one | 48.74 | 48.82 | 48.79 | -0.08 |
| 649 | 3-Methyl-1H-indene | 43.44 | 43.36 | 43.33 | 0.08 |
| 650 | 3-Methyloctane | 43.75 | 43.67 | 43.69 | 0.08 |
| 651 | 4-Ethylaniline | 40.21 | 40.13 | 40.13 | 0.08 |
| 652 | 4-Methylene-1-isopropylcyclohexene | 45.81 | 45.89 | 45.94 | -0.08 |
| 653 | 2-Bromopropanoic acid | 25.35 | 25.43 | 25.46 | -0.08 |
| 654 | Chlordimeform | 57.07 | 57.15 | 57.33 | -0.08 |
| 655 | Chloro(chloromethyl)dimethylsilane | 34.43 | 34.35 |  | 0.08 |
| 656 | cis-1,3-Dichloropropene | 25.21 | 25.29 | 25.29 | -0.08 |
| 657 | cis-3-Methyl-2-hexene | 34.36 | 34.44 | 34.49 | -0.08 |
| 658 | Decamethylpentasiloxane | 93.33 | 93.25 | 93.25 | 0.08 |
| 659 | Decanenitrile | 48.25 | 48.17 | 48.17 | 0.08 |
| 660 | Di-n-propylsulfate | 41.12 | 41.04 | 41.06 | 0.08 |
| 661 | Diethyl 2-aminomalonate | 41.58 | 41.50 | 41.46 | 0.08 |
| 662 | Dioctyl maleate | 98.08 | 98.16 | 98.18 | -0.08 |
| 663 | Dodecylbenzene | 82.22 | 82.30 | 82.29 | -0.08 |
| 664 | Ethyl 3-methyl-2-butenoate | 36.32 | 36.24 | 36.22 | 0.08 |
| 665 | Ethyl fluoroacetate | 22.28 | 22.36 | 22.37 | -0.08 |
| 666 | Ethylbenzene sulfonate | 45.63 | 45.71 | 45.88 | -0.08 |
| 667 | Ethylcyclohexane | 37.01 | 36.93 | 36.99 | 0.08 |
| 668 | Hexane | 29.76 | 29.84 | 29.86 | -0.08 |
| 669 | Hydrosorbic acid | 31.42 | 31.50 | 31.47 | -0.08 |
| 670 | Isopropyl methacrylate | 36.06 | 35.98 | 35.97 | 0.08 |
| 671 | Methyllaurate | 63.88 | 63.96 | 63.97 | -0.08 |
| 672 | Methyllactate | 23.81 | 23.73 | 23.70 | 0.08 |
| 673 | Caprylic aldehyde | 39.66 | 39.74 | 39.76 | -0.08 |
| 674 | Octamethylcyclotetrasiloxane | 74.68 | 74.60 | 74.60 | 0.08 |
| 675 | Octyl propanoate | 54.66 | 54.74 | 54.74 | -0.08 |
| 676 | Phenylurethane | 46.67 | 46.75 | 46.98 | -0.08 |
| 677 | Propanamide | 19.14 | 19.22 | 19.21 | -0.08 |
| 678 | t-Butylcyclohexane | 46.11 | 46.19 | 46.20 | -0.08 |
| 679 | Thymol | 47.29 | 47.21 | 47.21 | 0.08 |
| 680 | trans-3-Heptene | 34.42 | 34.50 | 34.48 | -0.08 |
| 681 | 10-Undecenoic acid | 54.44 | 54.36 | 54.42 | 0.08 |
| 682 | 1-(Bromomethyl)-2-methylbenzene | 44.15 | 44.07 | 44.05 | 0.08 |
| 683 | Tripropoxymethane | 53.28 | 53.36 |  | -0.08 |
| 684 | 1,1,2-Trichloroethane | 25.92 | 25.84 | 25.86 | 0.08 |
| 685 | 1,2-Dibromo-1,1-difluoroethane | 26.82 | 26.90 |  | -0.08 |
| 686 | Isopentyl chloride | 30.08 | 30.00 | 30.00 | 0.08 |
| 687 | 2-Methyloxetane | 20.26 | 20.18 | 20.16 | 0.08 |
| 688 | 3-Bromopropanenitrile | 23.56 | 23.64 | 23.64 | -0.08 |
| 689 | 3-Pentanol | 26.65 | 26.57 | 26.57 | 0.08 |
| 690 | 4-Bromobutanenitrile | 28.18 | 28.26 | 28.28 | -0.08 |
| 691 | Bis(2-cyanoethyl) ether | 31.18 | 31.26 | 31.20 | -0.08 |
| 692 | Methyl dichloroacetate | 27.51 | 27.43 | 27.40 | 0.08 |
| 693 | Trimethylacetic acid | 26.94 | 26.86 | 26.87 | 0.08 |
| 694 | Propyl isocyanate | 22.57 | 22.65 | 22.86 | -0.08 |
| 695 | 1,2-Dibromo-2-methylpropane | 36.34 | 36.26 | 36.28 | 0.08 |
| 696 | 1,2-Dichlorobenzene | 35.94 | 36.02 | 36.04 | -0.08 |
| 697 | 1-Ethynylcyclohexanamine | 38.45 | 38.37 | 38.44 | 0.08 |
| 698 | 2-Hydroxybicyclopentyl | 45.45 | 45.37 | 45.36 | 0.08 |
| 699 | 3,7-Dimethyl-3-octanol | 49.80 | 49.72 | 49.71 | 0.08 |
| 700 | 4-Ethyl-1,2-dimethylbenzene | 45.58 | 45.66 | 45.63 | -0.08 |
| 701 | Anabasine | 48.91 | 48.83 | 48.80 | 0.08 |
| 702 | cis-3,4-Dimethyl-2-pentene | 34.33 | 34.41 | 34.45 | -0.08 |
| 703 | cis-4,4-Dimethyl-2-pentene | 34.44 | 34.52 | 34.53 | -0.08 |
| 704 | Ethyl isovalerate | 36.15 | 36.23 | 36.21 | -0.08 |
| 705 | 1-Iodobutane | 33.51 | 33.59 | 33.58 | -0.08 |
| 706 | TEPP | 61.73 | 61.65 | 61.71 | 0.08 |
| 707 | Ricinelaidic acid | 88.38 | 88.29 | 88.25 | 0.09 |
| 708 | (1-Bromoethyl)benzene | 43.84 | 43.93 | 43.93 | -0.09 |
| 709 | 1,2-Bis(2-chloroethoxy)ethane | 42.81 | 42.90 | 42.88 | -0.09 |
| 710 | 1-Amino-1-phenylethane | 39.45 | 39.54 | 39.49 | -0.09 |
| 711 | 1-Hepten-4-ol | 35.52 | 35.61 | 35.61 | -0.09 |
| 712 | 1-Naphthalenecarbonitrile | 49.18 | 49.09 | 49.09 | 0.09 |
| 713 | 2,3,6-Collidine | 39.00 | 38.91 | 39.05 | 0.09 |
| 714 | 2,3-Dichloropentane | 34.89 | 34.80 | 34.82 | 0.09 |
| 715 | 2,5-Dichlorothiophene | 34.44 | 34.35 | 34.29 | 0.09 |
| 716 | 2,6-Dimethyloctane | 48.17 | 48.26 | 48.26 | -0.09 |
| 717 | 2-Ethyl-1-hexanoic acid | 40.76 | 40.67 | 40.69 | 0.09 |
| 718 | 2-t-Butyl-4-methylphenol | 51.97 | 51.88 | 51.93 | 0.09 |
| 719 | 3-Chlorobenzeneacetonitrile | 40.52 | 40.61 | 40.59 | -0.09 |
| 720 | Allyl 2-furancarboxylate | 39.76 | 39.67 | 39.72 | 0.09 |
| 721 | Bromocyclohexane | 35.64 | 35.55 | 35.55 | 0.09 |
| 722 | Decanedinitrile | 48.11 | 48.02 | 48.04 | 0.09 |
| 723 | Dimethyl citraconate | 37.91 | 38.00 | 38.01 | -0.09 |
| 724 | Ethylcyclopentane | 32.40 | 32.31 | 32.31 | 0.09 |
| 725 | Methyl 2-hydroxy-3-methylbenzoate | 44.31 | 44.40 | 44.42 | -0.09 |
| 726 | Methyl benzoate | 37.97 | 37.88 | 37.84 | 0.09 |
| 727 | Nerol | 49.56 | 49.65 | 49.65 | -0.09 |
| 728 | Triisopropyl borate | 52.52 | 52.61 | 52.68 | -0.09 |
| 729 | Cyclohexanol | 29.16 | 29.07 | 29.11 | 0.09 |
| 730 | 1,2-Dichloro-1,2-difluoroethane | 21.13 | 21.22 | 21.34 | -0.09 |
| 731 | 2-Fluoro-1,1,2-trichloroethane | 25.87 | 25.96 | 25.93 | -0.09 |
| 732 | 3-Methyl-cis-2-pentene | 29.73 | 29.82 | 29.84 | -0.09 |
| 733 | 3-Methyl-trans-2-pentene | 29.73 | 29.82 | 29.86 | -0.09 |
| 734 | 4-Methyl-trans-2-pentene | 29.76 | 29.85 | 29.85 | -0.09 |
| 735 | Butyl vinyl ether | 30.96 | 30.87 | 30.79 | 0.09 |
| 736 | cis-1,4-Dichloro-2-butene | 30.35 | 30.26 | 30.24 | 0.09 |
| 737 | Cyclopentanol | 24.54 | 24.45 | 24.51 | 0.09 |
| 738 | Ethyl crotonate | 31.77 | 31.68 | 31.66 | 0.09 |
| 739 | Hexafluorobenzene | 26.49 | 26.40 | 26.34 | 0.09 |
| 740 | Methylenecyclopentane | 27.55 | 27.46 | 27.47 | 0.09 |
| 741 | 3-Pyridinemethanol | 30.53 | 30.44 | 30.41 | 0.09 |
| 742 | Tetrahydropyran | 24.73 | 24.82 | 24.81 | -0.09 |
| 743 | 3,4-Dimethoxytoluene | 44.43 | 44.52 | 44.56 | -0.09 |
| 744 | 1,7-Octadiyne | 35.07 | 35.16 | 35.16 | -0.09 |
| 745 | 1-Bromoundecane | 60.84 | 60.75 | 60.76 | 0.09 |
| 746 | 1-Methyl-4-propylbenzene | 45.59 | 45.50 | 45.48 | 0.09 |
| 747 | 10-Chloro-1-decanol | 54.59 | 54.50 | 54.57 | 0.09 |
| 748 | 2-(Benzyloxy)ethanol | 43.72 | 43.81 | 43.81 | -0.09 |
| 749 | 2-Butyl-1,1'-biphenyl | 70.33 | 70.24 | 70.27 | 0.09 |
| 750 | 2-Chloro-N-(2-chloroethyl)-N-ethylethanamine | 43.32 | 43.41 | 43.46 | -0.09 |
| 751 | 2-Ethoxyethyl acrylate | 37.69 | 37.78 | 37.76 | -0.09 |
| 752 | 3,3-Dimethylhexane | 39.01 | 39.10 | 39.11 | -0.09 |
| 753 | 3-Ethylhexane | 38.96 | 39.05 | 39.11 | -0.09 |
| 754 | 4-Methoxy-2-methylaniline | 41.94 | 42.03 | 42.06 | -0.09 |
| 755 | 4-Methylcyclohexanone | 32.68 | 32.77 | 32.81 | -0.09 |
| 756 | 4-Methylnonane | 48.38 | 48.29 | 48.29 | 0.09 |
| 757 | 5-Bromopentanenitrile | 32.79 | 32.88 | 32.87 | -0.09 |
| 758 | 5-Methyl-1-hexanol | 35.71 | 35.80 | 35.83 | -0.09 |
| 759 | Allyltrimethylsilane | 39.14 | 39.23 | 39.27 | -0.09 |
| 760 | beta-Citronellol | 49.73 | 49.64 | 49.70 | 0.09 |
| 761 | Butyldimethylamine | 33.82 | 33.91 | 33.89 | -0.09 |
| 762 | cis-2,2-Dimethyl-3-hexene | 39.23 | 39.14 | 39.11 | 0.09 |
| 763 | Decane | 48.41 | 48.32 | 48.32 | 0.09 |
| 764 | Dichlordiethylsilane | 38.71 | 38.80 | 38.87 | -0.09 |
| 765 | Heptyl pentanoate | 59.45 | 59.36 | 59.36 | 0.09 |
| 766 | Iodocyclohexane | 41.05 | 40.96 | 40.95 | 0.09 |
| 767 | Ethyl 4-pyridinecarboxylate | 40.20 | 40.11 | 40.07 | 0.09 |
| 768 | N,N-Diethyl-3-methylbutanamide | 47.50 | 47.59 | 47.60 | -0.09 |
| 769 | n-Octylamine | 42.59 | 42.50 | 42.51 | 0.09 |
| 770 | n-Amyl mercaptan | 32.75 | 32.84 | 32.86 | -0.09 |
| 771 | Patchouli alcohol | 66.35 | 66.44 | 66.45 | -0.09 |
| 772 | t-Butyl acrylate | 36.21 | 36.12 | 36.11 | 0.09 |
| 773 | Tetracosane | 113.09 | 113.00 | 113.02 | 0.09 |
| 774 | trans-4-Methyl-2-hexene | 34.56 | 34.47 | 34.47 | 0.09 |
| 775 | Tripropyl phosphite | 56.91 | 56.82 | 56.84 | 0.09 |
| 776 | 1,3-Dichlorobenzene | 36.12 | 36.02 | 35.96 | 0.10 |
| 777 | 1-Pentylnaphthalene | 67.62 | 67.72 | 67.75 | -0.10 |
| 778 | 2-Iodobutane | 33.94 | 33.84 | 33.85 | 0.10 |
| 779 | 3-Chlorobenzaldehyde | 36.90 | 36.80 | 36.82 | 0.10 |
| 780 | 3-Methyl-2-heptanol | 40.30 | 40.40 | 40.42 | -0.10 |
| 781 | Butyl 4-toluenesulfonate | 59.83 | 59.73 | 59.75 | 0.10 |
| 782 | Exaltol | 70.75 | 70.65 | 70.64 | 0.10 |
| 783 | Ethoheptazine | 76.67 | 76.77 | 76.72 | -0.10 |
| 784 | Ethyl 2-methylacetoacetate | 36.59 | 36.69 | 36.69 | -0.10 |
| 785 | Ethylidenecyclohexane | 36.84 | 36.94 | 36.94 | -0.10 |
| 786 | Butyl propanoate | 36.16 | 36.26 | 36.24 | -0.10 |
| 787 | Propyl butanoate | 36.16 | 36.26 | 36.24 | -0.10 |
| 788 | Triethoxypentylsilane | 64.96 | 65.06 | 65.04 | -0.10 |
| 789 | 1-Bromobutane | 28.31 | 28.41 | 28.42 | -0.10 |
| 790 | Epichlorohydrin | 20.47 | 20.37 | 20.36 | 0.10 |
| 791 | 1-Iodopropane | 28.87 | 28.97 | 28.96 | -0.10 |
| 792 | 1-Nitropropane | 21.77 | 21.67 | 21.68 | 0.10 |
| 793 | 2,3-Dibromo-1-propene | 30.90 | 31.00 | 31.05 | -0.10 |
| 794 | Methyl acrylate | 22.08 | 22.18 | 22.18 | -0.10 |
| 795 | Ethyl mercaptoacetate | 29.92 | 30.02 | 30.10 | -0.10 |
| 796 | 1-Fluorobutane | 20.46 | 20.56 | 20.56 | -0.10 |
| 797 | Pentafluorobenzene | 26.35 | 26.45 | 26.50 | -0.10 |
| 798 | Propionic anhydride | 31.47 | 31.57 | 31.62 | -0.10 |
| 799 | trans-2-Methyl-2-pentenoic acid | 31.54 | 31.44 | 31.43 | 0.10 |
| 800 | 1,2,3-Propanetriol tripropanoate | 62.84 | 62.94 | 62.88 | -0.10 |
| 801 | 1,4-Bis(3-aminopropoxy)butane | 58.50 | 58.60 | 58.60 | -0.10 |
| 802 | 4-Ethoxytoluene | 42.54 | 42.64 | 42.67 | -0.10 |
| 803 | Caprylene | 38.78 | 38.88 | 38.87 | -0.10 |
| 804 | 2,2-Dimethyloxirane | 20.16 | 20.26 | 20.28 | -0.10 |
| 805 | 2,3-Dimethyl-2-hexene | 39.10 | 39.00 | 39.00 | 0.10 |
| 806 | 2,4-Dimethyl-3-pentanol | 35.85 | 35.75 | 35.75 | 0.10 |
| 807 | 2,4-Dimethylpyrrole | 30.54 | 30.64 | 30.88 | -0.10 |
| 808 | 2-Butylnaphthalene | 63.20 | 63.10 | 63.13 | 0.10 |
| 809 | 2-Methyl-1,5-hexadiene | 33.90 | 34.00 | 33.99 | -0.10 |
| 810 | 2-Methyl-1-pentene | 29.48 | 29.58 | 29.59 | -0.10 |
| 811 | Isobutylbenzene | 45.21 | 45.31 | 45.30 | -0.10 |
| 812 | 2-Vinylpyridine | 33.53 | 33.63 | 33.70 | -0.10 |
| 813 | 3-Methyl-2-butanol | 26.64 | 26.54 | 26.53 | 0.10 |
| 814 | 3-Methyl-4-methylenehexane | 38.69 | 38.79 | 38.82 | -0.10 |
| 815 | 3-Methylnonane | 48.19 | 48.29 | 48.29 | -0.10 |
| 816 | 5,5-Dimethyl-1-hexene | 39.00 | 38.90 | 38.94 | 0.10 |
| 817 | 5-Butyldihydro-2(3H)-furanone | 38.64 | 38.74 | 38.79 | -0.10 |
| 818 | Bornyl acetate | 55.01 | 55.11 | 55.15 | -0.10 |
| 819 | Bromoacetyl bromide | 27.60 | 27.50 |  | 0.10 |
| 820 | Butoxybenzene | 47.00 | 47.10 | 47.10 | -0.10 |
| 821 | cis-2-Methyl-3-hexene | 34.57 | 34.47 | 34.49 | 0.10 |
| 822 | cis-4-Undecene | 52.88 | 52.98 | 53.05 | -0.10 |
| 823 | cis-5-Decene | 48.26 | 48.36 | 48.36 | -0.10 |
| 824 | Cyclohexylhydroperoxide | 31.49 | 31.39 |  | 0.10 |
| 825 | Iodocyclopentane | 36.24 | 36.34 | 36.37 | -0.10 |
| 826 | Di-n-propylmaleate | 51.86 | 51.96 | 51.92 | -0.10 |
| 827 | Ethyl dichloroacetate | 32.17 | 32.07 | 32.04 | 0.10 |
| 828 | Dipropyl carbonate | 37.63 | 37.73 | 37.68 | -0.10 |
| 829 | Colamine | 16.25 | 16.15 | 16.14 | 0.10 |
| 830 | Ethyl 2-butynoate | 30.48 | 30.58 | 30.61 | -0.10 |
| 831 | Ethyl 3-ethoxypropanoate | 37.88 | 37.98 | 37.96 | -0.10 |
| 832 | Ethyl cyclohexylacetate | 48.07 | 47.97 | 47.96 | 0.10 |
| 833 | Ethyl trichloroacetate | 37.22 | 37.12 | 37.14 | 0.10 |
| 834 | Isobutyl 3-methylbutanoate | 45.54 | 45.44 | 45.43 | 0.10 |
| 835 | Isobutyl isobutyrate | 40.92 | 40.82 | 40.83 | 0.10 |
| 836 | Methyl isonicotinate | 35.57 | 35.47 | 35.47 | 0.10 |
| 837 | Methyl-n-pentylether | 31.64 | 31.54 | 31.56 | 0.10 |
| 838 | N-Ethylaniline | 40.46 | 40.36 | 40.34 | 0.10 |
| 839 | N-Methyldiphenylamine | 61.39 | 61.49 | 61.48 | -0.10 |
| 840 | Phenylpropylether | 42.38 | 42.48 | 42.50 | -0.10 |
| 841 | t-Butyl vinyl ether | 31.03 | 30.93 | 30.82 | 0.10 |
| 842 | trans-4,4-Dimethyl-2-pentene | 34.42 | 34.52 | 34.52 | -0.10 |
| 843 | Tributylamine | 61.61 | 61.51 | 61.51 | 0.10 |
| 844 | Undecane | 53.04 | 52.94 | 52.94 | 0.10 |
| 845 | 1-Hexadecyne | 74.18 | 74.08 | 74.10 | 0.10 |
| 846 | Undecylbenzene | 77.58 | 77.68 | 77.67 | -0.10 |
| 847 | Squalene | 140.49 | 140.60 | 140.78 | -0.11 |
| 848 | (cis-2-Bromovinyl)benzene | 43.67 | 43.56 | 43.59 | 0.11 |
| 849 | 1,1,2-Trimethylcyclopentane | 37.06 | 36.95 | 36.95 | 0.11 |
| 850 | 1,1-Dichloroethane | 21.14 | 21.03 | 21.03 | 0.11 |
| 851 | 1,2-Dihydronaphthalene | 43.53 | 43.42 | 43.42 | 0.11 |
| 852 | 1,3,5-Triethylbenzene | 55.01 | 54.90 | 54.90 | 0.11 |
| 853 | Diglycidyl resorcinol ether | 57.31 | 57.42 | 57.36 | -0.11 |
| 854 | 1-Bromo-2-chloroethane | 23.87 | 23.98 | 23.99 | -0.11 |
| 855 | Isopentyl bromide | 33.11 | 33.00 | 33.01 | 0.11 |
| 856 | 1-Bromopropane | 23.68 | 23.79 | 23.81 | -0.11 |
| 857 | 1-Bromotetradecane | 74.72 | 74.61 | 74.61 | 0.11 |
| 858 | 1-Chlorohexane | 34.76 | 34.65 | 34.66 | 0.11 |
| 859 | 1-Methyl-1H-indene | 43.50 | 43.39 | 43.37 | 0.11 |
| 860 | 2,2,3-Tribromobutane | 43.90 | 44.01 | 44.07 | -0.11 |
| 861 | 2,3-Dihydro-1H-inden-1-one | 39.11 | 39.22 | 39.26 | -0.11 |
| 862 | 2,4,6,8-Tetramethylcyclotetrasiloxane | 57.13 | 57.24 | 57.24 | -0.11 |
| 863 | 2,6-Dimethyl-2-heptanol | 44.99 | 45.10 | 45.14 | -0.11 |
| 864 | 2-Chloro-N,N-dimethylaniline | 45.32 | 45.43 | 45.51 | -0.11 |
| 865 | 2-Chloropropene | 20.45 | 20.56 | 20.55 | -0.11 |
| 866 | 3-Methyleneheptane | 38.71 | 38.82 | 38.83 | -0.11 |
| 867 | Methyl 2-furancarboxylate | 30.72 | 30.61 | 30.65 | 0.11 |
| 868 | 2-Methyl-1-butene | 24.85 | 24.96 | 24.97 | -0.11 |
| 869 | 3,3,4-Trimethylhexane | 43.58 | 43.69 | 43.72 | -0.11 |
| 870 | 3,4-Dihydro-2-methyl-1(2H)-naphthalenone | 48.54 | 48.43 | 48.40 | 0.11 |
| 871 | 3-(Chloromethyl)heptane | 43.97 | 43.86 | 43.87 | 0.11 |
| 872 | 3-Ethyl-2-methylhexane | 43.53 | 43.64 | 43.64 | -0.11 |
| 873 | m-Anisidine | 37.36 | 37.25 | 37.19 | 0.11 |
| 874 | 3-Methylanisole | 37.89 | 38.00 | 37.98 | -0.11 |
| 875 | 3-Methylbutyl 3-chloropropanoate | 45.77 | 45.66 | 45.65 | 0.11 |
| 876 | 4-Heptanamine | 37.79 | 37.90 | 37.92 | -0.11 |
| 877 | 4-Methyl-3-penten-2-ol | 31.28 | 31.17 | 31.18 | 0.11 |
| 878 | 4-Methylbenzenemethanamine | 39.57 | 39.68 | 39.66 | -0.11 |
| 879 | 4-Pentenenitrile | 24.98 | 24.87 | 24.87 | 0.11 |
| 880 | 4-t-Butyl-2-methylphenol | 51.99 | 51.88 | 51.92 | 0.11 |
| 881 | 5-Butylnonane | 62.04 | 62.15 | 62.18 | -0.11 |
| 882 | Allyltriethoxysilane | 55.73 | 55.62 | 55.60 | 0.11 |
| 883 | alpha-Methyl-4-isopropylbenzenepropanal | 59.85 | 59.96 | 59.95 | -0.11 |
| 884 | Benzyl nitrite | 37.45 | 37.56 | 37.57 | -0.11 |
| 885 | Bis(2-chloroethyl)carbonate | 38.00 | 38.11 | 38.04 | -0.11 |
| 886 | Chloro(dichloromethyl)dimethylsilane | 39.39 | 39.28 |  | 0.11 |
| 887 | cis-1-Ethyl-3-methylcyclopentane | 36.79 | 36.90 | 36.94 | -0.11 |
| 888 | cis-2-Hexen-1-ol | 31.14 | 31.25 | 31.25 | -0.11 |
| 889 | Cyclohexanemethanamine | 35.84 | 35.73 | 35.74 | 0.11 |
| 890 | Dibutyl malonate | 56.43 | 56.54 | 56.50 | -0.11 |
| 891 | Dibutyl phosphonate | 50.10 | 50.21 | 50.25 | -0.11 |
| 892 | Diethyl 2-bromomalonate | 46.00 | 45.89 | 45.86 | 0.11 |
| 893 | Dimethyl carbonate | 19.10 | 19.21 | 19.27 | -0.11 |
| 894 | Ethoxytrimethylsilane | 35.67 | 35.56 | 35.65 | 0.11 |
| 895 | Ethyl isobutyrate | 31.50 | 31.61 | 31.62 | -0.11 |
| 896 | Ethyl methacrylate | 31.27 | 31.38 | 31.39 | -0.11 |
| 897 | Isopentyl formate | 31.88 | 31.77 | 31.76 | 0.11 |
| 898 | Isopropylcyclopentane | 37.01 | 36.90 | 36.90 | 0.11 |
| 899 | m-Xylene | 36.15 | 36.26 | 36.26 | -0.11 |
| 900 | Methyl 3-methyl-2-butenoate | 31.71 | 31.60 | 31.61 | 0.11 |
| 901 | N,N-Bis(2-hydroxyethyl)ethylamine | 36.42 | 36.53 | 36.50 | -0.11 |
| 902 | n-Butyldichloroacetate | 41.42 | 41.31 | 41.30 | 0.11 |
| 903 | n-Propyl acetate | 26.91 | 27.02 | 27.02 | -0.11 |
| 904 | o-Toluenesulfonyl chloride | 45.82 | 45.93 | 46.02 | -0.11 |
| 905 | Propanal oxime | 20.34 | 20.45 | 20.54 | -0.11 |
| 906 | Propyl valerate | 40.77 | 40.88 | 40.87 | -0.11 |
| 907 | Pyrrolidine | 21.97 | 21.86 | 21.90 | 0.11 |
| 908 | t-Butylhydroperoxide | 24.46 | 24.35 | 24.37 | 0.11 |
| 909 | t-Butylmercaptan | 28.92 | 28.81 | 28.81 | 0.11 |
| 910 | Tetramethoxymethane | 31.15 | 31.26 |  | -0.11 |
| 911 | Tetramethyloxirane | 29.67 | 29.56 | 29.54 | 0.11 |
| 912 | trans-2-Hepten-1-ol | 35.76 | 35.87 | 35.89 | -0.11 |
| 913 | Tropacocaine | 70.14 | 70.03 | 70.01 | 0.11 |
| 914 | Vinyldiethoxymethylsilane | 45.09 | 45.20 | 45.19 | -0.11 |
| 915 | 2-Bromo-3-methyl-1-butene | 32.51 | 32.40 | 32.45 | 0.11 |
| 916 | 1-Chloro-2-methylpropane | 25.49 | 25.38 | 25.38 | 0.11 |
| 917 | 1,2,3-Propanetriol trioctanoate | 132.13 | 132.24 | 132.26 | -0.11 |
| 918 | Trilaurin | 187.57 | 187.68 | 187.71 | -0.11 |
| 919 | Cadalene | 68.13 | 68.01 | 67.99 | 0.12 |
| 920 | (Bromomethyl)chlorodimethylsilane | 37.57 | 37.45 |  | 0.12 |
| 921 | (Chloromethyl)trichlorosilane | 34.10 | 34.22 | 34.18 | -0.12 |
| 922 | 1-Bromo-3-fluorobenzene | 34.26 | 34.14 | 34.15 | 0.12 |
| 923 | 1-Chloro-2-isopropylbenzene | 45.23 | 45.35 | 45.36 | -0.12 |
| 924 | 1-Heptene | 34.14 | 34.26 | 34.29 | -0.12 |
| 925 | 1-Hexyne | 27.76 | 27.88 | 27.92 | -0.12 |
| 926 | 2,4-Dimethyl-2-pentene | 34.53 | 34.41 | 34.41 | 0.12 |
| 927 | 2-(2-Pyridyl)ethylamine | 37.13 | 37.25 | 37.26 | -0.12 |
| 928 | 2-Fluorobenzaldehyde | 32.21 | 32.09 | 32.12 | 0.12 |
| 929 | 3,4-Dimethylheptane | 43.52 | 43.64 | 43.64 | -0.12 |
| 930 | 3-Chloropentane | 30.13 | 30.01 | 30.03 | 0.12 |
| 931 | 3-Chlorophenol | 33.22 | 33.10 | 33.13 | 0.12 |
| 932 | 3-Methyl-1-pentene | 29.49 | 29.61 | 29.60 | -0.12 |
| 933 | beta-Ionol | 61.51 | 61.39 | 61.35 | 0.12 |
| 934 | 4-Methylacetophenone | 41.46 | 41.34 | 41.36 | 0.12 |
| 935 | 4-Piperidineethanol | 37.18 | 37.06 | 37.12 | 0.12 |
| 936 | 2-Ethylbutyl acetate | 40.73 | 40.85 | 40.83 | -0.12 |
| 937 | Allylamine | 19.08 | 19.20 | 19.19 | -0.12 |
| 938 | Butyl enanthate | 54.62 | 54.74 | 54.74 | -0.12 |
| 939 | cis-1,2-Dichloroethylene | 20.26 | 20.38 | 20.40 | -0.12 |
| 940 | cis-2-Hexene | 29.76 | 29.88 | 29.88 | -0.12 |
| 941 | Ethyl 2-cyclopentanone-1-carboxylate | 39.07 | 39.19 | 39.18 | -0.12 |
| 942 | Ethyl propyl sulfide | 33.21 | 33.09 | 33.11 | 0.12 |
| 943 | Acetol | 17.69 | 17.81 | 17.78 | -0.12 |
| 944 | Iodobenzene | 39.14 | 39.26 | 39.31 | -0.12 |
| 945 | Isopentyl alcohol | 26.68 | 26.56 | 26.55 | 0.12 |
| 946 | N,N-Dimethylaniline | 40.89 | 40.77 | 40.88 | 0.12 |
| 947 | n-Butyl-2-chloropropanoate | 41.17 | 41.05 | 41.04 | 0.12 |
| 948 | n-Butyl-3-chloropropanoate | 41.19 | 41.07 | 41.09 | 0.12 |
| 949 | n-Heptylamine | 38.00 | 37.88 | 37.89 | 0.12 |
| 950 | Exalgin | 45.24 | 45.36 | 45.52 | -0.12 |
| 951 | n-Propylmethacrylate | 35.88 | 36.00 | 36.01 | -0.12 |
| 952 | Mercaptoacetic acid | 20.72 | 20.60 | 20.57 | 0.12 |
| 953 | Tripropyl borate | 52.55 | 52.67 | 52.68 | -0.12 |
| 954 | 2-Propylpentanoic acid | 40.79 | 40.67 | 40.64 | 0.12 |
| 955 | Benzenesulfonylchloride | 41.03 | 41.15 | 41.20 | -0.12 |
| 956 | 1-Fluoropropane | 15.82 | 15.94 | 15.94 | -0.12 |
| 957 | Cyanoethylene | 15.75 | 15.63 | 15.62 | 0.12 |
| 958 | 1,4-Pentanediol | 28.06 | 27.94 | 27.91 | 0.12 |
| 959 | 1-Hexene | 29.52 | 29.64 | 29.64 | -0.12 |
| 960 | 1-Pentyne | 23.14 | 23.26 | 23.32 | -0.12 |
| 961 | 2-Chloroethanesulfonyl chloride | 30.41 | 30.29 | 30.28 | 0.12 |
| 962 | 2-Pyridinemethanol | 30.46 | 30.58 | 30.59 | -0.12 |
| 963 | Isopentane | 25.31 | 25.19 | 25.22 | 0.12 |
| 964 | Allyl chloride | 20.47 | 20.59 | 20.58 | -0.12 |
| 965 | 4-Chloro-1-butanol | 26.90 | 26.78 | 26.80 | 0.12 |
| 966 | Allyl vinyl ether | 26.17 | 26.05 | 26.08 | 0.12 |
| 967 | 7-Oxabicyclo[4.1.0]heptane | 27.40 | 27.28 | 27.31 | 0.12 |
| 968 | Diethyl sulphide | 28.59 | 28.47 | 28.47 | 0.12 |
| 969 | Ethyl lactate | 28.49 | 28.37 | 28.35 | 0.12 |
| 970 | Ethyl iodide | 24.23 | 24.35 | 24.36 | -0.12 |
| 971 | N-Methylpropionamide | 24.41 | 24.29 | 24.22 | 0.12 |
| 972 | N-Methyl-2-pyrrolidone | 26.96 | 27.08 | 27.10 | -0.12 |
| 973 | n-Propylchloroacetate | 31.71 | 31.83 | 31.85 | -0.12 |
| 974 | Methylvalerate | 31.50 | 31.62 | 31.62 | -0.12 |
| 975 | 1,2-Dichloro-1,2-difluoroethene | 20.48 | 20.60 |  | -0.12 |
| 976 | 1,2-Diisopropylhydrazine | 37.04 | 37.16 | 37.34 | -0.12 |
| 977 | 3,5-Dimethoxytoluene | 44.40 | 44.52 | 44.48 | -0.12 |
| 978 | 1-Bromotridecane | 70.11 | 69.99 | 69.98 | 0.12 |
| 979 | Norpempidine | 44.98 | 44.86 | 44.62 | 0.12 |
| 980 | 2,3,4-Trimethylpentane | 38.87 | 38.99 | 39.01 | -0.12 |
| 981 | 2-Ethyl-1,4-dimethylbenzene | 45.54 | 45.66 | 45.69 | -0.12 |
| 982 | 2-Tolunitrile | 36.23 | 36.11 | 36.13 | 0.12 |
| 983 | 2-Methylheptyl acetate | 49.97 | 50.09 | 50.10 | -0.12 |
| 984 | 2-Methylnonane | 48.41 | 48.29 | 48.29 | 0.12 |
| 985 | 2-Phenyl-1,3-butadiene | 44.76 | 44.88 | 44.91 | -0.12 |
| 986 | 3-Methoxyacetophenone | 43.20 | 43.08 | 43.10 | 0.12 |
| 987 | 3-Methylquinoline | 46.95 | 46.83 | 46.69 | 0.12 |
| 988 | 4-Chloro-1-(4-fluorophenyl)-1-butanone | 50.44 | 50.56 | 50.54 | -0.12 |
| 989 | 6-Methylquinoline | 46.95 | 46.83 | 46.85 | 0.12 |
| 990 | cis-3-Methyl-3-hexene | 34.56 | 34.44 | 34.44 | 0.12 |
| 991 | Decahydro-2-naphthol | 45.49 | 45.37 | 45.36 | 0.12 |
| 992 | Diallyldimethylsilane | 48.34 | 48.22 | 48.28 | 0.12 |
| 993 | Ethyl phenoxyacetate | 49.02 | 48.90 | 48.89 | 0.12 |
| 994 | Isobutyl 3-chloropropanoate | 41.16 | 41.04 | 41.06 | 0.12 |
| 995 | 1,3-Dibromobenzene | 41.80 | 41.68 | 41.70 | 0.12 |
| 996 | Methylsilanetriol triacetate | 46.27 | 46.15 | 46.13 | 0.12 |
| 997 | Myristonitrile | 66.53 | 66.65 | 66.70 | -0.12 |
| 998 | trans-2-Undecene | 52.86 | 52.98 | 52.98 | -0.12 |
| 999 | Tributyl borate | 66.65 | 66.53 | 66.48 | 0.12 |
| 1000 | Vinylcyclohexane | 36.61 | 36.73 | 36.71 | -0.12 |
| 1001 | Vinyltriethoxysilane | 50.75 | 50.87 | 50.85 | -0.12 |
| 1002 | 1-Ethyl-1-methylcyclopentane | 37.11 | 36.98 | 37.04 | 0.13 |
| 1003 | 1-Isopropyl-4-methylbenzene | 45.34 | 45.47 | 45.50 | -0.13 |
| 1004 | 2,2,3,4,4-Pentamethylpentane | 48.20 | 48.33 | 48.37 | -0.13 |
| 1005 | 2,2,4,4-Tetramethylpentane | 43.87 | 43.74 | 43.78 | 0.13 |
| 1006 | 2,4-Dimethylhexane | 39.15 | 39.02 | 39.02 | 0.13 |
| 1007 | 2-Methyl-2-heptene | 39.19 | 39.06 | 39.06 | 0.13 |
| 1008 | 3-Methyldecane | 53.04 | 52.91 | 52.94 | 0.13 |
| 1009 | 3-Vinyl-7-oxabicyclo[4.1.0]heptane | 36.16 | 36.29 | 36.25 | -0.13 |
| 1010 | 4-Methylstyrene | 40.55 | 40.68 | 40.70 | -0.13 |
| 1011 | 5-Methyl-1-hexene | 34.36 | 34.23 | 34.24 | 0.13 |
| 1012 | 8-Methylquinoline | 46.70 | 46.83 | 46.88 | -0.13 |
| 1013 | Cyclohexylacetate | 38.87 | 38.74 | 38.71 | 0.13 |
| 1014 | Decylbenzene | 72.93 | 73.06 | 73.07 | -0.13 |
| 1015 | Diethyl disulfide | 36.65 | 36.52 | 36.50 | 0.13 |
| 1016 | Dimethyl isophthalate | 49.19 | 49.06 | 49.06 | 0.13 |
| 1017 | Hexamethyldisiloxane | 48.94 | 48.81 | 48.84 | 0.13 |
| 1018 | n-Butylcarbonate | 46.84 | 46.97 | 47.01 | -0.13 |
| 1019 | Phenylsilane | 37.44 | 37.31 |  | 0.13 |
| 1020 | Propylcyclohexane | 41.68 | 41.55 | 41.55 | 0.13 |
| 1021 | Triethyltriphenylcyclotrisiloxane | 129.17 | 129.30 | 129.51 | -0.13 |
| 1022 | 2-Bromo-5-chlorothiophene | 37.09 | 37.22 |  | -0.13 |
| 1023 | 1,4-Dichloro-2-butyne | 29.03 | 29.16 | 29.22 | -0.13 |
| 1024 | trans-1,4-Dichloro-2-butene | 30.39 | 30.26 | 30.24 | 0.13 |
| 1025 | 1-Hexyn-3-ol | 29.36 | 29.23 | 29.19 | 0.13 |
| 1026 | 2-Butanone oxime | 24.92 | 25.05 | 25.03 | -0.13 |
| 1027 | 2-Chloro-2-methylbutane | 30.23 | 30.10 | 30.08 | 0.13 |
| 1028 | 2-Hexanol | 31.32 | 31.19 | 31.19 | 0.13 |
| 1029 | 3-Chloro-1-propanol | 22.29 | 22.16 | 22.18 | 0.13 |
| 1030 | 4-Hexen-2-ol | 31.36 | 31.23 | 31.25 | 0.13 |
| 1031 | Acetylbromide | 19.82 | 19.69 |  | 0.13 |
| 1032 | Succinonitrile | 20.43 | 20.30 | 20.40 | 0.13 |
| 1033 | cis-3-Hexene | 29.75 | 29.88 | 29.88 | -0.13 |
| 1034 | Chlorocyclopentane | 28.02 | 27.89 | 27.90 | 0.13 |
| 1035 | Ethanesulfonylchloride | 25.61 | 25.48 | 25.46 | 0.13 |
| 1036 | Ethyl butanoate | 31.51 | 31.64 | 31.65 | -0.13 |
| 1037 | Ethyl chloride | 16.30 | 16.17 | 16.18 | 0.13 |
| 1038 | Methacrolein | 20.87 | 21.00 | 21.01 | -0.13 |
| 1039 | Propyl propanoate | 31.51 | 31.64 | 31.62 | -0.13 |
| 1040 | trans-3-Hexene | 29.75 | 29.88 | 29.90 | -0.13 |
| 1041 | Cyclopentyl formate | 29.53 | 29.66 | 29.66 | -0.13 |
| 1042 | Acetonitrile | 11.08 | 11.21 | 11.26 | -0.13 |
| 1043 | Chloroethylene | 15.52 | 15.65 | 15.66 | -0.13 |
| 1044 | 2-Chlorocrotonaldehyde | 25.95 | 26.08 | 26.09 | -0.13 |
| 1045 | 1,2,2,3-Tetrachloropropane | 35.30 | 35.17 | 35.12 | 0.13 |
| 1046 | 1,2-Diethylhydrazine | 28.01 | 27.88 | 27.78 | 0.13 |
| 1047 | 1,7-Dichloroheptane | 44.21 | 44.08 | 44.10 | 0.13 |
| 1048 | 1-Bromo-3-ethylbenzene | 43.46 | 43.59 | 43.60 | -0.13 |
| 1049 | 1-Ethyl-1-phenylhydrazine | 43.96 | 43.83 | 43.78 | 0.13 |
| 1050 | Methylenanthate | 40.99 | 40.86 | 40.85 | 0.13 |
| 1051 | 2,5-Dimethylheptane | 43.51 | 43.64 | 43.64 | -0.13 |
| 1052 | 2-Bromopropane | 23.94 | 23.81 | 23.83 | 0.13 |
| 1053 | 2-Chloroethyl ethyl ether | 27.26 | 27.13 | 27.12 | 0.13 |
| 1054 | 2-Heptanone | 34.79 | 34.92 | 34.92 | -0.13 |
| 1055 | 2-Methylbiphenyl | 56.25 | 56.38 | 56.38 | -0.13 |
| 1056 | 3-(Aminomethyl)-3,5,5-trimethylcyclohexanol | 51.14 | 51.01 | 51.03 | 0.13 |
| 1057 | 4-Bromoacetophenone | 43.92 | 44.05 | 44.06 | -0.13 |
| 1058 | 4-Ethyltoluene | 40.75 | 40.88 | 40.86 | -0.13 |
| 1059 | 4-Methyloctane | 43.80 | 43.67 | 43.67 | 0.13 |
| 1060 | 5-Amino-1-pentanol | 29.88 | 30.01 | 30.00 | -0.13 |
| 1061 | Benzedrine | 44.03 | 44.16 | 44.18 | -0.13 |
| 1062 | Butylisopropylether | 36.03 | 36.16 | 36.16 | -0.13 |
| 1063 | Butyltrichlorosilane | 43.18 | 43.31 | 43.36 | -0.13 |
| 1064 | Methylcaprylate | 45.35 | 45.48 | 45.49 | -0.13 |
| 1065 | cis-Decalin | 43.89 | 44.02 | 44.02 | -0.13 |
| 1066 | Chlorocyclohexane | 32.64 | 32.51 | 32.52 | 0.13 |
| 1067 | 2,2'-Dichlorodiisopropyl ether | 41.01 | 41.14 | 41.19 | -0.13 |
| 1068 | Diethyl 2-butylmalonate | 56.64 | 56.51 | 56.53 | 0.13 |
| 1069 | Diethyl malonate | 37.93 | 38.06 | 38.07 | -0.13 |
| 1070 | Ethyl 2-ethylcaproate | 49.96 | 50.09 | 50.07 | -0.13 |
| 1071 | Ethyl bromide | 19.04 | 19.17 | 19.17 | -0.13 |
| 1072 | Ethyldimethylamine | 24.54 | 24.67 | 24.66 | -0.13 |
| 1073 | Isophorone | 42.17 | 42.04 | 42.07 | 0.13 |
| 1074 | Isopropyl 2-chloropropanoate | 36.56 | 36.43 | 36.46 | 0.13 |
| 1075 | n-Butylbenzoate | 51.89 | 51.76 | 51.72 | 0.13 |
| 1076 | n-Butylchloroacetate | 36.32 | 36.45 | 36.47 | -0.13 |
| 1077 | Pentyl propanoate | 40.75 | 40.88 | 40.86 | -0.13 |
| 1078 | Propham | 51.48 | 51.35 | 51.32 | 0.13 |
| 1079 | trans-3-Methyl-3-hexene | 34.57 | 34.44 | 34.49 | 0.13 |
| 1080 | Triethoxymethane | 39.63 | 39.50 | 39.47 | 0.13 |
| 1081 | Triethoxymethylsilane | 46.50 | 46.63 | 46.65 | -0.13 |
| 1082 | Tripropylamine | 47.78 | 47.65 | 47.67 | 0.13 |
| 1083 | 2-Heptanamine | 38.03 | 37.90 | 37.88 | 0.13 |
| 1084 | 2,3-Dihydro-1,1,3-trimethyl-3-phenyl-1H-indene | 77.27 | 77.40 | 77.35 | -0.13 |
| 1085 | Guaiol | 68.57 | 68.44 | 68.53 | 0.13 |
| 1086 | 1-Bromohexadecane | 83.99 | 83.85 | 83.86 | 0.14 |
| 1087 | 2,2-Dimethylpentane | 34.62 | 34.48 | 34.49 | 0.14 |
| 1088 | N-Propylaniline | 45.12 | 44.98 | 44.96 | 0.14 |
| 1089 | 1-Bromo-2-fluoroethane | 18.99 | 19.13 | 19.12 | -0.14 |
| 1090 | 2,2-Dichloroethanol | 22.54 | 22.40 | 22.41 | 0.14 |
| 1091 | 3-Methyl-2-butanamine | 28.49 | 28.63 | 28.63 | -0.14 |
| 1092 | Methyl vinyl ether | 17.13 | 16.99 | 16.91 | 0.14 |
| 1093 | trans-2-Penten-1-ol | 26.49 | 26.63 | 26.65 | -0.14 |
| 1094 | 1,3-Bis(trifluoromethyl)benzene | 36.94 | 37.08 | 36.94 | -0.14 |
| 1095 | 1-Chloro-4-ethoxybenzene | 42.66 | 42.52 | 42.50 | 0.14 |
| 1096 | 1-Chloroheptane | 39.41 | 39.27 | 39.27 | 0.14 |
| 1097 | 1-Methyl-2-propylbenzene | 45.36 | 45.50 | 45.50 | -0.14 |
| 1098 | 1-Phenyl-1-dodecanone | 82.62 | 82.76 | 82.88 | -0.14 |
| 1099 | 2,2,3-Trimethylpentane | 38.93 | 39.07 | 39.08 | -0.14 |
| 1100 | 2,3-Dimethylthiophene | 33.99 | 34.13 | 34.23 | -0.14 |
| 1101 | 2-Chloroanisole | 38.02 | 37.88 | 37.91 | 0.14 |
| 1102 | t-Butyl cyanide | 25.23 | 25.09 | 25.10 | 0.14 |
| 1103 | 2-Methyl-2-hexanol | 36.03 | 35.89 | 35.86 | 0.14 |
| 1104 | 2-Methylpentanedinitrile | 29.65 | 29.51 | 29.51 | 0.14 |
| 1105 | 2-Pentanol | 26.71 | 26.57 | 26.60 | 0.14 |
| 1106 | 2-Propylpyridine | 38.59 | 38.45 | 38.46 | 0.14 |
| 1107 | 2-Pyridinecarboxaldehyde | 30.01 | 29.87 | 29.94 | 0.14 |
| 1108 | 2-Thiocresol | 39.14 | 39.28 | 39.30 | -0.14 |
| 1109 | 3,3,3-Trichloro-1-propene | 30.36 | 30.50 | 30.48 | -0.14 |
| 1110 | 2-Pyridinepropanol | 39.96 | 39.82 | 39.87 | 0.14 |
| 1111 | 3-Chloropropyl chloroformate | 32.36 | 32.22 | 32.20 | 0.14 |
| 1112 | 3-Thiocresol | 39.14 | 39.28 | 39.29 | -0.14 |
| 1113 | 1-(4-Pyridinyl)ethanone | 34.01 | 34.15 | 34.14 | -0.14 |
| 1114 | 4-Bromo-1-butene | 28.07 | 28.21 | 28.24 | -0.14 |
| 1115 | 4-Chloro-2-pentene | 30.19 | 30.05 | 30.03 | 0.14 |
| 1116 | 4-Morpholinepropanamine | 41.29 | 41.43 | 41.45 | -0.14 |
| 1117 | 5-Bromo-1-pentene | 32.69 | 32.83 | 32.84 | -0.14 |
| 1118 | 5-Oxohexanoic acid | 31.78 | 31.92 | 31.92 | -0.14 |
| 1119 | 6-Methyl-3-heptanol | 40.54 | 40.40 | 40.40 | 0.14 |
| 1120 | 7-Chloro-1-heptanol | 40.78 | 40.64 | 40.64 | 0.14 |
| 1121 | Acetaldoxime | 15.69 | 15.83 | 15.90 | -0.14 |
| 1122 | Ethyl acrylate | 26.68 | 26.82 | 26.81 | -0.14 |
| 1123 | cis-1,2-Cyclohexanediamine | 34.74 | 34.60 | 34.60 | 0.14 |
| 1124 | Demeton-S-methyl | 57.04 | 56.90 |  | 0.14 |
| 1125 | Dicyclohexylamine | 56.88 | 56.74 | 56.65 | 0.14 |
| 1126 | Diethylenetriamine | 30.68 | 30.82 | 30.85 | -0.14 |
| 1127 | Diethyl oxalate | 33.58 | 33.44 | 33.42 | 0.14 |
| 1128 | Diethyl phthalate | 58.20 | 58.34 | 58.32 | -0.14 |
| 1129 | Diisopropyl disulfide | 46.18 | 46.32 | 46.20 | -0.14 |
| 1130 | Dimethylperoxide | 15.40 | 15.54 | 15.56 | -0.14 |
| 1131 | Dipropyl disulfide | 45.90 | 45.76 | 45.68 | 0.14 |
| 1132 | Undecylcyanide | 57.55 | 57.41 | 57.41 | 0.14 |
| 1133 | Ethyl methyl carbonate | 23.71 | 23.85 | 23.86 | -0.14 |
| 1134 | Ethyl acetate | 22.26 | 22.40 | 22.38 | -0.14 |
| 1135 | Ethyl cyanoacetate | 26.73 | 26.87 | 26.85 | -0.14 |
| 1136 | Fluoroethane | 11.18 | 11.32 | 11.32 | -0.14 |
| 1137 | Ethyl formate | 17.80 | 17.94 | 17.96 | -0.14 |
| 1138 | Heptane | 34.60 | 34.46 | 34.53 | 0.14 |
| 1139 | Isopropyl laurate | 73.06 | 73.20 | 73.32 | -0.14 |
| 1140 | Isopropyl myristate | 82.30 | 82.44 | 82.43 | -0.14 |
| 1141 | Isopropylnitrate | 24.16 | 24.02 | 24.04 | 0.14 |
| 1142 | Methanol | 8.23 | 8.09 | 8.09 | 0.14 |
| 1143 | Methyl 2-chloroacrylate | 26.82 | 26.96 | 26.95 | -0.14 |
| 1144 | Methyl carbamate | 16.46 | 16.60 | 16.65 | -0.14 |
| 1145 | Methyl isopentanoate | 31.45 | 31.59 | 31.60 | -0.14 |
| 1146 | Glycerol 1-butanoate | 38.84 | 38.98 | 39.02 | -0.14 |
| 1147 | N-Methyl-2-propyne-1-amine | 21.94 | 22.08 | 22.13 | -0.14 |
| 1148 | n-Nonanal | 44.22 | 44.36 | 44.37 | -0.14 |
| 1149 | Amylamine | 28.78 | 28.64 | 28.67 | 0.14 |
| 1150 | Piperitone | 46.72 | 46.58 | 46.61 | 0.14 |
| 1151 | Propylbenzenesulfonate | 50.19 | 50.33 | 50.50 | -0.14 |
| 1152 | 2-Chlorobutane | 25.53 | 25.39 | 25.38 | 0.14 |
| 1153 | s-Butylmethylether | 26.76 | 26.90 | 26.90 | -0.14 |
| 1154 | s-Butylnitrite | 26.80 | 26.66 | 26.62 | 0.14 |
| 1155 | 2-Methyl-2-propanol | 22.17 | 22.03 | 22.05 | 0.14 |
| 1156 | Tetrachlorothiophene | 44.05 | 43.91 | 43.93 | 0.14 |
| 1157 | Thiane | 30.83 | 30.97 | 30.99 | -0.14 |
| 1158 | trans-1,4-Dimethylcyclohexane | 36.76 | 36.90 | 36.90 | -0.14 |
| 1159 | Trichloroacetaldehyde | 26.60 | 26.74 | 26.75 | -0.14 |
| 1160 | Triphenylphosphane | 87.45 | 87.31 |  | 0.14 |
| 1161 | Bis(1-chloroethyl)ether | 32.63 | 32.48 | 32.91 | 0.15 |
| 1162 | (Propylthio)benzene | 49.05 | 49.20 | 49.21 | -0.15 |
| 1163 | 1,1,1-Trichloroacetone | 31.01 | 31.16 | 31.11 | -0.15 |
| 1164 | 1,1-Dimethylindane | 47.87 | 48.02 | 48.03 | -0.15 |
| 1165 | 1,2-Dibromopropane | 31.77 | 31.62 | 31.64 | 0.15 |
| 1166 | 1,2-Dichloropropane | 25.73 | 25.58 | 25.57 | 0.15 |
| 1167 | 1,3-Diaminopropane | 22.97 | 22.82 | 22.82 | 0.15 |
| 1168 | 1,6-Heptadiyne | 30.39 | 30.54 | 30.66 | -0.15 |
| 1169 | 1,8-Dibromooctane | 54.55 | 54.70 | 54.72 | -0.15 |
| 1170 | 1-Bromo-4-fluorobenzene | 33.99 | 34.14 | 34.14 | -0.15 |
| 1171 | 1-Butoxy-2-propanol | 37.68 | 37.53 | 37.55 | 0.15 |
| 1172 | 2,2-Dichloropropane | 25.40 | 25.55 | 25.45 | -0.15 |
| 1173 | 2,2-Dimethylbutanoic acid | 31.33 | 31.48 | 31.50 | -0.15 |
| 1174 | 2,2-Dimethylhexane | 39.25 | 39.10 | 39.10 | 0.15 |
| 1175 | 2-Chloropentane | 30.16 | 30.01 | 30.04 | 0.15 |
| 1176 | 2-Decanol | 49.82 | 49.67 | 49.75 | 0.15 |
| 1177 | 2-Ethyl-3-methl-1-butene | 34.02 | 34.17 | 34.18 | -0.15 |
| 1178 | 2-Ethylhexyl methacrylate | 58.92 | 59.07 | 59.05 | -0.15 |
| 1179 | 2-Fluorobenzoyl chloride | 37.26 | 37.11 | 37.09 | 0.15 |
| 1180 | 2-Methylhexane | 34.58 | 34.43 | 34.43 | 0.15 |
| 1181 | beta-Methylbenzeneethanamine | 43.96 | 44.11 | 44.09 | -0.15 |
| 1182 | 2-Trifluoromethylaniline | 35.77 | 35.92 | 35.99 | -0.15 |
| 1183 | 3,3,5-Trimethylcyclohexanone | 41.88 | 42.03 | 42.04 | -0.15 |
| 1184 | 3-Ethyl-2,4-pentanedione | 35.50 | 35.35 | 35.39 | 0.15 |
| 1185 | 3-Ethylpentane | 34.28 | 34.43 | 34.46 | -0.15 |
| 1186 | 3-Methoxybenzenemethanol | 39.22 | 39.37 | 39.39 | -0.15 |
| 1187 | 3-Methyl-1-hexene | 34.38 | 34.23 | 34.25 | 0.15 |
| 1188 | 3-Methyl-2-pentanol | 31.01 | 31.16 | 31.16 | -0.15 |
| 1189 | 4-Formylmorpholine | 28.62 | 28.77 | 28.81 | -0.15 |
| 1190 | 4-(Methylthio)aniline | 44.05 | 44.20 | 44.30 | -0.15 |
| 1191 | 4-Trifluoromethylaniline | 35.77 | 35.92 | 35.89 | -0.15 |
| 1192 | 4-Vinylpyridine | 33.64 | 33.49 | 33.50 | 0.15 |
| 1193 | Caproleic acid | 49.59 | 49.74 | 49.76 | -0.15 |
| 1194 | 2-Bromo-1,1-dimethoxyethane | 31.46 | 31.61 | 31.61 | -0.15 |
| 1195 | Butylcyclopentane | 41.70 | 41.55 | 41.55 | 0.15 |
| 1196 | cis-2,3-Epoxybutane | 20.31 | 20.16 | 20.19 | 0.15 |
| 1197 | Di-n-propylsulfoxide | 38.53 | 38.38 |  | 0.15 |
| 1198 | Diethyleneglycol bischloroformate | 45.03 | 45.18 | 45.16 | -0.15 |
| 1199 | Diethylsilane | 30.71 | 30.56 | 30.54 | 0.15 |
| 1200 | Ethyl hydrogen succinate | 33.11 | 33.26 | 33.25 | -0.15 |
| 1201 | Ethylamine | 14.93 | 14.78 | 14.76 | 0.15 |
| 1202 | Ethylbenzene | 35.95 | 36.10 | 36.09 | -0.15 |
| 1203 | Ethylnitrite | 17.59 | 17.44 | 17.41 | 0.15 |
| 1204 | Isopentylbenzene | 49.78 | 49.93 | 49.92 | -0.15 |
| 1205 | m-Tolualdehyde | 37.07 | 36.92 | 36.89 | 0.15 |
| 1206 | 2-Methylacrylonitrile | 20.34 | 20.19 | 20.24 | 0.15 |
| 1207 | Methylcyclopentane | 27.84 | 27.69 | 27.73 | 0.15 |
| 1208 | n-Butyl acetate | 31.49 | 31.64 | 31.62 | -0.15 |
| 1209 | n-Butylethylether | 31.71 | 31.56 | 31.57 | 0.15 |
| 1210 | N-Ethylacetamide | 24.08 | 24.23 | 24.20 | -0.15 |
| 1211 | 4-Methoxy-4-methyl-2-pentanone | 36.53 | 36.68 | 36.71 | -0.15 |
| 1212 | Pentylcyclopentane | 46.32 | 46.17 | 46.18 | 0.15 |
| 1213 | Ethoxybenzene | 37.71 | 37.86 | 37.85 | -0.15 |
| 1214 | 1-Phenyl-1-propanone | 41.03 | 41.18 | 41.21 | -0.15 |
| 1215 | Propylcyclopentane | 37.08 | 36.93 | 36.99 | 0.15 |
| 1216 | Tetrachloroethylene | 30.35 | 30.50 | 30.56 | -0.15 |
| 1217 | Tetraethoxymethane | 49.67 | 49.82 |  | -0.15 |
| 1218 | Tetraethylsilane | 48.37 | 48.52 | 48.61 | -0.15 |
| 1219 | 1,2-Dichloro-2-methylpropane | 30.44 | 30.29 | 30.26 | 0.15 |
| 1220 | 1-Cyclopentene-1-carboxaldehyde | 28.51 | 28.36 | 28.32 | 0.15 |
| 1221 | 2,2-Dimethoxyethanamine | 27.37 | 27.22 | 27.22 | 0.15 |
| 1222 | 3-Mercaptopropionic acid | 25.37 | 25.22 | 25.19 | 0.15 |
| 1223 | Chloroethylene carbonate | 22.06 | 22.21 | 22.26 | -0.15 |
| 1224 | Acetone | 16.29 | 16.44 | 16.45 | -0.15 |
| 1225 | Dimethyl sulfone | 20.47 | 20.62 | 20.54 | -0.15 |
| 1226 | 4-Methylpent-3-en-2-one | 30.13 | 30.28 | 30.28 | -0.15 |
| 1227 | N,N-Dimethylacrylamide | 28.91 | 29.06 | 29.01 | -0.15 |
| 1228 | Thiirane | 17.26 | 17.11 | 17.09 | 0.15 |
| 1229 | 3,4-Dichlorotoluene | 40.65 | 40.80 | 40.79 | -0.15 |
| 1230 | 3,4-Dimethyloctane | 48.41 | 48.26 | 48.32 | 0.15 |
| 1231 | 4-Isopropylcyclohexanone | 41.83 | 41.98 | 42.00 | -0.15 |
| 1232 | Dibutyl tartrate | 64.01 | 63.86 | 63.88 | 0.15 |
| 1233 | Hexyl butanoate | 49.97 | 50.12 | 50.11 | -0.15 |
| 1234 | Triethanolamine | 38.05 | 37.90 | 37.87 | 0.15 |
| 1235 | 1,4-Butanediol diacetate | 42.52 | 42.68 | 42.66 | -0.16 |
| 1236 | 1-Bromoheptadecane | 88.63 | 88.47 | 88.47 | 0.16 |
| 1237 | 1-Ethyl-4-methoxybenzene | 42.46 | 42.62 | 42.64 | -0.16 |
| 1238 | 1-Isopropyl-3-methylbenzene | 45.31 | 45.47 | 45.50 | -0.16 |
| 1239 | 1-Nonadecene | 89.86 | 89.70 | 89.72 | 0.16 |
| 1240 | 2,3-Lutidine | 34.15 | 33.99 | 33.93 | 0.16 |
| 1241 | 2,6-Diisopropylaniline | 58.55 | 58.71 | 58.72 | -0.16 |
| 1242 | 2-Butanone (1-methylpropylidene)hydrazone | 44.94 | 44.78 | 44.52 | 0.16 |
| 1243 | 3-Methylundecane | 57.69 | 57.53 | 57.55 | 0.16 |
| 1244 | 3-Pentanethiol | 32.96 | 33.12 | 33.22 | -0.16 |
| 1245 | 4-Heptanol | 35.97 | 35.81 | 35.81 | 0.16 |
| 1246 | 5-Butyldocosane | 122.37 | 122.21 | 122.21 | 0.16 |
| 1247 | Anise alcohol | 39.21 | 39.37 | 39.39 | -0.16 |
| 1248 | Bis(2-bromoethyl) ether | 37.78 | 37.94 | 37.98 | -0.16 |
| 1249 | Di-t-butylsulfide | 48.29 | 48.13 | 48.13 | 0.16 |
| 1250 | Ethyl-n-hexylether | 40.96 | 40.80 | 40.81 | 0.16 |
| 1251 | 3-Methyl-1-butanethiol | 32.97 | 32.81 | 32.81 | 0.16 |
| 1252 | Methyl isobutanoate | 26.81 | 26.97 | 26.96 | -0.16 |
| 1253 | Methyloleate | 91.56 | 91.72 | 91.70 | -0.16 |
| 1254 | N-(2-Pyridinylmethyl)-2-pyridinemethanamine | 59.52 | 59.68 | 59.62 | -0.16 |
| 1255 | 1,2-Dibromobenzene | 41.52 | 41.68 | 41.74 | -0.16 |
| 1256 | trans-3-Methylcyclohexanol | 33.82 | 33.66 | 33.72 | 0.16 |
| 1257 | Triisopentylborate | 80.14 | 80.30 | 80.32 | -0.16 |
| 1258 | 1-Ethoxy-2-methoxyethane | 28.80 | 28.64 | 28.67 | 0.16 |
| 1259 | 1-Pentene | 24.86 | 25.02 | 25.02 | -0.16 |
| 1260 | 2,3-Dihydro-1,4-dioxin | 20.82 | 20.98 | 21.10 | -0.16 |
| 1261 | 2,3-Pentanediol | 28.08 | 27.92 | 27.92 | 0.16 |
| 1262 | 3-Methyl-3-pentanol | 31.11 | 31.27 | 31.28 | -0.16 |
| 1263 | 4-Chloro-1-butene | 25.05 | 25.21 | 25.20 | -0.16 |
| 1264 | Dimethylphosphate | 23.51 | 23.35 | 23.72 | 0.16 |
| 1265 | Ethylnitrate | 19.26 | 19.42 | 19.43 | -0.16 |
| 1266 | Mecrylate | 26.75 | 26.59 | 26.59 | 0.16 |
| 1267 | Pentyl nitrite | 31.14 | 31.30 | 31.17 | -0.16 |
| 1268 | Propyl nitrite | 22.22 | 22.06 | 22.03 | 0.16 |
| 1269 | Trimethylphosphite | 29.20 | 29.04 | 29.04 | 0.16 |
| 1270 | Dimethyl cyclobutane- 1,l -dicarboxylate | 40.70 | 40.54 | 40.56 | 0.16 |
| 1271 | 1,2-Benzopyran | 40.40 | 40.56 | 40.55 | -0.16 |
| 1272 | 1,2-Epoxy-4-(epoxyethyl)cyclohexane | 36.23 | 36.07 | 36.07 | 0.16 |
| 1273 | 1-Chlorooctane | 44.05 | 43.89 | 43.90 | 0.16 |
| 1274 | 1-Iododecane | 61.15 | 61.31 | 61.40 | -0.16 |
| 1275 | 1-Methyl-4-isopropylcyclohexanol | 47.73 | 47.57 | 47.58 | 0.16 |
| 1276 | 2,2,3-Trimethylhexane | 43.85 | 43.69 | 43.70 | 0.16 |
| 1277 | 2-Methyl-3-heptanol | 40.56 | 40.40 | 40.40 | 0.16 |
| 1278 | 3,3-Dimethylpentane | 34.32 | 34.48 | 34.49 | -0.16 |
| 1279 | 3,7-Dimethyl-1,7-octanediol | 50.93 | 51.09 | 51.05 | -0.16 |
| 1280 | p-Toluidine | 35.67 | 35.51 | 35.61 | 0.16 |
| 1281 | 5-Bromoacenaphthene | 59.55 | 59.39 | 59.46 | 0.16 |
| 1282 | Azelaic acid | 47.10 | 46.94 | 46.92 | 0.16 |
| 1283 | Camphor | 44.39 | 44.55 | 44.53 | -0.16 |
| 1284 | cis-1-Methyl-4-isopropylcyclohexane | 46.27 | 46.11 | 46.14 | 0.16 |
| 1285 | Cycloheptane | 32.18 | 32.34 | 32.34 | -0.16 |
| 1286 | Di-t-butyl ether | 40.76 | 40.92 | 41.02 | -0.16 |
| 1287 | Indene | 38.64 | 38.80 | 38.80 | -0.16 |
| 1288 | DEET | 58.50 | 58.66 | 58.63 | -0.16 |
| 1289 | n-Nonylamine | 47.28 | 47.12 | 47.19 | 0.16 |
| 1290 | Azidobenzene | 35.41 | 35.25 | 35.17 | 0.16 |
| 1291 | trans-5-Methyl-2-hexene | 34.63 | 34.47 | 34.51 | 0.16 |
| 1292 | Vinyltriacetoxysilane | 50.55 | 50.39 | 50.37 | 0.16 |
| 1293 | (Bromomethyl)cyclohexane | 39.95 | 40.12 | 40.12 | -0.17 |
| 1294 | 2,2-Dimethyl-1-phenyl-1-propanone | 50.27 | 50.44 | 50.44 | -0.17 |
| 1295 | o-Toluidine | 35.34 | 35.51 | 35.49 | -0.17 |
| 1296 | 2-Undecanone | 53.23 | 53.40 | 53.42 | -0.17 |
| 1297 | 3,4-Diethylhexane | 48.09 | 48.26 | 48.26 | -0.17 |
| 1298 | 4-Ethylpyridine | 33.52 | 33.69 | 33.72 | -0.17 |
| 1299 | cis-4-Octene | 38.95 | 39.12 | 39.16 | -0.17 |
| 1300 | Ethyl 2-bromobutanoate | 39.30 | 39.47 | 39.47 | -0.17 |
| 1301 | Isopentylacetate | 36.40 | 36.23 | 36.22 | 0.17 |
| 1302 | N,N-Dimethyl-m-toluidine | 45.72 | 45.55 | 45.59 | 0.17 |
| 1303 | 1,1,2-Trichlorotrifluoroethane | 26.17 | 26.34 | 26.40 | -0.17 |
| 1304 | 2,2,3,3-Tetrafluoro-1-propanol | 17.62 | 17.79 | 17.98 | -0.17 |
| 1305 | 2,2-Dimethylpropylamine | 28.49 | 28.66 | 28.67 | -0.17 |
| 1306 | 2-Chloroallyl alcohol | 21.76 | 21.93 | 21.97 | -0.17 |
| 1307 | Dimethyldimethoxysilane | 31.51 | 31.68 | 31.61 | -0.17 |
| 1308 | Ethyl 2-propynoate | 25.23 | 25.06 | 25.06 | 0.17 |
| 1309 | Isobutyl formate | 27.32 | 27.15 | 27.14 | 0.17 |
| 1310 | 1-Propanethiol | 23.77 | 23.60 | 23.60 | 0.17 |
| 1311 | Tetrahydro-3-furanol | 21.72 | 21.55 | 21.55 | 0.17 |
| 1312 | (2,2-Dimethylpropyl)benzene | 49.81 | 49.98 | 50.02 | -0.17 |
| 1313 | Acetic acid 2-bromoethylester | 30.04 | 30.21 | 30.23 | -0.17 |
| 1314 | (Chloromethyl)trimethylsilane | 35.13 | 34.96 |  | 0.17 |
| 1315 | 1,3,5-Cycloheptatriene | 32.29 | 32.46 | 32.46 | -0.17 |
| 1316 | Isopropanolamine | 20.92 | 20.75 | 20.73 | 0.17 |
| 1317 | 1-Bromooctadecane | 93.26 | 93.09 | 93.11 | 0.17 |
| 1318 | 1-Butanol | 22.14 | 21.97 | 21.96 | 0.17 |
| 1319 | 1-Fluoro-2-(trichloromethyl)benzene | 46.32 | 46.15 | 46.18 | 0.17 |
| 1320 | 1-Methoxy-4-propylbenzene | 47.07 | 47.24 | 47.28 | -0.17 |
| 1321 | Allyl alcohol | 16.98 | 17.15 | 17.15 | -0.17 |
| 1322 | 2,3-Dichlorobutane | 30.35 | 30.18 | 30.16 | 0.17 |
| 1323 | 2,3-Dimethyl-1-pentene | 34.00 | 34.17 | 34.20 | -0.17 |
| 1324 | 2,3-Dimethyl-2-butene | 29.59 | 29.76 | 29.76 | -0.17 |
| 1325 | 2,4-Xylidine | 40.12 | 40.29 | 40.30 | -0.17 |
| 1326 | 2-Chloro-2-propenenitrile | 20.58 | 20.41 | 20.46 | 0.17 |
| 1327 | 2-Ethyltetrahydrofuran | 29.25 | 29.42 | 29.44 | -0.17 |
| 1328 | Tiglic acid | 26.99 | 26.82 | 26.81 | 0.17 |
| 1329 | 2-Methyl-2-hexene | 34.61 | 34.44 | 34.49 | 0.17 |
| 1330 | alpha-Methylstyrene | 40.63 | 40.46 | 40.44 | 0.17 |
| 1331 | 3-Decanol | 49.84 | 49.67 | 49.67 | 0.17 |
| 1332 | 4-Chloroheptane | 39.42 | 39.25 | 39.28 | 0.17 |
| 1333 | 5-Methyl-2-hexanone oxime | 38.71 | 38.88 | 38.92 | -0.17 |
| 1334 | 6-Methyl-1-heptene | 39.02 | 38.85 | 38.88 | 0.17 |
| 1335 | Allyl ethyl ether | 26.57 | 26.74 | 26.75 | -0.17 |
| 1336 | alpha-Terpinene | 45.96 | 46.13 | 46.17 | -0.17 |
| 1337 | Butyrophenone | 45.63 | 45.80 | 45.81 | -0.17 |
| 1338 | Butyl ethyl sulfide | 37.88 | 37.71 | 37.67 | 0.17 |
| 1339 | cis-1,2-Dibromoethylene | 26.07 | 26.24 | 26.30 | -0.17 |
| 1340 | cis-1-Phenyl-1-propene | 40.93 | 40.76 | 40.76 | 0.17 |
| 1341 | Bis(2-ethylhexyl) adipate | 107.47 | 107.30 | 107.26 | 0.17 |
| 1342 | Dibutyl succinate | 60.99 | 61.16 | 61.22 | -0.17 |
| 1343 | Dibutyldisulfide | 55.17 | 55.00 | 55.00 | 0.17 |
| 1344 | Diethyl 2-propylmalonate | 51.72 | 51.89 | 51.88 | -0.17 |
| 1345 | Diethyleneglycol diaminopropyl ether | 60.49 | 60.32 | 60.34 | 0.17 |
| 1346 | Diethyleneglycol-di-n-butylether | 62.89 | 62.72 | 62.72 | 0.17 |
| 1347 | Ethyl ether | 22.49 | 22.32 | 22.31 | 0.17 |
| 1348 | Dimethyldiacetoxysilane | 40.81 | 40.64 | 40.63 | 0.17 |
| 1349 | Ethoxytriethylsilane | 49.10 | 49.27 | 49.34 | -0.17 |
| 1350 | Ethyl 2,4-dibromobutanoate | 47.11 | 47.28 | 47.28 | -0.17 |
| 1351 | Ethoglucid | 63.77 | 63.60 | 63.54 | 0.17 |
| 1352 | Isopropyl formate | 22.71 | 22.54 | 22.52 | 0.17 |
| 1353 | N,N-Diethyl-1,3-propanediamine | 42.00 | 41.83 | 41.82 | 0.17 |
| 1354 | N-Butylaniline | 49.77 | 49.60 | 49.58 | 0.17 |
| 1355 | Octylbenzene | 63.65 | 63.82 | 63.83 | -0.17 |
| 1356 | Ethyl 2-picolinate | 40.42 | 40.25 | 40.29 | 0.17 |
| 1357 | s-Butyl propanoate | 36.07 | 36.24 | 36.26 | -0.17 |
| 1358 | t-Butylformate | 27.07 | 27.24 | 27.23 | -0.17 |
| 1359 | trans-1,2-Dichloroethylene | 20.55 | 20.38 | 20.40 | 0.17 |
| 1360 | Trifluoroacetoacetic acid ethylester | 33.75 | 33.58 | 33.61 | 0.17 |
| 1361 | Di-n-butylsebacate | 88.70 | 88.88 | 88.90 | -0.18 |
| 1362 | Acetic acid 1-chloroethylester | 27.30 | 27.48 | 27.48 | -0.18 |
| 1363 | (3-Chloropropyl)trimethoxysilane | 46.53 | 46.71 | 46.79 | -0.18 |
| 1364 | 1,1,1,2-Tetrachloroethane | 31.07 | 30.89 | 30.89 | 0.18 |
| 1365 | Veratrole | 39.56 | 39.74 | 39.74 | -0.18 |
| 1366 | 1-Bromo-2-fluorobenzene | 33.96 | 34.14 | 34.18 | -0.18 |
| 1367 | 1-Chloro-1-ethoxyethane | 27.58 | 27.40 | 27.39 | 0.18 |
| 1368 | n-Decylamine | 51.92 | 51.74 | 51.74 | 0.18 |
| 1369 | 1-Penten-3-ol | 26.19 | 26.37 | 26.38 | -0.18 |
| 1370 | 1-Piperidineethanol | 37.48 | 37.66 | 37.69 | -0.18 |
| 1371 | 2,2-Dimethoxypropane | 28.35 | 28.53 | 28.86 | -0.18 |
| 1372 | 2,3,4-Trimethyl-2-pentene | 38.79 | 38.97 | 38.97 | -0.18 |
| 1373 | 2,3-Dimethyl-2-pentene | 34.20 | 34.38 | 34.45 | -0.18 |
| 1374 | 2,4-Dimethyl-3-ethylpentane | 43.43 | 43.61 | 43.66 | -0.18 |
| 1375 | 2-Bromonaphthalene | 51.77 | 51.95 | 52.01 | -0.18 |
| 1376 | 2-Butylthiophene | 43.25 | 43.43 | 43.37 | -0.18 |
| 1377 | 2-Chlorocyclopentanone | 28.17 | 28.35 | 28.36 | -0.18 |
| 1378 | 2-Cyclohexylcyclohexanone | 53.54 | 53.72 | 53.76 | -0.18 |
| 1379 | 2-Octanol | 40.25 | 40.43 | 40.43 | -0.18 |
| 1380 | 3,3-Dimethylcyclohexanol | 38.51 | 38.33 | 38.35 | 0.18 |
| 1381 | 3,5-Dimethylheptane | 43.82 | 43.64 | 43.64 | 0.18 |
| 1382 | 3-Ethyl-2-methyl-1-pentene | 38.61 | 38.79 | 38.84 | -0.18 |
| 1383 | 3-Ethyl-2-methylpentane | 38.84 | 39.02 | 39.06 | -0.18 |
| 1384 | 3-Fluoropyridine | 24.06 | 24.24 | 24.24 | -0.18 |
| 1385 | 3-Methylbutyl 2-chloropropanoate | 45.82 | 45.64 | 45.64 | 0.18 |
| 1386 | 3-Methylcyclopentene | 27.55 | 27.73 | 27.73 | -0.18 |
| 1387 | 4-Ethyl-2-methylhexane | 43.82 | 43.64 | 43.69 | 0.18 |
| 1388 | 4-Ethylheptane | 43.85 | 43.67 | 43.67 | 0.18 |
| 1389 | 4-Penten-2-ol | 26.19 | 26.37 | 26.43 | -0.18 |
| 1390 | 5-Methyl-2-isopropylcyclohexanol acetate | 57.31 | 57.13 | 57.14 | 0.18 |
| 1391 | 5-Nonanol | 45.23 | 45.05 | 45.12 | 0.18 |
| 1392 | 6-Chloro-1-hexanol | 36.20 | 36.02 | 36.06 | 0.18 |
| 1393 | Allyldiethylamine | 38.39 | 38.21 | 38.18 | 0.18 |
| 1394 | Benzaldehyde | 32.32 | 32.14 | 32.12 | 0.18 |
| 1395 | Benzonitrile | 31.51 | 31.33 | 31.29 | 0.18 |
| 1396 | 1,5-Pentanediamine | 32.24 | 32.06 | 32.02 | 0.18 |
| 1397 | Methylcaproate | 36.06 | 36.24 | 36.23 | -0.18 |
| 1398 | Chlorodimethylphenylsilane | 49.33 | 49.51 | 49.50 | -0.18 |
| 1399 | cis-(1-Methyl-1-propenyl)benzene | 45.14 | 45.32 | 45.30 | -0.18 |
| 1400 | Cyclooctyne | 35.72 | 35.90 | 35.84 | -0.18 |
| 1401 | Dichloro(dichloromethyl)methylsilane | 39.13 | 39.31 | 39.36 | -0.18 |
| 1402 | Dichloroacetic acid | 22.83 | 22.65 | 22.63 | 0.18 |
| 1403 | Dipropoxymethane | 37.89 | 37.71 | 37.70 | 0.18 |
| 1404 | Ethanesulfonic acid | 21.48 | 21.66 |  | -0.18 |
| 1405 | Ethyl 2,2-dimethylpropionate | 36.10 | 36.28 | 36.27 | -0.18 |
| 1406 | Ethyl pentyl ether | 36.36 | 36.18 | 36.18 | 0.18 |
| 1407 | 2-Chloroethanol | 17.72 | 17.54 | 17.53 | 0.18 |
| 1408 | Hexyl pentanoate | 54.92 | 54.74 | 54.72 | 0.18 |
| 1409 | Hexylcyclopentane | 50.97 | 50.79 | 50.80 | 0.18 |
| 1410 | Isobutylmercaptan | 28.37 | 28.19 | 28.26 | 0.18 |
| 1411 | 2-Methylheptane | 39.23 | 39.05 | 39.05 | 0.18 |
| 1412 | Isopropylamine | 19.60 | 19.42 | 19.43 | 0.18 |
| 1413 | Methylpropylether | 22.12 | 22.30 | 22.31 | -0.18 |
| 1414 | 4-Methyl-2-hexanamine | 37.69 | 37.87 | 37.92 | -0.18 |
| 1415 | Nonane | 43.88 | 43.70 | 43.70 | 0.18 |
| 1416 | Pentachloroethane | 35.57 | 35.75 | 35.78 | -0.18 |
| 1417 | Pentylcyclohexane | 50.97 | 50.79 | 50.79 | 0.18 |
| 1418 | trans-2-Butene-1,4-diol | 23.20 | 23.38 | 23.36 | -0.18 |
| 1419 | trans-Hexahydroindan | 39.58 | 39.40 | 39.40 | 0.18 |
| 1420 | Triisobutylborane | 62.31 | 62.13 | 62.08 | 0.18 |
| 1421 | Tripropylborane | 48.54 | 48.36 |  | 0.18 |
| 1422 | Methylnitrate | 14.60 | 14.78 | 14.80 | -0.18 |
| 1423 | 1,1,1-Trifluoro-2-propanol | 17.56 | 17.74 | 17.75 | -0.18 |
| 1424 | Formaldehyde diethylacetal | 28.65 | 28.47 | 28.28 | 0.18 |
| 1425 | Bis(2-hydroxyethyl)methylamine | 31.79 | 31.97 | 32.01 | -0.18 |
| 1426 | n-Decanoic acid | 50.12 | 49.94 | 49.91 | 0.18 |
| 1427 | Longifolene | 64.93 | 64.75 | 64.70 | 0.18 |
| 1428 | Perillaldehyde | 46.37 | 46.55 | 46.60 | -0.18 |
| 1429 | (2-Phenylethyl)trichlorosilane | 58.62 | 58.81 | 58.82 | -0.19 |
| 1430 | (Methylthio)ethene | 23.49 | 23.68 | 23.84 | -0.19 |
| 1431 | 1,2,4-Triisopropylbenzene | 68.86 | 68.67 | 68.67 | 0.19 |
| 1432 | 1,2-Dichloronaphthalene | 53.59 | 53.78 | 53.66 | -0.19 |
| 1433 | 1,2-Diethoxybenzene | 49.21 | 49.02 | 49.02 | 0.19 |
| 1434 | 1-Bromo-2,4-dimethylbenzene | 43.94 | 43.75 | 43.77 | 0.19 |
| 1435 | 1-Chloro-3-ethylbenzene | 40.57 | 40.76 | 40.76 | -0.19 |
| 1436 | 1-Iodohexadecane | 89.22 | 89.03 | 89.01 | 0.19 |
| 1437 | 1-Methylcyclopentene | 27.51 | 27.70 | 27.71 | -0.19 |
| 1438 | 1-Pyrrolidineethanamine | 35.28 | 35.09 | 35.15 | 0.19 |
| 1439 | 1-Vinyl-4-fluorobenzene | 36.04 | 35.85 | 35.85 | 0.19 |
| 1440 | 2,2,3-Trichlorobutyraldehyde | 35.43 | 35.62 | 35.57 | -0.19 |
| 1441 | 2,3,4-Trichloro-1-butene | 34.59 | 34.78 | 34.79 | -0.19 |
| 1442 | 2-(2-(1-Methylethoxy)ethyl)pyridine | 49.58 | 49.39 | 49.38 | 0.19 |
| 1443 | 2-Methyl-1-butanol | 26.75 | 26.56 | 26.60 | 0.19 |
| 1444 | 2-Methylenecyclohexanol | 33.62 | 33.43 | 33.47 | 0.19 |
| 1445 | 2-Octen-1-ol | 40.30 | 40.49 | 40.50 | -0.19 |
| 1446 | 3,5,5-Trimethyl-1-hexanol | 45.25 | 45.06 | 45.05 | 0.19 |
| 1447 | 3-Chloroheptane | 39.44 | 39.25 | 39.25 | 0.19 |
| 1448 | 3-Fluorobenzaldehyde | 32.28 | 32.09 | 32.04 | 0.19 |
| 1449 | 3-Hexanol | 31.38 | 31.19 | 31.25 | 0.19 |
| 1450 | 3-Methyl-1-butene | 25.18 | 24.99 | 24.97 | 0.19 |
| 1451 | 3-Phenyl-2-propyn-1-ol | 41.22 | 41.03 | 40.96 | 0.19 |
| 1452 | 4-Hexen-3-one | 30.15 | 30.34 | 30.32 | -0.19 |
| 1453 | Bis(2-ethylhexyl) sebacate | 125.97 | 125.78 | 125.78 | 0.19 |
| 1454 | 2-Chloro-1,1-diethoxyethane | 37.70 | 37.89 | 37.87 | -0.19 |
| 1455 | Cyclopentadecanone | 69.57 | 69.76 | 69.77 | -0.19 |
| 1456 | Di-n-hexylether | 59.47 | 59.28 | 59.28 | 0.19 |
| 1457 | Diallyl oxalate | 42.09 | 42.28 | 42.32 | -0.19 |
| 1458 | Diethyl 2-acetylsuccinate | 52.16 | 52.35 | 52.33 | -0.19 |
| 1459 | Dimethyl methylmalonate | 33.18 | 33.37 | 33.35 | -0.19 |
| 1460 | Dipropylene glycol monomethyl ether | 39.40 | 39.21 | 39.22 | 0.19 |
| 1461 | gamma-Coniceine | 39.63 | 39.82 | 39.36 | -0.19 |
| 1462 | Hexylcyclohexane | 55.60 | 55.41 | 55.41 | 0.19 |
| 1463 | Isopropyl acetoacetate | 36.89 | 36.70 | 36.66 | 0.19 |
| 1464 | Isopropyl methyl ether | 22.47 | 22.28 | 22.30 | 0.19 |
| 1465 | Methyl 2,3-dibromopropanoate | 38.21 | 38.02 | 38.07 | 0.19 |
| 1466 | Methyl pentyl sulfide | 37.73 | 37.92 | 38.03 | -0.19 |
| 1467 | Butyric acid methylester | 26.81 | 27.00 | 27.01 | -0.19 |
| 1468 | Methylcyclohexane | 32.50 | 32.31 | 32.31 | 0.19 |
| 1469 | Pentafluoromethoxybenzene | 32.78 | 32.97 | 33.07 | -0.19 |
| 1470 | Perchlorocyclopentene | 61.60 | 61.41 | 60.97 | 0.19 |
| 1471 | Fenchlorphos | 69.36 | 69.55 | 69.78 | -0.19 |
| 1472 | Tetramethyltetraphenylcyclotetrasiloxane | 154.31 | 154.12 | 154.20 | 0.19 |
| 1473 | Bromal | 35.75 | 35.94 |  | -0.19 |
| 1474 | Ethanol | 12.92 | 12.73 | 12.72 | 0.19 |
| 1475 | 2-Bromoisobutyronitrile | 28.11 | 28.30 | 28.33 | -0.19 |
| 1476 | Trichlorofluoroethene | 25.36 | 25.55 |  | -0.19 |
| 1477 | Ethylepoxide | 20.37 | 20.18 | 20.18 | 0.19 |
| 1478 | 2-Chloro-1,1,1-trifluoroethane | 16.39 | 16.58 | 16.61 | -0.19 |
| 1479 | 2-Ethyl-1-butene | 29.39 | 29.58 | 29.61 | -0.19 |
| 1480 | 2-Ethylthioethanol | 30.03 | 29.84 | 29.87 | 0.19 |
| 1481 | 2-Methyl-2-pentenal | 30.67 | 30.48 | 30.46 | 0.19 |
| 1482 | 2-Methyl-3-butyn-2-amine | 26.82 | 26.63 | 26.54 | 0.19 |
| 1483 | 3,3,4,4-Tetrafluorodihydro-2,5-furandione | 21.30 | 21.11 | 21.07 | 0.19 |
| 1484 | 3,3-Dimethyl-1-butanol | 31.42 | 31.23 | 31.25 | 0.19 |
| 1485 | 3-Methoxy-1,2-propanediol | 25.21 | 25.02 | 25.00 | 0.19 |
| 1486 | 4-Methyl-2-pentanol | 31.35 | 31.16 | 31.22 | 0.19 |
| 1487 | Angelic acid | 27.01 | 26.82 | 26.79 | 0.19 |
| 1488 | Cyclopropanecarboxaldehyde | 19.30 | 19.11 | 19.10 | 0.19 |
| 1489 | DMSO | 20.13 | 20.32 |  | -0.19 |
| 1490 | N-Ethyl-1-propanamine | 28.41 | 28.60 | 28.58 | -0.19 |
| 1491 | 1,1,2,2-Tetrachlorohexane | 49.27 | 49.08 | 49.05 | 0.19 |
| 1492 | 2,3,3-Trimethyl-1-pentene | 38.65 | 38.84 | 38.85 | -0.19 |
| 1493 | 2,4,4-Trimethylhexane | 43.88 | 43.69 | 43.69 | 0.19 |
| 1494 | 2,5-Dichlorotoluene | 40.61 | 40.80 | 40.81 | -0.19 |
| 1495 | 2-Methyl-1H-indene | 43.55 | 43.36 | 43.33 | 0.19 |
| 1496 | 3-Ethyloctane | 48.48 | 48.29 | 48.29 | 0.19 |
| 1497 | Adamantane | 41.65 | 41.84 | 41.84 | -0.19 |
| 1498 | Dipentyl sulfide | 56.38 | 56.19 | 56.25 | 0.19 |
| 1499 | Ethyl hexanoate | 40.69 | 40.88 | 40.89 | -0.19 |
| 1500 | Pentyl octanoate | 63.79 | 63.98 | 63.96 | -0.19 |
| 1501 | 1-Dodecylpiperidine | 82.68 | 82.49 | 82.46 | 0.19 |
| 1502 | 1,2-Dibromohexane | 45.68 | 45.48 | 45.50 | 0.20 |
| 1503 | 1,2-Diisopropylbenzene | 54.48 | 54.68 | 54.70 | -0.20 |
| 1504 | 1,2-Dimethylpiperidine | 36.17 | 36.37 | 36.37 | -0.20 |
| 1505 | Enanthic acid | 35.88 | 36.08 | 36.07 | -0.20 |
| 1506 | 2-Chloro-1-ethylbenzene | 40.56 | 40.76 | 40.81 | -0.20 |
| 1507 | 2-Methyl-1-butanol acetate | 36.43 | 36.23 | 36.21 | 0.20 |
| 1508 | 4-Morpholineethanol | 34.56 | 34.76 | 34.75 | -0.20 |
| 1509 | 4-tert-Butylpyridine | 43.15 | 42.95 | 42.96 | 0.20 |
| 1510 | 6-Dodecyne | 56.30 | 56.50 | 56.56 | -0.20 |
| 1511 | Benzyl benzoate | 62.44 | 62.64 | 62.71 | -0.20 |
| 1512 | Chlorotriethylsilane | 43.54 | 43.34 | 43.32 | 0.20 |
| 1513 | cis-3-Heptene | 34.30 | 34.50 | 34.52 | -0.20 |
| 1514 | Di-n-butylsulfide | 47.15 | 46.95 | 46.96 | 0.20 |
| 1515 | Diethyl methylmalonate | 42.45 | 42.65 | 42.67 | -0.20 |
| 1516 | Ethyl 2-acetyl-3-methylbutanoate | 45.70 | 45.90 | 45.88 | -0.20 |
| 1517 | Heptyl butanoate | 54.94 | 54.74 | 54.72 | 0.20 |
| 1518 | Heptylcyclopentane | 55.61 | 55.41 | 55.51 | 0.20 |
| 1519 | Adiponitrile | 29.34 | 29.54 | 29.54 | -0.20 |
| 1520 | 1-Bromo-2-propanol | 25.34 | 25.14 | 25.15 | 0.20 |
| 1521 | 2-Chloro-1-butene | 24.98 | 25.18 | 25.21 | -0.20 |
| 1522 | 2-Chloroethyl vinyl ether | 26.64 | 26.44 | 26.45 | 0.20 |
| 1523 | Thiolactic acid | 25.30 | 25.50 | 25.47 | -0.20 |
| 1524 | 3-Chloro-2-butanone | 25.65 | 25.85 | 25.83 | -0.20 |
| 1525 | 3-Methylbutanoyl chloride | 30.67 | 30.87 | 30.91 | -0.20 |
| 1526 | 4-Methyl-1-pentyne | 28.05 | 27.85 | 27.87 | 0.20 |
| 1527 | Propynal | 16.24 | 16.44 | 16.50 | -0.20 |
| 1528 | Azetidine | 17.44 | 17.24 | 17.27 | 0.20 |
| 1529 | n-Hexanoic acid | 31.66 | 31.46 | 31.50 | 0.20 |
| 1530 | N-Acetyl-N-methylacetamide | 29.03 | 29.23 | 29.31 | -0.20 |
| 1531 | Diethylphosphite | 31.93 | 31.73 | 31.66 | 0.20 |
| 1532 | Ethyl 2-aminoacetate | 25.62 | 25.82 | 25.78 | -0.20 |
| 1533 | 4-Methylpyridine | 28.87 | 29.07 | 29.09 | -0.20 |
| 1534 | Glycidol | 17.13 | 16.93 | 16.93 | 0.20 |
| 1535 | Propionic acid | 17.40 | 17.60 | 17.60 | -0.20 |
| 1536 | trans-1,2-Dichloropropene | 25.09 | 25.29 | 25.31 | -0.20 |
| 1537 | (4-Bromophenoxy)trimethylsilane | 58.55 | 58.35 | 58.34 | 0.20 |
| 1538 | 1,5-Dibromopentane | 40.64 | 40.84 | 40.84 | -0.20 |
| 1539 | 1,5-Diiodopentane | 51.00 | 51.20 | 51.26 | -0.20 |
| 1540 | 1-Iodooctadecane | 98.47 | 98.27 | 98.26 | 0.20 |
| 1541 | 2,2-Dimethylheptane | 43.92 | 43.72 | 43.77 | 0.20 |
| 1542 | 2,4-Dimethylsulfolane | 36.61 | 36.81 | 36.84 | -0.20 |
| 1543 | 2-Bromo-1-butene | 27.61 | 27.81 | 27.82 | -0.20 |
| 1544 | 2-Chlorotoluene | 35.94 | 36.14 | 36.14 | -0.20 |
| 1545 | 2-Ethoxy-3,4-dihydro-2H-pyran | 34.93 | 35.13 | 35.14 | -0.20 |
| 1546 | 2-Ethyl-1-pentene | 34.00 | 34.20 | 34.26 | -0.20 |
| 1547 | 2-Methyl-2-cyclohexen-1-one | 32.58 | 32.78 | 32.77 | -0.20 |
| 1548 | 5-Methyl-2-hexanol | 35.98 | 35.78 | 35.84 | 0.20 |
| 1549 | 6,7,8,9-Tetrahydro-5H-benzocyclohepten-5-one | 48.66 | 48.46 | 48.44 | 0.20 |
| 1550 | Methyl acetate | 17.56 | 17.76 | 17.77 | -0.20 |
| 1551 | Cyclohexanecarbonitrile | 31.96 | 32.16 | 32.21 | -0.20 |
| 1552 | Decylcyclopentane | 69.47 | 69.27 | 69.40 | 0.20 |
| 1553 | Diisobutyl ketone | 43.90 | 44.10 | 44.07 | -0.20 |
| 1554 | Dimethyl sulfide | 19.45 | 19.65 | 19.65 | -0.20 |
| 1555 | Ethyl diethoxyacetate | 44.32 | 44.12 | 44.15 | 0.20 |
| 1556 | Ethyl 4-chlorobutanoate | 36.25 | 36.45 | 36.45 | -0.20 |
| 1557 | Isobutyl trichloroacetate | 46.53 | 46.33 | 46.33 | 0.20 |
| 1558 | N-Phenyl-N-benzylbenzenemethanamine | 90.33 | 90.13 | 90.18 | 0.20 |
| 1559 | Octanoic anhydride | 77.97 | 77.77 | 77.88 | 0.20 |
| 1560 | Pentadecane | 71.62 | 71.42 | 71.44 | 0.20 |
| 1561 | Pentyl heptanoate | 59.56 | 59.36 | 59.36 | 0.20 |
| 1562 | 2,3-Dimethyl-2-norbornene | 39.12 | 39.32 | 39.32 | -0.20 |
| 1563 | Senecialdehyde | 26.06 | 25.86 | 25.85 | 0.20 |
| 1564 | 2-Buten-1-ol | 21.81 | 22.01 | 21.99 | -0.20 |
| 1565 | Trifluoroacetophenone | 37.17 | 36.97 | 36.94 | 0.20 |
| 1566 | Trichlorovinylsilane | 33.54 | 33.74 | 33.80 | -0.20 |
| 1567 | 2-Methoxybenzaldehyde | 38.87 | 38.66 | 38.75 | 0.21 |
| 1568 | Di-n-butylether | 41.01 | 40.80 | 40.80 | 0.21 |
| 1569 | Fenchone | 44.76 | 44.55 | 44.61 | 0.21 |
| 1570 | Hexylbenzene | 54.37 | 54.58 | 54.68 | -0.21 |
| 1571 | Isopulegol | 47.44 | 47.23 | 47.26 | 0.21 |
| 1572 | n-Butylmethylsulfide | 33.09 | 33.30 | 33.30 | -0.21 |
| 1573 | n-Octylformate | 45.87 | 45.66 | 45.65 | 0.21 |
| 1574 | Octadecanoyl chloride | 91.17 | 90.96 | 91.13 | 0.21 |
| 1575 | Tetrabutyl silicate | 88.87 | 89.08 | 89.30 | -0.21 |
| 1576 | Triisopropoxymethane | 53.51 | 53.30 | 53.22 | 0.21 |
| 1577 | 1-Chloro-3-methyl-2-butene | 30.22 | 30.01 | 30.02 | 0.21 |
| 1578 | 1-Methylcyclopentanol | 29.36 | 29.15 | 29.20 | 0.21 |
| 1579 | 1-Chloro-2-methoxyethane | 22.70 | 22.49 | 22.49 | 0.21 |
| 1580 | 2-Ethoxyethanamine | 25.95 | 25.74 | 25.73 | 0.21 |
| 1581 | 2-Propoxyethanol | 28.52 | 28.31 | 28.30 | 0.21 |
| 1582 | Chlorobenzene | 31.15 | 31.36 | 31.34 | -0.21 |
| 1583 | Di-n-propylether | 31.77 | 31.56 | 31.56 | 0.21 |
| 1584 | Propionic acid methylester | 22.17 | 22.38 | 22.37 | -0.21 |
| 1585 | Chloroacetone cyanohydrin | 26.90 | 26.69 | 26.66 | 0.21 |
| 1586 | (Dichloromethyl)trichlorosilane | 39.36 | 39.15 | 39.21 | 0.21 |
| 1587 | 1,2-Dichlorobutane | 30.41 | 30.20 | 30.20 | 0.21 |
| 1588 | 3,5-Diethyltoluene | 50.07 | 50.28 | 50.31 | -0.21 |
| 1589 | 1,3-Dioxane | 21.52 | 21.73 | 21.71 | -0.21 |
| 1590 | 3-Chlorobenzyl chloride | 40.74 | 40.95 | 40.94 | -0.21 |
| 1591 | 1-Isopropoxy-2-propanol | 33.10 | 32.89 | 32.90 | 0.21 |
| 1592 | 2,3-Dichloro-2-methylbutane | 35.10 | 34.89 | 34.88 | 0.21 |
| 1593 | 2-Chloro-4-methylpentane | 34.81 | 34.60 | 34.58 | 0.21 |
| 1594 | 2-Methyl-5-isopropylbicyclo[3.1.0]hex-2-ene | 44.23 | 44.02 | 44.01 | 0.21 |
| 1595 | 2-Methylhexanoic acid | 35.84 | 36.05 | 36.05 | -0.21 |
| 1596 | 2-Nonanone | 43.95 | 44.16 | 44.13 | -0.21 |
| 1597 | 3,3-Dimethoxy-1-propene | 28.43 | 28.22 | 28.24 | 0.21 |
| 1598 | 3-Chlorostyrene | 40.77 | 40.56 | 40.53 | 0.21 |
| 1599 | 3-Methylthiophene | 29.22 | 29.43 | 29.52 | -0.21 |
| 1600 | 4-Ethyl-4-heptanol | 44.92 | 45.13 | 45.18 | -0.21 |
| 1601 | 4-t-Butylacetophenone | 55.43 | 55.22 | 55.25 | 0.21 |
| 1602 | 4-t-Butylphenol | 46.89 | 47.10 | 47.14 | -0.21 |
| 1603 | Benzoyl bromide | 39.60 | 39.81 |  | -0.21 |
| 1604 | Bicyclo[2.2.1]hept-5-ene-2-carbonitrile | 34.40 | 34.61 | 34.62 | -0.21 |
| 1605 | Butyl octanoate | 59.15 | 59.36 | 59.34 | -0.21 |
| 1606 | Cyclobutanecarboxylic acid | 24.90 | 24.69 | 24.71 | 0.21 |
| 1607 | Cyclopentanepropanoic acid | 38.34 | 38.55 | 38.54 | -0.21 |
| 1608 | Dichloro(chloromethyl)methylsilane | 34.17 | 34.38 | 34.17 | -0.21 |
| 1609 | Diethylphosphate | 32.84 | 32.63 | 32.59 | 0.21 |
| 1610 | Dimethyl sulfate | 22.31 | 22.52 | 22.61 | -0.21 |
| 1611 | Diphenyl ether | 53.04 | 53.25 | 53.33 | -0.21 |
| 1612 | Dipropyl oxalate | 42.89 | 42.68 | 42.70 | 0.21 |
| 1613 | Ethoxyacetylchloride | 27.79 | 28.00 | 28.00 | -0.21 |
| 1614 | Ethyl 5-oxohexanoate | 41.13 | 41.34 | 41.32 | -0.21 |
| 1615 | Ethylmethylether | 17.47 | 17.68 | 17.69 | -0.21 |
| 1616 | Ethyl methyl sulfide | 23.85 | 24.06 | 24.14 | -0.21 |
| 1617 | Ethyl vinyl ether | 21.84 | 21.63 | 21.70 | 0.21 |
| 1618 | Acetone glycerol | 32.63 | 32.42 | 32.41 | 0.21 |
| 1619 | Methyl chlorooxoacetate | 23.23 | 23.44 | 23.46 | -0.21 |
| 1620 | Myrcene | 47.63 | 47.84 | 47.86 | -0.21 |
| 1621 | Butyraldehyde | 21.05 | 21.26 | 21.29 | -0.21 |
| 1622 | N-Ethylformamide | 19.85 | 19.64 | 19.66 | 0.21 |
| 1623 | n-Heptylacetate | 45.29 | 45.50 | 45.49 | -0.21 |
| 1624 | n-Undecylamine | 56.57 | 56.36 | 56.38 | 0.21 |
| 1625 | 2-Methoxyaniline | 37.04 | 37.25 | 37.37 | -0.21 |
| 1626 | Octyl butanoate | 59.57 | 59.36 | 59.37 | 0.21 |
| 1627 | p-Menthane | 46.32 | 46.11 | 46.11 | 0.21 |
| 1628 | s-Butyl pentanoate | 45.27 | 45.48 | 45.49 | -0.21 |
| 1629 | 1-Chloro-2-propanol | 22.35 | 22.14 | 22.13 | 0.21 |
| 1630 | t-Butylethylether | 31.83 | 31.62 | 31.61 | 0.21 |
| 1631 | Tetrahydro-2,5-dimethoxyfuran | 32.65 | 32.44 | 32.48 | 0.21 |
| 1632 | Tris(p-tolyl) phosphite | 102.54 | 102.33 | 102.42 | 0.21 |
| 1633 | 1,1,3,3-Tetramethoxypropane | 40.64 | 40.86 | 40.90 | -0.22 |
| 1634 | 1,2,4-Trichlorobenzene | 40.90 | 40.68 | 40.71 | 0.22 |
| 1635 | 1,2-Dimethylenecyclohexane | 36.22 | 36.44 | 36.46 | -0.22 |
| 1636 | 1,2-Diphenylbutane | 69.83 | 70.05 | 70.03 | -0.22 |
| 1637 | 1,7-Dibromoheptane | 49.86 | 50.08 | 50.11 | -0.22 |
| 1638 | 1-Chlorodecane | 53.35 | 53.13 | 53.13 | 0.22 |
| 1639 | 1-Eicosene | 94.54 | 94.32 | 94.32 | 0.22 |
| 1640 | 1-Heptadecylamine | 84.30 | 84.08 | 84.10 | 0.22 |
| 1641 | 1-Methylcyclohexanol | 33.99 | 33.77 | 33.72 | 0.22 |
| 1642 | 2,4-Dimethyl-2-pentanol | 36.08 | 35.86 | 35.91 | 0.22 |
| 1643 | 2-(Chloromethyl)furan | 28.80 | 29.02 | 29.12 | -0.22 |
| 1644 | 1-Chloro-2-(trichloromethyl)benzene | 50.64 | 50.86 | 50.86 | -0.22 |
| 1645 | 2-Chloroheptane | 39.47 | 39.25 | 39.24 | 0.22 |
| 1646 | 2-Methyl-4-heptanol | 40.18 | 40.40 | 40.40 | -0.22 |
| 1647 | 2-Methylpiperidine | 31.34 | 31.12 | 31.10 | 0.22 |
| 1648 | 1-Ethoxy-2-nitrobenzene | 44.23 | 44.45 | 44.52 | -0.22 |
| 1649 | 3-(Chloromethyl)pentane | 34.40 | 34.62 | 34.64 | -0.22 |
| 1650 | 3-Ethyltoluene | 40.66 | 40.88 | 40.86 | -0.22 |
| 1651 | 3-Methylhexane | 34.21 | 34.43 | 34.43 | -0.22 |
| 1652 | 4-tert-Butyltoluene | 49.92 | 50.14 | 50.11 | -0.22 |
| 1653 | 4-Methylanisole | 37.78 | 38.00 | 37.99 | -0.22 |
| 1654 | 4-Propylheptane | 48.51 | 48.29 | 48.29 | 0.22 |
| 1655 | Acetone cyanohydrin | 21.66 | 21.88 | 21.93 | -0.22 |
| 1656 | cis-1,2-Dimethylcyclopentane | 32.50 | 32.28 | 32.28 | 0.22 |
| 1657 | cis-1-Methyl-2-propylcyclopentane | 41.74 | 41.52 | 41.52 | 0.22 |
| 1658 | Cyclononane | 41.36 | 41.58 | 41.58 | -0.22 |
| 1659 | Cyclopentanemethanol | 29.28 | 29.06 | 29.04 | 0.22 |
| 1660 | Cyclopentylbenzene | 47.59 | 47.81 | 47.82 | -0.22 |
| 1661 | Methylelaidate | 91.50 | 91.72 | 91.71 | -0.22 |
| 1662 | Heptylcyclohexane | 60.25 | 60.03 | 60.03 | 0.22 |
| 1663 | Hexadecanenitrile | 76.11 | 75.89 | 76.03 | 0.22 |
| 1664 | Isobutyl 2-chloropropanoate | 40.80 | 41.02 | 41.02 | -0.22 |
| 1665 | Isopentyl trichloroacetate | 51.17 | 50.95 | 50.95 | 0.22 |
| 1666 | 1-Methyl-4-isopropylcyclohexene | 45.90 | 46.12 | 46.16 | -0.22 |
| 1667 | N-Butylpiperidine | 45.75 | 45.53 | 45.53 | 0.22 |
| 1668 | Butyl pentanoate | 45.28 | 45.50 | 45.49 | -0.22 |
| 1669 | n-Dodecylamine | 61.20 | 60.98 | 61.01 | 0.22 |
| 1670 | n-Propylformate | 22.34 | 22.56 | 22.57 | -0.22 |
| 1671 | Ethyl nicotinate | 40.33 | 40.11 | 40.13 | 0.22 |
| 1672 | Oxepane | 29.66 | 29.44 | 29.45 | 0.22 |
| 1673 | Propofol | 56.20 | 56.42 | 56.41 | -0.22 |
| 1674 | Isobutylcyclohexane | 46.36 | 46.14 | 46.14 | 0.22 |
| 1675 | trans-Crotononitrile | 20.71 | 20.49 | 20.51 | 0.22 |
| 1676 | trans-2-Heptene | 34.28 | 34.50 | 34.50 | -0.22 |
| 1677 | Tributylphosphine | 68.48 | 68.26 |  | 0.22 |
| 1678 | Tridecane | 62.40 | 62.18 | 62.18 | 0.22 |
| 1679 | Bromoethylene | 18.80 | 18.58 | 18.56 | 0.22 |
| 1680 | Pyridine | 24.07 | 24.29 | 24.31 | -0.22 |
| 1681 | 1,2-Dichloro-1,1-difluoroethane | 21.13 | 21.35 | 21.36 | -0.22 |
| 1682 | 1,5-Hexadiene | 29.22 | 29.44 | 29.48 | -0.22 |
| 1683 | 2-Methyl-1,3-dioxolane | 21.94 | 21.72 | 21.70 | 0.22 |
| 1684 | 3-Methylcyclopentanone | 27.93 | 28.15 | 28.15 | -0.22 |
| 1685 | 4-Methyloxazole | 21.22 | 21.44 |  | -0.22 |
| 1686 | Trimethoxymethane | 25.36 | 25.58 |  | -0.22 |
| 1687 | 1,1-Diethoxypentane | 47.16 | 46.94 | 46.93 | 0.22 |
| 1688 | 1-Phenylpiperazine | 51.37 | 51.15 | 51.20 | 0.22 |
| 1689 | 2,2,3,4-Tetramethylpentane | 43.44 | 43.66 | 43.66 | -0.22 |
| 1690 | 4-Isopropylheptane | 48.48 | 48.26 | 48.28 | 0.22 |
| 1691 | Benzo[b]furan | 35.08 | 35.30 | 35.38 | -0.22 |
| 1692 | Diethoxymethylsilane | 36.84 | 36.62 | 36.59 | 0.22 |
| 1693 | Dipropyl adipate | 60.94 | 61.16 | 61.16 | -0.22 |
| 1694 | Di-n-butyl phthalate | 77.05 | 76.82 | 76.88 | 0.23 |
| 1695 | Dihexylsulfate | 68.99 | 68.76 | 68.72 | 0.23 |
| 1696 | Hexaethylbenzene | 83.33 | 83.10 | 83.16 | 0.23 |
| 1697 | (3-Chloropropyl)trimethylsilane | 44.47 | 44.24 | 44.38 | 0.23 |
| 1698 | 1,4-Piperazinedipropanamine | 60.71 | 60.94 | 60.96 | -0.23 |
| 1699 | 4-Chlorodiphenyl ether | 58.14 | 57.91 | 57.89 | 0.23 |
| 1700 | 1-Octanol | 40.68 | 40.45 | 40.45 | 0.23 |
| 1701 | 2,2,3,3-Tetramethylhexane | 48.13 | 48.36 | 48.38 | -0.23 |
| 1702 | 2,5-Dimethylacetophenone | 45.89 | 46.12 | 46.15 | -0.23 |
| 1703 | 3-Ethyl-3-pentanol | 35.66 | 35.89 | 35.91 | -0.23 |
| 1704 | 5-Methyl-3-heptanol | 40.17 | 40.40 | 40.41 | -0.23 |
| 1705 | Dihydro-5-methyl-2(3H)-furanone | 24.65 | 24.88 | 24.87 | -0.23 |
| 1706 | L-Menthone | 46.34 | 46.57 | 46.57 | -0.23 |
| 1707 | n-Heptaldehyde | 34.89 | 35.12 | 35.14 | -0.23 |
| 1708 | n-Hexylmercaptan | 37.69 | 37.46 | 37.46 | 0.23 |
| 1709 | N-Nitrosodiethylamine | 28.49 | 28.72 | 28.76 | -0.23 |
| 1710 | Octodrine | 42.72 | 42.49 | 42.54 | 0.23 |
| 1711 | Octylcyclopentane | 60.26 | 60.03 | 60.14 | 0.23 |
| 1712 | Amylbenzene | 49.73 | 49.96 | 50.05 | -0.23 |
| 1713 | Propyltrichlorosilane | 38.46 | 38.69 | 38.72 | -0.23 |
| 1714 | trans-2-Octene | 38.89 | 39.12 | 39.12 | -0.23 |
| 1715 | 2-Bromoethanesulfonylchloride | 33.06 | 33.29 | 33.36 | -0.23 |
| 1716 | Isobutyroylbromide | 29.14 | 28.91 | 28.90 | 0.23 |
| 1717 | (Chloromethoxy)ethane | 22.69 | 22.46 | 22.40 | 0.23 |
| 1718 | 1,1,1-Trichlorotrifluoroethane | 26.26 | 26.49 | 26.55 | -0.23 |
| 1719 | 1-Pentanol | 26.82 | 26.59 | 26.62 | 0.23 |
| 1720 | 2-Butanol | 22.18 | 21.95 | 21.97 | 0.23 |
| 1721 | 2-Butylacetate | 31.39 | 31.62 | 31.60 | -0.23 |
| 1722 | 2-Chloropropane | 21.00 | 20.77 | 20.78 | 0.23 |
| 1723 | alpha-Chlorohydrin | 23.74 | 23.51 | 23.52 | 0.23 |
| 1724 | 3-Chloro-2,5-furandione | 25.26 | 25.03 | 25.06 | 0.23 |
| 1725 | 4-Methyl-1,3-dioxolane | 21.48 | 21.71 | 21.75 | -0.23 |
| 1726 | Butoxyacetylene | 29.44 | 29.21 | 29.17 | 0.23 |
| 1727 | Chloroacetic acid | 17.56 | 17.79 | 17.81 | -0.23 |
| 1728 | Cyclobutane | 18.25 | 18.48 | 18.48 | -0.23 |
| 1729 | Dihydro-4-methyl-2(3H)-furanone | 24.64 | 24.87 | 24.86 | -0.23 |
| 1730 | Isopropyl isocyanate | 22.90 | 22.67 | 22.67 | 0.23 |
| 1731 | Methoxyacetyl chloride | 23.13 | 23.36 | 23.32 | -0.23 |
| 1732 | Methyl-n-propylsulfide | 28.45 | 28.68 | 28.75 | -0.23 |
| 1733 | n-Butylformate | 26.95 | 27.18 | 27.19 | -0.23 |
| 1734 | N-Methylmorpholine | 29.06 | 28.83 | 28.83 | 0.23 |
| 1735 | Succinic acid | 24.07 | 23.84 | 23.84 | 0.23 |
| 1736 | trans-4-Hexen-1-ol | 31.02 | 31.25 | 31.22 | -0.23 |
| 1737 | 1,3-Diphenyl-1-butene | 70.32 | 70.09 | 70.08 | 0.23 |
| 1738 | Ethyl 1-bromoacrylate | 34.00 | 34.23 | 34.26 | -0.23 |
| 1739 | 1-Chloro-3-fluoro-2-methylbenzene | 35.86 | 36.09 | 36.15 | -0.23 |
| 1740 | 1-Hexyl-1,2,3,4-tetrahydronaphthalene | 70.84 | 71.07 | 71.09 | -0.23 |
| 1741 | 2,4-Dimethylpentane | 34.63 | 34.40 | 34.40 | 0.23 |
| 1742 | 2-Iodo-1,4-dimethylbenzene | 49.05 | 48.82 | 48.85 | 0.23 |
| 1743 | 4-Octanol | 40.66 | 40.43 | 40.43 | 0.23 |
| 1744 | 5-Methyl-2,3-hexanedione | 35.12 | 35.35 | 35.35 | -0.23 |
| 1745 | 7-Hydroxy-3,7-dimethyloctanal | 50.15 | 50.38 | 50.36 | -0.23 |
| 1746 | Arecoline | 42.50 | 42.73 | 42.70 | -0.23 |
| 1747 | Benzyl isocyanide | 36.60 | 36.37 |  | 0.23 |
| 1748 | Butylethylamine | 33.45 | 33.22 | 33.21 | 0.23 |
| 1749 | cis-1,2-Divinylcyclobutane | 36.73 | 36.50 | 36.50 | 0.23 |
| 1750 | Cyclodecane | 45.97 | 46.20 | 46.20 | -0.23 |
| 1751 | n-Amyl ether | 50.27 | 50.04 | 50.04 | 0.23 |
| 1752 | Diethyl isobutylmalonate | 56.25 | 56.48 | 56.44 | -0.23 |
| 1753 | Dihexylamine | 61.17 | 60.94 | 61.06 | 0.23 |
| 1754 | Ethyl 10-undecenoate | 64.01 | 63.78 | 63.76 | 0.23 |
| 1755 | Ethyl 3-hydroxybutanoate | 32.76 | 32.99 | 33.05 | -0.23 |
| 1756 | Jasmone | 51.01 | 51.24 | 51.25 | -0.23 |
| 1757 | N-Methylbenzeneethanamine | 43.93 | 44.16 | 44.16 | -0.23 |
| 1758 | Pelargonic acid | 45.55 | 45.32 | 45.32 | 0.23 |
| 1759 | Phenyl propanoate | 42.18 | 42.41 | 42.43 | -0.23 |
| 1760 | trans-1,2-Dimethylcyclohexane | 37.13 | 36.90 | 36.94 | 0.23 |
| 1761 | 1-Chloro-4-tert-butylbenzene | 50.26 | 50.02 | 50.07 | 0.24 |
| 1762 | 10-Undecen-1-ol | 53.87 | 54.11 | 54.12 | -0.24 |
| 1763 | 2,6-Di-t-butyl-4-methylphenol | 70.78 | 70.54 | 70.56 | 0.24 |
| 1764 | 3-Ethyl-3-methylhexane | 43.48 | 43.72 | 43.71 | -0.24 |
| 1765 | 4-(1-Butylpentyl)pyridine | 66.24 | 66.00 | 66.01 | 0.24 |
| 1766 | 4-Methoxybenzenethiol | 45.38 | 45.62 | 45.63 | -0.24 |
| 1767 | Diethyl azelate | 65.54 | 65.78 | 65.74 | -0.24 |
| 1768 | Bromobenzene | 33.95 | 34.19 | 34.25 | -0.24 |
| 1769 | n-Octanoic acid | 40.94 | 40.70 | 40.70 | 0.24 |
| 1770 | Ethyleneglycol diacetate | 33.20 | 33.44 | 33.42 | -0.24 |
| 1771 | Heptadecanenitrile | 80.75 | 80.51 | 80.51 | 0.24 |
| 1772 | Isopropyl 3-methylbutanoate | 40.59 | 40.83 | 40.82 | -0.24 |
| 1773 | Nonylcyclopentane | 64.89 | 64.65 | 64.65 | 0.24 |
| 1774 | Octylcyclohexane | 64.89 | 64.65 | 64.65 | 0.24 |
| 1775 | s-Butylethylamine | 33.48 | 33.24 | 33.22 | 0.24 |
| 1776 | 1,5-Pentanediol | 28.20 | 27.96 | 27.92 | 0.24 |
| 1777 | 3-Methylpyridine | 28.83 | 29.07 | 29.08 | -0.24 |
| 1778 | cis-3-Hexen-1-ol | 31.01 | 31.25 | 31.26 | -0.24 |
| 1779 | Dibromoacetonitrile | 26.31 | 26.55 | 26.56 | -0.24 |
| 1780 | Ethyl isocyanate | 18.27 | 18.03 | 18.24 | 0.24 |
| 1781 | 2-Butanone | 20.82 | 21.06 | 21.09 | -0.24 |
| 1782 | Glyceryl-1-nitrate | 27.00 | 26.76 | 26.71 | 0.24 |
| 1783 | Methyl cyclobutanecarboxylate | 29.71 | 29.47 | 29.46 | 0.24 |
| 1784 | 1,4-Dioxane | 21.68 | 21.92 | 21.92 | -0.24 |
| 1785 | (3-Chloropropyl)trichlorosilane | 43.26 | 43.50 | 43.54 | -0.24 |
| 1786 | 1,2-Dipiperidinoethane | 61.46 | 61.22 | 61.16 | 0.24 |
| 1787 | 1,3-Propanediol diacetate | 37.82 | 38.06 | 38.02 | -0.24 |
| 1788 | 1,4-Dimethylpiperazine | 35.50 | 35.74 | 35.64 | -0.24 |
| 1789 | 1,5-Dimethylcyclopentene | 32.05 | 32.29 | 32.29 | -0.24 |
| 1790 | 1,6-Diiodohexane | 55.58 | 55.82 | 55.80 | -0.24 |
| 1791 | 1-Bromo-2-chloro-1,1,2-trifluoroethane | 24.26 | 24.02 |  | 0.24 |
| 1792 | 1-Iodo-2-triazobenzene | 48.05 | 47.81 |  | 0.24 |
| 1793 | 1-Nonanol | 45.31 | 45.07 | 45.08 | 0.24 |
| 1794 | 1-Propylnaphthalene | 58.24 | 58.48 | 58.51 | -0.24 |
| 1795 | 10,11-Dihydro-5H-dibenzo[a,d]cyclohepten-5-one | 63.88 | 64.12 | 64.05 | -0.24 |
| 1796 | 2,2,4-Trimethylcyclopentanone | 37.17 | 37.41 | 37.44 | -0.24 |
| 1797 | 2,4,4-Trimethyl-2-pentanol | 40.77 | 40.53 | 40.51 | 0.24 |
| 1798 | 2,6-Lutidine | 33.89 | 34.13 | 34.02 | -0.24 |
| 1799 | 2-(2-Methoxyethoxy)ethanol | 29.77 | 30.01 | 30.02 | -0.24 |
| 1800 | 2-(3-Pentenyl)pyridine | 47.49 | 47.73 | 47.72 | -0.24 |
| 1801 | 2-Pyridinemethanamine | 32.39 | 32.63 | 32.64 | -0.24 |
| 1802 | 2-Ethyl-6-methylaniline | 44.67 | 44.91 | 44.92 | -0.24 |
| 1803 | 2-Hydroxyethylmethacrylate | 32.51 | 32.75 | 32.71 | -0.24 |
| 1804 | 2-Methyl-1-nonene | 48.30 | 48.06 | 48.06 | 0.24 |
| 1805 | 3,5-Dimethyl-2-cyclohexen-1-one | 37.61 | 37.37 | 37.38 | 0.24 |
| 1806 | Propanolamine | 21.01 | 20.77 | 20.76 | 0.24 |
| 1807 | Isovaleraldehyde | 25.61 | 25.85 | 25.82 | -0.24 |
| 1808 | 3-Methylstyrene | 40.92 | 40.68 | 40.68 | 0.24 |
| 1809 | 4-Chlorobutanenitrile | 25.02 | 25.26 | 25.27 | -0.24 |
| 1810 | 4-Ethyloctane | 48.53 | 48.29 | 48.29 | 0.24 |
| 1811 | Acetylisothiocyanate | 26.81 | 26.57 | 26.38 | 0.24 |
| 1812 | Butyl caproate | 49.88 | 50.12 | 50.13 | -0.24 |
| 1813 | Butylbenzene | 45.10 | 45.34 | 45.42 | -0.24 |
| 1814 | Caproyl chloride | 35.28 | 35.52 | 35.55 | -0.24 |
| 1815 | Cyclohexanemethanol | 33.92 | 33.68 | 33.66 | 0.24 |
| 1816 | Cyclooctane | 36.72 | 36.96 | 36.96 | -0.24 |
| 1817 | Dicyclopentadiene | 42.16 | 41.92 | 41.92 | 0.24 |
| 1818 | Dimethyl sebacate | 60.88 | 61.12 | 61.10 | -0.24 |
| 1819 | Ethyl 4-methylpentanoate | 40.61 | 40.85 | 40.86 | -0.24 |
| 1820 | Ethylidene diacetate | 33.00 | 33.24 | 33.22 | -0.24 |
| 1821 | Ethyltrichlorosilane | 33.83 | 34.07 | 34.09 | -0.24 |
| 1822 | Hexachloropropene | 44.83 | 45.07 | 45.18 | -0.24 |
| 1823 | Methyl 2-methoxybenzoate | 44.64 | 44.40 | 44.43 | 0.24 |
| 1824 | N,N-Diethylpropanamide | 38.14 | 38.38 | 38.34 | -0.24 |
| 1825 | 1-Butanethiol | 28.46 | 28.22 | 28.21 | 0.24 |
| 1826 | N-Nitrosodipropylamine | 37.72 | 37.96 | 37.93 | -0.24 |
| 1827 | Phenyltrimethylsilane | 49.88 | 50.12 | 50.12 | -0.24 |
| 1828 | trans-Octahydro-1(2H)-naphthalenone | 44.24 | 44.48 | 44.47 | -0.24 |
| 1829 | Widdrene | 64.83 | 65.07 | 65.15 | -0.24 |
| 1830 | Tridecylamine | 65.84 | 65.60 | 65.59 | 0.24 |
| 1831 | 1,2-Dichloro-1,3-butadiene | 29.96 | 29.71 | 29.60 | 0.25 |
| 1832 | Neopentylidenebromide | 40.83 | 40.58 | 40.50 | 0.25 |
| 1833 | 1,2-Diethoxyethane | 33.53 | 33.28 | 33.28 | 0.25 |
| 1834 | 1,2-Dipropoxyethane | 42.77 | 42.52 | 42.50 | 0.25 |
| 1835 | 1,4-Dibromo-2-butyne | 35.41 | 35.16 | 35.16 | 0.25 |
| 1836 | 1-Acetonaphthone | 54.07 | 54.32 | 54.34 | -0.25 |
| 1837 | 1-Chlorononane | 48.26 | 48.51 | 48.52 | -0.25 |
| 1838 | 1-Ethylcyclohexene | 36.69 | 36.94 | 36.94 | -0.25 |
| 1839 | Heptanophenone | 59.41 | 59.66 | 59.63 | -0.25 |
| 1840 | 1-Propoxy-2-propanol | 33.16 | 32.91 | 32.91 | 0.25 |
| 1841 | 3-t-Butyltoluene | 49.89 | 50.14 | 50.19 | -0.25 |
| 1842 | 2,2,5-Trimethylhexane | 43.94 | 43.69 | 43.70 | 0.25 |
| 1843 | 2,3-Dibromo-2-methylbutane | 41.15 | 40.90 | 40.88 | 0.25 |
| 1844 | 2,3-Dichloro-1,3-butadiene | 30.01 | 29.76 | 29.76 | 0.25 |
| 1845 | 2-(Allyloxy)ethanol | 27.86 | 28.11 | 28.10 | -0.25 |
| 1846 | 2-Bromo-1,4-dimethylbenzene | 43.50 | 43.75 | 43.80 | -0.25 |
| 1847 | 2-Chloro-6-phenylphenol | 58.25 | 58.00 | 58.03 | 0.25 |
| 1848 | 2-Chlorooctane | 44.12 | 43.87 | 43.85 | 0.25 |
| 1849 | 2-Chloropropanoic acid | 22.64 | 22.39 | 22.38 | 0.25 |
| 1850 | Phlorol | 37.59 | 37.84 | 37.85 | -0.25 |
| 1851 | 2-Iodoacetophenone | 49.37 | 49.12 | 49.20 | 0.25 |
| 1852 | 2-Methyl-2-butene | 24.95 | 25.20 | 25.20 | -0.25 |
| 1853 | 3-Chloro-(1,1'-biphenyl)-2-ol | 58.25 | 58.00 | 58.03 | 0.25 |
| 1854 | 3-Chlorohexane | 34.88 | 34.63 | 34.61 | 0.25 |
| 1855 | 3-Ethylheptane | 43.92 | 43.67 | 43.69 | 0.25 |
| 1856 | 3-Hexyne-2,5-diol | 31.23 | 31.48 | 31.50 | -0.25 |
| 1857 | 3-Hydroxyacetophenone | 38.55 | 38.30 | 38.30 | 0.25 |
| 1858 | 3-Iodopentane | 38.21 | 38.46 | 38.52 | -0.25 |
| 1859 | 4-Chloro-1-phenyl-1-butanone | 50.86 | 50.61 | 50.61 | 0.25 |
| 1860 | 4-Propylphenol | 42.21 | 42.46 | 42.46 | -0.25 |
| 1861 | Allyl chloroformate | 26.96 | 27.21 | 27.23 | -0.25 |
| 1862 | Benzyl chloroacetate | 47.58 | 47.33 | 47.36 | 0.25 |
| 1863 | (Chloromethyl)benzene | 36.04 | 36.29 | 36.30 | -0.25 |
| 1864 | Bis(3-aminopropyl)amine | 39.81 | 40.06 | 40.01 | -0.25 |
| 1865 | Carvacrol | 46.96 | 47.21 | 47.24 | -0.25 |
| 1866 | Chlorotrimethylsilane | 29.88 | 29.63 | 29.61 | 0.25 |
| 1867 | Menthone | 46.32 | 46.57 | 46.57 | -0.25 |
| 1868 | 2-Propylpiperidine | 40.61 | 40.36 | 40.32 | 0.25 |
| 1869 | Crotonaldehyde | 21.55 | 21.30 | 21.33 | 0.25 |
| 1870 | Cumic alcohol | 46.59 | 46.84 | 46.84 | -0.25 |
| 1871 | Cyclohexylisocyanate | 34.66 | 34.41 | 34.48 | 0.25 |
| 1872 | d-Verbenone | 44.23 | 44.48 | 44.46 | -0.25 |
| 1873 | Dichloroacetonitrile | 21.13 | 20.88 | 20.89 | 0.25 |
| 1874 | Diethyleneglycol diethyl ether | 44.49 | 44.24 | 44.24 | 0.25 |
| 1875 | Dimethyl malonate | 28.53 | 28.78 | 28.78 | -0.25 |
| 1876 | Dinonyl ether | 87.25 | 87.00 | 87.15 | 0.25 |
| 1877 | Ethyl chloroformate | 23.04 | 22.79 | 22.75 | 0.25 |
| 1878 | Heptanedinitrile | 34.41 | 34.16 | 34.16 | 0.25 |
| 1879 | Hexadecamethylheptasiloxane | 142.31 | 142.06 | 142.07 | 0.25 |
| 1880 | 2-Methyl-1-propanol | 22.19 | 21.94 | 21.98 | 0.25 |
| 1881 | Isobutyl chlorocarbonate | 32.25 | 32.00 | 32.02 | 0.25 |
| 1882 | Isobutyl isothiocyanate | 35.21 | 35.46 | 35.53 | -0.25 |
| 1883 | Methyl pentafluoropropanoate | 22.99 | 23.24 | 23.25 | -0.25 |
| 1884 | Methylacetoacetate | 27.21 | 27.46 | 27.46 | -0.25 |
| 1885 | N-Acetylethanolamine | 25.85 | 25.60 | 25.61 | 0.25 |
| 1886 | n-Hexylacetate | 40.63 | 40.88 | 40.89 | -0.25 |
| 1887 | 1-Methyl-1H-pyrazole | 23.44 | 23.69 | 23.76 | -0.25 |
| 1888 | N-Phenylethanolamine | 41.48 | 41.73 | 41.77 | -0.25 |
| 1889 | Nitroglycerin | 40.39 | 40.14 | 39.96 | 0.25 |
| 1890 | Nonylcyclohexane | 69.52 | 69.27 | 69.28 | 0.25 |
| 1891 | Pentafluoroiodobenzene | 38.76 | 39.01 | 39.11 | -0.25 |
| 1892 | Phenylglycidyl ether | 41.81 | 42.06 | 42.06 | -0.25 |
| 1893 | Rhodinol | 49.65 | 49.40 | 49.45 | 0.25 |
| 1894 | t-Amyl methyl ether | 31.35 | 31.60 | 31.64 | -0.25 |
| 1895 | t-Butylacetate | 31.45 | 31.70 | 31.75 | -0.25 |
| 1896 | Tetraethylurea | 50.12 | 50.37 |  | -0.25 |
| 1897 | trans-(1-Methyl-1-propenyl)benzene | 45.57 | 45.32 | 45.30 | 0.25 |
| 1898 | Tris(2-chloroethyl) phosphite | 57.64 | 57.39 | 57.35 | 0.25 |
| 1899 | 3-Butenoic acid | 21.77 | 22.02 | 22.01 | -0.25 |
| 1900 | Ethyl but-3-yne- 1-carboxylate | 34.05 | 34.30 | 34.36 | -0.25 |
| 1901 | Entprol | 80.36 | 80.10 | 80.04 | 0.26 |
| 1902 | Hexaethyl tetraphosphate | 100.69 | 100.43 | 100.42 | 0.26 |
| 1903 | Hexaethyltetraphosphate | 100.69 | 100.43 | 100.31 | 0.26 |
| 1904 | 1,3-Dioxolane | 16.85 | 17.11 | 17.11 | -0.26 |
| 1905 | Propane-1,3-dithiol | 31.48 | 31.22 | 31.21 | 0.26 |
| 1906 | 1,8-Nonadiyne | 39.52 | 39.78 | 39.80 | -0.26 |
| 1907 | 1-Propanesulfonylchloride | 30.36 | 30.10 | 30.02 | 0.26 |
| 1908 | 1-Propanol | 17.61 | 17.35 | 17.34 | 0.26 |
| 1909 | 1-Propylcyclopentanol | 38.13 | 38.39 | 38.41 | -0.26 |
| 1910 | 10-Undecenoyl chloride | 58.16 | 58.42 | 58.45 | -0.26 |
| 1911 | 2-Mercaptoethanol | 20.61 | 20.35 | 20.43 | 0.26 |
| 1912 | 2-Acetoxyanisole | 44.05 | 44.31 | 44.38 | -0.26 |
| 1913 | 2-Methyldecane | 53.17 | 52.91 | 52.93 | 0.26 |
| 1914 | o-Cresol | 32.96 | 33.22 | 33.19 | -0.26 |
| 1915 | 2-Nitroanisol | 39.55 | 39.81 | 39.80 | -0.26 |
| 1916 | 3,3-Dimethylcyclohexanone | 37.18 | 37.44 | 37.43 | -0.26 |
| 1917 | 3-Bromotoluene | 38.71 | 38.97 | 39.02 | -0.26 |
| 1918 | 3-Chlorobenzotrichloride | 50.60 | 50.86 | 50.90 | -0.26 |
| 1919 | 3-Chlorotoluene | 35.88 | 36.14 | 36.13 | -0.26 |
| 1920 | 3-Trifluoromethylbenzoyl chloride | 42.61 | 42.35 | 42.33 | 0.26 |
| 1921 | Acetylchloride | 16.78 | 17.04 | 17.07 | -0.26 |
| 1922 | 1-Phenyl-1-butanol | 46.43 | 46.69 | 46.69 | -0.26 |
| 1923 | cis-2-Penten-1-ol | 26.37 | 26.63 | 26.65 | -0.26 |
| 1924 | Diglyme | 35.22 | 34.96 | 35.02 | 0.26 |
| 1925 | Dimethyl methylenesuccinate | 37.50 | 37.76 | 37.76 | -0.26 |
| 1926 | Heptanoyl chloride | 40.40 | 40.14 | 40.12 | 0.26 |
| 1927 | Isobutylnitrite | 26.91 | 26.65 | 26.52 | 0.26 |
| 1928 | Methyl methanesulfonate | 21.25 | 20.99 | 20.95 | 0.26 |
| 1929 | Neomenthol | 47.72 | 47.46 | 47.46 | 0.26 |
| 1930 | 1,4-Dimethylbenzene | 36.00 | 36.26 | 36.26 | -0.26 |
| 1931 | trans-2-Hexen-1-ol, acetate | 40.66 | 40.92 | 40.93 | -0.26 |
| 1932 | trans-2-Methylcyclohexanol | 33.92 | 33.66 | 33.66 | 0.26 |
| 1933 | n-Propylbromoacetate | 34.57 | 34.83 | 34.83 | -0.26 |
| 1934 | Methylhydrazine | 13.69 | 13.43 | 12.44 | 0.26 |
| 1935 | Propargyl aldehyde | 14.42 | 14.68 | 14.79 | -0.26 |
| 1936 | 1,2,4-Trifluorobenzene | 26.29 | 26.55 | 26.52 | -0.26 |
| 1937 | 2,3-Dichloropropene | 25.11 | 25.37 | 25.36 | -0.26 |
| 1938 | 2-Amino-2-methyl-1-propanol | 25.60 | 25.34 | 25.39 | 0.26 |
| 1939 | 2-Iodopropane | 29.48 | 29.22 | 29.17 | 0.26 |
| 1940 | 2-Isopropoxyethanol | 28.55 | 28.29 | 28.27 | 0.26 |
| 1941 | 3-Hydroxypropanenitrile | 17.46 | 17.20 | 17.26 | 0.26 |
| 1942 | 3-Methyl-2-buten-1-ol | 26.83 | 26.57 | 26.57 | 0.26 |
| 1943 | 4-Chloro-1,2-butadiene | 25.32 | 25.58 | 25.42 | -0.26 |
| 1944 | Ethylisocyanide | 16.51 | 16.25 |  | 0.26 |
| 1945 | Perfluorotoluene | 31.90 | 31.64 | 31.63 | 0.26 |
| 1946 | Piperidine | 26.74 | 26.48 | 26.49 | 0.26 |
| 1947 | Propargyl acetate | 24.80 | 25.06 | 25.03 | -0.26 |
| 1948 | trans-1,3-Dichloropropene | 25.58 | 25.32 | 25.31 | 0.26 |
| 1949 | 1,1,1,3-Tetrachloropropane | 35.77 | 35.51 | 35.49 | 0.26 |
| 1950 | 1,3,5-Triisopropylbenzene | 68.93 | 68.67 | 68.64 | 0.26 |
| 1951 | 1-Bromo-2-ethylbenzene | 43.33 | 43.59 | 43.65 | -0.26 |
| 1952 | 1-Chloronaphthalene | 48.86 | 49.12 | 49.03 | -0.26 |
| 1953 | 1-Tetradecylamine | 70.48 | 70.22 | 70.20 | 0.26 |
| 1954 | 6-Methyl-5-hepten-2-one | 39.26 | 39.52 | 39.54 | -0.26 |
| 1955 | 2,3-Dihydrobenzofuran | 36.16 | 35.90 | 35.89 | 0.26 |
| 1956 | Diethyl ethylmalonate | 47.01 | 47.27 | 47.26 | -0.26 |
| 1957 | Dimethoxydiphenylsilane | 71.70 | 71.44 | 71.29 | 0.26 |
| 1958 | Homocamfin | 46.84 | 46.58 | 46.57 | 0.26 |
| 1959 | 2-Phenoxyethanol | 38.97 | 39.23 | 39.24 | -0.26 |
| 1960 | trans-1-Ethyl-3-methylcyclopentane | 37.16 | 36.90 | 36.90 | 0.26 |
| 1961 | 1-Methylanthracene | 66.73 | 67.00 | 67.07 | -0.27 |
| 1962 | 2,3,3-Trimethyl-1-butene | 33.95 | 34.22 | 34.26 | -0.27 |
| 1963 | 2,3-Dimethyl-3-ethylpentane | 43.42 | 43.69 | 43.69 | -0.27 |
| 1964 | 2,4,6-Trimethyl-2,4,6-triphenylcyclotrisiloxane | 115.86 | 115.59 | 115.47 | 0.27 |
| 1965 | 2,4-Difluoronitrobenzene | 32.92 | 33.19 | 33.20 | -0.27 |
| 1966 | 2-Bromooctane | 47.18 | 46.91 | 46.87 | 0.27 |
| 1967 | 2-Heptanol | 36.08 | 35.81 | 35.81 | 0.27 |
| 1968 | 3,3'-Dimethylazobenzene | 72.51 | 72.78 | 73.30 | -0.27 |
| 1969 | 3-(4-Methyl-3-pentenyl)furan | 46.52 | 46.79 | 46.67 | -0.27 |
| 1970 | Decylcyclohexane | 74.16 | 73.89 | 73.89 | 0.27 |
| 1971 | Dimethyl 2-butynedioate | 32.61 | 32.34 | 32.36 | 0.27 |
| 1972 | Linoleic acid | 86.71 | 86.98 | 86.98 | -0.27 |
| 1973 | Methyl benzenesulfonate | 40.80 | 41.07 | 41.25 | -0.27 |
| 1974 | N-Methyl-m-toluidine | 40.31 | 40.58 | 40.62 | -0.27 |
| 1975 | Tridecyl methacrylate | 77.31 | 77.58 | 77.59 | -0.27 |
| 1976 | Triethylphosphine | 40.27 | 40.54 |  | -0.27 |
| 1977 | 1,2-Butanediol | 23.59 | 23.32 | 23.29 | 0.27 |
| 1978 | 1,2-Pentanediol | 28.21 | 27.94 | 27.94 | 0.27 |
| 1979 | 1-Bromo-3-chloropropane | 28.33 | 28.60 | 28.62 | -0.27 |
| 1980 | 2-Methylpyrazine | 27.07 | 26.80 | 26.83 | 0.27 |
| 1981 | 2-Nitro-1-propanol | 23.29 | 23.02 | 22.93 | 0.27 |
| 1982 | Pinacolone | 30.05 | 30.32 | 30.29 | -0.27 |
| 1983 | 3-(Dimethylamino)-1-propanol | 30.93 | 30.66 | 30.65 | 0.27 |
| 1984 | 1,1-Dichlorobutane | 30.54 | 30.27 | 30.26 | 0.27 |
| 1985 | Cyclopropylamine | 17.57 | 17.30 | 17.32 | 0.27 |
| 1986 | Diethyleneglycol | 25.33 | 25.06 | 25.06 | 0.27 |
| 1987 | Formic acid methylester | 13.03 | 13.30 | 13.29 | -0.27 |
| 1988 | Methyl 3-methoxypropanoate | 28.43 | 28.70 | 28.71 | -0.27 |
| 1989 | Methylchloroacetate | 22.30 | 22.57 | 22.57 | -0.27 |
| 1990 | Methylethanolamine | 21.06 | 20.79 | 20.80 | 0.27 |
| 1991 | Methyl formate | 13.03 | 13.30 | 13.28 | -0.27 |
| 1992 | N,N-Diethylformamide | 28.90 | 29.17 | 29.28 | -0.27 |
| 1993 | N-Methylpiperidone | 31.43 | 31.70 | 31.73 | -0.27 |
| 1994 | trans-3-Penten-2-one | 25.45 | 25.72 | 25.77 | -0.27 |
| 1995 | n-Propyl trifluoacetate | 27.70 | 27.43 | 27.42 | 0.27 |
| 1996 | 1-Ethyl-2-propyn-1-ylether | 24.71 | 24.98 | 24.98 | -0.27 |
| 1997 | 2-Fluoroethanol | 12.96 | 12.69 | 12.70 | 0.27 |
| 1998 | Ethyl chlorohydroxyacetate | 28.58 | 28.85 | 28.76 | -0.27 |
| 1999 | 1,6-Dibromohexane | 45.19 | 45.46 | 45.48 | -0.27 |
| 2000 | 1-(1-Methylethoxy)propane | 31.81 | 31.54 | 31.54 | 0.27 |
| 2001 | 2,4-Dimethyl-1,3-pentadiene | 34.45 | 34.18 | 34.18 | 0.27 |
| 2002 | 3,3-Dimethyl-1-pentene | 34.01 | 34.28 | 34.30 | -0.27 |
| 2003 | 3-Pyridinepropanol | 39.95 | 39.68 | 39.67 | 0.27 |
| 2004 | 3-Bromohexane | 37.40 | 37.67 | 37.68 | -0.27 |
| 2005 | 4,6,6-Trimethylbicyclo[3.1.1]hept-3-en-2-ol | 45.64 | 45.37 | 45.34 | 0.27 |
| 2006 | 5-Ethyl-2-norbornene | 39.14 | 39.41 | 39.40 | -0.27 |
| 2007 | 7-Methylquinoline | 47.10 | 46.83 | 46.84 | 0.27 |
| 2008 | Di-n-propylsulfone | 38.95 | 38.68 | 38.63 | 0.27 |
| 2009 | Ethyltrimethoxysilane | 37.01 | 37.28 | 37.35 | -0.27 |
| 2010 | Methyl methoxyacetate | 23.81 | 24.08 | 24.05 | -0.27 |
| 2011 | 1,2-Dimethoxy-4-allylbenzene | 53.29 | 53.56 | 53.58 | -0.27 |
| 2012 | N-(3-(Trimethoxysilyl)propyl)-1,2-ethanediamine | 58.21 | 57.94 | 57.91 | 0.27 |
| 2013 | 1-Heptanethiol | 42.35 | 42.08 | 42.08 | 0.27 |
| 2014 | N-Methylcyclohexylamine | 35.53 | 35.80 | 35.87 | -0.27 |
| 2015 | trans-2-Methyl-3-hexene | 34.74 | 34.47 | 34.49 | 0.27 |
| 2016 | Triisopropyl phosphate | 55.79 | 56.06 | 56.21 | -0.27 |
| 2017 | Trimethoxyphenylsilane | 52.32 | 52.59 | 52.67 | -0.27 |
| 2018 | Octadecylbenzene | 110.29 | 110.02 | 110.02 | 0.27 |
| 2019 | 1-Chlorododecane | 62.65 | 62.37 | 62.36 | 0.28 |
| 2020 | 2-Ethylthiophene | 33.91 | 34.19 | 34.20 | -0.28 |
| 2021 | 5-Methylquinoline | 46.55 | 46.83 | 46.85 | -0.28 |
| 2022 | alpha-(Chloromethyl)benzenemethanol | 41.98 | 42.26 | 42.27 | -0.28 |
| 2023 | Diisoamyl ether | 50.26 | 49.98 | 49.98 | 0.28 |
| 2024 | Ethyl 3-bromopropanoate | 34.55 | 34.83 | 34.80 | -0.28 |
| 2025 | Refrigerant 216 | 26.51 | 26.79 | 26.96 | -0.28 |
| 2026 | 1-(Chloromethoxy)propane | 27.36 | 27.08 | 27.02 | 0.28 |
| 2027 | Isobutyl bromide | 28.10 | 28.38 | 28.39 | -0.28 |
| 2028 | 2,4-Pentanediol | 28.20 | 27.92 | 27.90 | 0.28 |
| 2029 | 4-Pentyn-1-ol | 24.35 | 24.63 | 24.66 | -0.28 |
| 2030 | 2-Methoxyethyl acetate | 28.44 | 28.72 | 28.70 | -0.28 |
| 2031 | Acetic acid methylenediester | 28.35 | 28.63 | 28.67 | -0.28 |
| 2032 | N-Ethyl-1,2-ethanediamine | 27.68 | 27.40 | 27.41 | 0.28 |
| 2033 | t-Butylformamide | 29.11 | 28.83 | 28.66 | 0.28 |
| 2034 | Propiolic acid | 15.92 | 15.64 | 15.64 | 0.28 |
| 2035 | Vinylidenechloride | 20.43 | 20.71 | 20.73 | -0.28 |
| 2036 | 1,1-Difluoro-1,2,2-trichloroethane | 25.93 | 26.21 | 26.30 | -0.28 |
| 2037 | 1,2-Dibutoxyethane | 52.04 | 51.76 | 51.74 | 0.28 |
| 2038 | 1-Bromo-4-(trifluoromethyl)benzene | 39.10 | 39.38 | 39.36 | -0.28 |
| 2039 | 1-Bromo-4-methylpentane | 37.90 | 37.62 | 37.64 | 0.28 |
| 2040 | 2-Chloroiodobenzene | 43.64 | 43.92 | 43.95 | -0.28 |
| 2041 | 1-Chloro-3-iodopropane | 34.06 | 33.78 | 33.75 | 0.28 |
| 2042 | 1-Fluoro-3-methoxybenzene | 32.89 | 33.17 | 33.16 | -0.28 |
| 2043 | Isopropenylacetate | 26.90 | 26.62 | 26.60 | 0.28 |
| 2044 | 2-Decanone | 48.50 | 48.78 | 48.75 | -0.28 |
| 2045 | Methyl cellosolve | 19.33 | 19.05 | 19.04 | 0.28 |
| 2046 | 2-Methylthiophene | 29.29 | 29.57 | 29.51 | -0.28 |
| 2047 | 2-Phenyl-1-propanol | 42.34 | 42.06 | 42.09 | 0.28 |
| 2048 | s-Butylbenzene | 45.03 | 45.31 | 45.31 | -0.28 |
| 2049 | 2-Pyrone | 24.62 | 24.34 | 24.32 | 0.28 |
| 2050 | 3,3-Dimethyl-2-butanol | 31.49 | 31.21 | 31.23 | 0.28 |
| 2051 | 3,6-Dimethyloctane | 48.54 | 48.26 | 48.32 | 0.28 |
| 2052 | 3-Methoxypyridine | 30.53 | 30.81 | 30.80 | -0.28 |
| 2053 | 4-Acetylmorpholine | 33.08 | 33.36 | 33.25 | -0.28 |
| 2054 | 4-Methyl-3-heptanol | 40.68 | 40.40 | 40.40 | 0.28 |
| 2055 | 5-Decanol | 49.95 | 49.67 | 49.73 | 0.28 |
| 2056 | Acrylic acid chloride | 21.18 | 21.46 | 21.49 | -0.28 |
| 2057 | Anisole | 32.94 | 33.22 | 33.22 | -0.28 |
| 2058 | Benzotrichloride | 45.92 | 46.20 | 46.24 | -0.28 |
| 2059 | beta-Farnesene | 70.64 | 70.92 | 70.94 | -0.28 |
| 2060 | Camphene | 43.50 | 43.78 | 43.77 | -0.28 |
| 2061 | Cyclohexylideneacetonitrile | 36.51 | 36.79 | 36.78 | -0.28 |
| 2062 | Dihydro-3-methyl-2(3H)-furanone | 24.59 | 24.87 | 24.86 | -0.28 |
| 2063 | Diphenyl sulfide | 59.77 | 59.49 | 59.46 | 0.28 |
| 2064 | Ethyl hydrosorbate | 40.64 | 40.92 | 40.92 | -0.28 |
| 2065 | Hexabromoethane | 59.48 | 59.20 |  | 0.28 |
| 2066 | n-Butyl acrylate | 36.34 | 36.06 | 36.06 | 0.28 |
| 2067 | 1-Octanethiol | 46.98 | 46.70 | 46.70 | 0.28 |
| 2068 | Pentyl butanoate | 45.22 | 45.50 | 45.51 | -0.28 |
| 2069 | Phytol | 95.50 | 95.78 | 95.81 | -0.28 |
| 2070 | Diethyl dicarbonate | 34.79 | 34.51 | 34.45 | 0.28 |
| 2071 | Tetradecahydrophenanthrene | 60.04 | 60.32 | 60.38 | -0.28 |
| 2072 | trans-2-Bromo-2-butene | 27.77 | 28.05 | 28.10 | -0.28 |
| 2073 | 2-Butoxyphenol | 48.56 | 48.84 | 48.79 | -0.28 |
| 2074 | Tristearin | 271.13 | 270.84 | 271.32 | 0.29 |
| 2075 | Methyl stearate | 91.97 | 91.68 | 91.67 | 0.29 |
| 2076 | Pentadecylamine | 75.13 | 74.84 | 74.97 | 0.29 |
| 2077 | Tetradicamethylcycloheptasiloxane | 130.84 | 130.55 | 130.55 | 0.29 |
| 2078 | n-Propyliodoacetate | 39.72 | 40.01 | 40.04 | -0.29 |
| 2079 | 1,1,2,2-Tetrabromoethane | 41.75 | 42.04 | 41.98 | -0.29 |
| 2080 | 1,2-Dibromo-3-chloropropane | 36.14 | 36.43 | 36.40 | -0.29 |
| 2081 | 1,3,6-Tricyanohexane | 42.93 | 43.22 | 43.19 | -0.29 |
| 2082 | Tetramethylene glycol | 23.63 | 23.34 | 23.42 | 0.29 |
| 2083 | 1,4-Dichlorobenzene | 36.31 | 36.02 | 35.98 | 0.29 |
| 2084 | 1-Bromo-4-ethoxybenzene | 45.64 | 45.35 | 45.35 | 0.29 |
| 2085 | 1-Isopropyl-4-nitrobenzene | 47.57 | 47.28 | 47.25 | 0.29 |
| 2086 | 1-Methyl-3-phenoxybenzene | 57.74 | 58.03 | 58.03 | -0.29 |
| 2087 | 2-(Isopropylamino)ethanol | 30.28 | 29.99 | 29.98 | 0.29 |
| 2088 | 2-Bromoallylalcohol | 24.85 | 24.56 | 24.61 | 0.29 |
| 2089 | 2-Hydroxy-1-phenyl-1-propanone | 42.24 | 42.53 | 42.55 | -0.29 |
| 2090 | 2-Methyl-1,2-propanediamine | 27.68 | 27.39 | 27.38 | 0.29 |
| 2091 | 2-Methyl-1-phenyl-1-propanone | 45.48 | 45.77 | 45.80 | -0.29 |
| 2092 | 2-Methyl-2-butanethiol | 33.72 | 33.43 | 33.44 | 0.29 |
| 2093 | 2-Methyl-2-phenyloxirane | 40.09 | 40.38 | 40.36 | -0.29 |
| 2094 | 2-Methylbutanoyl chloride | 30.58 | 30.87 | 30.89 | -0.29 |
| 2095 | 2-Methylcyclohexanone | 32.48 | 32.77 | 32.81 | -0.29 |
| 2096 | 3,4-Dihydro-2H-pyran | 24.08 | 24.37 | 24.35 | -0.29 |
| 2097 | 3-Bromopyridine | 31.49 | 31.78 | 31.78 | -0.29 |
| 2098 | 3-Methoxybenzaldehyde | 38.95 | 38.66 | 38.67 | 0.29 |
| 2099 | 4-Bromopyridine | 31.49 | 31.78 | 31.83 | -0.29 |
| 2100 | 2,2-Diethoxyethanamine | 36.21 | 36.50 | 36.50 | -0.29 |
| 2101 | Diamylamine | 51.99 | 51.70 | 51.72 | 0.29 |
| 2102 | Dibutyl oxalate | 52.21 | 51.92 | 51.94 | 0.29 |
| 2103 | Diethyl succinate | 42.39 | 42.68 | 42.64 | -0.29 |
| 2104 | Diisopropyl sulfide | 38.56 | 38.27 | 38.23 | 0.29 |
| 2105 | Ethyl 2-hydroxy-3-butenoate | 32.50 | 32.79 | 32.85 | -0.29 |
| 2106 | Ethyl iodoacetate | 35.10 | 35.39 | 35.42 | -0.29 |
| 2107 | Isopropyl lactate | 32.68 | 32.97 | 33.01 | -0.29 |
| 2108 | MTBE | 26.69 | 26.98 | 27.02 | -0.29 |
| 2109 | n-Butyl methyl ether | 27.21 | 26.92 | 26.92 | 0.29 |
| 2110 | 4-Methoxyaniline | 36.96 | 37.25 | 37.37 | -0.29 |
| 2111 | Paraldehyde | 32.57 | 32.28 | 32.25 | 0.29 |
| 2112 | sec-Amyl acetate | 35.95 | 36.24 | 36.25 | -0.29 |
| 2113 | Tetralin | 43.09 | 43.38 | 43.40 | -0.29 |
| 2114 | trans-2,2-Dimethyl-3-hexene | 39.43 | 39.14 | 39.14 | 0.29 |
| 2115 | Triethyl borate | 39.10 | 38.81 | 38.72 | 0.29 |
| 2116 | Triisobutylamine | 61.71 | 61.42 | 61.41 | 0.29 |
| 2117 | Propoxyacetylene | 24.88 | 24.59 | 24.53 | 0.29 |
| 2118 | 1-Ethyl-1H-pyrrole | 30.22 | 30.51 | 30.70 | -0.29 |
| 2119 | 2-Acetylfuran | 29.58 | 29.29 | 29.31 | 0.29 |
| 2120 | 3-Methyl-3-buten-2-ol | 26.02 | 26.31 | 26.30 | -0.29 |
| 2121 | Diisopropyl ether | 31.81 | 31.52 | 31.52 | 0.29 |
| 2122 | N-Nitrosodimethylamine | 19.31 | 19.60 | 19.65 | -0.29 |
| 2123 | t-Butylamine | 24.26 | 23.97 | 23.91 | 0.29 |
| 2124 | Tetramethylsilane | 29.95 | 30.24 | 30.28 | -0.29 |
| 2125 | trans-1-Chloro-1-butene | 25.42 | 25.13 | 25.12 | 0.29 |
| 2126 | 2-Methyl-2,4-pentanediol | 32.91 | 32.62 | 32.67 | 0.29 |
| 2127 | Diethyl glutarate | 47.01 | 47.30 | 47.26 | -0.29 |
| 2128 | Diheptyl ether | 68.81 | 68.52 | 68.51 | 0.29 |
| 2129 | Parathion | 71.73 | 71.44 | 71.55 | 0.29 |
| 2130 | Phosphamidon | 69.15 | 68.86 | 68.71 | 0.29 |
| 2131 | Squalane | 140.83 | 140.54 | 140.56 | 0.29 |
| 2132 | 1,1,3,3-Tetramethyl-1,3-diphenyldisiloxane | 88.87 | 88.57 | 88.64 | 0.30 |
| 2133 | 1-(4-Isopropylphenyl)ethanone | 50.85 | 50.55 | 50.56 | 0.30 |
| 2134 | Margaric acid | 82.58 | 82.28 | 82.28 | 0.30 |
| 2135 | 1-Naphthylamine | 48.79 | 48.49 | 48.47 | 0.30 |
| 2136 | 2-(2-(Diethylamino)ethoxy)ethanol | 45.82 | 46.12 | 46.08 | -0.30 |
| 2137 | 2-Chloronaphthalene | 49.42 | 49.12 | 49.16 | 0.30 |
| 2138 | 2-Chlorothiophene | 29.31 | 29.61 | 29.70 | -0.30 |
| 2139 | 2-n-Hexoxyethanol | 42.47 | 42.17 | 42.17 | 0.30 |
| 2140 | 3-Heptyne | 33.10 | 33.40 | 33.34 | -0.30 |
| 2141 | 3-Methyl-1-hexylamine | 38.15 | 37.85 | 37.88 | 0.30 |
| 2142 | Di-t-butyldisulfide | 55.88 | 56.18 | 56.58 | -0.30 |
| 2143 | Dihexyl sulfide | 65.73 | 65.43 | 65.47 | 0.30 |
| 2144 | Ethyl 2-acetylpentanoate | 45.63 | 45.93 | 45.92 | -0.30 |
| 2145 | Ethyltriethoxysilane | 51.50 | 51.20 | 51.09 | 0.30 |
| 2146 | 2-Methoxyphenol | 34.66 | 34.96 | 34.97 | -0.30 |
| 2147 | Hexadecylamine | 79.76 | 79.46 | 79.47 | 0.30 |
| 2148 | Hexyl octanoate | 68.90 | 68.60 | 68.60 | 0.30 |
| 2149 | Pheniramine | 76.17 | 76.47 | 76.51 | -0.30 |
| 2150 | Trimethyleneglycol | 19.02 | 18.72 | 18.72 | 0.30 |
| 2151 | Neohexane | 30.16 | 29.86 | 29.89 | 0.30 |
| 2152 | Chloroprene | 25.28 | 24.98 | 24.98 | 0.30 |
| 2153 | 2-Fluoroaniline | 30.38 | 30.68 | 30.72 | -0.30 |
| 2154 | 3-Iodo-1-propanol | 30.04 | 30.34 | 30.35 | -0.30 |
| 2155 | 3-Methylpiperidine | 31.37 | 31.07 | 31.05 | 0.30 |
| 2156 | 4-Chlorobutanoyl chloride | 30.79 | 31.09 | 31.07 | -0.30 |
| 2157 | Cyclobutanone | 18.64 | 18.94 | 18.95 | -0.30 |
| 2158 | Ethyl 3-chloropropanoate | 31.53 | 31.83 | 31.82 | -0.30 |
| 2159 | Benzyl alcohol | 32.55 | 32.85 | 32.87 | -0.30 |
| 2160 | 1-Benzylpyrrole | 50.33 | 50.63 | 50.94 | -0.30 |
| 2161 | 2,2,4-Trimethyl-1,3-pentanediol | 42.09 | 41.79 | 41.79 | 0.30 |
| 2162 | 2,4,6-Collidine | 38.61 | 38.91 | 38.93 | -0.30 |
| 2163 | 2-(2-Methylpropoxy)ethanol | 33.20 | 32.90 | 32.89 | 0.30 |
| 2164 | 2-Bromohexane | 37.37 | 37.67 | 37.67 | -0.30 |
| 2165 | 4-s-Butylaniline | 49.04 | 49.34 | 49.32 | -0.30 |
| 2166 | Cyclodecanol | 47.25 | 47.55 | 47.55 | -0.30 |
| 2167 | Di-n-propylamine | 33.52 | 33.22 | 33.28 | 0.30 |
| 2168 | Ethyl 4-bromobutanoate | 39.15 | 39.45 | 39.43 | -0.30 |
| 2169 | Heptanenitrile | 34.01 | 34.31 | 34.33 | -0.30 |
| 2170 | Hexahydro-1-methyl-1H-1,4-diazepine | 35.41 | 35.11 | 35.11 | 0.30 |
| 2171 | Heptadecane | 80.96 | 80.66 | 80.66 | 0.30 |
| 2172 | Tetrapropoxymethane | 68.60 | 68.30 |  | 0.30 |
| 2173 | Triphenylmethane | 80.63 | 80.93 | 80.96 | -0.30 |
| 2174 | 2-Bromotoluene | 38.66 | 38.97 | 38.90 | -0.31 |
| 2175 | 2-Methoxy-1-phenylethanone | 43.19 | 42.88 | 42.91 | 0.31 |
| 2176 | 3,3-Dimethylbutanoyl chloride | 35.23 | 35.54 | 35.60 | -0.31 |
| 2177 | 3,4-Dihydro-2H-1-benzopyran-2-one | 40.76 | 40.45 | 40.48 | 0.31 |
| 2178 | 3,7-Dimethyl-1-octanol | 49.94 | 49.63 | 49.64 | 0.31 |
| 2179 | Ethyl 4-fluorobenzoate | 42.16 | 42.47 | 42.47 | -0.31 |
| 2180 | Isocumene | 40.41 | 40.72 | 40.71 | -0.31 |
| 2181 | 1,3-Butyleneglycol | 23.63 | 23.32 | 23.32 | 0.31 |
| 2182 | 1,2-Oxathiane 2,2-dioxide | 28.23 | 27.92 | 27.85 | 0.31 |
| 2183 | 3,5-Dimethylisoxazole | 25.96 | 26.27 | 26.26 | -0.31 |
| 2184 | cis-1-Chloro-1-butene | 24.82 | 25.13 | 25.12 | -0.31 |
| 2185 | Cyclohexanone | 27.87 | 28.18 | 28.15 | -0.31 |
| 2186 | Iodoacetonitrile | 23.89 | 24.20 | 24.22 | -0.31 |
| 2187 | Methyl dimethylmalonate | 37.73 | 38.04 | 38.01 | -0.31 |
| 2188 | N-Isobutylaniline | 49.26 | 49.57 | 49.56 | -0.31 |
| 2189 | 1-(Chloromethyl)naphthalene | 53.74 | 54.05 | 54.08 | -0.31 |
| 2190 | n-Decyl alcohol | 50.00 | 49.69 | 49.67 | 0.31 |
| 2191 | 1-Ethylcyclopentene | 32.01 | 32.32 | 32.31 | -0.31 |
| 2192 | 1-Heptanol | 36.14 | 35.83 | 35.87 | 0.31 |
| 2193 | 1-Nitrohexane | 35.84 | 35.53 | 35.54 | 0.31 |
| 2194 | 1-Phenyl-2-methyl-2-propanol | 46.46 | 46.77 | 46.80 | -0.31 |
| 2195 | 2,2,3,3-Tetramethylpentane | 43.43 | 43.74 | 43.78 | -0.31 |
| 2196 | Triptane | 34.14 | 34.45 | 34.44 | -0.31 |
| 2197 | 2,3,3-Trimethylpentane | 38.76 | 39.07 | 39.06 | -0.31 |
| 2198 | 2,4,4-Trimethylcyclohexanone | 41.72 | 42.03 | 42.00 | -0.31 |
| 2199 | 2-Bromoanisole | 41.02 | 40.71 | 40.65 | 0.31 |
| 2200 | 3,5-Lupetidine | 35.35 | 35.66 | 35.67 | -0.31 |
| 2201 | 4-Nonanone | 43.85 | 44.16 | 44.21 | -0.31 |
| 2202 | 5-Hexenoic acid | 30.95 | 31.26 | 31.23 | -0.31 |
| 2203 | Allyl sulfide | 37.00 | 37.31 | 37.34 | -0.31 |
| 2204 | alpha-Phenylacetoacetic acid ethylester | 57.54 | 57.85 | 57.77 | -0.31 |
| 2205 | cis-2-Pentene | 24.95 | 25.26 | 25.27 | -0.31 |
| 2206 | Dipropyl succinate | 51.61 | 51.92 | 51.94 | -0.31 |
| 2207 | Ethyl 2,3-dichloropropanoate | 36.93 | 36.62 | 36.57 | 0.31 |
| 2208 | Hexaethyldisiloxane | 75.92 | 76.23 | 76.36 | -0.31 |
| 2209 | Nonanedioyl dichloride | 54.75 | 55.06 | 55.02 | -0.31 |
| 2210 | Phenoxytrimethylsilane | 51.17 | 50.86 | 50.90 | 0.31 |
| 2211 | Benzenemethanethiol | 38.79 | 39.10 | 39.09 | -0.31 |
| 2212 | trans-2-Octenal | 40.09 | 39.78 | 39.81 | 0.31 |
| 2213 | cis-2-Octene | 38.80 | 39.12 | 39.12 | -0.32 |
| 2214 | Sparteine | 71.14 | 70.82 | 70.76 | 0.32 |
| 2215 | 3-Methyl-2,5-furandione | 25.13 | 24.81 | 24.79 | 0.32 |
| 2216 | 1-Ethyl-2-isopropylbenzene | 49.77 | 50.09 | 50.07 | -0.32 |
| 2217 | 1-Methyl-3-phenylpropylamine | 48.46 | 48.78 | 48.80 | -0.32 |
| 2218 | 2,5-Xylidine | 39.97 | 40.29 | 40.23 | -0.32 |
| 2219 | 2,6-Dichlorotoluene | 40.48 | 40.80 | 40.75 | -0.32 |
| 2220 | 2-Ethyl-1,3-dimethylbenzene | 45.34 | 45.66 | 45.66 | -0.32 |
| 2221 | 2-Hexanone | 29.98 | 30.30 | 30.32 | -0.32 |
| 2222 | 2-Methyl-1,3-propanediol | 23.63 | 23.31 | 23.33 | 0.32 |
| 2223 | 2-Methyl-3-pentenoic acid | 31.15 | 31.47 | 31.45 | -0.32 |
| 2224 | 3-Ethyl-2,5-dimethylpyrazine | 41.58 | 41.26 | 41.10 | 0.32 |
| 2225 | 3-Ethyl-2-pentene | 34.12 | 34.44 | 34.44 | -0.32 |
| 2226 | 4-Ethyl-1,3-dimethylbenzene | 45.34 | 45.66 | 45.69 | -0.32 |
| 2227 | 4-Fluoroacetophenone | 36.19 | 36.51 | 36.53 | -0.32 |
| 2228 | 4-Methylcatechol | 34.64 | 34.96 | 34.91 | -0.32 |
| 2229 | 4-Hydroxyundecanoic acid lactone | 52.28 | 52.60 | 52.59 | -0.32 |
| 2230 | 5-Methylisoxazole | 21.35 | 21.67 | 21.81 | -0.32 |
| 2231 | 6-Methyl-1-heptanol | 40.74 | 40.42 | 40.42 | 0.32 |
| 2232 | Acetone oxime | 20.11 | 20.43 | 20.56 | -0.32 |
| 2233 | Benzylideneaniline | 59.73 | 60.05 | 60.08 | -0.32 |
| 2234 | cis-2-Methylcyclopentanol | 28.72 | 29.04 | 29.10 | -0.32 |
| 2235 | cis-Crotyl alcohol | 21.69 | 22.01 | 22.02 | -0.32 |
| 2236 | Cyclohexylbenzene | 52.75 | 52.43 | 52.43 | 0.32 |
| 2237 | Dimethyl 2-methylsuccinate | 37.67 | 37.99 | 37.95 | -0.32 |
| 2238 | Dimethyl suberate | 51.56 | 51.88 | 51.86 | -0.32 |
| 2239 | Fluoroacetone | 16.72 | 16.40 | 16.37 | 0.32 |
| 2240 | Isopentyl nitrite | 31.59 | 31.27 | 31.23 | 0.32 |
| 2241 | Isodecyl acrylate | 63.43 | 63.75 | 63.80 | -0.32 |
| 2242 | Levulinic acid | 26.98 | 27.30 | 27.29 | -0.32 |
| 2243 | Methylconiine | 45.93 | 45.61 | 45.61 | 0.32 |
| 2244 | Methylphosphoric acid ethylester | 26.93 | 26.61 | 26.43 | 0.32 |
| 2245 | N-(2-Aminoethyl)ethanolamine | 29.09 | 28.77 | 28.78 | 0.32 |
| 2246 | n-Nonylmercaptan | 51.64 | 51.32 | 51.34 | 0.32 |
| 2247 | Perfluorocyclobutene | 18.80 | 19.12 | 19.22 | -0.32 |
| 2248 | Tetrahydrofuran | 19.88 | 20.20 | 20.20 | -0.32 |
| 2249 | Tetravinylsilane | 46.88 | 47.20 | 47.51 | -0.32 |
| 2250 | trans-1,2-Dibromoethene | 26.56 | 26.24 | 26.12 | 0.32 |
| 2251 | Dodecylcyclohexane | 83.45 | 83.13 | 83.13 | 0.32 |
| 2252 | Cyclobutylphenylketone | 48.60 | 48.27 | 48.24 | 0.33 |
| 2253 | (Methylthio)acetic acid | 25.35 | 25.68 | 25.75 | -0.33 |
| 2254 | 1,1,2,3-Tetrachloropropane | 35.58 | 35.25 | 35.21 | 0.33 |
| 2255 | 1,1-Dimethylcyclopentane | 32.69 | 32.36 | 32.36 | 0.33 |
| 2256 | Propylenediamine | 22.51 | 22.84 | 22.81 | -0.33 |
| 2257 | 1,4-Dichloronaphthalene | 53.45 | 53.78 | 53.82 | -0.33 |
| 2258 | 1,7-Octadiene | 38.35 | 38.68 | 38.68 | -0.33 |
| 2259 | 1-Acetyl-4-piperidinone | 36.39 | 36.72 | 36.80 | -0.33 |
| 2260 | 1-Butoxy-4-methylbenzene | 52.21 | 51.88 | 51.90 | 0.33 |
| 2261 | 1-Chlorotetradecane | 71.94 | 71.61 | 71.60 | 0.33 |
| 2262 | 1-Isopropylnaphthalene | 58.12 | 58.45 | 58.41 | -0.33 |
| 2263 | 1-Formylnaphthalene | 49.57 | 49.90 | 49.94 | -0.33 |
| 2264 | 1-Undecanol | 54.64 | 54.31 | 54.40 | 0.33 |
| 2265 | 11-Butyldocosane | 122.54 | 122.21 | 122.21 | 0.33 |
| 2266 | 2,2-Diethoxypropane | 38.14 | 37.81 | 37.25 | 0.33 |
| 2267 | 2,6-Dimethylheptane | 43.97 | 43.64 | 43.64 | 0.33 |
| 2268 | Phenethyl alcohol | 37.14 | 37.47 | 37.48 | -0.33 |
| 2269 | 2-t-Butyl-6-methylphenol | 51.55 | 51.88 | 51.90 | -0.33 |
| 2270 | 3-Amino-1,2-propanediol, | 22.45 | 22.12 | 22.09 | 0.33 |
| 2271 | 3-Bromonitrobenzene | 40.45 | 40.78 | 40.81 | -0.33 |
| 2272 | 3-Chloro-2-methylpropene | 25.48 | 25.15 | 25.16 | 0.33 |
| 2273 | 3-Decanone | 48.45 | 48.78 | 48.84 | -0.33 |
| 2274 | 3-Methyl-1-octyn-3-ol | 43.50 | 43.17 | 43.21 | 0.33 |
| 2275 | 5,6,7,8-Tetrahydroquinoline | 40.78 | 41.11 | 41.17 | -0.33 |
| 2276 | Hydrocinnamonitrile | 40.24 | 40.57 | 40.58 | -0.33 |
| 2277 | Diacetone alcohol | 31.40 | 31.73 | 31.72 | -0.33 |
| 2278 | Dimethyl adipate | 42.31 | 42.64 | 42.66 | -0.33 |
| 2279 | Methyl azelate | 56.17 | 56.50 | 56.48 | -0.33 |
| 2280 | Methyl bromoacetate | 25.24 | 25.57 | 25.56 | -0.33 |
| 2281 | N-Butylacetamide | 33.80 | 33.47 | 33.49 | 0.33 |
| 2282 | Propylenecarbonate | 21.40 | 21.73 | 21.80 | -0.33 |
| 2283 | Sulfolane | 26.99 | 27.32 | 27.40 | -0.33 |
| 2284 | 2-Bromo-2-methylbutane | 33.40 | 33.07 | 33.01 | 0.33 |
| 2285 | trans-1,3-Dimethylcyclohexane | 36.57 | 36.90 | 36.90 | -0.33 |
| 2286 | trans-1-Ethyl-4-methylcyclohexane | 41.85 | 41.52 | 41.52 | 0.33 |
| 2287 | Chloromethylchloroformate | 22.60 | 22.93 | 22.99 | -0.33 |
| 2288 | 3-Methylcyclopentanol | 29.37 | 29.04 | 29.02 | 0.33 |
| 2289 | 1,2,2-Trichloropropane | 30.69 | 30.36 | 30.35 | 0.33 |
| 2290 | 2,2,3,3,4,4,4-Heptafluoro-1-butanol | 22.95 | 23.28 | 23.22 | -0.33 |
| 2291 | 2-Bromo-1,3-butadiene | 27.94 | 27.61 | 27.48 | 0.33 |
| 2292 | 3-Methyl-3-penten-1-yne | 28.19 | 27.86 | 27.83 | 0.33 |
| 2293 | cis-2-Bromo-2-butene | 27.72 | 28.05 | 28.10 | -0.33 |
| 2294 | 2-Chloro-2-methylpropane | 25.81 | 25.48 | 25.41 | 0.33 |
| 2295 | 3-Ethyl-1,2-dimethylbenzene | 45.33 | 45.66 | 45.69 | -0.33 |
| 2296 | 1,4-Dibromobutane | 35.88 | 36.22 | 36.26 | -0.34 |
| 2297 | 1,4-Diiodobutane | 46.24 | 46.58 | 46.62 | -0.34 |
| 2298 | 1-(1-Cyclohexen-1-yl)ethanone | 37.06 | 37.40 | 37.43 | -0.34 |
| 2299 | 1-Ethyl-2-pyrrolidinemethanamine | 40.07 | 39.73 | 39.68 | 0.34 |
| 2300 | 1-Methyl-4-(1-methylvinyl)benzene | 45.58 | 45.24 | 45.23 | 0.34 |
| 2301 | 2-(Butylamino)ethanol | 34.93 | 34.59 | 34.58 | 0.34 |
| 2302 | 2-Ethylhexanoyl chloride | 45.07 | 44.73 | 44.71 | 0.34 |
| 2303 | 3-Bromocyclohexene | 35.93 | 35.59 | 35.57 | 0.34 |
| 2304 | Decanedioyl dichloride | 59.34 | 59.68 | 59.68 | -0.34 |
| 2305 | Myristicin | 52.85 | 53.19 | 53.26 | -0.34 |
| 2306 | n-Undecylmercaptan | 60.90 | 60.56 | 60.63 | 0.34 |
| 2307 | Octahydroazocine | 35.38 | 35.72 | 35.69 | -0.34 |
| 2308 | 1,2,3-Trichloropropene | 30.44 | 30.10 | 30.09 | 0.34 |
| 2309 | 1,2-Dimethylhydrazine | 18.42 | 18.76 | 18.98 | -0.34 |
| 2310 | 1-Chloro-3-fluorobenzene | 30.97 | 31.31 | 31.34 | -0.34 |
| 2311 | 1-Methylimidazole | 23.27 | 23.61 | 23.89 | -0.34 |
| 2312 | 2,5-Dimethylpyrazine | 32.06 | 31.72 | 31.72 | 0.34 |
| 2313 | 3-Chlorocyclopentene | 27.59 | 27.93 | 27.92 | -0.34 |
| 2314 | 3-Nitro-2-butanol | 27.96 | 27.62 | 27.56 | 0.34 |
| 2315 | Allyl glycidyl ether | 30.60 | 30.94 | 30.91 | -0.34 |
| 2316 | Butylnitrate | 28.32 | 28.66 | 28.67 | -0.34 |
| 2317 | Butylsilane | 30.90 | 31.24 |  | -0.34 |
| 2318 | Dichloromethylphosphine | 26.10 | 25.76 |  | 0.34 |
| 2319 | Methylhydroperoxide | 10.75 | 10.41 |  | 0.34 |
| 2320 | Perfluorobutanoic acid | 23.87 | 23.53 | 23.52 | 0.34 |
| 2321 | Bis(2-hydroxyethyl) sulfide | 31.55 | 31.21 | 31.25 | 0.34 |
| 2322 | Methyl but-3-yne- 1-carboxylate | 29.32 | 29.66 | 29.62 | -0.34 |
| 2323 | 2-Fluorophenylethylether | 37.47 | 37.81 | 37.83 | -0.34 |
| 2324 | 3-Fluorophenylethylether | 37.47 | 37.81 | 37.88 | -0.34 |
| 2325 | 1,1-Dichlorocyclohexane | 37.63 | 37.29 | 37.26 | 0.34 |
| 2326 | trans-1,2-Dibromocyclohexane | 43.04 | 43.38 | 43.42 | -0.34 |
| 2327 | 1,2-Dimethylnaphthalene | 53.68 | 54.02 | 54.06 | -0.34 |
| 2328 | 1,3-Dimethylnaphthalene | 53.68 | 54.02 | 54.08 | -0.34 |
| 2329 | 1,7-Dichloronaphthalene | 54.12 | 53.78 | 53.66 | 0.34 |
| 2330 | 1-Bromo-3-(trifluoromethyl)benzene | 39.04 | 39.38 | 39.36 | -0.34 |
| 2331 | 1-Bromo-4-(hexyloxy)benzene | 64.17 | 63.83 | 63.92 | 0.34 |
| 2332 | 1-Chloro-1-phenyl-2-propene | 44.97 | 45.31 | 45.32 | -0.34 |
| 2333 | 2,2'-Dimethylbiphenyl | 60.82 | 61.16 | 61.16 | -0.34 |
| 2334 | 2,4-Dimethylthiophene | 33.79 | 34.13 | 34.17 | -0.34 |
| 2335 | 2-Undecanol | 54.63 | 54.29 | 54.35 | 0.34 |
| 2336 | 3-Bromoaniline | 38.56 | 38.22 | 38.14 | 0.34 |
| 2337 | 4-Chlorophenol | 32.76 | 33.10 | 33.13 | -0.34 |
| 2338 | Benzylazide | 38.58 | 38.92 |  | -0.34 |
| 2339 | beta-Cyclocitral | 46.46 | 46.80 | 46.86 | -0.34 |
| 2340 | Butyl 4-oxopentanoate | 45.62 | 45.96 | 45.95 | -0.34 |
| 2341 | Di-n-octylether | 78.10 | 77.76 | 77.76 | 0.34 |
| 2342 | Diisoamyl phthalate | 86.34 | 86.00 | 86.00 | 0.34 |
| 2343 | Dioctyl sulfide | 84.25 | 83.91 | 83.92 | 0.34 |
| 2344 | N-Methylaniline | 35.46 | 35.80 | 35.83 | -0.34 |
| 2345 | trans-1,2-Divinylcyclobutane | 36.84 | 36.50 | 36.48 | 0.34 |
| 2346 | 1-Ethyl-2-phenylhydrazine | 43.87 | 43.52 | 43.34 | 0.35 |
| 2347 | 5-Phenyl-1-pentanol | 50.98 | 51.33 | 51.33 | -0.35 |
| 2348 | cis-Tetrahydro-2,5-dimethylthiophene | 35.80 | 36.15 | 36.16 | -0.35 |
| 2349 | Diethyl isopropylidenemalonate | 52.19 | 51.84 | 51.80 | 0.35 |
| 2350 | Fichtelite | 83.03 | 83.38 | 83.39 | -0.35 |
| 2351 | 1-Decanethiol | 55.59 | 55.94 | 56.01 | -0.35 |
| 2352 | N-Ethyl-1-naphthalenamine | 58.47 | 58.12 | 58.11 | 0.35 |
| 2353 | 6,6-Dimethylbicyclo[3.1.1]hept-2-ene-2-ethanol | 49.66 | 50.01 | 49.99 | -0.35 |
| 2354 | Ethohexadiol | 42.12 | 41.77 | 41.79 | 0.35 |
| 2355 | Tetradecylcyclohexane | 92.72 | 92.37 | 92.37 | 0.35 |
| 2356 | Triethyl citrate | 64.74 | 64.39 | 64.31 | 0.35 |
| 2357 | 1-Bromo-2-chloropropane | 28.93 | 28.58 | 28.61 | 0.35 |
| 2358 | Butane-1,2,4-triol | 25.04 | 24.69 | 24.67 | 0.35 |
| 2359 | Cyclopentanone | 23.21 | 23.56 | 23.58 | -0.35 |
| 2360 | Methylthiocyanate | 19.00 | 18.65 | 18.55 | 0.35 |
| 2361 | Ethyl tribromoacetate | 45.97 | 46.32 |  | -0.35 |
| 2362 | 1-(1-Hydroxycyclohexyl)ethanone | 38.50 | 38.85 | 38.85 | -0.35 |
| 2363 | 1-Bromo-2-butene | 28.80 | 28.45 | 28.47 | 0.35 |
| 2364 | 2',5'-Dimethoxyacetophenone | 49.95 | 49.60 | 49.60 | 0.35 |
| 2365 | 2,3-Dihydro-4H-1-benzothiopyran-4-one | 47.35 | 47.70 | 47.76 | -0.35 |
| 2366 | 2,3-Xylidine | 39.94 | 40.29 | 40.27 | -0.35 |
| 2367 | 2,5-Hexanediol | 32.89 | 32.54 | 32.66 | 0.35 |
| 2368 | Isopropenyl methyl ketone | 25.07 | 25.42 | 25.41 | -0.35 |
| 2369 | 2-Chlorocyclohexanone | 32.62 | 32.97 | 32.96 | -0.35 |
| 2370 | 2-Nitrophenol | 35.38 | 35.03 | 35.04 | 0.35 |
| 2371 | 3-(Methylthio)-1-propene | 28.13 | 28.48 | 28.40 | -0.35 |
| 2372 | 3-Methyl-2-cyclohexen-1-one | 33.13 | 32.78 | 32.75 | 0.35 |
| 2373 | 4-Chlorobutanal | 25.72 | 26.07 | 26.07 | -0.35 |
| 2374 | 4-Methoxy-alpha-methylbenzenemethanol | 43.62 | 43.97 | 44.01 | -0.35 |
| 2375 | 6-Heptenoic acid | 35.53 | 35.88 | 35.90 | -0.35 |
| 2376 | 1,1-Diethoxyethane | 33.43 | 33.08 | 33.03 | 0.35 |
| 2377 | Acetaldehyde bis(2-chloroethyl)acetal | 43.05 | 42.70 | 42.73 | 0.35 |
| 2378 | Acetoin | 22.06 | 22.41 | 22.42 | -0.35 |
| 2379 | alpha-Bromoacetoacetic acid ethylester | 40.28 | 39.93 | 39.91 | 0.35 |
| 2380 | (Fluoromethyl)benzene | 31.09 | 31.44 | 31.51 | -0.35 |
| 2381 | Butyl 2-methylbutanoate | 45.82 | 45.47 | 45.46 | 0.35 |
| 2382 | Chloroacetyl chloride | 21.50 | 21.85 | 21.87 | -0.35 |
| 2383 | Diphenylsilane | 61.53 | 61.18 |  | 0.35 |
| 2384 | Ethyleneglycol | 14.45 | 14.10 | 14.06 | 0.35 |
| 2385 | N-Allylaniline | 44.43 | 44.78 | 44.77 | -0.35 |
| 2386 | N-Ethylmorpholine | 33.74 | 33.39 | 33.36 | 0.35 |
| 2387 | 1,2,4-Trimethylbenzene | 40.69 | 41.04 | 41.07 | -0.35 |
| 2388 | Triethylsilane | 39.60 | 39.25 | 39.14 | 0.35 |
| 2389 | Diethoxydiphenylsilane | 81.07 | 80.72 | 80.69 | 0.35 |
| 2390 | Methyl 2-benzoylbenzoate | 68.21 | 67.86 | 67.89 | 0.35 |
| 2391 | Methyl linoleate | 91.41 | 91.76 | 91.72 | -0.35 |
| 2392 | Tri-n-butyl phosphate | 69.63 | 69.98 | 70.16 | -0.35 |
| 2393 | Tridecylcyclohexane | 88.10 | 87.75 | 87.92 | 0.35 |
| 2394 | 1-(2,5-Dichlorophenyl)ethanone | 46.24 | 45.88 | 45.88 | 0.36 |
| 2395 | 1-Fluoro-3-isothiocyanatobenzene | 42.29 | 42.65 | 42.73 | -0.36 |
| 2396 | 1-Octene-3-yne | 38.18 | 37.82 | 37.85 | 0.36 |
| 2397 | 1-Phenyl-2-propanol | 41.71 | 42.07 | 42.07 | -0.36 |
| 2398 | 2,4-Dibromotoluene | 46.82 | 46.46 | 46.49 | 0.36 |
| 2399 | 2,4-Difluoroaniline | 30.27 | 30.63 | 30.61 | -0.36 |
| 2400 | 2-Bromoaniline | 37.86 | 38.22 | 38.32 | -0.36 |
| 2401 | 2-Dodecanol | 59.27 | 58.91 | 58.93 | 0.36 |
| 2402 | 2-Ethoxyethanol | 24.05 | 23.69 | 23.68 | 0.36 |
| 2403 | 2-Ethylaniline | 39.77 | 40.13 | 40.11 | -0.36 |
| 2404 | Ethyl 2-furancarboxylate | 35.61 | 35.25 | 35.21 | 0.36 |
| 2405 | 3-Fluoroaniline | 30.32 | 30.68 | 30.65 | -0.36 |
| 2406 | 3-Fluorobenzoyl chloride | 37.47 | 37.11 | 37.07 | 0.36 |
| 2407 | 3-Hexanone | 29.94 | 30.30 | 30.32 | -0.36 |
| 2408 | Allyl iodide | 29.13 | 28.77 | 28.76 | 0.36 |
| 2409 | p-Cresol | 32.86 | 33.22 | 33.23 | -0.36 |
| 2410 | 5-Chloropentanoyl chloride | 35.35 | 35.71 | 35.73 | -0.36 |
| 2411 | 3-Phenylpropylthiol | 47.98 | 48.34 | 48.35 | -0.36 |
| 2412 | Benzyl ether | 62.20 | 62.56 | 62.53 | -0.36 |
| 2413 | Bromoacetic acid | 20.43 | 20.79 | 20.82 | -0.36 |
| 2414 | 3-Carene | 44.38 | 44.02 | 44.02 | 0.36 |
| 2415 | Refrigerant 115 | 16.59 | 16.95 | 16.97 | -0.36 |
| 2416 | Di-s-butylsulfide | 47.15 | 47.51 | 47.73 | -0.36 |
| 2417 | Diethylacetaldehyde | 30.11 | 30.47 | 30.52 | -0.36 |
| 2418 | Diethyl adipate | 51.56 | 51.92 | 51.94 | -0.36 |
| 2419 | Dimethyl thiodipropionate | 50.15 | 50.51 | 50.49 | -0.36 |
| 2420 | Dimethyl disulfide | 27.34 | 27.70 | 28.00 | -0.36 |
| 2421 | Dodecanoyl chloride | 63.60 | 63.24 | 63.22 | 0.36 |
| 2422 | Ethanephosphonic acid diethylester | 40.67 | 41.03 | 41.13 | -0.36 |
| 2423 | Ethanethiol | 19.34 | 18.98 | 18.93 | 0.36 |
| 2424 | Ethyl heptafluorobutanoate | 32.59 | 32.95 | 32.95 | -0.36 |
| 2425 | Ethyl-isoamylketone | 39.15 | 39.51 | 39.48 | -0.36 |
| 2426 | Chloroacetic acid ethylester | 26.85 | 27.21 | 27.23 | -0.36 |
| 2427 | Ethyl hydroperoxide | 15.41 | 15.05 | 14.96 | 0.36 |
| 2428 | (Ethylthio)benzene | 44.22 | 44.58 | 44.72 | -0.36 |
| 2429 | Heneicosane | 99.50 | 99.14 | 99.16 | 0.36 |
| 2430 | N,N-Dimethylpentanamide | 38.14 | 38.50 | 38.56 | -0.36 |
| 2431 | N-(3-Aminopropyl)-N-methyl-1,3-propanediamine | 44.95 | 45.31 | 45.30 | -0.36 |
| 2432 | Nonadecane | 90.26 | 89.90 | 89.90 | 0.36 |
| 2433 | Parasorbic acid | 29.18 | 29.54 | 29.52 | -0.36 |
| 2434 | 2-Methyl-3-hexanone | 34.53 | 34.89 | 34.90 | -0.36 |
| 2435 | Tetrahydro-4H-pyran-4-one | 24.92 | 25.28 | 25.31 | -0.36 |
| 2436 | Thujone | 44.83 | 44.47 | 44.46 | 0.36 |
| 2437 | Triethoxyphenylsilane | 66.15 | 66.51 | 66.55 | -0.36 |
| 2438 | Triisopropoxyvinylsilane | 65.03 | 64.67 | 64.68 | 0.36 |
| 2439 | Tripropylsilane | 52.75 | 53.11 | 53.24 | -0.36 |
| 2440 | 1,3-Dichloro-2-propanol | 27.31 | 26.95 | 26.91 | 0.36 |
| 2441 | 1,3-Dichloroacetone | 25.70 | 26.06 | 26.05 | -0.36 |
| 2442 | 1-Chloro-2-fluorobenzene | 30.95 | 31.31 | 31.32 | -0.36 |
| 2443 | 3-(Dimethylamino)-1-propyne | 26.97 | 27.33 | 27.30 | -0.36 |
| 2444 | 2,2,4-Trimethylhexane | 44.05 | 43.69 | 43.69 | 0.36 |
| 2445 | Ethyl 6-bromohexanoate | 49.05 | 48.69 | 48.66 | 0.36 |
| 2446 | Quinoline | 41.69 | 42.05 | 42.06 | -0.36 |
| 2447 | n-Dodecylmercaptan | 65.55 | 65.18 | 65.28 | 0.37 |
| 2448 | 1,2-Diethylbenzene | 45.13 | 45.50 | 45.48 | -0.37 |
| 2449 | 2-Bromo-1,3,5-trimethylbenzene | 48.16 | 48.53 | 48.55 | -0.37 |
| 2450 | 2-Chloroaniline | 35.76 | 35.39 | 35.35 | 0.37 |
| 2451 | 2-Chloroethylthiobenzene | 49.02 | 49.39 | 49.49 | -0.37 |
| 2452 | 2-Furancarbonitrile | 24.43 | 24.06 | 24.09 | 0.37 |
| 2453 | Cumene | 40.32 | 40.69 | 40.69 | -0.37 |
| 2454 | 3-Ethyl-3-methylpentane | 38.73 | 39.10 | 39.09 | -0.37 |
| 2455 | 3-Methyl-1-heptanol | 40.79 | 40.42 | 40.41 | 0.37 |
| 2456 | alpha,alpha-Dichloroacetoacetic acid ethylester | 41.30 | 41.67 | 41.66 | -0.37 |
| 2457 | alpha-Chloroacetoacetic acid ethylester | 36.52 | 36.89 | 36.87 | -0.37 |
| 2458 | Diethyl methylenesuccinate | 46.67 | 47.04 | 47.06 | -0.37 |
| 2459 | Methyl 4-bromo-2-butenoate | 35.22 | 34.85 | 34.87 | 0.37 |
| 2460 | p-Tolualdehyde | 37.29 | 36.92 | 36.90 | 0.37 |
| 2461 | t-Butylbenzene | 44.99 | 45.36 | 45.36 | -0.37 |
| 2462 | trans-1,4-Hexadiene | 29.31 | 29.68 | 29.68 | -0.37 |
| 2463 | Vinylformate | 17.47 | 17.10 | 17.14 | 0.37 |
| 2464 | 1,2-Propanediol | 19.07 | 18.70 | 18.68 | 0.37 |
| 2465 | 1-Penten-3-one | 25.11 | 25.48 | 25.49 | -0.37 |
| 2466 | 2,3-Dichloropropanoyl chloride | 30.89 | 31.26 | 31.27 | -0.37 |
| 2467 | 3-(Methylamino)propionitrile | 24.26 | 23.89 | 23.94 | 0.37 |
| 2468 | 4-Pentenoic acid | 26.27 | 26.64 | 26.64 | -0.37 |
| 2469 | 5-Ethyldihydro-2(3H)-furanone | 29.87 | 29.50 | 29.49 | 0.37 |
| 2470 | 5-Hydroxy-2-pentanone | 26.68 | 27.05 | 27.13 | -0.37 |
| 2471 | Allyl acetate | 26.45 | 26.82 | 26.82 | -0.37 |
| 2472 | Methyltrichlorosilane | 29.13 | 29.50 | 29.57 | -0.37 |
| 2473 | N-Methyl-1,2-ethylenediamine | 23.21 | 22.84 | 22.81 | 0.37 |
| 2474 | Phosphoric acid ethylester | 22.63 | 23.00 | 23.61 | -0.37 |
| 2475 | Thioglycerol | 26.69 | 26.32 | 26.42 | 0.37 |
| 2476 | 1,2,3,4-Tetrahydro-6-methylnaphthalene | 47.79 | 48.16 | 48.11 | -0.37 |
| 2477 | 1,3-Bis(trimethylsiloxy)benzene | 75.39 | 75.02 | 75.06 | 0.37 |
| 2478 | 1-Bromonaphthalene | 51.58 | 51.95 | 51.93 | -0.37 |
| 2479 | 2-Chlorobenzyl chloride | 40.58 | 40.95 | 40.93 | -0.37 |
| 2480 | 1-Tetralone | 43.47 | 43.84 | 43.86 | -0.37 |
| 2481 | 1-Vinylcyclohexene | 36.37 | 36.74 | 36.73 | -0.37 |
| 2482 | 2,3-Dimethyl-1-hexene | 39.16 | 38.79 | 38.76 | 0.37 |
| 2483 | 2,4-Dibromopentane | 40.51 | 40.88 | 40.80 | -0.37 |
| 2484 | 2-Methyl-5-(1-methylethenyl)cyclohexanone | 45.97 | 46.34 | 46.39 | -0.37 |
| 2485 | 1-(3-Methylphenyl)ethanone | 40.97 | 41.34 | 41.31 | -0.37 |
| 2486 | 3-Piperidinemethanol | 32.81 | 32.44 | 32.42 | 0.37 |
| 2487 | Cyclohexanecarboxaldehyde | 33.34 | 32.97 | 32.96 | 0.37 |
| 2488 | Hexachloroacetone | 45.51 | 45.88 | 45.93 | -0.37 |
| 2489 | Isophthaloylchloride | 47.99 | 47.62 | 47.60 | 0.37 |
| 2490 | N-Methylbenzaldehydeimine | 39.41 | 39.04 | 39.09 | 0.37 |
| 2491 | trans-1-Ethyl-2-methylcyclopentane | 37.27 | 36.90 | 36.90 | 0.37 |
| 2492 | trans-5-Methyl-2-(1-methylvinyl)cyclohexanone | 45.97 | 46.34 | 46.37 | -0.37 |
| 2493 | 1,1-Dimethoxyhexadecane | 88.10 | 88.48 | 88.52 | -0.38 |
| 2494 | 1,2,3,4-Tetrahydroquinoline | 43.40 | 43.02 | 43.01 | 0.38 |
| 2495 | 1,3-Dimethoxybenzene | 40.12 | 39.74 | 39.76 | 0.38 |
| 2496 | 2,2-Dimethyl-3-pentanone | 34.56 | 34.94 | 34.99 | -0.38 |
| 2497 | 2,2-Dimethyloctane | 48.72 | 48.34 | 48.33 | 0.38 |
| 2498 | 2-(1-Methylvinyl)aniline | 44.87 | 44.49 | 44.45 | 0.38 |
| 2499 | 2-Octanone | 39.16 | 39.54 | 39.54 | -0.38 |
| 2500 | 4-Methyl-1-isopropyl-3-cyclohexen-1-ol | 47.20 | 47.58 | 47.63 | -0.38 |
| 2501 | 5-Methyl-3-hexen-2-one | 34.55 | 34.93 | 34.93 | -0.38 |
| 2502 | Dibenzylamine | 63.84 | 64.22 | 64.24 | -0.38 |
| 2503 | Dibutoxymethane | 47.33 | 46.95 | 46.99 | 0.38 |
| 2504 | Diisopentyl sulfide | 56.51 | 56.13 | 56.12 | 0.38 |
| 2505 | Diisopropyl adipate | 61.50 | 61.12 | 61.06 | 0.38 |
| 2506 | Ethyl oleate | 95.98 | 96.36 | 96.49 | -0.38 |
| 2507 | Octanoyl chloride | 44.38 | 44.76 | 44.69 | -0.38 |
| 2508 | Pentadecylcyclohexane | 97.37 | 96.99 | 97.00 | 0.38 |
| 2509 | 1,3-Difluoro-2-propanol | 17.63 | 17.25 | 17.14 | 0.38 |
| 2510 | 2-Chloro-1,3-propanediol | 23.89 | 23.51 | 23.47 | 0.38 |
| 2511 | alpha-Methyl-2-furanmethanol | 29.80 | 30.18 | 30.26 | -0.38 |
| 2512 | Bis(2-chloroethyl) ether | 31.56 | 31.94 | 31.94 | -0.38 |
| 2513 | Dimethyl sulfite | 22.41 | 22.79 | 22.96 | -0.38 |
| 2514 | Ethyl nitroacetate | 28.47 | 28.09 | 28.08 | 0.38 |
| 2515 | n-Crotylacetate | 31.30 | 31.68 | 31.66 | -0.38 |
| 2516 | trans-2,3-Epoxybutane | 20.54 | 20.16 | 20.17 | 0.38 |
| 2517 | 1,3-Butanediolsulfite | 30.31 | 29.93 | 29.82 | 0.38 |
| 2518 | 2-t-Butylaniline | 49.01 | 49.39 | 49.33 | -0.38 |
| 2519 | 2-Chloroacrylaldehyde | 20.79 | 21.17 | 21.19 | -0.38 |
| 2520 | 1,1-Diphenylethylene | 60.96 | 60.58 | 60.53 | 0.38 |
| 2521 | Pyruvaldehyde | 16.72 | 17.10 | 17.17 | -0.38 |
| 2522 | 1,3-Diiodopropane | 41.58 | 41.96 | 42.00 | -0.38 |
| 2523 | 1,9-Dichlorononane | 52.94 | 53.32 | 53.32 | -0.38 |
| 2524 | 1-[(2-Aminoethyl)amino]-2-propanol | 33.75 | 33.37 | 33.38 | 0.38 |
| 2525 | 1-Bromo-2-iodobenzene | 46.37 | 46.75 | 46.72 | -0.38 |
| 2526 | Butylcellosolve | 33.31 | 32.93 | 32.94 | 0.38 |
| 2527 | 2-Nitroethylbenzene | 42.17 | 41.79 | 41.76 | 0.38 |
| 2528 | 4-Pyridinemethanamine | 32.11 | 32.49 | 32.51 | -0.38 |
| 2529 | 4-Chloroaniline | 35.01 | 35.39 | 35.41 | -0.38 |
| 2530 | 4-t-Butylaniline | 49.01 | 49.39 | 49.50 | -0.38 |
| 2531 | Bromocycloheptane | 39.79 | 40.17 | 40.20 | -0.38 |
| 2532 | Dimethylphenylsilane | 45.04 | 45.42 | 45.57 | -0.38 |
| 2533 | Ethyl 2-bromo-2-methylpropanoate | 39.11 | 39.49 | 39.61 | -0.38 |
| 2534 | 4-Allyl-2-methoxyphenol | 48.40 | 48.78 | 48.80 | -0.38 |
| 2535 | Geranyl acetate | 58.94 | 59.32 | 59.28 | -0.38 |
| 2536 | m-Cresylacetate | 42.19 | 42.57 | 42.66 | -0.38 |
| 2537 | Z,E-Nepetalactone | 44.75 | 45.13 | 45.20 | -0.38 |
| 2538 | Tetrahydrofurfuryl methacrylate | 44.44 | 44.82 | 44.84 | -0.38 |
| 2539 | Eicosane | 94.90 | 94.52 | 94.52 | 0.38 |
| 2540 | 1-Chlorohexadecane | 81.24 | 80.85 | 81.00 | 0.39 |
| 2541 | 2-Ethoxy-1,2-diphenylethanone | 71.85 | 72.24 | 72.24 | -0.39 |
| 2542 | 2,4-Dichlorotoluene | 41.19 | 40.80 | 40.75 | 0.39 |
| 2543 | trans-4-Methylcyclohexanol | 34.05 | 33.66 | 33.66 | 0.39 |
| 2544 | 1-Methyl-4-piperidinone | 31.80 | 32.19 | 32.18 | -0.39 |
| 2545 | 2,3-Pentadiene | 25.24 | 25.63 | 25.79 | -0.39 |
| 2546 | [1,1'-Bicyclopentyl]-2-one | 44.09 | 44.48 | 44.52 | -0.39 |
| 2547 | 1,1-Dichloro-2-fluoropropene | 25.03 | 25.42 | 25.22 | -0.39 |
| 2548 | 1,4-Pentadien-3-ol | 25.78 | 26.17 | 26.14 | -0.39 |
| 2549 | 1-(Chloromethyl)-2-fluorobenzene | 35.85 | 36.24 | 36.23 | -0.39 |
| 2550 | 1-Chloro-2-(methylthio)ethane | 28.48 | 28.87 | 28.87 | -0.39 |
| 2551 | 1-Ethoxynaphthalene | 55.23 | 55.62 | 55.66 | -0.39 |
| 2552 | 1-Propynylbenzene | 40.05 | 39.66 | 39.69 | 0.39 |
| 2553 | 2,4,6-Trimethylaniline | 44.68 | 45.07 | 45.02 | -0.39 |
| 2554 | 2,4-Dimethylheptane | 44.03 | 43.64 | 43.64 | 0.39 |
| 2555 | 2-(Bromomethyl)tetrahydrofuran | 32.22 | 32.61 | 32.58 | -0.39 |
| 2556 | 2-(Propylamino)ethanol | 30.36 | 29.97 | 29.97 | 0.39 |
| 2557 | 2-Butylphenol | 46.69 | 47.08 | 47.14 | -0.39 |
| 2558 | 2-Methyl-2-heptanol | 40.90 | 40.51 | 40.55 | 0.39 |
| 2559 | 3,4-Dimethylthiophene | 34.38 | 33.99 | 33.94 | 0.39 |
| 2560 | 3-Ethyl-2,4-dimethyl-1H-pyrrole | 39.43 | 39.82 | 39.53 | -0.39 |
| 2561 | 3-Methyl-1-pentanol | 30.79 | 31.18 | 31.18 | -0.39 |
| 2562 | 3-Methyl-2-pentanone | 29.88 | 30.27 | 30.31 | -0.39 |
| 2563 | 3-Nonenoic acid | 44.97 | 45.36 | 45.34 | -0.39 |
| 2564 | 4-Decanone | 48.39 | 48.78 | 48.78 | -0.39 |
| 2565 | 4-Fluoronitrobenzene | 32.85 | 33.24 | 33.28 | -0.39 |
| 2566 | alpha-Terpineol | 47.19 | 47.58 | 47.60 | -0.39 |
| 2567 | 2-(Acetyloxy)-1-phenylethanone | 47.21 | 47.60 | 47.58 | -0.39 |
| 2568 | Allylacetoacetate | 36.13 | 36.52 | 36.47 | -0.39 |
| 2569 | Allyl isothiocyanate | 30.28 | 30.67 | 30.71 | -0.39 |
| 2570 | Butyroin | 41.28 | 40.89 | 40.91 | 0.39 |
| 2571 | cis-1,2,3,5,6,8a-Hexahydro-4,7-dimethyl-1-isopropylnaphthalene | 66.60 | 66.99 | 67.14 | -0.39 |
| 2572 | o-Cymene | 45.08 | 45.47 | 45.47 | -0.39 |
| 2573 | Diethylamine | 24.37 | 23.98 | 23.96 | 0.39 |
| 2574 | Diisobutyl adipate | 69.95 | 70.34 | 70.30 | -0.39 |
| 2575 | Dimethyl succinate | 33.01 | 33.40 | 33.40 | -0.39 |
| 2576 | 4-Allyl anisole | 46.65 | 47.04 | 47.01 | -0.39 |
| 2577 | Isopropyl 2-furancarboxylate | 40.24 | 39.85 | 39.73 | 0.39 |
| 2578 | 4-Methylquinoline | 46.44 | 46.83 | 46.90 | -0.39 |
| 2579 | Methyl 2-methyl-2-butenoate | 31.99 | 31.60 | 31.61 | 0.39 |
| 2580 | Methyl 4-bromobutanoate | 35.20 | 34.81 | 34.83 | 0.39 |
| 2581 | Methyl phenylacetate | 42.11 | 42.50 | 42.50 | -0.39 |
| 2582 | Propyl hexanoate | 45.89 | 45.50 | 45.48 | 0.39 |
| 2583 | Ethyl myristate | 78.24 | 77.84 | 77.82 | 0.40 |
| 2584 | 1-Bromo-2-vinylbenzene | 43.79 | 43.39 | 43.33 | 0.40 |
| 2585 | 1-Methylcyclohexene | 31.92 | 32.32 | 32.37 | -0.40 |
| 2586 | 1-Phenyl-1,2-propanedione | 42.04 | 41.64 | 41.65 | 0.40 |
| 2587 | sec-Phenethyl alcohol | 37.05 | 37.45 | 37.49 | -0.40 |
| 2588 | 2,6-Dimethyl-4-heptanol | 45.39 | 44.99 | 45.02 | 0.40 |
| 2589 | 2-(2-Butoxyethoxy)ethyl acetate | 53.16 | 53.56 | 53.57 | -0.40 |
| 2590 | 2-Methylstyrene | 41.08 | 40.68 | 40.67 | 0.40 |
| 2591 | 3,4-Dibromohexane | 45.10 | 45.50 | 45.56 | -0.40 |
| 2592 | 3,5-Xylidine | 39.89 | 40.29 | 40.31 | -0.40 |
| 2593 | 3,5-Dimethylanisole | 42.38 | 42.78 | 42.80 | -0.40 |
| 2594 | m-Cresol | 32.82 | 33.22 | 33.23 | -0.40 |
| 2595 | 4-Chloroacetophenone | 41.62 | 41.22 | 41.23 | 0.40 |
| 2596 | 4-Methyl-2-pentyne | 28.35 | 28.75 | 28.79 | -0.40 |
| 2597 | Allyl butanoate | 35.66 | 36.06 | 36.06 | -0.40 |
| 2598 | cis-1,4-Dimethylcyclohexane | 36.50 | 36.90 | 36.90 | -0.40 |
| 2599 | Dichloroacetyl chloride | 26.31 | 26.71 | 26.77 | -0.40 |
| 2600 | Dimethyl carbate | 51.78 | 52.18 | 52.18 | -0.40 |
| 2601 | Durene | 45.42 | 45.82 | 45.86 | -0.40 |
| 2602 | Ethyl 2-methoxybenzoate | 49.44 | 49.04 | 49.06 | 0.40 |
| 2603 | 2-Propanol | 17.73 | 17.33 | 17.33 | 0.40 |
| 2604 | Nitrobenzene | 32.89 | 33.29 | 33.36 | -0.40 |
| 2605 | Nonyl acetate | 54.34 | 54.74 | 54.82 | -0.40 |
| 2606 | n-Valeraldehyde | 25.48 | 25.88 | 25.90 | -0.40 |
| 2607 | Propyl octanoate | 54.34 | 54.74 | 54.75 | -0.40 |
| 2608 | Propylhexedrine | 50.03 | 49.63 | 49.61 | 0.40 |
| 2609 | t-Butylacetylene | 28.30 | 27.90 | 27.89 | 0.40 |
| 2610 | Glyceryltriacetate | 48.68 | 49.08 | 49.02 | -0.40 |
| 2611 | 1,2,2,3-Tetrabromobutane | 51.42 | 51.82 | 51.92 | -0.40 |
| 2612 | Butyl isocyanate | 27.67 | 27.27 | 27.28 | 0.40 |
| 2613 | Diethanolamine | 27.12 | 26.72 | 26.72 | 0.40 |
| 2614 | Ethyl 3-butenoate | 31.04 | 31.44 | 31.44 | -0.40 |
| 2615 | 3-Methyl-2-butanone | 25.25 | 25.65 | 25.62 | -0.40 |
| 2616 | Phenol | 28.04 | 28.44 | 28.45 | -0.40 |
| 2617 | Pyruvic acid | 18.46 | 18.06 | 18.06 | 0.40 |
| 2618 | trans-2-Chloro-2-butene | 25.02 | 25.42 | 25.47 | -0.40 |
| 2619 | trans-2-Hexenal | 30.94 | 30.54 | 30.55 | 0.40 |
| 2620 | 2,5-Dimethyloctane | 48.66 | 48.26 | 48.32 | 0.40 |
| 2621 | 5-Nonanone | 43.76 | 44.16 | 44.13 | -0.40 |
| 2622 | cis-1,3-Dimethylcyclohexane | 37.30 | 36.90 | 36.90 | 0.40 |
| 2623 | Methyl tert-pentyl sulfide | 38.34 | 37.94 | 37.89 | 0.40 |
| 2624 | 1,1-Dibromobutane | 35.53 | 35.94 | 35.97 | -0.41 |
| 2625 | 1-Ethylnaphthalene | 53.45 | 53.86 | 53.89 | -0.41 |
| 2626 | Cyclohexanecarboxylic acid | 33.52 | 33.93 | 33.92 | -0.41 |
| 2627 | Diheptyl sulfide | 75.08 | 74.67 | 74.69 | 0.41 |
| 2628 | Docosane | 104.17 | 103.76 | 103.76 | 0.41 |
| 2629 | Ethyl trans-9-octadecenoate | 95.95 | 96.36 | 96.37 | -0.41 |
| 2630 | Isobutyl urethane | 39.67 | 40.08 | 40.14 | -0.41 |
| 2631 | p-Isopropenylisopropylbenzene | 54.86 | 54.45 | 54.42 | 0.41 |
| 2632 | Tetra(2-ethylbutyl) silicate | 125.51 | 125.92 | 126.08 | -0.41 |
| 2633 | trans-1,3,5-Trimethylcyclohexane | 41.90 | 41.49 | 41.49 | 0.41 |
| 2634 | Triisobutylphosphate | 69.51 | 69.92 | 70.05 | -0.41 |
| 2635 | Methyl-1chloroethylether | 23.17 | 22.76 | 22.76 | 0.41 |
| 2636 | 1,2-Difluorobenzene | 26.19 | 26.60 | 26.64 | -0.41 |
| 2637 | 2,3-Dimethyl-1-butene | 29.96 | 29.55 | 29.55 | 0.41 |
| 2638 | 3-Chloropropanenitrile | 20.23 | 20.64 | 20.65 | -0.41 |
| 2639 | 4-Methyl-1H-imidazole | 23.33 | 23.74 | 23.75 | -0.41 |
| 2640 | 3-Acetyldihydro-2(3H)-furanone | 29.54 | 29.95 | 29.96 | -0.41 |
| 2641 | Caprolactone | 29.11 | 29.52 | 29.53 | -0.41 |
| 2642 | cis-2-Chloro-2-butene | 25.01 | 25.42 | 25.46 | -0.41 |
| 2643 | 4-Methylene-2-oxetanone | 20.29 | 19.88 | 19.80 | 0.41 |
| 2644 | Pentanedinitrile | 24.51 | 24.92 | 24.90 | -0.41 |
| 2645 | Imidazole | 18.77 | 18.36 | 18.29 | 0.41 |
| 2646 | 2-Methyl-1-propanal | 20.82 | 21.23 | 21.20 | -0.41 |
| 2647 | Propyl vinyl ether | 26.66 | 26.25 | 26.29 | 0.41 |
| 2648 | 2-Chloro-1-propanol | 22.55 | 22.14 | 22.15 | 0.41 |
| 2649 | Methyloxiran | 15.15 | 15.56 | 15.56 | -0.41 |
| 2650 | S-Ethyl thioacetate | 29.04 | 28.63 | 28.51 | 0.41 |
| 2651 | s-Butylnitrate | 28.23 | 28.64 | 28.66 | -0.41 |
| 2652 | n-Propylvinylacetate | 35.65 | 36.06 | 36.06 | -0.41 |
| 2653 | Carbitol | 35.06 | 34.65 | 34.66 | 0.41 |
| 2654 | 2-Methylbenzothiazole | 43.93 | 44.34 |  | -0.41 |
| 2655 | 2-Nonenal | 44.81 | 44.40 | 44.36 | 0.41 |
| 2656 | 3-Nonanone | 43.75 | 44.16 | 44.16 | -0.41 |
| 2657 | Methylpelargonate | 49.69 | 50.10 | 50.10 | -0.41 |
| 2658 | Tetrapropoxysilane | 70.19 | 70.60 | 70.82 | -0.41 |
| 2659 | Vitamin E | 134.70 | 135.11 | 135.17 | -0.41 |
| 2660 | 1-Tetradecene | 66.18 | 66.60 | 66.60 | -0.42 |
| 2661 | 2,2,3,3,4-Pentamethylpentane | 47.91 | 48.33 | 48.31 | -0.42 |
| 2662 | 2-Dodecanone | 58.44 | 58.02 | 58.02 | 0.42 |
| 2663 | 2-Methyl-3-hexyne | 32.95 | 33.37 | 33.36 | -0.42 |
| 2664 | Benzalchloride | 40.73 | 41.15 | 41.15 | -0.42 |
| 2665 | Benzyl chloroformate | 43.33 | 42.91 | 42.88 | 0.42 |
| 2666 | Ethyl levulinate | 36.30 | 36.72 | 36.70 | -0.42 |
| 2667 | Indane | 38.34 | 38.76 | 38.78 | -0.42 |
| 2668 | trans-1-Methyl-2-propylcyclopentane | 41.94 | 41.52 | 41.52 | 0.42 |
| 2669 | 1-Methylpyrrole | 25.53 | 25.95 | 25.84 | -0.42 |
| 2670 | 2-Pyridinecarbonitrile | 29.48 | 29.06 | 29.05 | 0.42 |
| 2671 | Cyclobutylamine | 22.34 | 21.92 | 21.91 | 0.42 |
| 2672 | 2,2’-Bioxirane | 20.18 | 19.76 | 19.76 | 0.42 |
| 2673 | Methylcyclopropylketone | 23.95 | 23.53 | 23.53 | 0.42 |
| 2674 | Methylbenzene | 31.06 | 31.48 | 31.47 | -0.42 |
| 2675 | Isobutyroylchloride | 25.83 | 26.25 | 26.23 | -0.42 |
| 2676 | 1,1,2,2,3-Pentachloropropane | 39.61 | 40.03 | 40.09 | -0.42 |
| 2677 | 1-Iodonaphthalene | 56.60 | 57.02 | 56.97 | -0.42 |
| 2678 | 1-Methyl-3-piperidinol | 33.50 | 33.08 | 33.06 | 0.42 |
| 2679 | 2,2,6-Trimethylheptane | 48.73 | 48.31 | 48.31 | 0.42 |
| 2680 | Tetrahydromyrcenol | 50.14 | 49.72 | 49.70 | 0.42 |
| 2681 | 2,6-Xylidine | 39.87 | 40.29 | 40.31 | -0.42 |
| 2682 | 2-Heptyl bromide | 42.71 | 42.29 | 42.30 | 0.42 |
| 2683 | 2-Picoline | 28.79 | 29.21 | 29.22 | -0.42 |
| 2684 | 4,4-Dimethyl-2-pentyne | 33.00 | 33.42 | 33.47 | -0.42 |
| 2685 | 4-Cumylphenol | 42.01 | 42.43 | 42.46 | -0.42 |
| 2686 | 1-Hexen-5-one | 29.68 | 30.10 | 30.10 | -0.42 |
| 2687 | Methyl isoamyl ketone | 34.47 | 34.89 | 34.92 | -0.42 |
| 2688 | alpha-Vetivone | 67.95 | 67.53 | 67.59 | 0.42 |
| 2689 | Butyl cyanoacetate | 35.69 | 36.11 | 36.12 | -0.42 |
| 2690 | Butyl ricinoleate | 106.53 | 106.95 | 106.99 | -0.42 |
| 2691 | cis-1-Chloropropene | 20.09 | 20.51 | 20.52 | -0.42 |
| 2692 | cis-3,7-Dimethyl-2,6-octadienal | 49.36 | 48.94 | 48.89 | 0.42 |
| 2693 | Diethyleneglycol diacetate | 44.82 | 44.40 | 44.39 | 0.42 |
| 2694 | Ethyl bromoacetate | 29.79 | 30.21 | 30.21 | -0.42 |
| 2695 | 1,8-Cineole | 45.47 | 45.89 | 45.89 | -0.42 |
| 2696 | Dimethyl glutarate | 37.60 | 38.02 | 38.04 | -0.42 |
| 2697 | Monomethyl glutarate | 32.82 | 33.24 | 33.22 | -0.42 |
| 2698 | Octyl octanoate | 78.26 | 77.84 | 77.82 | 0.42 |
| 2699 | Phenylacetate | 37.37 | 37.79 | 37.78 | -0.42 |
| 2700 | Tricosane | 108.80 | 108.38 | 108.38 | 0.42 |
| 2701 | 1,3-Dibromopropane | 31.18 | 31.60 | 31.64 | -0.42 |
| 2702 | Vinyloxirane | 19.56 | 19.98 | 19.98 | -0.42 |
| 2703 | Tributyrin | 76.37 | 76.80 | 76.86 | -0.43 |
| 2704 | (2-Chloroethyl)benzene | 40.48 | 40.91 | 40.91 | -0.43 |
| 2705 | 1-Ethyl-2,4,5-trimethylbenzene | 50.01 | 50.44 | 50.48 | -0.43 |
| 2706 | 2-Ethyltoluene | 40.45 | 40.88 | 40.90 | -0.43 |
| 2707 | 1-Hexanol | 31.64 | 31.21 | 31.22 | 0.43 |
| 2708 | 2,4-Dimethyloctane | 48.69 | 48.26 | 48.28 | 0.43 |
| 2709 | 2-Chloroacrolein | 20.79 | 21.22 | 21.18 | -0.43 |
| 2710 | 3,5-Dimethyl-1-hexen-3-ol | 39.85 | 40.28 | 40.32 | -0.43 |
| 2711 | 3-Methyl-3-octanol | 45.56 | 45.13 | 45.11 | 0.43 |
| 2712 | Acetic acid 2-phenoxyethylester | 48.47 | 48.90 | 48.90 | -0.43 |
| 2713 | Tetrahydrofurfuryl acetate | 35.41 | 35.84 | 35.86 | -0.43 |
| 2714 | Allylacrylate | 30.81 | 31.24 | 31.25 | -0.43 |
| 2715 | 2-Chlorobutanoic acid | 27.44 | 27.01 | 27.00 | 0.43 |
| 2716 | GBL | 19.85 | 20.28 | 20.29 | -0.43 |
| 2717 | Butyl chloroformate | 31.60 | 32.03 | 32.06 | -0.43 |
| 2718 | Chlorotrifluoroethylene | 17.52 | 17.09 |  | 0.43 |
| 2719 | p-Menthadiene | 45.46 | 45.89 | 45.89 | -0.43 |
| 2720 | cis-1-Bromopropene | 23.01 | 23.44 | 23.47 | -0.43 |
| 2721 | cis-2-Heptene | 34.07 | 34.50 | 34.53 | -0.43 |
| 2722 | Diisopropyl oxalate | 43.07 | 42.64 | 42.60 | 0.43 |
| 2723 | Dimethylacetamide | 24.21 | 24.64 | 24.68 | -0.43 |
| 2724 | Ethyl 4-bromobenzoate | 50.44 | 50.01 | 50.01 | 0.43 |
| 2725 | Isobutylamine | 24.42 | 23.99 | 23.99 | 0.43 |
| 2726 | Isopentyl lactate | 42.63 | 42.20 | 42.21 | 0.43 |
| 2727 | Methanesulfonic acid | 16.82 | 17.25 |  | -0.43 |
| 2728 | Methyl 10-undecenoate | 58.71 | 59.14 | 59.15 | -0.43 |
| 2729 | Phenylhydrazine | 34.06 | 33.63 | 33.19 | 0.43 |
| 2730 | trans-1-Chloropropene | 20.08 | 20.51 | 20.54 | -0.43 |
| 2731 | Methyl 4-chlorobutanoate | 31.38 | 31.81 | 31.79 | -0.43 |
| 2732 | 1a,2a,4b-1,2,4-Trimethylcyclopentane | 37.30 | 36.87 | 36.87 | 0.43 |
| 2733 | 2-Ethoxynaphthalene | 55.19 | 55.62 | 55.68 | -0.43 |
| 2734 | 5-Iodoacenaphthene | 64.03 | 64.46 | 64.57 | -0.43 |
| 2735 | Hexadecanoyl chloride | 82.15 | 81.72 | 81.75 | 0.43 |
| 2736 | 1,2,3,4-Tetrahydro-1-methylnaphthalene | 47.53 | 47.97 | 47.99 | -0.44 |
| 2737 | 1,2-Dichloro-4-(trichloromethyl)benzene | 55.96 | 55.52 | 55.52 | 0.44 |
| 2738 | 1-Ethynylcyclopentanol | 32.25 | 31.81 | 31.76 | 0.44 |
| 2739 | 2,5-Dimethylhexane | 39.46 | 39.02 | 39.04 | 0.44 |
| 2740 | 2-Ethyl-1,3,5-trimethylbenzene | 50.00 | 50.44 | 50.48 | -0.44 |
| 2741 | 2-Methylbenzo[b]furane | 39.92 | 40.36 | 40.48 | -0.44 |
| 2742 | Diethylketone | 25.24 | 25.68 | 25.69 | -0.44 |
| 2743 | Benzene | 26.26 | 26.70 | 26.70 | -0.44 |
| 2744 | Suberone | 32.36 | 32.80 | 32.81 | -0.44 |
| 2745 | Ethyl 3-oxopentanoate | 36.28 | 36.72 | 36.72 | -0.44 |
| 2746 | Ethyl 4-methylbenzoate | 47.74 | 47.30 | 47.31 | 0.44 |
| 2747 | Ethylcyclobutane | 28.13 | 27.69 | 27.69 | 0.44 |
| 2748 | Ethyldiisopropylamin | 43.51 | 43.07 | 43.20 | 0.44 |
| 2749 | Isobutylcyclopentane | 41.96 | 41.52 | 41.52 | 0.44 |
| 2750 | trans-1,2-Dimethylcyclopentane | 32.72 | 32.28 | 32.28 | 0.44 |
| 2751 | Triethyl acetylcitrate | 73.62 | 74.06 | 74.06 | -0.44 |
| 2752 | Propylallene | 29.57 | 30.01 | 29.97 | -0.44 |
| 2753 | 1,5-Hexadiyne | 25.48 | 25.92 | 25.94 | -0.44 |
| 2754 | 5-Chloro-2-pentanone | 30.05 | 30.49 | 30.50 | -0.44 |
| 2755 | Acetylacetone (enol form) | 27.65 | 27.21 | 27.26 | 0.44 |
| 2756 | Dihydro-3-methylene-2(3H)-furanone | 24.20 | 24.64 | 24.64 | -0.44 |
| 2757 | Propionyl chloride | 21.22 | 21.66 | 21.69 | -0.44 |
| 2758 | 1,1,2,2-Tetrabromobutane | 51.08 | 51.52 | 51.53 | -0.44 |
| 2759 | 2,2,6,6-Tetramethyl-4-piperidinamine | 47.86 | 48.30 | 48.32 | -0.44 |
| 2760 | 3-Methylhexanal | 34.65 | 35.09 | 35.08 | -0.44 |
| 2761 | 1-Phenyl-2-methylpropanol | 46.22 | 46.66 | 46.67 | -0.44 |
| 2762 | Cyclooctanol | 37.87 | 38.31 | 38.33 | -0.44 |
| 2763 | Isopropyl pentanoate | 41.30 | 40.86 | 40.84 | 0.44 |
| 2764 | Methyl 3-methoxybenzoate | 44.84 | 44.40 | 44.36 | 0.44 |
| 2765 | N,N-Dimethyl-N'-benzyl-1,2-ethanediamine | 56.97 | 57.41 | 57.48 | -0.44 |
| 2766 | Triphenylphosphite | 88.43 | 87.99 | 88.12 | 0.44 |
| 2767 | Methyl pentadecanoate | 78.27 | 77.82 | 77.83 | 0.45 |
| 2768 | 11-Decylheneicosane | 145.76 | 145.31 | 145.31 | 0.45 |
| 2769 | Octadecylcyclohexane | 111.30 | 110.85 | 110.86 | 0.45 |
| 2770 | 1,2,3-Trimethylindene | 52.06 | 52.51 | 52.63 | -0.45 |
| 2771 | 1-Phenyl-2-buten-1-one | 46.29 | 45.84 | 45.84 | 0.45 |
| 2772 | 1-Phenylpyrrolidine | 48.22 | 47.77 | 47.75 | 0.45 |
| 2773 | 2,2-Dimethylcyclohexanone | 36.99 | 37.44 | 37.41 | -0.45 |
| 2774 | 2,3-Dichlorotoluene | 41.25 | 40.80 | 40.75 | 0.45 |
| 2775 | 2-Methoxy-1,1'-biphenyl | 58.57 | 58.12 | 58.15 | 0.45 |
| 2776 | 2-Methyloctane | 44.12 | 43.67 | 43.67 | 0.45 |
| 2777 | 2-Thiopheneacetonitrile | 33.59 | 34.04 | 34.10 | -0.45 |
| 2778 | 4,4-Dimethyl-2-pentanone | 34.49 | 34.94 | 34.94 | -0.45 |
| 2779 | Acetic acid 1,3-dichloroisopropylester | 36.17 | 36.62 | 36.61 | -0.45 |
| 2780 | Allyl benzoate | 46.49 | 46.94 | 46.93 | -0.45 |
| 2781 | Ethyl dibromoacetate | 38.19 | 37.74 | 37.69 | 0.45 |
| 2782 | Diethyl phenylmalonate | 63.22 | 62.77 | 62.79 | 0.45 |
| 2783 | Methyl propyl disulfide | 37.18 | 36.73 | 36.66 | 0.45 |
| 2784 | Novoldiamine | 51.54 | 51.09 | 51.04 | 0.45 |
| 2785 | o-Xylene | 35.81 | 36.26 | 36.28 | -0.45 |
| 2786 | Phthaloyl chloride | 47.17 | 47.62 | 47.64 | -0.45 |
| 2787 | (N,N-Dimethylamino)acetonitrile | 24.07 | 24.52 | 24.50 | -0.45 |
| 2788 | 1,2-Pentadiene | 24.94 | 25.39 | 25.44 | -0.45 |
| 2789 | 1,4-Pentadiene | 24.37 | 24.82 | 24.84 | -0.45 |
| 2790 | 2,2,3,3,4,4,5,5-Octafluoro-1-pentanol | 27.48 | 27.93 | 27.98 | -0.45 |
| 2791 | 2-Bromo-1-chloropropane | 29.07 | 28.62 | 28.61 | 0.45 |
| 2792 | 4-Methylcyclopentene | 27.28 | 27.73 | 27.75 | -0.45 |
| 2793 | Ethylene carbonate | 16.68 | 17.13 | 17.19 | -0.45 |
| 2794 | Propane-1,2-dithiol | 31.05 | 31.50 | 31.59 | -0.45 |
| 2795 | Methylisocyanate | 13.02 | 13.47 | 13.58 | -0.45 |
| 2796 | 1,1-Dichloro-2-methylpropane | 29.79 | 30.24 | 30.30 | -0.45 |
| 2797 | (Diethylamino)acetonitrile | 33.19 | 33.64 | 33.62 | -0.45 |
| 2798 | 1,5-Pentanediol diacetate | 46.85 | 47.30 | 47.32 | -0.45 |
| 2799 | 1-(Chloromethyl)-4-fluorobenzene | 35.79 | 36.24 | 36.24 | -0.45 |
| 2800 | 1-Chlorooctadecane | 90.54 | 90.09 | 90.09 | 0.45 |
| 2801 | 2,3-Heptanedione | 34.93 | 35.38 | 35.38 | -0.45 |
| 2802 | 4-(1,1-Dimethylpropyl)cyclohexanone | 50.82 | 51.27 | 51.24 | -0.45 |
| 2803 | 4-Phenyl-1,3-dioxane | 46.00 | 46.45 | 46.27 | -0.45 |
| 2804 | Benzyl methyl ether | 37.35 | 37.80 | 37.82 | -0.45 |
| 2805 | Diethyl sulfite | 32.52 | 32.07 | 31.96 | 0.45 |
| 2806 | Heptacosane | 127.31 | 126.86 | 126.86 | 0.45 |
| 2807 | Hexamethylene diisocyanate | 43.63 | 43.18 | 43.02 | 0.45 |
| 2808 | Isopropyl nicotinate | 45.16 | 44.71 | 44.71 | 0.45 |
| 2809 | Isopropyl trichloroacetate | 42.17 | 41.72 | 41.72 | 0.45 |
| 2810 | N,N-Dimethylbutyramide | 33.43 | 33.88 | 33.79 | -0.45 |
| 2811 | trans-1,3-Dimethylcyclopentane | 32.73 | 32.28 | 32.28 | 0.45 |
| 2812 | trans-1-Bromo-1-butene | 27.61 | 28.06 | 28.10 | -0.45 |
| 2813 | Methyl dipropylmalonate | 56.07 | 56.52 | 56.49 | -0.45 |
| 2814 | Diethyl benzylmalonate | 66.93 | 67.39 | 67.36 | -0.46 |
| 2815 | 1,4-Butanediol diglycidyl ether | 50.46 | 50.92 | 50.98 | -0.46 |
| 2816 | 1,6-Hexanedithiol | 45.54 | 45.08 | 45.04 | 0.46 |
| 2817 | 2,3,3,4-Tetramethylpentane | 43.20 | 43.66 | 43.69 | -0.46 |
| 2818 | 2,3-Dihydrofuran | 19.29 | 19.75 | 19.73 | -0.46 |
| 2819 | 2-Octyn-1-ol | 38.93 | 39.39 | 39.43 | -0.46 |
| 2820 | 4-Chlorotoluene | 35.68 | 36.14 | 36.19 | -0.46 |
| 2821 | 4-Morpholinamine | 27.26 | 26.80 | 26.62 | 0.46 |
| 2822 | Allyl methacrylate | 35.34 | 35.80 | 35.79 | -0.46 |
| 2823 | Allyl propanoate | 30.98 | 31.44 | 31.44 | -0.46 |
| 2824 | Chloromethyl methyl ether | 18.28 | 17.82 | 17.78 | 0.46 |
| 2825 | Perfluorocyclohexene | 28.80 | 29.26 | 29.34 | -0.46 |
| 2826 | 2-Diethylaminoethanol | 34.70 | 35.16 | 35.14 | -0.46 |
| 2827 | Diisopropylamine | 33.72 | 33.26 | 33.26 | 0.46 |
| 2828 | Ethyl 4-chloroacetoacetate | 36.45 | 36.91 | 36.95 | -0.46 |
| 2829 | Ethyl hydroxyacetate | 24.23 | 23.77 | 23.76 | 0.46 |
| 2830 | Pentafluorotoluene | 30.77 | 31.23 | 31.33 | -0.46 |
| 2831 | Phenyltrichlorosilane | 48.92 | 49.38 | 49.36 | -0.46 |
| 2832 | Tri-n-propylphosphate | 55.66 | 56.12 | 56.06 | -0.46 |
| 2833 | 4-Nonylphenol | 69.72 | 70.18 | 70.20 | -0.46 |
| 2834 | 1,2,2-Tribromobutane | 44.46 | 43.99 | 43.87 | 0.47 |
| 2835 | 1,2,3,4-Tetrahydro-1,5-dimethylnaphthalene | 52.28 | 52.75 | 52.72 | -0.47 |
| 2836 | 4-(Ethylthio)-toluene | 48.89 | 49.36 | 49.40 | -0.47 |
| 2837 | 1-Cyclohexene-1-carboxaldehyde | 33.45 | 32.98 | 32.99 | 0.47 |
| 2838 | 1-Methylnaphthalene | 48.77 | 49.24 | 49.27 | -0.47 |
| 2839 | 2-Chloro-4-methylaniline | 40.64 | 40.17 | 40.12 | 0.47 |
| 2840 | 2-Chloro-5-(trifluoromethyl)aniline | 40.11 | 40.58 | 40.56 | -0.47 |
| 2841 | 2-Chlorostyrene | 41.03 | 40.56 | 40.55 | 0.47 |
| 2842 | Methyl-n-propylketone | 25.21 | 25.68 | 25.69 | -0.47 |
| 2843 | 3-Methyl-2-cyclopenten-1-one | 27.69 | 28.16 | 28.16 | -0.47 |
| 2844 | Dill-Apiole | 59.24 | 59.71 | 59.90 | -0.47 |
| 2845 | 4H-Pyran-4-one | 24.70 | 24.23 | 24.18 | 0.47 |
| 2846 | Aconitic acid triethylester | 63.41 | 62.94 | 62.93 | 0.47 |
| 2847 | (Iodomethyl)benzene | 44.94 | 44.47 | 44.49 | 0.47 |
| 2848 | beta-Santalol | 67.76 | 68.23 | 68.28 | -0.47 |
| 2849 | Cyclopropyl methyl ether | 20.63 | 20.16 | 20.16 | 0.47 |
| 2850 | 2,3-Butandione | 21.05 | 21.52 | 21.50 | -0.47 |
| 2851 | Difluoroacetic acid | 13.44 | 12.97 | 12.93 | 0.47 |
| 2852 | Ethyl 2-cyano-2-phenylacetate | 51.11 | 51.58 | 51.64 | -0.47 |
| 2853 | Glycerol 1-oleate | 104.17 | 103.70 | 103.67 | 0.47 |
| 2854 | Glycerol | 20.54 | 20.07 | 20.01 | 0.47 |
| 2855 | Hexachlorocyclopentadiene | 52.34 | 51.87 | 51.49 | 0.47 |
| 2856 | Methyl 3-hexenoate | 35.81 | 36.28 | 36.25 | -0.47 |
| 2857 | Methyl perfluorooctanoate | 48.12 | 48.59 | 48.92 | -0.47 |
| 2858 | Oxalylchloride | 22.25 | 22.72 | 22.76 | -0.47 |
| 2859 | Pentacosane | 118.09 | 117.62 | 117.62 | 0.47 |
| 2860 | Phenyl chloroacetate | 42.13 | 42.60 | 42.62 | -0.47 |
| 2861 | Tri-n-octylamine | 117.42 | 116.95 | 116.98 | 0.47 |
| 2862 | Trimethoxymethylsilane | 32.24 | 32.71 | 32.59 | -0.47 |
| 2863 | Isoamylisonitrile | 29.61 | 30.08 |  | -0.47 |
| 2864 | 3,4-Dihydro-2-methoxy-2H-pyran | 30.02 | 30.49 | 30.60 | -0.47 |
| 2865 | Isobutyric acid | 21.72 | 22.19 | 22.18 | -0.47 |
| 2866 | 2-s-Butylphenol | 46.58 | 47.05 | 47.07 | -0.47 |
| 2867 | 3-Chloro-1,1-diethoxypropane | 42.98 | 42.51 | 42.55 | 0.47 |
| 2868 | Capraldehyde | 48.51 | 48.98 | 48.95 | -0.47 |
| 2869 | Lupeol | 130.68 | 131.16 | 131.24 | -0.48 |
| 2870 | Methyl linolenate | 91.32 | 91.80 | 91.85 | -0.48 |
| 2871 | 2,2-Dichlorohexane | 39.89 | 39.41 | 39.40 | 0.48 |
| 2872 | 1-(2-Hydroxy-5-methylphenyl)-1-propanone | 48.18 | 47.70 | 47.73 | 0.48 |
| 2873 | 1-Nonen-3-ol | 45.33 | 44.85 | 44.84 | 0.48 |
| 2874 | 2-Heptyne | 32.92 | 33.40 | 33.40 | -0.48 |
| 2875 | 2-Methylbenzeneethanol | 41.77 | 42.25 | 42.21 | -0.48 |
| 2876 | 3-Octanone | 39.06 | 39.54 | 39.54 | -0.48 |
| 2877 | 4-Bromo-1,2-dimethoxybenzene | 47.71 | 47.23 | 47.17 | 0.48 |
| 2878 | Allyltrichlorosilane | 38.97 | 38.49 | 38.37 | 0.48 |
| 2879 | Ethchlorvynol | 37.98 | 38.46 | 38.46 | -0.48 |
| 2880 | Hexachlorobutadiene | 49.82 | 49.34 | 49.16 | 0.48 |
| 2881 | N,N-Diethylbutanamide | 42.52 | 43.00 | 43.04 | -0.48 |
| 2882 | Phenanthrene | 61.74 | 62.22 | 62.30 | -0.48 |
| 2883 | t-Butyl isopropyl ketone | 39.05 | 39.53 | 39.58 | -0.48 |
| 2884 | trans-Cyclooctene | 36.52 | 37.00 | 37.02 | -0.48 |
| 2885 | 2-(Methoxymethyl)furan | 30.05 | 30.53 | 30.56 | -0.48 |
| 2886 | 3-Bromothiophene | 31.80 | 32.28 | 32.34 | -0.48 |
| 2887 | Butadiyne | 17.16 | 16.68 | 16.70 | 0.48 |
| 2888 | Butyramide | 24.32 | 23.84 | 23.70 | 0.48 |
| 2889 | Butanoyl chloride | 25.80 | 26.28 | 26.21 | -0.48 |
| 2890 | trans-3-Pentenenitrile | 24.63 | 25.11 | 25.10 | -0.48 |
| 2891 | Trichloroacetylchloride | 31.28 | 31.76 | 31.75 | -0.48 |
| 2892 | Dichloro(4-ethylphenyl)phosphine | 56.02 | 55.54 |  | 0.48 |
| 2893 | 4-Fluorophenylethylether | 37.33 | 37.81 | 37.80 | -0.48 |
| 2894 | 1,1,2-Tribromopropane | 39.63 | 39.15 | 39.14 | 0.48 |
| 2895 | 1,1,3,3-Tetramethylcyclopentane | 42.10 | 41.62 | 41.64 | 0.48 |
| 2896 | 1,3-Dibromo-2-propanol | 32.47 | 32.95 | 32.93 | -0.48 |
| 2897 | 2,2,5,5-Tetramethylhexane | 48.84 | 48.36 | 48.38 | 0.48 |
| 2898 | 2-Heptenal | 34.68 | 35.16 | 35.13 | -0.48 |
| 2899 | Di-2-propenoyldiethyleneglycol | 52.76 | 53.24 | 53.23 | -0.48 |
| 2900 | Diethyl glutaconate | 46.86 | 47.34 | 47.30 | -0.48 |
| 2901 | Methyl benzoylacetate | 48.06 | 47.58 | 47.55 | 0.48 |
| 2902 | Methylphenylsilane | 40.82 | 41.30 | 41.58 | -0.48 |
| 2903 | Tridecanal | 62.36 | 62.84 | 62.86 | -0.48 |
| 2904 | Stearyl mercaptan | 93.39 | 92.90 | 92.90 | 0.49 |
| 2905 | 2,2,3,3-Tetramethylbutane | 38.63 | 39.12 | 39.14 | -0.49 |
| 2906 | 4-(Pentyloxy)benzoyl chloride | 61.69 | 62.18 | 62.24 | -0.49 |
| 2907 | 4-Methoxybenzeneacetonitrile | 41.98 | 42.47 | 42.50 | -0.49 |
| 2908 | Isopropyl benzoate | 46.63 | 47.12 | 47.13 | -0.49 |
| 2909 | trans-Decalin | 44.51 | 44.02 | 44.02 | 0.49 |
| 2910 | Triethylphosphate | 41.77 | 42.26 | 42.39 | -0.49 |
| 2911 | Allyl cyanide | 19.76 | 20.25 | 20.27 | -0.49 |
| 2912 | 4-Fluorobenzonitrile | 31.77 | 31.28 | 31.34 | 0.49 |
| 2913 | 1,3-Cyclohexanedione | 28.15 | 28.64 | 28.62 | -0.49 |
| 2914 | (-)-Carveol | 46.75 | 47.24 | 47.32 | -0.49 |
| 2915 | (1,1-Dimethylpropyl)benzene | 49.49 | 49.98 | 50.00 | -0.49 |
| 2916 | 1,2,3-Tribromopropane | 38.94 | 39.43 | 39.43 | -0.49 |
| 2917 | 1-Bromo-3-methyl-2-butene | 33.50 | 33.01 | 33.05 | 0.49 |
| 2918 | 2,5-Dimethyl-2,5-hexanediamine | 45.33 | 45.82 | 45.98 | -0.49 |
| 2919 | 2,6-Dinitrotoluene | 44.17 | 44.66 | 44.53 | -0.49 |
| 2920 | 2-Hydroxypropionitrile | 17.67 | 17.18 | 17.18 | 0.49 |
| 2921 | 5-Chloroacenaphthene | 56.07 | 56.56 | 56.49 | -0.49 |
| 2922 | Acetoacetic acid butylester | 40.85 | 41.34 | 41.38 | -0.49 |
| 2923 | Cyclohexylformate | 33.79 | 34.28 | 34.29 | -0.49 |
| 2924 | 4-Bromocrotonic acid ethylester | 39.98 | 39.49 | 39.46 | 0.49 |
| 2925 | Methylsalicylate | 40.11 | 39.62 | 39.64 | 0.49 |
| 2926 | Citronellene | 47.58 | 48.07 | 48.08 | -0.49 |
| 2927 | N,N-Diethylaniline | 50.38 | 49.89 | 49.88 | 0.49 |
| 2928 | N,N-Dimethyl-p-toluidine | 45.06 | 45.55 | 45.58 | -0.49 |
| 2929 | Cedrol | 66.93 | 66.44 | 66.44 | 0.49 |
| 2930 | Tri-p-tolylamine | 94.58 | 95.07 |  | -0.49 |
| 2931 | 1,1-Diethoxypropane | 38.20 | 37.70 | 37.70 | 0.50 |
| 2932 | 1,6-Dimethylnaphthalene | 54.52 | 54.02 | 54.06 | 0.50 |
| 2933 | 1-Allylcyclohexanol | 42.31 | 42.81 | 42.83 | -0.50 |
| 2934 | 3-Ethoxytoluene | 43.14 | 42.64 | 42.63 | 0.50 |
| 2935 | 1-Ethylpiperidine | 36.79 | 36.29 | 36.26 | 0.50 |
| 2936 | (2-Iodoethyl)benzene | 49.59 | 49.09 | 49.09 | 0.50 |
| 2937 | 2-t-Butyltoluene | 49.64 | 50.14 | 50.14 | -0.50 |
| 2938 | 2,2,4-Trimethylpiperidine | 40.76 | 40.26 | 40.26 | 0.50 |
| 2939 | 2,2-Dichlorobutane | 29.67 | 30.17 | 30.19 | -0.50 |
| 2940 | 2-Bromo-N,N-dimethylaniline | 47.76 | 48.26 | 48.40 | -0.50 |
| 2941 | 3,7,11-Trimethyl-2,6,10-dodecatrienal | 72.52 | 72.02 | 72.03 | 0.50 |
| 2942 | 3-Ethyl-4-methylhexane | 43.14 | 43.64 | 43.64 | -0.50 |
| 2943 | 4,5-Dihydro-2-methylthiazole | 28.82 | 28.32 |  | 0.50 |
| 2944 | 4-Methylbenzoyl chloride | 42.44 | 41.94 | 41.90 | 0.50 |
| 2945 | 5-Methylbenzo[b]thiophene | 46.56 | 46.06 | 45.97 | 0.50 |
| 2946 | Acetaldehyde | 11.52 | 12.02 | 12.03 | -0.50 |
| 2947 | Acetoxyacetone | 26.98 | 27.48 | 27.47 | -0.50 |
| 2948 | 2-Propene-1-thiol | 22.90 | 23.40 | 23.41 | -0.50 |
| 2949 | Benzyl ethyl ether | 41.94 | 42.44 | 42.41 | -0.50 |
| 2950 | Bromopentafluorobenzene | 33.44 | 33.94 | 34.01 | -0.50 |
| 2951 | Cyclopentadecane | 68.80 | 69.30 | 69.30 | -0.50 |
| 2952 | Dicosamethyldecasiloxane | 197.51 | 198.01 | 198.28 | -0.50 |
| 2953 | Dipropyl fumarate | 52.46 | 51.96 | 51.94 | 0.50 |
| 2954 | Ethyl 3-phenylpropanoate | 51.26 | 51.76 | 51.77 | -0.50 |
| 2955 | Ethyl 2-bromopropanoate | 34.35 | 34.85 | 34.85 | -0.50 |
| 2956 | Ethylene glycol dimethacrylate | 50.90 | 51.40 | 51.42 | -0.50 |
| 2957 | Fluorobenzene | 26.15 | 26.65 | 26.64 | -0.50 |
| 2958 | Heptafluorobutanoic anhydride | 43.93 | 43.43 | 43.13 | 0.50 |
| 2959 | Isobornyl thiocyanoacetate | 66.31 | 66.81 |  | -0.50 |
| 2960 | Isopropyl vinyl ether | 26.73 | 26.23 | 26.26 | 0.50 |
| 2961 | Methacrylic acid chloride | 25.52 | 26.02 | 26.04 | -0.50 |
| 2962 | 1,2-Butadiene | 20.27 | 20.77 | 20.91 | -0.50 |
| 2963 | Methylenecyclobutane | 23.34 | 22.84 | 22.87 | 0.50 |
| 2964 | Pulegone | 47.05 | 46.55 | 46.59 | 0.50 |
| 2965 | 2-Pyrrolidone | 21.61 | 22.11 | 22.10 | -0.50 |
| 2966 | 1-(Chloromethyl)-2-methylbenzene | 41.57 | 41.07 | 41.10 | 0.50 |
| 2967 | Methylvinylacetate | 26.30 | 26.80 | 26.82 | -0.50 |
| 2968 | 1,4-Difluorooctachlorobutane | 58.84 | 59.34 | 59.58 | -0.50 |
| 2969 | n-Butylnonanoate | 64.49 | 63.98 | 63.97 | 0.51 |
| 2970 | 3-Ethoxybenzaldehyde | 43.81 | 43.30 | 43.26 | 0.51 |
| 2971 | 1,1-Dichloro-1-fluoroethane | 20.65 | 21.16 | 21.21 | -0.51 |
| 2972 | 1,1-Diphenylethane | 60.30 | 60.81 | 60.83 | -0.51 |
| 2973 | 2-(Diethoxymethyl)furan | 45.42 | 45.93 | 45.82 | -0.51 |
| 2974 | 2-Bromo-4-methylphenol | 40.20 | 40.71 | 40.70 | -0.51 |
| 2975 | 2-Cyclopentylidenecyclopentanone | 44.97 | 44.46 | 44.43 | 0.51 |
| 2976 | 2-Methyl-1-heptene | 39.33 | 38.82 | 38.83 | 0.51 |
| 2977 | 2-Methyl-2-cyclopenten-1-one | 27.65 | 28.16 | 28.20 | -0.51 |
| 2978 | 4-Fluoroanisole | 32.66 | 33.17 | 33.21 | -0.51 |
| 2979 | 5-Ethylidene-2-norbornene | 38.91 | 39.42 | 39.42 | -0.51 |
| 2980 | Allylmethyldichlorosilane | 38.14 | 38.65 | 38.73 | -0.51 |
| 2981 | Azacycloheptane | 31.61 | 31.10 | 31.12 | 0.51 |
| 2982 | 2-Bromo-1,1-diethoxyethane | 40.38 | 40.89 | 40.89 | -0.51 |
| 2983 | cis-1,3-Dimethylcyclopentane | 32.79 | 32.28 | 32.31 | 0.51 |
| 2984 | Dimethyl 1,3-acetonedicarboxylate | 38.99 | 38.48 | 38.48 | 0.51 |
| 2985 | Ethyl cyclopropanecarboxylate | 30.00 | 29.49 | 29.47 | 0.51 |
| 2986 | Isodurene | 45.31 | 45.82 | 45.82 | -0.51 |
| 2987 | Nitromethylbenzene | 36.66 | 37.17 | 37.20 | -0.51 |
| 2988 | Phenyl vinyl ether | 37.53 | 37.02 | 36.99 | 0.51 |
| 2989 | Undecanenitrile | 52.28 | 52.79 | 52.79 | -0.51 |
| 2990 | 1-(Dimethylamino)-2-propanol | 31.15 | 30.64 | 30.59 | 0.51 |
| 2991 | 3,4-Hexanedione | 30.25 | 30.76 | 30.76 | -0.51 |
| 2992 | Norcarane | 30.67 | 30.16 | 30.16 | 0.51 |
| 2993 | Thiophene | 24.36 | 24.87 | 24.95 | -0.51 |
| 2994 | (Isopropylthio)benzene | 48.97 | 49.48 | 49.48 | -0.51 |
| 2995 | 1-Bromo-2-methylpentane | 38.13 | 37.62 | 37.61 | 0.51 |
| 2996 | 4-Octyne | 37.51 | 38.02 | 38.08 | -0.51 |
| 2997 | 2-(2-Methoxyethyl)pyridine | 40.66 | 40.15 | 40.18 | 0.51 |
| 2998 | 1-Phenyl-1-propanol | 41.55 | 42.07 | 42.08 | -0.52 |
| 2999 | 3,3,4,4-Tetramethylhexane | 47.84 | 48.36 | 48.38 | -0.52 |
| 3000 | 3-Methoxybenzonitrile | 38.37 | 37.85 | 37.89 | 0.52 |
| 3001 | 4-Methylphenethyl alcohol | 41.73 | 42.25 | 42.24 | -0.52 |
| 3002 | cis-9-Octadecenenitrile | 84.65 | 85.17 | 85.19 | -0.52 |
| 3003 | Phenacetin | 51.79 | 51.27 | 51.15 | 0.52 |
| 3004 | Pristane | 90.30 | 89.78 | 89.78 | 0.52 |
| 3005 | 3-Methylmethylenecyclohexane | 37.19 | 36.67 | 36.68 | 0.52 |
| 3006 | 3-Chloro-1,1,1-trifluoropropane | 20.68 | 21.20 | 21.23 | -0.52 |
| 3007 | 3-Ethoxypropanenitrile | 26.27 | 26.79 | 26.84 | -0.52 |
| 3008 | Allyl methyl ether | 21.58 | 22.10 | 22.09 | -0.52 |
| 3009 | 1-Methyl-4-isopropyl-2-nitrobenzene | 51.54 | 52.06 | 52.03 | -0.52 |
| 3010 | 3,3-Diacetoxy-1-propene | 37.14 | 37.66 | 37.64 | -0.52 |
| 3011 | cis-1-Bromo-1-butene | 27.54 | 28.06 | 28.10 | -0.52 |
| 3012 | Ethyl 2-acetyl-4-pentenoate | 45.21 | 45.73 | 45.73 | -0.52 |
| 3013 | Ethyl 4-bromoacetoacetate | 39.39 | 39.91 | 39.87 | -0.52 |
| 3014 | N,N-Dimethyl-1-naphthylamine | 58.01 | 58.53 | 58.58 | -0.52 |
| 3015 | N-Ethyl-2-methylallylamine | 33.48 | 32.96 | 32.93 | 0.52 |
| 3016 | N-Methylalanine ethylester | 34.58 | 35.10 | 35.11 | -0.52 |
| 3017 | Cyclopropylbenzene | 39.09 | 38.57 | 38.57 | 0.52 |
| 3018 | Octadecamethyloctasiloxane | 160.19 | 160.71 | 160.74 | -0.52 |
| 3019 | 3-Ethylpyridine | 33.16 | 33.69 | 33.70 | -0.53 |
| 3020 | 5-Decyne | 46.73 | 47.26 | 47.28 | -0.53 |
| 3021 | 2-(Methylthio)ethanol | 24.90 | 25.43 | 25.51 | -0.53 |
| 3022 | Methyl levulinate | 31.55 | 32.08 | 32.09 | -0.53 |
| 3023 | 1-Chloro-3-(trifluoromethyl)benzene | 36.02 | 36.55 | 36.50 | -0.53 |
| 3024 | 2,5-Dimethyl-1,5-hexadiene | 39.09 | 38.56 | 38.62 | 0.53 |
| 3025 | 2,6-Nonadien-1-ol | 44.62 | 45.15 | 45.15 | -0.53 |
| 3026 | 2-Furanmethanethiol | 31.30 | 31.83 | 31.86 | -0.53 |
| 3027 | 2-Methylanisole | 37.47 | 38.00 | 38.02 | -0.53 |
| 3028 | 2-Methylbenzenemethanamine | 39.15 | 39.68 | 39.68 | -0.53 |
| 3029 | 3,6-Dihydro-4-methyl-2H-pyran | 28.89 | 29.42 | 29.42 | -0.53 |
| 3030 | 4-Nitromethyltoluene | 41.42 | 41.95 | 41.94 | -0.53 |
| 3031 | Ethyl acetoacetate | 31.57 | 32.10 | 32.10 | -0.53 |
| 3032 | 5-Methyl-2(3H)-furanone | 24.21 | 24.74 | 24.80 | -0.53 |
| 3033 | Butyric anhydride | 40.28 | 40.81 | 40.85 | -0.53 |
| 3034 | Diphenyl 2-ethylhexyl phosphate | 99.44 | 99.97 | 100.05 | -0.53 |
| 3035 | Ethoxyacetaldehyde | 22.45 | 22.98 | 22.95 | -0.53 |
| 3036 | Ethyl 2-ethylacetoacetate | 40.78 | 41.31 | 41.29 | -0.53 |
| 3037 | Ethyl N-butylcarbamate | 39.58 | 40.11 | 40.26 | -0.53 |
| 3038 | Octyl phenyl ether | 65.05 | 65.58 | 65.58 | -0.53 |
| 3039 | Phenylisocyanate | 33.95 | 34.48 | 34.36 | -0.53 |
| 3040 | Tetradecane | 67.33 | 66.80 | 66.82 | 0.53 |
| 3041 | trans-2-Hexen-1-ol | 30.72 | 31.25 | 31.25 | -0.53 |
| 3042 | Tris(o-tolyl) phosphite | 101.79 | 102.33 | 102.48 | -0.54 |
| 3043 | 1,3-Dibromobutane | 35.70 | 36.24 | 36.26 | -0.54 |
| 3044 | 1-(2-Hydroxy-4-methylphenyl)ethanone | 43.62 | 43.08 | 43.05 | 0.54 |
| 3045 | 1-Nonyne | 41.20 | 41.74 | 41.80 | -0.54 |
| 3046 | 2,4,6-Trimethylacetophenone | 50.36 | 50.90 | 50.96 | -0.54 |
| 3047 | 2,4-Xylenol | 37.46 | 38.00 | 38.02 | -0.54 |
| 3048 | Eucarvone | 47.24 | 46.70 | 46.68 | 0.54 |
| 3049 | 2-Fluoronitrobenzene | 33.78 | 33.24 | 33.22 | 0.54 |
| 3050 | 2-Hydroxyacetophenone | 38.84 | 38.30 | 38.31 | 0.54 |
| 3051 | 2-Iodo-1,3-dimethylbenzene | 49.36 | 48.82 | 48.88 | 0.54 |
| 3052 | Allyl bromide | 24.13 | 23.59 | 23.56 | 0.54 |
| 3053 | 3-Hexyne | 28.24 | 28.78 | 28.80 | -0.54 |
| 3054 | 3-Methylcyclohexene | 31.81 | 32.35 | 32.35 | -0.54 |
| 3055 | 3-Nitrotoluene | 37.53 | 38.07 | 38.04 | -0.54 |
| 3056 | 4-Heptanone | 34.38 | 34.92 | 34.93 | -0.54 |
| 3057 | 4-Isopropylpyridine | 37.74 | 38.28 | 38.26 | -0.54 |
| 3058 | 4-Methylcyclohexene | 31.81 | 32.35 | 32.35 | -0.54 |
| 3059 | 4-Propylpyridine | 37.77 | 38.31 | 38.35 | -0.54 |
| 3060 | Dimethoxane | 42.32 | 41.78 | 41.65 | 0.54 |
| 3061 | cis-4-Methylcyclohexanol | 34.20 | 33.66 | 33.65 | 0.54 |
| 3062 | Dicyclohexyl ether | 55.54 | 55.00 | 54.98 | 0.54 |
| 3063 | Divinyl sulfone | 28.34 | 27.80 | 26.99 | 0.54 |
| 3064 | Ethanephosphonic acid dimethylester | 31.21 | 31.75 | 31.96 | -0.54 |
| 3065 | Formanilide | 36.06 | 35.52 | 35.25 | 0.54 |
| 3066 | Isobutyl pentanoate | 44.93 | 45.47 | 45.51 | -0.54 |
| 3067 | N-(1-Naphthyl)ethylenediamine | 62.08 | 61.54 | 61.53 | 0.54 |
| 3068 | N-Methylpyrrolidine | 26.57 | 27.11 | 27.17 | -0.54 |
| 3069 | Propanedioyl dichloride | 26.80 | 27.34 | 27.38 | -0.54 |
| 3070 | Safrole | 46.13 | 46.67 | 46.62 | -0.54 |
| 3071 | tert-Butyl 3-oxobutanoate | 40.86 | 41.40 | 41.43 | -0.54 |
| 3072 | Trichloroacetonitrile | 26.47 | 25.93 | 25.95 | 0.54 |
| 3073 | Bromochloroacetic acid | 25.76 | 26.30 |  | -0.54 |
| 3074 | 1,2,2-Tribromopropane | 39.91 | 39.37 | 39.36 | 0.54 |
| 3075 | 2,7-Dimethyloctane | 48.80 | 48.26 | 48.30 | 0.54 |
| 3076 | Geranyl 2-methylpropanoate | 67.99 | 68.53 | 68.50 | -0.54 |
| 3077 | Tributylphosphite | 70.14 | 70.68 | 70.74 | -0.54 |
| 3078 | Tributylsilane | 67.51 | 66.97 | 66.83 | 0.54 |
| 3079 | 1-Phenylpropargyl alcohol | 39.56 | 40.11 | 40.15 | -0.55 |
| 3080 | 2,4-Dimethylquinoline | 51.20 | 51.75 | 51.60 | -0.55 |
| 3081 | 2-Bromododecanoic acid | 66.46 | 67.01 | 67.03 | -0.55 |
| 3082 | Apiole | 60.26 | 59.71 | 59.68 | 0.55 |
| 3083 | 4-Bromo-1,2-dimethylbenzene | 43.20 | 43.75 | 43.79 | -0.55 |
| 3084 | 4-Butylphenol | 46.53 | 47.08 | 47.14 | -0.55 |
| 3085 | 4-Methylcyclohexanemethanol | 38.82 | 38.27 | 38.28 | 0.55 |
| 3086 | 5-Methyl-2-hexyne | 32.82 | 33.37 | 33.42 | -0.55 |
| 3087 | Allyl phenyl ether | 41.73 | 42.28 | 42.27 | -0.55 |
| 3088 | N,N'-Dibenzyl-1,2-ethanediamine | 76.29 | 76.84 | 76.86 | -0.55 |
| 3089 | Benzyl-t-butanol | 50.84 | 51.39 | 51.40 | -0.55 |
| 3090 | Benzylmethylsulfide | 43.63 | 44.18 | 44.29 | -0.55 |
| 3091 | Diethylaminoacetone | 38.32 | 38.87 | 38.90 | -0.55 |
| 3092 | Menthylacetate | 57.68 | 57.13 | 57.15 | 0.55 |
| 3093 | N,N-Dibutylaniline | 68.92 | 68.37 | 68.48 | 0.55 |
| 3094 | (1-Methylvinyl)cyclopropane | 27.98 | 27.43 | 27.44 | 0.55 |
| 3095 | 1,2,4,5-Tetrafluorobenzene | 25.95 | 26.50 | 26.46 | -0.55 |
| 3096 | 1-Chloro-2-butyne | 23.80 | 24.35 | 24.35 | -0.55 |
| 3097 | 2-Methoxy-1,3-butadiene | 27.02 | 26.47 | 26.49 | 0.55 |
| 3098 | 3-Bromofuran | 26.01 | 26.56 | 26.58 | -0.55 |
| 3099 | 1,4-Dimethylnaphthalene | 53.47 | 54.02 | 54.06 | -0.55 |
| 3100 | 2,4-Dinitroanisol | 46.95 | 46.40 | 46.42 | 0.55 |
| 3101 | 3-Fluoronitrobenzene | 32.69 | 33.24 | 33.21 | -0.55 |
| 3102 | Carvacrylmethylketone | 55.88 | 55.33 | 55.33 | 0.55 |
| 3103 | Creosol | 39.19 | 39.74 | 39.79 | -0.55 |
| 3104 | Triisopentylborane | 76.54 | 75.99 | 75.89 | 0.55 |
| 3105 | 2-Ethylstyrene | 45.86 | 45.30 | 45.31 | 0.56 |
| 3106 | Mustard gas | 38.65 | 38.09 | 38.05 | 0.56 |
| 3107 | 2-(Ethylamino)ethanol | 25.91 | 25.35 | 25.34 | 0.56 |
| 3108 | 2-Pentyne | 23.60 | 24.16 | 24.22 | -0.56 |
| 3109 | Ethyl ethylcarbamate | 30.31 | 30.87 | 30.87 | -0.56 |
| 3110 | 1-Methoxybut-1-yn-3-ene | 25.83 | 25.27 | 25.68 | 0.56 |
| 3111 | 1,8-Nonadiene | 42.74 | 43.30 | 43.32 | -0.56 |
| 3112 | 1-Ethynylcyclohexanol | 35.87 | 36.43 | 36.38 | -0.56 |
| 3113 | 1-Nitrooctane | 44.21 | 44.77 | 44.78 | -0.56 |
| 3114 | alpha-Methylbenzeneacetic acid | 41.75 | 42.31 | 42.32 | -0.56 |
| 3115 | 3-Methylbenzenemethanamine | 39.12 | 39.68 | 39.70 | -0.56 |
| 3116 | 4-Methoxybenzeneacetaldehyde | 42.72 | 43.28 | 43.26 | -0.56 |
| 3117 | 3,3-Diethoxy-1-propene | 36.94 | 37.50 | 37.53 | -0.56 |
| 3118 | Bis(1-phenylethyl)amine | 72.94 | 73.50 | 73.51 | -0.56 |
| 3119 | Citral | 49.50 | 48.94 | 48.96 | 0.56 |
| 3120 | Ethyl thiophene-2-carboxylate | 41.17 | 40.61 | 40.56 | 0.56 |
| 3121 | Malaoxon | 71.95 | 71.39 |  | 0.56 |
| 3122 | Methyl 3-mercaptopropanoate | 30.56 | 30.00 | 29.99 | 0.56 |
| 3123 | n-Octylacetate | 49.56 | 50.12 | 50.11 | -0.56 |
| 3124 | Ethyl phenylacetate | 46.58 | 47.14 | 47.13 | -0.56 |
| 3125 | 3,3-Dimethyl-2-butylamine | 32.73 | 33.30 | 33.33 | -0.57 |
| 3126 | Dichlorodiphenylsilane | 69.99 | 69.42 | 69.24 | 0.57 |
| 3127 | 1,1,3-Trimethylcyclopentane | 37.52 | 36.95 | 36.95 | 0.57 |
| 3128 | 1-Bromo-2-(trifluoromethyl)benzene | 38.81 | 39.38 | 39.42 | -0.57 |
| 3129 | 1-Bromo-4-isopropylbenzene | 48.75 | 48.18 | 48.15 | 0.57 |
| 3130 | 1-Phenyl-1H-imidazole | 43.42 | 42.85 |  | 0.57 |
| 3131 | 2-Chlorophenol | 32.53 | 33.10 | 33.13 | -0.57 |
| 3132 | 2-Ethylbutanoyl chloride | 34.92 | 35.49 | 35.51 | -0.57 |
| 3133 | Peonol | 45.39 | 44.82 | 44.85 | 0.57 |
| 3134 | 2-Pentyn-1-ol | 24.96 | 25.53 | 25.58 | -0.57 |
| 3135 | 3,5-Dimethyl-4-heptanol | 44.42 | 44.99 | 45.02 | -0.57 |
| 3136 | 3-Butynylbenzene | 42.81 | 43.38 | 43.43 | -0.57 |
| 3137 | Hydrocinnamyl alcohol | 42.66 | 42.09 | 42.10 | 0.57 |
| 3138 | (3-Bromopropyl)benzene | 47.96 | 48.53 | 48.53 | -0.57 |
| 3139 | 3-Trifluoromethylbenzonitrile | 35.95 | 36.52 | 36.57 | -0.57 |
| 3140 | 3-Vinylpyridine | 34.06 | 33.49 | 33.50 | 0.57 |
| 3141 | 4-Methyl-2-isopropylphenol | 46.64 | 47.21 | 47.17 | -0.57 |
| 3142 | Cyclopentanecarboxaldehyde | 27.78 | 28.35 | 28.37 | -0.57 |
| 3143 | Di-s-butylamine | 43.07 | 42.50 | 42.52 | 0.57 |
| 3144 | Di-t-butyl ketone | 43.63 | 44.20 | 44.25 | -0.57 |
| 3145 | Furfural | 25.44 | 24.87 | 24.86 | 0.57 |
| 3146 | Methylvinyldichlorosilane | 33.33 | 33.90 | 33.97 | -0.57 |
| 3147 | N,N,N',N'-Tetramethyl-1,6-hexanediamine | 55.89 | 56.46 | 56.62 | -0.57 |
| 3148 | Styrene | 36.47 | 35.90 | 35.91 | 0.57 |
| 3149 | Pentaethylbenzene | 73.13 | 73.70 | 73.75 | -0.57 |
| 3150 | Nonacosane | 136.67 | 136.10 | 136.10 | 0.57 |
| 3151 | 5-Hexyn-2-one | 27.76 | 28.34 | 28.37 | -0.58 |
| 3152 | 4-Butylmorpholine | 42.05 | 42.63 | 42.66 | -0.58 |
| 3153 | alpha,alpha-Dimethylacetoacetic acid ethylester | 40.78 | 41.36 | 41.35 | -0.58 |
| 3154 | Citronellal | 48.35 | 48.93 | 48.89 | -0.58 |
| 3155 | Ethyl palmitate | 86.50 | 87.08 | 87.09 | -0.58 |
| 3156 | Heptafluorobutanoyl chloride | 27.01 | 27.59 | 27.61 | -0.58 |
| 3157 | Methyl cyclopropanecarboxylate | 25.43 | 24.85 | 24.84 | 0.58 |
| 3158 | n-Nonylformate | 50.86 | 50.28 | 50.26 | 0.58 |
| 3159 | Trimethylphosphate | 27.76 | 28.34 | 28.61 | -0.58 |
| 3160 | 3-Methyl-2-butenoyl chloride | 31.46 | 30.88 | 30.90 | 0.58 |
| 3161 | 5-Aminomethyl-1,3-benzodioxole | 40.47 | 41.05 | 41.08 | -0.58 |
| 3162 | 2,3,3-Trimethyl-2-butanol | 35.33 | 35.91 | 35.92 | -0.58 |
| 3163 | Solanone | 62.94 | 62.36 | 62.35 | 0.58 |
| 3164 | Hemimellitene | 40.45 | 41.04 | 41.01 | -0.59 |
| 3165 | 1-Acetylpiperidine | 35.67 | 36.26 | 36.32 | -0.59 |
| 3166 | Chlorodiphenylmethane | 60.42 | 61.01 | 60.98 | -0.59 |
| 3167 | Ethyl phenylglyoxylate | 48.19 | 47.60 | 47.57 | 0.59 |
| 3168 | o-Toluic acid | 38.47 | 37.88 | 37.88 | 0.59 |
| 3169 | 1-Chlorocyclohexene | 31.95 | 32.54 | 32.59 | -0.59 |
| 3170 | 3-Chloropropanoyl chloride | 25.88 | 26.47 | 26.48 | -0.59 |
| 3171 | 3-Methylfuran | 23.12 | 23.71 | 23.67 | -0.59 |
| 3172 | 4-Methylpiperidine | 30.48 | 31.07 | 31.07 | -0.59 |
| 3173 | 1-Methoxy-2-propanone | 22.17 | 22.76 | 22.78 | -0.59 |
| 3174 | 1,7-Dimethylnaphthalene | 53.43 | 54.02 | 54.02 | -0.59 |
| 3175 | 2,4-Dimethyl-1-nitrobenzene | 42.26 | 42.85 | 42.83 | -0.59 |
| 3176 | 2-Cyclohexene-1-one | 28.81 | 28.22 | 28.21 | 0.59 |
| 3177 | 2-Nonanol | 44.46 | 45.05 | 45.12 | -0.59 |
| 3178 | 3-Octyne | 37.43 | 38.02 | 37.96 | -0.59 |
| 3179 | 4-Methylbenzeneacetonitrile | 40.14 | 40.73 | 40.73 | -0.59 |
| 3180 | beta-Phellandrene | 45.33 | 45.92 | 45.95 | -0.59 |
| 3181 | Hexanoic anhydride | 59.88 | 59.29 | 59.32 | 0.59 |
| 3182 | Tetracosamethylhendecasiloxane | 217.25 | 216.66 | 216.67 | 0.59 |
| 3183 | 2,4-Dimethylacetophenone | 45.52 | 46.12 | 46.09 | -0.60 |
| 3184 | 1-(1-Methyl-1H-pyrrol-2-yl)ethanone | 37.01 | 36.41 | 36.39 | 0.60 |
| 3185 | Decyl vinyl ether | 59.19 | 58.59 | 58.51 | 0.60 |
| 3186 | 1,2,5,6-Tetrahydropyridine | 25.92 | 26.52 | 26.55 | -0.60 |
| 3187 | Ethylisopropylketone | 29.67 | 30.27 | 30.27 | -0.60 |
| 3188 | Hydroquinone | 29.58 | 30.18 | 30.22 | -0.60 |
| 3189 | Dimethylamine | 14.26 | 14.86 | 14.84 | -0.60 |
| 3190 | 1a,2b,4a-1,2,4-Trimethylcyclopentane | 37.47 | 36.87 | 36.87 | 0.60 |
| 3191 | 2,2,6-Trimethylcyclohexanone | 41.43 | 42.03 | 42.04 | -0.60 |
| 3192 | 2,3-Diaminonaphthalene | 51.92 | 52.52 | 52.50 | -0.60 |
| 3193 | 2,5-Dibromotoluene | 47.06 | 46.46 | 46.45 | 0.60 |
| 3194 | 2-Butenylbenzene | 44.78 | 45.38 | 45.37 | -0.60 |
| 3195 | 2-Hydroxybenzonitrile | 33.67 | 33.07 | 33.08 | 0.60 |
| 3196 | 2-Methoxystyrene | 41.82 | 42.42 | 42.40 | -0.60 |
| 3197 | 2-Phenyl-2-aminopropane | 43.49 | 44.09 | 44.12 | -0.60 |
| 3198 | Tetraethylmethane | 43.12 | 43.72 | 43.71 | -0.60 |
| 3199 | 4-Benzylpyridine | 53.21 | 53.81 | 53.82 | -0.60 |
| 3200 | 4-Chlorobenzenethiol | 38.56 | 39.16 | 39.35 | -0.60 |
| 3201 | 4-Methylbenzeneacetaldehyde | 40.94 | 41.54 | 41.58 | -0.60 |
| 3202 | 4-Tolylacetate | 43.17 | 42.57 | 42.64 | 0.60 |
| 3203 | 2-Chloro-1,1-dimethoxyethane | 29.21 | 28.61 | 28.64 | 0.60 |
| 3204 | Cyclohexanecarbonyl chloride | 37.39 | 37.99 | 38.01 | -0.60 |
| 3205 | Ethyl N-methylcarbamate | 25.71 | 26.31 | 26.31 | -0.60 |
| 3206 | N-Isopropylaniline | 44.40 | 45.00 | 45.07 | -0.60 |
| 3207 | n-Undecanoic acid | 53.96 | 54.56 | 54.57 | -0.60 |
| 3208 | Terpinolene | 45.50 | 46.10 | 46.18 | -0.60 |
| 3209 | 1-Chloro-2,2-difluoropropane | 20.64 | 21.24 | 21.27 | -0.60 |
| 3210 | beta-Elemene | 67.94 | 68.54 | 68.58 | -0.60 |
| 3211 | 1,3-Bis(1-methyl-4-piperidyl)propane | 75.63 | 75.02 | 74.99 | 0.61 |
| 3212 | 1-Fluoronaphthalene | 43.80 | 44.41 | 44.46 | -0.61 |
| 3213 | 1-Pentadecanoic acid | 73.65 | 73.04 | 73.06 | 0.61 |
| 3214 | 2-Butyn-1-al | 19.59 | 20.20 | 20.14 | -0.61 |
| 3215 | 4-(3-Phenylpropyl)pyridine | 62.44 | 63.05 | 63.02 | -0.61 |
| 3216 | 5-Methyl-1-heptanol | 41.03 | 40.42 | 40.42 | 0.61 |
| 3217 | Acetic acid 1,1-dimethylbutylester | 40.33 | 40.94 | 40.93 | -0.61 |
| 3218 | Diethyl allylmalonate | 47.68 | 47.07 | 47.06 | 0.61 |
| 3219 | alpha-Acetylacetoacetic acid ethylester | 43.42 | 42.81 | 42.84 | 0.61 |
| 3220 | cis-Nerolidol | 73.22 | 72.61 | 72.59 | 0.61 |
| 3221 | Diethyl isophthalate | 58.95 | 58.34 | 58.32 | 0.61 |
| 3222 | Diethyl isopropylmalonate | 51.25 | 51.86 | 51.82 | -0.61 |
| 3223 | Dimethylmaleate | 32.83 | 33.44 | 33.46 | -0.61 |
| 3224 | Ethyl pyruvate | 26.87 | 27.48 | 27.52 | -0.61 |
| 3225 | Furoylchloride | 30.50 | 29.89 | 29.84 | 0.61 |
| 3226 | Succinyl chloride | 31.35 | 31.96 | 31.92 | -0.61 |
| 3227 | Tris(2,4-dimethylphenyl) phosphate | 115.36 | 115.97 | 116.14 | -0.61 |
| 3228 | Acetamide | 15.21 | 14.60 | 14.62 | 0.61 |
| 3229 | Formamide | 10.62 | 10.01 | 9.86 | 0.61 |
| 3230 | Furfurylalcohol | 24.97 | 25.58 | 25.61 | -0.61 |
| 3231 | Tetrahydro-2H-pyran-2-one | 24.29 | 24.90 | 24.88 | -0.61 |
| 3232 | Cuscohygrine | 65.85 | 66.46 | 66.60 | -0.61 |
| 3233 | Nonylbenzene | 67.82 | 68.44 | 68.44 | -0.62 |
| 3234 | (1-Bromovinyl)benzene | 43.93 | 43.31 | 43.30 | 0.62 |
| 3235 | 2-Nitroethanol | 19.04 | 18.42 | 18.38 | 0.62 |
| 3236 | 3-Butyn-2-ol | 20.61 | 19.99 | 19.97 | 0.62 |
| 3237 | 4-t-Butylbenzaldehyde | 51.42 | 50.80 | 50.78 | 0.62 |
| 3238 | 6-Methyl-2-heptanol acetate | 50.69 | 50.07 | 50.09 | 0.62 |
| 3239 | Benzyl butanoate | 51.14 | 51.76 | 51.75 | -0.62 |
| 3240 | beta-Methylbenzenepropanoic acid | 46.31 | 46.93 | 46.94 | -0.62 |
| 3241 | Diisopentylamine | 52.26 | 51.64 | 51.64 | 0.62 |
| 3242 | Methyl phenoxyacetate | 43.64 | 44.26 | 44.24 | -0.62 |
| 3243 | o-Cresylacetate | 41.95 | 42.57 | 42.61 | -0.62 |
| 3244 | Pargyline | 51.39 | 52.01 | 52.02 | -0.62 |
| 3245 | Perfluoroheptane | 36.91 | 37.53 | 37.59 | -0.62 |
| 3246 | 1,2-Propanediolsulfite | 24.69 | 25.31 | 25.52 | -0.62 |
| 3247 | 2,3-Dimethyl-1,3-butadiene | 29.94 | 29.32 | 29.30 | 0.62 |
| 3248 | 2-Methoxypyridine | 30.91 | 31.53 |  | -0.62 |
| 3249 | 3-Hexyn-1-ol | 29.53 | 30.15 | 30.19 | -0.62 |
| 3250 | 5-Methylfurfural | 30.55 | 29.93 | 29.65 | 0.62 |
| 3251 | 4-Chlorobutanoic acid | 27.65 | 27.03 | 26.99 | 0.62 |
| 3252 | Vinyl acetate | 22.18 | 21.56 | 21.62 | 0.62 |
| 3253 | 1,1-Bis(methoxycarbonyl)cyclohexane | 49.16 | 49.78 | 49.79 | -0.62 |
| 3254 | Anisyl methyl ketone | 47.08 | 47.70 | 47.72 | -0.62 |
| 3255 | Chlorovinyldimethylsilane | 34.49 | 33.87 | 33.81 | 0.62 |
| 3256 | Dotriacontane | 150.58 | 149.96 | 149.96 | 0.62 |
| 3257 | Farnesol | 72.11 | 72.73 | 72.89 | -0.62 |
| 3258 | 1,1,2,3,3-Pentachloro-1-propene | 40.65 | 40.02 | 39.98 | 0.63 |
| 3259 | 1,3-Dihydro-2H-inden-2-one | 38.59 | 39.22 | 39.23 | -0.63 |
| 3260 | Benzylamine | 34.27 | 34.90 | 34.89 | -0.63 |
| 3261 | Diisopropyl phthalate | 68.17 | 67.54 | 67.46 | 0.63 |
| 3262 | Hexadecyl vinyl ether | 86.94 | 86.31 | 86.29 | 0.63 |
| 3263 | Methyl 3-(methylthio)propanoate | 34.45 | 35.08 | 35.07 | -0.63 |
| 3264 | Methyl abietate | 93.84 | 94.47 | 94.60 | -0.63 |
| 3265 | n-Dodecanal | 57.59 | 58.22 | 58.27 | -0.63 |
| 3266 | 4-Fluorotoluene | 30.80 | 31.43 | 31.45 | -0.63 |
| 3267 | 1,3-Cycloheptadiene | 31.79 | 32.42 | 32.38 | -0.63 |
| 3268 | 1,4-Butanediol dimethacrylate | 60.01 | 60.64 | 60.66 | -0.63 |
| 3269 | 2-Acetylthiophene | 35.28 | 34.65 | 34.61 | 0.63 |
| 3270 | 1-Indanamine | 41.57 | 42.20 | 42.23 | -0.63 |
| 3271 | Dimethylketazine | 36.17 | 35.54 | 35.64 | 0.63 |
| 3272 | Metasystox | 57.04 | 57.67 | 57.74 | -0.63 |
| 3273 | 2-t-Butylfuran | 37.46 | 38.09 | 38.04 | -0.63 |
| 3274 | Allylchlorodimethylsilane | 37.98 | 38.62 | 38.68 | -0.64 |
| 3275 | 1,1-Dimethylhydrazine | 18.72 | 18.08 |  | 0.64 |
| 3276 | Isopropylsulfonic acid | 27.20 | 26.56 |  | 0.64 |
| 3277 | 2,2-Difluoropropane | 15.79 | 16.43 | 16.47 | -0.64 |
| 3278 | ((3-Methylbutoxy)methyl)benzene | 55.63 | 56.27 | 56.28 | -0.64 |
| 3279 | 1,2-Diiodobenzene | 51.18 | 51.82 | 51.98 | -0.64 |
| 3280 | 1-(2-Furanyl)-2-propanone | 33.27 | 33.91 | 33.93 | -0.64 |
| 3281 | 3-Pentyn-1-ol | 24.89 | 25.53 | 25.48 | -0.64 |
| 3282 | 4-Fluorobenzoyl chloride | 36.47 | 37.11 | 37.13 | -0.64 |
| 3283 | 2-Vinylfuran | 29.27 | 28.63 | 28.51 | 0.64 |
| 3284 | 2-Hydroxybenzaldehyde | 34.52 | 33.88 | 33.87 | 0.64 |
| 3285 | Fluoro-2,4,6-trimethylbenzene | 40.35 | 40.99 | 41.04 | -0.64 |
| 3286 | 5-Amino-2-butoxypyridine | 50.08 | 49.44 |  | 0.64 |
| 3287 | Oleic acid | 86.29 | 86.94 | 86.95 | -0.65 |
| 3288 | 4-Methyl-2-pentenoic acid | 32.12 | 31.47 | 31.49 | 0.65 |
| 3289 | 3-Propoxy-1-propene | 30.71 | 31.36 | 31.36 | -0.65 |
| 3290 | Diallyl maleate | 50.91 | 51.56 | 51.50 | -0.65 |
| 3291 | N,N,N',N'-Tetraethyl-1,2-ethylenediamine | 55.57 | 56.22 | 56.20 | -0.65 |
| 3292 | Pentanoic anhydride | 50.70 | 50.05 | 50.15 | 0.65 |
| 3293 | Acetic acid 3-chloropropylester | 32.48 | 31.83 | 31.80 | 0.65 |
| 3294 | Cycloheptene | 31.73 | 32.38 | 32.41 | -0.65 |
| 3295 | Isobutyl vinyl ether | 31.49 | 30.84 | 30.84 | 0.65 |
| 3296 | 2,6-Dimethylpiperidine | 36.41 | 35.76 | 35.78 | 0.65 |
| 3297 | 5,6,7,8-Tetrahydro-1-naphthalenamine | 46.76 | 47.41 | 47.44 | -0.65 |
| 3298 | 1-Ethyl-2-nitrobenzene | 42.03 | 42.69 | 42.67 | -0.66 |
| 3299 | Hexanophenone | 54.38 | 55.04 | 55.04 | -0.66 |
| 3300 | 2,2'-Dimethylazobenzene | 72.12 | 72.78 | 72.96 | -0.66 |
| 3301 | 3,4-Lutidine | 34.51 | 33.85 | 33.83 | 0.66 |
| 3302 | 3-Chloroacetophenone | 40.56 | 41.22 | 41.25 | -0.66 |
| 3303 | alpha-Terpineol acetate | 56.59 | 57.25 | 57.31 | -0.66 |
| 3304 | Arachidonic acid | 95.64 | 96.30 | 96.30 | -0.66 |
| 3305 | Diphenylmethane | 55.56 | 56.22 | 56.31 | -0.66 |
| 3306 | 2,3-Dihydro-1H-indole | 37.74 | 38.40 | 38.40 | -0.66 |
| 3307 | N,N-Diethyl-1,2-ethanediamine | 36.55 | 37.21 | 37.22 | -0.66 |
| 3308 | Vitamin E acetate | 145.12 | 144.46 | 144.50 | 0.66 |
| 3309 | 2-Nitrobutane | 25.61 | 26.27 | 26.41 | -0.66 |
| 3310 | Dimethylpropylamine | 28.63 | 29.29 | 29.29 | -0.66 |
| 3311 | DMMP | 26.30 | 26.96 |  | -0.66 |
| 3312 | Crotonic acid | 22.92 | 22.26 | 22.26 | 0.66 |
| 3313 | 1-Chloro-4-(trifluoromethyl)benzene | 35.89 | 36.55 | 36.61 | -0.66 |
| 3314 | Carvone | 45.69 | 46.35 | 46.31 | -0.66 |
| 3315 | Cyclohexyl propanoate | 44.02 | 43.36 | 43.34 | 0.66 |
| 3316 | Benzal bromide | 47.48 | 46.82 | 46.76 | 0.66 |
| 3317 | 1-t-Butyl-4-methoxybenzene | 65.10 | 65.76 | 65.74 | -0.66 |
| 3318 | 3-Fluorotoluene | 30.76 | 31.43 | 31.43 | -0.67 |
| 3319 | Cyclobutanol | 20.50 | 19.83 | 19.82 | 0.67 |
| 3320 | Dicyclopropyl ketone | 31.29 | 30.62 | 30.61 | 0.67 |
| 3321 | Methoxyacetaldehyde | 17.67 | 18.34 | 18.36 | -0.67 |
| 3322 | 1,2,3,4-Tetrahydro-5-methylnaphthalene | 47.49 | 48.16 | 48.19 | -0.67 |
| 3323 | 1-Hydroxycyclohexanecarbonitrile | 34.29 | 33.62 | 33.58 | 0.67 |
| 3324 | 2-Methylpyrrole | 25.41 | 26.08 | 26.08 | -0.67 |
| 3325 | 2-Naphthylamine | 49.16 | 48.49 | 48.50 | 0.67 |
| 3326 | 3-Butenylbenzene | 44.47 | 45.14 | 45.13 | -0.67 |
| 3327 | 3-Heptanone | 34.25 | 34.92 | 34.92 | -0.67 |
| 3328 | 4-Fluoroiodobenzene | 38.54 | 39.21 | 39.30 | -0.67 |
| 3329 | Ethyl diethylmalonate | 57.23 | 56.56 | 56.53 | 0.67 |
| 3330 | Ethyl linolenate | 95.77 | 96.44 | 96.38 | -0.67 |
| 3331 | N,N'-Diethylurea | 30.64 | 31.31 | 31.64 | -0.67 |
| 3332 | Diallyl succinate | 50.85 | 51.52 | 51.52 | -0.67 |
| 3333 | Methyltriphenoxysilane | 91.85 | 92.53 | 92.77 | -0.68 |
| 3334 | (2-Methyl-1-propenyl)benzene | 46.00 | 45.32 | 45.33 | 0.68 |
| 3335 | 1,1,1,2-Tetrachloroacetone | 35.29 | 35.97 | 36.00 | -0.68 |
| 3336 | 2,6-Dichloroanisole | 43.22 | 42.54 | 42.62 | 0.68 |
| 3337 | 2-Amino-1-butanol | 26.09 | 25.41 | 25.40 | 0.68 |
| 3338 | 3-Bromo-1-propanol | 25.84 | 25.16 | 25.16 | 0.68 |
| 3339 | 3-Ethylstyrene | 45.98 | 45.30 | 45.31 | 0.68 |
| 3340 | 3-Methyl-1H-pyrazole | 23.33 | 24.01 |  | -0.68 |
| 3341 | Benzylisopropylamine | 49.42 | 48.74 | 48.74 | 0.68 |
| 3342 | Benzylisothiocyanate | 45.69 | 46.37 | 46.40 | -0.68 |
| 3343 | Diethylacetamide | 33.08 | 33.76 | 33.80 | -0.68 |
| 3344 | Ethyl valerate | 36.94 | 36.26 | 36.26 | 0.68 |
| 3345 | Ethylene glycol monosulfite | 20.03 | 20.71 | 20.82 | -0.68 |
| 3346 | trans-Geraniol | 48.97 | 49.65 | 49.66 | -0.68 |
| 3347 | Trifluoroacetic anhydride | 23.83 | 23.15 | 22.95 | 0.68 |
| 3348 | Tropine | 39.56 | 40.24 | 40.38 | -0.68 |
| 3349 | 3,3'-Difluorobiphenyl | 52.18 | 51.50 | 51.48 | 0.68 |
| 3350 | 3-Methyl-5-hepten-2-one | 38.87 | 39.55 | 39.57 | -0.68 |
| 3351 | 1,2,3,4-Tetrachloro-5,5-dimethoxy-1,3-cyclopentadiene | 54.16 | 54.85 | 55.28 | -0.69 |
| 3352 | 4-(3-Cyclohexen-1-yl)pyridine | 49.37 | 50.06 | 50.06 | -0.69 |
| 3353 | Thianaphthene | 41.97 | 41.28 | 41.24 | 0.69 |
| 3354 | ((Chloromethoxy)methyl)benzene | 41.89 | 42.58 | 42.67 | -0.69 |
| 3355 | Benzylethylamine | 43.41 | 44.10 | 44.11 | -0.69 |
| 3356 | Butylcyclohexane | 46.86 | 46.17 | 46.17 | 0.69 |
| 3357 | m-Toluic acid | 38.57 | 37.88 | 37.90 | 0.69 |
| 3358 | 4-Ethoxyaniline | 41.20 | 41.89 | 41.91 | -0.69 |
| 3359 | Ethynylbenzene | 34.83 | 34.14 | 34.18 | 0.69 |
| 3360 | Safranal | 47.53 | 46.84 | 46.78 | 0.69 |
| 3361 | 1,2,4-Triazole | 17.50 | 16.81 |  | 0.69 |
| 3362 | 1-Piperidinamine | 30.39 | 29.70 | 29.50 | 0.69 |
| 3363 | 2,3-Pentanedione | 25.45 | 26.14 | 26.22 | -0.69 |
| 3364 | 2-Butyn-1-ol | 20.22 | 20.91 | 20.96 | -0.69 |
| 3365 | Cyclopentene | 22.45 | 23.14 | 23.16 | -0.69 |
| 3366 | 4-Chloroanisole | 37.19 | 37.88 | 37.91 | -0.69 |
| 3367 | Allylbenzene | 39.83 | 40.52 | 40.51 | -0.69 |
| 3368 | Cyclohexyl benzoate | 59.55 | 58.86 | 58.86 | 0.69 |
| 3369 | Diacetylethylamine | 34.48 | 33.79 | 33.46 | 0.69 |
| 3370 | trans-3,7-Dimethyl-2,6-octadien-1-ol formate | 55.55 | 54.86 | 54.86 | 0.69 |
| 3371 | 1-Vinyl-3-nitrobenzene | 43.19 | 42.49 | 42.56 | 0.70 |
| 3372 | Propyl 2-hydroxybenzoate | 49.58 | 48.88 | 48.91 | 0.70 |
| 3373 | 1,1-Dichloro-1,3-butadiene | 30.69 | 29.99 | 29.97 | 0.70 |
| 3374 | Acetic acid 2-chloroethylester | 26.51 | 27.21 | 27.20 | -0.70 |
| 3375 | 1,3,5-Trifluorobenzene | 25.85 | 26.55 | 26.52 | -0.70 |
| 3376 | 2-Fluoro-1,3-butadiene | 20.75 | 20.05 | 20.14 | 0.70 |
| 3377 | Methyl 2,2-dimethylpropanoate | 30.94 | 31.64 | 31.65 | -0.70 |
| 3378 | n-Butylnitrite | 25.98 | 26.68 | 26.76 | -0.70 |
| 3379 | 2-Undecyne | 51.18 | 51.88 | 51.94 | -0.70 |
| 3380 | 4-Methylbenzenepropanal | 45.46 | 46.16 | 46.15 | -0.70 |
| 3381 | Benzylidene fluoride | 30.77 | 31.47 | 31.70 | -0.70 |
| 3382 | Chloropropham | 56.71 | 56.01 | 55.63 | 0.70 |
| 3383 | cis-1,2-Dimethylcyclopropane | 23.74 | 23.04 | 23.04 | 0.70 |
| 3384 | Ylangene | 65.61 | 64.91 | 64.97 | 0.70 |
| 3385 | dl-Lactic acid | 19.65 | 18.95 | 18.93 | 0.70 |
| 3386 | N,N-Diethyl-1-naphthalenamine | 66.95 | 67.65 | 67.71 | -0.70 |
| 3387 | Omethoate | 47.40 | 48.10 |  | -0.70 |
| 3388 | Tripropylene glycol | 53.72 | 54.42 | 54.40 | -0.70 |
| 3389 | 2-((2-Methylphenyl)amino)ethanol | 45.80 | 46.51 | 46.67 | -0.71 |
| 3390 | 2-(Phenylmethylene)heptanal | 65.17 | 64.46 | 64.43 | 0.71 |
| 3391 | Linolenic acid | 86.31 | 87.02 | 86.96 | -0.71 |
| 3392 | 1,1,2,2-Tetrachloro-1-fluoroethane | 31.54 | 30.83 | 30.86 | 0.71 |
| 3393 | 1,3-Pentadiyne | 21.49 | 22.20 | 22.26 | -0.71 |
| 3394 | 1,4-Divinylbenzene | 45.81 | 45.10 | 45.10 | 0.71 |
| 3395 | 1-Bromo-3,5-dimethylbenzene | 43.04 | 43.75 | 43.79 | -0.71 |
| 3396 | 1-Tridecyne | 59.51 | 60.22 | 60.24 | -0.71 |
| 3397 | 2-Chloroethyl acetoacetate | 36.20 | 36.91 | 36.95 | -0.71 |
| 3398 | 2-Octyne | 37.31 | 38.02 | 38.04 | -0.71 |
| 3399 | 4-(1-Methylvinyl)-1-cyclohexene-1-methanol | 41.99 | 42.70 | 42.68 | -0.71 |
| 3400 | 4-Methyl-cis-2-pentene | 29.14 | 29.85 | 29.83 | -0.71 |
| 3401 | Cyclooctanone | 36.71 | 37.42 | 37.42 | -0.71 |
| 3402 | Linalyl acetate | 58.49 | 59.20 | 59.20 | -0.71 |
| 3403 | Methylisothiocyanate | 20.98 | 21.69 | 21.72 | -0.71 |
| 3404 | Phenyl-2-thienylmethanone | 55.48 | 54.77 | 54.73 | 0.71 |
| 3405 | Pentatriacontane | 164.53 | 163.82 | 163.84 | 0.71 |
| 3406 | ((4-tert-Butylphenoxy)methyl)oxirane | 60.00 | 60.72 | 60.69 | -0.72 |
| 3407 | 1,1-Dimethylcyclopropane | 23.84 | 23.12 | 23.15 | 0.72 |
| 3408 | 1,2,3,4-Tetramethylbenzene | 45.10 | 45.82 | 45.86 | -0.72 |
| 3409 | 1,8-Diaminonaphthalene | 53.24 | 52.52 | 52.52 | 0.72 |
| 3410 | 2-(Benzylamino)ethanol | 44.75 | 45.47 | 45.52 | -0.72 |
| 3411 | 2-Methylundecanal | 57.47 | 58.19 | 58.18 | -0.72 |
| 3412 | 2-t-Butylphenol | 46.38 | 47.10 | 47.12 | -0.72 |
| 3413 | 3,4-Dichloro-1,4-dioxane | 31.36 | 32.08 | 32.52 | -0.72 |
| 3414 | alpha-Ionone | 61.25 | 60.53 | 60.55 | 0.72 |
| 3415 | Benzoxazole | 32.74 | 33.46 |  | -0.72 |
| 3416 | cis-1,4-Hexadiene | 28.96 | 29.68 | 29.65 | -0.72 |
| 3417 | cis- Cyclooctene | 36.28 | 37.00 | 36.98 | -0.72 |
| 3418 | Dibenzyl malonate | 79.02 | 78.30 | 78.28 | 0.72 |
| 3419 | Linalol | 48.81 | 49.53 | 49.59 | -0.72 |
| 3420 | Thioanisole | 39.45 | 40.17 | 40.17 | -0.72 |
| 3421 | trans-Cinnamyl acetate | 52.52 | 51.80 | 51.77 | 0.72 |
| 3422 | 2-Ethylfuran | 28.11 | 28.83 | 28.92 | -0.72 |
| 3423 | Cyclohexene | 27.04 | 27.76 | 27.74 | -0.72 |
| 3424 | 2-Phenylfuran | 45.05 | 44.33 | 44.34 | 0.72 |
| 3425 | 4-Phenyl-2-butanone | 45.08 | 45.80 | 45.79 | -0.72 |
| 3426 | 1,2,3-Propanetriol trihexanoate | 103.79 | 104.52 | 104.45 | -0.73 |
| 3427 | 5-Hydroxy-3-methyl-1-phenylpyrazole | 49.63 | 50.36 | 50.63 | -0.73 |
| 3428 | 1-Methyl-4-isopropyl-7-oxabicyclo[2.2.1]heptane | 46.62 | 45.89 | 45.87 | 0.73 |
| 3429 | 2-Nitrotoluene | 37.34 | 38.07 | 38.07 | -0.73 |
| 3430 | cis-1,3-Pentadiene | 25.79 | 25.06 | 25.04 | 0.73 |
| 3431 | Di-t-pentyl peroxide | 53.39 | 52.66 | 52.30 | 0.73 |
| 3432 | Dichloromethylphenylsilane | 48.81 | 49.54 | 49.69 | -0.73 |
| 3433 | 2,4-Dimethyl-3-pentanone | 34.13 | 34.86 | 34.89 | -0.73 |
| 3434 | 3-Allylpiperidine | 39.38 | 40.11 | 40.12 | -0.73 |
| 3435 | 2-Hexyne | 28.05 | 28.78 | 28.76 | -0.73 |
| 3436 | Isoprene | 25.49 | 24.76 | 24.77 | 0.73 |
| 3437 | Cyclopropanecarboxylic acid | 20.80 | 20.07 | 20.07 | 0.73 |
| 3438 | Di-n-octylamine | 80.15 | 79.42 | 79.43 | 0.73 |
| 3439 | Palmitic acid | 78.39 | 77.66 | 77.66 | 0.73 |
| 3440 | 3-Methyl-4-heptanol | 39.66 | 40.40 | 40.39 | -0.74 |
| 3441 | Allyl trans-cinnamate | 55.48 | 56.22 | 56.19 | -0.74 |
| 3442 | Benzyl acrylate | 46.20 | 46.94 | 46.91 | -0.74 |
| 3443 | 4-Allylphenol | 41.52 | 42.26 | 42.27 | -0.74 |
| 3444 | 3-Methyl-1,3-pentadiene | 30.36 | 29.62 | 29.60 | 0.74 |
| 3445 | 4-Pentenal | 24.94 | 25.68 | 25.74 | -0.74 |
| 3446 | Pentanamide | 29.20 | 28.46 | 28.46 | 0.74 |
| 3447 | Valerophenone | 49.68 | 50.42 | 50.42 | -0.74 |
| 3448 | 2-Cyclopenten-1-one | 22.86 | 23.60 | 23.62 | -0.74 |
| 3449 | 3-(Diethylamino)-1-propanol | 40.52 | 39.78 | 39.82 | 0.74 |
| 3450 | 4-Chlorobenzenemethanethiol | 44.50 | 43.76 | 43.88 | 0.74 |
| 3451 | 8-Chloroquinoline | 45.97 | 46.71 | 46.79 | -0.74 |
| 3452 | Ethyl cyanoformate | 22.99 | 22.25 | 22.25 | 0.74 |
| 3453 | Isoquinoline | 41.31 | 42.05 | 42.11 | -0.74 |
| 3454 | Phenylacetyl chloride | 41.04 | 41.78 | 41.77 | -0.74 |
| 3455 | Cetyl palmitate | 152.50 | 151.76 | 151.77 | 0.74 |
| 3456 | (trans-1-Butenyl)benzene | 46.13 | 45.38 | 45.35 | 0.75 |
| 3457 | 1,3-Difluorobenzene | 25.85 | 26.60 | 26.64 | -0.75 |
| 3458 | 1-Bromo-3-vinylbenzene | 44.14 | 43.39 | 43.33 | 0.75 |
| 3459 | 1-Penten-4-yne | 22.31 | 23.06 | 23.03 | -0.75 |
| 3460 | 2-Chloro-2-methylpropanal | 25.39 | 26.14 | 26.27 | -0.75 |
| 3461 | 3-(Trimethylsilyl)-1-propanol | 41.55 | 40.80 | 40.76 | 0.75 |
| 3462 | 5-Methyl-2(5H)-furanone | 24.17 | 24.92 | 24.89 | -0.75 |
| 3463 | 1-Bromo-2-ethoxyethane | 29.38 | 30.13 | 30.15 | -0.75 |
| 3464 | N,N-Dipropylaniline | 59.88 | 59.13 | 59.02 | 0.75 |
| 3465 | Benzeneethanamine | 38.77 | 39.52 | 39.53 | -0.75 |
| 3466 | Pinane | 44.76 | 44.01 | 44.00 | 0.75 |
| 3467 | N-Methyl-2-furanmethanamine | 31.52 | 32.27 | 32.28 | -0.75 |
| 3468 | Chlorthion | 67.58 | 66.82 | 66.84 | 0.76 |
| 3469 | Laurocapram | 86.32 | 87.08 | 87.12 | -0.76 |
| 3470 | Methylenebis(4-cyclohexylisocyanate) | 72.02 | 71.26 | 71.02 | 0.76 |
| 3471 | Tetratriacontane | 159.96 | 159.20 | 159.20 | 0.76 |
| 3472 | 1,2-Dichloro-3,3,4,4,5,5-hexafluorocyclopentene | 33.29 | 34.05 | 33.82 | -0.76 |
| 3473 | 1-Phenylallyl alcohol | 41.11 | 41.87 | 41.85 | -0.76 |
| 3474 | 2,2-Dimethyl-3-ethylpentane | 42.93 | 43.69 | 43.68 | -0.76 |
| 3475 | 2,6-Dimethylanisole | 42.02 | 42.78 | 42.80 | -0.76 |
| 3476 | 2-Isopropylphenol | 41.67 | 42.43 | 42.45 | -0.76 |
| 3477 | 2-Methyl-2-oxazoline | 22.05 | 22.81 | 22.95 | -0.76 |
| 3478 | 2-Phenylpropionitrile | 39.78 | 40.54 | 40.50 | -0.76 |
| 3479 | trans-3-Phenyl-2-propen-1-ol | 42.89 | 42.13 | 42.09 | 0.76 |
| 3480 | cis-3-Phenyl-2-propen-1-ol | 42.89 | 42.13 | 42.12 | 0.76 |
| 3481 | Ethylisopropylether | 27.68 | 26.92 | 26.94 | 0.76 |
| 3482 | Heptaldoxime | 38.17 | 38.93 | 39.09 | -0.76 |
| 3483 | Isopropyl (2,4-dichlorophenoxy)acetate | 63.58 | 62.82 | 62.74 | 0.76 |
| 3484 | 1,1-Dibromoethane | 27.46 | 26.70 | 26.65 | 0.76 |
| 3485 | Benzo[c]isoxazole | 33.73 | 32.97 | 32.66 | 0.76 |
| 3486 | Diethyl ethylphenylmalonate | 71.30 | 72.06 | 72.08 | -0.76 |
| 3487 | Exaltolide | 70.34 | 71.10 | 71.08 | -0.76 |
| 3488 | 4-Iodo-1,2-butadiene | 34.52 | 33.76 | 33.68 | 0.76 |
| 3489 | 3,4-Dihydro-1H-2-benzopyran | 39.72 | 40.48 | 40.42 | -0.76 |
| 3490 | 3,3'-Dimethylbiphenyl | 61.93 | 61.16 | 61.12 | 0.77 |
| 3491 | 3-Cyclohexene-1-carboxylic acid | 33.20 | 33.97 | 33.94 | -0.77 |
| 3492 | alpha-Chlorobenzeneacetyl chloride | 45.80 | 46.57 | 46.57 | -0.77 |
| 3493 | 6-Undecanone | 52.63 | 53.40 | 53.43 | -0.77 |
| 3494 | Ethyl 5-bromopentanoate | 43.30 | 44.07 | 44.08 | -0.77 |
| 3495 | 1,1-Dichloropropene | 24.80 | 25.57 | 25.63 | -0.77 |
| 3496 | 1-Hydroxy-2-butanone | 21.66 | 22.43 | 22.40 | -0.77 |
| 3497 | 1,2-Dinitroethane | 21.97 | 22.74 | 22.96 | -0.77 |
| 3498 | 1,2-Dis(trimethylsilyl)-ethylene | 58.81 | 58.04 | 57.84 | 0.77 |
| 3499 | 1-(Chloromethyl)-4-methylbenzene | 41.84 | 41.07 | 41.08 | 0.77 |
| 3500 | 1-Methoxy-2-propanol | 22.88 | 23.65 | 23.66 | -0.77 |
| 3501 | 2-Butyl-2-ethyl-1,3-propanediol | 47.23 | 46.46 | 46.55 | 0.77 |
| 3502 | 2-Methylnaphthalene | 48.47 | 49.24 | 49.27 | -0.77 |
| 3503 | Benzylcarbinol isobutyrate | 55.58 | 56.35 | 56.32 | -0.77 |
| 3504 | Bicyclo[2.2.1]hept-5-ene-2-carbaldehyde | 34.65 | 35.42 | 35.43 | -0.77 |
| 3505 | Pivaldehyde | 25.13 | 25.90 | 25.91 | -0.77 |
| 3506 | alpha-Methylbenzenepropanol | 45.91 | 46.69 | 46.75 | -0.78 |
| 3507 | Pentanedioyl dichloride | 35.80 | 36.58 | 36.64 | -0.78 |
| 3508 | 1,1-Dichloro-2,2-difluoroethane | 20.24 | 21.02 | 21.15 | -0.78 |
| 3509 | 2-Methyl-1,3-cyclohexadiene | 31.58 | 32.36 | 32.36 | -0.78 |
| 3510 | 1-Methyl-2-benzylbenzene | 60.22 | 61.00 | 61.08 | -0.78 |
| 3511 | 5-Methyl-2-furanmethanol | 29.86 | 30.64 | 30.40 | -0.78 |
| 3512 | Butyl 2-hydroxybenzoate | 54.28 | 53.50 | 53.50 | 0.78 |
| 3513 | Ethylcyclopropane | 23.85 | 23.07 | 23.08 | 0.78 |
| 3514 | Methyl 3-cyclohexene-1-carboxylate | 37.97 | 38.75 | 38.76 | -0.78 |
| 3515 | N-Nitroso-N-methylaniline | 40.14 | 39.36 | 37.86 | 0.78 |
| 3516 | Pyrazine | 22.66 | 21.88 | 21.80 | 0.78 |
| 3517 | Triethylborane | 33.72 | 34.50 |  | -0.78 |
| 3518 | Caryophyllene | 66.07 | 66.86 | 66.95 | -0.79 |
| 3519 | 1-Methyl-1-phenylhydrazine | 38.48 | 39.27 | 39.75 | -0.79 |
| 3520 | 1,4-Difluorobenzene | 25.81 | 26.60 | 26.70 | -0.79 |
| 3521 | 2-Methylfuran | 23.42 | 24.21 | 24.24 | -0.79 |
| 3522 | 4,4-Dimethylcyclohexanone | 36.65 | 37.44 | 37.43 | -0.79 |
| 3523 | Cyclopropanecarbonitrile | 19.09 | 18.30 | 18.30 | 0.79 |
| 3524 | (Ethoxymethyl)oxirane | 27.31 | 26.52 | 26.48 | 0.79 |
| 3525 | 3,7-Dimethyl-6-octen-3-ol | 48.94 | 49.73 | 49.80 | -0.79 |
| 3526 | 1-Bromo-3-methylcyclohexane | 40.94 | 40.14 | 40.14 | 0.80 |
| 3527 | 2-Phenylethyl propanoate | 50.96 | 51.76 | 51.75 | -0.80 |
| 3528 | 4-Fluoro-2-methylaniline | 34.66 | 35.46 | 35.57 | -0.80 |
| 3529 | Bis(2-mercaptoethyl) sulfide | 44.51 | 43.71 | 43.71 | 0.80 |
| 3530 | Butyl thiocyanate | 31.50 | 32.30 | 32.58 | -0.80 |
| 3531 | Dichlorophenylphosphine | 45.34 | 46.14 |  | -0.80 |
| 3532 | Pseudopelletierine | 43.17 | 43.97 | 44.02 | -0.80 |
| 3533 | 1-Fluoro-2-(trifluoromethyl)benzene | 31.04 | 31.84 | 31.82 | -0.80 |
| 3534 | Decanoyl chloride | 54.80 | 54.00 | 53.98 | 0.80 |
| 3535 | Diethyl fumarate | 43.52 | 42.72 | 42.68 | 0.80 |
| 3536 | Butopyroxonyl | 60.27 | 59.47 | 59.43 | 0.80 |
| 3537 | Sorbic alcohol | 32.09 | 31.29 | 31.31 | 0.80 |
| 3538 | 1,3-Diaminobenzene | 33.95 | 34.76 | 34.94 | -0.81 |
| 3539 | 4-Isopropylbenzaldehyde | 46.94 | 46.13 | 46.12 | 0.81 |
| 3540 | Diethyl cyclopropane-1,1-dicarboxylate | 46.01 | 45.20 | 45.15 | 0.81 |
| 3541 | Heptanoamide | 38.51 | 37.70 | 37.69 | 0.81 |
| 3542 | 1-Ethyl-1-methylcyclopropane | 28.55 | 27.74 | 27.74 | 0.81 |
| 3543 | 2-Fluorotoluene | 30.62 | 31.43 | 31.45 | -0.81 |
| 3544 | Bis(trifluoroacetyl)methane (enol form) | 28.84 | 28.03 | 27.95 | 0.81 |
| 3545 | Carbonyl dicyanide | 16.95 | 16.14 | 16.11 | 0.81 |
| 3546 | 1-Benzylpiperidine | 55.60 | 56.41 | 56.43 | -0.81 |
| 3547 | 4-Vinylcyclohexene | 35.96 | 36.77 | 36.75 | -0.81 |
| 3548 | 5-Methyl-1-phenyl-1-hexen-3-one | 60.48 | 59.67 | 59.67 | 0.81 |
| 3549 | cis-3,7-Dimethyl-2,6-octadien-1-ol acetate | 58.51 | 59.32 | 59.33 | -0.81 |
| 3550 | Methyl cyclohexanecarboxylate | 37.90 | 38.71 | 38.72 | -0.81 |
| 3551 | 1,1,1-Trifluoro-2-iodoethane | 23.94 | 24.76 | 24.80 | -0.82 |
| 3552 | 1,2-Cyclohexanedione | 29.46 | 28.64 | 28.64 | 0.82 |
| 3553 | 1-Chloro-2,4-dinitrobenzene | 45.36 | 44.54 | 44.61 | 0.82 |
| 3554 | 2-Nitroacetophenone | 42.33 | 43.15 | 43.08 | -0.82 |
| 3555 | 2-Thiophenecarbonitrile | 30.24 | 29.42 | 29.36 | 0.82 |
| 3556 | 4,4-Dimethyl-1-pentene | 33.46 | 34.28 | 34.29 | -0.82 |
| 3557 | Benzyl 3-methylbutanoate | 55.53 | 56.35 | 56.31 | -0.82 |
| 3558 | Crotonic acid anhydride | 41.71 | 40.89 | 40.84 | 0.82 |
| 3559 | Diethyl acetylenedicarboxylate | 42.44 | 41.62 | 41.68 | 0.82 |
| 3560 | Glyoxal | 11.86 | 12.68 | 12.78 | -0.82 |
| 3561 | (2-Chloro-1,1-dimethylethyl)benzene | 49.35 | 50.17 | 50.15 | -0.82 |
| 3562 | Paramethadione | 37.61 | 38.43 | 38.76 | -0.82 |
| 3563 | Chloro-2,3-dinitrobenzene | 45.36 | 44.54 | 44.46 | 0.82 |
| 3564 | 1,2,3,4-Tetrachlorobutadiene | 40.04 | 39.22 | 39.02 | 0.82 |
| 3565 | Iophendylate | 102.07 | 101.25 | 101.21 | 0.82 |
| 3566 | cis-3-Hexenal | 29.71 | 30.54 | 30.57 | -0.83 |
| 3567 | Ethylene glycol monostearate | 96.86 | 97.69 | 97.66 | -0.83 |
| 3568 | gamma-Terpinene | 45.30 | 46.13 | 46.13 | -0.83 |
| 3569 | 2-Bromo-1,1,1-trifluoroethane | 18.75 | 19.58 | 19.59 | -0.83 |
| 3570 | 3-Chlorobutanoic acid | 26.18 | 27.01 | 27.01 | -0.83 |
| 3571 | Dicyanoacetylene | 18.41 | 19.24 | 19.38 | -0.83 |
| 3572 | 3-Chlorobiphenyl | 57.09 | 56.26 | 56.25 | 0.83 |
| 3573 | 3,7-Dimethyl-1,3,7-octatriene | 48.91 | 48.08 | 48.06 | 0.83 |
| 3574 | Tris(2-chloroethyl) phosphate | 57.52 | 56.69 | 56.61 | 0.83 |
| 3575 | Tetracontane | 187.76 | 186.92 | 186.92 | 0.84 |
| 3576 | Isophytol | 94.82 | 95.66 | 95.77 | -0.84 |
| 3577 | Pentaerythritol tetrakis(2-mercaptoacetate) | 99.04 | 99.88 | 100.20 | -0.84 |
| 3578 | 1H-Pentadecafluoroheptane | 36.27 | 37.11 | 37.38 | -0.84 |
| 3579 | Isopentyl salicylate | 58.93 | 58.09 | 58.09 | 0.84 |
| 3580 | 1-Chloro-1,3-butadiene | 25.77 | 24.93 | 24.96 | 0.84 |
| 3581 | Cyanoacetylene | 14.71 | 13.87 | 13.86 | 0.84 |
| 3582 | Methyl vinyl ketone | 20.02 | 20.86 | 20.90 | -0.84 |
| 3583 | 2,5-Dihydro-1H-pyrrole | 21.06 | 21.90 | 21.90 | -0.84 |
| 3584 | Thiepane | 34.75 | 35.59 | 35.65 | -0.84 |
| 3585 | 2-Bromo-4-phenyl-1-butene | 51.71 | 52.55 | 52.44 | -0.84 |
| 3586 | (Chloromethyl)cyclopropane | 24.11 | 23.26 | 23.26 | 0.85 |
| 3587 | 1-Bromo-1-chloroethane | 23.83 | 24.68 | 24.84 | -0.85 |
| 3588 | 3-Furancarboxaldehyde | 25.22 | 24.37 | 24.35 | 0.85 |
| 3589 | 1,1-Dibromopropane | 30.47 | 31.32 | 31.40 | -0.85 |
| 3590 | 1,3-Bis(1-methylethenyl)benzene | 55.07 | 54.22 | 54.24 | 0.85 |
| 3591 | 2-Bromo-4-methylaniline | 42.15 | 43.00 | 43.01 | -0.85 |
| 3592 | Anatabine | 48.02 | 48.87 | 48.89 | -0.85 |
| 3593 | Bis(2-methallyl) carbonate | 47.30 | 46.45 | 46.28 | 0.85 |
| 3594 | Isopulegol acetate | 57.75 | 56.90 | 56.86 | 0.85 |
| 3595 | N-Cyclohexylaniline | 55.89 | 56.74 | 56.94 | -0.85 |
| 3596 | N-Ethylbenzaldehydeimine | 44.45 | 43.60 | 43.21 | 0.85 |
| 3597 | Pentanoyl chloride | 30.05 | 30.90 | 30.90 | -0.85 |
| 3598 | Arachidic acid | 96.99 | 96.14 | 96.14 | 0.85 |
| 3599 | Aspon | 94.44 | 95.29 | 95.63 | -0.85 |
| 3600 | 2-Ethylpyridine | 32.97 | 33.83 | 33.86 | -0.86 |
| 3601 | alpha-Santalol | 67.22 | 66.36 | 66.32 | 0.86 |
| 3602 | Trimethyl trimellitate | 61.10 | 60.24 | 60.24 | 0.86 |
| 3603 | Diethyl chloromalonate | 41.99 | 42.85 | 42.85 | -0.86 |
| 3604 | Dipropylacetamide | 42.14 | 43.00 | 43.11 | -0.86 |
| 3605 | Bolecic acid | 85.44 | 84.58 | 84.49 | 0.86 |
| 3606 | Mesoxalic acid diethylester oxime | 43.37 | 42.51 | 42.35 | 0.86 |
| 3607 | Pentyltrichlorosilane | 48.79 | 47.93 | 47.92 | 0.86 |
| 3608 | Quinoxaline | 40.50 | 39.64 | 39.34 | 0.86 |
| 3609 | Trimethylamine | 20.97 | 20.11 | 20.11 | 0.86 |
| 3610 | Cryofluorane | 22.58 | 21.72 | 21.34 | 0.86 |
| 3611 | 1,4-Dimethyl-2-nitrobenzene | 41.98 | 42.85 | 42.89 | -0.87 |
| 3612 | Chloroethyldimethylsilane | 35.07 | 34.20 | 34.11 | 0.87 |
| 3613 | Octyl nitrite | 46.03 | 45.16 | 45.03 | 0.87 |
| 3614 | Piperitenone | 47.43 | 46.56 | 46.70 | 0.87 |
| 3615 | Bromoacetone | 23.38 | 24.25 | 24.29 | -0.87 |
| 3616 | Dimethyl oxalate | 23.29 | 24.16 | 24.18 | -0.87 |
| 3617 | Isopropylcyclopropane | 28.53 | 27.66 | 27.66 | 0.87 |
| 3618 | t-Butyl isocyanate | 28.09 | 27.22 | 27.14 | 0.87 |
| 3619 | 2-Phenyl-1-butene | 45.95 | 45.08 | 45.06 | 0.87 |
| 3620 | 4-s-Butylphenol | 46.18 | 47.05 | 47.07 | -0.87 |
| 3621 | Muscone | 73.48 | 74.35 | 74.39 | -0.87 |
| 3622 | Petroselinic acid | 87.81 | 86.94 | 86.94 | 0.87 |
| 3623 | (trans-2-Bromovinyl)benzene | 44.44 | 43.56 | 43.50 | 0.88 |
| 3624 | 2-Iodonaphthalene | 57.90 | 57.02 | 56.97 | 0.88 |
| 3625 | cis-Benzaldehyde oxime | 36.83 | 35.95 | 35.80 | 0.88 |
| 3626 | cis-2-Methoxy-4-(1-propenyl)phenol | 49.90 | 49.02 | 48.97 | 0.88 |
| 3627 | Heptanoic anhydride | 67.65 | 68.53 | 68.58 | -0.88 |
| 3628 | Hexacosane | 123.12 | 122.24 | 122.24 | 0.88 |
| 3629 | n-Heptylnitrite | 39.66 | 40.54 | 40.63 | -0.88 |
| 3630 | 1-Phenoxy-2-propanone | 42.06 | 42.94 | 42.93 | -0.88 |
| 3631 | Phenylacetaldehyde | 35.88 | 36.76 | 36.74 | -0.88 |
| 3632 | Phenylisothiocyanate | 43.58 | 42.70 | 42.74 | 0.88 |
| 3633 | 3,4-Dichloro-1-butene | 30.88 | 30.00 | 29.96 | 0.88 |
| 3634 | 3-Bromo-4,5-dihydro-2(3H)-furanone | 27.23 | 28.11 | 28.09 | -0.88 |
| 3635 | Perfluorohexane | 31.58 | 32.46 | 32.62 | -0.88 |
| 3636 | alpha-Phellandrene | 45.28 | 46.16 | 46.17 | -0.88 |
| 3637 | Methoxyacetylene | 16.22 | 15.33 | 15.10 | 0.89 |
| 3638 | Bis(2-ethylhexyl) azelate | 120.27 | 121.16 | 121.31 | -0.89 |
| 3639 | Methylcyclobutane | 23.96 | 23.07 | 23.07 | 0.89 |
| 3640 | Suberonitrile | 37.89 | 38.78 | 38.74 | -0.89 |
| 3641 | Pivalic acid chloride | 30.03 | 30.92 | 30.93 | -0.89 |
| 3642 | 1-Fluoro-4-(trifluoromethyl)benzene | 30.94 | 31.84 | 31.87 | -0.90 |
| 3643 | 2-Butyne | 18.64 | 19.54 | 19.60 | -0.90 |
| 3644 | 4-Methyl-1-heptanol | 41.32 | 40.42 | 40.42 | 0.90 |
| 3645 | Cyclononanone | 41.14 | 42.04 | 42.09 | -0.90 |
| 3646 | (Bromomethyl)oxirane | 24.27 | 23.37 | 23.34 | 0.90 |
| 3647 | Pentamethylbenzene | 49.70 | 50.60 | 50.65 | -0.90 |
| 3648 | 2,5-Dihydro-2,5-dimethoxyfuran | 31.58 | 32.48 | 32.48 | -0.90 |
| 3649 | Acetonylacetone | 29.86 | 30.76 | 30.80 | -0.90 |
| 3650 | 2-Bromopropanoyl chloride | 28.59 | 29.49 | 29.54 | -0.90 |
| 3651 | trans-1,2-Dimethylcyclopropane | 23.94 | 23.04 | 23.06 | 0.90 |
| 3652 | 1,2,3,4-Tetrahydro-1-naphthol | 43.83 | 44.73 | 44.77 | -0.90 |
| 3653 | Isobutyl propanoate | 35.33 | 36.23 | 36.24 | -0.90 |
| 3654 | Propyl isobutanoate | 35.33 | 36.23 | 36.22 | -0.90 |
| 3655 | Octadecane | 86.18 | 85.28 | 85.28 | 0.90 |
| 3656 | Propyl 3-(2-furyl)acrylate | 50.05 | 49.15 | 49.14 | 0.90 |
| 3657 | Sulfotep | 75.91 | 76.81 | 77.17 | -0.90 |
| 3658 | 1,1-Diphenylbutane | 69.14 | 70.05 | 70.03 | -0.91 |
| 3659 | 1-(4-Methylphenyl)ethanol | 43.14 | 42.23 | 42.24 | 0.91 |
| 3660 | 2-Hydroxy-5-methylbenzaldehyde | 39.57 | 38.66 | 38.61 | 0.91 |
| 3661 | 1-Chloro-4-nitro-2-(trifluoromethyl)benzene | 44.05 | 43.14 | 43.08 | 0.91 |
| 3662 | 2-Hydroxybenzoyl chloride | 39.81 | 38.90 | 38.89 | 0.91 |
| 3663 | N-Methylformanilide | 39.86 | 40.77 | 40.93 | -0.91 |
| 3664 | Tripalmitin | 242.20 | 243.12 | 243.09 | -0.92 |
| 3665 | Ethyl 4-methylcaproate | 44.55 | 45.47 | 45.48 | -0.92 |
| 3666 | 2-Propyn-1-amine | 18.36 | 17.44 | 17.40 | 0.92 |
| 3667 | 4-Pentylphenol | 52.62 | 51.70 | 51.71 | 0.92 |
| 3668 | Dodecanoic anhydride | 115.65 | 114.73 | 114.70 | 0.92 |
| 3669 | Ethyl (4-methoxyphenyl)acetate | 52.74 | 53.66 | 53.65 | -0.92 |
| 3670 | 1,3-Cyclohexadiene | 26.87 | 27.80 | 27.80 | -0.93 |
| 3671 | 2-Allylphenol | 41.33 | 42.26 | 42.23 | -0.93 |
| 3672 | alpha-Methylcyclopropanemethanol | 25.35 | 24.42 | 24.41 | 0.93 |
| 3673 | Ethyl benzoylacetate | 53.15 | 52.22 | 52.25 | 0.93 |
| 3674 | Ethyl N-phenylformimidate | 45.70 | 44.77 |  | 0.93 |
| 3675 | Ethyl 3-aminobenzoate | 45.62 | 46.55 | 46.58 | -0.93 |
| 3676 | 1,1,3-Trimethylcyclohexane | 42.51 | 41.57 | 41.62 | 0.94 |
| 3677 | Isoquinaldine | 46.03 | 46.97 | 46.90 | -0.94 |
| 3678 | 3-(Trifluoromethyl)phenyl isocyanate | 38.73 | 39.67 | 40.39 | -0.94 |
| 3679 | 3-Trifluoromethylaniline | 34.98 | 35.92 | 35.87 | -0.94 |
| 3680 | 4-Chlorobenzaldehyde | 37.74 | 36.80 | 36.74 | 0.94 |
| 3681 | Acetoacetic acid ethylester Enolform | 34.11 | 33.17 | 33.09 | 0.94 |
| 3682 | cis,cis-2,4-Hexadiene | 30.86 | 29.92 | 29.88 | 0.94 |
| 3683 | Urea | 12.99 | 12.05 |  | 0.94 |
| 3684 | Cyclopentylamine | 27.48 | 26.54 | 26.50 | 0.94 |
| 3685 | Glycidyl methacrylate | 36.52 | 35.58 | 35.59 | 0.94 |
| 3686 | 4-Acetylanisole | 44.02 | 43.08 | 43.14 | 0.94 |
| 3687 | 1-Methyl-4-benzylbenzene | 60.05 | 61.00 | 61.08 | -0.95 |
| 3688 | 2-Methyl-2-pentanol | 30.32 | 31.27 | 31.30 | -0.95 |
| 3689 | Tetrahydrothiophene | 25.40 | 26.35 | 26.41 | -0.95 |
| 3690 | 1,3-Diphenylpropane | 64.51 | 65.46 | 65.46 | -0.95 |
| 3691 | 6-Vinyl-6-methyl-1-isopropyl-3-(1-methylethylidene)cyclohexene | 69.94 | 68.99 | 68.98 | 0.95 |
| 3692 | 2,5-Dimethylfuran | 28.31 | 29.27 | 29.43 | -0.96 |
| 3693 | 2-Chloro-1-trifluoromethylbenzene | 35.59 | 36.55 | 36.52 | -0.96 |
| 3694 | 2-Methyl-5-phenylpyridine | 55.07 | 54.11 | 54.16 | 0.96 |
| 3695 | Glycolaldehyde | 12.43 | 13.39 | 13.48 | -0.96 |
| 3696 | Diethyl ketomalonate | 39.48 | 38.52 | 38.49 | 0.96 |
| 3697 | Monomethyl adipate | 38.82 | 37.86 | 37.86 | 0.96 |
| 3698 | 8-Methyl-8-azabicyclo[3.2.1]octan-3-one | 38.39 | 39.35 | 39.36 | -0.96 |
| 3699 | 4-Methyl-2-nitrophenol | 40.78 | 39.81 | 39.90 | 0.97 |
| 3700 | alpha-Allylbenzenemethanol | 45.52 | 46.49 | 46.49 | -0.97 |
| 3701 | Nopinene | 44.75 | 43.78 | 43.82 | 0.97 |
| 3702 | Diethyl ethylidenemalonate | 46.31 | 47.28 | 47.30 | -0.97 |
| 3703 | Dimethylaminoacetaldehyde diethylacetal | 45.42 | 46.39 | 46.42 | -0.97 |
| 3704 | 1-Docosanoic acid | 106.35 | 105.38 | 105.39 | 0.97 |
| 3705 | 2,6-Dimethylcyclohexanone | 36.38 | 37.36 | 37.36 | -0.98 |
| 3706 | Benzyl acetate | 43.50 | 42.52 | 42.52 | 0.98 |
| 3707 | cis-Stilbene | 61.86 | 60.88 | 60.88 | 0.98 |
| 3708 | Cyclohexylisopropylamine | 44.02 | 45.00 | 45.02 | -0.98 |
| 3709 | Lauric acid | 58.20 | 59.18 | 59.15 | -0.98 |
| 3710 | 1,1,2-Trimethylcyclopropane | 28.69 | 27.71 | 27.70 | 0.98 |
| 3711 | cis-1,3,5-Hexatriene | 30.46 | 29.48 | 29.44 | 0.98 |
| 3712 | cis-1,3-Hexadiene | 30.66 | 29.68 | 29.69 | 0.98 |
| 3713 | Aramite | 87.62 | 86.64 | 86.51 | 0.98 |
| 3714 | 3,4-Dihydro-2H-1-benzopyran | 39.54 | 40.52 | 40.53 | -0.98 |
| 3715 | 4-Fluorobenzeneacetonitrile | 34.91 | 35.90 | 35.90 | -0.99 |
| 3716 | beta-Irone | 66.08 | 65.09 | 65.11 | 0.99 |
| 3717 | Paraoxon | 64.85 | 63.86 | 63.71 | 0.99 |
| 3718 | 3-Chlorocrotonic acid | 28.06 | 27.07 | 27.09 | 0.99 |
| 3719 | 1,2-Dimethyl-3-nitrobenzene | 41.86 | 42.85 | 42.94 | -0.99 |
| 3720 | 2,5-Dichlorostyrene | 46.21 | 45.22 | 45.17 | 0.99 |
| 3721 | Diphenylmethylamine | 58.67 | 59.66 | 59.60 | -0.99 |
| 3722 | 2,3-Dihydro-1,4-benzodioxin | 36.67 | 37.66 | 37.66 | -0.99 |
| 3723 | Phenylacetonitrile | 34.96 | 35.95 | 35.94 | -0.99 |
| 3724 | Furan | 18.16 | 19.15 | 19.21 | -0.99 |
| 3725 | (4-Chlorophenyl)trichlorosilane | 55.04 | 54.04 | 53.85 | 1.00 |
| 3726 | 2-Phenoxypropanoyl chloride | 47.14 | 48.14 | 48.14 | -1.00 |
| 3727 | 4-Methyl-2-heptanol | 41.40 | 40.40 | 40.40 | 1.00 |
| 3728 | 4-Nonanol | 44.05 | 45.05 | 45.05 | -1.00 |
| 3729 | Diallyl fumarate | 50.56 | 51.56 | 51.56 | -1.00 |
| 3730 | trans-1-Propenylbenzene | 41.76 | 40.76 | 40.79 | 1.00 |
| 3731 | 2,4,6-Cycloheptatrien-1-one | 33.92 | 32.92 | 32.98 | 1.00 |
| 3732 | Pentyl nonanoate | 69.61 | 68.60 | 68.58 | 1.01 |
| 3733 | 2-Methyl-1-heptanol | 41.43 | 40.42 | 40.46 | 1.01 |
| 3734 | Pempidine | 49.10 | 50.11 | 50.34 | -1.01 |
| 3735 | 1,1-Dichlorotetrafluoroethane | 22.58 | 21.57 | 21.40 | 1.01 |
| 3736 | Ethyl thiocyanate | 24.07 | 23.06 |  | 1.01 |
| 3737 | 2-Chloro-N,N-diallylacetamide | 46.40 | 47.41 | 47.36 | -1.01 |
| 3738 | N-Butyl-N-phenylacetamide | 58.15 | 59.16 | 59.33 | -1.01 |
| 3739 | 3,5-Bis(trifluoromethyl)aniline | 40.09 | 41.11 | 41.26 | -1.02 |
| 3740 | N,N-Diethylcarbamic acid | 32.00 | 30.98 | 30.98 | 1.02 |
| 3741 | Ethyl 4-methoxybenzoate | 50.06 | 49.04 | 49.00 | 1.02 |
| 3742 | Isobutyl salicylate | 54.49 | 53.47 | 53.47 | 1.02 |
| 3743 | N-Ethyl-3-methylaniline | 46.16 | 45.14 | 45.14 | 1.02 |
| 3744 | Hexadecyl acetate | 88.10 | 87.08 | 87.09 | 1.02 |
| 3745 | 12-Tricosanone | 107.81 | 108.84 | 108.84 | -1.03 |
| 3746 | 2-Iodobiphenyl | 65.19 | 64.16 | 64.05 | 1.03 |
| 3747 | 4(10)-Thujene-3-ol | 46.16 | 45.13 | 45.14 | 1.03 |
| 3748 | Benzyl laurate | 87.69 | 88.72 | 88.71 | -1.03 |
| 3749 | Diethyl thiodipropionate | 58.76 | 59.79 | 59.77 | -1.03 |
| 3750 | Ethyl oenanthate | 44.47 | 45.50 | 45.56 | -1.03 |
| 3751 | Ethyl salicylate | 45.29 | 44.26 | 44.23 | 1.03 |
| 3752 | Tetradecanoic anhydride | 134.25 | 133.21 | 133.26 | 1.04 |
| 3753 | 3-Cyclohexene-1-carboxaldehyde | 31.97 | 33.01 | 33.02 | -1.04 |
| 3754 | 1-Cyclohexene-1-carboxylic acid | 32.90 | 33.94 | 33.94 | -1.04 |
| 3755 | 4-Chlorostyrene | 41.60 | 40.56 | 40.54 | 1.04 |
| 3756 | 4-Oxopentanal | 25.30 | 26.34 | 26.33 | -1.04 |
| 3757 | 1,1,1,2,2,3,3-Heptafluorobutane | 22.95 | 21.91 | 21.93 | 1.04 |
| 3758 | Nornicotine | 43.16 | 44.21 | 44.18 | -1.05 |
| 3759 | 1-Chloro-3-(1-methylethoxy)-2-propanol | 36.65 | 37.70 | 37.68 | -1.05 |
| 3760 | 2-Fluoroanisole | 32.12 | 33.17 | 33.16 | -1.05 |
| 3761 | 2-Propylcyclohexanone | 40.95 | 42.01 | 42.00 | -1.06 |
| 3762 | 4-Methoxy-1,1'-biphenyl | 59.18 | 58.12 | 58.08 | 1.06 |
| 3763 | trans-5-(1-Propenyl)-1,3-benzodioxole | 47.97 | 46.91 | 47.03 | 1.06 |
| 3764 | 2-Cyclohexen-1-ol | 28.05 | 29.11 | 29.12 | -1.06 |
| 3765 | 2-Thiophenecarboxaldehyde | 31.29 | 30.23 | 30.12 | 1.06 |
| 3766 | cis-2-Methyl-1,3-pentadiene | 30.68 | 29.62 | 29.60 | 1.06 |
| 3767 | 2-Bromo-2-chloro-1,1,1-trifluoroethane | 24.03 | 25.09 |  | -1.06 |
| 3768 | N,N-Dimethyltrichloroacetamide | 40.42 | 39.36 | 39.36 | 1.06 |
| 3769 | 2-Phenylpyridine | 50.39 | 49.33 | 49.33 | 1.06 |
| 3770 | 3,3,5-Trimethylheptane | 49.37 | 48.31 | 48.31 | 1.06 |
| 3771 | 4-Isopropylstyrene | 50.95 | 49.89 | 49.92 | 1.06 |
| 3772 | Divinylacetylene | 29.44 | 28.38 | 28.42 | 1.06 |
| 3773 | 1-Bromo-4-vinylbenzene | 44.46 | 43.39 | 43.42 | 1.07 |
| 3774 | 1-Nonene | 44.57 | 43.50 | 43.50 | 1.07 |
| 3775 | 4-Bromoanisole | 41.78 | 40.71 | 40.71 | 1.07 |
| 3776 | 1-Methyl-4-(5-methyl-1-methylene-4-hexenyl)cyclohexene | 67.89 | 68.97 | 68.98 | -1.08 |
| 3777 | alpha-Methylbenzeneacetaldehyde | 40.27 | 41.35 | 41.41 | -1.08 |
| 3778 | Cyclododecanone | 54.82 | 55.90 | 55.89 | -1.08 |
| 3779 | Isopropyl salicylate | 49.94 | 48.86 | 48.89 | 1.08 |
| 3780 | alpha-Curcumene | 67.46 | 68.55 | 68.62 | -1.09 |
| 3781 | 1-Methyl-1,3-cyclopentadiene | 26.65 | 27.74 | 27.74 | -1.09 |
| 3782 | 4-Hydroxy-2-butanone | 23.52 | 22.43 | 22.41 | 1.09 |
| 3783 | 2-Formylnaphthalene | 50.99 | 49.90 | 49.92 | 1.09 |
| 3784 | 3,4-Dihydro-2(1H)-naphthalenone | 42.75 | 43.84 | 43.81 | -1.09 |
| 3785 | p-Isopropenylstyrene | 50.76 | 49.66 | 49.69 | 1.10 |
| 3786 | 2-Chloro-p-cresol | 36.78 | 37.88 | 37.89 | -1.10 |
| 3787 | 1-(2-Methylphenyl)ethanone | 40.24 | 41.34 | 41.35 | -1.10 |
| 3788 | 1,2-Dimethoxy-4-(1-propenyl)benzene | 54.91 | 53.80 | 53.81 | 1.11 |
| 3789 | 2-(Phenylmethoxy)phenol | 58.61 | 59.72 | 59.78 | -1.11 |
| 3790 | Tetracosanoic acid | 115.73 | 114.62 | 114.62 | 1.11 |
| 3791 | Phenothrin | 104.96 | 103.85 | 103.78 | 1.11 |
| 3792 | 3-Methylbutanoic anhydride | 48.87 | 49.99 | 49.87 | -1.12 |
| 3793 | Caproic aldehyde | 29.38 | 30.50 | 30.55 | -1.12 |
| 3794 | Diethyl sebacate | 69.28 | 70.40 | 70.38 | -1.12 |
| 3795 | Isopropyl chloroacetate | 32.94 | 31.81 | 31.78 | 1.13 |
| 3796 | Triphenylethylene | 86.69 | 85.56 | 85.56 | 1.13 |
| 3797 | 2-Methylpentane | 30.94 | 29.81 | 29.85 | 1.13 |
| 3798 | 3-Fluorophenol | 27.26 | 28.39 | 28.42 | -1.13 |
| 3799 | 2,2-Dimethylpropane | 26.37 | 25.24 | 25.25 | 1.13 |
| 3800 | Benzotrifluoride | 30.76 | 31.89 | 31.90 | -1.13 |
| 3801 | Hexyl propanoate | 46.63 | 45.50 | 45.49 | 1.13 |
| 3802 | Cyclohexylmercaptan | 34.48 | 35.62 | 35.72 | -1.14 |
| 3803 | 2,3-Dimethylhexane | 40.16 | 39.02 | 39.02 | 1.14 |
| 3804 | 4-Mercaptophenol | 35.10 | 36.24 | 36.47 | -1.14 |
| 3805 | N-Methyl-p-toluidine | 41.72 | 40.58 | 40.63 | 1.14 |
| 3806 | Vinyl ether | 21.93 | 20.79 | 20.69 | 1.14 |
| 3807 | Xibornol | 78.96 | 80.10 | 80.16 | -1.14 |
| 3808 | 1,2-Divinylbenzene | 46.25 | 45.10 | 45.10 | 1.15 |
| 3809 | Diethoxydimethylsilane | 39.81 | 40.96 | 41.14 | -1.15 |
| 3810 | 3-Methoxypropylamine | 26.87 | 25.72 | 25.74 | 1.15 |
| 3811 | trans,cis-2,4-Hexadiene | 31.08 | 29.92 | 29.90 | 1.16 |
| 3812 | 1,4-Dihydronaphthalene | 42.26 | 43.42 | 43.40 | -1.16 |
| 3813 | Benzyl isobutyrate | 50.57 | 51.73 | 51.70 | -1.16 |
| 3814 | 2-Chloropropanal | 20.26 | 21.43 | 21.45 | -1.17 |
| 3815 | trans-1,3-Pentadiene | 26.23 | 25.06 | 25.04 | 1.17 |
| 3816 | 1,2-Dichloro-1-ethoxyethane | 33.38 | 32.21 | 32.13 | 1.17 |
| 3817 | 2,5-Dimethyl-2,4-hexadiene | 40.21 | 39.04 | 39.04 | 1.17 |
| 3818 | 4,4'-Diphenylmethane diisocyanate | 70.61 | 71.78 | 73.14 | -1.17 |
| 3819 | Hexacosanoic acid | 125.03 | 123.86 | 123.86 | 1.17 |
| 3820 | 1-Hexadecanol | 76.24 | 77.41 | 77.55 | -1.17 |
| 3821 | 2,4-Dichloronitrobenzene | 41.43 | 42.61 | 42.72 | -1.18 |
| 3822 | Methyl pyruvate | 21.66 | 22.84 | 22.83 | -1.18 |
| 3823 | 1,3,5-Trimethyl-1H-pyrazole | 32.48 | 33.67 | 34.25 | -1.19 |
| 3824 | 4-Benzylpiperidine | 54.62 | 55.81 | 55.82 | -1.19 |
| 3825 | Dibutyl fumarate | 62.39 | 61.20 | 61.18 | 1.19 |
| 3826 | 1-Chloro-2-methyl-1-butene | 28.50 | 29.69 | 29.75 | -1.19 |
| 3827 | 1-Nitrobutane | 27.48 | 26.29 | 26.22 | 1.19 |
| 3828 | Trimyristin | 216.60 | 215.40 | 215.33 | 1.20 |
| 3829 | 1,2-Diiodoethane | 38.54 | 37.34 | 37.22 | 1.20 |
| 3830 | 2,4-Dimethyloxazole | 26.09 | 27.29 | 28.02 | -1.20 |
| 3831 | Butanedial | 20.72 | 21.92 | 21.86 | -1.20 |
| 3832 | Ethylorthosilicate | 53.33 | 52.12 | 51.81 | 1.21 |
| 3833 | 1-Methyl-4-vinylcyclohexene | 40.12 | 41.33 | 41.36 | -1.21 |
| 3834 | 1-Nitro-3-(trifluoromethyl)benzene | 37.27 | 38.48 | 38.55 | -1.21 |
| 3835 | Montanic acid | 134.31 | 133.10 | 133.09 | 1.21 |
| 3836 | 4-Ethylstyrene | 46.52 | 45.30 | 45.28 | 1.22 |
| 3837 | trans-2-Methoxy-4-(1-propenyI)phenol | 50.24 | 49.02 | 49.01 | 1.22 |
| 3838 | Methylanthranilate | 43.13 | 41.91 | 42.00 | 1.22 |
| 3839 | Thioacetic acid | 19.77 | 18.55 | 18.20 | 1.22 |
| 3840 | Pentafluorophenol | 26.97 | 28.19 | 28.33 | -1.22 |
| 3841 | Cyclodecanone | 45.44 | 46.66 | 46.66 | -1.22 |
| 3842 | 1-Vinyl-3-methoxybenzene | 43.65 | 42.42 | 42.39 | 1.23 |
| 3843 | 1-Vinyl-4-methoxybenzene | 43.65 | 42.42 | 42.44 | 1.23 |
| 3844 | Phenylacetone | 39.95 | 41.18 | 41.18 | -1.23 |
| 3845 | Di-n-propylsulfide | 38.94 | 37.71 | 37.69 | 1.23 |
| 3846 | (Methoxymethyl)oxirane | 23.11 | 21.88 | 21.88 | 1.23 |
| 3847 | trans,trans-2,4-Hexadiene | 31.15 | 29.92 | 29.92 | 1.23 |
| 3848 | 1,3,5-Cyclooctatriene | 35.84 | 37.08 | 37.02 | -1.24 |
| 3849 | 1,2-Dibromo-1,2-dichloroethane | 39.24 | 38.00 | 36.34 | 1.24 |
| 3850 | 2-Phenyl-2-propanol | 43.39 | 42.15 | 42.12 | 1.24 |
| 3851 | Ethyl 4,4,4-trifluoroacetoacetate | 33.75 | 32.51 | 32.49 | 1.24 |
| 3852 | 1,2,4a,5,8,8a-Hexahydro-4,7-dimethyl-1-isopropylnaphthalene | 65.77 | 67.02 | 67.00 | -1.25 |
| 3853 | 2-Methoxyphenylisothiocyanate | 50.47 | 49.22 | 48.99 | 1.25 |
| 3854 | Benzyldimethylamine | 43.54 | 44.79 | 44.85 | -1.25 |
| 3855 | Chloromethyldiphenylsilane | 68.14 | 69.39 | 69.68 | -1.25 |
| 3856 | 1,3-Diphenyl-2-buten-1-one | 71.77 | 70.52 | 70.49 | 1.25 |
| 3857 | Isobutyl butanoate | 42.10 | 40.85 | 40.83 | 1.25 |
| 3858 | Naphthalene | 43.21 | 44.46 | 44.50 | -1.25 |
| 3859 | trans-(3-Chloro-1-propenyl)benzene | 46.82 | 45.57 | 45.57 | 1.25 |
| 3860 | 1,3-Divinylbenzene | 46.36 | 45.10 | 45.08 | 1.26 |
| 3861 | 4-Ethylnitrobenzene | 50.67 | 51.93 | 52.01 | -1.26 |
| 3862 | 4-Acetylphenol | 39.56 | 38.30 | 38.31 | 1.26 |
| 3863 | 1-Vinylnaphthalene | 52.40 | 53.66 | 53.72 | -1.26 |
| 3864 | Isopropyl palmitate | 92.95 | 91.68 | 91.70 | 1.27 |
| 3865 | Octadecanenitrile | 83.86 | 85.13 | 85.12 | -1.27 |
| 3866 | 1,2,3,4-Tetrahydroisoquinoline | 40.87 | 42.14 | 42.19 | -1.27 |
| 3867 | 2-Acetylcyclohexanone | 39.13 | 37.85 | 37.83 | 1.28 |
| 3868 | 2-Benzylpyridine | 52.67 | 53.95 | 54.01 | -1.28 |
| 3869 | 5-(1-Methylethylidene)-1,3-cyclopentadiene | 38.24 | 36.96 | 36.96 | 1.28 |
| 3870 | Forstab | 100.03 | 98.74 | 98.15 | 1.29 |
| 3871 | 1,4-Cyclohexadiene | 26.51 | 27.80 | 27.84 | -1.29 |
| 3872 | 2-(Methacryloyloxy)ethyl acetoacetate | 51.90 | 53.19 | 53.10 | -1.29 |
| 3873 | 2-Methyl-1-buten-3-yne | 24.29 | 23.00 | 22.98 | 1.29 |
| 3874 | 3-Chloronitrobenzene | 36.66 | 37.95 | 37.97 | -1.29 |
| 3875 | Cyclopentadiene | 21.89 | 23.18 | 23.15 | -1.29 |
| 3876 | trans-2-Butenedinitrile | 21.63 | 20.34 | 20.30 | 1.29 |
| 3877 | 1,2-Propanediol 1-methacrylate | 36.05 | 37.35 | 37.36 | -1.30 |
| 3878 | 3-Tolunitrile | 34.81 | 36.11 | 36.15 | -1.30 |
| 3879 | (3-Chloropropyl)benzene | 44.23 | 45.53 | 45.53 | -1.30 |
| 3880 | Julolidine | 56.51 | 55.21 | 55.26 | 1.30 |
| 3881 | sec-Hexyl acetate | 39.53 | 40.83 | 40.86 | -1.30 |
| 3882 | 1,2,3,5-Tetrafluorobenzene | 27.80 | 26.50 | 26.46 | 1.30 |
| 3883 | Butyl isobutyl ether | 42.08 | 40.77 | 40.77 | 1.31 |
| 3884 | R-11 | 56.95 | 58.26 | 58.32 | -1.31 |
| 3885 | 4-Methyl-1,3-pentadiene | 30.93 | 29.62 | 29.63 | 1.31 |
| 3886 | 2-(Diethylamino)ethyl acrylate | 47.94 | 49.25 | 49.24 | -1.31 |
| 3887 | 3-Methoxyphenylacetone | 46.39 | 47.70 | 47.72 | -1.31 |
| 3888 | Saccharose | 67.70 | 69.01 | 69.74 | -1.31 |
| 3889 | trans-1,3-Hexadiene | 30.99 | 29.68 | 29.67 | 1.31 |
| 3890 | 1-Methoxy-1-buten-3-yne | 25.83 | 24.51 | 24.47 | 1.32 |
| 3891 | 4,4-Diethoxy-1-butanamine | 44.42 | 45.74 | 45.78 | -1.32 |
| 3892 | Allyl ether | 29.84 | 31.16 | 31.14 | -1.32 |
| 3893 | Anethole | 48.60 | 47.28 | 47.24 | 1.32 |
| 3894 | trans-1,3-Butadienylbenzene | 46.50 | 45.18 | 45.17 | 1.32 |
| 3895 | 2,4-Hexanedione | 32.09 | 30.76 | 30.70 | 1.33 |
| 3896 | 3-Methyl-1,3-butanediol | 29.35 | 28.02 | 28.04 | 1.33 |
| 3897 | 2,5-Dimethyloxazole | 25.63 | 26.97 | 27.23 | -1.34 |
| 3898 | Cumulene | 17.25 | 15.91 | 15.77 | 1.34 |
| 3899 | p-Anisylchloride | 45.02 | 43.68 | 43.66 | 1.34 |
| 3900 | beta-Ionone | 61.84 | 60.50 | 60.47 | 1.34 |
| 3901 | Tricyclene | 40.57 | 41.91 | 41.96 | -1.34 |
| 3902 | Ethylcarbamate | 22.59 | 21.24 | 20.95 | 1.35 |
| 3903 | 1,10-Dibromodecane | 65.29 | 63.94 | 63.90 | 1.35 |
| 3904 | 2,3'-Bipyridine | 48.27 | 46.92 | 46.91 | 1.35 |
| 3905 | 4-(2-Furanyl)-2-butanone | 37.18 | 38.53 | 38.60 | -1.35 |
| 3906 | Hexachlorobenzene | 53.31 | 54.66 | 54.96 | -1.35 |
| 3907 | 1-Butyne | 20.00 | 18.64 | 18.62 | 1.36 |
| 3908 | 2,5-Dimethylpyrrole | 30.10 | 31.46 | 31.99 | -1.36 |
| 3909 | Benzylaniline | 61.84 | 60.48 | 60.47 | 1.36 |
| 3910 | Benzyltrimethylsilane | 53.56 | 54.93 | 55.06 | -1.37 |
| 3911 | Diethyleneglycol dimethacrylate | 60.99 | 62.36 | 62.39 | -1.37 |
| 3912 | Difurfuryl ether | 46.64 | 48.02 | 48.21 | -1.38 |
| 3913 | Panthenol | 50.05 | 51.44 | 51.64 | -1.39 |
| 3914 | 2,3-Dihydro-2-methylbenzofuran | 39.11 | 40.50 | 40.47 | -1.39 |
| 3915 | Ascaridole | 47.04 | 48.43 | 48.75 | -1.39 |
| 3916 | Humulene | 67.93 | 69.32 | 69.31 | -1.39 |
| 3917 | Propylparaben | 50.28 | 48.88 | 48.88 | 1.40 |
| 3918 | Dimethyl pimelate | 45.85 | 47.26 | 47.28 | -1.41 |
| 3919 | 2-(Vinyloxy)ethanol | 24.41 | 23.00 | 22.96 | 1.41 |
| 3920 | Cyclooctanamine | 38.98 | 40.40 | 40.44 | -1.42 |
| 3921 | Azomethane | 19.78 | 21.20 | 21.88 | -1.42 |
| 3922 | 1-Tetradecanethiol | 73.00 | 74.42 | 74.43 | -1.42 |
| 3923 | cis-3-Phenyl-2-propenenitrile | 42.03 | 40.61 | 40.62 | 1.42 |
| 3924 | Ethoxyquin | 69.39 | 67.97 | 67.77 | 1.42 |
| 3925 | 4-Anisaldehyde | 40.09 | 38.66 | 38.67 | 1.43 |
| 3926 | Diphenylacetaldehyde | 60.04 | 61.47 | 61.50 | -1.43 |
| 3927 | Methyl 1-naphthoate | 57.08 | 55.64 | 55.69 | 1.44 |
| 3928 | Antipyrine | 57.44 | 56.00 | 54.51 | 1.44 |
| 3929 | Resorcinol | 28.74 | 30.18 | 30.20 | -1.44 |
| 3930 | 1,4-Dibromopentane | 42.30 | 40.86 | 40.84 | 1.44 |
| 3931 | 9,10-Dihydrophenanthrene | 60.48 | 59.04 | 58.96 | 1.44 |
| 3932 | Triallylamine | 45.60 | 47.05 | 47.15 | -1.45 |
| 3933 | 1,6-Hexanedial | 29.70 | 31.16 | 31.26 | -1.46 |
| 3934 | 2-Vinyl-5-norbornene | 40.67 | 39.21 | 39.17 | 1.46 |
| 3935 | cis-3-Methylcyclohexanol | 35.13 | 33.66 | 33.65 | 1.47 |
| 3936 | 1-Methoxy-1,3-butadiene | 27.74 | 26.27 | 26.22 | 1.47 |
| 3937 | Butyl oleate | 104.13 | 105.60 | 105.58 | -1.47 |
| 3938 | Tridecanoic acid | 65.28 | 63.80 | 63.79 | 1.48 |
| 3939 | 1-(Chloromethyl)-4-methoxybenzene | 41.33 | 42.81 | 42.79 | -1.48 |
| 3940 | 1-Methylbenzimidazole | 40.25 | 41.73 | 41.81 | -1.48 |
| 3941 | 2-Methyl-7,8-benzoquinoline | 63.25 | 64.73 | 64.96 | -1.48 |
| 3942 | Pentafluorobenzonitrile | 32.57 | 31.08 | 31.01 | 1.49 |
| 3943 | Propyne | 15.51 | 14.02 | 13.99 | 1.49 |
| 3944 | 2-Methylbenzoxazole | 37.82 | 39.31 | 39.83 | -1.49 |
| 3945 | beta-Phenylethylacetate | 45.65 | 47.14 | 47.15 | -1.49 |
| 3946 | Cyclohexyl acrylate | 41.67 | 43.16 | 43.17 | -1.49 |
| 3947 | Hentriacontane | 143.85 | 145.34 | 145.34 | -1.49 |
| 3948 | Dodecyl acetate | 70.10 | 68.60 | 68.58 | 1.50 |
| 3949 | Ethyl cyclohexanecarboxylate | 44.85 | 43.35 | 43.36 | 1.50 |
| 3950 | N,N-Dimethyl-2-nitroaniline | 48.86 | 47.36 | 47.36 | 1.50 |
| 3951 | Hexafluoro-1,3-butadiene | 24.06 | 22.56 |  | 1.50 |
| 3952 | 1,2-Dimethyl-4-nitrobenzene | 41.34 | 42.85 | 42.89 | -1.51 |
| 3953 | 1,3-Dimethyl-2-nitrobenzene | 41.34 | 42.85 | 42.94 | -1.51 |
| 3954 | Acetylacetone | 27.65 | 26.14 | 26.11 | 1.51 |
| 3955 | alpha-Bromobutyric acid | 28.53 | 30.05 | 30.07 | -1.52 |
| 3956 | 2-(Phenylmethylene)butanal | 52.12 | 50.60 | 50.57 | 1.52 |
| 3957 | (2-Bromoethyl)benzene | 42.38 | 43.91 | 43.91 | -1.53 |
| 3958 | Phenylthiocarboxylic acid | 37.13 | 38.67 | 39.35 | -1.54 |
| 3959 | Tri-m-cresylphosphate | 103.19 | 101.63 | 101.36 | 1.56 |
| 3960 | 2,5-Dimethylbenzoxazole | 42.52 | 44.09 | 43.64 | -1.57 |
| 3961 | Tetracyanoethylene | 30.73 | 29.16 | 29.04 | 1.57 |
| 3962 | (-)-Globulol | 67.93 | 66.36 | 66.39 | 1.57 |
| 3963 | Triethylene glycol dimethacrylate | 71.75 | 73.32 | 73.34 | -1.57 |
| 3964 | Cinoxate | 70.86 | 69.28 | 69.27 | 1.58 |
| 3965 | 2-Ethoxycarbonylaniline | 48.13 | 46.55 | 46.48 | 1.58 |
| 3966 | 1,5-Cyclooctadiene | 35.45 | 37.04 | 37.00 | -1.59 |
| 3967 | Cinnamoylchloride | 48.03 | 46.44 | 46.37 | 1.59 |
| 3968 | Sanger's Reagent | 41.43 | 39.83 | 39.73 | 1.60 |
| 3969 | 2-Nitrobenzeneethanol | 45.67 | 44.06 | 44.03 | 1.61 |
| 3970 | 2-Phenoxypropanoic acid | 42.47 | 44.08 | 44.06 | -1.61 |
| 3971 | 1,2,3,4,5,6,7,8-Octahydrophenanthrene | 58.44 | 60.06 | 60.06 | -1.62 |
| 3972 | Sucrose octaacetate | 147.99 | 146.37 | 145.53 | 1.62 |
| 3973 | Hexanamide | 34.71 | 33.08 | 33.14 | 1.63 |
| 3974 | N,N-Diethyl-2-methylaniline | 53.04 | 54.67 | 54.71 | -1.63 |
| 3975 | N,N-Dimethyl-o-toluidine | 43.92 | 45.55 | 45.67 | -1.63 |
| 3976 | 4-Hydroxybenzaldehyde | 35.52 | 33.88 | 33.94 | 1.64 |
| 3977 | N-Benzyl-N-ethylaniline | 71.66 | 70.01 | 69.90 | 1.65 |
| 3978 | 1,1-Diphenyl-1-propene | 63.79 | 65.44 | 65.51 | -1.65 |
| 3979 | Tartaric acid | 24.89 | 26.54 | 26.70 | -1.65 |
| 3980 | 3-Mercaptopropyl-trimethoxysilane | 51.18 | 49.52 | 49.38 | 1.66 |
| 3981 | Methylparathion | 60.50 | 62.16 | 62.37 | -1.66 |
| 3982 | 1,2-Diphenylethane | 59.16 | 60.84 | 60.94 | -1.68 |
| 3983 | alpha-Farnesene | 69.47 | 71.16 | 71.17 | -1.69 |
| 3984 | 2-Fluorobenzeneacetonitrile | 37.59 | 35.90 | 35.90 | 1.69 |
| 3985 | Propanal | 14.94 | 16.64 | 16.65 | -1.70 |
| 3986 | Biphenyl | 49.90 | 51.60 | 51.62 | -1.70 |
| 3987 | 3-(Phenylmethylene)-2-pentanone | 56.73 | 55.02 | 55.05 | 1.71 |
| 3988 | Ethyl 2-bromopentanoate | 45.80 | 44.09 | 44.09 | 1.71 |
| 3989 | Perfluoroisoprene | 25.97 | 27.68 |  | -1.71 |
| 3990 | Methylmyristate | 71.48 | 73.20 | 73.33 | -1.72 |
| 3991 | Cyclooctatetraene | 35.39 | 37.12 | 37.20 | -1.73 |
| 3992 | Ethyl sorbate | 42.69 | 40.96 | 40.91 | 1.73 |
| 3993 | 9-Decen-1-ol | 47.76 | 49.49 | 49.54 | -1.73 |
| 3994 | 3,4,5-Trimethoxybenzyl alcohol | 50.67 | 52.41 | 52.41 | -1.74 |
| 3995 | 2-Fluorophenol | 30.15 | 28.39 | 28.41 | 1.76 |
| 3996 | Dicyclohexylketone | 56.58 | 58.34 | 58.38 | -1.76 |
| 3997 | 2-Acetylcyclopentanone | 35.00 | 33.23 | 33.17 | 1.77 |
| 3998 | 2-Thiophenemethanol | 29.17 | 30.94 | 31.07 | -1.77 |
| 3999 | Butane-1,4-dithiol | 37.62 | 35.84 | 35.74 | 1.78 |
| 4000 | Methyl 2-(methylamino)benzoate | 48.76 | 46.98 | 46.91 | 1.78 |
| 4001 | 4-Vinylaniline | 41.71 | 39.93 | 39.91 | 1.78 |
| 4002 | N,N-Diethyldodecanamide | 81.75 | 79.96 | 79.85 | 1.79 |
| 4003 | 1,1-Diethylhydrazine | 25.41 | 27.20 |  | -1.79 |
| 4004 | Dichloroethylmethylsilane | 36.02 | 34.23 | 34.00 | 1.79 |
| 4005 | Methyl sorbate | 38.11 | 36.32 | 36.33 | 1.79 |
| 4006 | Dicyanomethane | 13.88 | 15.68 | 15.78 | -1.80 |
| 4007 | 2,6-Dimethylhepta-2,5-dien-4-one | 45.92 | 44.12 | 44.14 | 1.80 |
| 4008 | N,N-Dimethyl-2-naphthylamine | 60.34 | 58.53 | 58.52 | 1.81 |
| 4009 | Isobutylene | 22.16 | 20.34 | 20.31 | 1.82 |
| 4010 | beta-Bromocinnamaldehyde | 50.67 | 48.83 | 48.68 | 1.84 |
| 4011 | Methyl 3-bromopropanoate | 32.04 | 30.19 | 30.18 | 1.85 |
| 4012 | Benzeneazoethane | 44.92 | 46.77 | 47.34 | -1.85 |
| 4013 | Monoacetin | 27.89 | 29.74 | 29.73 | -1.85 |
| 4014 | o-Dinitrobenzene | 41.74 | 39.88 | 39.82 | 1.86 |
| 4015 | 2,5-Norbornadiene | 28.37 | 30.24 | 30.26 | -1.87 |
| 4016 | Bis(trifluoroacetyl)methane | 28.84 | 26.96 | 26.82 | 1.88 |
| 4017 | 1-Bromo-4-t-butylbenzene | 54.73 | 52.85 | 52.79 | 1.88 |
| 4018 | 9-Methyl-9H-fluorene | 60.89 | 59.01 | 59.01 | 1.88 |
| 4019 | 4-Fluoroaniline | 28.79 | 30.68 | 30.73 | -1.89 |
| 4020 | Methyl 3-phenyl-2-propynoate | 47.95 | 46.06 | 46.02 | 1.89 |
| 4021 | Antimalarine | 91.20 | 89.28 | 89.11 | 1.92 |
| 4022 | 9H-Fluoren-9-one | 56.81 | 54.88 | 54.81 | 1.93 |
| 4023 | Phenoxyacetylene | 33.43 | 35.36 | 35.94 | -1.93 |
| 4024 | Cyclopropane | 15.79 | 13.86 | 13.86 | 1.93 |
| 4025 | 1,3-Benzodioxole-5-ethanamine | 43.73 | 45.67 | 45.78 | -1.94 |
| 4026 | Cyclohexylamine | 33.10 | 31.16 | 31.17 | 1.94 |
| 4027 | n-Butylstearate | 103.61 | 105.56 | 105.56 | -1.95 |
| 4028 | Chloropentafluorobenzene | 33.07 | 31.11 | 31.03 | 1.96 |
| 4029 | Ethane-1,2-dithiol | 24.64 | 26.60 | 26.74 | -1.96 |
| 4030 | 2,6-Di-t-pentyl-4-methylphenol | 77.81 | 79.78 | 79.87 | -1.97 |
| 4031 | Dibenzofuran | 53.42 | 51.45 | 51.48 | 1.97 |
| 4032 | 1,4-Butanediamine | 29.41 | 27.44 | 27.40 | 1.97 |
| 4033 | 4-Phenyl-3-butyn-2-one | 46.72 | 44.74 | 44.68 | 1.98 |
| 4034 | 3-Acetyl-6-methylpyridine | 41.08 | 39.07 | 39.02 | 2.01 |
| 4035 | (2-Methoxyvinyl)benzene | 43.98 | 41.97 | 41.85 | 2.01 |
| 4036 | 1-Aziridineethanol | 21.77 | 23.80 | 23.83 | -2.03 |
| 4037 | 3-Phenyl-2-propynal | 42.36 | 40.32 | 40.24 | 2.04 |
| 4038 | 2-Bromo-1,4-dimethoxybenzene | 49.29 | 47.23 | 47.19 | 2.06 |
| 4039 | Ethyl 3-phenylpropynoate | 52.76 | 50.70 | 50.65 | 2.06 |
| 4040 | Furfurylamine | 25.57 | 27.63 | 27.66 | -2.06 |
| 4041 | N,N-Diisopropylethanolamine | 46.50 | 44.44 | 44.32 | 2.06 |
| 4042 | trans-1,3,5-Hexatriene | 31.55 | 29.48 | 29.44 | 2.07 |
| 4043 | n-Undecanal | 55.68 | 53.60 | 53.70 | 2.08 |
| 4044 | 4-t-Butyl-2,5-dimethylphenol | 58.75 | 56.66 | 56.60 | 2.09 |
| 4045 | Diethyl oxalacetate | 45.24 | 43.14 | 43.11 | 2.10 |
| 4046 | Stearic acid anhydride | 172.28 | 170.17 | 170.05 | 2.11 |
| 4047 | 6-Nitro-9-oxaphenanthren-10-one | 64.81 | 62.70 | 62.63 | 2.11 |
| 4048 | Potasan | 85.31 | 83.20 | 82.92 | 2.11 |
| 4049 | 4-t-Butylbenzenemethanol | 53.62 | 51.51 | 51.54 | 2.11 |
| 4050 | 9-Ethyl-9H-carbazole | 66.38 | 64.25 | 64.03 | 2.13 |
| 4051 | Perfluoro-n-octane | 44.73 | 42.60 | 42.06 | 2.13 |
| 4052 | 2-Methyl-3-phenyl-2-propenal | 48.13 | 45.98 | 45.95 | 2.15 |
| 4053 | Vitamin K1 | 143.28 | 141.13 | 141.07 | 2.15 |
| 4054 | Glycerol trioleate | 268.81 | 270.97 | 270.96 | -2.16 |
| 4055 | 2-Oxobutyric acid | 20.50 | 22.68 | 22.69 | -2.18 |
| 4056 | Oxotremorine | 62.31 | 60.13 | 60.11 | 2.18 |
| 4057 | 1,3-Butadien-1-ol acetate | 33.04 | 30.84 | 30.83 | 2.20 |
| 4058 | 2-Methylpropane | 22.80 | 20.57 | 20.57 | 2.23 |
| 4059 | Acetaldol | 20.37 | 22.61 | 22.72 | -2.24 |
| 4060 | 6-t-Butyl-2,4-dimethylphenol | 58.94 | 56.66 | 56.72 | 2.28 |
| 4061 | Ethyl 2,4-dioxopentanoate | 39.47 | 37.18 | 37.14 | 2.29 |
| 4062 | Spiro[2.2]pentane | 23.33 | 21.00 | 21.01 | 2.33 |
| 4063 | trans-3-Phenyl-2-propenenitrile | 42.96 | 40.61 | 40.62 | 2.35 |
| 4064 | Tri-o-cresylphosphate | 99.27 | 101.63 | 102.11 | -2.36 |
| 4065 | 4-Chromanone | 43.36 | 40.98 | 40.98 | 2.38 |
| 4066 | Glycerol tri-3-methylbutanoate | 79.09 | 76.71 | 76.71 | 2.38 |
| 4067 | 4-Pentylbenzoyl chloride | 62.82 | 60.42 | 60.35 | 2.40 |
| 4068 | Cannabidiol | 94.93 | 97.34 | 97.46 | -2.41 |
| 4069 | 4-Nitroanisole | 37.38 | 39.81 | 39.91 | -2.43 |
| 4070 | 4-Nitrothioanisole | 49.20 | 46.76 | 46.47 | 2.44 |
| 4071 | Cellosolve acetate | 26.22 | 23.77 | 23.76 | 2.45 |
| 4072 | 4-t-Butylbenzoyl chloride | 58.27 | 55.82 | 55.75 | 2.45 |
| 4073 | 9-Methylanthracene | 69.46 | 67.00 | 66.89 | 2.46 |
| 4074 | 4-Butylbenzoyl chloride | 58.27 | 55.80 | 55.82 | 2.47 |
| 4075 | Ethyl 2-benzylidene-2-cyanoacetate | 53.73 | 56.21 | 56.27 | -2.48 |
| 4076 | 1,1-Difluoroethane | 13.83 | 11.35 | 10.92 | 2.48 |
| 4077 | s-trans-1,3-Butadiene | 22.69 | 20.20 | 20.18 | 2.49 |
| 4078 | 2-Methyl-5-isopropylaniline | 47.00 | 49.50 | 49.61 | -2.50 |
| 4079 | Ethyl trans-cinnamate | 54.30 | 51.80 | 51.78 | 2.50 |
| 4080 | Cyclobutyrol | 51.72 | 49.22 | 49.18 | 2.50 |
| 4081 | (3-Bromo-1-propenyl)benzene | 51.09 | 48.57 | 48.58 | 2.52 |
| 4082 | Diethyl benzylidenemalonate | 69.94 | 67.40 | 67.34 | 2.54 |
| 4083 | trans-Geranyl bromide | 58.65 | 56.09 | 56.08 | 2.56 |
| 4084 | 2,6-Diethylaniline | 52.09 | 49.53 | 49.47 | 2.56 |
| 4085 | Pentafluorobenzenethiol | 36.83 | 34.25 | 33.76 | 2.58 |
| 4086 | 4-Phenylbut-3-en-2-one | 48.43 | 45.84 | 45.82 | 2.59 |
| 4087 | 5-(Hydroxymethyl)-2-furaldehyde | 33.94 | 31.30 | 31.04 | 2.64 |
| 4088 | Isopropyl methyl sulfide | 31.62 | 28.96 | 28.80 | 2.66 |
| 4089 | Furfurylacetate | 32.57 | 35.25 | 35.29 | -2.68 |
| 4090 | Hexadecanoic anhydride | 154.37 | 151.69 | 151.57 | 2.68 |
| 4091 | Hexatriacontane | 171.14 | 168.44 | 168.44 | 2.70 |
| 4092 | 2-t-Butyl-5-methylphenol | 54.59 | 51.88 | 51.85 | 2.71 |
| 4093 | 2-Methoxybenzylalcohol | 42.09 | 39.37 | 39.39 | 2.72 |
| 4094 | 1-Phenylbutane-1,3-dione | 50.05 | 47.33 | 47.21 | 2.72 |
| 4095 | 3-Methylbenzoyl chloride | 44.67 | 41.94 | 41.90 | 2.73 |
| 4096 | Tetramethyldiaminobutane | 49.95 | 47.22 | 47.04 | 2.73 |
| 4097 | N'-Ethyl-N,N-dimethyl-1,2-ethanediamine | 40.03 | 37.29 | 37.25 | 2.74 |
| 4098 | Hydroxyacetaldehyde diethylacetal | 37.22 | 34.45 | 34.43 | 2.77 |
| 4099 | 1-Bromo-4-ethylbenzene | 40.82 | 43.59 | 43.58 | -2.77 |
| 4100 | Pyrocatechol | 32.95 | 30.18 | 30.22 | 2.77 |
| 4101 | Cinnamaldehyde | 44.20 | 41.42 | 41.48 | 2.78 |
| 4102 | 3,4,5,6-Tetrahydro-7-methoxy-2H-azepine | 39.49 | 36.65 | 35.69 | 2.84 |
| 4103 | 1,2,5-Trimethylpyrrole | 39.58 | 36.71 | 35.22 | 2.87 |
| 4104 | 2,2-Diethyl-1,3-propanediol | 34.32 | 37.22 | 37.31 | -2.90 |
| 4105 | 6,6,7,7,8,8,8-Heptafluoro-2,2-dimethyl-3,5-octanedione | 53.47 | 50.57 | 50.50 | 2.90 |
| 4106 | O,O,O-Triethyl thiophosphate | 38.82 | 35.92 | 35.61 | 2.90 |
| 4107 | 1-Chloroanthracene | 69.90 | 66.88 | 66.73 | 3.02 |
| 4108 | 1,3-Benzodioxole | 35.90 | 32.85 | 32.66 | 3.05 |
| 4109 | Sorbinaldehyde | 33.64 | 30.58 | 30.52 | 3.06 |
| 4110 | 1-Nitro-2-phenoxybenzene | 56.72 | 59.84 | 59.98 | -3.12 |
| 4111 | 3-Ethylbenzoic acid | 45.64 | 42.50 | 42.49 | 3.14 |
| 4112 | Dipivaloylmethane | 57.04 | 53.90 | 53.88 | 3.14 |
| 4113 | Methylsuccinic acid | 25.19 | 28.43 | 28.44 | -3.24 |
| 4114 | 2-Bromocyclohexanone | 39.40 | 36.01 | 35.94 | 3.39 |
| 4115 | 2,5-Diphenyloxazole | 70.72 | 67.21 | 66.51 | 3.51 |
| 4116 | Monoethyl adipate | 46.04 | 42.50 | 42.47 | 3.54 |
| 4117 | trans,trans-2,4-Hexadienoyl chloride | 39.26 | 35.60 | 35.58 | 3.66 |
| 4118 | Salicylic acid | 31.18 | 34.84 | 34.87 | -3.66 |
| 4119 | trans,trans-2,4-Heptadienal | 38.87 | 35.20 | 35.21 | 3.67 |
| 4120 | Ethyl 3-methyl-3-phenylglycidate | 59.88 | 56.02 | 55.91 | 3.86 |
| 4121 | Butyl trifluoroacetate | 35.93 | 32.05 | 31.97 | 3.88 |
| 4122 | 4-Ethoxyazobenzene | 78.57 | 74.38 | 73.42 | 4.19 |
| 4123 | trans-Stilbene | 65.77 | 60.88 | 60.88 | 4.89 |
