# Supplementary material for: A Generally Applicable Computer Algorithm Based on the Group Additivity Method for the Calculation of Seven Molecular Descriptors: Heat of Combustion, LogPO/W, LogS, Refractivity, Polarizability, Toxicity and LogBB of Organic Compounds; Scope and Limits of Applicability
Source: Molecules. 2015 Oct 7;20(10):18279–351. doi: 10.3390/molecules201018279 (PMC6332030; doi:10.3390/molecules201018279)
Supplement: Supplementary file 1 [file molecules-20-18279-s001.zip › Experimental vs Calculated Toxicity Data Table.doc]

**Experimental vs. Calculated Toxicity Data, sorted by absolute Deviation**

| **No.** | **Molecule name** | **Toxicity** | **Toxicity calc** | **Toxicity test** | **Deviation** |
| --- | --- | --- | --- | --- | --- |
|  |  |  |  |  |  |
| 1 | 3,4-Dibromohexane | 1.00 | 1.00 |  | 0.00 |
| 2 | 2-Fluorophenol | 0.19 | 0.19 | 0.17 | 0.00 |
| 3 | p-Toluidine | -0.05 | -0.05 | -0.11 | 0.00 |
| 4 | 4-Fluorobenzonitrile | -0.26 | -0.26 | -0.27 | 0.00 |
| 5 | 4-Hydroxy-3-nitrobenzaldehyde | 0.61 | 0.61 | 0.61 | 0.00 |
| 6 | 4-Iodophenol | 0.85 | 0.85 | 0.83 | 0.00 |
| 7 | Tetramethylhydroquinone | 1.28 | 1.28 | 1.28 | 0.00 |
| 8 | 3,5-Dichloroaniline | 0.71 | 0.71 | 0.71 | 0.00 |
| 9 | Diethylacetamide | -1.54 | -1.54 |  | 0.00 |
| 10 | cis-3-Hexen-1-ol | -0.81 | -0.81 | -0.77 | 0.00 |
| 11 | Benzo[h]quinoline | 1.31 | 1.31 | 1.29 | 0.00 |
| 12 | 1-Nitrobutane | -0.69 | -0.69 | -0.69 | 0.00 |
| 13 | Dibenzofuran | 1.42 | 1.42 | 1.43 | 0.00 |
| 14 | cis-2-Butene-1,4-diol | -2.21 | -2.20 | -2.17 | -0.01 |
| 15 | 4-Chloro-3-ethylphenol | 1.08 | 1.07 | 1.02 | 0.01 |
| 16 | 2-Biphenyl methanol | 0.82 | 0.81 | 0.83 | 0.01 |
| 17 | 1,7-Phenanthroline | 1.07 | 1.06 | 1.03 | 0.01 |
| 18 | 3-Methyl-3-buten-1-ol | -1.18 | -1.19 | -1.27 | 0.01 |
| 19 | 3,5-Dichloroanisole | 0.93 | 0.92 | 0.87 | 0.01 |
| 20 | Bornyl acetate | 0.68 | 0.67 | 0.75 | 0.01 |
| 21 | 2,6-Dinitroaniline | 0.84 | 0.85 | 0.82 | -0.01 |
| 22 | Caprylonitrile | 0.28 | 0.29 | 0.27 | -0.01 |
| 23 | 2,6-Difluorophenol | 0.47 | 0.48 | 0.49 | -0.01 |
| 24 | Methyl phenoxyacetate | -0.21 | -0.20 | -0.21 | -0.01 |
| 25 | 4-Acetylphenol | -0.30 | -0.31 | -0.35 | 0.01 |
| 26 | 3-Nitrophenol | 0.51 | 0.50 | 0.54 | 0.01 |
| 27 | 2-Tolunitrile | -0.24 | -0.25 | -0.22 | 0.01 |
| 28 | 3-Tolunitrile | -0.24 | -0.25 | -0.25 | 0.01 |
| 29 | (Chloromethyl)benzene | 0.10 | 0.09 | 0.10 | 0.01 |
| 30 | 4-Methylcatechol | 0.37 | 0.38 | 0.35 | -0.01 |
| 31 | Octylcyanide | 0.62 | 0.63 | 0.66 | -0.01 |
| 32 | cis-2-Methoxy-4-(1-propenyl)phenol | 0.75 | 0.74 | 0.80 | 0.01 |
| 33 | trans-2-Methoxy-4-(1-propenyI)phenol | 0.75 | 0.74 | 0.65 | 0.01 |
| 34 | Hexanenitrile | -0.38 | -0.39 | -0.36 | 0.01 |
| 35 | 2,3,5,6-Tetrachloroaniline | 1.76 | 1.77 | 1.77 | -0.01 |
| 36 | 3-Fluorobenzyl alcohol | -0.39 | -0.40 | -0.38 | 0.01 |
| 37 | 4-tert-Pentylphenol | 1.23 | 1.22 | 1.24 | 0.01 |
| 38 | 2,4-Lutidine | -0.65 | -0.66 | -0.65 | 0.01 |
| 39 | 3,4-Dichlorotoluene | 1.07 | 1.08 | 1.08 | -0.01 |
| 40 | N,N-Dimethylpropionamide | -1.57 | -1.58 |  | 0.01 |
| 41 | 1-Bromo-2,6-dichlorobenzene | 1.34 | 1.35 | 1.31 | -0.01 |
| 42 | 4-Phenylphenol | 1.39 | 1.40 | 1.35 | -0.01 |
| 43 | 1,2-Propanediol | -2.51 | -2.49 | -2.46 | -0.02 |
| 44 | 3-Penten-2-ol | -1.40 | -1.38 | -1.38 | -0.02 |
| 45 | 5-Hexen-1-ol | -0.84 | -0.82 | -0.80 | -0.02 |
| 46 | 4,5-Dichloro-2-nitroaniline | 1.66 | 1.68 | 1.67 | -0.02 |
| 47 | 1-Methoxynaphthalene | 0.80 | 0.78 | 0.79 | 0.02 |
| 48 | 2-Buten-1-ol | -1.47 | -1.49 | -1.47 | 0.02 |
| 49 | 5-Chloropentylacetate | -0.13 | -0.15 | -0.11 | 0.02 |
| 50 | Methyl isoamyl ketone | -0.65 | -0.63 | -0.68 | -0.02 |
| 51 | Butyrophenone | 0.21 | 0.19 | 0.21 | 0.02 |
| 52 | 3-Phenyl-1-butanol | 0.01 | 0.03 | 0.03 | -0.02 |
| 53 | 2-Ethylfuran | -0.52 | -0.50 | -0.51 | -0.02 |
| 54 | 4-Chlorobenzonitrile | 0.00 | -0.02 | -0.05 | 0.02 |
| 55 | 4'-Hydroxypropiophenone | 0.05 | 0.03 | 0.11 | 0.02 |
| 56 | 3-Octanol | -0.06 | -0.08 | -0.10 | 0.02 |
| 57 | 2-(4-Chlorophenyl)ethylamine | 0.14 | 0.12 |  | 0.02 |
| 58 | 2,3-Dimethylfuran | -0.51 | -0.53 | -0.56 | 0.02 |
| 59 | 4-Bromobenzophenone | 1.26 | 1.28 | 1.27 | -0.02 |
| 60 | 2-Methyl-3-nitrophenol | 0.78 | 0.80 | 0.77 | -0.02 |
| 61 | 4-Bromo-6-chloro-2-methylphenol | 1.28 | 1.30 | 1.30 | -0.02 |
| 62 | 2-Nitroresorcinol | 0.66 | 0.68 | 0.71 | -0.02 |
| 63 | 2-Fluoropyridine | -1.20 | -1.18 |  | -0.02 |
| 64 | 3-Benzylpyridine | 0.68 | 0.70 | 0.71 | -0.02 |
| 65 | 2-Bromo-5-nitrotoluene | 1.16 | 1.19 | 1.17 | -0.03 |
| 66 | Valerophenone | 0.56 | 0.53 | 0.52 | 0.03 |
| 67 | 4-Chloro-2-methylphenol | 0.70 | 0.73 | 0.72 | -0.03 |
| 68 | Benzaldehyde | -0.20 | -0.17 | -0.21 | -0.03 |
| 69 | Acetophenone | -0.46 | -0.49 | -0.51 | 0.03 |
| 70 | 4-Bromoanisole | 0.40 | 0.43 | 0.47 | -0.03 |
| 71 | 4-t-Butylphenol | 0.91 | 0.88 | 0.85 | 0.03 |
| 72 | 3-Chlorobenzonitrile | -0.05 | -0.02 | -0.06 | -0.03 |
| 73 | Ethyl 3-hydroxybutanoate | -1.53 | -1.50 | -1.48 | -0.03 |
| 74 | 3-Methyl-1-hexyn-3-ol | -0.90 | -0.87 | -0.95 | -0.03 |
| 75 | 4-Nitrotoluene | 0.65 | 0.62 | 0.58 | 0.03 |
| 76 | Methylenanthate | 0.10 | 0.13 | 0.15 | -0.03 |
| 77 | 2-Phenylpyridine | 0.27 | 0.24 | 0.20 | 0.03 |
| 78 | 2,3,5,6-Tetrafluoropyridine | -0.61 | -0.64 |  | 0.03 |
| 79 | Isobutyl bromide | -0.32 | -0.29 | -0.28 | -0.03 |
| 80 | 1,6-Hexanedithiol | 0.63 | 0.60 | 0.62 | 0.03 |
| 81 | Allyl propanoate | -0.88 | -0.85 | -0.83 | -0.03 |
| 82 | 2-Chloro-4-nitroaniline | 0.75 | 0.78 | 0.78 | -0.03 |
| 83 | Ethyl phenyl sulfone | -0.75 | -0.72 |  | -0.03 |
| 84 | n-Crotylacetate | -0.87 | -0.84 | -0.79 | -0.03 |
| 85 | 3-Isopropylphenol | 0.61 | 0.58 | 0.59 | 0.03 |
| 86 | trans-3-Hexen-1-ol | -0.78 | -0.81 | -0.82 | 0.03 |
| 87 | Dimethylacetamide | -1.95 | -1.92 |  | -0.03 |
| 88 | 4-Pentoxyphenol | 1.36 | 1.39 | 1.36 | -0.03 |
| 89 | cis-2-Penten-1-ol | -1.12 | -1.15 | -1.16 | 0.03 |
| 90 | 4-Heptyloxyphenol | 2.03 | 2.07 | 2.09 | -0.04 |
| 91 | Di-n-butyl phthalate | 1.60 | 1.56 | 1.64 | 0.04 |
| 92 | 4-Nitrophenetole | 0.83 | 0.79 | 0.75 | 0.04 |
| 93 | 2-Methylbenzylalcohol | -0.43 | -0.39 | -0.38 | -0.04 |
| 94 | Allyl hexanoate | 0.21 | 0.17 | 0.18 | 0.04 |
| 95 | 3-Ethyl-2,2-dimethyl-3-pentanol | -0.17 | -0.21 | -0.26 | 0.04 |
| 96 | Pentane | -0.20 | -0.16 | -0.13 | -0.04 |
| 97 | 4-Ethylpyridine | -0.46 | -0.50 | -0.53 | 0.04 |
| 98 | 2-Bromopentane | 0.21 | 0.25 |  | -0.04 |
| 99 | 2-Fluorobenzophenone | 0.96 | 1.00 | 0.98 | -0.04 |
| 100 | 2-Amino-4-nitrophenol | 0.47 | 0.43 | 0.43 | 0.04 |
| 101 | 3-Chloroaniline | 0.22 | 0.18 | 0.20 | 0.04 |
| 102 | Anisole | -0.10 | -0.14 | -0.15 | 0.04 |
| 103 | 4-Chlorobenzaldehyde | 0.40 | 0.36 | 0.40 | 0.04 |
| 104 | 1-Nitropropane | -0.99 | -1.03 | -1.02 | 0.04 |
| 105 | 4-Ethoxy-2-nitroaniline | 0.76 | 0.72 | 0.66 | 0.04 |
| 106 | 4-Methylpyridine | -0.88 | -0.84 | -0.85 | -0.04 |
| 107 | 3,4-Lutidine | -0.50 | -0.54 | -0.56 | 0.04 |
| 108 | Methylacetamide | -1.98 | -1.94 | -1.92 | -0.04 |
| 109 | 2,4-Dinitrophenol | 1.06 | 1.10 | 1.15 | -0.04 |
| 110 | 2,5-Dimethyl-3-hexyne-2,5-diol | -1.58 | -1.62 | -1.71 | 0.04 |
| 111 | 1-Penten-3-ol | -1.35 | -1.39 | -1.35 | 0.04 |
| 112 | 1,2-Dibromohexane | 1.15 | 1.10 |  | 0.05 |
| 113 | Methylcaprylate | 0.52 | 0.47 | 0.49 | 0.05 |
| 114 | 2-Butanone oxime | -1.07 | -1.02 | -1.03 | -0.05 |
| 115 | Thioanisole | 0.18 | 0.13 |  | 0.05 |
| 116 | N-Ethylacetamide | -1.70 | -1.75 | -1.73 | 0.05 |
| 117 | 1-Nitrohexane | 0.04 | -0.01 | -0.02 | 0.05 |
| 118 | Ethyl benzoate | 0.01 | -0.04 | -0.03 | 0.05 |
| 119 | Apocynin | -0.12 | -0.17 | -0.16 | 0.05 |
| 120 | 2-Heptanol | -0.47 | -0.42 | -0.39 | -0.05 |
| 121 | 2-Picoline | -1.01 | -0.96 | -0.97 | -0.05 |
| 122 | Benzonitrile | -0.50 | -0.55 | -0.58 | 0.05 |
| 123 | 2,4-Dichloro-6-nitroaniline | 1.26 | 1.31 | 1.32 | -0.05 |
| 124 | 3-Bromopyridine | -0.52 | -0.57 | -0.56 | 0.05 |
| 125 | 4-Fluoro-3-(trifluoromethyl)-aniline | 0.77 | 0.82 | 0.85 | -0.05 |
| 126 | 1-Bromo-2,3-dichlorobenzene | 1.30 | 1.35 | 1.38 | -0.05 |
| 127 | 3-Phenylphenol | 1.35 | 1.40 | 1.35 | -0.05 |
| 128 | 2-Pentanol | -1.16 | -1.10 | -1.11 | -0.06 |
| 129 | 4-s-Butylphenol | 0.98 | 0.92 | 0.89 | 0.06 |
| 130 | 1-Nitropentane | -0.41 | -0.35 | -0.32 | -0.06 |
| 131 | Dimethyl phthalate | -0.52 | -0.46 | -0.44 | -0.06 |
| 132 | t-Pentyl alcohol | -1.17 | -1.23 | -1.22 | 0.06 |
| 133 | 4-Bromo-2,3,5,6-tetrafluoropyridine | -0.13 | -0.07 |  | -0.06 |
| 134 | 2-Ethylbutyl acetate | -0.12 | -0.18 | -0.17 | 0.06 |
| 135 | 4-Benzoylpyridine | -0.09 | -0.15 | -0.11 | 0.06 |
| 136 | 5-Fluoro-2-hydroxyacetophenone | 0.04 | -0.02 | -0.03 | 0.06 |
| 137 | 2-Octanol | -0.02 | -0.08 | -0.06 | 0.06 |
| 138 | 2-Chlorobenzyl alcohol | -0.10 | -0.16 | -0.14 | 0.06 |
| 139 | 2,4,5-Trichloroaniline | 1.30 | 1.24 | 1.23 | 0.06 |
| 140 | Ethyl acetate | -1.30 | -1.24 | -1.26 | -0.06 |
| 141 | trans-3-Pentenenitrile | -0.95 | -1.01 | -0.96 | 0.06 |
| 142 | 2-Methyl-3-butyn-2-ol | -1.49 | -1.55 | -1.57 | 0.06 |
| 143 | 4-Methyl-3-nitrophenol | 0.74 | 0.80 | 0.82 | -0.06 |
| 144 | trans-4-Hexen-1-ol | -0.75 | -0.81 | -0.81 | 0.06 |
| 145 | 3-Decanone | 0.63 | 0.69 | 0.71 | -0.06 |
| 146 | 3-Methyl-2-buten-1-ol | -1.24 | -1.18 | -1.18 | -0.06 |
| 147 | 2,5-Dinitrophenol | 1.04 | 1.10 | 1.06 | -0.06 |
| 148 | Ethyl 2-hydroxyisobutyrate | -1.57 | -1.63 | -1.55 | 0.06 |
| 149 | Dimethyl sulfone | -2.20 | -2.26 |  | 0.06 |
| 150 | 3,5-Di-tert-butylcatechol | 2.11 | 2.04 | 2.06 | 0.07 |
| 151 | 2-Iodobenzyl alcohol | 0.33 | 0.26 | 0.27 | 0.07 |
| 152 | Methylundecylate | 1.42 | 1.49 | 1.51 | -0.07 |
| 153 | 3-Hydroxyacetophenone | -0.38 | -0.31 | -0.35 | -0.07 |
| 154 | Allyl sulfide | -0.31 | -0.38 | -0.27 | 0.07 |
| 155 | 1,4-Dimethylbenzene | 0.25 | 0.32 | 0.33 | -0.07 |
| 156 | 1,2-Dimethylnaphthalene | 1.31 | 1.24 | 1.22 | 0.07 |
| 157 | 3-Heptanol | -0.49 | -0.42 | -0.42 | -0.07 |
| 158 | 4-Chloro-m-cresol | 0.80 | 0.73 | 0.74 | 0.07 |
| 159 | Benzamide | -0.91 | -0.84 | -0.83 | -0.07 |
| 160 | Diethyl malonate | -1.03 | -0.96 | -1.00 | -0.07 |
| 161 | 4-Methyl-2-heptyn-4-ol | -0.48 | -0.55 | -0.61 | 0.07 |
| 162 | Ethyl butanoate | -0.49 | -0.56 | -0.55 | 0.07 |
| 163 | n-Butyl acetate | -0.49 | -0.56 | -0.58 | 0.07 |
| 164 | N-Methylpropionamide | -1.53 | -1.60 | -1.65 | 0.07 |
| 165 | Di-n-propylsulfoxide | -1.22 | -1.14 |  | -0.08 |
| 166 | 4,7-Phenanthroline | 1.14 | 1.06 | 0.94 | 0.08 |
| 167 | 5-Chloro-1-pentyne | -0.49 | -0.41 | -0.35 | -0.08 |
| 168 | Phenyethyl isovalerate | 1.10 | 1.02 | 1.00 | 0.08 |
| 169 | 2,4,6-Trichlorophenol | 1.41 | 1.49 | 1.49 | -0.08 |
| 170 | 1-Phenyl-1-propanone | -0.07 | -0.15 | -0.16 | 0.08 |
| 171 | 3-Hydroxybenzaldehyde | 0.09 | 0.01 | 0.00 | 0.08 |
| 172 | 2,4-Dichlorophenol | 1.04 | 0.96 | 0.93 | 0.08 |
| 173 | sec-Phenethyl alcohol | -0.66 | -0.58 | -0.62 | -0.08 |
| 174 | 2-Naphthaleneethanol | 0.49 | 0.57 | 0.59 | -0.08 |
| 175 | 1,3-Dinitro-2,4,5-trichlorobenzene | 2.60 | 2.51 | 2.52 | 0.09 |
| 176 | 4-Allyl anisole | 0.64 | 0.55 | 0.51 | 0.09 |
| 177 | 4-Hexyloxyphenol | 1.64 | 1.73 | 1.76 | -0.09 |
| 178 | Ethyl 4-cyano-3-hydroxybutyrate | -1.64 | -1.73 | -1.66 | 0.09 |
| 179 | 3-Hexanol | -0.85 | -0.76 | -0.78 | -0.09 |
| 180 | 4-Methylanisole | 0.25 | 0.16 | 0.17 | 0.09 |
| 181 | 2-Phenyl-2-butanol | 0.06 | -0.03 | -0.06 | 0.09 |
| 182 | Pentabromophenol | 2.66 | 2.75 | 2.78 | -0.09 |
| 183 | Ethoxybenzene | 0.10 | 0.19 | 0.18 | -0.09 |
| 184 | 2-Phenyl-1-propanol | -0.40 | -0.31 | -0.32 | -0.09 |
| 185 | Styrene | -0.02 | 0.07 | 0.08 | -0.09 |
| 186 | 4-Acetylbiphenyl | 0.92 | 1.01 | 1.00 | -0.09 |
| 187 | 4-Aminophenol | -0.08 | -0.17 | -0.16 | 0.09 |
| 188 | 3,4,5,6-Tetrabromo-2-cresol | 2.57 | 2.48 | 2.48 | 0.09 |
| 189 | 1,8-Octanedithiol | 1.19 | 1.28 |  | -0.09 |
| 190 | 4-Penten-1-ol | -1.25 | -1.16 | -1.14 | -0.09 |
| 191 | Propylacetamide | -1.50 | -1.41 | -1.39 | -0.09 |
| 192 | DMSO | -2.45 | -2.36 |  | -0.09 |
| 193 | Tetrachlorohydroquinone | 2.11 | 2.20 | 2.20 | -0.09 |
| 194 | Ethanol | -1.99 | -1.89 | -1.87 | -0.10 |
| 195 | 1,2-Butanediol | -2.05 | -2.15 | -2.15 | 0.10 |
| 196 | 4-Bromo-2,6-dimethylphenol | 1.17 | 1.07 | 1.08 | 0.10 |
| 197 | 4-Methylvaleronitrile | -0.79 | -0.69 | -0.65 | -0.10 |
| 198 | 5-Bromovanillin | 0.62 | 0.72 | 0.76 | -0.10 |
| 199 | 2-Propanol | -1.88 | -1.78 | -1.74 | -0.10 |
| 200 | 4-Hexyloxyaniline | 1.38 | 1.48 | 1.46 | -0.10 |
| 201 | 4-Methylbenzylalcohol | -0.49 | -0.39 | -0.37 | -0.10 |
| 202 | Di-n-butylsulfone | -0.26 | -0.36 |  | 0.10 |
| 203 | 3-Nonanol | 0.36 | 0.26 | 0.26 | 0.10 |
| 204 | Phenylacetate | -0.23 | -0.13 | -0.16 | -0.10 |
| 205 | Di-n-propyl phthalate | 0.98 | 0.88 | 0.92 | 0.10 |
| 206 | Trifluoroacetophenone | -0.01 | 0.09 | 0.12 | -0.10 |
| 207 | 2-Heptanone oxime | 0.10 | 0.00 | -0.02 | 0.10 |
| 208 | 3-Methylcatechol | 0.28 | 0.38 | 0.42 | -0.10 |
| 209 | Decamethylene glycol | 0.22 | 0.12 | 0.14 | 0.10 |
| 210 | 1-Naphthalenemethanol | 0.13 | 0.23 | 0.27 | -0.10 |
| 211 | Diethyl phthalate | 0.10 | 0.20 | 0.24 | -0.10 |
| 212 | 3-Chloropyridine | -0.71 | -0.61 | -0.59 | -0.10 |
| 213 | 3-Chloroanisole | 0.29 | 0.39 | 0.43 | -0.10 |
| 214 | Ethyl 2-picolinate | -0.92 | -1.02 | -1.02 | 0.10 |
| 215 | (Ethylthio)benzene | 0.30 | 0.40 | 0.36 | -0.10 |
| 216 | 4-Butylaniline | 1.07 | 0.97 | 0.99 | 0.10 |
| 217 | 1,2,3-Trichlorobenzene | 1.21 | 1.31 | 1.28 | -0.10 |
| 218 | 2-Butanol | -1.54 | -1.44 | -1.43 | -0.10 |
| 219 | 1-Phenylnaphthalene | 2.04 | 2.14 | 2.18 | -0.10 |
| 220 | 4-Ethylbiphenyl | 1.97 | 1.86 | 1.79 | 0.11 |
| 221 | 2,3,5,6-Tetrafluorophenol | 1.17 | 1.06 | 1.09 | 0.11 |
| 222 | 4-Biphenylmethanol | 0.92 | 0.81 | 0.83 | 0.11 |
| 223 | 4-Cumylphenol | 0.47 | 0.58 | 0.59 | -0.11 |
| 224 | Benzylbromide | 0.64 | 0.53 | 0.54 | 0.11 |
| 225 | Nonyl 4-hydroxybenzoate | 2.63 | 2.52 | 2.53 | 0.11 |
| 226 | Propyl valerate | 0.01 | 0.12 | 0.15 | -0.11 |
| 227 | o-Toluidine | -0.16 | -0.05 | -0.03 | -0.11 |
| 228 | 1-Phenyl-1-butanol | -0.01 | 0.10 | 0.08 | -0.11 |
| 229 | Cyclohexene | -0.01 | 0.10 | 0.13 | -0.11 |
| 230 | 4-Chlorophenol | 0.54 | 0.43 | 0.45 | 0.11 |
| 231 | 3-Phenylpyridine | 0.47 | 0.36 | 0.34 | 0.11 |
| 232 | 4-Isopropylaniline | 0.22 | 0.33 | 0.32 | -0.11 |
| 233 | Isobutyl isobutyrate | -0.59 | -0.48 | -0.47 | -0.11 |
| 234 | 2-Butylacetate | -0.68 | -0.79 | -0.82 | 0.11 |
| 235 | 2-Decanone | 0.58 | 0.69 | 0.71 | -0.11 |
| 236 | Allyl cyanide | -1.48 | -1.36 | -1.32 | -0.12 |
| 237 | Trimethylacetamide | -1.48 | -1.36 | -1.37 | -0.12 |
| 238 | Methyl 2,5-dichlorobenzoate | 0.81 | 0.69 | 0.69 | 0.12 |
| 239 | 4-Hydroxybenzamide | -0.78 | -0.66 | -0.67 | -0.12 |
| 240 | 1-Bromo-4-isopropylbenzene | 0.85 | 0.97 | 0.95 | -0.12 |
| 241 | 3-Chloronitrobenzene | 0.73 | 0.85 | 0.81 | -0.12 |
| 242 | Syringaldehyde | 0.17 | 0.29 | 0.24 | -0.12 |
| 243 | Isobutyl propanoate | -0.64 | -0.52 | -0.54 | -0.12 |
| 244 | 2-Chloromethyl-4-nitrophenol | 0.75 | 0.87 | 0.87 | -0.12 |
| 245 | Pyrazine | -1.88 | -2.00 | -2.04 | 0.12 |
| 246 | n-Caproic acid vinyl ester | 0.08 | -0.04 | -0.06 | 0.12 |
| 247 | 4-Ethylbenzyl alcohol | 0.07 | -0.05 | -0.04 | 0.12 |
| 248 | (Propylthio)benzene | 0.86 | 0.74 | 0.70 | 0.12 |
| 249 | n-Propyl benzoate | 0.42 | 0.30 | 0.25 | 0.12 |
| 250 | Phenyl propargyl sulfide | 0.54 | 0.42 | 0.30 | 0.12 |
| 251 | 2,4-Difluorophenol | 0.60 | 0.48 | 0.48 | 0.12 |
| 252 | Diethylsulfone | -1.84 | -1.72 | -1.61 | -0.12 |
| 253 | Aniline | -0.23 | -0.35 | -0.33 | 0.12 |
| 254 | Dibutyl isophthalate | 1.44 | 1.56 | 1.64 | -0.12 |
| 255 | 2-t-Butyl-4-methylphenol | 1.30 | 1.18 | 1.15 | 0.12 |
| 256 | 2-Methyl-2,4-pentanediol | -1.96 | -1.83 | -1.75 | -0.13 |
| 257 | Isopentyl alcohol | -1.04 | -1.17 | -1.15 | 0.13 |
| 258 | 3,4,5-Trimethylphenol | 0.93 | 0.80 | 0.83 | 0.13 |
| 259 | 2,5-Dichloroaniline | 0.58 | 0.71 | 0.70 | -0.13 |
| 260 | 1-Bromopentane | 0.48 | 0.35 | 0.34 | 0.13 |
| 261 | 2-Methyl-2-propenol | -1.66 | -1.53 | -1.50 | -0.13 |
| 262 | 2,4-Dinitroaniline | 0.72 | 0.85 | 0.82 | -0.13 |
| 263 | 4-Hydroxybenzophenone | 1.02 | 0.89 | 0.84 | 0.13 |
| 264 | Ethyl 2-thiopheneacetate | 0.27 | 0.14 | 0.14 | 0.13 |
| 265 | Allyl butanoate | -0.64 | -0.51 | -0.49 | -0.13 |
| 266 | 4-Chloroaniline | 0.05 | 0.18 | 0.19 | -0.13 |
| 267 | 3,3-Dimethyl-1-butanol | -0.74 | -0.87 | -0.87 | 0.13 |
| 268 | Isoborneol | 0.15 | 0.02 | 0.01 | 0.13 |
| 269 | 2-Methyl-3-buten-2-ol | -1.39 | -1.52 | -1.52 | 0.13 |
| 270 | 1-Ethoxynaphthalene | 1.25 | 1.11 | 1.08 | 0.14 |
| 271 | 3-Pentanol | -1.24 | -1.10 | -1.06 | -0.14 |
| 272 | Benzyl alcohol | -0.83 | -0.69 | -0.69 | -0.14 |
| 273 | 2-Methylnaphthalene | 1.08 | 0.94 | 0.91 | 0.14 |
| 274 | 2,2'-Bithiophene | 1.04 | 0.90 | 0.70 | 0.14 |
| 275 | 4-Methylbiphenyl | 1.66 | 1.52 | 1.46 | 0.14 |
| 276 | 3-Bromonitrobenzene | 1.03 | 0.89 | 0.89 | 0.14 |
| 277 | beta-Ethylphenethylalcohol | -0.11 | 0.03 | 0.02 | -0.14 |
| 278 | Ethyl valerate | -0.36 | -0.22 | -0.19 | -0.14 |
| 279 | Methyl 3-hydroxybenzoate | -0.05 | -0.19 | -0.23 | 0.14 |
| 280 | 4-Chlorophenethyl alcohol | 0.32 | 0.18 | 0.18 | 0.14 |
| 281 | 5-Ethyl-2-picoline | -0.18 | -0.32 | -0.34 | 0.14 |
| 282 | 6-Phenyl-1-hexanol | 0.87 | 1.01 | 0.97 | -0.14 |
| 283 | 2-Methyl-1-propanol | -1.37 | -1.51 | -1.52 | 0.14 |
| 284 | 1-Bromo-2-nitrobenzene | 0.75 | 0.89 | 0.87 | -0.14 |
| 285 | 2-Bromophenol | 0.33 | 0.47 | 0.54 | -0.14 |
| 286 | 5-Phenyl-1-pentanol | 0.53 | 0.67 | 0.71 | -0.14 |
| 287 | 3-Methyl-2-butanone | -1.17 | -1.31 | -1.34 | 0.14 |
| 288 | 4-Pentyloxybenzaldehyde | 1.18 | 1.32 | 1.26 | -0.14 |
| 289 | 4-t-Octylphenol | 2.10 | 2.24 | 2.24 | -0.14 |
| 290 | 1,6-Dibromohexane | 1.05 | 1.20 | 1.26 | -0.15 |
| 291 | 3,4-Dichloroaniline | 0.56 | 0.71 | 0.69 | -0.15 |
| 292 | 3-Fluoro-4-nitrophenol | 0.94 | 0.79 | 0.77 | 0.15 |
| 293 | 4-Tolunitrile | -0.10 | -0.25 | -0.27 | 0.15 |
| 294 | 4-Hydroxy-3-methoxyphenethylalcohol | -0.18 | -0.03 | 0.02 | -0.15 |
| 295 | 3-t-Butylphenol | 0.73 | 0.88 | 0.88 | -0.15 |
| 296 | Phenylacetonitrile | -0.36 | -0.21 | -0.18 | -0.15 |
| 297 | 1,4-Dithiane | -0.11 | -0.26 | -0.50 | 0.15 |
| 298 | 6-Methyl-5-hepten-2-one | -0.45 | -0.30 | -0.16 | -0.15 |
| 299 | 3-Methylbenzylalcohol | -0.24 | -0.39 | -0.40 | 0.15 |
| 300 | 1-Hexanol | -0.38 | -0.53 | -0.47 | 0.15 |
| 301 | Isovaleronitrile | -0.88 | -1.03 | -0.96 | 0.15 |
| 302 | 3-Methylpyridine | -0.99 | -0.84 | -0.85 | -0.15 |
| 303 | 1,2,4-Trichlorobenzene | 1.16 | 1.31 | 1.27 | -0.15 |
| 304 | 1,3-Butyleneglycol | -2.30 | -2.15 | -2.12 | -0.15 |
| 305 | 1,2,4,5-Tetrachlorobenzene | 2.00 | 1.84 | 1.82 | 0.16 |
| 306 | 2,3,4,6-Tetrachlorophenol | 2.18 | 2.02 | 2.02 | 0.16 |
| 307 | 2-Heptanone | -0.49 | -0.33 | -0.30 | -0.16 |
| 308 | Thianaphthene | 0.26 | 0.42 | 0.61 | -0.16 |
| 309 | 2-Naphthalene methanol | 0.39 | 0.23 | 0.14 | 0.16 |
| 310 | 4-Isopropylbenzaldehyde | 0.67 | 0.51 | 0.55 | 0.16 |
| 311 | Di-n-butylsulfide | 1.04 | 0.88 | 0.83 | 0.16 |
| 312 | n-Hexylacetate | -0.04 | 0.12 | 0.13 | -0.16 |
| 313 | Pentyl propanoate | -0.04 | 0.12 | 0.15 | -0.16 |
| 314 | 2-Octanone | -0.15 | 0.01 | 0.05 | -0.16 |
| 315 | 1,2,4-Trimethylbenzene | 0.78 | 0.62 | 0.68 | 0.16 |
| 316 | 4-Trifluoromethylphenol | 0.62 | 0.78 | 0.82 | -0.16 |
| 317 | Benzophenone | 0.87 | 0.71 | 0.73 | 0.16 |
| 318 | m-Dinitrobenzene | 0.76 | 0.92 | 0.96 | -0.16 |
| 319 | Isobutyl formate | -1.33 | -1.17 | -1.15 | -0.16 |
| 320 | 1-Bromo-4-ethylbenzene | 1.10 | 0.93 | 0.94 | 0.17 |
| 321 | t-Butyl propionate | -0.41 | -0.58 | -0.58 | 0.17 |
| 322 | 4-Cyclopentylphenol | 1.29 | 1.46 | 1.50 | -0.17 |
| 323 | Chloroxylenol | 1.20 | 1.03 | 1.01 | 0.17 |
| 324 | Biphenyl | 1.05 | 1.22 | 1.14 | -0.17 |
| 325 | 2-Chloronitrobenzene | 0.68 | 0.85 | 0.85 | -0.17 |
| 326 | 1-Propynylbenzene | 0.19 | 0.36 | 0.44 | -0.17 |
| 327 | 3,7-Dimethyl-3-octanol | 0.34 | 0.17 | 0.12 | 0.17 |
| 328 | 2-Hexanol | -0.93 | -0.76 | -0.77 | -0.17 |
| 329 | Phenyl vinyl sulfide | 0.42 | 0.25 | 0.03 | 0.17 |
| 330 | 2-Nitroaniline | 0.08 | 0.25 | 0.25 | -0.17 |
| 331 | 2-Nitrophenol | 0.67 | 0.50 | 0.49 | 0.17 |
| 332 | 4-Fluorophenol | 0.02 | 0.19 | 0.21 | -0.17 |
| 333 | 2-Chloroanisole | 0.22 | 0.39 | 0.38 | -0.17 |
| 334 | 2-Bromo-4-methylphenol | 0.60 | 0.77 | 0.77 | -0.17 |
| 335 | 2-Phenyl-3-butyn-2-ol | -0.18 | -0.35 | -0.46 | 0.17 |
| 336 | 2,5-Dichlorophenol | 1.13 | 0.96 | 0.93 | 0.17 |
| 337 | Iodobenzene | 0.50 | 0.67 | 0.60 | -0.17 |
| 338 | 4,6-Dichlororesorcinol | 0.97 | 1.14 | 1.16 | -0.17 |
| 339 | 4-Pentyloxyaniline | 0.97 | 1.14 | 1.12 | -0.17 |
| 340 | 2,6-Dinitro-p-cresol | 1.23 | 1.40 | 1.38 | -0.17 |
| 341 | Pyridine | -1.32 | -1.14 | -1.15 | -0.18 |
| 342 | 2-Bromo-4,6-dinitroaniline | 1.24 | 1.42 | 1.44 | -0.18 |
| 343 | p-Dibromobenzene | 0.68 | 0.86 | 0.84 | -0.18 |
| 344 | 1,2,4-Benzenetriol | 0.44 | 0.26 | 0.23 | 0.18 |
| 345 | 3-Methyl-4-hydroxybenzaldehyde | -0.03 | 0.15 | 0.14 | -0.18 |
| 346 | 4-Methoxyphenol | -0.14 | 0.04 | 0.06 | -0.18 |
| 347 | Ethynylbenzene | -0.14 | 0.04 | 0.04 | -0.18 |
| 348 | Nitrobenzene | 0.14 | 0.32 | 0.30 | -0.18 |
| 349 | 2-Phenylpropionitrile | 0.01 | -0.17 | -0.23 | 0.18 |
| 350 | 2,5-Dimethylfuran | -0.48 | -0.66 | -0.94 | 0.18 |
| 351 | 1-Hexen-5-one | -1.14 | -0.96 | -0.95 | -0.18 |
| 352 | 4-Methyl-2-nitroaniline | 0.37 | 0.55 | 0.53 | -0.18 |
| 353 | 1,2-Pentanediol | -1.63 | -1.81 | -1.83 | 0.18 |
| 354 | 3-Chloro-5-methoxyphenol | 0.76 | 0.57 | 0.51 | 0.19 |
| 355 | 2,4-Dichloro-6-nitrophenol | 1.75 | 1.56 | 1.53 | 0.19 |
| 356 | 4-Propoxyphenol | 0.52 | 0.71 | 0.69 | -0.19 |
| 357 | 7-Tridecanone | 1.52 | 1.71 | 1.77 | -0.19 |
| 358 | n-Amyl ether | 1.09 | 0.90 |  | 0.19 |
| 359 | 2,3,4,5-Tetrachloroaniline | 1.96 | 1.77 | 1.73 | 0.19 |
| 360 | 3,3-Diphenyl-1-propanol | 1.04 | 1.23 | 1.22 | -0.19 |
| 361 | Heptanenitrile | -0.24 | -0.05 | -0.03 | -0.19 |
| 362 | 3-Fluorophenol | 0.38 | 0.19 | 0.16 | 0.19 |
| 363 | n-Hexylformate | -0.38 | -0.19 | -0.04 | -0.19 |
| 364 | Propyl butanoate | -0.41 | -0.22 | -0.19 | -0.19 |
| 365 | 1-Bromobutane | -0.18 | 0.01 | 0.07 | -0.19 |
| 366 | Cumic alcohol | 0.18 | -0.01 | -0.07 | 0.19 |
| 367 | 1,4-Dimethylnaphthalene | 1.43 | 1.24 | 1.24 | 0.19 |
| 368 | 1-Phenyl-1-propanol | -0.43 | -0.24 | -0.22 | -0.19 |
| 369 | 4-t-Butylbenzenemethanol | 0.48 | 0.29 | 0.31 | 0.19 |
| 370 | 2,3,4-Trichloroanisole | 1.64 | 1.45 | 1.42 | 0.19 |
| 371 | 3-Methyl-2-nitrophenol | 0.61 | 0.80 | 0.77 | -0.19 |
| 372 | 4-Butoxyaniline | 0.61 | 0.80 | 0.75 | -0.19 |
| 373 | 4-Methyl-1-pentanol | -0.64 | -0.83 | -0.79 | 0.19 |
| 374 | 4-Bromo-3,5-dimethyIphenol | 1.27 | 1.07 | 1.08 | 0.20 |
| 375 | 1-Propanol | -1.75 | -1.55 | -1.54 | -0.20 |
| 376 | 2-Benzylpyridine | 0.38 | 0.58 | 0.57 | -0.20 |
| 377 | 1,2-Hexanediol | -1.27 | -1.47 | -1.47 | 0.20 |
| 378 | 2,3,5,6-Tetrachlorophenol | 2.22 | 2.02 | 2.04 | 0.20 |
| 379 | Ethyl isovalerate | -0.72 | -0.52 | -0.48 | -0.20 |
| 380 | 3-Nitrotoluene | 0.42 | 0.62 | 0.60 | -0.20 |
| 381 | Isopentyl bromide | 0.25 | 0.05 | 0.00 | 0.20 |
| 382 | Hydrocinnamyl alcohol | -0.21 | -0.01 | -0.03 | -0.20 |
| 383 | 3-Buten-1-ol | -1.70 | -1.50 | -1.47 | -0.20 |
| 384 | 4-Hydroxy-2-methylacetophenone | 0.19 | -0.01 | -0.04 | 0.20 |
| 385 | 2-Phenyl-2-propanol | -0.57 | -0.37 | -0.30 | -0.20 |
| 386 | 1-Pentanol | -1.07 | -0.87 | -0.85 | -0.20 |
| 387 | 4-Hydroxy-3-methoxybenzonitrile | -0.03 | -0.23 | -0.21 | 0.20 |
| 388 | Di-n-propylsulfide | 0.00 | 0.20 | 0.20 | -0.20 |
| 389 | Di-n-butylsulfoxide | -0.26 | -0.46 |  | 0.20 |
| 390 | 1-Methylnaphthalene | 0.74 | 0.94 | 0.90 | -0.20 |
| 391 | Butylcellosolve | -1.37 | -1.17 |  | -0.20 |
| 392 | Benzyl 4-hydroxybenzoate | 1.55 | 1.34 | 1.32 | 0.21 |
| 393 | Butylbenzene | 1.25 | 1.04 | 1.04 | 0.21 |
| 394 | 4-Bromophenol | 0.68 | 0.47 | 0.42 | 0.21 |
| 395 | 4-Chloroanisole | 0.60 | 0.39 | 0.34 | 0.21 |
| 396 | Methyl-n-propylketone | -1.22 | -1.01 | -0.99 | -0.21 |
| 397 | Ethyl 4-methoxybenzoate | 0.31 | 0.10 | 0.10 | 0.21 |
| 398 | 4-Methylphenethyl alcohol | -0.26 | -0.05 | -0.07 | -0.21 |
| 399 | 4-Phenyl-1-butanol | 0.12 | 0.33 | 0.34 | -0.21 |
| 400 | 2-Amino-4-chloro-5-nitrophenol | 1.17 | 0.96 | 0.94 | 0.21 |
| 401 | 5-Methyl-2-nitrophenol | 0.59 | 0.80 | 0.77 | -0.21 |
| 402 | 2-Methylpropionamide | -1.88 | -1.66 | -1.69 | -0.22 |
| 403 | 2-Methyl-1-butanol | -0.95 | -1.17 | -1.14 | 0.22 |
| 404 | 2-Methyl-2-propanol | -1.79 | -1.57 | -1.57 | -0.22 |
| 405 | 1,2-Dichlorobenzene | 1.00 | 0.78 | 0.74 | 0.22 |
| 406 | 2-Bromo-2'-hydroxy-5'-nitroacetanilide | 0.87 | 0.65 |  | 0.22 |
| 407 | 4-Fluorobenzyl alcohol | -0.62 | -0.40 | -0.40 | -0.22 |
| 408 | 5-Methyl-5-hexen-2-one | -0.87 | -0.65 | -0.75 | -0.22 |
| 409 | 3-Hydroxydiphenylamine | 1.01 | 1.23 |  | -0.22 |
| 410 | Allyl heptanoate | 0.73 | 0.51 | 0.59 | 0.22 |
| 411 | 2-Bromobenzyl alcohol | 0.10 | -0.12 | -0.12 | 0.22 |
| 412 | 3-Nitroaniline | 0.03 | 0.25 | 0.23 | -0.22 |
| 413 | 1,3-Dichlorobenzene | 0.56 | 0.78 | 0.74 | -0.22 |
| 414 | Allylbenzene | 0.19 | 0.41 | 0.38 | -0.22 |
| 415 | 4-Acetylanisole | -0.13 | -0.35 | -0.38 | 0.22 |
| 416 | 1-Butanol | -1.43 | -1.21 | -1.19 | -0.22 |
| 417 | 1,4-Dinitrotetrachlorobenzene | 2.82 | 3.04 | 3.06 | -0.22 |
| 418 | 2-Isopropylphenol | 0.80 | 0.58 | 0.56 | 0.22 |
| 419 | 3,5-Di-t-butylphenol | 1.64 | 1.86 | 1.87 | -0.22 |
| 420 | 3,4-Dichloronitrobenzene | 1.16 | 1.38 | 1.35 | -0.22 |
| 421 | Methyl caprate | 1.38 | 1.15 | 1.13 | 0.23 |
| 422 | Methylpelargonate | 1.04 | 0.81 | 0.79 | 0.23 |
| 423 | Hexanamide | -0.91 | -0.68 | -0.74 | -0.23 |
| 424 | 2,6-Dichlorophenol | 0.73 | 0.96 | 0.93 | -0.23 |
| 425 | m-Toluidine | -0.28 | -0.05 | -0.06 | -0.23 |
| 426 | Methylbenzene | 0.25 | 0.02 | 0.04 | 0.23 |
| 427 | 3-Methoxysalicylaldehyde | 0.38 | 0.15 | 0.12 | 0.23 |
| 428 | Ethyl thiophene-2-carboxylate | 0.03 | -0.20 | -0.22 | 0.23 |
| 429 | trans-Cinnamyl acetate | 0.59 | 0.36 | 0.34 | 0.23 |
| 430 | 1,2-Dichloro-4,5-dinitrobenzene | 2.21 | 1.98 | 1.96 | 0.23 |
| 431 | 4-Methyl-2-nitrophenol | 0.57 | 0.80 | 0.79 | -0.23 |
| 432 | 4-Chloro-3-nitrophenol | 1.27 | 1.03 | 1.01 | 0.24 |
| 433 | Ethyl propionate | -1.14 | -0.90 | -0.87 | -0.24 |
| 434 | Allyl phenyl sulfide | 0.69 | 0.45 | 0.41 | 0.24 |
| 435 | 2-Methylbenzo[b]furane | 0.62 | 0.38 | 0.39 | 0.24 |
| 436 | Ethyl 4-fluorobenzoate | 0.01 | 0.25 | 0.27 | -0.24 |
| 437 | 4-Propylphenol | 0.64 | 0.88 | 0.90 | -0.24 |
| 438 | 2,2-Diphenylethanol | 0.65 | 0.89 | 0.91 | -0.24 |
| 439 | 4-Bromophenyl acetonitrile | 0.60 | 0.36 | 0.33 | 0.24 |
| 440 | Methyl isobutyl ketone | -1.21 | -0.97 | -0.94 | -0.24 |
| 441 | 1-Hexen-3-ol | -0.81 | -1.05 | -1.05 | 0.24 |
| 442 | Cyclohexylphenol | 1.56 | 1.80 | 1.77 | -0.24 |
| 443 | Undecylcyanide | 1.90 | 1.65 | 1.64 | 0.25 |
| 444 | 1-Iodo-2,4-dinitrobenzene | 2.12 | 1.87 | 1.78 | 0.25 |
| 445 | Hexyl bromide | 0.94 | 0.69 | 0.74 | 0.25 |
| 446 | 4-Bromo-2,6-dichlorophenol | 1.78 | 1.53 | 1.50 | 0.25 |
| 447 | Amyl formate | -0.78 | -0.53 | -0.48 | -0.25 |
| 448 | 2-Chlorophenol | 0.18 | 0.43 | 0.45 | -0.25 |
| 449 | Phenol | -0.35 | -0.10 | -0.08 | -0.25 |
| 450 | 1,1-Diphenyl-2-propanol | 0.75 | 1.00 | 1.01 | -0.25 |
| 451 | 2-Ethylpyridine | -0.87 | -0.62 | -0.64 | -0.25 |
| 452 | Hexamethyleneglycol | -1.49 | -1.24 | -1.22 | -0.25 |
| 453 | 2,6-Diisopropylaniline | 0.76 | 1.01 | 1.02 | -0.25 |
| 454 | 2-Vinylnaphthalene | 1.24 | 0.99 | 1.02 | 0.25 |
| 455 | 1-Hydroxyadamantane | -0.08 | 0.17 | 0.39 | -0.25 |
| 456 | 4-Decanol | 0.85 | 0.60 | 0.67 | 0.25 |
| 457 | 1,4-Dichlorobenzene | 0.53 | 0.78 | 0.76 | -0.25 |
| 458 | 3-Ethylphenol | 0.29 | 0.54 | 0.56 | -0.25 |
| 459 | n-Propylformate | -1.46 | -1.21 | -1.15 | -0.25 |
| 460 | 1,4-Dichloro-2-nitrobenzene | 1.13 | 1.38 | 1.39 | -0.25 |
| 461 | 3,5-Dichloronitrobenzene | 1.13 | 1.38 | 1.36 | -0.25 |
| 462 | 4-Bromo-2-fluoro-6-nitrophenol | 1.62 | 1.36 | 1.35 | 0.26 |
| 463 | 3-Butylthiophene | 0.96 | 0.70 | 0.10 | 0.26 |
| 464 | t-Butyl cyanide | -1.33 | -1.07 | -1.08 | -0.26 |
| 465 | n-Octylacetate | 1.06 | 0.80 | 0.79 | 0.26 |
| 466 | 2-Allylphenol | 0.33 | 0.59 | 0.60 | -0.26 |
| 467 | 4-Ethylaniline | 0.03 | 0.29 | 0.31 | -0.26 |
| 468 | 3-Nitroanisol | 0.72 | 0.46 | 0.44 | 0.26 |
| 469 | (2-Chloroethyl)benzene | 0.17 | 0.43 | 0.46 | -0.26 |
| 470 | Methyl 4-chlorobenzoate | 0.42 | 0.16 | 0.14 | 0.26 |
| 471 | 1-Chloropentane | 0.17 | -0.09 | -0.05 | 0.26 |
| 472 | 4-Hydroxybenzaldehyde | 0.27 | 0.01 | 0.00 | 0.26 |
| 473 | 1-Phenyl-2-butanol | -0.16 | 0.10 | 0.11 | -0.26 |
| 474 | 4-Chlorobenzophenone | 1.50 | 1.24 | 1.22 | 0.26 |
| 475 | Ethyl 4-chlorobenzoate | 0.75 | 0.49 | 0.50 | 0.26 |
| 476 | 2,5-Hexanediol | -1.96 | -1.70 | -1.66 | -0.26 |
| 477 | 1-Chlorooctane | 0.67 | 0.93 | 0.98 | -0.26 |
| 478 | 1-Bromo-4-chlorobenzene | 0.56 | 0.82 | 0.83 | -0.26 |
| 479 | 6-Chloro-2,4-dinitroaniline | 1.12 | 1.38 | 1.37 | -0.26 |
| 480 | 3-(Trifluoromethyl)-4-nitrophenol | 1.65 | 1.38 | 1.30 | 0.27 |
| 481 | 2-Fluorobenzyl alcohol | -0.67 | -0.40 | -0.40 | -0.27 |
| 482 | 3,5-Dimethoxyphenol | -0.09 | 0.18 | 0.27 | -0.27 |
| 483 | Methyl 4-hydroxybenzoate | 0.08 | -0.19 | -0.16 | 0.27 |
| 484 | (3-Chloropropyl)benzene | 0.50 | 0.77 | 0.78 | -0.27 |
| 485 | 3-Iodophenol | 1.12 | 0.85 | 0.82 | 0.27 |
| 486 | 2,6-Dinitrophenol | 0.83 | 1.10 | 1.07 | -0.27 |
| 487 | Pentafluorophenol | 1.63 | 1.35 | 1.29 | 0.28 |
| 488 | Phenyl benzoate | 1.35 | 1.07 | 1.09 | 0.28 |
| 489 | Acetamide | -2.32 | -2.04 | -2.10 | -0.28 |
| 490 | 2-Fluoro-4-nitrophenol | 1.07 | 0.79 | 0.77 | 0.28 |
| 491 | 5-Hydroxy-2-nitrobenzaldehyde | 0.33 | 0.61 | 0.61 | -0.28 |
| 492 | n-Butylbenzoate | 0.92 | 0.64 | 0.67 | 0.28 |
| 493 | 2-Propyl-1-pentanol | 0.13 | -0.15 | -0.12 | 0.28 |
| 494 | 1-Phenyl-2-pentanol | 0.16 | 0.44 | 0.46 | -0.28 |
| 495 | 5-Bromo-2-2-hydroxybenzyl alcohol | 0.34 | 0.06 | 0.04 | 0.28 |
| 496 | n-Butylformate | -1.15 | -0.87 | -0.74 | -0.28 |
| 497 | m-Cresol | -0.08 | 0.20 | 0.18 | -0.28 |
| 498 | Vinyl benzoate | 0.08 | -0.20 | 0.11 | 0.28 |
| 499 | Pentanenitrile | -1.01 | -0.73 | -0.68 | -0.28 |
| 500 | 5-Nonanone | 0.07 | 0.35 | 0.35 | -0.28 |
| 501 | 3,5-Dimethyl-1-hexyne-3-ol | -0.55 | -0.83 | -0.88 | 0.28 |
| 502 | 4-Hexylresorcinol | 1.80 | 2.08 | 2.17 | -0.28 |
| 503 | 4-t-Butyl-2,6-dinitrophenol | 1.80 | 2.08 | 2.06 | -0.28 |
| 504 | 2,6-Dibromo-4-nitrophenol | 1.36 | 1.64 | 1.64 | -0.28 |
| 505 | 4-Biphenylcarbonitrile | 1.24 | 0.95 | 0.88 | 0.29 |
| 506 | Cumene | 0.69 | 0.40 | 0.42 | 0.29 |
| 507 | 4,6-Dimethyl-2-pyrone | -0.93 | -0.64 | -0.51 | -0.29 |
| 508 | 4-Hydroxy-3-methoxybenzaldehyde | -0.14 | 0.15 | 0.15 | -0.29 |
| 509 | 4-Bromobenzonitrile | 0.31 | 0.02 | -0.03 | 0.29 |
| 510 | 1-Heptanol | 0.10 | -0.19 | -0.19 | 0.29 |
| 511 | Hydrocinnamonitrile | -0.16 | 0.13 | 0.17 | -0.29 |
| 512 | 1-Heptanethiol | 1.02 | 0.73 |  | 0.29 |
| 513 | Benzyl benzoate | 1.45 | 1.16 | 1.11 | 0.29 |
| 514 | 2-Dodecanone | 1.67 | 1.37 | 1.39 | 0.30 |
| 515 | 2,4,6-Tribromophenol | 1.91 | 1.61 | 1.56 | 0.30 |
| 516 | 4-Chloro-6-nitro-m-cresol | 1.63 | 1.33 | 1.32 | 0.30 |
| 517 | 2-Chloro-4-methylaniline | 0.18 | 0.48 | 0.50 | -0.30 |
| 518 | Methyl-p-toluate | 0.23 | -0.07 | -0.03 | 0.30 |
| 519 | 3-Hydroxyacetanilide | -0.16 | -0.46 | -0.60 | 0.30 |
| 520 | 1-Naphthaleneethanol | 0.27 | 0.57 | 0.54 | -0.30 |
| 521 | 2-Nonanone | 0.66 | 0.35 | 0.34 | 0.31 |
| 522 | 4-Allyl-2-methoxyphenol | 0.42 | 0.73 | 0.73 | -0.31 |
| 523 | 3-Cyanophenol | -0.06 | -0.37 | -0.38 | 0.31 |
| 524 | Ethyl hexanoate | -0.19 | 0.12 | 0.19 | -0.31 |
| 525 | 3-Chlorobenzylalcohol | 0.15 | -0.16 | -0.18 | 0.31 |
| 526 | 3-Chlorobenzophenone | 1.55 | 1.24 | 1.23 | 0.31 |
| 527 | Benzo[b]furan | -0.11 | 0.20 | 0.46 | -0.31 |
| 528 | Naphthalene | 0.33 | 0.64 | 0.68 | -0.31 |
| 529 | Methyl 4-t-butylbenzoate | 0.92 | 0.61 | 0.59 | 0.31 |
| 530 | Quinoline | 0.08 | 0.39 | 0.39 | -0.31 |
| 531 | 2,4-Dimethyl-3-pentanol | -0.71 | -1.02 | -1.03 | 0.31 |
| 532 | 2,3-Dichloronitrobenzene | 1.07 | 1.38 | 1.38 | -0.31 |
| 533 | 2-Phenylphenol | 1.09 | 1.40 | 1.35 | -0.31 |
| 534 | 4,6-Dinitro-o-cresol | 1.72 | 1.40 | 1.45 | 0.32 |
| 535 | 3-Ethylaniline | -0.03 | 0.29 | 0.28 | -0.32 |
| 536 | 6-t-Butyl-2,4-dimethylphenol | 1.16 | 1.48 | 1.53 | -0.32 |
| 537 | 2-Ethyl-1-hexanol | 0.17 | -0.15 | -0.13 | 0.32 |
| 538 | 2-Hydroxy-5-methylacetophenone | 0.31 | -0.01 | -0.03 | 0.32 |
| 539 | Hexanophenone | 1.19 | 0.87 | 0.89 | 0.32 |
| 540 | Methyl 2-methylbutyrate | -1.17 | -0.85 | -0.84 | -0.32 |
| 541 | 2,3-Dichlorophenol | 1.28 | 0.96 | 0.97 | 0.32 |
| 542 | Tetramethylene glycol | -2.24 | -1.92 | -1.88 | -0.32 |
| 543 | 1,3,5-Trichloro-2,4-dinitrobenzene | 2.19 | 2.51 | 2.48 | -0.32 |
| 544 | Ethyl levulinate | -1.40 | -1.07 | -1.05 | -0.33 |
| 545 | o-Dinitrobenzene | 1.25 | 0.92 | 0.91 | 0.33 |
| 546 | Phenethyl alcohol | -0.68 | -0.35 | -0.34 | -0.33 |
| 547 | 4-Hydroxy-3-methoxybenzylalcohol | -0.70 | -0.37 | -0.42 | -0.33 |
| 548 | 4-Phenylbutyronitrile | 0.14 | 0.47 | 0.50 | -0.33 |
| 549 | 4-Ethylphenol | 0.21 | 0.54 | 0.55 | -0.33 |
| 550 | 1,2-Dimethyl-4-nitrobenzene | 0.59 | 0.92 | 0.89 | -0.33 |
| 551 | 4-Chloro-2-nitrotoluene | 0.82 | 1.15 | 1.12 | -0.33 |
| 552 | 2,4-Dibromo-6-phenylphenol | 2.21 | 2.54 | 2.62 | -0.33 |
| 553 | Isovaleramide | -1.66 | -1.32 | -1.35 | -0.34 |
| 554 | Undecanenitrile | 1.65 | 1.31 | 1.37 | 0.34 |
| 555 | Octanophenone | 1.89 | 1.55 | 1.50 | 0.34 |
| 556 | Neopentyl alcohol | -0.87 | -1.21 | -1.30 | 0.34 |
| 557 | 5-Fluoro-2-nitrophenol | 1.13 | 0.79 | 0.77 | 0.34 |
| 558 | 2-Chloro-5-methylphenol | 0.39 | 0.73 | 0.74 | -0.34 |
| 559 | 4-Heptanone | -0.67 | -0.33 | -0.34 | -0.34 |
| 560 | 3-Hydroxy-4-nitrobenzaldehyde | 0.27 | 0.61 | 0.66 | -0.34 |
| 561 | Ethyl 3-hydroxybenzoate | 0.48 | 0.14 | 0.11 | 0.34 |
| 562 | 2-Hydroxybenzophenone | 1.23 | 0.89 | 0.89 | 0.34 |
| 563 | 4-(4-Bromophenyl)phenol | 2.31 | 1.97 | 1.92 | 0.34 |
| 564 | 2-Chloro-4,5-dimethylphenol | 0.69 | 1.03 | 1.01 | -0.34 |
| 565 | 3-Chloro-4-fluoro-1-nitrobenzene | 0.80 | 1.14 | 1.14 | -0.34 |
| 566 | 1,5-Pentanediol | -1.93 | -1.58 | -1.53 | -0.35 |
| 567 | 2,4-Dinitrotoluene | 0.87 | 1.22 | 1.22 | -0.35 |
| 568 | 3-Aminophenol | -0.52 | -0.17 | -0.22 | -0.35 |
| 569 | 2-Chloroaniline | -0.17 | 0.18 | 0.19 | -0.35 |
| 570 | 4-Hydroxyphenylacetonitrile | -0.38 | -0.03 | 0.00 | -0.35 |
| 571 | Methylcaproate | -0.56 | -0.21 | -0.16 | -0.35 |
| 572 | Heptanophenone | 1.56 | 1.21 | 1.19 | 0.35 |
| 573 | 4-Butoxyphenol | 0.70 | 1.05 | 1.02 | -0.35 |
| 574 | 2,4-Dibromophenol | 1.40 | 1.04 | 0.99 | 0.36 |
| 575 | Paracetamol | -0.82 | -0.46 |  | -0.36 |
| 576 | Butyric acid methylester | -1.25 | -0.89 | -0.86 | -0.36 |
| 577 | 2-Methylbenzophenone | 1.37 | 1.01 | 1.00 | 0.36 |
| 578 | 2-Nonanol | 0.62 | 0.26 | 0.31 | 0.36 |
| 579 | 2-Nitrotoluene | 0.26 | 0.62 | 0.59 | -0.36 |
| 580 | 4-Ethoxyphenol | 0.01 | 0.37 | 0.34 | -0.36 |
| 581 | 2,4-Xylenol | 0.14 | 0.50 | 0.49 | -0.36 |
| 582 | 4-tert-Butylpyridine | 0.20 | -0.16 | -0.18 | 0.36 |
| 583 | Salicylic acid | -0.51 | -0.15 | -0.09 | -0.36 |
| 584 | p-Cresol | -0.16 | 0.20 | 0.19 | -0.36 |
| 585 | Trimethylhydroquinone | 1.34 | 0.98 | 0.96 | 0.36 |
| 586 | 1,2-Dimethyl-3-nitrobenzene | 0.56 | 0.92 | 0.95 | -0.36 |
| 587 | 2,6-Di-t-butyl-4-methylphenol | 1.80 | 2.16 | 2.06 | -0.36 |
| 588 | 1,2-Diphenyl-2-propanol | 0.80 | 1.17 | 1.13 | -0.37 |
| 589 | Butyronitrile | -1.44 | -1.07 | -1.07 | -0.37 |
| 590 | 4-Dodecanol | 1.65 | 1.28 | 1.27 | 0.37 |
| 591 | Ethyl 2-methylbutyrate | -0.89 | -0.52 | -0.51 | -0.37 |
| 592 | 2,4-Diaminophenol | 0.13 | -0.24 | -0.34 | 0.37 |
| 593 | 3-Aminobenzylalcohol | -1.13 | -0.76 | -0.74 | -0.37 |
| 594 | (2-Bromoethyl)benzene | 0.50 | 0.87 | 0.89 | -0.37 |
| 595 | 3-Methoxyphenol | -0.33 | 0.04 | 0.10 | -0.37 |
| 596 | Methyl phenylacetate | -0.40 | -0.03 | 0.01 | -0.37 |
| 597 | 1-Nonanol | 0.86 | 0.49 | 0.50 | 0.37 |
| 598 | Isobutyronitrile | -1.75 | -1.37 | -1.39 | -0.38 |
| 599 | Cyclohexanol | -0.94 | -0.56 | -0.53 | -0.38 |
| 600 | Ethyl 3-pyridylacetate | -0.94 | -0.56 | -0.52 | -0.38 |
| 601 | p-Dinitrobenzene | 1.30 | 0.92 | 0.92 | 0.38 |
| 602 | 2-Dodecanol | 1.66 | 1.28 | 1.26 | 0.38 |
| 603 | 3-Methylthiophene | -0.70 | -0.32 | -0.23 | -0.38 |
| 604 | Benzyl-t-butanol | -0.07 | 0.31 | 0.22 | -0.38 |
| 605 | 2,3-Xylenol | 0.12 | 0.50 | 0.52 | -0.38 |
| 606 | 3,4-Xylenol | 0.12 | 0.50 | 0.51 | -0.38 |
| 607 | Chlorobenzene | -0.13 | 0.25 | 0.27 | -0.38 |
| 608 | 1-Phenyl-2-methyl-2-propanol | -0.41 | -0.03 | -0.12 | -0.38 |
| 609 | Amyl acetate | 0.16 | -0.22 | -0.20 | 0.38 |
| 610 | 2-Methylcyclohexanone | -0.81 | -0.43 | -0.42 | -0.38 |
| 611 | 1-Phenyl-2-propanol | -0.62 | -0.24 | -0.25 | -0.38 |
| 612 | Propionic acid methylester | -1.61 | -1.23 | -1.20 | -0.38 |
| 613 | Phlorol | 0.16 | 0.54 | 0.58 | -0.38 |
| 614 | 2,4,6-Trimethylphenol | 0.42 | 0.80 | 0.83 | -0.38 |
| 615 | Methyl formate | -1.50 | -1.88 | -1.92 | 0.38 |
| 616 | 1,2,4-Trichloro-5-nitrobenzene | 1.53 | 1.91 | 1.91 | -0.38 |
| 617 | 2-Hydroxy-4-methoxybenzophenone | 1.42 | 1.03 | 1.14 | 0.39 |
| 618 | Methyl 2,2-dimethylpropanoate | -1.28 | -0.89 | -0.83 | -0.39 |
| 619 | 4-Methylbenzophenone | 1.40 | 1.01 | 0.96 | 0.39 |
| 620 | 2-Hydroxyacetophenone | 0.08 | -0.31 | -0.32 | 0.39 |
| 621 | Hydroquinone | 0.47 | 0.08 | 0.06 | 0.39 |
| 622 | 3,5-Xylenol | 0.11 | 0.50 | 0.52 | -0.39 |
| 623 | Butyl propanoate | 0.17 | -0.22 | -0.23 | 0.39 |
| 624 | 2,3,4,6-Tetrafluoronitrobenzene | 1.87 | 1.48 | 1.45 | 0.39 |
| 625 | n-Amyl benzoate | 1.37 | 0.98 | 1.01 | 0.39 |
| 626 | 2-Decanol | 0.99 | 0.60 | 0.59 | 0.39 |
| 627 | 1-Ethylnaphthalene | 0.89 | 1.28 | 1.28 | -0.39 |
| 628 | 2,4-Dichloronitrobenzene | 0.99 | 1.38 | 1.39 | -0.39 |
| 629 | 2-Butanone | -1.75 | -1.35 | -1.35 | -0.40 |
| 630 | Methyl 4-bromobenzoate | 0.60 | 0.20 | 0.22 | 0.40 |
| 631 | 2-Hydroxybenzonitrile | 0.03 | -0.37 | -0.40 | 0.40 |
| 632 | 3-Nitrobenzonitrile | 0.45 | 0.05 | 0.07 | 0.40 |
| 633 | 1-Phenyl-2-butanone | -0.21 | 0.19 | 0.26 | -0.40 |
| 634 | Butyl butanoate | 0.52 | 0.12 | 0.13 | 0.40 |
| 635 | n-Decylacetate | 1.88 | 1.48 | 1.51 | 0.40 |
| 636 | 3-Methyl-2-butanol | -1.00 | -1.40 | -1.42 | 0.40 |
| 637 | 1,2,3-Trichloro-4-nitrobenzene | 1.51 | 1.91 | 1.89 | -0.40 |
| 638 | Amylbenzene | 1.79 | 1.38 | 1.33 | 0.41 |
| 639 | o-Toluamide | -0.95 | -0.54 | -0.56 | -0.41 |
| 640 | 2-t-Butylphenol | 1.29 | 0.88 | 0.88 | 0.41 |
| 641 | 2-Hydroxybenzaldehyde | 0.42 | 0.01 | -0.04 | 0.41 |
| 642 | 3,4-Xylidine | -0.16 | 0.25 | 0.21 | -0.41 |
| 643 | 1-Cyclohexen-1-yl acetate | -0.40 | 0.01 | 0.04 | -0.41 |
| 644 | 4-Chlorobenzylalcohol | 0.25 | -0.16 | -0.17 | 0.41 |
| 645 | Ethyl isobutyrate | -1.27 | -0.86 | -0.89 | -0.41 |
| 646 | Isobutyl acetate | -1.27 | -0.86 | -0.83 | -0.41 |
| 647 | 3-Chloro-4-fluorophenol | 1.13 | 0.72 | 0.71 | 0.41 |
| 648 | 4-Bromotoluene | 1.00 | 0.59 | 0.55 | 0.41 |
| 649 | 2-Tridecanone | 2.12 | 1.71 | 1.70 | 0.41 |
| 650 | Phenylhydroquinone | 2.00 | 1.58 | 1.56 | 0.42 |
| 651 | Propyl propanoate | -0.98 | -0.56 | -0.55 | -0.42 |
| 652 | Methylvalerate | -0.97 | -0.55 | -0.57 | -0.42 |
| 653 | 4-Chloronitrobenzene | 0.43 | 0.85 | 0.84 | -0.42 |
| 654 | Ethyl 4-methylbenzoate | 0.68 | 0.26 | 0.23 | 0.42 |
| 655 | 2,5-Xylenol | 0.08 | 0.50 | 0.49 | -0.42 |
| 656 | 2-Hydroxy-5-chloroaniline | 0.78 | 0.36 | 0.33 | 0.42 |
| 657 | 2-Hydroxy-4,5-dimethylacetophenone | 0.71 | 0.29 | 0.27 | 0.42 |
| 658 | 2-Hydroxybenzamide | -0.24 | -0.66 | -0.64 | 0.42 |
| 659 | 3,5-Diiodosalicylaldehyde | 2.34 | 1.91 | 1.70 | 0.43 |
| 660 | Butyramide | -1.79 | -1.36 | -1.35 | -0.43 |
| 661 | Methyl acetate | -2.00 | -1.57 | -1.51 | -0.43 |
| 662 | 2-Chloro-5-nitrobenzaldehyde | 0.53 | 0.96 | 0.90 | -0.43 |
| 663 | n-Propyl acetate | -1.33 | -0.90 | -0.87 | -0.43 |
| 664 | 2-Methylcyclopentanone | -1.20 | -0.77 | -0.72 | -0.43 |
| 665 | Ethyl 4-hydroxybenzoate | 0.57 | 0.14 | 0.12 | 0.43 |
| 666 | 2-Hydroxydiphenylmethane | 1.31 | 1.74 | 1.72 | -0.43 |
| 667 | Pinacolone | -1.44 | -1.01 | -0.99 | -0.43 |
| 668 | 5-Pentylresorcinol | 1.31 | 1.74 | 1.75 | -0.43 |
| 669 | 2-Hydroxybenzylalcohol | -0.95 | -0.51 | -0.52 | -0.44 |
| 670 | 3-Chlorophenol | 0.87 | 0.43 | 0.45 | 0.44 |
| 671 | 1,5-Dichloro-2,3-dinitrobenzene | 2.42 | 1.98 | 1.94 | 0.44 |
| 672 | 5-Hexyn-1-ol | -1.29 | -0.85 | -0.88 | -0.44 |
| 673 | 2,3,5-Trimethylphenol | 0.36 | 0.80 | 0.81 | -0.44 |
| 674 | 1,3,5-Trichlorobenzene | 0.87 | 1.31 | 1.34 | -0.44 |
| 675 | 2-Amino-4-t-butylphenol | 0.37 | 0.81 | 0.83 | -0.44 |
| 676 | Chlorothymol | 1.85 | 1.41 | 1.41 | 0.44 |
| 677 | Methanol | -2.67 | -2.22 | -2.16 | -0.45 |
| 678 | 4-Nonylphenol | 2.47 | 2.92 | 3.03 | -0.45 |
| 679 | Diethylketone | -1.46 | -1.01 | -0.94 | -0.45 |
| 680 | 2,6-Dichloro-4-fluorophenol | 0.80 | 1.25 | 1.22 | -0.45 |
| 681 | 4-Amino-2-nitrophenol | 0.88 | 0.43 | 0.42 | 0.45 |
| 682 | 4-Chlorocatecol | 1.06 | 0.61 | 0.61 | 0.45 |
| 683 | Isopropylacetate | -1.59 | -1.13 | -1.08 | -0.46 |
| 684 | 3,3'-Bithiophene | 1.00 | 0.54 | -0.74 | 0.46 |
| 685 | Heptyl bromide | 1.49 | 1.03 | 1.02 | 0.46 |
| 686 | 3-Ethoxy-4-hydroxybenzaldehyde | 0.02 | 0.48 | 0.58 | -0.46 |
| 687 | 6-Amino-2,4-dimethylphenol | 0.89 | 0.43 | 0.41 | 0.46 |
| 688 | Bromobenzene | 0.75 | 0.29 | 0.23 | 0.46 |
| 689 | Pentafluorobenzaldehyde | 0.82 | 1.28 | 1.32 | -0.46 |
| 690 | Acetone | -2.15 | -1.69 | -1.62 | -0.46 |
| 691 | 2,2',4,4'-Tetrahydroxybenzophenone | 0.96 | 1.43 | 1.48 | -0.47 |
| 692 | 1-Bromo-4-fluorobenzene | 0.11 | 0.58 | 0.58 | -0.47 |
| 693 | 4-Chlorobenzamide | 0.16 | -0.31 | -0.34 | 0.47 |
| 694 | 1-Fluoro-3-iodo-5-nitrobenzene | 1.09 | 1.56 | 1.53 | -0.47 |
| 695 | 2-Chloro-6-nitrotoluene | 0.68 | 1.15 | 1.12 | -0.47 |
| 696 | 3,5-Dichlorosalicylaldehyde | 1.55 | 1.07 | 1.03 | 0.48 |
| 697 | Ethyl 4-t-butylbenzoate | 1.42 | 0.94 | 0.87 | 0.48 |
| 698 | 4-Chlororesorcinol | 0.13 | 0.61 | 0.62 | -0.48 |
| 699 | Methyl 4-ethoxybenzoate | 0.58 | 0.10 | 0.09 | 0.48 |
| 700 | 5-Amino-2-methoxyphenol | 0.45 | -0.03 | -0.10 | 0.48 |
| 701 | 1-Chlorobutane | -0.91 | -0.43 | -0.40 | -0.48 |
| 702 | 1,3,5-Trichloro-2-nitrobenzene | 1.43 | 1.91 | 1.90 | -0.48 |
| 703 | Pentachlorophenol | 2.07 | 2.55 | 2.57 | -0.48 |
| 704 | 3,5-Dibromosalicylaldehyde | 1.64 | 1.15 | 1.24 | 0.49 |
| 705 | 4-Nitrobenzyl chloride | 1.18 | 0.69 | 0.64 | 0.49 |
| 706 | 4-Nitrocatechol | 1.17 | 0.68 | 0.71 | 0.49 |
| 707 | Suberonitrile | -0.77 | -0.28 | -0.20 | -0.49 |
| 708 | 3-Bromobenzyl alcohol | 0.37 | -0.12 | -0.10 | 0.49 |
| 709 | o-Cresol | -0.29 | 0.20 | 0.20 | -0.49 |
| 710 | 2-Hydroxyethyl salicylate | -0.08 | -0.57 | -0.60 | 0.49 |
| 711 | 1-Bromooctane | 1.87 | 1.37 | 1.31 | 0.50 |
| 712 | 2-Undecanone | 1.53 | 1.03 | 1.02 | 0.50 |
| 713 | 1-Indanol | -0.58 | -0.08 | -0.04 | -0.50 |
| 714 | Diethyl sulphide | -0.98 | -0.48 | -0.37 | -0.50 |
| 715 | Sanger's Reagent | 1.71 | 1.21 | 1.19 | 0.50 |
| 716 | Tetrachlorocatechol | 1.70 | 2.20 | 2.20 | -0.50 |
| 717 | n-Decyl alcohol | 1.34 | 0.83 | 0.85 | 0.51 |
| 718 | Butyl 4-hydroxybenzoate | 1.33 | 0.82 | 0.82 | 0.51 |
| 719 | 2-Ethylaniline | -0.22 | 0.29 | 0.28 | -0.51 |
| 720 | 4-Fluoronitrobenzene | 0.10 | 0.61 | 0.61 | -0.51 |
| 721 | Benzylamine | -0.24 | -0.75 |  | 0.51 |
| 722 | Myristyl alcohol | 1.67 | 2.19 | 2.19 | -0.52 |
| 723 | (3-Bromopropyl)benzene | 0.69 | 1.21 | 1.24 | -0.52 |
| 724 | 1-Octanol | 0.67 | 0.15 | 0.21 | 0.52 |
| 725 | 1-Bromo-4-t-butylbenzene | 0.75 | 1.27 | 1.31 | -0.52 |
| 726 | 2-Bromoethylphenyl sulfide | 0.39 | 0.91 | 1.03 | -0.52 |
| 727 | 2,3,6-Trimethylphenol | 0.28 | 0.80 | 0.79 | -0.52 |
| 728 | 3-Hydroxybenzylalcohol | -1.04 | -0.51 | -0.53 | -0.53 |
| 729 | 2-Amino-5-chlorobenzonitrile | 0.44 | -0.09 | -0.14 | 0.53 |
| 730 | 1-Chlorohexane | 0.78 | 0.25 | 0.25 | 0.53 |
| 731 | Acetonitrile | -2.28 | -1.75 | -1.77 | -0.53 |
| 732 | 4-Benzyloxyphenol | 1.04 | 1.57 | 1.54 | -0.53 |
| 733 | 4-Hydroxy-3-methoxybenzylamine | -0.97 | -0.43 |  | -0.54 |
| 734 | Hexane | 0.72 | 0.18 | 0.24 | 0.54 |
| 735 | 1-Fluoronaphthalene | 0.39 | 0.93 | 0.95 | -0.54 |
| 736 | 2-Tridecanol | 2.17 | 1.62 | 1.65 | 0.55 |
| 737 | 2-Methoxyphenol | -0.51 | 0.04 | 0.06 | -0.55 |
| 738 | Methyl trans-3-pentenoate | -0.28 | -0.83 | -0.87 | 0.55 |
| 739 | 2-Chloro-4-nitrophenol | 1.59 | 1.03 | 1.03 | 0.56 |
| 740 | Propanenitrile | -1.97 | -1.41 | -1.38 | -0.56 |
| 741 | t-Butylacetate | -1.49 | -0.92 | -0.83 | -0.57 |
| 742 | 1-Iodonaphthalene | 2.17 | 1.59 | 1.54 | 0.58 |
| 743 | 3,4-Dinitrobenzyl alcohol | 1.09 | 0.51 | 0.49 | 0.58 |
| 744 | 1,5-Difluoro-2,4-dinitrobenzene | 2.08 | 1.50 | 1.50 | 0.58 |
| 745 | 1-(Chloromethyl)naphthalene | 0.43 | 1.01 | 0.98 | -0.58 |
| 746 | Adiponitrile | -1.54 | -0.96 | -0.87 | -0.58 |
| 747 | Disophenol | 1.81 | 2.40 | 2.60 | -0.59 |
| 748 | 4-Fluoro-2-nitrophenol | 1.38 | 0.79 | 0.77 | 0.59 |
| 749 | 1,2,3-Benzenetriol | 0.85 | 0.26 | 0.31 | 0.59 |
| 750 | 1-Tridecanol | 2.45 | 1.85 | 1.87 | 0.60 |
| 751 | Methyl benzoate | 0.23 | -0.37 | -0.42 | 0.60 |
| 752 | Tetrafluorohydroquinone | 1.84 | 1.24 | 1.26 | 0.60 |
| 753 | 3,5-Dichlorophenol | 1.56 | 0.96 | 0.97 | 0.60 |
| 754 | 2-Indanol | -0.69 | -0.08 | -0.07 | -0.61 |
| 755 | 2,4,5-Trichlorophenol | 2.10 | 1.49 | 1.44 | 0.61 |
| 756 | 6-Tridecanol | 2.24 | 1.62 | 1.65 | 0.62 |
| 757 | n-Hexyl benzoate | 1.94 | 1.32 | 1.29 | 0.62 |
| 758 | 2,3,5,6-Tetrachloronitrobenzene | 1.82 | 2.44 | 2.47 | -0.62 |
| 759 | Ethyl 4-iodobenzoate | 1.53 | 0.91 | 0.86 | 0.62 |
| 760 | 3-Hydroxy-4-methoxybenzylalcohol | -0.99 | -0.37 | -0.37 | -0.62 |
| 761 | Isoamyl-4-hydroxybenzoate | 1.48 | 0.86 | 0.85 | 0.62 |
| 762 | 2,6-Diethylaniline | 0.31 | 0.93 | 0.95 | -0.62 |
| 763 | 2-Ethylhexyl 4'-hydroxybenzoate | 2.51 | 1.88 | 1.89 | 0.63 |
| 764 | 2-Ethylthiophene | -0.43 | 0.20 | 0.34 | -0.63 |
| 765 | 1-Phenyl-1,2-propanedione-2-oxime | -0.04 | -0.67 | -0.92 | 0.63 |
| 766 | 2-Undecanol | 1.58 | 0.94 | 1.01 | 0.64 |
| 767 | Isobutyl benzoate | 0.98 | 0.34 | 0.27 | 0.64 |
| 768 | 2,3-Dinitrophenol | 0.46 | 1.10 | 1.07 | -0.64 |
| 769 | 1-Dodecanol | 2.16 | 1.51 | 1.51 | 0.65 |
| 770 | Chlorohydroquinone | 1.26 | 0.61 | 0.58 | 0.65 |
| 771 | 1-Bromonaphthalene | 1.86 | 1.21 | 1.09 | 0.65 |
| 772 | 2,3,4,5-Tetrachloronitrobenzene | 1.78 | 2.44 | 2.45 | -0.66 |
| 773 | Pentafluoronitrobenzene | 2.43 | 1.77 | 1.68 | 0.66 |
| 774 | Cyclohexanone oxime | -0.80 | -0.14 | 0.15 | -0.66 |
| 775 | 4-Hydroxyphenethylalcohol | -0.83 | -0.17 | -0.16 | -0.66 |
| 776 | 3-Carboxyphenol | -0.81 | -0.15 | -0.06 | -0.66 |
| 777 | 2-Hexanone | -1.34 | -0.67 | -0.65 | -0.67 |
| 778 | Pyrocatechol | 0.75 | 0.08 | 0.02 | 0.67 |
| 779 | Methyl 4-methoxysalicylate | 0.62 | -0.05 | -0.07 | 0.67 |
| 780 | 3-Bromophenol | 1.15 | 0.47 | 0.44 | 0.68 |
| 781 | 2,3,4,5-Tetrachlorophenol | 2.71 | 2.02 | 1.97 | 0.69 |
| 782 | 1,2,3-Trifluoro-4-nitrobenzene | 1.89 | 1.19 | 1.18 | 0.70 |
| 783 | 2,2'-Biphenol | 0.88 | 1.58 | 1.56 | -0.70 |
| 784 | 1-Chloro-2,4-dinitrobenzene | 2.16 | 1.45 | 1.44 | 0.71 |
| 785 | Peonol | 0.55 | -0.17 | -0.17 | 0.72 |
| 786 | 2,3-Dimethylhydroquinone | 1.41 | 0.68 | 0.68 | 0.73 |
| 787 | Benzene | -1.01 | -0.28 | -0.34 | -0.73 |
| 788 | 2-Ethoxyphenol | -0.36 | 0.37 | 0.44 | -0.73 |
| 789 | Resorcinol | -0.65 | 0.08 | 0.08 | -0.73 |
| 790 | 2,4,6-Tribromoresorcinol | 1.06 | 1.79 | 1.83 | -0.73 |
| 791 | Cyclohexanone | -1.23 | -0.47 | -0.42 | -0.76 |
| 792 | 5-Methylresorcinol | -0.39 | 0.38 | 0.42 | -0.77 |
| 793 | 1-Chloroheptane | 1.36 | 0.59 | 0.57 | 0.77 |
| 794 | 2,6-Dimethoxyphenol | -0.60 | 0.18 | 0.19 | -0.78 |
| 795 | 1-Undecanol | 1.95 | 1.17 | 1.18 | 0.78 |
| 796 | 2,6-Diphenylphenol | 2.11 | 2.90 | 2.93 | -0.79 |
| 797 | Vinyl 4-t-butyl benzoate | 1.57 | 0.78 | 0.55 | 0.79 |
| 798 | 3,4-Dichlorophenol | 1.75 | 0.96 | 0.93 | 0.79 |
| 799 | 4-Bromo-2-nitrophenol | 1.87 | 1.07 | 1.05 | 0.80 |
| 800 | Ethyl 4-bromobenzoate | 1.33 | 0.53 | 0.50 | 0.80 |
| 801 | Thiophene | -1.44 | -0.63 | -0.34 | -0.81 |
| 802 | 3--Ethoxy-4-methoxyphenol | -0.30 | 0.51 | 0.53 | -0.81 |
| 803 | t-Butylformate | -0.41 | -1.23 | -1.42 | 0.82 |
| 804 | 1-Bromo-2,4-dinitrobenzene | 2.31 | 1.49 | 1.50 | 0.82 |
| 805 | 3,4-Dinitrophenol | 0.27 | 1.10 | 1.11 | -0.83 |
| 806 | Pentafluoroaniline | 0.26 | 1.10 | 1.18 | -0.84 |
| 807 | Ethyl-4-hydroxy-3-methoxyphenylacetate | -0.23 | 0.62 | 0.69 | -0.85 |
| 808 | delta-Decanolactone | -0.08 | 0.77 | 0.87 | -0.85 |
| 809 | Benzyl-4-hydroxyphenyl ketone | -0.50 | 0.37 | 0.45 | -0.87 |
| 810 | 1,2-Decanediol | 0.76 | -0.11 | -0.13 | 0.87 |
| 811 | 4-Carboxyphenol | -1.02 | -0.15 | -0.06 | -0.87 |
| 812 | 2,3,5-Trichlorophenol | 2.37 | 1.49 | 1.44 | 0.88 |
| 813 | 4-Bromoiodobenzene | 0.35 | 1.24 | 1.33 | -0.89 |
| 814 | 4-Cyanophenol | 0.52 | -0.37 | -0.37 | 0.89 |
| 815 | Caprolactone | -1.26 | -0.36 | -0.27 | -0.90 |
| 816 | 4-Nitrophenol | 1.42 | 0.50 | 0.47 | 0.92 |
| 817 | 3-Methyl-4-nitrophenol | 1.73 | 0.80 | 0.77 | 0.93 |
| 818 | 2,6-Dichloro-4-nitrophenol | 0.63 | 1.56 | 1.56 | -0.93 |
| 819 | Caprylic acid vinylester | -0.30 | 0.64 | 1.01 | -0.94 |
| 820 | Pentafluorobenzyl alcohol | -0.20 | 0.76 | 0.85 | -0.96 |
| 821 | 4-Fluorobiphenyl | 0.53 | 1.51 | 1.50 | -0.98 |
| 822 | 4-Amino-2,3-dimethylphenol | 1.44 | 0.43 | 0.38 | 1.01 |
| 823 | 4-Chloro-2-nitrophenol | 2.05 | 1.03 | 1.01 | 1.02 |
| 824 | Bromohydroquinone | 1.68 | 0.65 | 0.67 | 1.03 |
| 825 | Propanamide | -0.66 | -1.70 | -1.76 | 1.04 |
| 826 | 2-Aminophenol | 0.94 | -0.17 | -0.23 | 1.11 |
| 827 | 4-Amino-2-cresol | 1.31 | 0.13 | 0.11 | 1.18 |
| 828 | Tetrabromocatechol | 0.98 | 2.36 | 2.62 | -1.38 |
| 829 | Allyl decanoate | 0.48 | 1.87 | 1.99 | -1.39 |
| 830 | Methylhydroquinone | 1.86 | 0.38 | 0.35 | 1.48 |
| 831 | 4-Chloro-3,5-dinitrobenzonitrile | 2.66 | 1.18 | 1.17 | 1.48 |
| 832 | Phloroglucinol | -1.26 | 0.26 | 0.36 | -1.52 |
| 833 | Allyl acetate | 0.48 | -1.19 | -1.22 | 1.67 |
| 834 | 4-Hydroxyphenylacetic acid | -1.50 | 0.19 | 0.21 | -1.69 |
| 835 | 2,4,6-Trinitrophenol | -0.16 | 1.70 | 1.76 | -1.86 |
| 836 | Methoxyhydroquinone | 2.20 | 0.22 | 0.16 | 1.98 |
