# Supplementary material for: A Generally Applicable Computer Algorithm Based on the Group Additivity Method for the Calculation of Seven Molecular Descriptors: Heat of Combustion, LogPO/W, LogS, Refractivity, Polarizability, Toxicity and LogBB of Organic Compounds; Scope and Limits of Applicability
Source: Molecules. 2015 Oct 7;20(10):18279–351. doi: 10.3390/molecules201018279 (PMC6332030; doi:10.3390/molecules201018279)
Supplement: Supplementary file 1 [file molecules-20-18279-s001.zip › LogBB Parameters Table.doc]

**LogBB Parameters Table**

|  | **Atom type** | **Neighbours** | **Contribution** | **Occurrences** | **Molecules** |
| --- | --- | --- | --- | --- | --- |
| 1 | Const |  | 0.21 | 486 | 486 |
| 2 | C sp3 | H3C | 0.06 | 519 | 255 |
| 3 | C sp3 | H3N | 0.08 | 160 | 110 |
| 4 | C sp3 | H3O | -0.25 | 85 | 66 |
| 5 | C sp3 | H3S | -0.04 | 11 | 11 |
| 6 | C sp3 | H2C2 | 0.06 | 908 | 303 |
| 7 | C sp3 | H2CN | -0.10 | 594 | 260 |
| 8 | C sp3 | H2CN(+) | -0.92 | 7 | 3 |
| 9 | C sp3 | H2CO | -0.28 | 115 | 92 |
| 10 | C sp3 | H2CS | 0.00 | 35 | 22 |
| 11 | C sp3 | H2CF | 0.28 | 7 | 7 |
| 12 | C sp3 | H2CCl | -0.19 | 13 | 8 |
| 13 | C sp3 | H2CBr | -0.06 | 1 | 1 |
| 14 | C sp3 | H2N2 | -0.31 | 8 | 8 |
| 15 | C sp3 | H2NO | 0.41 | 2 | 2 |
| 16 | C sp3 | H2O2 | -0.71 | 3 | 3 |
| 17 | C sp3 | H2OF | -0.44 | 1 | 1 |
| 18 | C sp3 | HC3 | 0.03 | 179 | 111 |
| 19 | C sp3 | HC2N | -0.07 | 175 | 115 |
| 20 | C sp3 | HC2N(+) | -0.41 | 4 | 4 |
| 21 | C sp3 | HC2O | -0.15 | 125 | 79 |
| 22 | C sp3 | HC2S | 0.61 | 1 | 1 |
| 23 | C sp3 | HC2F | -0.17 | 2 | 2 |
| 24 | C sp3 | HC2Br | 0.22 | 1 | 1 |
| 25 | C sp3 | HCN2 | -0.19 | 2 | 2 |
| 26 | C sp3 | HCNO | -0.18 | 11 | 11 |
| 27 | C sp3 | HCNS | 0.52 | 1 | 1 |
| 28 | C sp3 | HCO2 | -0.60 | 7 | 4 |
| 29 | C sp3 | HCOCl | -0.26 | 1 | 1 |
| 30 | C sp3 | HCS2 | 0.00 | 1 | 1 |
| 31 | C sp3 | HCFCl | -0.44 | 1 | 1 |
| 32 | C sp3 | HCFBr | -0.14 | 1 | 1 |
| 33 | C sp3 | HCCl2 | -0.34 | 3 | 3 |
| 34 | C sp3 | HCClBr | -0.06 | 1 | 1 |
| 35 | C sp3 | HOF2 | -0.02 | 3 | 3 |
| 36 | C sp3 | C4 | -0.17 | 82 | 64 |
| 37 | C sp3 | C3N | -0.29 | 19 | 19 |
| 38 | C sp3 | C3N(+) | -0.43 | 2 | 2 |
| 39 | C sp3 | C3O | 0.02 | 43 | 40 |
| 40 | C sp3 | C3S | -0.46 | 4 | 2 |
| 41 | C sp3 | C2NO | -0.76 | 3 | 3 |
| 42 | C sp3 | C2O2 | -0.21 | 2 | 2 |
| 43 | C sp3 | C2F2 | -0.87 | 1 | 1 |
| 44 | C sp3 | CNO2 | -0.38 | 2 | 2 |
| 45 | C sp3 | COF2 | 0.20 | 2 | 2 |
| 46 | C sp3 | CF3 | 0.20 | 19 | 17 |
| 47 | C sp3 | CCl3 | 0.22 | 2 | 2 |
| 48 | C sp3 | OF3 | -0.23 | 5 | 5 |
| 49 | C sp2 | H2=C | 0.06 | 21 | 17 |
| 50 | C sp2 | HC=C | -0.05 | 148 | 82 |
| 51 | C sp2 | HC=N | -0.70 | 2 | 2 |
| 52 | C sp2 | H=CN(+) | -0.64 | 1 | 1 |
| 53 | C sp2 | HC=N(+) | -0.25 | 3 | 3 |
| 54 | C sp2 | H=CN | -0.37 | 53 | 47 |
| 55 | C sp2 | H=CO | 0.03 | 7 | 5 |
| 56 | C sp2 | H=CS | -0.36 | 14 | 14 |
| 57 | C sp2 | H=CF | 0.08 | 2 | 1 |
| 58 | C sp2 | H=CCl | -0.11 | 6 | 4 |
| 59 | C sp2 | H=CJ | 1.46 | 1 | 1 |
| 60 | C sp2 | HN=N | -0.13 | 32 | 24 |
| 61 | C sp2 | HN=O | -0.37 | 1 | 1 |
| 62 | C sp2 | H=NS | -0.33 | 1 | 1 |
| 63 | C sp2 | C2=C | -0.01 | 69 | 55 |
| 64 | C sp2 | C2=N | 0.36 | 26 | 21 |
| 65 | C sp2 | C2=O | -0.20 | 46 | 39 |
| 66 | C sp2 | C=CN | -0.30 | 72 | 60 |
| 67 | C sp2 | C=CN(+) | 0.27 | 7 | 7 |
| 68 | C sp2 | C=CO | -0.20 | 19 | 12 |
| 69 | C sp2 | C=CS | 0.09 | 4 | 4 |
| 70 | C sp2 | C=CF | 0.00 | 1 | 1 |
| 71 | C sp2 | C=CCl | 0.48 | 1 | 1 |
| 72 | C sp2 | C=CBr | 0.27 | 1 | 1 |
| 73 | C sp2 | C=CJ | -0.05 | 1 | 1 |
| 74 | C sp2 | =CN2 | -0.10 | 17 | 17 |
| 75 | C sp2 | CN=N | -0.14 | 28 | 26 |
| 76 | C sp2 | CN=O | -0.24 | 190 | 138 |
| 77 | C sp2 | C=NO | 0.00 | 4 | 4 |
| 78 | C sp2 | C=NS | -0.97 | 9 | 9 |
| 79 | C sp2 | =CNS | -0.23 | 1 | 1 |
| 80 | C sp2 | =CNBr | -0.53 | 1 | 1 |
| 81 | C sp2 | =COS | 0.30 | 1 | 1 |
| 82 | C sp2 | =CS2 | 1.80 | 1 | 1 |
| 83 | C sp2 | CO=O | 0.00 | 76 | 64 |
| 84 | C sp2 | CO=O(-) | 0.61 | 7 | 6 |
| 85 | C sp2 | =CSJ | -0.36 | 1 | 1 |
| 86 | C sp2 | =CF2 | -0.12 | 1 | 1 |
| 87 | C sp2 | =CCl2 | 0.11 | 3 | 2 |
| 88 | C sp2 | N2=N | -0.49 | 28 | 26 |
| 89 | C sp2 | N2=N(+) | 0.29 | 3 | 3 |
| 90 | C sp2 | N2=O | 0.03 | 42 | 41 |
| 91 | C sp2 | N=NO | 0.00 | 1 | 1 |
| 92 | C sp2 | N2=S | -0.08 | 4 | 4 |
| 93 | C sp2 | N=NS | -0.08 | 18 | 18 |
| 94 | C sp2 | NO=O | 0.13 | 5 | 5 |
| 95 | C aromatic | H:C2 | 0.01 | 1970 | 341 |
| 96 | C aromatic | H:C:N | -0.13 | 41 | 29 |
| 97 | C aromatic | H:C:N(+) | -0.06 | 2 | 1 |
| 98 | C aromatic | :C3 | 0.01 | 23 | 13 |
| 99 | C aromatic | C:C2 | 0.03 | 508 | 286 |
| 100 | C aromatic | C:C:N | -0.21 | 30 | 26 |
| 101 | C aromatic | :C2N | -0.06 | 204 | 159 |
| 102 | C aromatic | :C2N(+) | -0.16 | 3 | 3 |
| 103 | C aromatic | :C2:N | 0.15 | 8 | 8 |
| 104 | C aromatic | :C2O | 0.14 | 182 | 127 |
| 105 | C aromatic | :C2S | -0.03 | 53 | 32 |
| 106 | C aromatic | :C2F | 0.08 | 33 | 29 |
| 107 | C aromatic | :C2Cl | 0.17 | 71 | 57 |
| 108 | C aromatic | :C2Br | 0.24 | 17 | 14 |
| 109 | C aromatic | :C2J | 0.59 | 5 | 5 |
| 110 | C aromatic | :CN:N | 0.22 | 14 | 13 |
| 111 | C aromatic | C:N2 | 1.26 | 1 | 1 |
| 112 | C aromatic | :C:N2 | -0.19 | 1 | 1 |
| 113 | C aromatic | :C:NCl | 1.06 | 1 | 1 |
| 114 | C aromatic | N:N2 | 0.58 | 6 | 6 |
| 115 | C aromatic | :N3 | 0.86 | 1 | 1 |
| 116 | C sp | H#C | -0.05 | 2 | 2 |
| 117 | C sp | C#C | -0.10 | 6 | 4 |
| 118 | C sp | C#N | -0.68 | 8 | 8 |
| 119 | C sp | N#N | -0.66 | 4 | 4 |
| 120 | N sp3 | H2C | 0.23 | 12 | 12 |
| 121 | N sp3 | H2C(pi) | -0.21 | 48 | 38 |
| 122 | N sp3 | H2N | -0.45 | 1 | 1 |
| 123 | N sp3 | HC2 | 0.33 | 86 | 66 |
| 124 | N sp3 | HC2(pi) | -0.06 | 108 | 73 |
| 125 | N sp3 | HC2(2pi) | -0.02 | 106 | 87 |
| 126 | N sp3 | HCN | 0.00 | 1 | 1 |
| 127 | N sp3 | HCN(2pi) | -0.86 | 1 | 1 |
| 128 | N sp3 | HCS | -0.75 | 1 | 1 |
| 129 | N sp3 | HCS(pi) | -0.82 | 3 | 3 |
| 130 | N sp3 | C3 | 0.39 | 143 | 133 |
| 131 | N sp3 | C3(pi) | 0.16 | 76 | 61 |
| 132 | N sp3 | C3(2pi) | -0.03 | 70 | 62 |
| 133 | N sp3 | C3(3pi) | 0.02 | 23 | 23 |
| 134 | N sp3 | C2N(pi) | 0.07 | 4 | 4 |
| 135 | N sp3 | C2N(2pi) | 0.08 | 24 | 20 |
| 136 | N sp3 | C2N(3pi) | -0.22 | 8 | 8 |
| 137 | N sp3 | C2O(2pi) | 0.14 | 1 | 1 |
| 138 | N sp3 | C2S | -0.06 | 2 | 2 |
| 139 | N sp3 | C2S(pi) | -1.24 | 2 | 2 |
| 140 | N sp3 | C2S(2pi) | -1.42 | 1 | 1 |
| 141 | N(+) sp3 | H3C | -0.90 | 4 | 4 |
| 142 | N(+) sp3 | HC3 | 0.13 | 2 | 2 |
| 143 | N sp2 | H=C | 0.00 | 2 | 2 |
| 144 | N sp2 | C=C | 0.09 | 113 | 93 |
| 145 | N sp2 | C=N | -0.35 | 1 | 1 |
| 146 | N sp2 | C=N(+) | 0.01 | 1 | 1 |
| 147 | N sp2 | =CN | -0.05 | 29 | 19 |
| 148 | N sp2 | =CO | -0.75 | 8 | 8 |
| 149 | N sp2 | N=N | -0.14 | 7 | 4 |
| 150 | N sp2 | N=O | -0.19 | 1 | 1 |
| 151 | N(+) sp2 | C=CO(-) | 0.25 | 2 | 2 |
| 152 | N(+) sp2 | CO=O(-) | -0.22 | 15 | 15 |
| 153 | N aromatic | H2:C(+) | 0.46 | 2 | 1 |
| 154 | N aromatic | HC:C(+) | 0.00 | 1 | 1 |
| 155 | N aromatic | :C2 | -0.18 | 54 | 41 |
| 156 | N aromatic | :C:N | -0.05 | 2 | 1 |
| 157 | N(+) aromatic | :C2O(-) | -0.27 | 1 | 1 |
| 158 | N(+) sp | =C=N(-) | 0.16 | 1 | 1 |
| 159 | N(+) sp | =N2(-) | 0.00 | 1 | 1 |
| 160 | O | HC | -0.02 | 137 | 102 |
| 161 | O | HC(pi) | -0.44 | 69 | 51 |
| 162 | O | HS | -3.96 | 1 | 1 |
| 163 | O | C2 | 0.29 | 58 | 46 |
| 164 | O | C2(pi) | 0.03 | 168 | 125 |
| 165 | O | C2(2pi) | -0.32 | 26 | 26 |
| 166 | O | CN | 0.39 | 1 | 1 |
| 167 | O | CN(pi) | 0.37 | 2 | 2 |
| 168 | O | CN(2pi) | -0.06 | 6 | 6 |
| 169 | S2 | C2 | 0.17 | 15 | 15 |
| 170 | S2 | C2(pi) | -0.47 | 12 | 10 |
| 171 | S2 | C2(2pi) | 0.64 | 41 | 40 |
| 172 | S2 | CS | -0.54 | 2 | 1 |
| 173 | S4 | C2=O | -0.89 | 3 | 3 |
| 174 | S4 | C2=O2 | -0.84 | 2 | 2 |
| 175 | S4 | CN=O2 | -0.12 | 9 | 9 |
| 176 | S4 | CO=O2 | 0.00 | 1 | 1 |
| **A** | **Based on** |  |  |  | **486** |
| **B** | **Goodness of fit** | **R^2** | **0.6991** |  | **413** |
| **C** | **Deviation** | **Average** | **0.30** |  | **413** |
| **D** | **Deviation** | **Standard** | **0.39** |  | **413** |
| **E** | **K-fold cv** | **K** | **10.00** |  | **385** |
| **F** | **Goodness of fit** | **Q^2** | **0.4786** |  | **385** |
| **G** | **Deviation** | **Average (cv)** | **0.40** |  | **385** |
| **H** | **Deviation** | **Standard (cv)** | **0.52** |  | **385** |
